# Supplementary material for: Disrupted host-microbiota crosstalk promotes nonalcoholic fatty liver disease progression by impaired mitophagy
Source: Microbiol Spectr. 2025 May 22;13(7):e00100-25. doi: 10.1128/spectrum.00100-25 (PMC12211054; doi:10.1128/spectrum.00100-25)
Supplement: Supplemental material — Fig. S1 to S15 and legends for Tables S1 to S17. [file spectrum.00100-25-s0001.pdf]

Supplementary Material for

Disrupted Host-Microbiota Crosstalk Promotes Nonalcoholic Fatty Liver Disease Progression by Impaired Mitophagy

Wenjing Yin[1, #], Wenxing Gao[1, #], Yuwei Yang[1], Weili Lin[1], Wanning Chen[1], Xinyue Zhu[1], Ruixin Zhu[1, *], Lixin Zhu[2, *], Na Jiao[3, *]

Correspondence to: najiao@fudan.edu.cn

This PDF file includes:

Supplementary Figure 1-15

Captions for Supplementary Table 1-17

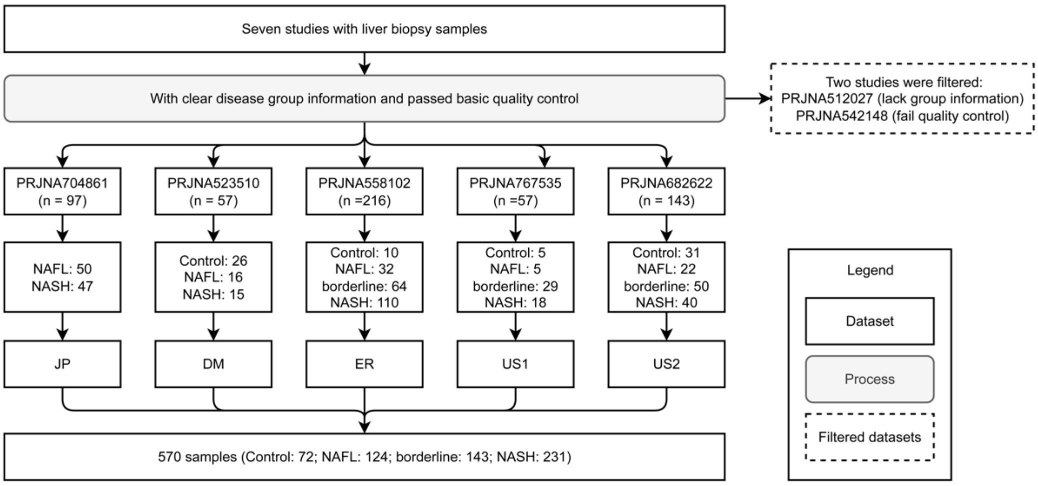

Figure S1. Discovery cohort collection and sample filtering criteria

Seven studies were initially collected with public RNA data. Study PRJNA512027 was filtered for lacking clear clinical diagnostic standard declaration. Another study PRJNA542148 was excluded for not passing the basic quality control standard, as the average qualified read count was less than 20 million. At last, five cohorts of 570 samples were investigated for further analyses.

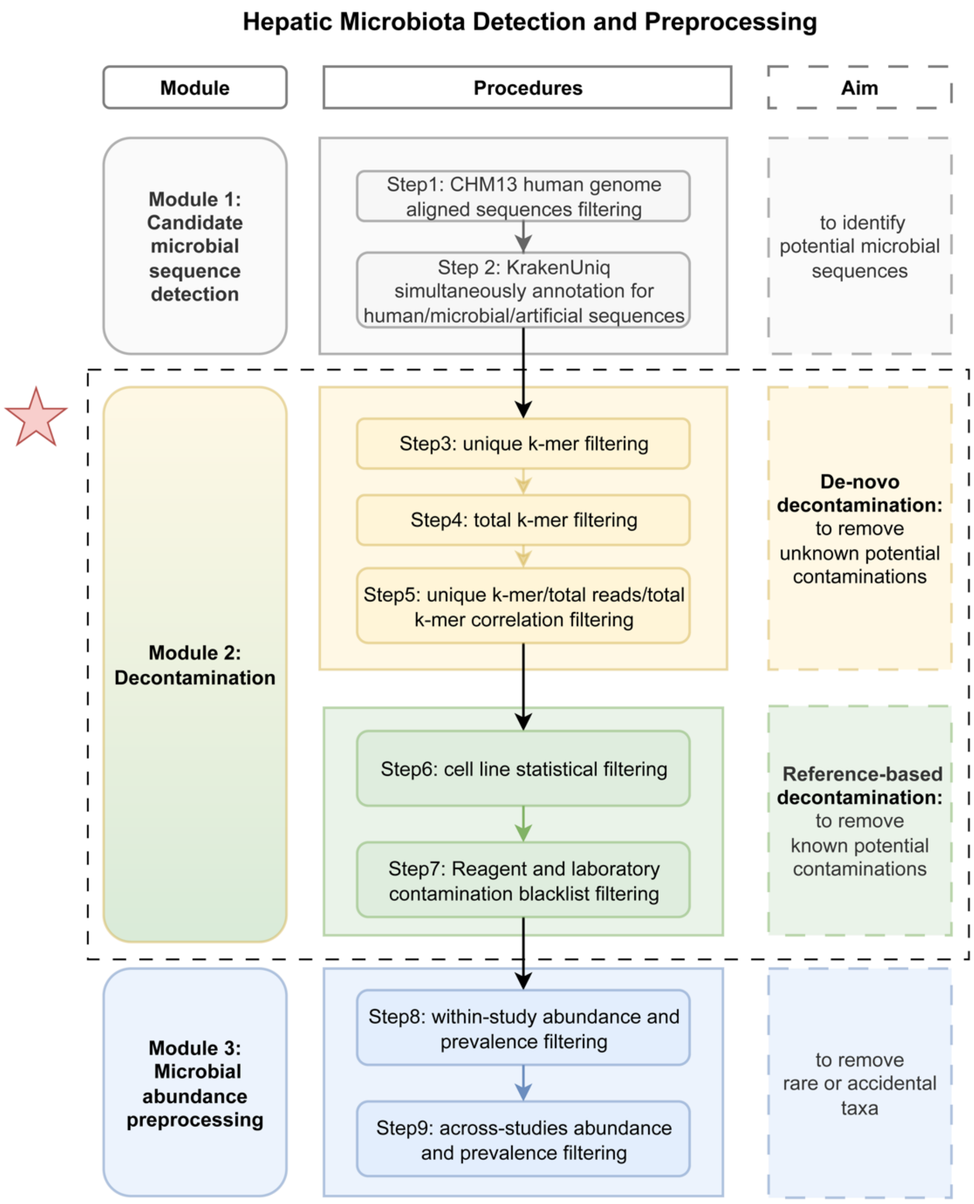

Figure S2. Procedures of hepatic microbiota reads detection

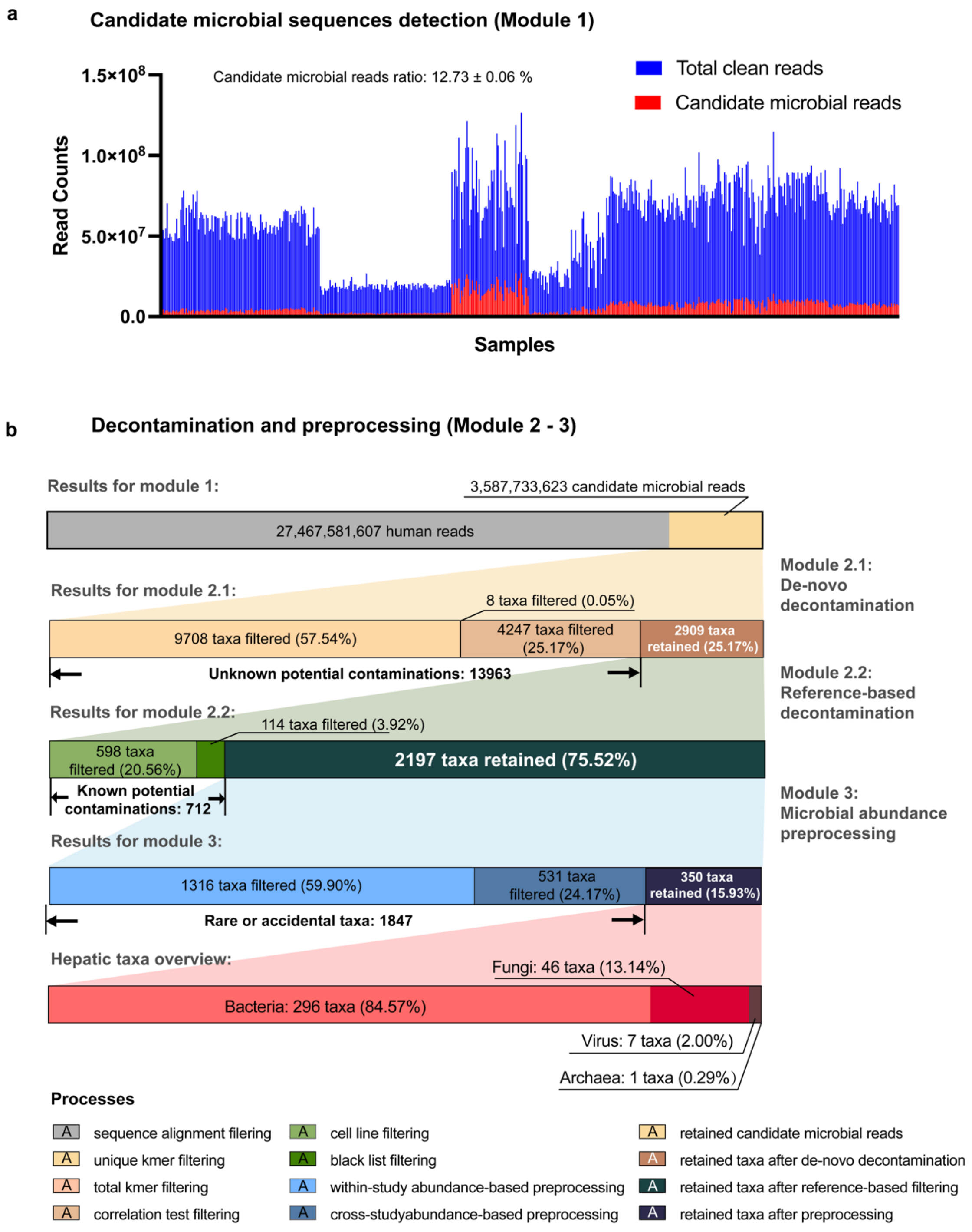

Figure S3. Statistics of hepatic microbiota reads detection

a Statistics after candidate microbial sequences detection process. **b** Statistics after decontamination and remaining preprocessing.

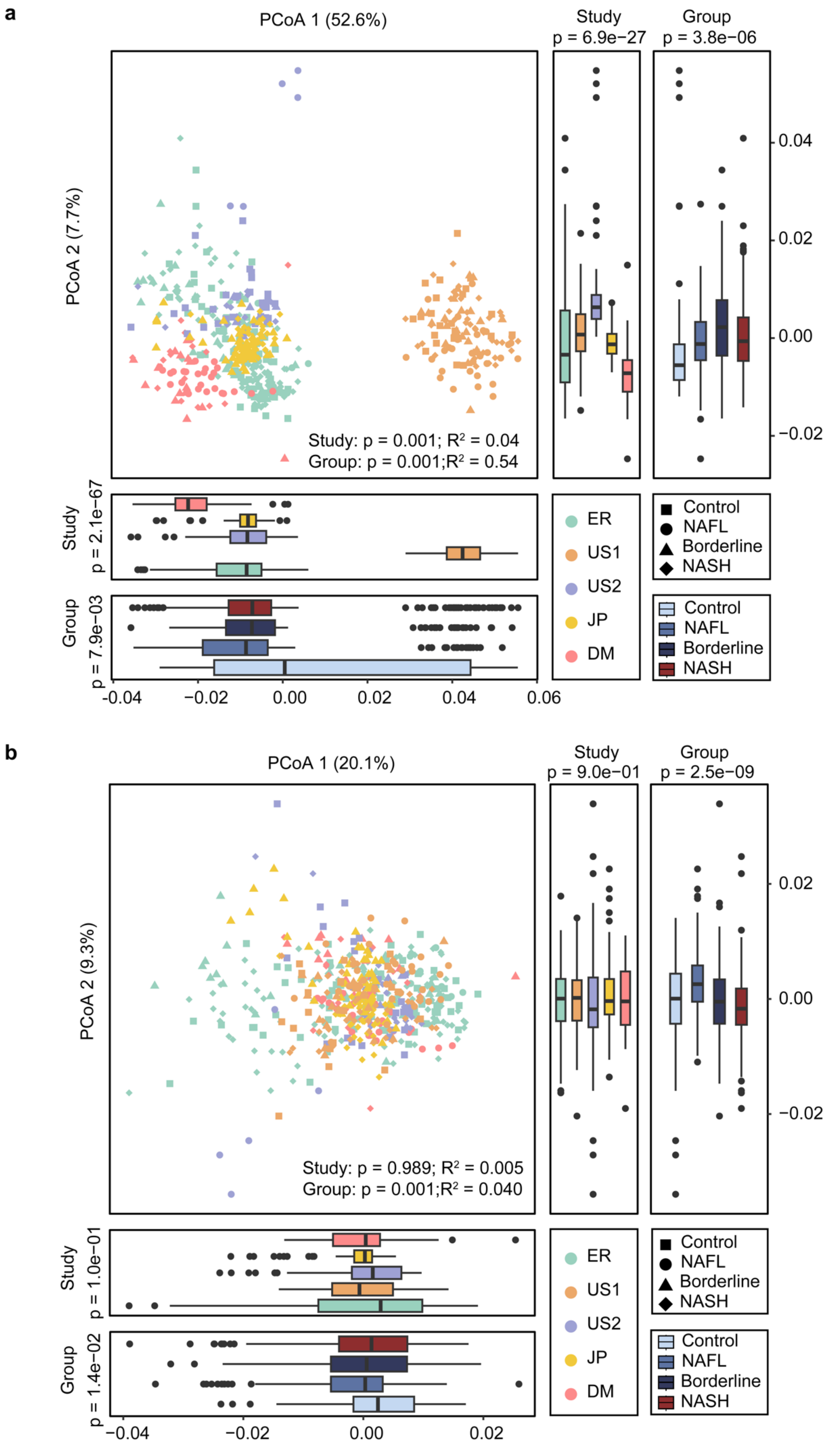

a
PCoA 1 (52.6%)
PCoA 2 (7.7%)
Study
p = 6.9e−27
Group
p = 3.8e−06
Study: p = 0.001; R² = 0.04
Group: p = 0.001;R² = 0.54
Study
p = 2.1e−67
Group
p = 7.9e−03
ER
US1
US2
JP
DM
Control
NAFL
Borderline
NASH
b
PCoA 1 (20.1%)
PCoA 2 (9.3%)
Study
p = 9.0e−01
Group
p = 2.5e−09
Study: p = 0.989; R² = 0.005
Group: p = 0.001;R² = 0.040
Study
p = 1.0e−01
Group
p = 1.4e−02

Figure S4. Batch effect detection and removal for host gene profile

a Principal coordinate analysis (PCoA) of samples from all five studies based on Bray–Curtis distance, which shows the host gene expression was different among studies ($P = 0.001$, $R^2 = 0.04$) and stages ($P = 0.001$, $R^2 = 0.54$) before batch effect correction. P values were calculated with PERMANOVA. Colors of the dots represented studies while shapes stood for the stages (control, NAFL, borderline and NASH). The upper-right and the bottom-left boxplots illustrate that samples projected onto the first two principal coordinates broken down by study and stages, respectively. P values of the first and second principal components were calculated with a two-sided Kruskal–Wallis test for study and stage. All boxplots represent the interquartile range (25th–75th percentile) of the distribution, with the median indicated by a thick line at the center of the box; the whiskers extend up to values within 1.5 times of IQR, and outliers are represented as dots. **b** After batch effect adjustment, PCoA plot of samples based on Bray–Curtis distance of host gene expression, shows a non-significant p value contributed by study ($P = 0.989$, $R^2 = 0.005$), while the difference contributed by stage remained statistically significant ($P = 0.001$, $R^2 = 0.040$).

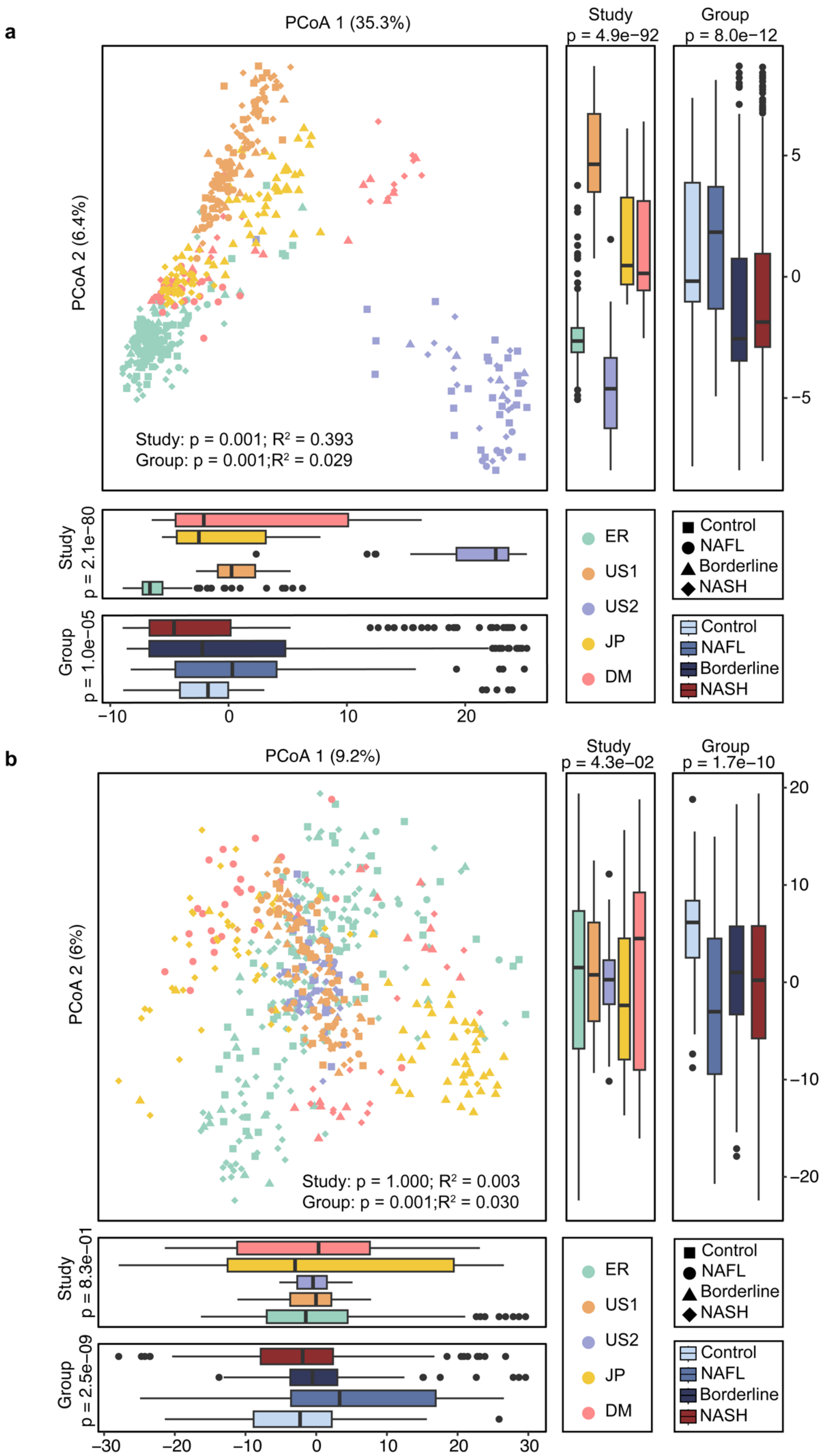
a
PCoA 1 (35.3%)
PCoA 2 (6.4%)
Study: p = 0.001; R² = 0.393
Group: p = 0.001;R² = 0.029
Study
p = 4.9e−92
Group
p = 8.0e−12
5
0
−5
Study
p = 2.1e−80
Group
p = 1.0e−05
−10
0
10
20
ER
US1
US2
JP
DM
Control
NAFL
Borderline
NASH
Control
NAFL
Borderline
NASH
b
PCoA 1 (9.2%)
PCoA 2 (6%)
Study: p = 1.000; R² = 0.003
Group: p = 0.001;R² = 0.030
Study
p = 4.3e−02
Group
p = 1.7e−10
20
10
0
−10
−20
Study
p = 8.3e−01
Group
p = 2.5e−09
−30
−20
−10
0
10
20
30
ER
US1
US2
JP
DM
Control
NAFL
Borderline
NASH
Control
NAFL
Borderline
NASH

Figure S5. Batch effect detection and removal for hepatic microbiota profile

a Principal coordinate analysis (PCoA) of samples from all five studies based on Aitchison distance of hepatic microbial abundance before batch effect correction, which shows difference among studies (P = 0.001, R^2 = 0.0393) and stages (P = 0.001, R^2 =0.029). P values were calculated with PERMANOVA. Colors of the dots represented studies while shapes stood for the stages (control, NAFL, borderline and NASH). The upper-right and the bottom-left boxplots illustrate that samples projected onto the first two principal coordinates broken down by study and stages, respectively. P values of the first and second principal components were calculated with a two-sided Kruskal–Wallis test for study and stage. All boxplots represent the interquartile range (25th–75th percentile) of the distribution, with the median indicated by a thick line at the center of the box; the whiskers extend up to values within 1.5 times of IQR, and outliers are represented as dots. **b** After batch effect adjustment, PCoA plot of samples based on Aitchison distance of hepatic microbial abundance, shows a non-significant p value contributed by study (P = 1.000, R^2 =0.003), while the difference contributed by stage remained statistically significant (P = 0.001, R^2 =0.030).

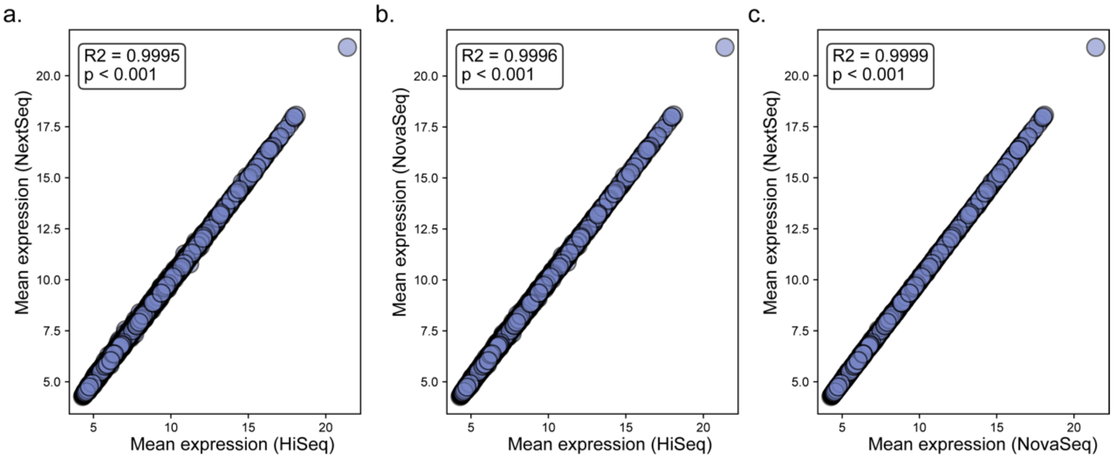

Figure S6. High consistency of gene expression across different sequencing platforms.

Scatter plots showed that gene expression showed high agreement between NextSeq and HiSeq (a), NovaSeq and HiSeq (b), NextSeq and NovaSeq (c).

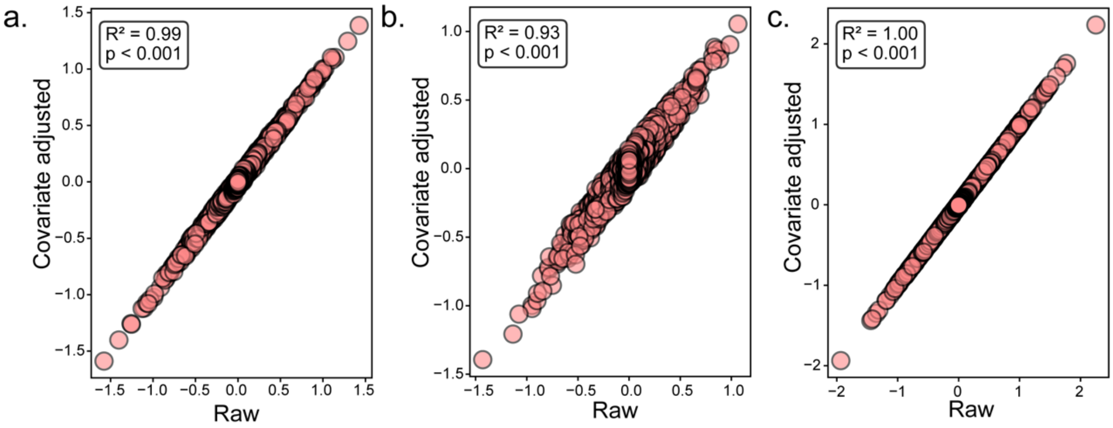

Figure S7. The influence of adjusting covariates to gene expression fold change of diseased stages compared to healthy controls.

Scatter plots showed that, compared to the healthy control, the fold change of gene expression in NAFL (a), Borderline (b) and NASH (c) remained stable after adjusting for covariates including

gene coverage per sample, number of clean reads after trimming, sequencing platform, RNA extraction method, and library preparation method.

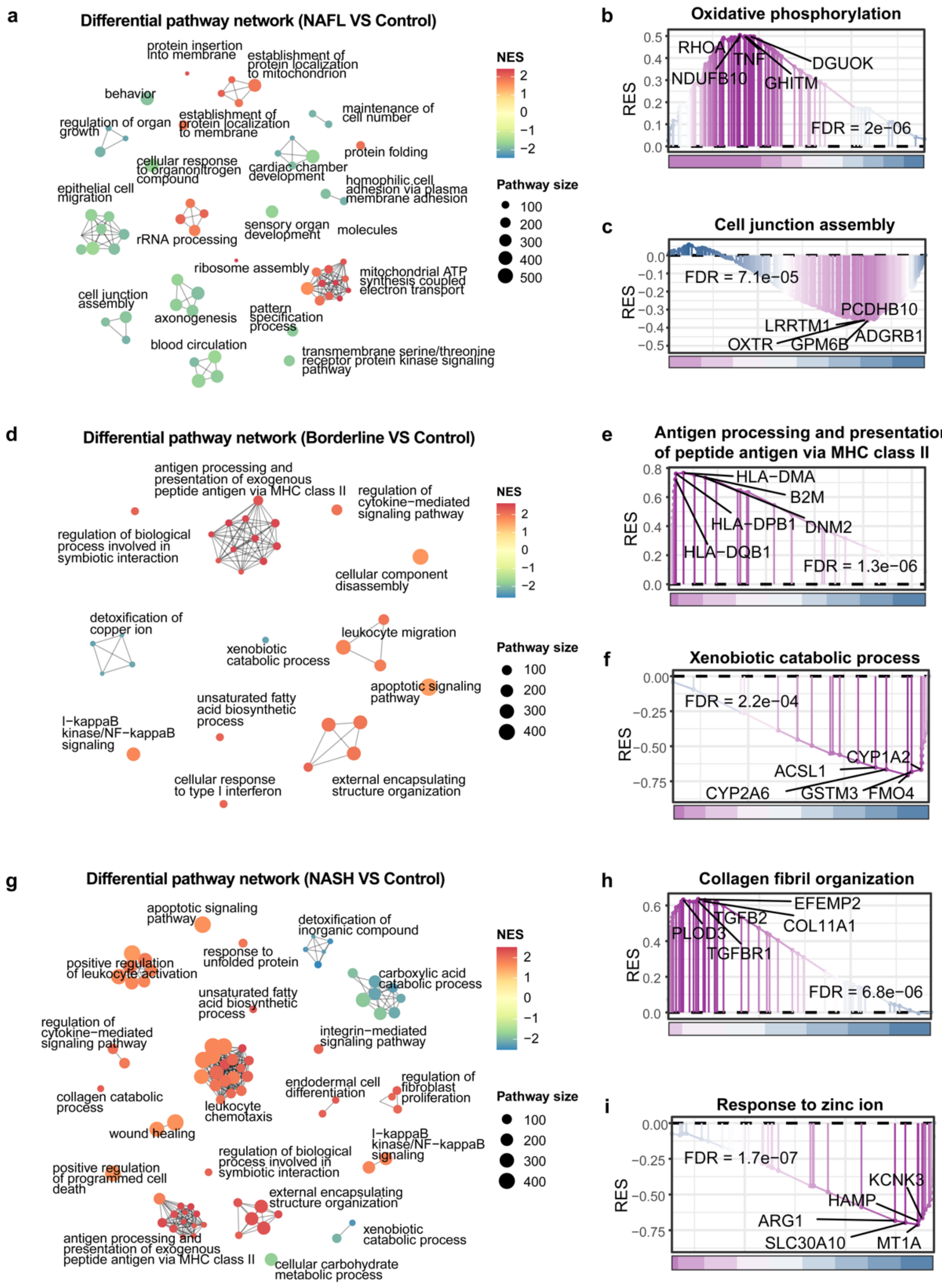

Figure S8. GSEA Pathway enrichment results of differentially expressed genes

a Enrichment pathway plot showing enriched pathways in NAFL compared to control, enriched by gene set enrichment analysis (GSEA). Each dot represents a pathway; the dot size correlates with the number of genes in the pathway; the dot color represents the Normalized Enrichment Score (NES) of the pathway. Similar pathways were connected into clusters. The most significant pathway in a cluster was chosen as the representative of that cluster and was annotated on the plot. **b**

Oxidative phosphorylation pathway was further visualized as the representative up-regulated pathway in NAFL compared to control. GSEA plot shows the genes (x-axis) that in this pathway in the order of descending log Fold Change and their Running Enrichment Score (RES) (y-axis). The most five influential genes with the highest RES were annotated in the plot. The color of the bar demonstrating for the RES of pathway-related genes, with purple stands for an absolute RES higher than 0.2 and blue means the opposite. The heatmap below representing the log Fold Change of the gene, with the largest values shown in purple and smallest shown in blue. The FDR of the pathway was annotated in the plot. **c** Cell junction assembly pathway was visualized as the representative down-regulated pathway in NAFL compared to control. **d** Enrichment pathway plot showing enriched pathways in borderline compared to control. **e** Antigen processing and presentation of peptide antigen via MHC class II pathway was visualized as the representative up-regulated pathway in borderline compared to control. **f** Xenobiotic catabolic process pathway was visualized as the representative down-regulated pathway in borderline compared to control. **g** Enrichment pathway plot showing enriched pathways in NASH compared to control. **h** Collagen fibril organization pathway was visualized as the representative up-regulated pathway in NASH compared to control. **i** Response to zinc ion pathway was visualized as the representative down-regulated pathway in NASH compared to control.

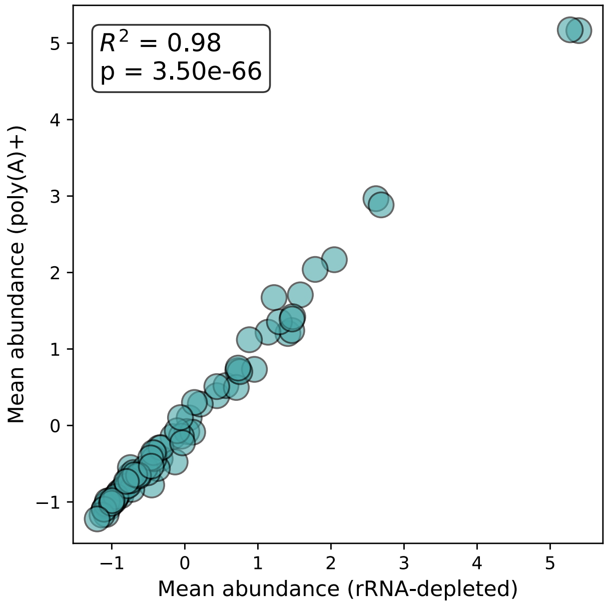

Figure S9. High consistency of hepatic microbial abundance across different RNA library preparation methods.

Scatter plots showed that hepatic microbial abundance showed high agreement between polyA enrichment and rRNA depletion library preparation.

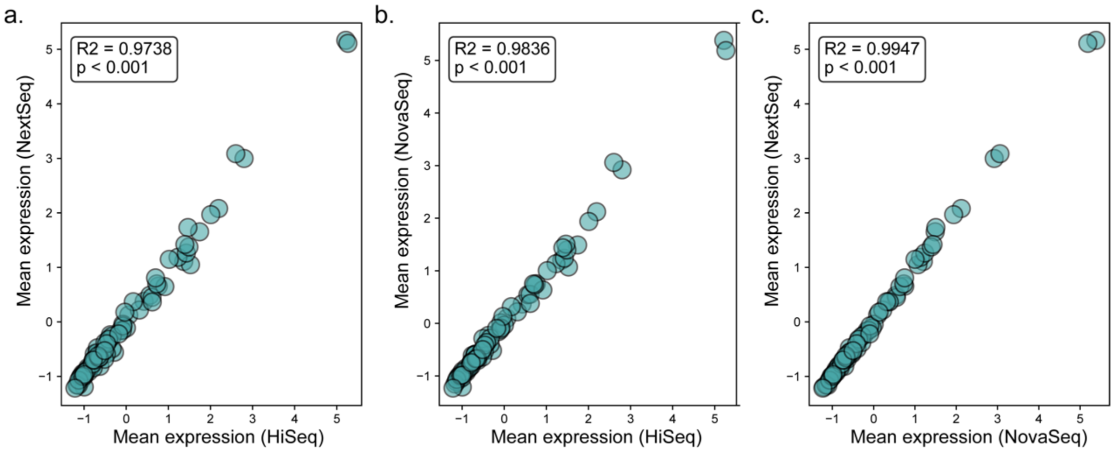

Figure S10. High consistency of hepatic microbial abundance across different sequencing platforms.

Scatter plots showed that hepatic microbial abundance showed high agreement between NextSeq and HiSeq (a), NovaSeq and HiSeq (b), NextSeq and NovaSeq (c).

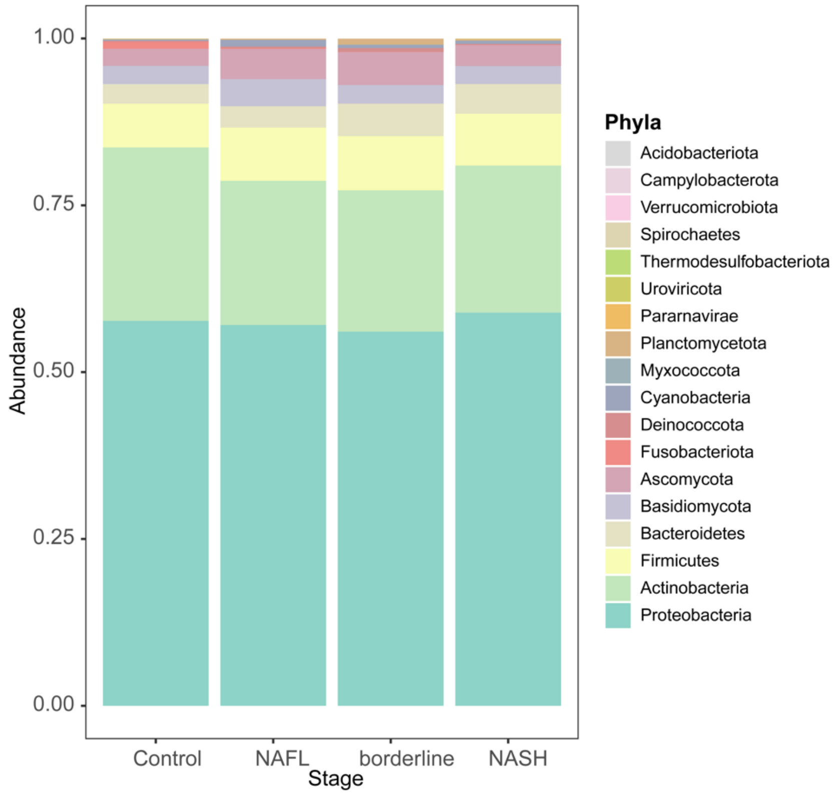

Figure S11. Phyla abundances of hepatic microbes among different stages

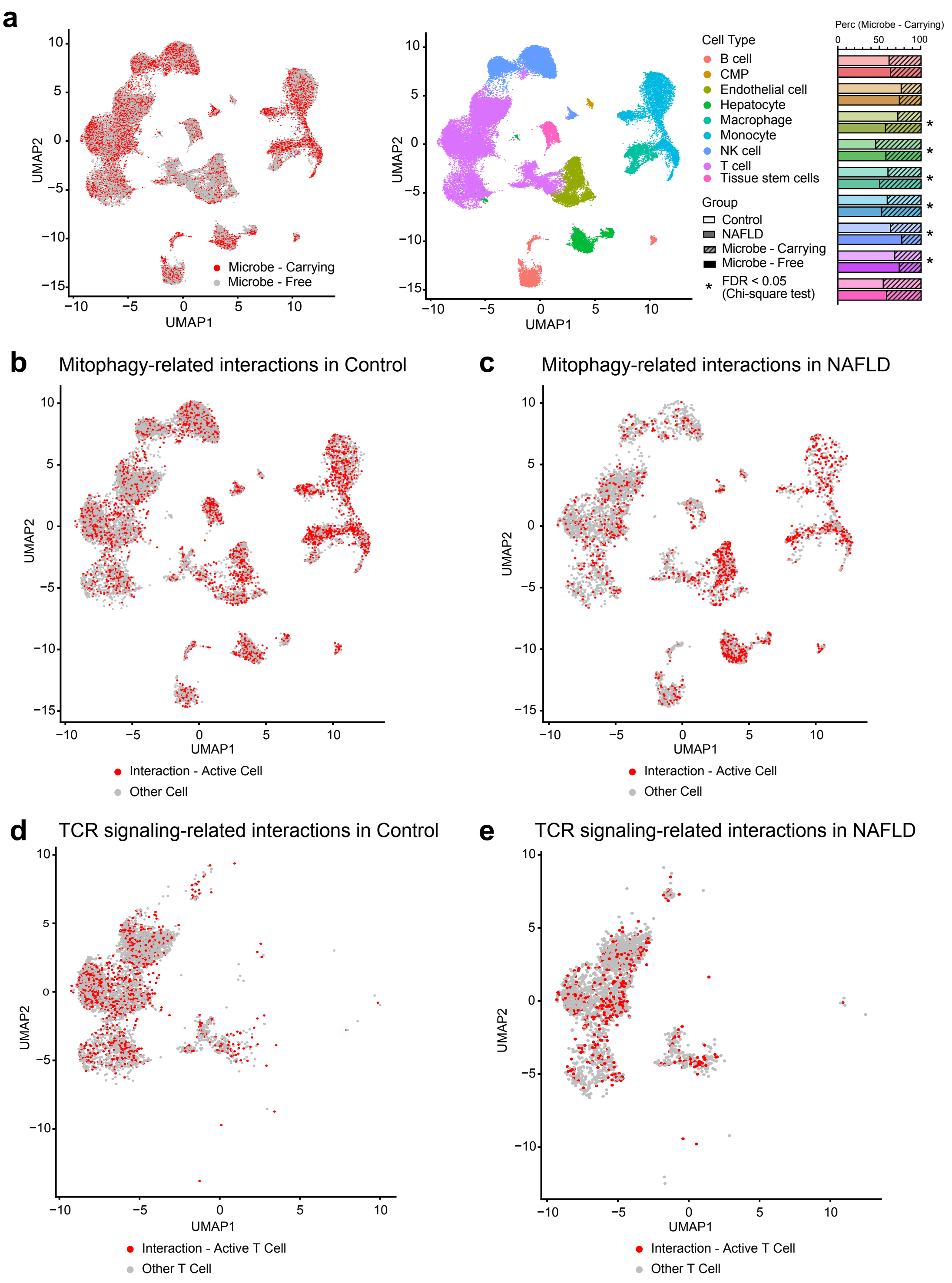

Figure S12. Hepatic microbiota in single cell validation data. a Hepatic microbiota distribution in cells, cell type annotations, and the percentage of microbes-carrying cells in each cell type in control samples and NAFLD patients. **b-c** Distribution of cells which express highly of mitophagy pathway related genes and interacted microbes in control (**b**) and NAFLD (**c**). **d-e**

Distribution of cells which express highly of T cell receptor pathway related genes and interacted microbes in control (**d**) and NAFLD (**e**).

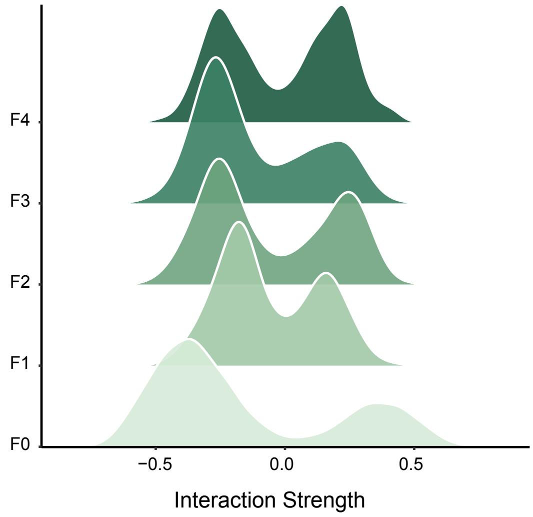

Figure S13. Interaction strength distribution in bulk validation data

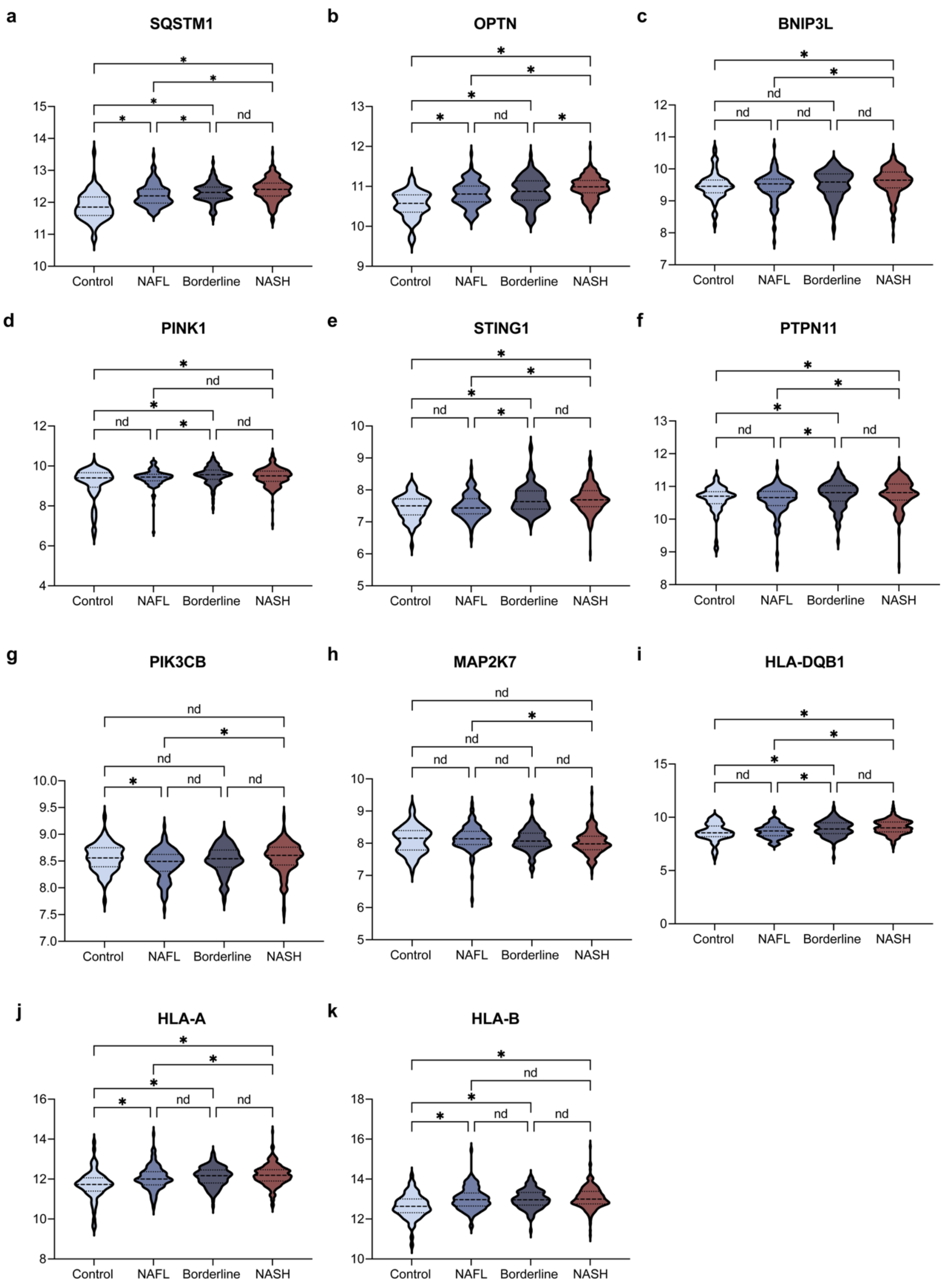

Figure S14. Expression of genes relating to mitophagy (a-d), xenophagy (e) and T cell receptor signaling pathway (f-h). P values of the expression difference in stages were calculated with a two-sided Kruskal–Wallis coupled with multiple comparison tests, with FDR values evaluated using post hoc Bonferroni correction (*FDR < 0.05, nd: not detected for any statistical significance)

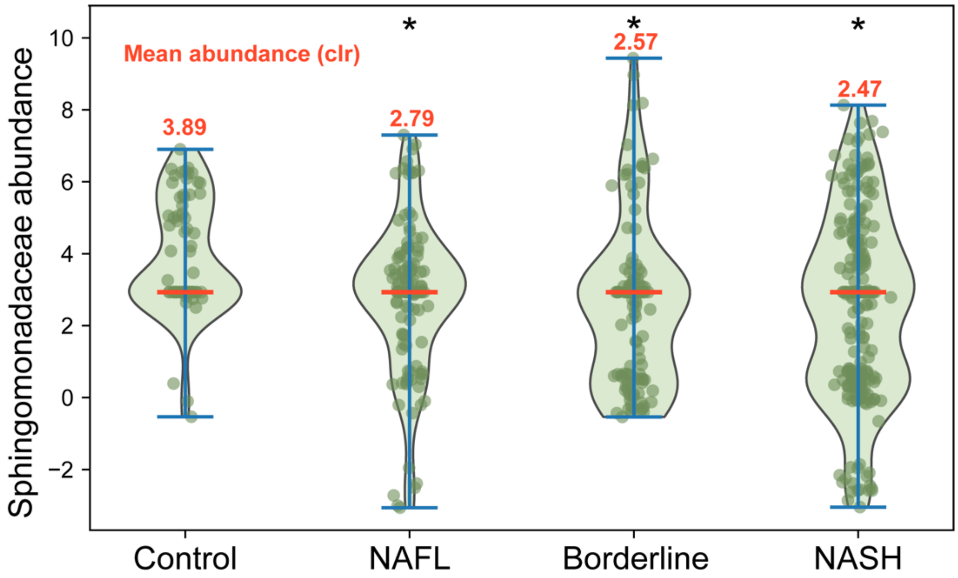

Figure S15. Abundance of Sphingomonadaceae in different stages. Mean abundance of samples in each group was annotated in orange, p value was calculated by U test. *: $p < 0.05$

Captions for Supplementary Tables 1-17:

Supplementary Table 1. Studies collected in this research and demographic details

Supplementary Table 2. Differentially expressed genes list of NAFL vs Control

Supplementary Table 3. Differentially expressed genes list of Borderline vs Control

Supplementary Table 4. Differentially expressed genes list of NASH vs Control

Supplementary Table 5. Gene Set Enrichment Analysis results of NAFL vs Control

Supplementary Table 6. Gene Set Enrichment Analysis results of Borderline vs Control

Supplementary Table 7. Gene Set Enrichment Analysis results of NASH vs Control

Supplementary Table 8. Differentially abundant hepatic microbes

Supplementary Table 9. Hepatic microbiota in independent bulk RNA-seq validation data

Supplementary Table 10. Hepatic microbiota in independent single cell RNA-seq validation data

Supplementary Table 11. Host gene-microbiota interaction results by LASSO in Control

Supplementary Table 12. Host gene-microbiota interaction results by LASSO in NAFL

Supplementary Table 13. Host gene-microbiota interaction results by LASSO in Borderline

Supplementary Table 14. Host gene-microbiota interaction results by LASSO in NASH

Supplementary Table 15. Multi-variable regression of interaction strength on various clinical variables

Supplementary Table 16. Functional annotation of host-microbiota interactions by SparceCCA in all stages in discovery data

Supplementary Table 17. Functional annotation of host-microbiota interactions by SparceCCA in all stages in bulk RNA-seq validation data
