# Supplementary material for: Disrupted host-microbiota crosstalk promotes nonalcoholic fatty liver disease progression by impaired mitophagy
Source: Microbiol Spectr. 2025 May 22;13(7):e00100-25. doi: 10.1128/spectrum.00100-25 (PMC12211054; doi:10.1128/spectrum.00100-25)
Supplement: Supplemental tables — Tables S1 to S17. [file spectrum.00100-25-s0002.pdf]

Supplementary table 1: Studies collected in this research and demographic details

Cohort	Group	Sample	Age	Sex		NAS	Fibrosis	BMI	Nation	Publication	RNA Extraction Type	RNA Library Type	Instrument
				Female	Male								
ER	Control	10	\	\	\	0.00±0.00	0.30±0.67	\	France, Germany, Italy, the United Kingdom	Science Translational Medicine, 2020	Column-based	poly(A)+	NextSeq 550
	NAFL	32	\	\	\	1.66±0.48	0.56±1.01	\					
	Borderline	64	\	\	\	3.59±0.49	1.59±1.18	\					
	NASH	110	\	\	\	5.88±0.93	2.28±0.98	\					
US1	Control	5	\	\	\	0.00±0.00	0.00±0.00	\	\	Hepatology Communications,2022	Column-based	poly(A)+	Nova-Seq 6000
	NAFL	5	\	\	\	0.80±0.84	1.40±1.14	\	\				
	Borderline	29	\	\	\	3.65±0.48	1.17±0.76	\	\				
	NASH	18	\	\	\	5.39±0.61	\	\	\				
US1	Control	31	43.68±11.35	28	3	0	0	44.9±5.9	the United State	Scientific Reports,2021	Magnetic bead	rRNA-	HiSeq 3000
	NAFL	22	41.14±12.93	18	3	1.43±0.51	0.38±0.59	\					
	Borderline	50	\	\	\	\	\	\					
	NASH	40	44.68±13.37	24	16	5.75±0.81	1.70±0.85	\					
JP	NAFL	50	50.14±11.56	8	43	\	\	26.6±4.04	Japan	Hepatology, 2021	Column-based	poly(A)+	HiSeq 3000
	NASH	47	58.32±13.84	26	21	\	\	27.6±4.37					
US2	Control	26	\	31	5	\	\	\	the United State	Journal of the Endocrine Society, 2018	Column-based	poly(A)+	HiSeq 2000
	NAFL	16	\	39	14	\	\	\					
	NASH	15	\	\	\	\	\	\	\				

Supplementary table 2: Differentially expressed genes list of NAFL vs Control

Name	logFC	FDR	Name	logFC	FDR	Name	logFC	FDR	Name	logFC	FDR	Name	logFC	FDR	Name	logFC	FDR	Name	logFC	FDR	Name	logFC	FDR
PTPA	0.607	7.32E-26	MED22	0.300	1.46E-09	HDAC9	-0.395	1.27E-07	DOK7	0.347	1.45E-06	IL1RAP	-0.498	9.66E-06	PHTF2	-0.328	4.69E-05	TIMM13	0.297	2.84E-04	OAS1	0.270	1.86E-03
DYRK1A	-0.421	9.06E-24	ERGIC1	0.328	1.51E-09	GAREM1	-0.357	1.29E-07	C1GALT1	-0.324	1.45E-06	AHSG	0.296	9.67E-06	DDR2	-0.309	4.71E-05	TOMM7	0.348	2.87E-04	SLC10A5	-0.277	1.87E-03
TSPAN17	0.478	1.77E-22	DCTPP1	0.355	1.69E-09	SLC4A2	0.329	1.33E-07	TMEM67	-0.267	1.46E-06	PRADC1	0.294	9.70E-06	COL25A1	-0.341	4.77E-05	ANK2	-0.268	2.87E-04	MBNL3	-0.290	1.87E-03
TOR1AIP2	-0.530	1.77E-22	MRPL28	0.377	1.89E-09	CMYA5	-0.564	1.33E-07	BIN1	0.290	1.50E-06	SDF4	0.291	9.84E-06	CRIM1	-0.374	4.81E-05	FEM1C	-0.272	2.93E-04	PARP10	0.317	1.88E-03
DENND1C	0.437	5.83E-20	FAM9B	-0.606	1.91E-09	ZNF407	-0.270	1.35E-07	SLC23A2	-0.455	1.50E-06	CCSER1	-0.328	9.88E-06	METRN	0.393	4.81E-05	NDUFA3	0.322	2.93E-04	MEI4	-0.294	1.88E-03
FNBP1	-0.424	8.68E-18	TMEM109	0.297	1.92E-09	REEP6	0.480	1.36E-07	CUEDC2	0.267	1.51E-06	ZNF518A	-0.333	9.97E-06	GCFC2	-0.269	4.82E-05	BMF	0.295	2.95E-04	CLCN7	0.267	1.93E-03
FNDC5	1.063	1.12E-17	ITPKC	0.319	1.92E-09	CXCL10	0.818	1.41E-07	UNC79	-0.301	1.51E-06	PDE7B	-0.367	1.02E-05	GOLT1A	0.291	4.82E-05	ITGA6	0.280	2.95E-04	NUBP2	0.275	1.93E-03
TMEM129	0.532	1.89E-17	ADIRF	0.545	2.08E-09	SCARB1	0.321	1.42E-07	KANSL1	-0.270	1.52E-06	NBEA	-0.343	1.03E-05	TMEM132E	-0.277	4.83E-05	MS4A7	-0.449	2.95E-04	FGL2	-0.359	1.93E-03
LIX1L	-0.390	1.89E-17	FHIP1A	-0.404	2.10E-09	HAL	-0.675	1.43E-07	RAPH1	-0.422	1.54E-06	PAOX	0.291	1.03E-05	SMOC1	-0.280	4.83E-05	FAT4	-0.276	2.98E-04	DEFB1	0.339	1.95E-03
SLC35B2	0.416	1.94E-17	ZNF236	-0.342	2.16E-09	BAX	0.362	1.43E-07	MIGA2	0.299	1.54E-06	SNAI2	-0.342	1.03E-05	TMEM82	0.336	4.85E-05	PDGFA	0.335	2.98E-04	TM7SF2	0.365	1.99E-03
RPL39	-0.950	2.38E-17	PRKAR1A	-0.417	2.16E-09	MRPS34	0.381	1.52E-07	LAP3	0.293	1.56E-06	TCERG1	-0.282	1.04E-05	RPS2	0.323	4.99E-05	NAMPT	-0.399	2.98E-04	ZNF358	0.285	1.99E-03
TIAM1	-0.511	4.78E-17	PTGR2	-0.265	2.16E-09	HDAC11	0.294	1.58E-07	FOSB	-0.692	1.61E-06	ACADSB	-0.403	1.04E-05	MAP3K1	-0.322	5.01E-05	CD163	-0.575	3.04E-04	LRATD1	-0.361	1.99E-03
CPEB3	-0.705	8.24E-17	SLC29A1	0.386	2.29E-09	FOS	-1.076	1.59E-07	TOB1	-0.367	1.61E-06	MYL6B	0.290	1.05E-05	HLA-B	0.365	5.01E-05	IKZF1	-0.299	3.05E-04	GPR88	-0.488	2.05E-03
CEBPD	-0.949	8.24E-17	MAD2L2	0.374	2.42E-09	ST3GAL6	-0.394	1.61E-07	FRYL	-0.302	1.62E-06	DDX54	0.272	1.06E-05	SEMA6B	-0.294	5.02E-05	FGD2	-0.313	3.08E-04	AOAH	-0.274	2.08E-03
TCF7L2	-0.453	3.94E-16	TPI1	0.326	2.50E-09	CCL14	-0.284	1.64E-07	ST6GALNAC	-0.360	1.62E-06	ABCB8	0.299	1.06E-05	CYP3A7	-0.573	5.11E-05	RBMS3	-0.265	3.13E-04	ACHE	0.340	2.10E-03
CCL15	-0.447	4.71E-16	ENTPD8	0.459	2.54E-09	EXOC8	-0.295	1.64E-07	PIKFYVE	-0.320	1.66E-06	PPARGC1A	-0.577	1.08E-05	FZD6	-0.281	5.12E-05	CETP	-0.562	3.15E-04	LAMTOR4	0.279	2.11E-03
C2CD5	-0.462	1.06E-15	AGMAT	0.338	2.54E-09	LMAN2	0.338	1.64E-07	FNIP2	-0.507	1.66E-06	SLC39A10	-0.301	1.08E-05	UGT2B10	0.342	5.19E-05	PRDX5	0.273	3.17E-04	C9orf152	0.277	2.14E-03
ERN1	-0.604	1.46E-15	TBL1XR1	-0.509	2.56E-09	VPS13A	-0.428	1.65E-07	ZNF217	-0.314	1.68E-06	LEAP2	0.383	1.10E-05	C1orf122	0.295	5.20E-05	MYL5	0.276	3.19E-04	DTNA	0.416	2.15E-03
AJUBA	0.657	4.32E-15	ALG1	0.285	2.67E-09	HECTD1	-0.352	1.66E-07	ABCA9	-0.468	1.69E-06	PGAP1	-0.313	1.11E-05	C8G	0.418	5.21E-05	CDHR2	0.458	3.22E-04	PKHD1L1	-0.480	2.15E-03
RPS10	-0.438	4.32E-15	MAP7	-0.265	2.75E-09	SLC13A3	0.559	1.67E-07	CFAP251	-0.288	1.72E-06	GPCPD1	-0.285	1.13E-05	GPC5	-0.324	5.24E-05	TCAP	0.482	3.24E-04	IFI27	0.447	2.19E-03
PELI2	-0.619	7.93E-15	TNR	-0.506	3.16E-09	KCNJ2	-0.473	1.69E-07	JAK2	-0.326	1.81E-06	CDKN1A	0.544	1.14E-05	VAV3	-0.271	5.28E-05	RANBP17	-0.324	3.24E-04	GGT5	0.278	2.20E-03
PHF23	0.418	8.44E-15	NR2F1	-0.380	3.18E-09	VPS25	0.266	1.69E-07	NPAS3	-0.270	1.85E-06	ANGPT2	-0.379	1.14E-05	CSTB	0.319	5.33E-05	THEMIS2	-0.288	3.26E-04	SLC2A13	-0.286	2.22E-03
SLC12A4	0.436	1.18E-14	TMED9	0.333	3.24E-09	BTAF1	-0.395	1.72E-07	LGALS3BP	0.493	1.85E-06	KLHL3	-0.362	1.16E-05	CERS6	-0.371	5.42E-05	KDM6A	-0.283	3.26E-04	MCTP2	-0.289	2.24E-03
RAC3	0.601	1.91E-14	RBM47	-0.309	3.25E-09	LPCAT1	-0.289	1.72E-07	FANCL	-0.275	1.85E-06	LIPF	-0.317	1.16E-05	ADAMTSL1	-0.291	5.45E-05	SLCO1A2	-0.582	3.30E-04	STK17B	-0.264	2.29E-03
GAPVD1	-0.315	2.57E-14	ADD3	-0.442	3.29E-09	ACP2	0.264	1.72E-07	PAN3	-0.367	1.87E-06	SLC22A7	0.307	1.17E-05	ANXA2	0.399	5.46E-05	SETD1B	-0.305	3.32E-04	FMO1	0.415	2.29E-03
ENO3	0.850	2.57E-14	CADM2	-0.434	3.35E-09	CD59	0.279	1.73E-07	ANXA9	0.268	1.93E-06	TIMP2	-0.373	1.17E-05	RO60	-0.314	5.50E-05	SYNPO	0.276	3.33E-04	NDUFA13	0.284	2.31E-03
BICRAL	-0.350	3.60E-14	PPP1CA	0.299	3.47E-09	ZNF451	-0.289	1.76E-07	TBCEL	-0.320	1.93E-06	INTU	-0.276	1.18E-05	RNPEPL1	0.329	5.56E-05	RPL8	0.283	3.34E-04	RASGRP2	-0.281	2.31E-03
ITSN1	-0.322	4.48E-14	TMEM250	0.312	3.58E-09	MAN1C1	-0.416	1.78E-07	FLT4	-0.463	1.96E-06	ARHGEF4	-0.340	1.18E-05	C5	-0.450	5.56E-05	WNK3	-0.366	3.35E-04	RET	0.420	2.32E-03
SAE1	0.325	5.09E-14	SIL1	0.355	3.60E-09	ATP6V1F	0.300	1.86E-07	DGKH	-0.352	1.98E-06	TUT7	-0.388	1.19E-05	SEMA4D	-0.304	5.57E-05	FNDC3A	-0.315	3.36E-04	APOL5	-0.339	2.42E-03
CD151	0.591	5.09E-14	MARCHF6	-0.319	3.67E-09	TXNDC5	-0.428	1.86E-07	SLC20A1	-0.334	1.99E-06	PGLS	0.315	1.21E-05	B3GAT1	-0.697	5.69E-05	ARHGEF26	-0.300	3.42E-04	SNAP25	0.276	2.50E-03
TIAL1	-0.270	5.89E-14	RNF144A	-0.356	3.69E-09	PYCR3	0.326	1.86E-07	TBX18	-0.277	2.01E-06	ERBB2	0.329	1.23E-05	CPEB2	-0.319	5.76E-05	RASSF6	-0.316	3.43E-04	RPLP1	0.293	2.51E-03
SLC39A7	0.401	7.78E-14	NCOA3	-0.342	3.79E-09	SPSB2	0.352	1.86E-07	PFDN6	0.304	2.01E-06	GET3	0.268	1.23E-05	SCRN2	0.313	5.76E-05	LRP4	-0.288	3.45E-04	MKI67	0.288	2.55E-03
DLC1	-0.387	8.29E-14	CERS4	0.403	3.79E-09	ZDHHC12	0.417	1.90E-07	ANKS1A	-0.266	2.02E-06	TMEM209	-0.280	1.25E-05	IL17RE	0.365	5.78E-05	RORA	-0.415	3.47E-04	MB	0.327	2.62E-03
TMEM102	0.402	1.26E-13	DUSP10	-0.435	4.54E-09	UTRN	-0.444	1.97E-07	KCNMA1	-0.470	2.03E-06	CPS1	-0.438	1.25E-05	H2AC6	0.328	5.85E-05	TNPO1	-0.302	3.52E-04	MT1X	-0.443	2.65E-03
MTR	-0.414	1.26E-13	TEX264	0.310	4.56E-09	GADD45G	-0.750	2.08E-07	EDF1	0.360	2.04E-06	MEGF10	-0.276	1.30E-05	NOTCH4	-0.386	5.86E-05	MMP16	-0.267	3.55E-04	CYP7A1	0.698	2.68E-03
CA13	-0.358	1.49E-13	DICER1	-0.423	4.56E-09	CD38	-0.419	2.12E-07	ENHO	0.452	2.05E-06	FABP1	0.421	1.32E-05	MED13	-0.329	6.05E-05	TLCD4	-0.335	3.55E-04	MAMDC4	0.467	2.81E-03
LRRC20	0.475	1.65E-13	THEM6	0.445	5.03E-09	WASL	-0.283	2.18E-07	MMP14	0.343	2.05E-06	PRAG1	-0.320	1.32E-05	LRRK2	-0.409	6.13E-05	OLFM2	0.621	3.57E-04	ABCA13	-0.287	2.88E-03
ADCK2	0.411	1.65E-13	ZNF460	-0.371	5.47E-09	ZSWIM9	0.288	2.18E-07	MYBL1	-0.332	2.07E-06	CTU2	0.272	1.33E-05	GPRC5A	-0.280	6.15E-05	HERC5	-0.309	3.58E-04	SULT1B1	-0.333	2.89E-03
BCAP31	0.397	1.75E-13	PDS5A	-0.285	5.60E-09	SYNJ1	-0.286	2.18E-07	LTB	0.380	2.13E-06	ZBTB11	-0.271	1.34E-05	ATP6V0B	0.281	6.16E-05	GREB1	-0.314	3.58E-04	CYCS	0.264	2.95E-03
KRT8	0.641	1.83E-13	IL32	0.894	5.60E-09	RFX5	0.277	2.22E-07	PREX2	-0.388	2.14E-06	NAIP	-0.324	1.35E-05	DHRS4	0.402	6.17E-05	MISP3	0.282	3.59E-04	CHAD	-0.310	2.95E-03
ZNF280C	-0.389	2.18E-13	PSMA7	0.358	5.60E-09	CYB5R3	0.324	2.25E-07	INTS2	-0.289	2.17E-06	ATP13A3	-0.422	1.37E-05	NDUFV1	0.266	6.20E-05	NEGR1	-0.304	3.59E-04	AMY2B	-0.271	2.97E-03
PPP1R15B	-0.361	2.18E-13	TRIB2	-0.311	5.63E-09	ASPA	-0.432	2.28E-07	FAM83A	-0.278	2.18E-06	DSC2	-0.286	1.37E-05	MCRIP1	0.354	6.23E-05	STAB1	-0.450	3.60E-04	SOCS3	-0.355	3.04E-03
ATG13	0.271	2.18E-13	EMC4	0.344	5.75E-09	XRN1	-0.445	2.28E-07	GOLIM4	-0.306	2.19E-06	SENP7	-0.329	1.39E-05	C7orf50	0.330	6.24E-05	APOLD1	-0.305	3.61E-04	CD209	-0.368	3.09E-03
SNX11	0.308	2.35E-13	SDC1	0.388	5.90E-09	POU2F1	-0.268	2.28E-07	BBC3	0.336	2.19E-06	HIPK2	-0.315	1.40E-05	IGF2	-0.372	6.52E-05	ARMH4	-0.290	3.61E-04	ZNF334	-0.272	3.10E-03
ENO1	0.399	2.35E-13	RUNDC3B	-0.416	6.07E-09	SIGLEC1	-0.579	2.28E-07	NCAM1	-0.330	2.21E-06	NFKBIZ	-0.375	1.41E-05	TASOR2	-0.267	6.69E-05	RPS7	0.273	3.63E-04	JUND	-0.324	3.14E-03
CREB3	0.372	2.35E-13	DCAF16	-0.308	6.12E-09	B4GALT2	0.306	2.34E-07	MLST8	0.310	2.23E-06	RHOC	0.287	1.41E-05	PALM3	0.385	6.69E-05	CXCL2	-0.537	3.69E-04	CLIP4	-0.270	3.16E-03
IDS	-0.333	3.01E-13	BRMS1	0.367	6.12E-09	TIGD5	0.278	2.39E-07	MAP4K5	-0.269	2.23E-06	OLFM1	-0.321	1.43E-05	CMIP	-0.285	6.79E-05	CCDC57	0.351	3.69E-04	CCN2	-0.360	3.21E-03
ATP6V0D1	0.457	3.06E-13	S100A14	0.501	6.17E-09	SOX5	-0.432	2.45E-07	SNF8	0.277	2.28E-06	EYA4	-0.291	1.44E-05	STAB2	-0.562	6.79E-05	ADRM1	0.269	3.70E-04	SLC16A1	-0.385	3.22E-03
SMAD4	-0.301	3.12E-13	IRAK3	-0.416	6.22E-09	SYNGR2	0.320	2.46E-07	LURAP1L	-0.388	2.29E-06	CYTH2	0.265	1.45E-05	CIB1	0.272	6.79E-05	BMP6	-0.302	3.70E-04	KDR	-0.402	3.25E-03
AFF1	-0.452	3.12E-13	SAA4	0.448	6.29E-09	MTHFD2L	-0.474	2.46E-07	TMEM205	0.329	2.29E-06	TIMD4	-0.778	1.45E-05	KDM8	0.392	6.81E-05	CAVIN2	-0.344	3.73E-04	DUSP8	0.274	3.29E-03
SLX4IP	-0.377	3.23E-13	TNKS2	-0.373	6.53E-09	WEE1	-0.324	2.61E-07	GREB1L	-0.321	2.33E-06	CTTNBP2	-0.304	1.46E-05	LRRC59	0.263	6.83E-05	SMTN	-0.298	3.74E-04	ARL4D	0.277	3.42E-03

ROCK2	-0.381	3.92E-13	MAP3K5	-0.359	6.56E-09	ASAP1	-0.300	2.61E-07	CXXC4	-0.372	2.34E-06	CNOT6L	-0.273	1.46E-05	C9	-0.404	6.83E-05	PKN2	-0.271	3.76E-04	AGR2	-0.325	3.52E-03
MYO16	-0.478	5.26E-13	MEA1	0.354	6.60E-09	FBXO46	-0.306	2.63E-07	LRRC8E	0.281	2.38E-06	CDC42EP1	0.407	1.46E-05	PNKP	0.279	6.83E-05	FBXW5	0.319	3.82E-04	LILRB1	-0.272	3.55E-03
SNX12	0.327	7.87E-13	USP6NL	-0.312	6.60E-09	AGTR1	-0.341	2.65E-07	SMARCA1	-0.281	2.41E-06	UBN2	-0.301	1.46E-05	GPAT3	-0.377	6.83E-05	CHRM3	-0.417	3.85E-04	PFN2	-0.283	3.57E-03
LASP1	0.348	8.41E-13	CTSD	0.538	6.67E-09	TUBG1	0.285	2.73E-07	TSSC4	0.421	2.51E-06	SNORC	0.449	1.47E-05	PRPF39	-0.280	6.87E-05	CDH19	-0.307	3.86E-04	CUX2	-0.492	3.59E-03
IPO13	0.331	1.01E-12	LY6E	0.571	6.78E-09	RPS6KB2	0.337	2.83E-07	RGS1	-0.561	2.54E-06	SERPINA4	0.288	1.47E-05	SPEN	-0.302	6.91E-05	TRPM7	-0.293	3.92E-04	NAPRT	0.301	3.59E-03
TNIK	-0.585	1.10E-12	ZFAND5	-0.532	7.11E-09	SIAH1	-0.335	2.83E-07	PGA3	-0.295	2.56E-06	FBXO28	-0.314	1.48E-05	ZFYVE16	-0.293	6.91E-05	TMC8	-0.286	4.02E-04	TMEM106B	-0.280	3.60E-03
RBMS2	-0.443	1.15E-12	RGS7	-0.549	7.31E-09	FBXO44	0.360	2.86E-07	HMGN2	-0.273	2.58E-06	CREBZF	-0.350	1.50E-05	SORD	0.315	6.94E-05	CDH5	-0.346	4.08E-04	OASL	0.297	3.67E-03
MAGI1	-0.344	1.26E-12	SLC25A32	-0.330	7.31E-09	AMOTL1	-0.370	2.91E-07	EPHA7	-0.316	2.62E-06	KLF2	-0.329	1.52E-05	HBB	0.884	7.05E-05	TRPM8	0.288	4.09E-04	HLA-F	0.295	3.79E-03
TMEM41B	-0.424	1.72E-12	CHMP1A	0.308	7.31E-09	BAK1	0.282	2.93E-07	GPAA1	0.347	2.63E-06	NELFB	0.270	1.54E-05	POR	0.293	7.12E-05	ZNF468	0.279	4.09E-04	LSAMP	-0.275	3.80E-03
GPD1	0.508	1.75E-12	MON2	-0.412	7.32E-09	ESRRA	0.340	2.97E-07	IGF1R	-0.316	2.66E-06	PPARA	-0.279	1.58E-05	RIMBP2	-0.468	7.12E-05	ETS2	-0.280	4.10E-04	TRIB3	0.269	3.80E-03
DIPK2A	-0.620	1.83E-12	PPP2R2A	-0.288	7.40E-09	BRIP1	-0.363	3.00E-07	RPS6KA6	-0.290	2.67E-06	HSBP1L1	-0.316	1.58E-05	RFX1	-0.308	7.16E-05	PDE2A	-0.299	4.18E-04	ANKRD36C	0.323	3.94E-03
C19orf25	0.495	1.95E-12	RDH12	-0.500	8.30E-09	PRKCSH	0.342	3.12E-07	RPS6KL1	0.328	2.67E-06	TMX3	-0.384	1.59E-05	TMEM200B	0.317	7.16E-05	LGALS4	0.669	4.18E-04	UGT1A6	0.387	3.96E-03
IVNS1ABP	-0.379	1.96E-12	FCAMR	0.724	8.41E-09	HOXA3	-0.286	3.22E-07	DAO	0.362	2.67E-06	FCRL3	-0.302	1.59E-05	ADCY10	-0.331	7.21E-05	LSR	0.287	4.23E-04	DBP	0.280	4.01E-03
PARD6B	-0.496	3.19E-12	TMEM9	0.304	8.56E-09	TSR3	0.382	3.22E-07	SLC25A39	0.291	2.70E-06	TOM1	0.272	1.61E-05	TK1	0.312	7.21E-05	PLEKHH3	0.323	4.24E-04	GLS2	-0.392	4.05E-03
SUMF2	0.355	3.19E-12	PRX	0.300	8.81E-09	IRF2BP2	-0.305	3.24E-07	C9orf72	-0.355	2.72E-06	ARHGAP28	-0.290	1.61E-05	BIRC6	-0.329	7.34E-05	SLC2A12	-0.387	4.38E-04	SCAMP1	-0.264	4.13E-03
PER2	-0.523	3.22E-12	RTP3	0.516	9.28E-09	DNAJB2	0.318	3.24E-07	SPATA6	-0.283	2.73E-06	SHROOM2	-0.264	1.61E-05	DYNLL1	0.280	7.43E-05	HIF3A	-0.306	4.55E-04	DUSP6	-0.292	4.15E-03
SPRYD3	0.388	3.39E-12	USP5	0.275	9.28E-09	KDM2B	-0.269	3.38E-07	PCDHB16	-0.270	2.77E-06	MXD4	0.323	1.65E-05	VAMP1	0.287	7.49E-05	WNT11	0.387	4.57E-04	LRRC31	0.292	4.18E-03
FRRS1	-0.421	3.75E-12	KIAA0930	0.305	9.34E-09	RADX	-0.290	3.39E-07	MPDU1	0.281	2.77E-06	PHLPP1	-0.285	1.66E-05	MMD	-0.274	7.60E-05	SLC38A2	-0.382	4.59E-04	SLC38A4	-0.342	4.21E-03
HS1BP3	0.361	3.89E-12	CEBPA	0.493	9.34E-09	CUL4B	-0.301	3.40E-07	TMED1	0.276	2.78E-06	FBXO27	0.283	1.67E-05	OGFOD2	0.286	7.78E-05	PRSS8	0.344	4.63E-04	SERF2	0.267	4.27E-03
CDK19	-0.332	6.04E-12	NOTUM	0.569	1.01E-08	TRMT10A	-0.267	3.45E-07	CYB561A3	0.270	2.78E-06	RRP7A	0.307	1.67E-05	THRSP	0.488	7.79E-05	GSTZ1	0.306	4.64E-04	SH3PXD2A	-0.338	4.39E-03
ZSWIM6	-0.391	6.58E-12	SORBS1	-0.338	1.03E-08	PTBP2	-0.381	3.49E-07	BLK	-0.287	2.79E-06	TRAPPC6A	0.336	1.69E-05	CD9	-0.305	8.02E-05	DNM1	-0.485	4.67E-04	OSBPL8	-0.285	4.42E-03
DDRGK1	0.438	6.64E-12	VPS13C	-0.514	1.10E-08	DMXL2	-0.391	3.73E-07	R3HDM4	0.271	2.82E-06	RALGPS1	0.322	1.71E-05	PKLR	0.384	8.22E-05	TEK	-0.335	4.78E-04	CD24	0.414	4.46E-03
AFF3	-0.546	7.45E-12	MXI1	-0.319	1.12E-08	UFL1	-0.373	3.73E-07	LHX2	-0.284	2.87E-06	SRRM1	-0.280	1.72E-05	DMXL1	-0.330	8.22E-05	GNB4	-0.319	4.82E-04	COL1A1	0.435	4.55E-03
TAGLN2	0.471	9.69E-12	KLHL11	-0.318	1.15E-08	ZBTB42	0.278	3.73E-07	GLTPD2	0.478	2.87E-06	MTURN	-0.302	1.72E-05	AASS	-0.419	8.24E-05	CHL1	-0.455	4.82E-04	RAB27B	-0.273	4.58E-03
TP53I3	0.690	1.01E-11	PPM1K	-0.489	1.17E-08	SKP2	-0.275	3.75E-07	FAM83G	0.454	2.89E-06	TENM3	-0.416	1.73E-05	RPL36	0.370	8.41E-05	LRP3	0.343	4.82E-04	IL17RB	-0.277	4.67E-03
XPNPEP3	-0.328	1.04E-11	SPRED1	-0.420	1.18E-08	COX6A1	0.404	3.75E-07	NCAPH2	0.280	3.01E-06	ADGRF5	-0.442	1.75E-05	RTN4RL1	0.281	8.44E-05	PRDX4	0.273	4.87E-04	RPS15	0.270	4.71E-03
PRKAA1	-0.426	1.04E-11	P4HB	0.338	1.21E-08	SLK	-0.299	3.77E-07	SPSB4	-0.326	3.01E-06	MBD3	0.458	1.78E-05	KPNA2	0.294	8.54E-05	FAM76B	-0.292	4.89E-04	NRCAM	-0.310	4.84E-03
C6orf47	0.300	1.04E-11	KRT18	0.484	1.22E-08	CHMP6	0.312	3.77E-07	MED18	0.329	3.03E-06	ZG16	0.391	1.79E-05	FOXN2	-0.321	8.66E-05	SEMA5A	-0.279	4.91E-04	SIGIRR	0.302	4.94E-03
PRDM10	-0.329	1.08E-11	TPP2	-0.316	1.29E-08	CHID1	0.345	3.80E-07	EP400	-0.325	3.05E-06	BSG	0.312	1.81E-05	PPP1R3C	0.535	8.70E-05	SLC16A12	-0.388	4.94E-04	IL10RA	-0.270	4.97E-03
MAF	-0.470	1.20E-11	KCNC4	-0.363	1.34E-08	BMPER	-0.408	3.80E-07	GALC	-0.292	3.10E-06	CENPX	0.358	1.81E-05	ISG15	0.504	8.73E-05	ACOT1	0.419	5.02E-04	HLA-G	-0.289	5.01E-03
SS18	-0.280	1.25E-11	SLITRK3	-0.789	1.34E-08	APRT	0.367	3.92E-07	AURKAIP1	0.447	3.10E-06	PFKL	0.349	1.84E-05	PSKH1	0.264	8.81E-05	CROCC2	0.301	5.03E-04	SCN7A	-0.364	5.06E-03
ZC3H4	-0.383	1.25E-11	SPAG9	-0.352	1.38E-08	DMTN	0.445	4.00E-07	TNKS1BP1	0.331	3.13E-06	GTF2IRD2	-0.267	1.88E-05	GRN	0.336	8.94E-05	MAN2A1	-0.315	5.07E-04	SLC7A2	-0.286	5.06E-03
PRKAG2	-0.374	1.25E-11	IFI27L1	0.343	1.51E-08	PIN1	0.330	4.09E-07	ZNF532	-0.312	3.13E-06	NACC1	0.272	1.89E-05	SMPDL3A	-0.296	8.95E-05	RPS27A	0.312	5.14E-04	DDIT4	0.361	5.06E-03
ANGEL2	-0.305	1.26E-11	TIMM17B	0.381	1.56E-08	SSH2	-0.275	4.26E-07	PSTPIP2	-0.290	3.17E-06	ITPR2	-0.322	1.90E-05	EEF1D	0.312	9.07E-05	LPCAT2	-0.271	5.17E-04	PEG10	0.614	5.06E-03
RREB1	-0.450	1.26E-11	DUSP19	-0.318	1.61E-08	HS6ST1	0.353	4.31E-07	VIL1	-0.900	3.19E-06	FHIP2A	-0.302	1.91E-05	LIFR	-0.413	9.09E-05	MARCKS	-0.367	5.20E-04	TMPRSS9	0.340	5.27E-03
PGAM5	0.376	1.31E-11	CHRAC1	-0.265	1.61E-08	DACH1	-0.323	4.31E-07	PCDHGB6	-0.319	3.22E-06	GUCY1A2	-0.388	1.94E-05	PRLR	-0.424	9.14E-05	HOOK1	-0.284	5.22E-04	GLIS2	0.264	5.65E-03
LIN7C	-0.493	1.55E-11	TMBIM1	0.331	1.62E-08	ETV7	0.351	4.32E-07	DNAJB1	0.265	3.28E-06	TLE3	-0.305	1.94E-05	TAT	-0.576	9.16E-05	KLF13	0.286	5.23E-04	MS4A6A	-0.300	5.72E-03
INHBE	0.833	1.69E-11	AKAP11	-0.447	1.64E-08	PSMB7	0.308	4.32E-07	SLC7A5	0.321	3.33E-06	SAC3D1	0.312	1.94E-05	NBEAL1	-0.325	9.18E-05	MCF2L	-0.284	5.25E-04	CD5L	-0.537	5.87E-03
PMVK	0.476	1.69E-11	CCDC107	0.353	1.68E-08	SFXN2	-0.283	4.34E-07	KLF16	0.330	3.35E-06	ATP7A	-0.264	1.95E-05	DENND4C	-0.330	9.28E-05	IQGAP1	-0.363	5.29E-04	A1BG	0.290	6.07E-03
ARSK	-0.406	1.78E-11	SLC15A2	-0.351	1.69E-08	DGAT2	0.519	4.40E-07	IFRD2	0.309	3.36E-06	USP34	-0.321	1.95E-05	NRP2	-0.397	9.38E-05	PDIA6	0.381	5.36E-04	CPT1A	-0.298	6.09E-03
ATP5F1E	0.441	1.81E-11	ADAM15	0.345	1.69E-08	CEBPB	-0.466	4.41E-07	STRN	-0.302	3.36E-06	FOXO3	-0.273	2.00E-05	TTLL7	-0.324	9.65E-05	ACSM1	-0.514	5.36E-04	AGPAT5	-0.274	6.30E-03
FBXO6	0.383	1.85E-11	STK16	0.277	1.70E-08	CDH17	-0.324	4.42E-07	ZGPAT	0.428	3.48E-06	INPP5D	-0.308	2.05E-05	DCUN1D4	-0.288	9.70E-05	LYNX1	0.331	5.39E-04	ELOVL2	0.314	6.33E-03
CFD	-0.784	1.85E-11	GPC1	0.459	1.74E-08	UGCG	-0.362	4.45E-07	TMEM131	-0.278	3.65E-06	GFRA2	-0.355	2.06E-05	TSPAN13	-0.567	9.74E-05	DPY19L4	-0.284	5.39E-04	DNMT3L	0.295	6.38E-03
TMEM140	0.367	1.90E-11	SLC39A5	0.427	2.00E-08	NOTCH2NL	-0.302	4.45E-07	RC3H1	-0.299	3.66E-06	CCDC88C	-0.352	2.07E-05	ABCB4	0.335	9.82E-05	PSMB10	0.279	5.42E-04	RRM2	0.269	6.57E-03
LIMCH1	-0.492	2.21E-11	TAF4	-0.272	2.01E-08	SYT7	0.581	4.47E-07	PGC	-0.386	3.69E-06	ARHGAP42	-0.274	2.10E-05	CTBS	-0.291	9.95E-05	SLC17A1	-0.295	5.44E-04	RELN	-0.343	6.62E-03
MAK16	-0.284	2.39E-11	LDLRAP1	0.273	2.15E-08	SCYL1	0.303	4.53E-07	PSMB6	0.279	3.79E-06	MCC	-0.312	2.11E-05	KCNJ10	-0.458	9.97E-05	CNTNAP4	0.286	5.46E-04	COL1A2	0.328	6.64E-03
PEX11G	0.373	2.57E-11	UBL7	0.270	2.18E-08	MTX3	-0.280	4.57E-07	SERINC2	0.385	3.82E-06	SHROOM4	-0.291	2.13E-05	NTN4	-0.338	9.98E-05	PLIN4	0.437	5.50E-04	LGALS3	0.267	6.68E-03
ABCA1	-0.658	2.58E-11	RIMOC1	-0.422	2.22E-08	UBR2	-0.278	4.65E-07	SGSM3	0.291	3.88E-06	CRAT	0.265	2.16E-05	CRYM	0.285	1.00E-04	RPS14	0.309	5.52E-04	DNAH11	-0.301	6.77E-03
CACFD1	0.480	2.86E-11	CEP120	-0.297	2.23E-08	KLHDC7A	0.407	4.66E-07	TGFBI	0.322	3.91E-06	ZNF280D	-0.264	2.16E-05	ADCYAP1R1	-0.471	1.01E-04	SALL4	-0.289	5.55E-04	PNISR	0.312	6.78E-03
ETV5	-0.499	2.90E-11	DCBLD2	-0.305	2.24E-08	ACVR1C	-0.391	4.70E-07	SMC5	-0.298	3.94E-06	CCDC167	0.345	2.22E-05	RAB17	0.280	1.02E-04	SRD5A2	0.371	5.71E-04	TOP2A	0.289	6.78E-03
POLE3	0.324	2.90E-11	RHBDD3	0.439	2.26E-08	UQCRFS1	0.328	4.72E-07	MRAS	0.308	3.97E-06	NOVA2	-0.270	2.22E-05	DPP7	0.352	1.02E-04	MIDN	-0.287	5.73E-04	RWDD3	-0.292	6.98E-03
EXOC1	-0.354	2.90E-11	RTCB	0.270	2.30E-08	MORC3	-0.334	4.74E-07	SEC14L4	0.528	4.04E-06	TMEM104	0.264	2.23E-05	UBE2D1	-0.300	1.03E-04	TREH	0.475	5.74E-04	SULT1E1	-0.312	7.36E-03
CBLB	-0.318	2.98E-11	R3HCC1	0.315	2.30E-08	CEP162	-0.275	4.74E-07	CSNK1G3	-0.272	4.07E-06	PTPRS	-0.537	2.23E-05	IGFBP1	-1.135	1.03E-04	FSTL3	0.326	5.80E-04	SC5D	-0.312	7.40E-03
ANXA6	0.529	3.01E-11	GIPC1	0.388	2.31E-08	DYNLT1	0.324	4.75E-07	PPP1R3G	-0.452	4.21E-06	NPR1	-0.342	2.29E-05	SAMD5	-0.374	1.03E-04	NAT8	0.363	5.88E-04	SULF1	-0.320	7.61E-03

L3MBTL3	-0.430	3.55E-11	NIBAN2	0.457	2.40E-08	PLCB1	-0.364	4.82E-07	TIMM17A	0.312	4.21E-06	PROX1	-0.356	2.33E-05	LYVE1	-0.669	1.04E-04	KBTBD11	-0.278	5.94E-04	ELOVL6	0.309	7.62E-03
BCL2L1	0.385	3.77E-11	KHK	0.376	2.44E-08	TYMP	0.499	4.88E-07	FADS2	0.851	4.26E-06	TMEM208	0.299	2.37E-05	UBE2L6	0.304	1.04E-04	SAMD9L	-0.321	5.95E-04	TCIM	-0.304	7.84E-03
PSEN2	0.285	4.00E-11	KAT6A	-0.337	2.44E-08	ARHGAP6	-0.266	4.96E-07	PATJ	-0.294	4.39E-06	PEG3	-0.307	2.38E-05	PGAP6	0.271	1.05E-04	CDH23	-0.340	6.03E-04	CCR1	-0.302	7.95E-03
PELI1	-0.385	4.00E-11	C2CD4B	-0.288	2.48E-08	PSMC1	0.352	5.03E-07	ITCH	-0.303	4.44E-06	PTGES2	0.268	2.38E-05	PHLDA1	-0.427	1.05E-04	IGFBP2	-0.769	6.06E-04	PDE4B	-0.280	8.32E-03
MED13L	-0.445	4.18E-11	HEY2	-0.272	2.48E-08	MFSD3	0.426	5.03E-07	SCN9A	-0.449	4.47E-06	ASL	0.326	2.38E-05	GPT2	-0.353	1.05E-04	CREBRF	-0.288	6.07E-04	CSF1R	-0.366	8.47E-03
MUL1	0.343	4.55E-11	P4HA2	0.323	2.57E-08	POMK	-0.372	5.17E-07	ZEB2	-0.393	4.51E-06	IGF1	-0.593	2.40E-05	SEC14L1	-0.304	1.08E-04	MAD1L1	0.278	6.16E-04	LYZ	0.360	8.73E-03
NOL9	-0.357	4.60E-11	KANSL1L	-0.300	2.61E-08	FOXP1	-0.293	5.28E-07	ADARB2	-0.314	4.55E-06	EGLN3	-0.295	2.40E-05	FGFRL1	-0.296	1.08E-04	KLF5	-0.293	6.16E-04	TFPI2	-0.327	8.76E-03
CREBBP	-0.372	4.60E-11	BANF1	0.371	2.65E-08	LPA	-0.688	5.38E-07	GABRG3	-0.278	4.56E-06	TCF7L1	-0.291	2.42E-05	TFPI	-0.285	1.08E-04	SYT12	0.331	6.19E-04	JCHAIN	-0.462	8.77E-03
SNX17	0.299	6.62E-11	TUBB2A	0.513	2.70E-08	WWC1	0.323	5.42E-07	C11orf96	-0.391	4.65E-06	DOP1A	-0.322	2.46E-05	TUBA1C	0.279	1.08E-04	MRGPRF	0.342	6.20E-04	CYP3A43	-0.379	8.98E-03
EIF3G	0.383	6.97E-11	CDKN1C	-0.371	2.70E-08	DSCAM	-0.317	5.45E-07	SLC19A2	-0.383	4.72E-06	CDC34	0.291	2.47E-05	AEN	0.284	1.10E-04	IFI30	0.305	6.28E-04	TDGF1	0.271	9.15E-03
MARF1	-0.277	8.20E-11	CCM2	0.345	2.70E-08	RBMS1	-0.364	5.51E-07	WDR36	-0.294	4.75E-06	VAMP8	0.305	2.48E-05	APOBR	0.263	1.11E-04	DCHS1	-0.340	6.29E-04	CYFIP2	-0.356	9.43E-03
CCDC6	-0.291	9.14E-11	FRMD4B	-0.321	2.72E-08	DNAH6	-0.492	5.61E-07	WDR17	-0.274	4.76E-06	ETFB	0.368	2.49E-05	SYNE1	-0.263	1.11E-04	MERTK	-0.298	6.39E-04	CLEC1B	-0.302	9.54E-03
YKT6	0.286	9.75E-11	HECW2	-0.474	2.73E-08	GATA3	-0.297	5.64E-07	TSNAX	-0.287	4.77E-06	GABPA	-0.345	2.49E-05	ABHD1	0.264	1.11E-04	ABHD17A	0.273	6.43E-04	CD44	-0.265	9.66E-03
MACROH2A	0.416	9.75E-11	PHC3	-0.398	2.82E-08	SPHK2	0.387	5.64E-07	CROT	-0.284	4.84E-06	PRKCE	0.614	2.50E-05	FZR1	0.278	1.12E-04	FASTK	0.270	6.43E-04	OAT	-0.365	9.74E-03
TMEM141	0.481	9.85E-11	GAS6	0.503	2.83E-08	TNRC6C	-0.336	5.64E-07	IMPA1	-0.376	4.90E-06	GFUS	0.272	2.56E-05	RAB26	0.354	1.13E-04	TFCP2L1	-0.334	6.54E-04	GLUL	0.267	9.79E-03
ZCCHC14	-0.328	1.10E-10	CTNNA3	-0.636	2.90E-08	GALNT14	-0.315	5.72E-07	SOX18	-0.358	4.97E-06	CYP2B6	-0.520	2.60E-05	COL5A3	0.656	1.14E-04	CYP3A4	-0.456	6.55E-04	MYH7	-0.288	9.92E-03
KIAA1217	-0.370	1.12E-10	KCNMB4	-0.326	3.03E-08	FERMT2	-0.308	5.77E-07	CDK17	-0.282	4.98E-06	ST14	0.381	2.61E-05	N4BP2	-0.312	1.15E-04	MANF	0.410	6.73E-04	AKR1C8	0.333	1.01E-02
RPL17	0.335	1.12E-10	TREM2	0.557	3.14E-08	ZNF563	0.293	5.98E-07	EPB41L3	-0.446	4.98E-06	TMPRSS6	0.315	2.63E-05	LRRC8C	-0.295	1.16E-04	P2RY13	-0.377	6.75E-04	LIME1	0.345	1.01E-02
ERP29	0.420	1.14E-10	VCPIP1	-0.340	3.22E-08	SLC25A10	0.410	6.01E-07	SNX18	-0.271	5.02E-06	AP1AR	-0.280	2.64E-05	SF3B5	0.271	1.19E-04	SLC28A1	0.363	6.78E-04	MT1E	-0.378	1.02E-02
PPFIA3	0.407	1.22E-10	NICN1	0.302	3.30E-08	TRAF7	0.325	6.07E-07	EPB41L4A	-0.344	5.04E-06	MMRN1	-0.571	2.64E-05	TTC9	0.398	1.28E-04	PTPRB	-0.415	6.85E-04	CP	-0.318	1.03E-02
MRPS28	0.376	1.34E-10	PLSCR4	-0.478	3.33E-08	RALGAPA1	-0.334	6.07E-07	ISM1	0.466	5.09E-06	BBLN	0.413	2.65E-05	MAST1	-0.270	1.29E-04	FRK	-0.278	6.89E-04	ITGAD	-0.366	1.05E-02
BASP1	-0.550	1.46E-10	TST	0.416	3.35E-08	SNTB1	-0.309	6.10E-07	GPD2	-0.271	5.20E-06	ZBTB7B	0.291	2.69E-05	LAMA3	0.378	1.30E-04	GCK	0.632	7.06E-04	SLC38A1	-0.270	1.06E-02
TRIT1	-0.293	1.49E-10	FNIP1	-0.420	3.44E-08	DIO1	0.376	6.10E-07	NUDT16L1	0.271	5.21E-06	CKS2	0.299	2.70E-05	HLA-C	0.330	1.31E-04	JUNB	-0.402	7.08E-04	ASS1	-0.271	1.08E-02
TULP4	-0.312	1.51E-10	ZDHHC17	-0.370	3.46E-08	PRXL2A	0.329	6.11E-07	TSPYL5	-0.332	5.23E-06	UBD	0.354	2.75E-05	FABP4	0.619	1.31E-04	NPW	-0.467	7.14E-04	SDF2L1	0.279	1.13E-02
MEF2A	-0.343	1.86E-10	STYX	-0.446	3.52E-08	VAT1L	-0.328	6.13E-07	CFAP69	-0.333	5.25E-06	PRDM1	-0.291	2.75E-05	ADGRL4	-0.401	1.31E-04	TRDN	-0.287	7.17E-04	NR0B2	-0.371	1.14E-02
ENPP1	-0.368	2.06E-10	ADGRA3	-0.412	3.69E-08	ADHFE1	-0.340	6.13E-07	ZSCAN23	-0.398	5.26E-06	AKAP12	-0.488	2.77E-05	ABCC1	-0.267	1.33E-04	PHPT1	0.286	7.21E-04	KCTD12	-0.269	1.15E-02
FBXO11	-0.364	2.13E-10	DHRS7	0.498	3.92E-08	CHMP4A	0.294	6.22E-07	YTHDC2	-0.264	5.30E-06	LGALS1	0.427	2.86E-05	PPIB	0.265	1.33E-04	MVK	0.336	7.24E-04	CACNA1H	0.376	1.15E-02
THRAP3	-0.306	2.22E-10	PTPRT	-0.444	3.93E-08	DBNDD1	0.402	6.22E-07	USP9X	-0.264	5.34E-06	STRN3	-0.269	2.87E-05	IRAG2	-0.286	1.35E-04	SLA	-0.284	7.26E-04	DNAH5	-0.269	1.20E-02
ATP8A1	-0.474	2.66E-10	UNC119	0.374	4.05E-08	PYGB	0.290	6.22E-07	SLC27A4	0.290	5.45E-06	GMPPA	0.263	2.88E-05	FUOM	0.344	1.35E-04	GINS2	0.284	7.33E-04	ANTXR1	-0.265	1.23E-02
ZBTB10	-0.472	2.66E-10	CD2BP2	0.276	4.12E-08	BTBD8	-0.308	6.25E-07	FLT1	-0.445	5.45E-06	SERTAD1	0.284	2.89E-05	NTAN1	0.278	1.36E-04	GPAM	0.339	7.59E-04	MAPK4	-0.284	1.27E-02
HDGF	0.352	2.66E-10	CCDC85C	0.346	4.13E-08	NUCB1	0.349	6.27E-07	SP4	-0.329	5.47E-06	CNKSR2	-0.335	2.95E-05	CSTA	0.463	1.37E-04	TLR2	-0.275	7.63E-04	KLF6	-0.276	1.32E-02
ING1	-0.295	2.76E-10	PHF8	-0.406	4.15E-08	CHIC1	-0.457	6.28E-07	NEURL1	-0.304	5.49E-06	DENND3	-0.282	2.96E-05	ADAM23	-0.270	1.39E-04	DIRAS3	-0.283	7.72E-04	SPTBN5	0.319	1.35E-02
CALM1	-0.284	2.79E-10	CBFB	-0.357	4.15E-08	PTPRU	0.410	6.35E-07	ZNF521	-0.318	5.50E-06	TDRP	-0.280	3.03E-05	PKD1L1	-0.289	1.42E-04	RAD54L2	-0.270	8.05E-04	SLC35C1	0.264	1.38E-02
HEATR5B	-0.424	3.01E-10	C11orf68	0.310	4.19E-08	FCHO2	-0.365	6.35E-07	ARF3	0.265	5.52E-06	NPY1R	-0.390	3.03E-05	KMT2C	-0.290	1.42E-04	ZWINT	0.270	8.10E-04	LPL	0.324	1.38E-02
CD276	0.407	3.32E-10	MCCC2	-0.282	4.20E-08	KALRN	-0.269	6.48E-07	TUBB4B	0.353	5.52E-06	SIK2	-0.282	3.03E-05	LAD1	0.353	1.44E-04	VCAM1	-0.552	8.18E-04	SLC16A11	0.286	1.39E-02
PCDH20	-0.641	3.43E-10	APOL3	0.599	4.26E-08	SIM1	-0.503	6.48E-07	CAPG	0.423	5.58E-06	OGT	-0.318	3.03E-05	CXADR	-0.339	1.45E-04	STARD10	0.263	8.23E-04	SLC3A1	-0.492	1.42E-02
LDB2	-0.518	3.43E-10	RELL1	-0.311	4.26E-08	RASGEF1B	-0.352	6.56E-07	NOP10	0.299	5.60E-06	COL28A1	-0.533	3.03E-05	DRAP1	0.297	1.47E-04	RPS18	0.272	8.44E-04	NDUFB7	0.268	1.49E-02
ATF7IP	-0.375	3.63E-10	BST2	0.605	4.28E-08	AMDHD1	-0.502	6.74E-07	BMPR2	-0.339	5.62E-06	C1orf162	-0.299	3.09E-05	SEC11C	0.300	1.50E-04	TMIGD3	-0.331	8.51E-04	DCDC1	-0.263	1.51E-02
ZNF507	-0.319	3.64E-10	MYO9A	-0.359	4.41E-08	SNRPB	0.278	6.84E-07	H2AJ	0.397	5.63E-06	FAM241A	-0.302	3.16E-05	HS3ST3A1	-0.279	1.50E-04	SLC45A3	0.282	8.52E-04	ASB9	-0.267	1.52E-02
FBN2	-0.495	3.75E-10	EDA	-0.375	4.61E-08	MYDGF	0.361	6.86E-07	CITED2	-0.319	5.67E-06	HIPK1	-0.270	3.20E-05	MRPL51	0.268	1.54E-04	FAM151A	0.622	8.52E-04	GALK1	0.290	1.52E-02
PAQR7	0.351	3.83E-10	ZNF48	0.272	4.64E-08	CAPN1	0.303	7.11E-07	PLGLB2	-0.321	5.89E-06	SSNA1	0.287	3.27E-05	EPHA1	0.267	1.56E-04	CR1	-0.456	8.79E-04	NTHL1	0.267	1.55E-02
COQ8B	0.351	4.09E-10	ALDH6A1	-0.518	4.76E-08	ZNF638	-0.287	7.20E-07	SIRT1	-0.299	5.92E-06	TUBA1B	0.292	3.35E-05	SCIMP	-0.314	1.58E-04	RETREG1	-0.366	8.93E-04	THBS1	-0.347	1.55E-02
OAZ2	0.291	4.21E-10	IFI35	0.362	4.85E-08	AGPAT2	0.520	7.39E-07	YIF1B	0.299	6.04E-06	TINAGL1	-0.375	3.40E-05	DDX60L	-0.339	1.59E-04	CNTN3	-0.285	8.95E-04	IFI6	0.415	1.61E-02
GABPB1	-0.288	4.31E-10	BECN1	0.344	4.95E-08	MMP15	0.379	7.43E-07	SYNE4	0.347	6.18E-06	RNF19A	-0.272	3.40E-05	TTC6	-0.309	1.60E-04	FAM111B	0.369	8.97E-04	SAA1	0.590	1.62E-02
FBXO2	0.632	4.42E-10	RNF167	0.272	5.10E-08	RANGAP1	0.334	7.63E-07	TJP1	-0.264	6.26E-06	UBASH3B	-0.273	3.41E-05	FGD4	-0.322	1.62E-04	ATAD3A	0.267	9.12E-04	CA12	0.367	1.65E-02
FMR1	-0.439	4.74E-10	SHISA5	0.353	5.15E-08	TMEM19	0.373	7.82E-07	KLHDC1	-0.314	6.26E-06	PRKAR2B	-0.300	3.41E-05	GCH1	-0.287	1.62E-04	MYCT1	-0.266	9.18E-04	PPDPF	0.279	1.65E-02
UBR3	-0.439	4.77E-10	LAMTOR2	0.373	5.28E-08	CHD1	-0.308	7.89E-07	TARBP2	0.281	6.49E-06	FAM135A	-0.278	3.41E-05	ADTRP	-0.281	1.66E-04	NRXN3	-0.280	9.20E-04	SYBU	-0.272	1.66E-02
CYB5R1	0.267	4.81E-10	PSMC3	0.281	5.45E-08	CYP4F22	0.490	7.93E-07	PGA5	-0.308	6.61E-06	LTN1	-0.360	3.44E-05	ADGRE1	-0.417	1.66E-04	DDIT3	0.268	9.29E-04	NDST3	-0.276	1.68E-02
PDAP1	0.305	4.82E-10	POGZ	-0.372	5.56E-08	APBB2	-0.280	7.98E-07	DOCK4	-0.322	6.68E-06	LRP6	-0.328	3.48E-05	SOGA1	-0.301	1.70E-04	S100A4	0.292	9.33E-04	NEK10	-0.426	1.69E-02
CDK4	0.342	5.29E-10	SERPINA5	0.464	5.58E-08	SNRK	-0.350	7.99E-07	NHP2	0.274	6.78E-06	GK	-0.380	3.50E-05	GDF2	0.300	1.70E-04	PRODH2	0.272	9.69E-04	USH2A	-0.301	1.71E-02
TSC22D1	0.440	5.30E-10	CHPF2	0.276	5.63E-08	RP1	-0.352	8.04E-07	CD81	0.300	6.79E-06	TEDC1	0.485	3.51E-05	PDE3B	-0.299	1.70E-04	SUSD4	0.443	9.84E-04	C4A	0.315	1.71E-02
TGFBR3	-0.465	6.06E-10	FIBP	0.299	5.63E-08	HMGA1	0.334	8.09E-07	TRAF4	0.276	6.79E-06	LRRC45	0.360	3.51E-05	MROH2B	-0.302	1.70E-04	DOP1B	-0.505	1.00E-03	HMOX1	-0.263	1.83E-02
GAB1	-0.353	6.71E-10	CAPNS1	0.304	6.01E-08	PNRC1	-0.336	8.40E-07	PABPC4L	-0.265	6.84E-06	ASAP3	0.281	3.53E-05	SCD	0.647	1.71E-04	PDCD1LG2	-0.316	1.04E-03	MLIP	0.484	1.87E-02
LATS1	-0.356	6.88E-10	SEMA6A	-0.400	6.05E-08	HSPA8	0.435	8.40E-07	DDX3X	-0.375	6.89E-06	RIF1	-0.269	3.55E-05	ATF5	0.379	1.74E-04	ABCG2	-0.287	1.05E-03	OR2I1P	0.394	1.91E-02

SOCS2	-0.703	6.93E-10	OGA	-0.277	6.15E-08	S100A16	0.277	8.43E-07	REPS1	-0.287	7.18E-06	PHACTR2	-0.305	3.56E-05	PPIF	0.387	1.76E-04	P2RY12	-0.300	1.07E-03	EIF1AY	0.818	1.94E-02
VTN	0.405	7.03E-10	SCN11A	-0.317	6.15E-08	PALLD	-0.467	8.43E-07	SLC8A1	-0.394	7.20E-06	TMTC1	-0.473	3.57E-05	XKR4	-0.302	1.77E-04	ABCA5	-0.286	1.07E-03	VSIG4	-0.355	1.97E-02
FAM227B	-0.346	7.09E-10	ATG101	0.340	6.49E-08	PRXL2B	0.279	8.46E-07	RNASET2	-0.272	7.20E-06	EEA1	-0.401	3.57E-05	RPS3	0.284	1.78E-04	IL6ST	-0.348	1.09E-03	MTUS2	-0.275	1.97E-02
LMTK2	-0.307	7.42E-10	OSBPL6	-0.445	6.56E-08	UBE4A	-0.264	8.52E-07	BAD	0.363	7.22E-06	LITAF	0.290	3.57E-05	CD2AP	-0.306	1.81E-04	ZBED6	-0.479	1.09E-03	FOLR2	-0.315	2.01E-02
RERE	-0.404	7.43E-10	AGL	-0.496	6.63E-08	ZFHX4	-0.360	8.56E-07	FDX2	0.300	7.31E-06	BCL7C	0.281	3.59E-05	LGSN	-0.329	1.83E-04	BTNL9	-0.303	1.09E-03	MPEG1	-0.320	2.01E-02
NATD1	0.304	7.57E-10	RFX3	-0.324	6.83E-08	ARMC6	0.399	8.56E-07	GCNT1	-0.339	7.36E-06	PER3	-0.450	3.60E-05	RHOB	-0.417	1.87E-04	C5AR2	-0.279	1.10E-03	ARL17B	-0.275	2.05E-02
SURF4	0.278	8.45E-10	ATG2B	-0.344	6.91E-08	LARP4	-0.348	8.61E-07	HYAL3	0.322	7.40E-06	IKBKG	0.275	3.62E-05	CIT	-0.283	1.87E-04	ADAMTS1	-0.336	1.13E-03	MAB21L4	0.495	2.05E-02
PES1	0.295	8.45E-10	TRAPPC2	-0.284	6.93E-08	FEN1	0.293	8.68E-07	DNAJC12	-0.354	7.40E-06	C17orf67	-0.311	3.64E-05	COMT	0.324	1.92E-04	FGF14	-0.436	1.15E-03	FDPS	0.352	2.08E-02
LBR	-0.430	8.45E-10	NHSL2	-0.341	7.35E-08	PTPRD	-0.406	8.69E-07	ZNF160	-0.305	7.42E-06	RPL10	0.294	3.65E-05	NEDD9	-0.323	1.96E-04	CDHR5	0.323	1.20E-03	GASK1B	-0.273	2.10E-02
STK4	-0.328	8.45E-10	FOXP2	-0.379	7.35E-08	RDH5	0.372	8.75E-07	LILRB5	-0.503	7.43E-06	AKT3	-0.289	3.67E-05	LSM7	0.294	1.97E-04	TDRD10	-0.298	1.20E-03	ICA1	-0.313	2.35E-02
SLC16A9	-0.558	8.67E-10	ZMYM6	-0.283	7.42E-08	MPND	0.346	8.80E-07	SLC16A13	0.436	7.43E-06	LAGE3	0.290	3.68E-05	SPIC	-0.288	1.98E-04	RPS26	0.349	1.28E-03	CFP	-0.280	2.39E-02
EPS15	-0.329	8.82E-10	TMEM63A	0.381	7.42E-08	C15orf62	0.265	8.88E-07	MTMR4	-0.283	7.51E-06	CIAO3	0.268	3.68E-05	CIDEC	0.307	1.98E-04	PCK1	-0.595	1.28E-03	NEU4	0.295	2.44E-02
AAMP	0.283	8.82E-10	ZFP1	-0.409	7.53E-08	CENPC	-0.285	8.92E-07	MAGI2	-0.301	7.52E-06	EXPH5	-0.361	3.70E-05	MYC	-0.326	2.02E-04	TCEA2	0.325	1.30E-03	INHBB	-0.265	2.60E-02
LRRC42	0.285	8.94E-10	WDFY3	-0.379	7.53E-08	PRIMA1	-0.327	8.93E-07	HIVEP1	-0.356	7.68E-06	MVP	0.362	3.71E-05	CSRP2	0.303	2.09E-04	SLC25A22	0.268	1.32E-03	CRHBP	-0.310	2.68E-02
NHSL1	-0.381	9.39E-10	P3H2	-0.309	7.54E-08	HPR	-0.476	9.33E-07	DDAH2	0.293	7.78E-06	DHCR24	0.288	3.72E-05	SLC7A8	-0.377	2.11E-04	NR4A2	-0.277	1.33E-03	EFHD1	-0.432	2.74E-02
MYL6	0.427	9.47E-10	STOML1	0.294	7.68E-08	RICTOR	-0.421	9.39E-07	ABCC9	-0.414	7.79E-06	HBA2	0.692	3.73E-05	ESPN	0.344	2.12E-04	DNAJB14	-0.264	1.33E-03	EPB41L1	-0.385	2.76E-02
PPFIBP1	-0.394	1.03E-09	UBR5	-0.297	7.68E-08	MET	-0.366	9.40E-07	TBXA2R	-0.293	7.79E-06	PEAR1	-0.283	3.74E-05	VWA1	0.359	2.13E-04	FASN	0.555	1.34E-03	UBXN10	0.273	2.87E-02
EGR1	-1.431	1.03E-09	BLNK	-0.343	7.73E-08	CTSF	0.271	9.54E-07	BRWD3	-0.328	7.86E-06	ALDH4A1	0.264	3.76E-05	CPD	-0.296	2.19E-04	GGT1	0.301	1.36E-03	FAT1	0.266	2.91E-02
TNFAIP8L1	0.360	1.03E-09	RNF152	-0.639	7.74E-08	GPR137B	-0.359	9.66E-07	CLXN	-0.281	7.86E-06	CCDC196	-0.321	3.83E-05	PLAG1	-0.267	2.21E-04	LEPR	-0.460	1.36E-03	ADAMTSL2	0.268	2.99E-02
HIPK3	-0.578	1.07E-09	HELZ	-0.348	7.86E-08	CCDC68	-0.356	9.67E-07	HECA	-0.283	8.00E-06	FAR1	-0.310	3.85E-05	GALNT18	0.276	2.21E-04	HS3ST2	0.272	1.37E-03	TMEM45B	0.328	3.03E-02
TCHH	-0.358	1.07E-09	TXNDC16	-0.351	7.89E-08	F10	0.300	9.85E-07	ELL2	-0.317	8.06E-06	ARSA	0.326	3.86E-05	MCRIP2	0.399	2.21E-04	EPOR	-0.279	1.41E-03	PRAMEF10	0.269	3.04E-02
FADD	0.279	1.09E-09	FMNL2	-0.422	7.89E-08	ACADL	-0.271	1.00E-06	EXOSC5	0.264	8.07E-06	SLC4A7	-0.360	3.92E-05	B3GAT3	0.270	2.21E-04	GADD45B	-0.410	1.42E-03	KNDC1	-0.268	3.06E-02
FCHSD2	-0.275	1.09E-09	RPUSD3	0.268	8.13E-08	CFL1	0.299	1.01E-06	CPAMD8	-0.363	8.10E-06	NF1	-0.274	3.92E-05	HLA-A	0.347	2.22E-04	TMEM200C	-0.268	1.45E-03	ARSL	0.287	3.16E-02
GNA12	0.297	1.09E-09	ERGIC3	0.280	8.73E-08	PHLDA3	0.472	1.12E-06	COPE	0.351	8.24E-06	RPL21	0.350	3.96E-05	CYP2C19	-0.843	2.23E-04	CD163L1	-0.309	1.46E-03	TMEM154	0.349	3.18E-02
TOR3A	0.474	1.12E-09	OPTN	0.273	9.10E-08	TM4SF4	0.310	1.14E-06	PDK4	-0.495	8.28E-06	ALDH18A1	0.281	3.98E-05	SSR4	0.291	2.25E-04	FCER1G	-0.392	1.48E-03	IGSF9	0.270	3.36E-02
GPR137	0.375	1.18E-09	IRS2	-0.596	9.67E-08	PSME2	0.319	1.16E-06	MPV17L2	0.337	8.35E-06	SLCO4A1	-0.264	4.01E-05	TRIM2	-0.339	2.29E-04	GPM6A	-0.314	1.49E-03	DEPP1	-0.265	3.38E-02
ILRUN	0.300	1.18E-09	CLEC1A	-0.316	9.70E-08	PDLIM5	-0.291	1.17E-06	NRXN1	-0.401	8.35E-06	PFKFB3	-0.400	4.09E-05	MPG	0.268	2.40E-04	TNFRSF12A	0.368	1.50E-03	SREBF1	0.281	3.39E-02
LYPLA2	0.365	1.20E-09	MON1A	0.266	9.88E-08	IRAK1	0.289	1.18E-06	FBXL4	-0.285	8.40E-06	COL3A1	0.415	4.09E-05	PIK3C2A	-0.311	2.41E-04	RPS19	0.284	1.52E-03	CES1	0.276	3.54E-02
ZNF385B	-0.408	1.20E-09	SLC39A3	0.328	9.94E-08	ARFRP1	0.346	1.18E-06	CXCL9	0.681	8.59E-06	GTF2A1	-0.295	4.12E-05	RAMP1	0.438	2.43E-04	ANGPTL8	0.457	1.53E-03	NEB	-0.267	3.61E-02
MRPS18C	0.392	1.20E-09	KCNAB1	-0.375	9.94E-08	RAB32	0.319	1.19E-06	NUDT22	0.376	8.62E-06	RGL1	-0.321	4.14E-05	DPY19L2	-0.273	2.46E-04	ADGRG6	-0.278	1.55E-03	HEPACAM	-0.341	3.65E-02
USP31	-0.346	1.20E-09	ARL8A	0.273	9.98E-08	NHLRC2	-0.335	1.19E-06	SPNS2	0.316	8.65E-06	SMG1	-0.421	4.16E-05	COMMD4	0.264	2.47E-04	SLC6A16	-0.292	1.56E-03	TMED7	-0.284	3.76E-02
LATS2	-0.282	1.20E-09	COBLL1	-0.374	1.02E-07	ICOSLG	0.334	1.21E-06	AOX1	-0.384	8.75E-06	SLC25A37	-0.305	4.18E-05	RAPGEF5	-0.291	2.47E-04	RASSF7	0.320	1.57E-03	LRP1B	-0.333	3.92E-02
KCNT2	-0.478	1.20E-09	SEZ6L	-0.304	1.02E-07	POLR2E	0.351	1.22E-06	TRPM6	-0.264	8.88E-06	NINJ1	0.312	4.19E-05	SLCO4C1	-0.452	2.54E-04	PINK1	0.268	1.58E-03	NUDT8	0.265	3.96E-02
HPRT1	-0.500	1.20E-09	RNF187	0.333	1.04E-07	ZNF518B	-0.339	1.23E-06	ATP1B2	-0.302	9.01E-06	NECTIN1	0.280	4.21E-05	ST8SIA4	-0.321	2.54E-04	PDGFRB	0.269	1.58E-03	IP6K3	0.345	4.00E-02
RNF26	0.310	1.24E-09	P2RY2	0.428	1.06E-07	ZNF770	-0.329	1.25E-06	MTMR7	-0.265	9.02E-06	DNA2	-0.306	4.29E-05	FSTL5	-0.276	2.58E-04	RASD1	-0.385	1.64E-03	DHCR7	0.267	4.01E-02
URM1	0.291	1.24E-09	MGLL	0.357	1.08E-07	TMEM63C	-0.287	1.26E-06	SLC22A10	-0.896	9.07E-06	GIMAP8	-0.340	4.35E-05	RPL7	0.279	2.60E-04	TRABD2B	-0.347	1.64E-03	NR1D2	-0.265	4.02E-02
FBXO21	-0.368	1.30E-09	CDNF	-0.290	1.09E-07	HDHD3	0.342	1.27E-06	OSBPL1A	-0.279	9.12E-06	IGSF3	0.324	4.35E-05	CLEC4M	-0.485	2.61E-04	ZBTB41	-0.296	1.64E-03	CFHR4	-0.296	4.32E-02
P4HA1	-0.856	1.30E-09	LCP2	-0.423	1.12E-07	LRRTM2	-0.303	1.27E-06	LAMB2	0.330	9.12E-06	CPEB4	-0.396	4.35E-05	TMUB1	0.330	2.66E-04	GLS	-0.268	1.67E-03	FBLN1	-0.295	4.52E-02
MIDEAS	-0.365	1.33E-09	PDK3	-0.356	1.14E-07	CYP7B1	-0.405	1.31E-06	CENPB	0.274	9.34E-06	LPAR6	-0.381	4.39E-05	POU6F2	-0.333	2.66E-04	MUC5B	-0.403	1.67E-03	PZP	-0.647	4.59E-02
SNX27	-0.267	1.35E-09	CARNMT1	-0.360	1.15E-07	NUDT18	0.277	1.33E-06	POLR2L	0.435	9.38E-06	GSDMD	0.355	4.49E-05	APOF	-0.318	2.66E-04	KCNC2	-0.272	1.69E-03	CFAP74	-0.280	4.71E-02
HPS6	0.289	1.35E-09	HDAC4	-0.279	1.19E-07	ZDBF2	-0.347	1.33E-06	MRC1	-0.628	9.39E-06	ACADS	0.366	4.51E-05	ELOVL5	0.303	2.67E-04	TNFRSF14	0.289	1.70E-03	FKBP5	-0.340	4.72E-02
CELF1	-0.285	1.37E-09	LNPEP	-0.345	1.21E-07	ARID2	-0.292	1.34E-06	IPCEF1	-0.278	9.39E-06	TOMM40L	0.313	4.54E-05	MRPL54	0.298	2.68E-04	SLC9A9	-0.302	1.74E-03	CHST9	0.268	4.74E-02
MRPS7	0.320	1.37E-09	TLNRD1	-0.286	1.23E-07	CD52	0.462	1.35E-06	TSPAN5	-0.311	9.44E-06	HDGFL2	0.288	4.57E-05	FDXR	0.285	2.76E-04	ABCA8	-0.294	1.79E-03	MYH2	-0.272	4.76E-02
GPATCH4	0.336	1.42E-09	ACOT7	0.306	1.25E-07	EIF3K	0.309	1.35E-06	EFNB2	-0.360	9.53E-06	EBI3	-0.313	4.57E-05	EPPK1	0.319	2.76E-04	CLEC4G	-0.340	1.79E-03	PGGHG	0.386	4.77E-02
SQSTM1	0.352	1.42E-09	MAP1LC3A	0.469	1.26E-07	PLEKHG1	-0.271	1.40E-06	SLC19A1	0.416	9.58E-06	SLC6A12	0.313	4.57E-05	NCOA2	-0.314	2.76E-04	SLC17A3	-0.282	1.82E-03	SLC5A12	-0.408	4.97E-02
SASH1	-0.366	1.44E-09	DPP3	0.286	1.26E-07	DTYMK	0.292	1.41E-06	EEF1G	0.272	9.66E-06	EGFLAM	-0.339	4.64E-05	SLC7A1	-0.267	2.78E-04	CXCR4	-0.328	1.85E-03			

Supplementary table 3: Differentially expressed genes of Borderline vs Control

Name	logFC	FDR	Name	logFC	FDR	Name	logFC	FDR	Name	logFC	FDR	Name	logFC	FDR	Name	logFC	FDR	Name	logFC	FDR
TOR1AIP2	-0.595	6.20E-27	NSUN6	-0.430	6.10E-10	GDF2	0.428	7.19E-08	YWHAH	0.267	1.90E-06	HS3ST3A1	-0.316	2.00E-05	MBNL3	-0.354	1.41E-04	JUNB	-0.383	1.40E-03
SLC12A4	0.577	9.57E-24	AMDHD1	-0.627	6.11E-10	KALRN	-0.292	7.25E-08	TMEM141	0.356	1.90E-06	IGFBP1	-1.254	2.01E-05	ETV1	-0.331	1.43E-04	TMIGD3	-0.320	1.42E-03
CPEB3	-0.845	7.86E-23	TMEM19	0.469	6.15E-10	TMED9	0.304	7.27E-08	ZFP36L1	-0.280	1.98E-06	COL5A3	0.729	2.02E-05	PITPNM1	0.300	1.43E-04	TRDN	-0.274	1.46E-03
AJUBA	0.815	6.31E-22	TRIB1	-0.481	6.46E-10	YIPF3	0.267	7.27E-08	WDR59	-0.274	1.99E-06	PTPRT	-0.348	2.04E-05	MYDGF	0.280	1.44E-04	LCP1	0.315	1.46E-03
CEBPD	-1.104	9.56E-22	PTBP2	-0.463	6.66E-10	SPRED2	-0.341	7.29E-08	IL1RAP	-0.538	2.02E-06	CERS4	0.295	2.04E-05	HLA-B	0.346	1.46E-04	FASN	0.555	1.47E-03
PTPA	0.552	1.01E-21	RPS6KA1	0.458	6.76E-10	NUP62CL	-0.293	7.29E-08	VAMP4	-0.293	2.02E-06	PLAG1	-0.308	2.05E-05	PANX2	-0.400	1.46E-04	BCHE	-0.332	1.47E-03
TIAL1	-0.342	5.08E-21	PRH1	-0.338	7.08E-10	MXD4	0.404	7.50E-08	DBNDD1	0.386	2.06E-06	NUCB1	0.302	2.05E-05	AQP4	0.357	1.46E-04	INHBB	-0.374	1.48E-03
TSPAN17	0.461	7.83E-21	SPNS2	0.436	8.04E-10	CREBZF	-0.434	7.65E-08	GPT2	-0.433	2.08E-06	NCF2	0.391	2.06E-05	PLEKHO2	0.299	1.49E-04	ABCA2	0.298	1.50E-03
IL32	1.426	4.83E-20	FBXO2	0.623	8.07E-10	LYZ	0.721	7.74E-08	CYTH2	0.291	2.09E-06	TNKS2	-0.276	2.06E-05	SUSD2	0.315	1.51E-04	CYP4X1	-0.280	1.51E-03
MACROH2A	0.593	7.37E-20	TMEM109	0.305	8.07E-10	RAC3	0.420	7.78E-08	MYO9A	-0.314	2.09E-06	PFKFB3	-0.418	2.09E-05	MFSD3	0.326	1.51E-04	LPAR6	-0.301	1.52E-03
TP53I3	0.927	1.48E-19	CDKN1A	0.763	8.07E-10	COQ8B	0.302	7.78E-08	NR0B2	-0.681	2.09E-06	ILK	0.287	2.10E-05	MLIP	0.767	1.51E-04	PAQR5	0.263	1.54E-03
TREM2	0.917	2.09E-19	TCF7L1	-0.422	8.07E-10	EMC4	0.318	7.89E-08	CCT6B	-0.286	2.09E-06	FMOD	0.360	2.11E-05	CNPY3	-0.282	1.52E-04	PER1	-0.383	1.56E-03
FRRS1	-0.548	2.81E-19	RPN2	0.305	8.26E-10	LPA	-0.739	8.16E-08	STRN3	-0.306	2.09E-06	SLAMF8	0.327	2.15E-05	NOTUM	0.382	1.54E-04	E2F1	0.275	1.58E-03
ST3GAL6	-0.681	2.81E-19	P2RY2	0.495	8.63E-10	EGR1	-1.259	8.25E-08	GPR158	-0.307	2.10E-06	SGSM3	0.270	2.21E-05	PRLR	-0.414	1.55E-04	ABCA13	-0.306	1.58E-03
SAE1	0.388	3.69E-19	S100A14	0.530	8.63E-10	SNTB1	-0.333	8.25E-08	KLF9	-0.433	2.17E-06	ACTN4	0.312	2.28E-05	B3GALT1	-0.271	1.56E-04	FAP	0.290	1.60E-03
SLC39A7	0.480	5.59E-19	MKX	-0.381	9.34E-10	CCDC9B	0.444	8.31E-08	GCH1	-0.361	2.17E-06	LIG4	-0.338	2.30E-05	UFL1	-0.281	1.58E-04	TRPM7	-0.264	1.63E-03
GNA12	0.430	2.39E-18	DIPK2A	-0.538	9.56E-10	AASS	-0.570	8.32E-08	NACC1	0.303	2.19E-06	ASB9	-0.457	2.30E-05	SENP7	-0.289	1.60E-04	PADI1	0.574	1.65E-03
DENND1C	0.417	2.49E-18	FAM227B	-0.343	9.72E-10	SS18L1	-0.311	8.39E-08	ID2	-0.356	2.22E-06	RYBP	-0.273	2.31E-05	HSPA5	0.388	1.62E-04	KLF5	-0.271	1.79E-03
SUMF2	0.447	2.91E-18	GCFC2	-0.404	1.01E-09	PLGLB2	-0.380	8.57E-08	TGFBR3	-0.357	2.28E-06	FOXO1	-0.381	2.31E-05	M6PR	0.302	1.67E-04	SLCO4C1	-0.392	1.80E-03
PER2	-0.649	8.29E-18	TBL1XR1	-0.523	1.01E-09	MCM4	0.361	8.67E-08	ITM2C	0.334	2.28E-06	RPS6KA6	-0.264	2.33E-05	PRCP	0.271	1.67E-04	BIRC3	0.329	1.84E-03
TCF7L2	-0.476	1.46E-17	RNF26	0.312	1.02E-09	FBN2	-0.423	8.99E-08	ISLR	0.401	2.30E-06	ADD3	-0.320	2.33E-05	TMTC1	-0.435	1.69E-04	ARL16	-0.281	1.84E-03
LASP1	0.417	1.62E-17	VPS18	0.315	1.02E-09	TMCO3	0.306	9.17E-08	GGT5	0.425	2.32E-06	TMEM161B	-0.288	2.36E-05	MRC2	0.336	1.71E-04	A1CF	-0.270	1.86E-03
COL3A1	0.866	1.78E-17	PRDM10	-0.295	1.04E-09	VCP	0.278	9.36E-08	SNRK	-0.337	2.34E-06	AADAT	-0.278	2.36E-05	APRT	0.276	1.72E-04	NBEAL1	-0.264	1.87E-03
DYRK1A	-0.355	1.81E-17	AEN	0.447	1.05E-09	KPNA2	0.399	9.69E-08	RELL1	-0.270	2.36E-06	PNRC1	-0.292	2.36E-05	STMN2	0.505	1.76E-04	VWA1	0.307	1.87E-03
CAPG	0.796	1.93E-17	RHOB	-0.678	1.08E-09	CD24	0.762	9.71E-08	NECTIN1	0.324	2.36E-06	MBLAC2	-0.279	2.40E-05	ECT2	0.264	1.77E-04	ZYG11A	-0.271	1.90E-03
ING1	-0.399	1.93E-17	LY6E	0.603	1.08E-09	GPNMB	0.727	1.01E-07	ZNF460	-0.303	2.37E-06	HS6ST1	0.297	2.50E-05	MTA1	-0.274	1.78E-04	NFIC	-0.300	1.91E-03
ZCCHC14	-0.427	6.96E-17	STYX	-0.494	1.09E-09	GINS2	0.444	1.03E-07	ITGBL1	0.589	2.38E-06	RWDD3	-0.451	2.52E-05	TMPRSS6	0.284	1.80E-04	CFHR4	-0.446	1.92E-03
RNF103	-0.404	8.88E-17	MRPS28	0.357	1.19E-09	PEX11G	0.298	1.04E-07	ZNF331	-0.314	2.38E-06	FNIP1	-0.324	2.53E-05	H2AJ	0.332	1.80E-04	DOP1B	-0.480	1.95E-03
PELI2	-0.661	9.84E-17	PSMC1	0.427	1.21E-09	SDC1	0.356	1.04E-07	EFNB3	0.274	2.38E-06	HEBP2	-0.279	2.53E-05	CCPG1	-0.290	1.82E-04	MBD3	0.338	1.96E-03
ITSN1	-0.355	9.84E-17	QSOX1	0.458	1.26E-09	COL25A1	-0.446	1.05E-07	PLP2	0.348	2.44E-06	CALM3	0.264	2.53E-05	NCOA4	0.283	1.82E-04	GEN1	-0.322	1.96E-03
LIX1L	-0.380	1.12E-16	HAL	-0.780	1.27E-09	ASB4	-0.380	1.05E-07	RASGEF1B	-0.336	2.44E-06	AOX1	-0.368	2.53E-05	PLPPR1	-0.314	1.85E-04	AURKAIP1	0.304	1.97E-03
FNDC5	1.024	1.16E-16	DUSP19	-0.342	1.29E-09	SLC22A10	-1.074	1.05E-07	LRRC39	-0.304	2.44E-06	ZNF160	-0.289	2.55E-05	DUSP6	-0.379	1.85E-04	IL17RB	-0.304	1.98E-03
PTGR2	-0.370	1.17E-16	HDAC11	0.340	1.32E-09	PRKG2	-0.306	1.09E-07	RNF217	-0.354	2.51E-06	SLC19A1	0.400	2.55E-05	TCN2	0.278	1.87E-04	ETFRF1	-0.333	1.98E-03
MYO16	-0.550	1.29E-16	GPD1	0.437	1.34E-09	KCNMA1	-0.526	1.11E-07	ZC3H6	-0.358	2.52E-06	VAMP1	0.307	2.55E-05	ZG16	0.345	1.88E-04	ENPP3	-0.299	1.98E-03
HSBP1L1	-0.606	1.84E-16	CCL14	-0.329	1.34E-09	UNC119	0.363	1.11E-07	CEP95	-0.322	2.54E-06	HSD17B11	-0.365	2.63E-05	COX17	-0.274	1.88E-04	SELENOP	0.389	2.00E-03
RFX5	0.440	2.36E-16	ROCK2	-0.318	1.36E-09	CCDC85C	0.336	1.11E-07	RELB	0.342	2.56E-06	RBMS2	-0.264	2.64E-05	SLC17A3	-0.336	1.92E-04	TSSC4	0.283	2.00E-03
MRAS	0.550	2.37E-16	MAF	-0.419	1.45E-09	INPP1	-0.275	1.11E-07	DUSP2	-0.300	2.57E-06	TXNRD1	0.269	2.64E-05	PRELID2	-0.280	1.92E-04	FBLN5	0.372	2.02E-03
ATP6V0D1	0.513	3.07E-16	TYMS	0.461	1.52E-09	PLSCR4	-0.460	1.15E-07	LRRC70	-0.296	2.60E-06	MYBL2	0.282	2.64E-05	AKAP5	-0.285	1.95E-04	NDUFB1	-0.290	2.02E-03
OSBPL6	-0.672	4.66E-16	RPS6KL1	0.422	1.57E-09	ZBTB25	-0.318	1.20E-07	STK31	-0.287	2.60E-06	CNOT6L	-0.267	2.65E-05	TUBB2A	0.348	1.99E-04	HLA-DQB1	0.368	2.03E-03
SPRYD3	0.452	6.48E-16	DNAJC25	-0.344	1.61E-09	PDK3	-0.356	1.21E-07	TRAF2	0.281	2.61E-06	NFKBIA	-0.291	2.69E-05	RTP3	0.339	2.00E-04	NKAIN3	-0.269	2.04E-03
SLC35B2	0.395	6.57E-16	AIG1	-0.273	1.61E-09	ZNF132	0.306	1.22E-07	TENT5A	-0.286	2.69E-06	PMP2	-0.288	2.70E-05	CARMIL3	-0.340	2.00E-04	AGR2	-0.346	2.05E-03
SLITRK3	-1.128	6.57E-16	TUBA1B	0.423	1.66E-09	ZNF544	0.320	1.22E-07	NFKBIE	0.282	2.74E-06	ESRRA	0.281	2.70E-05	HMGN3	-0.281	2.02E-04	SNORC	0.326	2.07E-03
ASPA	-0.674	8.61E-16	GAREM1	-0.409	1.70E-09	ZNF507	-0.269	1.28E-07	SLC22A24	-0.276	2.74E-06	MEGF10	-0.269	2.73E-05	COMP	0.308	2.08E-04	LSMEM1	-0.283	2.07E-03
TMEM41B	-0.482	1.13E-15	TSC22D1	0.427	1.71E-09	GPC1	0.431	1.33E-07	FDXR	0.367	2.82E-06	EIF3K	0.271	2.73E-05	CENPU	0.295	2.08E-04	TMEM98	0.282	2.09E-03
PRKAG2	-0.443	1.28E-15	HS3ST2	0.505	1.84E-09	CIDEC	0.433	1.36E-07	TRPM6	-0.280	2.94E-06	PPP1R1A	-0.475	2.73E-05	PRSS50	-0.296	2.08E-04	ITGA3	0.310	2.12E-03
KRT8	0.686	3.78E-15	BRAF	-0.267	1.84E-09	FOXO3	-0.338	1.36E-07	CUEDC1	0.268	3.04E-06	STK17A	0.274	2.73E-05	ZSCAN23	-0.329	2.09E-04	TIMP1	0.336	2.14E-03
PYGB	0.460	3.87E-15	ORC2	-0.417	1.87E-09	ARHGEF26	-0.439	1.37E-07	ETV7	0.326	3.09E-06	SPTAN1	0.285	2.74E-05	CDC42EP1	0.353	2.10E-04	PLIN4	0.394	2.16E-03
MMP14	0.569	4.70E-15	REPS1	-0.384	1.92E-09	FOXP1	-0.309	1.37E-07	GRIK2	-0.304	3.12E-06	TNFRSF12A	0.483	2.74E-05	CHST4	0.450	2.10E-04	KCNB1	0.453	2.18E-03
CNKSR2	-0.627	5.92E-15	IRS2	-0.672	1.93E-09	GTF2IRD2	-0.329	1.42E-07	ABCA5	-0.405	3.20E-06	CKS2	0.301	2.82E-05	TCAP	0.499	2.12E-04	CA14	-0.291	2.22E-03
IVNS1ABP	-0.421	7.31E-15	C19orf25	0.422	1.97E-09	SLC16A3	0.362	1.43E-07	HIPK3	-0.445	3.20E-06	PLCB1	-0.306	2.84E-05	CEBPA	0.323	2.13E-04	CLEC3B	0.280	2.24E-03
HPRT1	-0.643	7.77E-15	MYO1C	0.342	1.99E-09	LATS1	-0.304	1.43E-07	GIPC1	0.325	3.22E-06	CTBS	-0.315	2.89E-05	CLEC11A	0.279	2.15E-04	TMPRSS9	0.374	2.26E-03

DHRS7	0.707	7.77E-15	PKD1L1	-0.453	2.01E-09	ACVR2B	-0.338	1.44E-07	CHIC1	-0.431	3.22E-06	HECTD1	-0.284	2.91E-05	FOXN4	0.419	2.16E-04	ZIC1	-0.319	2.28E-03
CEBPB	-0.721	7.87E-15	OR2I1P	0.981	2.03E-09	DPP3	0.286	1.46E-07	PRKCE	0.682	3.23E-06	AKAP11	-0.335	2.93E-05	IFRD1	-0.319	2.20E-04	CCNL1	-0.289	2.28E-03
ATG13	0.287	8.00E-15	RDH12	-0.522	2.08E-09	KDM2B	-0.278	1.48E-07	TIMM17A	0.318	3.26E-06	LGALS1	0.429	2.93E-05	IDH2	0.294	2.20E-04	HLA-F	0.313	2.30E-03
CCL15	-0.426	9.36E-15	POLE3	0.292	2.12E-09	RTTN	-0.369	1.52E-07	PKM	0.387	3.30E-06	CPEB2	-0.333	3.03E-05	ITM2B	-0.264	2.21E-04	FBXW5	0.278	2.31E-03
FNBP1	-0.378	1.17E-14	CDHR2	0.757	2.20E-09	L3MBTL3	-0.342	1.52E-07	AHSG	0.313	3.35E-06	GLYAT	-0.266	3.06E-05	KCNC2	-0.320	2.24E-04	COX5B	-0.265	2.32E-03
BECN1	0.487	1.56E-14	XPNPEP3	-0.288	2.28E-09	PSMC3	0.273	1.52E-07	FABP5	0.424	3.41E-06	RDH5	0.319	3.06E-05	LDB2	-0.309	2.25E-04	HEYL	0.263	2.36E-03
ENPP1	-0.446	1.69E-14	TLNRD1	-0.323	2.32E-09	SENP6	-0.264	1.53E-07	CDC25A	0.274	3.42E-06	FBXO27	0.276	3.07E-05	PRDX4	0.290	2.26E-04	UGT2A3	0.368	2.38E-03
TAGLN2	0.530	1.97E-14	FBXL4	-0.383	2.32E-09	SLC39A3	0.324	1.54E-07	PMVK	0.330	3.48E-06	CCSER1	-0.312	3.08E-05	RFFL	0.274	2.26E-04	CYP17A1	-0.387	2.39E-03
CA13	-0.370	2.07E-14	PPP1R3G	-0.587	2.37E-09	LRRC8E	0.314	1.54E-07	PAIP2B	-0.292	3.48E-06	PDK4	-0.467	3.08E-05	UGT2B15	0.297	2.30E-04	BACH2	-0.310	2.41E-03
MAGED2	0.364	2.68E-14	CREM	-0.370	2.40E-09	WASL	-0.288	1.55E-07	HMGA1	0.316	3.50E-06	CAPS2	-0.266	3.11E-05	NPHP3	-0.352	2.32E-04	PCLO	0.305	2.44E-03
PPP1R15B	-0.375	2.68E-14	NHSL1	-0.372	2.42E-09	APOBEC3B	0.304	1.55E-07	ZNRF2	-0.304	3.56E-06	EEF2	0.291	3.15E-05	RIC3	-0.330	2.34E-04	COMT	0.268	2.44E-03
CD151	0.596	2.98E-14	BAX	0.411	2.48E-09	ABCB4	0.451	1.56E-07	CDO1	-0.266	3.60E-06	MIDN	-0.347	3.18E-05	C9	-0.377	2.34E-04	SLC7A2	-0.310	2.49E-03
CDNF	-0.417	3.01E-14	SERTAD2	-0.269	2.49E-09	ZNF280D	-0.326	1.62E-07	GAPDH	0.334	3.66E-06	SMIM14	-0.272	3.18E-05	NUB1	0.298	2.34E-04	ARL5B	-0.282	2.50E-03
NIBAN2	0.625	3.01E-14	MTR	-0.331	2.50E-09	BCL2L1	0.306	1.65E-07	IL1RN	-0.304	3.66E-06	CHI3L1	1.005	3.18E-05	EIF5B	-0.271	2.37E-04	ANKRD29	0.271	2.54E-03
ERN1	-0.573	3.36E-14	NEURL1	-0.399	2.57E-09	RAPH1	-0.461	1.65E-07	PKLR	0.453	3.66E-06	LTBP2	0.487	3.19E-05	ARFRP1	0.266	2.39E-04	HBA2	0.518	2.54E-03
CMYA5	-0.812	4.25E-14	KIAA1958	-0.324	2.62E-09	KCNMB4	-0.309	1.65E-07	NAMPT	-0.511	3.69E-06	HUNK	0.294	3.21E-05	SCRN2	0.289	2.42E-04	PALLD	-0.293	2.63E-03
MXI1	-0.424	4.32E-14	ZNF121	-0.359	2.62E-09	SMN1	-0.287	1.66E-07	ADCY10	-0.388	3.69E-06	RORA	-0.483	3.21E-05	ZFHX4	-0.272	2.45E-04	CIART	-0.337	2.67E-03
CD59	0.404	4.93E-14	OGA	-0.305	2.62E-09	ATXN7L1	-0.267	1.68E-07	BTAF1	-0.352	3.79E-06	FGD4	-0.357	3.24E-05	ARPC1B	0.294	2.48E-04	SCGN	0.332	2.69E-03
MUL1	0.392	5.74E-14	MVP	0.521	2.62E-09	DGKE	-0.301	1.70E-07	BLK	-0.285	3.83E-06	EMILIN1	0.414	3.27E-05	PCSK6	0.408	2.51E-04	PEG10	0.659	2.73E-03
CXXC4	-0.591	8.24E-14	CENPC	-0.346	2.62E-09	PDGFA	0.481	1.72E-07	SYNGR2	0.288	3.89E-06	ZDHHC12	0.336	3.27E-05	FBXO28	-0.269	2.51E-04	ACSM5	-0.265	2.74E-03
MAGI1	-0.362	9.00E-14	TLE4	-0.266	2.65E-09	TNKS1BP1	0.372	1.76E-07	PINK1	0.388	3.96E-06	FCN1	0.432	3.28E-05	NBPF15	0.345	2.58E-04	LYVE1	-0.527	2.81E-03
TIAM1	-0.452	1.03E-13	P4HA1	-0.841	2.66E-09	ACO2	0.441	1.76E-07	P3H2	-0.267	3.96E-06	GOLM1	0.430	3.41E-05	DNAH6	-0.365	2.59E-04	C2orf16	-0.271	2.86E-03
SQSTM1	0.434	1.20E-13	FOSB	-0.860	2.66E-09	PRC1	0.348	1.79E-07	FGFRL1	-0.353	3.97E-06	NR4A2	-0.356	3.42E-05	PRSS12	-0.299	2.60E-04	ZDHHC2	0.269	2.88E-03
PPP4R3A	-0.322	1.28E-13	TMEM129	0.369	2.70E-09	CDH17	-0.336	1.80E-07	H2AC6	0.378	4.01E-06	ALDH4A1	0.267	3.42E-05	DDX60L	-0.331	2.62E-04	PSAT1	-0.270	2.90E-03
SS18	-0.307	1.28E-13	HSP90AB1	0.297	2.73E-09	BCKDHB	-0.323	1.83E-07	ZDHHC19	-0.346	4.03E-06	COL10A1	0.380	3.42E-05	GJA4	0.302	2.66E-04	CYCS	0.267	2.90E-03
IPO13	0.344	1.28E-13	RGS7	-0.566	2.74E-09	MCCC2	-0.269	1.83E-07	CFAP251	-0.279	4.07E-06	HLA-DRB5	0.432	3.43E-05	CYP11A1	0.269	2.71E-04	PLA2G7	0.290	2.95E-03
LIN7C	-0.543	1.30E-13	EPPK1	0.517	2.85E-09	MPZL1	0.273	1.83E-07	GLIPR1L2	-0.300	4.10E-06	ZNF404	-0.296	3.47E-05	FPR3	0.403	2.72E-04	PDE1C	-0.305	2.95E-03
SOCS2	-0.845	1.44E-13	PSAP	0.352	2.86E-09	PDLIM5	-0.313	1.86E-07	SRSF4	-0.319	4.10E-06	C7orf50	0.344	3.49E-05	RNF138	-0.295	2.74E-04	UGT1A9	0.378	2.97E-03
DTNA	0.985	1.79E-13	RASL10A	-0.364	3.05E-09	CCDC68	-0.380	1.86E-07	SLC16A9	-0.422	4.10E-06	ACKR1	0.305	3.54E-05	NUSAP1	0.279	2.77E-04	APLNR	0.288	2.99E-03
KLHDC1	-0.512	1.99E-13	HPS6	0.283	3.19E-09	SETDB2	-0.316	1.90E-07	SOX18	-0.363	4.12E-06	MAP1LC3A	0.371	3.57E-05	SLC5A11	-0.287	2.78E-04	AGPAT2	0.320	3.00E-03
ENO1	0.401	2.03E-13	SIL1	0.356	3.39E-09	SULF2	0.493	1.90E-07	C9orf152	0.412	4.15E-06	CRYM	0.305	3.57E-05	ZGPAT	0.340	2.91E-04	TIMD4	-0.545	3.08E-03
RTCB	0.356	2.21E-13	ADGRA3	-0.444	3.40E-09	CAPN1	0.319	1.90E-07	SAT1	-0.291	4.15E-06	SPN	0.324	3.62E-05	DCHS2	-0.265	2.98E-04	SLC49A3	0.289	3.13E-03
CREB3	0.372	2.51E-13	GPAT3	-0.559	3.43E-09	TOP2A	0.544	1.92E-07	SCYL1	0.278	4.24E-06	MAN1C1	-0.333	3.62E-05	MT1M	-0.629	3.03E-04	CSRNP1	-0.285	3.17E-03
SNX11	0.308	2.64E-13	ESYT1	0.318	3.51E-09	EBPL	-0.418	1.96E-07	PPP3R1	-0.298	4.24E-06	SYBU	-0.461	3.65E-05	STAT1	0.352	3.06E-04	PDE11A	0.423	3.22E-03
FBXO46	-0.436	2.85E-13	FIBP	0.326	3.54E-09	TTC9	0.539	1.99E-07	CCM2	0.288	4.26E-06	GGT1	0.387	3.65E-05	ART5	-0.286	3.06E-04	EEA1	-0.293	3.25E-03
ARF3	0.427	2.87E-13	EPB41L5	-0.322	3.59E-09	MYL6	0.364	2.01E-07	THEM6	0.353	4.31E-06	ANAPC2	0.282	3.67E-05	GCLM	-0.274	3.12E-04	COL4A1	0.343	3.27E-03
FHIP1A	-0.495	2.87E-13	ZNF451	-0.327	3.65E-09	PDIA6	0.567	2.04E-07	KRT222	0.381	4.36E-06	CDS1	-0.338	3.72E-05	ERRFI1	-0.397	3.16E-04	CENATAC	-0.294	3.31E-03
HS1BP3	0.379	3.33E-13	EXOC1	-0.314	3.74E-09	DGKH	-0.385	2.13E-07	VPS13A	-0.378	4.38E-06	CLEC14A	0.306	3.73E-05	GALNT3	-0.275	3.17E-04	THRSP	0.371	3.34E-03
ZFAND4	-0.304	3.47E-13	MKI67	0.553	3.83E-09	RNASE1	0.537	2.21E-07	KRTCAP3	-0.393	4.38E-06	THOC1	-0.272	3.75E-05	CHAD	-0.375	3.20E-04	IFI27	0.432	3.36E-03
SMAD4	-0.301	3.68E-13	ELOVL1	0.295	3.88E-09	IFI27L1	0.315	2.24E-07	ENTPD8	0.357	4.39E-06	FOXA2	-0.294	3.82E-05	BMPR2	-0.272	3.21E-04	RASEF	0.315	3.37E-03
FANCL	-0.420	4.17E-13	MIOS	-0.278	4.00E-09	CHRM3	-0.604	2.32E-07	ANXA4	0.267	4.42E-06	PPARA	-0.269	3.82E-05	CCL20	0.392	3.28E-04	SOCS3	-0.354	3.37E-03
CALM1	-0.327	4.31E-13	ABCA9	-0.575	4.30E-09	LRRC59	0.342	2.32E-07	BRMS1	0.292	4.44E-06	GNE	-0.285	3.91E-05	SIGLEC1	-0.408	3.31E-04	VXN	-0.365	3.40E-03
P4HA2	0.422	4.37E-13	BICDL1	0.468	4.53E-09	PIGT	0.296	2.32E-07	FAM76B	-0.384	4.45E-06	NUDT22	0.351	3.92E-05	CETP	-0.564	3.32E-04	TMED6	-0.278	3.44E-03
ALG1	0.348	4.46E-13	PTPRD	-0.484	4.63E-09	ALG13	-0.278	2.32E-07	POGLUT2	0.268	4.45E-06	AJM1	0.301	3.93E-05	PROX1	-0.306	3.34E-04	DHRS2	-0.731	3.45E-03
VIL1	-1.402	4.62E-13	DUSP10	-0.436	4.67E-09	RASD1	-0.624	2.33E-07	PAXBP1	-0.355	4.48E-06	MMP15	0.318	3.93E-05	SNTG1	-0.298	3.38E-04	SLC6A2	0.422	3.45E-03
CENPV	-0.532	6.31E-13	OAZ2	0.273	4.67E-09	DNA2	-0.387	2.36E-07	CLIC1	0.281	4.48E-06	VEGFB	-0.307	4.05E-05	CYS1	0.283	3.41E-04	CDHR3	-0.264	3.53E-03
WEE1	-0.453	6.65E-13	KIAA1217	-0.336	4.67E-09	KIAA0930	0.275	2.39E-07	CXADR	-0.410	4.50E-06	TUBB4B	0.322	4.10E-05	ELOVL2	0.410	3.42E-04	MAN2A1	-0.268	3.58E-03
TNIK	-0.590	7.41E-13	LAP3	0.358	4.74E-09	ATG2B	-0.330	2.51E-07	CACFD1	0.333	4.59E-06	ARMC12	-0.321	4.13E-05	CCDC3	0.279	3.43E-04	WDR27	-0.280	3.68E-03
SIAH1	-0.469	7.41E-13	CD52	0.561	4.74E-09	GPHN	-0.277	2.54E-07	MKLN1	-0.264	4.59E-06	PMF1	-0.353	4.14E-05	ZNF580	-0.275	3.43E-04	SEMA6C	-0.352	3.70E-03
FCAMR	0.905	7.52E-13	VTN	0.385	4.75E-09	FAM83A	-0.304	2.54E-07	EXPH5	-0.402	4.76E-06	PGC	-0.346	4.17E-05	PKD2	-0.321	3.44E-04	TMEM178A	-0.291	3.70E-03
C11orf71	-0.376	7.63E-13	GTPBP10	-0.286	4.75E-09	INPP5B	-0.300	2.59E-07	AMOTL1	-0.332	4.82E-06	GCDH	-0.319	4.19E-05	F2RL1	0.330	3.44E-04	PPP1R3C	0.405	3.73E-03
ELL2	-0.509	8.08E-13	SLC9A7	-0.282	4.75E-09	RETREG1	-0.562	2.60E-07	AP1AR	-0.306	4.92E-06	ADIRF	0.377	4.23E-05	NDUFA2	-0.305	3.45E-04	SLC44A3	0.299	3.75E-03
PCDH20	-0.731	8.55E-13	PHF8	-0.434	4.76E-09	PGD	0.345	2.62E-07	RAB40B	-0.264	4.94E-06	SPINT2	0.414	4.34E-05	MED13	-0.297	3.46E-04	THBS2	0.356	3.75E-03
KCNT2	-0.564	8.56E-13	GPATCH4	0.326	4.83E-09	SPAG9	-0.321	2.63E-07	ARHGAP30	0.357	4.94E-06	CLEC4M	-0.544	4.35E-05	CGREF1	0.290	3.50E-04	SFRP4	0.268	3.79E-03

INHBE	0.886	8.88E-13	HORMAD2	-0.473	5.05E-09	ALDH6A1	-0.490	2.63E-07	SLC6A16	-0.418	4.97E-06	SOX5	-0.346	4.36E-05	HECW2	-0.310	3.51E-04	LAD1	0.275	3.83E-03
RPS10	-0.397	8.88E-13	CDKN1C	-0.390	5.33E-09	MON2	-0.368	2.69E-07	NUDT1	0.264	5.07E-06	PPIB	0.285	4.37E-05	CAP1	0.342	3.54E-04	MT1F	-0.532	3.98E-03
IGSF3	0.566	8.88E-13	ZWINT	0.466	5.60E-09	UBE3D	-0.276	2.89E-07	LGSN	-0.402	5.11E-06	EPOR	-0.356	4.37E-05	ARMC6	0.294	3.64E-04	METRN	0.285	4.13E-03
ZFAND5	-0.658	9.49E-13	CADM2	-0.429	5.76E-09	NAT1	-0.303	2.90E-07	CDH23	-0.451	5.15E-06	GRID1	0.559	4.38E-05	ABCC4	0.330	3.65E-04	DDAH1	-0.265	4.15E-03
HEY2	-0.349	9.62E-13	FAM111B	0.640	5.86E-09	APOF	-0.445	2.90E-07	APOLD1	-0.389	5.15E-06	RFX1	-0.319	4.38E-05	KRT7	0.494	3.71E-04	ERICH5	0.286	4.21E-03
BLNK	-0.456	1.04E-12	LSP1	0.479	5.93E-09	PHC3	-0.369	2.90E-07	CYB5R3	0.287	5.20E-06	SPSB4	-0.288	4.42E-05	DOK6	-0.436	3.74E-04	EDA2R	0.282	4.37E-03
TMEM102	0.385	1.10E-12	LRRC20	0.373	6.16E-09	PCSK5	0.395	2.94E-07	C11orf96	-0.391	5.21E-06	HLA-DMB	0.389	4.43E-05	SLC25A10	0.297	3.78E-04	USH2A	-0.360	4.37E-03
RAB7B	0.486	1.17E-12	DISP1	-0.274	6.18E-09	N4BP2L2	-0.305	3.06E-07	FAM241A	-0.332	5.25E-06	WDR86	0.297	4.43E-05	PPM1E	-0.298	3.80E-04	GOLGA6B	-0.263	4.40E-03
SNX12	0.324	1.24E-12	MMP9	0.582	6.45E-09	TMEM214	0.266	3.10E-07	LIMCH1	-0.337	5.28E-06	SULT1E1	-0.469	4.44E-05	FLNA	0.413	3.80E-04	EEF1A2	0.473	4.49E-03
ANKRD13C	-0.304	1.31E-12	CREBBP	-0.329	6.52E-09	GPC5	-0.411	3.12E-07	MED13L	-0.310	5.29E-06	SREK1IP1	-0.297	4.49E-05	C19orf18	-0.271	3.87E-04	KDM8	0.286	4.58E-03
FABP4	1.144	1.31E-12	PPFIA3	0.367	6.54E-09	B3GAT1	-0.885	3.24E-07	SOD1	-0.305	5.31E-06	HIVEP1	-0.328	4.49E-05	HYOU1	0.353	3.87E-04	MUC5B	-0.368	4.63E-03
GABPB1	-0.327	1.35E-12	CFL1	0.356	6.58E-09	BRIP1	-0.363	3.29E-07	SLCO1B3	-0.748	5.32E-06	MAMDC4	0.634	4.50E-05	ARHGEF37	0.352	3.93E-04	COL4A4	0.277	4.66E-03
PPP2R2A	-0.353	1.67E-12	FERMT2	-0.359	6.62E-09	DICER1	-0.370	3.34E-07	EHD4	0.272	5.33E-06	LRP4	-0.329	4.52E-05	FAM171A1	-0.283	4.03E-04	ELF3	0.322	4.71E-03
MSN	0.533	1.72E-12	TBCEL	-0.390	6.65E-09	TYMP	0.508	3.44E-07	DCN	0.412	5.33E-06	RIMBP2	-0.484	4.61E-05	WDPCP	0.309	4.03E-04	IDUA	-0.283	4.77E-03
TDRP	-0.472	1.72E-12	GPR137	0.358	6.65E-09	TUBG1	0.283	3.46E-07	GLB1	0.307	5.33E-06	GMPPB	0.337	4.61E-05	NEGR1	-0.303	4.04E-04	LRP3	0.281	4.97E-03
BAK1	0.388	1.78E-12	MAGI2	-0.390	6.70E-09	PBLD	-0.309	3.46E-07	TMCO6	-0.295	5.35E-06	GRB14	-0.320	4.64E-05	ZNF680	-0.320	4.04E-04	DGAT2	0.297	5.05E-03
CDK19	-0.339	2.07E-12	MAD2L2	0.364	6.97E-09	CHMP1A	0.273	3.49E-07	GADD45B	-0.581	5.38E-06	WNT4	0.346	4.64E-05	SMTN	-0.298	4.08E-04	CCDC57	0.282	5.06E-03
IDS	-0.320	2.17E-12	SCPEP1	0.346	7.19E-09	SH3PXD2A	-0.595	3.50E-07	APOBR	0.311	5.40E-06	PRAMEF33	0.422	4.69E-05	RO60	-0.279	4.10E-04	MAB21L4	0.598	5.16E-03
MTHFD2L	-0.645	2.40E-12	DMTN	0.508	7.23E-09	DEPDC7	-0.382	3.62E-07	HLA-DOA	0.424	5.45E-06	HELZ	-0.267	4.71E-05	HBB	0.796	4.13E-04	SLC26A1	0.264	5.19E-03
DNAJC12	-0.552	2.75E-12	SNX17	0.265	7.66E-09	SLC16A13	0.496	3.63E-07	KLHL8	-0.279	5.51E-06	FSTL3	0.386	4.71E-05	HLA-DQA1	0.565	4.13E-04	HSPE1	-0.317	5.23E-03
C2CD5	-0.400	3.03E-12	NRXN1	-0.519	7.76E-09	GET3	0.312	3.69E-07	RIMOC1	-0.344	6.01E-06	PACSIN3	-0.386	4.85E-05	PLEKHA6	-0.275	4.13E-04	PTH2R	-0.289	5.25E-03
PRKAA1	-0.435	3.58E-12	DDRGK1	0.368	7.76E-09	PRXL2B	0.288	3.90E-07	RBM24	-0.266	6.02E-06	TSPAN13	-0.596	4.87E-05	ATP13A3	-0.348	4.23E-04	PCK1	-0.523	5.30E-03
RREB1	-0.462	3.63E-12	CBFB	-0.377	7.76E-09	TPP2	-0.282	4.05E-07	CPEB4	-0.441	6.02E-06	IFI27L2	0.286	4.94E-05	CAPN2	0.272	4.24E-04	SULT1C2	0.324	5.33E-03
NATD1	0.344	3.69E-12	KLHL11	-0.322	7.81E-09	CCR5	0.367	4.05E-07	DTWD1	-0.281	6.04E-06	FAAH2	-0.281	4.97E-05	BMF	0.290	4.31E-04	ARMH1	-0.273	5.43E-03
PHF23	0.372	3.88E-12	PAQR7	0.323	7.92E-09	BASP1	-0.435	4.33E-07	PHLPP1	-0.301	6.16E-06	IRAK3	-0.294	5.00E-05	LEAP2	0.312	4.34E-04	TCAF1	0.284	5.45E-03
CYP2C19	-1.577	3.88E-12	SLC4A2	0.360	8.10E-09	TK1	0.398	4.40E-07	RHBDD3	0.358	6.16E-06	FOXN2	-0.334	5.05E-05	NT5E	-0.284	4.34E-04	SLC6A13	-0.311	5.49E-03
RPL39	-0.774	3.98E-12	ZNF468	0.451	8.12E-09	DCT	-0.357	4.40E-07	YIF1B	0.300	6.41E-06	LAMC3	0.540	5.07E-05	KCNJ2	-0.323	4.42E-04	KLF6	-0.310	5.50E-03
APOL3	0.760	4.02E-12	TOR3A	0.449	8.26E-09	TLCD4	-0.472	4.40E-07	ABCA10	-0.499	6.59E-06	AGL	-0.377	5.08E-05	HLA-DRA	0.415	4.45E-04	RGS2	-0.268	5.69E-03
WWC1	0.447	4.47E-12	ERGIC1	0.313	9.01E-09	SLCO1A2	-0.814	4.50E-07	BANF1	0.303	6.74E-06	RBBP9	0.313	5.09E-05	SCN2A	-0.267	4.45E-04	OAS2	0.327	5.89E-03
TTC6	-0.564	4.47E-12	CARNMT1	-0.391	9.05E-09	C1QTNF3	-0.362	4.52E-07	RALGPS1	0.339	6.80E-06	RANGAP1	0.277	5.09E-05	THAP5	-0.270	4.45E-04	SLC35C1	0.295	6.08E-03
EML2	0.317	4.47E-12	ZC3H4	-0.325	9.08E-09	AQP1	0.715	4.62E-07	RET	0.615	6.99E-06	SLC23A2	-0.388	5.13E-05	ITPR2	-0.269	4.45E-04	C4A	0.363	6.14E-03
ADCK2	0.384	4.93E-12	TNR	-0.492	9.12E-09	DCAF16	-0.269	4.62E-07	MIDEAS	-0.273	7.15E-06	COL28A1	-0.522	5.20E-05	KLF13	0.291	4.48E-04	WNK3	-0.286	6.14E-03
PARD6B	-0.491	4.93E-12	PDCL3	0.279	9.33E-09	PPP1R9B	0.313	4.71E-07	MEOX1	0.295	7.24E-06	OGT	-0.311	5.23E-05	TPD52	-0.316	4.49E-04	COL16A1	0.319	6.26E-03
PPM1K	-0.591	5.83E-12	TRABD2B	-0.623	9.61E-09	TRIB2	-0.270	4.71E-07	RBM39	-0.276	7.32E-06	SCD	0.699	5.31E-05	AACS	0.274	4.57E-04	SC5D	-0.321	6.32E-03
PSEN2	0.297	6.42E-12	CEP120	-0.305	9.63E-09	GALNT18	0.375	4.74E-07	SPATA6	-0.273	7.35E-06	KIRREL1	0.309	5.32E-05	CDH19	-0.306	4.63E-04	CA12	0.418	6.41E-03
RBMS1	-0.500	6.44E-12	COL1A1	0.863	9.81E-09	CKAP4	0.275	4.74E-07	CCDC107	0.283	7.38E-06	RAB32	0.269	5.33E-05	KLF2	-0.271	4.64E-04	SLC12A1	0.322	6.52E-03
PCBP2	-0.329	6.98E-12	SMPDL3A	-0.432	1.02E-08	DPY19L3	-0.279	4.81E-07	GRAMD1C	-0.344	7.42E-06	OAS1	0.349	5.36E-05	FLT4	-0.346	4.66E-04	OASL	0.281	6.72E-03
GADD45G	-0.991	7.51E-12	CYB5R2	0.404	1.04E-08	CXCL2	-0.756	4.86E-07	LBH	0.375	7.42E-06	EBF4	-0.299	5.37E-05	PHTF2	-0.286	4.68E-04	CCN1	-0.364	6.83E-03
DNAJC19	-0.406	8.41E-12	PPP1CA	0.291	1.07E-08	HDAC4	-0.266	4.93E-07	UGCG	-0.324	7.60E-06	DAO	0.315	5.38E-05	TRIR	-0.272	4.73E-04	ELFN2	0.304	6.91E-03
SLC19A2	-0.572	8.59E-12	C1GALT1	-0.385	1.09E-08	OLFML3	0.359	4.95E-07	SAMD5	-0.432	7.74E-06	PLXNC1	0.314	5.43E-05	SLC25A27	-0.407	4.78E-04	NSDHL	0.309	7.15E-03
ALDH18A1	0.466	8.96E-12	NAAA	-0.307	1.12E-08	RCL1	-0.360	4.99E-07	UNC119B	0.264	7.85E-06	XRN1	-0.351	5.47E-05	ABCC9	-0.327	5.03E-04	ABCB11	-0.266	7.26E-03
KCNJ5	0.513	9.27E-12	FRMD4B	-0.331	1.13E-08	KHK	0.341	5.03E-07	TMEM233	-0.270	7.87E-06	COL14A1	0.441	5.49E-05	JAKMIP2	-0.295	5.11E-04	CPS1	-0.277	7.50E-03
IMPA1	-0.561	9.75E-12	FOXP2	-0.403	1.14E-08	FOS	-1.036	5.03E-07	RC3H1	-0.291	7.94E-06	MARVELD1	0.268	5.51E-05	NEK10	-0.613	5.11E-04	CDHR5	0.271	7.58E-03
ARSK	-0.411	1.04E-11	DHRS9	0.456	1.14E-08	ACADL	-0.279	5.07E-07	MCC	-0.329	8.02E-06	AP3S1	-0.273	5.55E-05	DPY19L4	-0.287	5.19E-04	EMP1	0.400	7.58E-03
LPL	0.872	1.07E-11	RACGAP1	0.301	1.14E-08	ANO7	-0.295	5.07E-07	ZNF710	0.373	8.11E-06	EGLN3	-0.284	5.64E-05	HLA-DRB1	0.489	5.21E-04	ADGRE1	-0.304	7.62E-03
PSMD2	0.346	1.08E-11	PER3	-0.620	1.14E-08	CYB561	0.307	5.12E-07	CMC4	-0.333	8.21E-06	ANKRD10	-0.297	5.68E-05	HLA-C	0.303	5.27E-04	VTCN1	0.270	7.74E-03
SEPTIN6	0.360	1.12E-11	CYP2B6	-0.704	1.14E-08	TAS2R20	-0.291	5.13E-07	DYNLT1	0.289	8.47E-06	VAT1L	-0.268	5.68E-05	DKK3	0.376	5.28E-04	UBXN10	0.332	7.77E-03
ANGEL2	-0.306	1.18E-11	NEURL1B	0.375	1.15E-08	HYAL3	0.362	5.13E-07	AKR1B10	1.292	8.65E-06	CLIP2	0.288	5.69E-05	UBE2D1	-0.271	5.36E-04	NES	0.273	7.85E-03
FBXO21	-0.413	1.18E-11	ATG7	0.267	1.17E-08	ZCCHC9	-0.278	5.14E-07	HEATR5B	-0.302	8.67E-06	RASGRP2	-0.369	5.73E-05	SPRY4	-0.306	5.40E-04	ME1	0.338	8.06E-03
FBXO11	-0.388	1.26E-11	CAMK2D	-0.331	1.17E-08	EPB41L4B	-0.301	5.14E-07	IL4I1	0.294	8.74E-06	CNTLN	-0.291	5.78E-05	ADAMTS16	0.298	5.42E-04	MRGPRF	0.271	8.07E-03
ADAM15	0.415	1.33E-11	KBTBD11	-0.458	1.18E-08	EGFLAM	-0.419	5.14E-07	KCNAB1	-0.316	8.78E-06	RPL10	0.289	5.79E-05	SETD1B	-0.296	5.50E-04	NR1D2	-0.341	8.10E-03
ABHD18	-0.321	1.53E-11	ZDHHC17	-0.383	1.21E-08	MB	0.538	5.16E-07	GNG5	-0.281	8.79E-06	SPATA18	0.408	5.80E-05	NTN4	-0.304	5.55E-04	LINGO4	0.287	8.18E-03
OPTN	0.345	1.58E-11	ESPNL	-0.601	1.21E-08	EMP3	0.311	5.21E-07	CMC2	-0.325	8.81E-06	PLP1	-0.384	5.80E-05	TAT	-0.516	5.62E-04	ZDHHC11	-0.315	8.22E-03
TMEM63A	0.478	1.61E-11	ACADSB	-0.521	1.22E-08	PSMB7	0.307	5.36E-07	CPNE6	-0.390	8.91E-06	ANKHD1	-0.407	5.85E-05	NIPAL1	-0.354	5.67E-04	DCPS	-0.265	8.64E-03

ADHFE1	-0.460	1.61E-11	GAB1	-0.326	1.24E-08	TDRD10	-0.456	5.42E-07	MFSD4B	-0.287	8.92E-06	BST2	0.449	5.90E-05	PRKAA2	0.407	5.71E-04	MMRN1	-0.369	8.66E-03
FOXJ3	-0.284	1.61E-11	PURB	-0.291	1.25E-08	NPAS3	-0.285	5.47E-07	AFG3L2	0.284	8.94E-06	IGFBPL1	0.330	5.90E-05	LUM	0.517	5.72E-04	C8G	0.280	8.68E-03
SPIDR	-0.293	1.61E-11	CHPF2	0.290	1.31E-08	CYB561D2	0.267	5.47E-07	ZNF684	-0.293	9.04E-06	GPR88	-0.632	5.93E-05	RGS5	-0.400	5.72E-04	CAPN3	-0.338	8.80E-03
NKRF	-0.300	1.72E-11	MR1	0.298	1.36E-08	PGK1	0.284	5.49E-07	GABRG3	-0.271	9.08E-06	IFRD2	0.270	6.04E-05	NKD1	0.270	5.83E-04	STAB2	-0.381	8.80E-03
TAF4	-0.326	2.04E-11	FAM76A	-0.290	1.44E-08	SVEP1	0.468	5.63E-07	GRIA3	0.305	9.12E-06	SP140	0.316	6.05E-05	MEI4	-0.326	5.84E-04	CSTA	0.328	8.86E-03
TRIM24	-0.315	2.10E-11	POGZ	-0.388	1.46E-08	XKR4	-0.402	5.63E-07	PLD6	-0.274	9.13E-06	NOTCH4	-0.388	6.23E-05	KDM6A	-0.273	5.89E-04	CHL1	-0.350	8.88E-03
ZFP1	-0.508	2.43E-11	USP6NL	-0.305	1.50E-08	TTLL7	-0.415	5.99E-07	FAM200B	-0.277	9.14E-06	FBXL18	0.280	6.24E-05	ANXA13	0.518	5.89E-04	MUC1	-0.274	9.34E-03
IFT88	-0.346	2.50E-11	MTUS1	-0.328	1.54E-08	NUDT18	0.286	6.24E-07	EDN2	0.313	9.18E-06	RHOBTB1	0.326	6.34E-05	TST	0.264	5.90E-04	MTUS2	-0.307	9.78E-03
C6orf47	0.294	2.51E-11	STK3	-0.310	1.54E-08	ACP5	0.483	6.35E-07	SORBS1	-0.264	9.27E-06	PPP2R1B	-0.332	6.39E-05	EARS2	0.267	6.08E-04	ADGRL4	-0.278	1.01E-02
USP5	0.320	2.65E-11	AGTR1	-0.376	1.54E-08	BTN3A3	0.275	6.39E-07	THY1	0.597	9.27E-06	SERINC2	0.337	6.44E-05	CLCN7	0.295	6.09E-04	SHANK3	-0.272	1.04E-02
PI4K2A	0.335	2.90E-11	HTRA1	0.363	1.54E-08	ABCA1	-0.492	6.81E-07	NPIPA2	-0.279	9.41E-06	LGI1	-0.483	6.44E-05	CLDN10	0.371	6.14E-04	ACE2	0.271	1.05E-02
SLX4IP	-0.344	2.92E-11	PGAP4	0.436	1.54E-08	EEF2KMT	0.267	6.81E-07	LCORL	-0.271	9.51E-06	ATP1B2	-0.275	6.44E-05	GFRA2	-0.290	6.16E-04	CISH	-0.386	1.07E-02
ZBTB10	-0.497	2.92E-11	TRAF7	0.369	1.54E-08	UBR3	-0.351	6.88E-07	CHMP4A	0.264	9.69E-06	MPC1	-0.308	6.48E-05	KDELR3	0.348	6.45E-04	CPT1A	-0.281	1.07E-02
SLC25A37	-0.494	2.94E-11	UBL7	0.273	1.59E-08	ZMAT3	0.484	6.90E-07	CYGB	0.367	9.70E-06	MCM2	0.368	6.54E-05	NFKBIZ	-0.300	6.47E-04	PTGDS	0.416	1.10E-02
CTSD	0.618	2.94E-11	MRPS30	-0.288	1.59E-08	ZNF804A	-0.313	6.90E-07	SPSB1	0.420	9.73E-06	MT2A	-0.550	6.61E-05	TFCP2L1	-0.337	6.48E-04	LRATD1	-0.302	1.12E-02
FAM9B	-0.671	3.27E-11	LBR	-0.396	1.61E-08	PTPRU	0.410	7.05E-07	IKBKG	0.296	9.81E-06	SLC25A18	-0.447	6.72E-05	CYP2A7	-0.699	6.54E-04	MME	-0.267	1.15E-02
PHLDA3	0.644	3.50E-11	GRN	0.484	1.62E-08	SLC39A5	0.379	7.16E-07	CYP4F22	0.443	9.87E-06	TAPBPL	0.277	6.83E-05	CFTR	0.483	6.57E-04	NDST3	-0.295	1.15E-02
FAT1	0.780	3.50E-11	MET	-0.422	1.63E-08	NALCN	0.450	7.20E-07	RALGAPA1	-0.298	9.94E-06	NPM2	0.295	6.89E-05	RAD54L2	-0.276	6.57E-04	ZNF385D	0.362	1.17E-02
RUNDC3B	-0.474	3.67E-11	SMOC1	-0.389	1.70E-08	ISM1	0.508	7.30E-07	RHOC	0.294	1.00E-05	SEC11C	0.317	6.91E-05	GET1	-0.264	6.63E-04	LEPR	-0.368	1.25E-02
IFI30	0.584	3.80E-11	TAPT1	-0.280	1.71E-08	GATA6	-0.302	7.34E-07	TRIM22	0.381	1.02E-05	VCAN	0.451	6.99E-05	CORO1A	0.302	6.70E-04	ADAMTS1	-0.265	1.25E-02
DCBLD2	-0.361	3.92E-11	HERC5	-0.483	1.77E-08	ZNF770	-0.338	7.34E-07	SLC7A5	0.307	1.03E-05	CFL2	-0.300	7.04E-05	PPIF	0.355	6.77E-04	C6orf141	-0.277	1.28E-02
CDK4	0.364	4.06E-11	LGALS3	0.542	1.82E-08	IRAK1	0.296	7.37E-07	GLRX5	-0.293	1.04E-05	CD81	0.267	7.07E-05	LILRB5	-0.388	6.81E-04	FCN3	0.328	1.28E-02
RP1	-0.471	4.27E-11	FNIP2	-0.597	1.83E-08	SNAP25	0.447	7.37E-07	RICTOR	-0.382	1.06E-05	TDO2	-0.387	7.08E-05	HLA-DMA	0.275	6.82E-04	MOXD1	0.303	1.30E-02
AFF3	-0.525	4.34E-11	TRMT10A	-0.295	1.86E-08	OLFM2	0.857	7.53E-07	SEMA6A	-0.329	1.06E-05	ADGRG6	-0.347	7.09E-05	LTK	-0.310	6.99E-04	CYP3A5	-0.366	1.30E-02
CHRAC1	-0.310	4.52E-11	EIF3G	0.330	1.97E-08	EPHA1	0.349	7.63E-07	ADAMTSL2	0.528	1.07E-05	POLR2L	0.393	7.24E-05	MVK	0.339	7.16E-04	UNC93A	0.395	1.31E-02
GAS6	0.597	4.77E-11	MTARC2	-0.309	1.98E-08	TSNAX	-0.311	7.75E-07	PTPRS	-0.561	1.08E-05	ITCH	-0.264	7.30E-05	N4BP2L1	-0.284	7.45E-04	NRG1	0.366	1.33E-02
LGALS3BP	0.680	5.20E-11	RNF152	-0.668	1.98E-08	CHST9	0.643	7.75E-07	DDOST	0.277	1.09E-05	SMLR1	-0.275	7.49E-05	DUSP23	-0.296	7.58E-04	NFASC	0.290	1.37E-02
IRF2BP2	-0.392	5.50E-11	NUDT19	-0.309	1.99E-08	DDX54	0.306	7.85E-07	PABIR2	-0.285	1.10E-05	PPARGC1A	-0.525	7.54E-05	RGPD2	-0.298	7.60E-04	ANKRD18A	0.287	1.43E-02
PRKRA	-0.344	5.57E-11	PDS5A	-0.274	2.05E-08	CARD8	-0.289	7.91E-07	PCOLCE2	-0.439	1.11E-05	IGF1	-0.560	7.80E-05	IER2	-0.311	7.63E-04	USP9Y	0.861	1.43E-02
TMEM140	0.358	5.97E-11	SPHK2	0.435	2.08E-08	CHD1	-0.309	7.91E-07	REEP6	0.404	1.11E-05	UGT2B10	0.337	7.81E-05	TRIM2	-0.313	7.68E-04	IGLL5	0.323	1.48E-02
CTNNA3	-0.751	6.13E-11	SLC16A10	-0.463	2.09E-08	SLCO1B3-SL	-0.527	8.04E-07	IGFBP2	-0.982	1.14E-05	EP400	-0.278	7.82E-05	TRPV4	0.286	7.74E-04	MEP1B	0.352	1.53E-02
MEF2A	-0.352	6.39E-11	ST14	0.507	2.13E-08	PRPF39	-0.347	8.16E-07	TSR3	0.331	1.14E-05	AUTS2	-0.280	7.84E-05	ERV3-1	0.298	7.78E-04	TXLNA	0.272	1.54E-02
PPP3CB	-0.281	6.56E-11	C9orf43	-0.309	2.16E-08	KCNN2	-0.486	8.29E-07	SALL4	-0.367	1.14E-05	SCTR	0.322	8.02E-05	SLC3A1	-0.670	7.81E-04	LRP1B	-0.391	1.56E-02
NOTCH2NLA	-0.391	6.91E-11	SCN11A	-0.328	2.24E-08	F11	-0.315	8.29E-07	PRIMA1	-0.294	1.16E-05	REELD1	-0.325	8.11E-05	LOXL4	0.407	7.81E-04	HSD17B3	-0.282	1.56E-02
TRIT1	-0.298	7.08E-11	ZNF638	-0.325	2.24E-08	MGLL	0.333	8.32E-07	CALR	0.322	1.17E-05	SNAI2	-0.309	8.23E-05	HAAO	-0.264	7.85E-04	MMP2	0.296	1.57E-02
CLXN	-0.409	7.52E-11	ASTN2	0.302	2.24E-08	DERL3	0.351	8.51E-07	SPTBN4	-0.265	1.18E-05	FHIP2A	-0.281	8.27E-05	HLA-DPA1	0.394	7.94E-04	GNMT	-0.301	1.67E-02
COL1A2	0.769	7.69E-11	SERPINA5	0.478	2.27E-08	NR5A2	-0.304	8.83E-07	ALDH1B1	0.375	1.19E-05	CBLN4	-0.328	8.43E-05	MDM4	-0.335	8.15E-04	EPHA3	0.294	1.71E-02
ZNF280C	-0.344	7.94E-11	PCSK2	-0.344	2.30E-08	BNIP3	-0.323	9.07E-07	TPPP2	-0.352	1.19E-05	NR4A1	-0.397	8.53E-05	PODN	0.375	8.39E-04	EIF1AY	0.842	1.76E-02
CYB561A3	0.375	8.14E-11	ERBB2	0.421	2.38E-08	PFKL	0.401	9.09E-07	DIRAS3	-0.367	1.22E-05	ITGB2	0.375	8.56E-05	TDRD6	-0.298	8.46E-04	GOLGA8A	-0.416	1.79E-02
MED22	0.324	8.25E-11	CCDC178	-0.293	2.47E-08	PGAP1	-0.351	9.14E-07	GCA	-0.265	1.23E-05	COPE	0.313	8.56E-05	PZP	-1.058	8.53E-04	SDF2L1	0.265	1.83E-02
AFF1	-0.402	8.62E-11	KCNC4	-0.357	2.54E-08	CUL4B	-0.291	9.27E-07	SORCS1	-0.340	1.24E-05	CTSS	0.389	8.66E-05	ALPK2	0.633	8.75E-04	SEMA3G	0.274	1.84E-02
ANO10	0.341	8.66E-11	ATP6V1F	0.321	2.55E-08	TUT7	-0.436	9.27E-07	LAMB3	0.348	1.27E-05	HOGA1	-0.283	8.67E-05	IGSF23	-0.321	8.97E-04	ANGPTL8	0.348	1.88E-02
PABPC4	-0.283	8.81E-11	TFPI	-0.408	2.67E-08	FADS2	0.912	9.27E-07	ZNF518A	-0.332	1.28E-05	NPR3	0.334	8.78E-05	UBE2L6	0.264	9.19E-04	C5	-0.273	1.88E-02
SPRING1	0.367	8.88E-11	MEIOB	-0.293	2.71E-08	CSNK1G3	-0.291	9.33E-07	SDF4	0.290	1.29E-05	SLC16A12	-0.438	8.78E-05	FAM124B	-0.361	9.37E-04	CXCL6	0.272	1.90E-02
BCAP31	0.348	9.68E-11	PAN3	-0.428	2.73E-08	EPDR1	0.321	9.35E-07	FRA10AC1	-0.296	1.30E-05	MICU3	-0.391	8.80E-05	BBLN	0.331	9.40E-04	S100A9	0.333	1.91E-02
SPP1	1.110	9.68E-11	LRRC31	0.556	2.75E-08	DCTPP1	0.290	9.37E-07	NOTCH2NLC	-0.317	1.32E-05	PTPRH	0.377	8.89E-05	MCM5	0.289	9.40E-04	SFRP5	0.311	1.97E-02
VMA21	-0.340	9.70E-11	SKP2	-0.301	2.75E-08	UNC5B	0.360	9.38E-07	ITGA6	0.338	1.32E-05	SP4	-0.286	9.10E-05	OAS3	0.365	9.49E-04	TMEM45B	0.355	2.00E-02
LAMA3	0.636	1.07E-10	MAJIN	-0.337	2.82E-08	RAD54B	-0.316	9.50E-07	PSKH1	0.295	1.32E-05	ELOVL5	0.326	9.21E-05	HSPA1A	-0.318	9.52E-04	EVC	0.344	2.03E-02
PELI1	-0.376	1.22E-10	SLC15A2	-0.347	2.83E-08	TMEM104	0.305	9.56E-07	GREM2	-0.300	1.34E-05	SRD5A1	-0.271	9.27E-05	VWDE	-0.286	9.53E-04	MT1H	-0.528	2.03E-02
TSPAN5	-0.452	1.25E-10	CFAP69	-0.406	2.84E-08	RFX3	-0.295	9.66E-07	DSCAM	-0.278	1.34E-05	UGT3A2	0.302	9.59E-05	CUX2	-0.559	9.71E-04	HSD17B14	-0.371	2.07E-02
KANSL1L	-0.348	1.25E-10	TMBIM1	0.326	2.88E-08	PRXL2A	0.325	9.67E-07	LAMA2	0.450	1.34E-05	TMEM220	-0.269	9.63E-05	EPSTI1	0.314	9.84E-04	NNMT	-0.451	2.14E-02
LURAP1L	-0.529	1.25E-10	PDGFRB	0.467	2.90E-08	CYP2C8	-0.368	9.77E-07	POLR2E	0.318	1.34E-05	SIK2	-0.266	9.65E-05	MYC	-0.293	9.87E-04	ANKRD36C	0.263	2.21E-02
ARHGEF4	-0.500	1.25E-10	SLC29A1	0.358	3.07E-08	GRTP1	-0.322	1.00E-06	GREB1L	-0.298	1.39E-05	SEMA6B	-0.285	9.66E-05	CD74	0.368	9.97E-04	UGT1A3	-0.302	2.28E-02
BICRAL	-0.296	1.29E-10	PRX	0.290	3.07E-08	LGALS4	0.922	1.02E-06	SATB1	-0.269	1.40E-05	MREG	-0.359	9.83E-05	ARRDC3	-0.400	9.99E-04	SAA1	0.568	2.30E-02

YAE1	-0.310	1.37E-10	SYDE2	-0.310	3.12E-08	EMP2	-0.286	1.04E-06	IDNK	-0.275	1.41E-05	NTAN1	0.285	9.97E-05	EFHD1	-0.636	1.01E-03	GBP3	0.335	2.32E-02
NFE2L2	-0.333	1.38E-10	FBXO6	0.316	3.18E-08	EYA4	-0.329	1.05E-06	PPA1	0.280	1.41E-05	TF	0.269	9.97E-05	HOOK1	-0.272	1.02E-03	HYDIN	-0.481	2.33E-02
ZSWIM6	-0.364	1.52E-10	CXCL9	0.846	3.22E-08	MALT1	-0.301	1.05E-06	MLST8	0.287	1.43E-05	GPRC5A	-0.274	9.98E-05	CLK1	-0.284	1.03E-03	KCNJ10	-0.278	2.37E-02
CCDC196	-0.499	1.56E-10	TUSC1	-0.299	3.33E-08	AMY2B	-0.440	1.07E-06	MLEC	0.263	1.45E-05	AEBP1	0.563	1.02E-04	STAB1	-0.419	1.04E-03	CYP3A7	-0.334	2.38E-02
FAM221A	-0.364	1.60E-10	SPSB2	0.373	3.35E-08	CFD	-0.571	1.10E-06	PFN2	-0.418	1.46E-05	CCN2	-0.472	1.02E-04	MNDA	0.275	1.04E-03	ZFY	0.681	2.42E-02
RERE	-0.420	1.61E-10	KLHDC7A	0.447	3.40E-08	SERPINH1	0.283	1.15E-06	ERP29	0.285	1.49E-05	CDK1	0.299	1.03E-04	CD83	0.265	1.05E-03	SAA2	0.579	2.44E-02
ENO3	0.709	1.70E-10	SIM1	-0.559	3.45E-08	CCDC71L	-0.312	1.21E-06	TXNDC16	-0.286	1.50E-05	BCAT1	0.372	1.03E-04	TTYH3	0.312	1.05E-03	GSN	0.282	2.48E-02
SCAMP2	0.275	1.97E-10	PLIN3	0.276	3.45E-08	TCERG1	-0.312	1.23E-06	TCIM	-0.488	1.52E-05	ECM2	-0.274	1.03E-04	CD68	0.419	1.06E-03	LRRC55	0.319	2.52E-02
ICMT	0.282	2.13E-10	FZD4	-0.290	3.45E-08	RGS1	-0.582	1.24E-06	RCOR3	-0.279	1.54E-05	PRKAR1A	-0.274	1.03E-04	FKBP5	-0.550	1.07E-03	CHRNA4	-0.460	2.56E-02
ZMYM6	-0.334	2.27E-10	DLC1	-0.285	3.62E-08	PRAMEF10	0.584	1.25E-06	OAT	-0.601	1.55E-05	LMNA	0.263	1.03E-04	SHISA9	-0.285	1.07E-03	TM7SF2	0.271	2.58E-02
TRAPPC2	-0.335	2.29E-10	GLIS2	0.515	3.65E-08	APOL5	-0.535	1.27E-06	COL11A2	-0.343	1.56E-05	CCDC88C	-0.324	1.03E-04	CYP3A43	-0.472	1.07E-03	NYNRIN	0.380	2.58E-02
MT1E	-0.912	2.33E-10	MT1X	-0.798	3.65E-08	CITED2	-0.342	1.28E-06	GALC	-0.273	1.56E-05	ANGPT2	-0.339	1.03E-04	KLHL29	0.359	1.08E-03	ETNPPL	0.265	2.63E-02
KRT18	0.539	2.55E-10	UBD	0.464	3.69E-08	GLUD2	0.354	1.32E-06	LYG1	-0.316	1.56E-05	TMEM200B	0.313	1.03E-04	FICD	0.289	1.09E-03	COL6A2	0.281	2.63E-02
CELF1	-0.299	2.60E-10	RASL10B	-0.438	3.87E-08	MAP3K5	-0.301	1.33E-06	MPV17L2	0.329	1.58E-05	SPTBN5	0.493	1.03E-04	TEDC1	0.390	1.10E-03	DEPP1	-0.278	2.72E-02
ANXA2	0.624	2.69E-10	SLC25A32	-0.315	3.96E-08	ZNF334	-0.439	1.33E-06	EMILIN2	0.326	1.61E-05	ACOT1	0.468	1.05E-04	ADH1C	-0.290	1.11E-03	TFF2	-0.266	2.74E-02
AMFR	0.294	2.84E-10	ACOT7	0.319	4.03E-08	UGT1A6	0.640	1.39E-06	ABCA6	-0.337	1.61E-05	SAMD11	0.323	1.05E-04	GJA5	0.343	1.11E-03	HKDC1	0.460	2.78E-02
HPR	-0.613	2.85E-10	NHLRC2	-0.380	4.05E-08	GALNT14	-0.306	1.41E-06	CYP7A1	0.995	1.61E-05	VMP1	-0.266	1.05E-04	ALDH3A1	0.366	1.12E-03	C1QA	0.328	2.87E-02
NET1	-0.307	2.86E-10	PGAM5	0.305	4.12E-08	DNM1	-0.665	1.44E-06	CD37	0.291	1.62E-05	COL5A2	0.308	1.09E-04	GRHL1	-0.267	1.13E-03	NOMO3	0.272	3.05E-02
FEN1	0.376	2.96E-10	COBLL1	-0.386	4.19E-08	ZNF224	-0.352	1.44E-06	PJVK	-0.355	1.63E-05	NR2C1	-0.274	1.11E-04	SLC16A14	0.369	1.14E-03	TBL1Y	0.441	3.14E-02
SYT7	0.726	2.98E-10	TPI1	0.301	4.19E-08	PEPD	0.329	1.44E-06	PCSK7	0.264	1.65E-05	TKT	0.291	1.15E-04	CHCHD10	-0.298	1.16E-03	KDM5D	0.894	3.19E-02
SLC20A1	-0.443	2.98E-10	SGCB	0.444	4.20E-08	DCDC1	-0.511	1.48E-06	LARP4	-0.308	1.65E-05	CCDC38	-0.263	1.15E-04	TMEM154	0.520	1.16E-03	IFI6	0.376	3.31E-02
CXCL10	0.978	3.28E-10	ZMYM1	-0.329	4.37E-08	SPRED1	-0.357	1.48E-06	DPPA4	-0.271	1.71E-05	LRFN5	-0.545	1.16E-04	ISG15	0.425	1.16E-03	CYP1A2	-0.354	3.40E-02
ANGPTL2	0.483	3.45E-10	VAT1	0.275	4.46E-08	ABCB8	0.328	1.52E-06	BSG	0.316	1.72E-05	PTGS1	0.264	1.17E-04	ADCYAP1R1	-0.400	1.17E-03	PLIN5	-0.267	3.40E-02
CLEC1A	-0.373	3.52E-10	TAP1	0.328	4.58E-08	DOK7	0.349	1.54E-06	WDFY3	-0.306	1.73E-05	FBXO44	0.273	1.18E-04	SLC35A3	-0.267	1.19E-03	PTCHD4	0.272	3.68E-02
NOL9	-0.341	3.54E-10	CHID1	0.372	4.86E-08	CFAP221	0.450	1.55E-06	SLC10A5	-0.381	1.73E-05	HLA-DPB1	0.357	1.18E-04	GK	-0.303	1.21E-03	UTY	0.824	3.71E-02
LAMB2	0.466	3.66E-10	CD276	0.353	4.93E-08	RRM2	0.468	1.55E-06	LMAN2	0.280	1.75E-05	INHBC	0.276	1.19E-04	SLC16A1	-0.424	1.22E-03	MMP7	0.266	3.71E-02
MARCHF6	-0.340	3.72E-10	ATL2	-0.291	5.06E-08	NR2F1	-0.310	1.56E-06	SLC13A3	0.463	1.77E-05	KCNK1	-0.304	1.19E-04	FEZ1	0.279	1.22E-03	SCN7A	-0.279	3.74E-02
FMR1	-0.441	3.86E-10	ETV5	-0.409	5.08E-08	IFI35	0.321	1.56E-06	TOB1	-0.331	1.77E-05	KLF15	-0.269	1.20E-04	CLEC4G	-0.354	1.22E-03	FGF14	-0.290	3.79E-02
RBM47	-0.327	4.26E-10	C17orf67	-0.409	5.37E-08	CNGA1	-0.420	1.59E-06	ACHE	0.471	1.81E-05	HLF	-0.297	1.20E-04	MAFF	-0.288	1.23E-03	ITGAD	-0.303	3.96E-02
ZNF385B	-0.420	4.33E-10	SIRT1	-0.360	5.47E-08	CALHM2	0.285	1.61E-06	GPRC5B	0.377	1.82E-05	LIPF	-0.281	1.22E-04	SLC2A13	-0.304	1.24E-03	IP6K3	0.350	4.00E-02
ACVR1C	-0.486	4.42E-10	PPP1R1C	-0.398	5.51E-08	DNMT1	0.299	1.67E-06	TLR7	0.309	1.83E-05	TAF13	0.277	1.22E-04	ENHO	0.314	1.25E-03	DHCR7	0.270	4.06E-02
HSPA8	0.552	4.47E-10	RTN1	0.330	5.54E-08	ETAA1	-0.282	1.70E-06	ABCG2	-0.374	1.83E-05	EDA	-0.267	1.22E-04	JUND	-0.355	1.25E-03	EPCAM	0.272	4.09E-02
WSB2	0.430	4.47E-10	CDK5RAP2	0.273	5.68E-08	ATP8A1	-0.361	1.71E-06	GMNC	-0.324	1.84E-05	RAP2C	-0.270	1.28E-04	ASS1	-0.342	1.25E-03	NPBWR1	0.397	4.12E-02
C9orf72	-0.471	4.96E-10	H4C8	0.288	5.75E-08	TMX3	-0.428	1.71E-06	PHLDA1	-0.475	1.87E-05	S100A4	0.338	1.28E-04	ETFB	0.287	1.26E-03	A2M	0.369	4.23E-02
MAK16	-0.265	4.96E-10	CAPNS1	0.304	5.98E-08	LTB	0.386	1.72E-06	MRPS18C	0.279	1.87E-05	IAPP	-0.274	1.29E-04	TREH	0.450	1.27E-03	TMEM92	0.300	4.31E-02
DAXX	0.288	5.05E-10	DNAJC14	0.299	6.06E-08	TENM3	-0.465	1.73E-06	DCDC2	0.522	1.87E-05	MT1B	-0.427	1.30E-04	SPINT1	0.320	1.27E-03	CYP1A1	-0.418	4.38E-02
KDM4A	0.312	5.06E-10	DUSP8	0.496	6.13E-08	SOCS1	-0.287	1.76E-06	FMO1	0.578	1.89E-05	CDC6	0.351	1.30E-04	TRPM8	0.266	1.29E-03	DDX3Y	0.898	4.46E-02
FZD5	-0.307	5.16E-10	DCUN1D4	-0.398	6.30E-08	COPZ1	0.288	1.78E-06	IL34	0.284	1.89E-05	CHKA	-0.347	1.31E-04	CFHR5	-0.287	1.30E-03	RPS4Y1	1.098	4.64E-02
P4HB	0.369	5.35E-10	BBC3	0.385	6.32E-08	WIPI1	0.285	1.80E-06	ZNF771	-0.332	1.89E-05	CYP3A4	-0.512	1.31E-04	USP2	-0.303	1.33E-03	GCK	0.389	4.65E-02
FAM83G	0.604	5.37E-10	NFIA	-0.339	6.46E-08	RTN4RL1	0.342	1.80E-06	ABR	0.289	1.89E-05	CYP7B1	-0.324	1.34E-04	COL5A1	0.347	1.33E-03	SORT1	0.420	4.86E-02
WDR75	-0.312	5.50E-10	MAPK3	0.281	6.60E-08	GPAA1	0.354	1.84E-06	HLA-A	0.402	1.91E-05	MAST1	-0.272	1.36E-04	ADGRG7	-0.308	1.36E-03	PGGHG	0.390	4.87E-02
ANXA6	0.495	5.69E-10	POU2F1	-0.280	6.76E-08	SEC14L4	0.550	1.84E-06	TNRC6C	-0.289	1.97E-05	SRD5A2	0.413	1.37E-04	GRAMD4	-0.281	1.36E-03			
USP31	-0.354	5.96E-10	TCF19	0.396	6.89E-08	MORC3	-0.318	1.84E-06	COL4A2	0.467	1.98E-05	MANF	0.461	1.37E-04	LTB4R	-0.282	1.37E-03			
SCN9A	-0.606	5.98E-10	EPHA7	-0.364	6.91E-08	SLC38A2	-0.518	1.89E-06	CSTB	0.340	1.98E-05	MCM6	0.324	1.37E-04	RANBP17	-0.292	1.39E-03			
TGFBI	0.433	6.01E-10	UNC79	-0.338	7.05E-08	CROT	-0.297	1.89E-06	KCNK3	-0.296	1.98E-05	YPEL3	-0.317	1.40E-04	CD38	-0.264	1.40E-03			

Supplementary table 4: Differentially expressed genes of NASH vs Control

Name	Log2FC	FDR	Name	Log2FC	FDR	Name	Log2FC	FDR	Name	Log2FC	FDR	Name	Log2FC	FDR	Name	Log2FC	FDR	Name	Log2FC	FDR	Name	Log2FC	FDR
TREM2	1.292	4.27E-43	PCSK5	0.542	1.03E-15	S100A4	0.532	3.23E-12	MANF	0.647	6.41E-10	GPC5	-0.395	1.82E-08	SPATA18	0.447	4.11E-07	PLIN4	0.502	5.47E-06	SGCD	0.309	1.40E-04
AJUBA	1.033	4.32E-41	USP5	0.339	1.08E-15	DEPDC7	-0.457	3.23E-12	ZNF771	-0.417	6.49E-10	SPINT1	0.480	1.84E-08	SP4	-0.322	4.13E-07	SLCO1B3	-0.653	5.53E-06	BST2	0.373	1.42E-04
COL3A1	1.187	2.40E-37	TMEM63A	0.500	1.08E-15	SNTB1	-0.378	3.29E-12	FBLN5	0.637	6.53E-10	SLC15A2	-0.308	1.86E-08	BTBD8	-0.275	4.15E-07	ATP13A3	-0.388	5.55E-06	PMEPA1	0.298	1.44E-04
CAPG	1.092	2.40E-37	COL14A1	0.763	1.11E-15	CDC20	0.409	3.34E-12	SOX18	-0.424	6.70E-10	GALNT14	-0.311	1.86E-08	CENPE	0.320	4.15E-07	PIK3C2G	-0.290	5.56E-06	VXN	-0.409	1.45E-04
TP53I3	1.180	4.78E-37	TDRP	-0.471	1.20E-15	IDNK	-0.382	3.35E-12	B3GAT1	-0.935	6.75E-10	MAP10	-0.269	1.87E-08	EPHA7	-0.299	4.16E-07	TINAGL1	-0.362	5.57E-06	PER1	-0.399	1.45E-04
IL32	1.771	2.18E-36	ELOVL1	0.353	1.30E-15	LTB	0.489	3.39E-12	RAC3	0.422	6.85E-10	NRXN1	-0.443	1.89E-08	COL25A1	-0.372	4.19E-07	CYP1A1	-0.786	5.58E-06	SPON2	0.347	1.55E-04
SLC12A4	0.632	6.71E-35	IGF1	-0.985	1.32E-15	PPP1R1A	-0.684	3.61E-12	FMR1	-0.380	7.06E-10	TSPAN13	-0.715	1.93E-08	NFKBIA	-0.306	4.22E-07	ELF3	0.443	5.68E-06	ADGRL4	-0.350	1.56E-04
LPL	1.407	1.73E-33	CLXN	-0.441	1.35E-15	HLA-DOA	0.564	3.62E-12	HELLS	0.437	7.23E-10	TRPV2	0.317	1.98E-08	ST3GAL1	-0.347	4.25E-07	FBLN2	0.380	5.90E-06	CYP4F12	-0.320	1.57E-04
MRAS	0.728	3.98E-33	HSPA8	0.621	1.44E-15	ABCA1	-0.601	3.64E-12	GREM2	-0.369	7.37E-10	ZC3H6	-0.372	2.03E-08	CFAP251	-0.267	4.25E-07	RBP5	-0.271	5.95E-06	TDO2	-0.322	1.60E-04
FCAMR	1.370	4.94E-33	SLX4IP	-0.363	1.47E-15	CD83	0.483	3.67E-12	INSR	-0.282	7.40E-10	NACC1	0.313	2.04E-08	LIMK1	0.279	4.27E-07	HBA2	0.668	5.95E-06	PLP1	-0.316	1.61E-04
TOR1AIP2	-0.578	3.50E-32	PSMD2	0.357	1.49E-15	C1GALT1	-0.410	3.70E-12	BASP1	-0.463	7.40E-10	ENTPD1	0.280	2.08E-08	LRRC39	-0.285	4.29E-07	RIMBP2	-0.468	6.02E-06	MERTK	-0.288	1.62E-04
FABP4	1.719	5.95E-32	MCM4	0.472	1.64E-15	SKP2	-0.329	4.05E-12	CAMK1D	0.365	7.46E-10	PEG3	-0.357	2.12E-08	PLPPR1	-0.368	4.30E-07	ARRDC4	0.402	6.05E-06	PROZ	-0.364	1.67E-04
GNA12	0.520	5.95E-32	SVEP1	0.654	1.66E-15	SRD5A1	-0.416	4.07E-12	DAPK1	-0.324	7.74E-10	SLC39A3	0.302	2.12E-08	CCDC107	0.278	4.30E-07	WDR86	0.287	6.07E-06	ETV1	-0.286	1.74E-04
PTPA	0.605	6.54E-32	SLC16A10	-0.579	1.71E-15	COL10A1	0.552	4.18E-12	TUBA1C	0.387	7.75E-10	C1QTNF3	-0.351	2.14E-08	SMTN	-0.369	4.30E-07	CCDC146	0.299	6.11E-06	DPP4	0.287	1.75E-04
MACROH2A2	0.683	7.10E-32	RACGAP1	0.369	1.73E-15	GLYAT	-0.384	4.40E-12	CREM	-0.334	7.85E-10	NAPEPLD	-0.353	2.22E-08	PRIMA1	-0.296	4.30E-07	YPEL2	-0.305	6.12E-06	SCGN	0.358	1.78E-04
DTNA	1.418	7.10E-32	RP1	-0.500	1.77E-15	UGT3A2	0.464	4.41E-12	CNGA1	-0.469	7.91E-10	SLC17A5	0.310	2.23E-08	FOXO3	-0.284	4.35E-07	PON3	-0.326	6.20E-06	RNF138	-0.265	1.81E-04
CPEB3	-0.889	1.77E-31	TNR	-0.597	2.10E-15	GPAT3	-0.573	4.50E-12	COL4A1	0.613	7.94E-10	ATP1B2	-0.334	2.26E-08	LIF	0.322	4.40E-07	BIRC6	-0.330	6.26E-06	ZDHHC11	-0.383	1.83E-04
RFX5	0.563	2.49E-31	ATG4A	0.318	2.18E-15	SPA17	0.342	4.66E-12	APOL5	-0.592	8.00E-10	LMNA	0.328	2.29E-08	TXNDC16	-0.290	4.42E-07	CCDC88C	-0.328	6.27E-06	ADGRV1	-0.271	1.87E-04
DNAJC12	-0.818	1.56E-30	CELF1	-0.329	2.34E-15	ZNF275	-0.354	4.68E-12	C9orf152	0.478	8.00E-10	SH2B3	0.311	2.30E-08	CDH17	-0.285	4.48E-07	VEGFB	-0.295	6.29E-06	FNDC1	0.302	1.87E-04
COL1A2	1.221	2.37E-30	SMOC1	-0.480	2.44E-15	WDR59	-0.347	4.75E-12	PLPP4	0.329	8.04E-10	NICN1	0.268	2.30E-08	FGD4	-0.377	4.54E-07	SMIM26	-0.271	6.31E-06	UBXN10	0.399	1.89E-04
KRT8	0.892	4.62E-30	KLHDC7A	0.563	2.57E-15	WDR75	-0.304	4.88E-12	PSMC1	0.378	8.13E-10	ALDH7A1	-0.263	2.30E-08	TRAF3IP3	0.273	4.55E-07	PNRC1	-0.272	6.38E-06	CHKA	-0.296	1.93E-04
SAE1	0.440	6.62E-30	SMYD2	0.324	2.68E-15	GADD45G	-0.875	5.00E-12	HIVEP1	-0.428	8.33E-10	HLA-DMB	0.461	2.37E-08	LY6E	0.438	4.55E-07	ITM2B	-0.280	6.38E-06	HPSE	0.265	1.93E-04
ANXA2	1.004	1.38E-29	FAM221A	-0.396	2.72E-15	MRC2	0.533	5.15E-12	BRIP1	-0.382	8.38E-10	RAB40B	-0.281	2.43E-08	ADAMTS2	0.331	4.63E-07	S1PR2	0.306	6.44E-06	SRD5A2	0.353	1.95E-04
ST3GAL6	-0.755	6.85E-29	HOGA1	-0.495	2.81E-15	CCDC38	-0.408	5.21E-12	SEMA6A	-0.398	8.41E-10	IL27RA	0.305	2.52E-08	LAP3	0.270	4.72E-07	IGFBP6	0.319	6.59E-06	GAMT	-0.297	1.96E-04
MYO16	-0.661	1.26E-28	PAIP2B	-0.435	2.84E-15	AGTR1	-0.401	5.51E-12	RTN4RL1	0.384	8.43E-10	PCMTD2	-0.273	2.54E-08	LDHB	0.306	4.74E-07	MROH2B	-0.317	6.72E-06	CSRNP1	-0.312	1.96E-04
RAB7B	0.682	1.39E-28	KLHDC1	-0.482	2.90E-15	CYB561	0.368	5.52E-12	SLC38A7	0.291	8.68E-10	SMLR1	-0.336	2.58E-08	MYO1B	-0.287	4.77E-07	EPCAM	0.501	6.76E-06	TMPRSS9	0.395	1.96E-04
FHIP1A	-0.674	1.48E-28	GCDH	-0.535	2.98E-15	GPT2	-0.548	5.62E-12	BLK	-0.330	8.72E-10	ARSJ	0.318	2.59E-08	DOK6	-0.533	4.94E-07	RHOQ	0.272	6.76E-06	SLC25A10	0.272	1.96E-04
NIBAN2	0.815	2.34E-28	ALDH18A1	0.474	3.01E-15	NUSAP1	0.456	5.71E-12	PCOLCE2	-0.533	8.76E-10	NCAPG	0.348	2.59E-08	IKZF5	-0.300	4.95E-07	MAMLD1	-0.277	6.79E-06	C6orf141	-0.353	2.01E-04
CEBPD	-1.125	2.45E-28	PELI1	-0.404	3.44E-15	TMED3	0.438	5.82E-12	TMEM104	0.333	8.89E-10	ZDHHC19	-0.364	2.62E-08	IER2	-0.400	4.96E-07	EVI2A	0.336	6.92E-06	GADD45GIP1	-0.266	2.02E-04
ING1	-0.462	2.96E-28	GRB10	-0.317	3.46E-15	SCN11A	-0.354	6.16E-12	TM4SF4	0.342	9.21E-10	GPC4	0.396	2.63E-08	C7orf50	0.363	4.97E-07	MAT2B	-0.268	7.00E-06	FAM169A	0.278	2.08E-04
KCNJ5	0.739	7.22E-28	GPATCH4	0.385	3.52E-15	DCBLD2	-0.329	6.42E-12	CPZ	0.410	9.28E-10	RRAD	0.382	2.65E-08	CNTLN	-0.316	4.97E-07	CTTNBP2	-0.277	7.02E-06	CTH	-0.297	2.09E-04
MXI1	-0.549	1.06E-27	MPZL1	0.362	3.55E-15	HEATR5B	-0.405	6.42E-12	SPINT2	0.537	9.76E-10	CXADR	-0.433	2.68E-08	WIPF3	0.331	5.13E-07	SERPINA5	0.339	7.02E-06	NRP2	-0.333	2.12E-04
CDNF	-0.535	1.16E-27	CTSD	0.642	3.82E-15	WEE1	-0.378	6.81E-12	ADPRM	-0.291	9.78E-10	LATS1	-0.281	2.69E-08	RBM33	-0.280	5.17E-07	EDA2R	0.380	7.06E-06	H1-10	-0.286	2.18E-04
HSBP1L1	-0.716	1.36E-27	BECN1	0.436	3.86E-15	TMEM129	0.373	6.87E-12	DCTPP1	0.316	9.87E-10	ACKR1	0.356	2.73E-08	GLIPR2	0.278	5.24E-07	CD38	-0.320	7.23E-06	SLC38A4	-0.385	2.19E-04
CENPV	-0.711	6.98E-27	NOL9	-0.375	3.88E-15	SLC16A13	0.585	6.90E-12	CCT6B	-0.321	1.00E-09	POGLUT2	0.283	2.73E-08	SPEN	-0.334	5.24E-07	ADAMTS16	0.335	7.23E-06	OXER1	-0.388	2.20E-04
IGSF3	0.759	1.02E-26	CHST9	0.894	4.25E-15	CDHR2	0.759	7.16E-12	AGL	-0.492	1.00E-09	VWA8	-0.300	2.73E-08	KLF12	-0.363	5.25E-07	SAC3D1	0.288	7.40E-06	TMEM232	-0.273	2.25E-04
SLC39A7	0.510	1.74E-26	FERMT2	-0.426	4.44E-15	DERL3	0.427	7.40E-12	FOSB	-0.774	1.00E-09	KLF9	-0.443	2.74E-08	CYP7B1	-0.370	5.26E-07	TCAP	0.524	7.46E-06	FAM107A	-0.275	2.28E-04
PTGR2	-0.423	2.16E-26	HERC5	-0.591	4.76E-15	IL4I1	0.393	7.66E-12	PCSK2	-0.330	1.01E-09	HKDC1	0.970	2.80E-08	ATP8B1	0.342	5.27E-07	CCDC8	0.264	7.50E-06	LRFN5	-0.457	2.30E-04
ENPP1	-0.551	2.51E-26	MT1X	-0.998	4.78E-15	PPP1R1C	-0.439	7.69E-12	SLC11A2	-0.297	1.03E-09	LACC1	0.324	2.81E-08	RNF217	-0.329	5.32E-07	TRIM31	0.398	7.52E-06	CDH19	-0.281	2.32E-04
TSPAN17	0.462	3.00E-26	YKT6	0.304	4.85E-15	SIAH1	-0.392	7.81E-12	MAT1A	-0.307	1.05E-09	GRIK2	-0.315	2.81E-08	PDIA6	0.479	5.54E-07	MAB21L2	0.271	7.77E-06	GGT1	0.303	2.33E-04
NAAA	-0.510	3.04E-26	DPP3	0.373	4.92E-15	MTHFD1L	0.465	7.91E-12	ERGIC1	0.291	1.09E-09	POM121	-0.283	2.81E-08	PCDHGA9	-0.270	5.62E-07	BMF	0.319	7.80E-06	ADSS1	0.273	2.34E-04
DUSP8	0.866	3.60E-26	REPS1	-0.440	5.00E-15	EYA4	-0.402	7.98E-12	IKBKG	0.355	1.09E-09	IGFBP1	-1.418	2.82E-08	CD109	0.391	5.63E-07	CYGB	0.324	7.81E-06	SAA2	0.800	2.41E-04
TAGLN2	0.652	4.64E-26	AEBP1	0.984	5.05E-15	LAMC3	0.789	8.81E-12	MEIS2	-0.268	1.10E-09	KDM7A	-0.292	2.86E-08	CFTR	0.611	5.63E-07	SPTBN2	-0.349	7.90E-06	LTB4R	-0.281	2.42E-04
APOL3	1.033	5.46E-26	AFF1	-0.426	5.21E-15	HPS6	0.286	8.82E-12	RAB32	0.351	1.11E-09	PHF19	0.269	2.88E-08	PEPD	0.299	5.65E-07	ARPP21	0.317	7.93E-06	FCGR2A	0.271	2.43E-04
FRRS1	-0.568	9.59E-26	WDPCP	0.589	5.71E-15	ECT2	0.415	8.82E-12	IGFBP2	-1.185	1.13E-09	STK31	-0.295	2.88E-08	RNF187	0.276	5.72E-07	PINK1	0.329	8.05E-06	EPO	-0.325	2.45E-04
TIAM1	-0.568	9.94E-26	CYP2C8	-0.513	6.51E-15	AQP3	-0.399	9.13E-12	ANXA1	0.459	1.20E-09	ASB9	-0.520	2.89E-08	TXNDC9	0.287	5.76E-07	FHL2	0.411	8.05E-06	CDS1	-0.264	2.47E-04
SPP1	1.607	1.00E-25	CD276	0.442	7.24E-15	ARPC2	0.279	9.21E-12	IFI27L1	0.324	1.24E-09	CMC2	-0.353	2.90E-08	CEBPA	0.377	5.77E-07	ODC1	0.264	8.42E-06	ICAM3	-0.291	2.49E-04
CXCL10	1.458	1.01E-25	PGAM5	0.380	7.33E-15	LEAP2	0.521	9.49E-12	GPC1	0.434	1.25E-09	RGS3	0.269	2.95E-08	PDE2A	-0.370	5.78E-07	ARPC1B	0.311	8.42E-06	FDFT1	0.398	2.53E-04
P4HA2	0.542	2.02E-25	MARF1	-0.291	7.57E-15	IMPA1	-0.492	9.50E-12	ICOSLG	0.366	1.28E-09	PDK4	-0.539	2.97E-08	WNK3	-0.445	5.84E-07	PRRT1B	-0.278	8.48E-06	TSSC4	0.290	2.62E-04
CALM1	-0.419	2.15E-25	TGFBI	0.477	8.17E-15	CFL1	0.366	9.56E-12	SPTAN1	0.358	1.30E-09	NBPF15	0.452	2.98E-08	SEC14L4	0.503	5.89E-07	SLC13A3	0.419	8.48E-06	CCL5	0.306	2.62E-04
COL1A1	1.398	2.39E-25	SIRT5	-0.314	8.93E-15	USB1	0.278	9.58E-12	GP5	-0.284	1.31E-09	FNIP1	-0.370	3.05E-08	RBM39	-0.268	5.90E-07	SASH3	0.283	8.71E-06	HPS5	0.337	2.64E-04

LASP1	0.450	3.48E-25	ANKRD13C	-0.291	9.28E-15	SS18L1	-0.346	1.00E-11	SHROOM2	-0.325	1.32E-09	ABCB8	0.330	3.09E-08	LRP6	-0.347	5.91E-07	SPX	-0.268	8.87E-06	KCNAB2	0.368	2.65E-04
CD151	0.721	5.45E-25	FNIP2	-0.722	9.28E-15	DIRAS3	-0.497	1.01E-11	ZNF334	-0.480	1.36E-09	PTCH1	-0.296	3.09E-08	PLCB1	-0.318	5.99E-07	UBN2	-0.272	8.98E-06	FLT1	-0.317	2.65E-04
APOF	-0.794	9.09E-25	PDE8A	-0.273	9.63E-15	PLD6	-0.366	1.01E-11	STPG2	-0.287	1.38E-09	RNASEH2A	0.285	3.09E-08	BLMH	-0.301	6.07E-07	DDB2	0.321	8.98E-06	CYP3A5	-0.459	2.65E-04
PRKAG2	-0.501	2.48E-24	MCCC2	-0.350	9.69E-15	KDM2B	-0.315	1.03E-11	PHC3	-0.381	1.40E-09	SLC20A1	-0.341	3.13E-08	SLC1A3	0.434	6.07E-07	RIC3	-0.346	9.07E-06	EMID1	0.267	2.66E-04
HPR	-0.883	2.50E-24	IFT88	-0.353	1.02E-14	TCEA3	-0.361	1.10E-11	ALDH2	-0.313	1.42E-09	SPTBN5	0.609	3.13E-08	PPM1E	-0.362	6.10E-07	TMEM141	0.291	9.10E-06	BBLN	0.318	2.68E-04
WWC1	0.585	2.99E-24	TMEM41B	-0.408	1.02E-14	TENM3	-0.576	1.11E-11	ZSWIM9	0.295	1.42E-09	IRF2BP2	-0.290	3.21E-08	SLCO4C1	-0.537	6.36E-07	CD6	0.299	9.14E-06	IFRD1	-0.275	2.69E-04
HS3ST2	0.761	3.17E-24	ISM1	0.694	1.07E-14	ANKRD29	0.522	1.11E-11	ORAI3	-0.310	1.43E-09	PLA2G7	0.460	3.24E-08	FZD1	0.273	6.40E-07	GZMK	0.323	9.17E-06	MREG	-0.295	2.73E-04
CIDEC	0.743	3.49E-24	RREB1	-0.451	1.11E-14	NT5E	-0.473	1.11E-11	OLFML3	0.378	1.44E-09	ETNK2	-0.406	3.29E-08	WDR19	-0.284	6.59E-07	VLDLR	0.355	9.23E-06	PRELP	0.297	2.74E-04
LGALS3	0.869	4.76E-24	PARD6B	-0.483	1.14E-14	WDFY3	-0.420	1.11E-11	P4HB	0.315	1.48E-09	MTMR11	0.382	3.32E-08	RCOR3	-0.280	6.59E-07	PTGS1	0.264	9.34E-06	MUC5B	-0.408	2.75E-04
MMP14	0.652	5.18E-24	OLR1	0.554	1.14E-14	CFD	-0.695	1.11E-11	IRAK1	0.315	1.52E-09	RICTOR	-0.417	3.34E-08	DEFB1	0.470	6.60E-07	SNRPG	-0.264	9.35E-06	CD53	0.289	2.81E-04
DYRK1A	-0.373	5.52E-24	CYP2B6	-0.837	1.14E-14	PAQR7	0.333	1.12E-11	KIRREL1	0.401	1.56E-09	CPLX1	-0.327	3.37E-08	SMIM19	-0.270	6.68E-07	ARRDC3	-0.465	9.40E-06	DENND4C	-0.270	3.07E-04
ERN1	-0.676	7.37E-24	XPNPEP3	-0.327	1.14E-14	DMTN	0.523	1.12E-11	KIAA1522	0.356	1.59E-09	ZNF563	0.285	3.41E-08	C17orf67	-0.328	6.74E-07	VEGFA	-0.305	9.48E-06	LRRK2	-0.326	3.08E-04
OR2I1P	1.467	7.54E-24	KANSL1L	-0.367	1.18E-14	FGFRL1	-0.452	1.14E-11	TMED9	0.299	1.59E-09	MCM5	0.414	3.44E-08	VPS13A	-0.357	6.76E-07	AGPAT5	-0.383	9.57E-06	NDUFB1	-0.293	3.16E-04
PELI2	-0.710	8.00E-24	ZNF280C	-0.358	1.23E-14	CCNB1	0.355	1.17E-11	ST13	-0.316	1.60E-09	HEYL	0.409	3.54E-08	ACO2	0.367	6.85E-07	ANPEP	-0.268	9.67E-06	UPF3B	0.274	3.16E-04
PDGFA	0.819	1.26E-23	CENPC	-0.394	1.24E-14	ISLR	0.501	1.17E-11	AP1AR	-0.352	1.62E-09	ZSWIM5	-0.322	3.59E-08	SLC10A5	-0.383	6.85E-07	FGB	-0.320	9.97E-06	COL6A2	0.385	3.18E-04
PHLDA3	0.867	1.26E-23	TTC6	-0.552	1.27E-14	PRKCE	0.865	1.17E-11	ST6GALNAC	0.285	1.62E-09	EBF4	-0.354	3.65E-08	PATJ	-0.279	6.88E-07	AUTS2	-0.273	1.01E-05	HLA-DRB1	0.443	3.19E-04
LIX1L	-0.406	1.32E-23	ADCK2	0.376	1.42E-14	CLEC1A	-0.353	1.19E-11	PER3	-0.574	1.63E-09	ACOX2	-0.274	3.67E-08	ECM2	-0.304	6.91E-07	SYNE1	-0.264	1.01E-05	PDCD1LG2	-0.304	3.23E-04
MSN	0.672	1.57E-23	HAAO	-0.522	1.43E-14	GINS1	0.368	1.20E-11	CKAP2	0.382	1.64E-09	MROH7	-0.356	3.69E-08	MBLAC2	-0.285	7.00E-07	ADCY1	-0.358	1.02E-05	C5AR2	-0.269	3.24E-04
ITSN1	-0.378	1.61E-23	CADM2	-0.497	1.43E-14	IGFBPL1	0.483	1.20E-11	HEBP2	-0.347	1.67E-09	PKD1L1	-0.365	3.69E-08	CYP3A4	-0.577	7.06E-07	HUNK	0.272	1.05E-05	SORCS2	0.430	3.24E-04
PYGB	0.515	3.09E-23	EPB41L5	-0.369	1.46E-14	ALDH3A1	0.653	1.20E-11	PPP1R9B	0.327	1.67E-09	AMT	-0.269	3.70E-08	RNF157	0.311	7.11E-07	EPHB1	-0.361	1.06E-05	MAN1A1	-0.286	3.26E-04
C11orf71	-0.461	4.54E-23	RDH12	-0.586	1.72E-14	RBM47	-0.310	1.22E-11	VAMP1	0.381	1.69E-09	S100A10	0.396	3.72E-08	ZKSCAN1	-0.319	7.25E-07	PMM1	-0.293	1.08E-05	ANGPTL8	0.452	3.47E-04
IVNS1ABP	-0.470	1.08E-22	UNC119	0.460	1.79E-14	SOD1	-0.395	1.32E-11	SLC16A1	-0.678	1.69E-09	SNORC	0.499	3.95E-08	PRSS23	0.351	7.37E-07	SEPSECS	-0.294	1.10E-05	LTK	-0.284	3.54E-04
SQSTM1	0.509	1.08E-22	MTHFD2L	-0.619	1.84E-14	AKAP8	-0.315	1.33E-11	CLEC11A	0.392	1.70E-09	TMTC1	-0.550	3.96E-08	GAS7	0.329	7.38E-07	TMEM92	0.545	1.10E-05	CCDC57	0.310	3.54E-04
ZFP1	-0.663	1.26E-22	CYP4F22	0.669	1.84E-14	ATG7	0.278	1.35E-11	ACLY	0.418	1.72E-09	SLAMF7	0.407	3.97E-08	TTLL7	-0.360	7.48E-07	PPP1R3B	0.356	1.11E-05	NR4A1	-0.317	3.57E-04
OPTN	0.446	1.26E-22	SMPDL3A	-0.507	1.84E-14	ASB4	-0.423	1.36E-11	H2AZ1	0.330	1.72E-09	FMO5	-0.410	4.06E-08	CARS1	0.272	7.48E-07	VSTM2L	0.296	1.12E-05	SULT1C2	0.357	3.61E-04
CD52	0.834	1.28E-22	PBLD	-0.407	1.89E-14	ADCY10	-0.493	1.36E-11	BTN3A3	0.290	1.75E-09	LARP4	-0.341	4.06E-08	MMP15	0.333	7.51E-07	C2CD4A	0.269	1.14E-05	NEK10	-0.549	3.61E-04
KPNA2	0.649	1.73E-22	CNST	-0.373	1.96E-14	LURAP1L	-0.487	1.48E-11	CLIC1	0.321	1.77E-09	ADIRF	0.439	4.06E-08	AKAP11	-0.345	7.54E-07	HGD	-0.286	1.15E-05	NAGS	-0.263	3.64E-04
DUSP10	-0.645	1.73E-22	NAT1	-0.396	2.06E-14	ZNF460	-0.377	1.50E-11	ZNF236	-0.302	1.78E-09	ID2	-0.359	4.11E-08	PRR15L	0.351	7.59E-07	SHFL	-0.270	1.15E-05	LCP1	0.306	3.71E-04
DNAJC19	-0.515	1.88E-22	F11	-0.428	2.07E-14	TRIB1	-0.460	1.51E-11	TBC1D2	-0.396	1.79E-09	ZFP36L1	-0.282	4.14E-08	ELOVL2	0.489	7.64E-07	TMSB4X	0.291	1.16E-05	BMP6	-0.266	3.79E-04
IDS	-0.395	1.88E-22	ZNF468	0.525	2.11E-14	PBK	0.361	1.55E-11	PPARGC1A	-0.691	1.79E-09	GMPPB	0.394	4.17E-08	PANX2	-0.452	7.64E-07	MFAP4	0.481	1.20E-05	HSPA4L	0.315	3.85E-04
NET1	-0.421	1.92E-22	FMOD	0.563	2.15E-14	LBR	-0.414	1.55E-11	INPP1	-0.273	1.80E-09	SLC7A2	-0.480	4.23E-08	SOAT2	-0.382	7.69E-07	CYP11A1	0.281	1.21E-05	NNMT	-0.591	3.87E-04
ATP6V0D1	0.542	1.92E-22	SNAP25	0.603	2.35E-14	VPS18	0.304	1.63E-11	PCDHGB6	-0.362	1.81E-09	SEZ6L	-0.275	4.25E-08	PAH	-0.288	7.69E-07	TRPV4	0.322	1.22E-05	FGG	-0.299	3.91E-04
CHRAC1	-0.407	2.04E-22	CAPN2	0.508	2.39E-14	MAGI3	-0.329	1.63E-11	FIBP	0.291	1.87E-09	DEPDC1	0.332	4.65E-08	PSMA7	0.267	7.79E-07	LCAT	-0.293	1.23E-05	OAS3	0.341	3.95E-04
FAT1	1.018	2.31E-22	LGALS3BP	0.693	2.40E-14	ZC3H4	-0.334	1.66E-11	SERPINE2	0.402	1.89E-09	RHBDF2	0.285	4.69E-08	SLC29A1	0.281	7.88E-07	CRACD	0.301	1.24E-05	IDI1	0.455	3.95E-04
FBXO21	-0.525	2.80E-22	TSPAN5	-0.470	2.49E-14	TOR3A	0.459	1.73E-11	TUBB2A	0.486	1.91E-09	MXD4	0.359	4.71E-08	GNG5	-0.272	7.95E-07	MEI4	-0.358	1.25E-05	BBOX1	-0.297	3.98E-04
ADHFE1	-0.589	2.92E-22	TRMT10A	-0.351	2.49E-14	KCNC4	-0.377	1.77E-11	DCDC2	0.636	1.91E-09	HECW2	-0.408	4.88E-08	SLC25A33	0.389	7.98E-07	FOXN2	-0.314	1.25E-05	NCOA2	-0.270	4.00E-04
PCDH20	-0.880	2.92E-22	ATG2B	-0.427	2.60E-14	TRPC3	-0.334	1.81E-11	ARF4	0.345	1.92E-09	MT1M	-0.820	4.90E-08	COL11A2	-0.341	8.00E-07	GJA4	0.314	1.26E-05	BHMT	-0.294	4.05E-04
ACADSB	-0.785	4.53E-22	RASGEF1B	-0.474	2.63E-14	TRAF2	0.349	1.96E-11	XRN1	-0.453	1.94E-09	FICD	0.414	4.92E-08	SARDH	-0.320	8.12E-07	PGAP1	-0.274	1.26E-05	CPXM2	0.353	4.08E-04
CXXC4	-0.677	4.84E-22	SLC22A10	-1.349	2.63E-14	SPNS2	0.416	2.07E-11	KRTCAP3	-0.448	1.94E-09	TPT1	-0.347	5.01E-08	SRSF4	-0.297	8.33E-07	FGA	-0.326	1.26E-05	FKBP5	-0.516	4.15E-04
PI4K2A	0.432	5.29E-22	LTBP2	0.776	2.63E-14	SRXN1	0.343	2.21E-11	FBXO27	0.345	1.94E-09	MAD2L1	0.315	5.16E-08	ANLN	0.338	8.45E-07	PFKFB3	-0.374	1.27E-05	CLK1	-0.266	4.20E-04
SLITRK3	-1.192	5.29E-22	MTR	-0.372	2.63E-14	STK3	-0.321	2.25E-11	C1orf198	0.407	1.98E-09	SMARCA1	-0.285	5.17E-08	HIPK2	-0.313	8.62E-07	EPHA1	0.270	1.31E-05	CISH	-0.457	4.23E-04
FNDC5	1.053	5.29E-22	ASS1	-0.695	2.64E-14	GLA	0.272	2.29E-11	CREBZF	-0.424	1.99E-09	PLCG1	0.279	5.22E-08	ZNF770	-0.294	8.65E-07	PDE7B	-0.319	1.31E-05	CXCL11	0.276	4.37E-04
SOCS2	-0.973	7.84E-22	SGCB	0.541	2.77E-14	PRKAA2	0.680	2.36E-11	SCN8A	-0.390	1.99E-09	SLC6A13	-0.520	5.22E-08	SLC6A2	0.608	8.65E-07	PTH2R	-0.386	1.32E-05	SGK1	0.292	4.47E-04
CYP2C19	-1.934	8.33E-22	PDCL3	0.325	2.77E-14	PLIN3	0.293	2.38E-11	DMRTA1	-0.342	2.03E-09	LOXL1	0.423	5.35E-08	SLCO1B3-SL	-0.460	8.65E-07	STMN1	0.295	1.32E-05	CD5L	-0.595	4.51E-04
RTN1	0.516	9.96E-22	GABPB1	-0.308	2.80E-14	CDK1	0.445	2.38E-11	KCNMB4	-0.310	2.04E-09	GPX8	0.321	5.39E-08	SLC35C1	0.452	8.72E-07	RESF1	-0.269	1.35E-05	MFSD3	0.264	4.58E-04
AMDHD1	-0.860	1.22E-21	CCL20	0.718	2.85E-14	CDK5RAP2	0.294	2.41E-11	SLCO1A2	-0.845	2.04E-09	TCHH	-0.280	5.41E-08	IQGAP3	0.316	8.72E-07	ADGRE1	-0.423	1.38E-05	USP43	-0.288	4.59E-04
PER2	-0.635	1.25E-21	PHLPP1	-0.441	2.94E-14	APOBR	0.397	2.44E-11	MGLL	0.353	2.07E-09	ACADL	-0.264	5.42E-08	STAT1	0.415	8.75E-07	ADCYAP1R1	-0.462	1.39E-05	POLR2L	0.304	4.81E-04
BICDL1	0.676	1.27E-21	MAJIN	-0.405	2.94E-14	ZCCHC8	-0.270	2.52E-11	CETP	-0.813	2.09E-09	RHOB	-0.530	5.42E-08	SDC1	0.288	8.76E-07	ANKRD1	0.281	1.40E-05	ADGRA2	0.272	4.89E-04
PPM1K	-0.728	1.28E-21	AFF3	-0.531	3.05E-14	TNRC6C	-0.393	2.56E-11	LRP4	-0.420	2.12E-09	CXCL2	-0.714	5.43E-08	MMP2	0.507	8.82E-07	NAMPT	-0.419	1.43E-05	PCLO	0.303	5.11E-04
RPS6KL1	0.592	1.43E-21	PLXNC1	0.513	3.05E-14	ALG1	0.280	2.61E-11	TRAPPC2	-0.277	2.13E-09	VAMP4	-0.292	5.53E-08	TKT	0.322	8.88E-07	CYP1A2	-0.607	1.43E-05	RASD1	-0.372	5.16E-04
ASPA	-0.707	1.54E-21	UBD	0.561	3.19E-14	FOXP2	-0.412	2.63E-11	LITAF	0.367	2.15E-09	SIL1	0.287	5.56E-08	ABCC2	-0.290	8.89E-07	GABPA	-0.312	1.43E-05	LEPR	-0.436	5.34E-04
TTC9	0.873	1.96E-21	TLR7	0.476	3.21E-14	CFAP69	-0.426	2.72E-11	DNAJC5B	0.323	2.15E-09	TRPM6	-0.283	5.67E-08	SENP7	-0.327	8.94E-07	INHBB	-0.440	1.44E-05	RGS4	0.265	5.42E-04
FEN1	0.501	2.90E-21	CDH23	-0.654	3.29E-14	MRPS28	0.342	2.75E-11	RMI2	0.421	2.18E-09	CENPF	0.348	5.72E-08	NUDT4	-0.309	9.19E-07	TRDN	-0.322	1.44E-05	MRC1	-0.436	5.43E-04
MAGED2	0.400	3.23E-21	HORMAD2	-0.539	3.51E-14	NFKBIE	0.348	2.90E-11	CYP7A1	1.199	2.19E-09	DYNLL1	0.336	5.84E-08	FBXL18	0.299	9.34E-07	ADI1	-0.265	1.44E-05	CYP4X1	-0.266	5.51E-04

SLC19A2	-0.701	4.18E-21	DNAJC25	-0.379	3.52E-14	KCNAB1	-0.411	3.10E-11	SLC4A2	0.327	2.19E-09	FPR3	0.519	5.85E-08	FAM162A	-0.318	9.45E-07	IL4R	-0.279	1.45E-05	TMPRSS3	0.293	5.61E-04
DHRS7	0.758	4.23E-21	DUSP19	-0.374	3.63E-14	MARCHF6	-0.315	3.26E-11	UGT1A3	-0.664	2.22E-09	ENDOD1	0.267	5.88E-08	TSNAX	-0.270	9.53E-07	PPP2R1B	-0.314	1.46E-05	LGR4	-0.290	5.88E-04
IPO13	0.386	6.64E-21	MCM6	0.558	3.63E-14	KLKB1	-0.321	3.41E-11	IL34	0.345	2.24E-09	SALL4	-0.395	5.88E-08	DIPK1C	-0.275	9.55E-07	PADI1	0.682	1.48E-05	GJA5	0.315	6.01E-04
KRT18	0.706	8.03E-21	MYO1C	0.379	3.75E-14	RANGAP1	0.392	3.61E-11	GINS4	0.291	2.28E-09	UBR5	-0.263	5.98E-08	FERMT1	0.308	9.77E-07	CHL1	-0.493	1.49E-05	VPS13C	-0.275	6.07E-04
LAMA3	0.817	8.59E-21	TK1	0.521	3.78E-14	PODN	0.639	3.78E-11	FADS2	0.969	2.32E-09	KLHL34	0.276	6.07E-08	CPAMD8	-0.349	9.80E-07	TMEM14A	-0.291	1.49E-05	CCL19	0.506	6.08E-04
CD59	0.443	8.69E-21	CFAP221	0.619	3.93E-14	GREB1L	-0.395	3.86E-11	CCL22	0.387	2.32E-09	EPHA3	0.562	6.08E-08	CDC7	0.282	9.89E-07	PFN2	-0.365	1.49E-05	ZIC1	-0.311	6.10E-04
MAGI1	-0.401	8.69E-21	ANGEL2	-0.299	3.93E-14	RNASE1	0.599	3.89E-11	KDELR3	0.524	2.32E-09	CAPN1	0.291	6.11E-08	DSC2	-0.282	1.01E-06	LRSAM1	0.304	1.50E-05	SULT1A2	-0.283	6.16E-04
TNFRSF12A	0.945	1.02E-20	MUL1	0.346	4.06E-14	RASL10A	-0.356	3.98E-11	GNMT	-0.631	2.33E-09	SLC26A5	-0.265	6.11E-08	MDN1	-0.494	1.03E-06	RAB3B	0.384	1.52E-05	MTUS2	-0.349	6.17E-04
CDKN1A	1.025	1.13E-20	FANCL	-0.383	4.39E-14	TRIM22	0.496	4.00E-11	CCNB2	0.313	2.33E-09	NCOR1	-0.281	6.16E-08	PGA5	-0.293	1.03E-06	ABCA10	-0.419	1.52E-05	MT1G	-0.539	6.18E-04
RTCB	0.399	1.31E-20	UNC5B	0.484	4.45E-14	TAP1	0.347	4.05E-11	KCNK3	-0.359	2.33E-09	SNAI2	-0.368	6.19E-08	TRPM8	0.347	1.03E-06	CHST4	0.456	1.53E-05	FRMD7	0.265	6.33E-04
CMYA5	-0.884	1.34E-20	STX3	0.322	4.48E-14	SAMD11	0.476	4.07E-11	DNAH6	-0.515	2.35E-09	SLC34A1	-0.301	6.24E-08	ZDHHC2	0.377	1.05E-06	NCOA4	0.284	1.57E-05	SLC25A27	-0.348	6.46E-04
INHBE	1.020	1.44E-20	MKX	-0.412	4.61E-14	CHAD	-0.594	4.07E-11	KIF20A	0.374	2.35E-09	COL4A4	0.451	6.28E-08	DDX60L	-0.383	1.06E-06	DEPP1	-0.457	1.58E-05	SPRY4	-0.264	6.46E-04
CDK19	-0.397	1.44E-20	MED22	0.330	4.63E-14	MEGF10	-0.367	4.22E-11	SPTSSB	-0.339	2.37E-09	HLA-DPB1	0.435	6.32E-08	EPHB6	0.285	1.06E-06	NR2C1	-0.267	1.59E-05	CAP1	0.285	6.83E-04
SEPTIN6	0.436	1.70E-20	BBC3	0.470	4.63E-14	SLC16A9	-0.526	4.25E-11	PAN3	-0.402	2.42E-09	SPATA21	0.431	6.41E-08	LIPF	-0.310	1.06E-06	CYS1	0.296	1.61E-05	CREB3L3	-0.271	6.88E-04
MMP9	0.824	1.70E-20	LRRC20	0.424	5.07E-14	PPP2R2A	-0.288	4.49E-11	FOS	-1.074	2.42E-09	ZNRF2	-0.309	6.43E-08	TNFRSF9	0.279	1.08E-06	ORM1	-0.426	1.66E-05	THEMIS	0.309	6.94E-04
SPRYD3	0.457	2.07E-20	PDK3	-0.444	5.08E-14	GPR158	-0.371	4.51E-11	UBR2	-0.289	2.47E-09	MB	0.506	6.46E-08	DHRS2	-1.044	1.08E-06	GPC3	0.604	1.70E-05	NEXN	0.285	7.04E-04
SNX11	0.344	2.21E-20	ETV5	-0.496	5.15E-14	CDC45	0.420	4.57E-11	SLC25A32	-0.299	2.47E-09	C1R	-0.264	6.46E-08	ITIH1	-0.266	1.09E-06	ZFAT	-0.285	1.70E-05	GOLGA6B	-0.271	7.09E-04
EPPK1	0.712	2.72E-20	ENO3	0.733	5.35E-14	UNC79	-0.361	4.59E-11	MELK	0.350	2.47E-09	C3orf70	-0.264	6.53E-08	STAB2	-0.603	1.10E-06	RARRES1	0.274	1.71E-05	IDUA	-0.293	7.10E-04
ZWINT	0.652	3.22E-20	NEURL1	-0.442	5.62E-14	ITIH4	-0.396	4.66E-11	MTSS1	-0.277	2.49E-09	TSHZ2	0.325	6.57E-08	ZNF160	-0.291	1.10E-06	PLEKHF1	-0.288	1.73E-05	SLC16A4	-0.321	7.22E-04
L3MBTL3	-0.529	3.52E-20	NSUN6	-0.459	5.69E-14	MARVELD1	0.379	4.74E-11	PRLR	-0.564	2.52E-09	NIN	0.318	6.60E-08	PRKAR1A	-0.299	1.10E-06	ZNF518B	-0.265	1.78E-05	SLC25A18	-0.332	7.86E-04
FAM111B	0.895	4.07E-20	ZFAND5	-0.606	6.89E-14	YPEL3	-0.473	4.75E-11	PCK1	-0.951	2.53E-09	GRID1	0.641	6.64E-08	DNA2	-0.320	1.10E-06	RPL29	-0.270	1.80E-05	NPY1R	-0.279	8.04E-04
ANO10	0.428	4.34E-20	ARF3	0.383	7.18E-14	RTTN	-0.405	4.76E-11	PARD3B	0.274	2.56E-09	SCN2A	-0.355	6.72E-08	RC3H1	-0.277	1.11E-06	SLC5A11	-0.294	1.80E-05	DHCR7	0.373	8.05E-04
MKI67	0.763	4.42E-20	AOX1	-0.568	7.36E-14	TPP2	-0.320	5.13E-11	SPRED2	-0.330	2.56E-09	ABCB11	-0.454	6.75E-08	IL17RB	-0.411	1.12E-06	RPS15A	-0.301	1.81E-05	SSPN	0.286	8.27E-04
YWHAH	0.452	5.78E-20	WBP1L	-0.282	7.66E-14	GNE	-0.395	5.20E-11	CD2	0.433	2.57E-09	HSP90AA1	0.397	7.13E-08	IFI35	0.284	1.13E-06	RGPD2	-0.328	1.82E-05	CDH6	0.334	8.37E-04
TOP2A	0.845	5.78E-20	CKS2	0.466	9.11E-14	SATB1	-0.353	5.34E-11	KCND3	-0.354	2.59E-09	COX17	-0.342	7.24E-08	TERB2	-0.318	1.13E-06	TOMM40L	0.290	1.83E-05	KDR	-0.399	8.50E-04
HPRT1	-0.668	5.78E-20	TMCO3	0.374	9.27E-14	POLE3	0.280	5.37E-11	ALDH1B1	0.443	2.60E-09	NCF2	0.430	7.26E-08	UGT2B15	0.340	1.14E-06	SEMA6B	-0.273	1.85E-05	LPAR6	-0.275	8.87E-04
TIAL1	-0.290	6.72E-20	MCM2	0.596	9.50E-14	HTR7	0.350	5.49E-11	CCDC178	-0.274	2.63E-09	ITGB2	0.446	7.28E-08	OLFM2	0.737	1.19E-06	SCTR	0.305	1.87E-05	RWDD3	-0.314	9.00E-04
CREB3	0.409	6.85E-20	TGFBR3	-0.491	1.01E-13	DCT	-0.405	5.51E-11	LDB2	-0.430	2.68E-09	CCDC3	0.362	7.47E-08	HLA-DRA	0.496	1.21E-06	BLVRA	0.288	1.87E-05	BGN	0.303	9.06E-04
ATG13	0.297	9.05E-20	BRAF	-0.290	1.02E-13	RTN4IP1	0.274	5.73E-11	PSAP	0.309	2.73E-09	FAS	0.331	7.48E-08	TNKS1BP1	0.303	1.22E-06	DUSP6	-0.377	1.90E-05	JUNB	-0.347	9.11E-04
PRKAA1	-0.503	9.26E-20	ZSWIM6	-0.370	1.13E-13	XYLB	-0.295	5.73E-11	WDR64	-0.267	2.76E-09	EFNA1	-0.358	7.54E-08	ACOT1	0.509	1.22E-06	DOCK4	-0.269	1.92E-05	ENHO	0.281	9.11E-04
TSC22D1	0.570	1.00E-19	RELB	0.470	1.22E-13	VSIG10	0.312	5.93E-11	CHMP4A	0.308	2.92E-09	CEP95	-0.321	7.55E-08	SMG1	-0.438	1.23E-06	ARMH4	-0.304	1.92E-05	MICAL1	0.269	9.19E-04
TCF7L2	-0.444	1.09E-19	RERE	-0.427	1.22E-13	TUBG1	0.318	5.93E-11	GYS2	-0.354	2.92E-09	ECE1	-0.296	7.55E-08	PTGFR	0.401	1.25E-06	MPZL2	0.270	1.92E-05	ACE2	0.301	9.34E-04
CD24	1.147	1.14E-19	MCM3	0.343	1.24E-13	B3GNT5	0.522	6.17E-11	HECTD1	-0.350	2.92E-09	CLEC14A	0.346	7.56E-08	SEMA4G	-0.288	1.25E-06	REEP6	0.344	1.93E-05	SLC16A14	0.327	9.45E-04
SUMF2	0.408	1.23E-19	PPARA	-0.421	1.27E-13	TYMP	0.569	6.37E-11	CD37	0.348	2.92E-09	RAD51AP1	0.308	7.66E-08	WDR72	-0.375	1.26E-06	POMK	-0.279	1.93E-05	EPHA2	-0.264	9.70E-04
ZCCHC14	-0.408	1.26E-19	MR1	0.342	1.32E-13	KLF15	-0.396	6.59E-11	C1orf216	0.278	2.94E-09	MSC	0.281	7.68E-08	PRPF39	-0.299	1.27E-06	TTC36	-0.318	1.97E-05	PPP1R16A	-0.316	1.05E-03
PSEN2	0.343	2.52E-19	EEF2KMT	0.348	1.40E-13	CDKN1C	-0.382	6.59E-11	GMNC	-0.389	2.99E-09	HLA-DMA	0.374	7.93E-08	MTURN	-0.299	1.27E-06	TRIM2	-0.344	1.99E-05	VSIG2	0.264	1.06E-03
TYMS	0.606	2.72E-19	BTN2A2	0.301	1.43E-13	SCD	0.979	6.75E-11	CLEC4M	-0.685	3.02E-09	COL16A1	0.532	8.03E-08	CMC4	-0.316	1.28E-06	PRRG4	-0.303	2.00E-05	CYBRD1	0.293	1.07E-03
ANGPTL2	0.611	2.80E-19	UNC13B	0.304	1.49E-13	C9	-0.578	6.91E-11	CILP	0.341	3.02E-09	TSPAN3	0.289	8.09E-08	FITM1	-0.374	1.29E-06	SSC5D	0.300	2.10E-05	CR1	-0.395	1.07E-03
GLIS2	0.739	3.58E-19	PPP4R3A	-0.281	1.65E-13	NR0B2	-0.816	6.97E-11	SAMD5	-0.498	3.03E-09	PPIF	0.482	8.16E-08	SEMA6C	-0.501	1.32E-06	GLP2R	-0.300	2.12E-05	AGR2	-0.319	1.10E-03
FAM9B	-0.801	3.69E-19	MTUS1	-0.376	1.65E-13	FBN2	-0.452	7.00E-11	SLC46A1	-0.272	3.03E-09	SLC44A3	0.473	8.24E-08	SPHK1	0.314	1.32E-06	RHOBTB1	0.302	2.13E-05	BTNL9	-0.266	1.14E-03
DENND1C	0.375	3.79E-19	PDGFRB	0.544	1.68E-13	ZDHHC17	-0.384	7.00E-11	PMF1	-0.443	3.09E-09	PHF3	-0.277	8.25E-08	CCL23	-0.328	1.33E-06	HLA-DPA1	0.432	2.18E-05	MVD	0.515	1.15E-03
S100A14	0.682	4.30E-19	PLP2	0.475	1.80E-13	NUDT18	0.327	7.00E-11	CGREF1	0.415	3.09E-09	ZNF607	0.287	8.66E-08	VAT1L	-0.280	1.33E-06	THEM6	0.286	2.19E-05	NQO1	0.279	1.15E-03
PHF8	-0.585	4.59E-19	SIRT1	-0.427	1.88E-13	EMP2	-0.334	7.26E-11	PPFIBP1	-0.335	3.10E-09	MYOM2	-0.380	8.84E-08	CFHR4	-0.597	1.37E-06	MYO5A	0.269	2.19E-05	MOGAT3	0.333	1.17E-03
OSBPL6	-0.650	4.84E-19	GPD1	0.465	1.90E-13	FAP	0.513	7.45E-11	KLHL11	-0.290	3.12E-09	PRKCI	-0.265	8.91E-08	DAO	0.328	1.37E-06	CDC42EP1	0.351	2.27E-05	TNS2	-0.268	1.17E-03
LYZ	1.057	5.09E-19	CSTB	0.509	1.98E-13	PTPRU	0.470	7.57E-11	SNX7	0.291	3.14E-09	TDRD10	-0.425	8.92E-08	LRRC70	-0.266	1.38E-06	LCN2	0.391	2.27E-05	ARMH1	-0.274	1.23E-03
AEN	0.576	5.80E-19	MAP2K1	-0.439	2.03E-13	KIAA1958	-0.310	7.57E-11	BTAF1	-0.393	3.15E-09	BAG3	0.298	8.99E-08	RHBDD3	0.334	1.38E-06	HOOK1	-0.303	2.28E-05	TEDC1	0.338	1.24E-03
YTHDC1	-0.311	5.91E-19	COL5A1	0.682	2.06E-13	CYTH2	0.348	7.87E-11	EFHD1	-0.983	3.23E-09	MBTD1	-0.307	9.00E-08	MIDN	-0.351	1.40E-06	CHIC1	-0.343	2.32E-05	UGT2B11	0.267	1.25E-03
HS1BP3	0.408	7.05E-19	C1RL	-0.353	2.08E-13	ZMYM6	-0.300	8.28E-11	RAD54L2	-0.413	3.23E-09	RAPH1	-0.412	9.08E-08	EDA	-0.292	1.40E-06	LAG3	-0.301	2.33E-05	ADGRF5	-0.296	1.26E-03
C8A	-0.424	8.51E-19	PGAP4	0.497	2.10E-13	MON2	-0.406	8.50E-11	MASP2	-0.302	3.24E-09	TNKS2	-0.302	9.12E-08	PTPRH	0.404	1.40E-06	VTCN1	0.366	2.33E-05	ABCA2	0.264	1.27E-03
AKR1B10	2.253	9.38E-19	SH3PXD2A	-0.751	2.14E-13	ARHGAP30	0.441	8.61E-11	SYDE2	-0.290	3.24E-09	PRXL2A	0.309	9.21E-08	TLCD1	0.293	1.41E-06	CCDC68	-0.272	2.35E-05	PRSS8	0.280	1.29E-03
FMO1	1.047	9.56E-19	PLGLB2	-0.457	2.19E-13	MYO9A	-0.374	8.61E-11	GTF2IRD2	-0.323	3.24E-09	ALPL	-0.354	9.23E-08	SLC17A1	-0.359	1.42E-06	CD74	0.409	2.38E-05	SPON1	0.325	1.33E-03
HAL	-1.003	9.72E-19	E2F1	0.549	2.20E-13	ARHGEF26	-0.473	8.63E-11	BCL2L1	0.302	3.24E-09	ATF7IP	-0.280	9.32E-08	ADGRG7	-0.399	1.43E-06	BSN	-0.308	2.41E-05	CHRNA4	-0.563	1.37E-03
FZD5	-0.385	9.86E-19	PRDM10	-0.311	2.21E-13	TUSC1	-0.307	8.78E-11	ATP9A	0.340	3.24E-09	GRIP1	-0.268	9.53E-08	FBXO8	0.339	1.47E-06	MCF2L	-0.302	2.41E-05	VWA1	0.275	1.37E-03
SCPEP1	0.466	1.17E-18	LRRC59	0.425	2.24E-13	TBL1XR1	-0.487	8.79E-11	CDKN3	0.332	3.32E-09	ERMARD	-0.264	9.62E-08	ITGA3	0.416	1.49E-06	ADM2	0.280	2.43E-05	FGF21	0.304	1.38E-03

CCL15	-0.427	1.21E-18	OAT	-0.887	2.31E-13	NPAS3	-0.322	8.82E-11	ZNF595	-0.268	3.33E-09	ZNF331	-0.310	9.72E-08	NPBWR1	0.780	1.49E-06	KLHL25	0.266	2.46E-05	RHBG	-0.445	1.39E-03
SMAD4	-0.322	1.27E-18	KLHL29	0.692	2.35E-13	CYB5R2	0.401	8.82E-11	SLC12A1	0.594	3.40E-09	IQGAP2	-0.319	9.72E-08	FBXO28	-0.307	1.53E-06	LCK	0.267	2.46E-05	PAPLN	0.286	1.39E-03
RNF152	-0.925	1.47E-18	CDC6	0.582	2.45E-13	KCNN2	-0.558	9.04E-11	NRIP3	0.313	3.42E-09	ANGPT2	-0.404	9.74E-08	GABRE	0.519	1.53E-06	PAMR1	0.270	2.48E-05	ANKRD36	0.280	1.49E-03
ZMAT3	0.755	1.47E-18	MAF	-0.445	2.45E-13	TTYH3	0.529	9.04E-11	E2F8	0.334	3.42E-09	TXNDC5	-0.384	9.81E-08	PMVK	0.299	1.54E-06	CDC42EP3	0.266	2.49E-05	MDM4	-0.279	1.50E-03
EML2	0.355	1.69E-18	SNX12	0.293	2.49E-13	NHLRC2	-0.392	9.09E-11	ABR	0.346	3.51E-09	CCR2	0.369	9.83E-08	PGA3	-0.265	1.54E-06	PJVK	-0.303	2.54E-05	COL4A3	0.327	1.53E-03
RPS10	-0.430	1.79E-18	YAE1	-0.310	2.50E-13	HMMR	0.401	9.26E-11	CLDN14	-0.425	3.53E-09	PEG10	1.003	9.97E-08	ENPP3	-0.399	1.56E-06	ANXA10	-0.303	2.55E-05	CLEC1B	-0.322	1.56E-03
TMCO6	-0.498	2.09E-18	STYX	-0.520	2.55E-13	MARS1	0.273	9.65E-11	USPL1	-0.291	3.65E-09	CPS1	-0.468	1.01E-07	XKR4	-0.339	1.56E-06	H2AJ	0.325	2.55E-05	DCXR	-0.275	1.79E-03
HEY2	-0.377	2.22E-18	CDC25A	0.377	2.58E-13	ZCCHC9	-0.313	9.73E-11	TRIR	-0.396	3.67E-09	FRYL	-0.294	1.01E-07	FAM124B	-0.451	1.56E-06	GPAA1	0.274	2.55E-05	SYT12	0.267	1.82E-03
BAX	0.532	2.28E-18	MAST1	-0.451	2.60E-13	PLK2	0.325	9.73E-11	FDXR	0.403	3.70E-09	ITGAX	0.464	1.02E-07	TCIM	-0.472	1.57E-06	VASH1	0.267	2.55E-05	ZYX	0.272	1.82E-03
ACVR2B	-0.494	2.58E-18	CCDC9B	0.531	2.66E-13	NFKBIZ	-0.489	9.73E-11	IER5	0.291	3.79E-09	CD248	0.329	1.02E-07	SCIN	0.286	1.58E-06	ENTPD8	0.287	2.55E-05	PDGFRA	0.277	1.87E-03
TMEM102	0.416	2.60E-18	LUM	0.946	2.87E-13	AP1S3	0.324	1.01E-10	PHKA1	-0.335	3.84E-09	CHID1	0.318	1.02E-07	CORO1A	0.367	1.61E-06	RGS1	-0.443	2.56E-05	SUSD4	0.368	1.95E-03
TRIT1	-0.352	2.63E-18	BCAP31	0.344	2.89E-13	FBXL4	-0.363	1.04E-10	ASTN2	0.278	3.89E-09	LAMB3	0.369	1.02E-07	IDH2	0.331	1.61E-06	F13A1	0.397	2.61E-05	CYP2A7	-0.557	1.99E-03
RBMS1	-0.561	2.70E-18	COL4A2	0.695	2.89E-13	USP6NL	-0.305	1.04E-10	ACOT12	0.381	3.93E-09	IP6K3	0.754	1.03E-07	LGALS1	0.429	1.62E-06	NBPF20	0.271	2.62E-05	TRPV1	-0.274	1.99E-03
DHRS9	0.614	3.14E-18	ORC2	-0.444	2.98E-13	DRAM1	0.300	1.05E-10	PNO1	0.270	4.00E-09	BRMS1	0.295	1.03E-07	GRB14	-0.327	1.62E-06	RPL38	-0.307	2.64E-05	SAA1	0.660	2.00E-03
CTNNA3	-0.881	3.70E-18	EMP3	0.395	3.01E-13	ART5	-0.441	1.12E-10	HIPK3	-0.489	4.01E-09	KRT222	0.384	1.04E-07	SLC38A2	-0.456	1.63E-06	RALGPS2	-0.296	2.64E-05	SLC1A7	0.287	2.02E-03
KIAA1217	-0.440	3.77E-18	C19orf25	0.450	3.04E-13	PGD	0.378	1.12E-10	CNPY3	-0.379	4.04E-09	MAD2L2	0.293	1.04E-07	ACKR3	0.426	1.64E-06	PLLP	-0.291	2.66E-05	KDM8	0.271	2.06E-03
TMEM19	0.580	4.15E-18	PRAMEF33	0.656	3.07E-13	DICER1	-0.409	1.13E-10	PRH1	-0.282	4.09E-09	FLT4	-0.454	1.08E-07	PAXBP1	-0.324	1.64E-06	MPDZ	-0.271	2.67E-05	SDS	-0.515	2.12E-03
NALCN	0.694	4.16E-18	COMP	0.524	3.31E-13	CARMIL3	-0.510	1.14E-10	TAT	-0.757	4.18E-09	BSG	0.339	1.08E-07	CDH2	-0.269	1.66E-06	DGKH	-0.274	2.67E-05	PZP	-0.850	2.25E-03
TNIK	-0.628	4.27E-18	PTPRT	-0.516	3.36E-13	DNAJB5	0.283	1.15E-10	NCAPH	0.283	4.22E-09	GALNT17	0.359	1.08E-07	CPEB2	-0.333	1.67E-06	ARHGEF37	0.362	2.69E-05	ANKS1B	0.287	2.30E-03
TCF19	0.561	4.52E-18	DYNLT1	0.412	3.45E-13	ATL2	-0.301	1.17E-10	RORA	-0.592	4.27E-09	APOM	-0.372	1.09E-07	DUSP23	-0.364	1.67E-06	MME	-0.377	2.74E-05	FBRSL1	-0.281	2.32E-03
GPNMB	1.041	5.07E-18	MED13L	-0.431	3.47E-13	MPV17L2	0.427	1.17E-10	ABCG2	-0.445	4.28E-09	ANXA4	0.269	1.12E-07	MDFI	0.288	1.68E-06	EBI3	-0.284	2.80E-05	ONECUT1	-0.287	2.38E-03
PDIA5	-0.399	5.14E-18	AMOTL1	-0.461	3.48E-13	TMEM184B	0.305	1.19E-10	ERICH2	-0.268	4.38E-09	BSPRY	0.276	1.14E-07	EPOR	-0.363	1.69E-06	ITM2A	0.289	2.81E-05	MBD3	0.290	2.39E-03
THY1	1.020	5.82E-18	IRS2	-0.713	3.55E-13	MAN1C1	-0.450	1.20E-10	TIGAR	0.422	4.38E-09	SYNGR2	0.288	1.15E-07	RAB25	0.379	1.69E-06	MYDGF	0.269	2.82E-05	SOX9	0.296	2.42E-03
CCT5	0.311	5.92E-18	AHCY	-0.355	3.76E-13	EMP1	0.819	1.21E-10	DCUN1D4	-0.378	4.38E-09	GFRA2	-0.387	1.15E-07	SMC5	-0.272	1.72E-06	NR4A2	-0.314	2.83E-05	LINGO4	0.284	2.47E-03
ACVR1C	-0.593	6.33E-18	AMFR	0.296	3.76E-13	DNAJA1	0.334	1.21E-10	CLASP1	-0.269	4.55E-09	ACP5	0.450	1.16E-07	CLIP2	0.298	1.72E-06	SORCS1	-0.285	2.83E-05	ANKRD24	-0.290	2.50E-03
PPP1R3G	-0.748	6.64E-18	AZGP1	-0.399	4.19E-13	GLI1	0.334	1.21E-10	GADD45B	-0.652	4.55E-09	NECTIN1	0.318	1.17E-07	NAT2	-0.295	1.74E-06	GSKIP	0.266	2.92E-05	FDPS	0.399	2.57E-03
LSP1	0.626	6.67E-18	NOTCH2NLA	-0.381	4.25E-13	WSB2	0.389	1.23E-10	PTPRS	-0.650	4.57E-09	TUBB4B	0.361	1.19E-07	ATP5F1E	0.276	1.76E-06	TLE2	-0.297	3.03E-05	FCN2	-0.325	2.62E-03
KCNT2	-0.599	6.75E-18	NR5A2	-0.392	4.26E-13	DNM1	-0.775	1.23E-10	CASP4	0.288	4.57E-09	PKDCC	0.309	1.19E-07	FZD6	-0.291	1.77E-06	CPSF7	-0.266	3.05E-05	HMGCS1	0.551	2.63E-03
LPA	-1.046	7.21E-18	DPPA4	-0.397	4.40E-13	PPIC	0.328	1.23E-10	EPHB3	0.337	4.65E-09	NES	0.461	1.20E-07	ALPK2	0.783	1.79E-06	DCHS2	-0.265	3.18E-05	SLC7A8	-0.272	2.66E-03
SFXN2	-0.426	7.48E-18	SYT7	0.732	4.45E-13	CCL14	-0.306	1.24E-10	HSPA5	0.521	4.76E-09	MCM10	0.319	1.22E-07	ANKRD52	0.268	1.79E-06	MAN2A1	-0.329	3.26E-05	PNPLA2	-0.272	2.68E-03
ACTR1A	0.301	7.70E-18	EDN2	0.445	4.45E-13	MTMR7	-0.336	1.25E-10	DPY19L3	-0.283	4.79E-09	TIMP1	0.495	1.26E-07	RPS27L	0.338	1.82E-06	ALPK3	0.321	3.27E-05	SLC16A11	0.303	2.74E-03
SPRING1	0.430	7.87E-18	GAB1	-0.363	4.67E-13	ZNF451	-0.312	1.37E-10	CUX2	-0.851	4.86E-09	FKBP10	0.339	1.28E-07	ARPC5	0.284	1.85E-06	CCSER1	-0.272	3.28E-05	OAS2	0.308	2.79E-03
FOXJ3	-0.319	8.77E-18	MAGI2	-0.427	4.83E-13	CUEDC1	0.321	1.38E-10	HELZ	-0.332	4.89E-09	TMIGD3	-0.455	1.28E-07	CFL2	-0.313	1.85E-06	PCED1A	-0.289	3.30E-05	CLGN	0.407	2.79E-03
BCAT1	0.717	9.43E-18	CLDN11	0.537	4.93E-13	GRAMD1C	-0.429	1.38E-10	SPSB2	0.346	4.95E-09	GOT1	-0.299	1.28E-07	PFKL	0.341	1.87E-06	S100A6	0.371	3.39E-05	MGP	0.414	2.93E-03
ENO1	0.411	1.04E-17	LRRC42	0.296	4.95E-13	ACOT13	-0.301	1.38E-10	CYCS	0.447	4.96E-09	F2RL1	0.420	1.29E-07	AKAP6	-0.269	1.87E-06	SLC35A3	-0.295	3.39E-05	DNAH5	-0.277	2.98E-03
KBTBD11	-0.607	1.14E-17	KIAA0930	0.337	5.00E-13	SMN1	-0.308	1.44E-10	MTHFD2	0.376	5.00E-09	APOL2	0.279	1.30E-07	KCNK1	-0.327	1.87E-06	NFASC	0.414	3.40E-05	IGFBP7	0.282	3.05E-03
CNKSR2	-0.604	1.24E-17	ESYT1	0.341	5.09E-13	SUSD2	0.461	1.46E-10	CALHM2	0.302	5.05E-09	GCHFR	-0.388	1.31E-07	FHIP2A	-0.295	1.88E-06	DPY19L4	-0.298	3.45E-05	GPX2	0.493	3.14E-03
CCN4	0.470	1.26E-17	BCKDHB	-0.392	5.13E-13	FKBP14	0.279	1.49E-10	PTPN7	0.377	5.29E-09	RALGAPA1	-0.310	1.37E-07	BAALC	-0.287	1.90E-06	THRSP	0.450	3.53E-05	PLIN5	-0.316	3.15E-03
MTARC2	-0.415	1.28E-17	CHPF2	0.323	5.18E-13	DSCAM	-0.355	1.52E-10	ITM2C	0.360	5.29E-09	GRN	0.396	1.38E-07	CHCHD10	-0.376	1.91E-06	PITPNM1	0.285	3.58E-05	TMEM256	-0.270	3.22E-03
FBXO46	-0.449	1.30E-17	SLC6A16	-0.576	5.26E-13	ANO7	-0.328	1.53E-10	METTL27	-0.357	5.29E-09	COL28A1	-0.590	1.38E-07	SPHK2	0.325	1.93E-06	ZSCAN23	-0.319	3.69E-05	C7	0.403	3.34E-03
FBXO2	0.763	1.40E-17	CKAP4	0.344	5.48E-13	PAQR5	0.456	1.56E-10	ERRFI1	-0.555	5.30E-09	CHD1	-0.288	1.40E-07	HP	-0.346	1.94E-06	NRG1	0.518	3.77E-05	BICC1	0.306	3.39E-03
ST14	0.681	1.50E-17	LIMCH1	-0.465	5.56E-13	DBNDD1	0.453	1.57E-10	C2CD4B	-0.264	5.30E-09	CCDC112	0.287	1.41E-07	HLA-DQB1	0.487	1.98E-06	PTMS	-0.328	3.96E-05	IFI27	0.376	3.47E-03
RNF103	-0.363	1.54E-17	PABPC4	-0.276	5.66E-13	ME1	0.691	1.67E-10	STIP1	0.278	5.34E-09	COL5A2	0.363	1.42E-07	FOXA2	-0.296	2.01E-06	NKD1	0.280	4.01E-05	INMT	0.267	3.54E-03
CEBPB	-0.695	1.55E-17	SLC25A48	-0.377	5.76E-13	AACS	0.430	1.67E-10	CHMP1A	0.273	5.63E-09	CLEC4G	-0.495	1.42E-07	ABCA5	-0.361	2.04E-06	IGLL5	0.461	4.09E-05	MSR1	0.358	3.54E-03
CXCL9	1.150	1.59E-17	VCP	0.329	5.82E-13	SPSB1	0.527	1.69E-10	KIF4A	0.320	5.75E-09	FBXO44	0.324	1.44E-07	LIG4	-0.330	2.05E-06	HLA-F	0.362	4.16E-05	SFRP5	0.334	3.54E-03
ZFAND4	-0.313	1.70E-17	USP31	-0.361	5.87E-13	ETV7	0.389	1.69E-10	FOXO1	-0.454	5.90E-09	MAP1LC3A	0.410	1.44E-07	CCL18	0.318	2.08E-06	BAAT	-0.317	4.20E-05	DNAH11	-0.284	3.60E-03
BLNK	-0.479	2.04E-17	DEPDC5	-0.329	5.90E-13	AGXT2	-0.361	1.78E-10	VSNL1	-0.349	5.95E-09	TMEM233	-0.276	1.45E-07	DAAM2	-0.275	2.10E-06	SPOCK3	-0.317	4.20E-05	ADA2	0.276	3.70E-03
PTPRD	-0.619	2.06E-17	SERPINB8	0.506	5.90E-13	ITCH	-0.368	1.85E-10	RIMOC1	-0.385	5.99E-09	STRN3	-0.296	1.49E-07	ZNF518A	-0.315	2.10E-06	HSD17B14	-0.555	4.22E-05	SORT1	0.523	3.77E-03
PSME3	0.339	2.17E-17	LAMA2	0.648	5.93E-13	DKK3	0.596	1.85E-10	MAP3K21	-0.296	6.00E-09	TMEM161B	-0.311	1.50E-07	ELFN2	0.454	2.12E-06	CXCL6	0.402	4.24E-05	G6PC1	-0.421	3.86E-03
PRAMEF10	0.896	2.37E-17	INPP5B	-0.366	6.16E-13	METAP1D	-0.296	1.86E-10	SAT1	-0.320	6.04E-09	PGC	-0.385	1.52E-07	KIF25	-0.278	2.21E-06	N4BP2L1	-0.298	4.34E-05	SDF2L1	0.278	3.92E-03
ALDH6A1	-0.710	2.50E-17	TMEM154	0.990	6.36E-13	CENPU	0.439	1.87E-10	SMIM14	-0.330	6.04E-09	MPC1	-0.352	1.55E-07	PITPNM3	-0.378	2.21E-06	TMSB10	0.360	4.35E-05	MS4A6A	-0.275	3.96E-03
FNBP1	-0.364	2.54E-17	GALNT18	0.468	6.36E-13	UBR3	-0.393	1.89E-10	ZFHX4	-0.373	6.20E-09	HAO2	-0.308	1.59E-07	PDE3B	-0.329	2.24E-06	IL2RG	0.297	4.43E-05	CD68	0.324	3.97E-03
ZBTB10	-0.557	2.69E-17	GPRC5B	0.549	6.36E-13	EGR1	-1.309	1.91E-10	POU6F2	-0.462	6.21E-09	AFDN	-0.275	1.59E-07	PROX1	-0.349	2.27E-06	TMED6	-0.333	4.48E-05	CP	-0.312	4.09E-03
HSP90AB1	0.372	2.89E-17	RADX	-0.360	6.42E-13	MIOS	-0.264	1.91E-10	ANG	-0.440	6.21E-09	ENPP7	0.486	1.60E-07	UQCRFS1	0.271	2.29E-06	SUMO4	0.271	4.63E-05	CD209	-0.315	4.16E-03

PACSIN3	-0.700	2.91E-17	ABCC4	0.574	6.42E-13	DPYSL2	0.422	1.92E-10	CHRNE	-0.302	6.46E-09	REELD1	-0.375	1.61E-07	CBLN4	-0.342	2.34E-06	HLA-DQA1	0.566	4.78E-05	MZB1	0.271	4.17E-03
VIL1	-1.440	3.14E-17	CAPNS1	0.354	6.54E-13	ABCA9	-0.546	1.93E-10	HLA-A	0.474	6.59E-09	CDCA2	0.277	1.62E-07	NDC80	0.276	2.34E-06	KCNJ10	-0.422	4.86E-05	GSTM3	-0.375	4.24E-03
FABP5	0.674	3.32E-17	PDS5A	-0.308	6.86E-13	PSMD1	0.280	1.95E-10	SIK2	-0.343	6.66E-09	RPL14	-0.280	1.62E-07	KLF16	0.295	2.36E-06	APBA1	-0.286	4.93E-05	ELOVL6	0.291	4.24E-03
MEOX1	0.485	3.36E-17	RGS7	-0.599	7.30E-13	GGT5	0.498	2.00E-10	EXPH5	-0.444	6.79E-09	NPIPA2	-0.288	1.62E-07	NPR1	-0.334	2.36E-06	GREB1	-0.313	4.98E-05	MAB21L4	0.532	4.32E-03
DCAF6	-0.315	3.72E-17	TMBIM1	0.370	7.31E-13	PPA1	0.357	2.01E-10	SEC61A1	0.272	6.80E-09	TAF13	0.327	1.63E-07	NSDHL	0.461	2.38E-06	STEAP2	-0.307	5.00E-05	MT1H	-0.556	4.38E-03
ELL2	-0.527	3.87E-17	PRKRA	-0.329	7.79E-13	MEAF6	0.313	2.17E-10	BUB1	0.319	6.86E-09	METTL24	0.308	1.64E-07	IZUMO4	-0.298	2.39E-06	FXYD6	-0.273	5.01E-05	ELN	-0.302	4.40E-03
C6orf47	0.327	3.87E-17	SLC16A3	0.431	7.88E-13	MYBL2	0.369	2.17E-10	ERICH3	-0.310	6.87E-09	COL6A3	0.374	1.66E-07	PLAG1	-0.297	2.39E-06	SEC11C	0.282	5.07E-05	IL20RB	-0.512	4.46E-03
PHF23	0.397	3.89E-17	TIMM17A	0.426	8.05E-13	CLDN23	-0.285	2.18E-10	MAP4	0.282	7.01E-09	ITPR2	-0.346	1.67E-07	MAP1A	0.330	2.39E-06	UGT2B10	0.302	5.12E-05	LAPTM5	0.273	4.48E-03
MVP	0.650	3.95E-17	TAPT1	-0.312	8.14E-13	FOXP1	-0.325	2.21E-10	ZNF546	-0.270	7.27E-09	ZNF132	0.265	1.67E-07	CHRM3	-0.483	2.43E-06	ITGAV	0.318	5.17E-05	TAGLN	0.345	4.68E-03
RRM2	0.718	4.14E-17	TFPI	-0.461	8.17E-13	GTPBP10	-0.272	2.21E-10	EGFLAM	-0.422	7.28E-09	PWWP2B	0.325	1.68E-07	C1orf122	0.301	2.49E-06	LGI1	-0.428	5.21E-05	CD163	-0.400	4.80E-03
MACO1	-0.289	4.77E-17	CDO1	-0.357	8.17E-13	MYO5B	0.307	2.22E-10	DLGAP5	0.349	7.40E-09	GOLIM4	-0.297	1.71E-07	NOTCH4	-0.397	2.50E-06	DDIT4	0.451	5.22E-05	SLC28A1	0.266	5.09E-03
MET	-0.553	5.11E-17	PCNA	0.374	8.69E-13	NPM2	0.407	2.25E-10	RHBDF1	0.325	7.40E-09	CD48	0.362	1.72E-07	ABCA13	-0.392	2.50E-06	N4BP2	-0.289	5.22E-05	ASPG	-0.300	5.09E-03
TRABD2B	-0.802	5.27E-17	INKA2	0.349	8.78E-13	SFRP4	0.501	2.26E-10	CUL4B	-0.299	7.73E-09	NFIA	-0.287	1.74E-07	MNDA	0.341	2.51E-06	SNED1	-0.283	5.22E-05	PKHD1L1	-0.387	5.11E-03
MEF2A	-0.397	5.38E-17	GOSR2	0.273	9.12E-13	H4C8	0.295	2.27E-10	MOCS2	-0.273	7.78E-09	SLK	-0.270	1.78E-07	QSER1	-0.269	2.67E-06	P2RX3	-0.288	5.24E-05	REX1BD	-0.274	5.13E-03
AASS	-0.784	5.43E-17	C9orf43	-0.345	9.27E-13	ROCK2	-0.292	2.29E-10	ERICH5	0.491	7.84E-09	ADGRG6	-0.396	1.78E-07	UGT2A3	0.488	2.67E-06	CLIC6	0.347	5.27E-05	TM7SF2	0.292	5.21E-03
EHD4	0.438	6.11E-17	ESPNL	-0.660	9.31E-13	ERBB2	0.418	2.38E-10	MAFG	0.268	7.88E-09	LAIR1	0.353	1.84E-07	ARMC12	-0.320	2.69E-06	GK	-0.327	5.40E-05	CYP17A1	-0.312	5.30E-03
ATXN7L1	-0.375	6.21E-17	CHI3L1	1.497	9.48E-13	LGSN	-0.486	2.39E-10	PLOD3	0.315	7.89E-09	EFEMP1	0.751	1.85E-07	CPEB4	-0.399	2.73E-06	RFTN1	0.281	5.77E-05	FADS1	0.491	5.34E-03
SLAMF8	0.562	6.86E-17	FXN	-0.301	9.67E-13	CA14	-0.515	2.45E-10	NPR3	0.426	7.94E-09	DTYMK	0.277	1.86E-07	SLC1A1	-0.320	2.80E-06	MMRN1	-0.482	5.82E-05	GSTM4	-0.511	5.36E-03
SLC35B2	0.358	7.00E-17	EFNB3	0.361	1.00E-12	POU2F1	-0.288	2.48E-10	FAAH2	-0.346	8.09E-09	NUDT13	-0.320	1.91E-07	SERPINF2	-0.272	2.80E-06	H2AC6	0.289	5.85E-05	PYROXD2	0.375	5.41E-03
QSOX1	0.553	7.92E-17	SULF2	0.591	1.01E-12	FZD4	-0.291	2.48E-10	MT2A	-0.689	8.20E-09	IFI27L2	0.318	1.91E-07	HS6ST1	0.288	2.83E-06	ZNHIT1	-0.280	6.05E-05	ZBED6	-0.360	5.65E-03
DAXX	0.340	8.18E-17	TMEM140	0.342	1.03E-12	SEC61A2	-0.323	2.51E-10	SH3RF2	-0.367	8.22E-09	SIGLEC1	-0.511	1.96E-07	GCNT4	0.315	2.87E-06	CD4	-0.379	6.11E-05	ITGAD	-0.348	5.66E-03
FAM227B	-0.412	8.20E-17	HSD17B11	-0.538	1.03E-12	CARNMT1	-0.377	2.54E-10	PTTG1	0.348	8.35E-09	HLA-B	0.410	1.97E-07	PMP2	-0.280	2.87E-06	CSTA	0.428	6.18E-05	ALDOC	0.291	5.86E-03
LRRC8E	0.438	9.20E-17	APOBEC3B	0.361	1.07E-12	KCNC2	-0.473	2.61E-10	AQP4	0.469	8.56E-09	SLC22A12	0.365	1.99E-07	BCHE	-0.421	2.88E-06	GSTP1	0.292	6.23E-05	P2RY13	-0.272	5.87E-03
DISP1	-0.345	9.75E-17	PDLIM5	-0.374	1.10E-12	NTN4	-0.479	2.63E-10	PPIL1	0.311	8.70E-09	UFL1	-0.336	2.01E-07	PLEKHO2	0.320	2.90E-06	NAV3	0.326	6.26E-05	ETNPPL	0.282	5.87E-03
C2CD5	-0.419	9.84E-17	DCN	0.561	1.12E-12	RCL1	-0.395	2.67E-10	FOXM1	0.383	8.74E-09	STRN	-0.297	2.05E-07	CECR2	-0.356	2.92E-06	BACE2	0.282	6.30E-05	EHF	0.270	6.42E-03
LOXL4	0.870	9.84E-17	LMTK2	-0.311	1.14E-12	JAML	0.386	2.72E-10	TLCD4	-0.470	8.81E-09	MAOA	-0.284	2.06E-07	ACAA2	-0.326	2.95E-06	PPT1	0.264	6.55E-05	DNASE1L3	-0.277	6.51E-03
MT1E	-1.053	9.92E-17	UBE2T	0.374	1.15E-12	AADAT	-0.361	2.74E-10	ATP6V1F	0.290	9.09E-09	IL6R	-0.340	2.07E-07	SNRK	-0.292	2.99E-06	TBC1D10C	0.273	6.69E-05	MRPL41	-0.267	6.56E-03
COBLL1	-0.514	1.01E-16	PPFIA3	0.395	1.16E-12	PSMD14	0.337	2.77E-10	NUDT19	-0.278	9.09E-09	HSPH1	0.334	2.09E-07	SDC2	-0.279	3.03E-06	FAM171A1	-0.278	6.74E-05	PNPLA7	-0.274	6.62E-03
ITGBL1	0.909	1.03E-16	IL1RAP	-0.702	1.19E-12	GPR176	0.355	2.77E-10	KIFC1	0.314	9.26E-09	WDR76	0.280	2.10E-07	VTN	0.270	3.05E-06	FAT4	-0.267	6.81E-05	CES1	0.308	6.75E-03
ABHD18	-0.347	1.12E-16	TPPP2	-0.497	1.21E-12	ZNF407	-0.283	2.82E-10	COL5A3	0.852	9.27E-09	NIPAL2	0.357	2.12E-07	SLC16A12	-0.453	3.08E-06	GPR174	0.280	6.92E-05	RND2	-0.314	6.77E-03
BAK1	0.402	1.13E-16	P2RY2	0.502	1.25E-12	MGAT2	0.312	2.82E-10	CERCAM	0.359	9.58E-09	TLE1	-0.277	2.20E-07	GLS2	-0.550	3.08E-06	ERV3-1	0.306	7.01E-05	FMO2	0.298	6.88E-03
PCBP2	-0.350	1.13E-16	EBPL	-0.499	1.33E-12	ZBED3	-0.404	2.92E-10	SRPRB	0.293	9.66E-09	LYVE1	-0.782	2.22E-07	OAS1	0.351	3.09E-06	ATOH7	-0.285	7.01E-05	VCAM1	-0.396	7.00E-03
IFI30	0.644	1.14E-16	CNBP	-0.294	1.35E-12	RBMS2	-0.344	2.93E-10	TLNRD1	-0.272	9.72E-09	RHOC	0.300	2.34E-07	MLIP	0.820	3.12E-06	MMP7	0.425	7.20E-05	PLCXD3	0.268	7.12E-03
SPIDR	-0.317	1.14E-16	NUP62CL	-0.338	1.36E-12	DCDC1	-0.582	3.01E-10	MTA1	-0.362	1.00E-08	CFHR5	-0.397	2.36E-07	TRPM2	0.330	3.12E-06	CLDN7	0.300	7.23E-05	ATP5F1D	-0.270	7.41E-03
CCDC196	-0.568	1.20E-16	TAF4	-0.302	1.36E-12	PAQR4	0.337	3.01E-10	TUBA1A	0.397	1.01E-08	HYOU1	0.443	2.47E-07	CCDC80	0.571	3.15E-06	NFIC	-0.331	7.39E-05	SLC2A12	-0.263	7.46E-03
FRMD4B	-0.422	1.23E-16	FBXO11	-0.356	1.39E-12	EXOC1	-0.294	3.04E-10	DCSTAMP	0.313	1.02E-08	PKLR	0.440	2.51E-07	PFKFB1	-0.308	3.15E-06	GPAM	0.349	7.53E-05	ZNF385D	0.331	7.98E-03
TUBA1B	0.512	1.25E-16	ZNF544	0.376	1.42E-12	ZNF280D	-0.343	3.09E-10	PLA2G4C	0.422	1.02E-08	PDE11A	0.633	2.52E-07	GEM	0.431	3.16E-06	RAP2A	0.274	7.79E-05	NR1D2	-0.295	8.57E-03
TCF7L1	-0.501	1.28E-16	GPR88	-0.968	1.43E-12	KDM4A	0.276	3.31E-10	ANXA5	0.378	1.06E-08	ATG101	0.286	2.52E-07	ADH1B	-0.315	3.16E-06	CAPN3	-0.435	7.81E-05	PPDPF	0.268	8.91E-03
RPL39	-0.812	1.29E-16	PKM	0.513	1.48E-12	PGK1	0.311	3.34E-10	TRAF7	0.327	1.09E-08	GLUD2	0.329	2.56E-07	SERINC2	0.342	3.22E-06	AKAP12	-0.406	7.81E-05	MS4A7	-0.288	9.46E-03
HYAL3	0.524	1.31E-16	OGA	-0.318	1.48E-12	MTMR4	-0.348	3.41E-10	CPNE6	-0.436	1.11E-08	MIDEAS	-0.273	2.58E-07	FCN1	0.422	3.25E-06	GCK	0.644	8.03E-05	TSKU	-0.265	9.50E-03
KCTD11	0.280	1.36E-16	SPAG9	-0.386	1.49E-12	LAMB2	0.409	3.46E-10	SOX5	-0.419	1.11E-08	CASP1	0.293	2.59E-07	OGT	-0.312	3.28E-06	TFCP2L1	-0.339	8.09E-05	NPNT	0.314	9.73E-03
RALGPS1	0.545	1.39E-16	LRRC31	0.620	1.52E-12	SIM1	-0.557	3.46E-10	VMA21	-0.263	1.12E-08	FAM83A	-0.266	2.61E-07	CFP	-0.491	3.34E-06	CDH11	0.367	8.23E-05	IFITM10	-0.271	9.79E-03
SLC25A37	-0.540	1.55E-16	SLC19A1	0.582	1.66E-12	SLC13A5	-0.419	3.48E-10	ZNF684	-0.328	1.12E-08	BRWD3	-0.332	2.62E-07	KCNJ2	-0.370	3.37E-06	ANKRD18A	0.393	8.29E-05	CFAP74	-0.314	1.00E-02
EPDR1	0.471	2.07E-16	CAMK2D	-0.359	1.70E-12	ATP2B2	-0.376	3.53E-10	SETDB2	-0.303	1.12E-08	ADH1C	-0.393	2.67E-07	IGF1R	-0.275	3.42E-06	SQLE	0.705	8.40E-05	GSTT2B	-0.476	1.04E-02
ITGA6	0.556	2.14E-16	ADAM15	0.379	1.76E-12	TOB1	-0.421	3.54E-10	SNX31	-0.279	1.13E-08	ZBTB33	0.415	2.67E-07	POT1	-0.277	3.47E-06	NECTIN3	-0.279	8.44E-05	UGT2B17	-0.907	1.07E-02
BICRAL	-0.333	2.14E-16	TBCEL	-0.416	1.76E-12	PTBP2	-0.412	3.55E-10	AMY2B	-0.450	1.14E-08	TMX3	-0.402	2.68E-07	EGLN3	-0.285	3.47E-06	PHLDB3	-0.263	8.60E-05	EHD3	-0.268	1.09E-02
FAM83G	0.702	2.30E-16	SLC23A2	-0.586	1.76E-12	GIPC1	0.382	3.58E-10	NUDT1	0.287	1.15E-08	KRT7	0.617	2.69E-07	MATK	0.271	3.48E-06	WNT11	0.380	8.71E-05	DOP1B	-0.348	1.10E-02
AIG1	-0.326	2.59E-16	ADAMTSL2	0.736	1.77E-12	GOLM1	0.564	3.69E-10	ZNF121	-0.301	1.19E-08	SLC27A4	0.288	2.76E-07	PTK7	0.320	3.51E-06	PAK3	0.342	8.73E-05	LRP1B	-0.356	1.11E-02
SLC29A3	0.334	2.60E-16	WNT4	0.519	1.77E-12	MOCS1	-0.285	3.69E-10	ACOT7	0.290	1.19E-08	C11orf96	-0.385	2.76E-07	PLAAT3	-0.316	3.53E-06	PPP1R3C	0.472	8.75E-05	PLCH2	0.319	1.12E-02
FSTL3	0.678	2.60E-16	ATP6V0D2	0.343	1.88E-12	VAT1	0.275	3.69E-10	UBE3D	-0.268	1.20E-08	SHISA9	-0.386	2.77E-07	STAB1	-0.511	3.60E-06	TRPM7	-0.285	8.79E-05	NOMO3	0.274	1.12E-02
KALRN	-0.390	2.60E-16	SORBS1	-0.365	1.95E-12	STK17A	0.355	3.69E-10	SULT1E1	-0.568	1.20E-08	HBB	1.001	2.79E-07	PTGDS	0.642	3.71E-06	CYP3A43	-0.491	8.85E-05	SLC29A4	0.363	1.13E-02
PRC1	0.480	2.72E-16	TTC38	-0.305	1.99E-12	MOXD1	0.646	3.69E-10	TMEM220	-0.340	1.21E-08	RGS5	-0.515	2.80E-07	SLC27A5	-0.327	3.72E-06	ARHGEF40	-0.275	9.06E-05	IFI6	0.383	1.14E-02
RASL10B	-0.572	3.14E-16	ABCB4	0.529	2.02E-12	ABCA6	-0.425	3.76E-10	NOTUM	0.497	1.22E-08	NR1I3	-0.293	2.83E-07	SETD1B	-0.343	3.74E-06	AQP8	0.298	9.09E-05	JUN	0.281	1.16E-02
HTRA1	0.461	3.14E-16	ACER3	0.379	2.03E-12	LBH	0.455	3.77E-10	MORC3	-0.331	1.24E-08	FASN	0.769	2.84E-07	CTSS	0.398	3.76E-06	NEGR1	-0.292	9.35E-05	CYFIP2	-0.303	1.21E-02

ICMT	0.320	3.19E-16	NFE2L2	-0.319	2.03E-12	CDK4	0.302	3.92E-10	CCDC85C	0.315	1.25E-08	ELMOD1	-0.282	2.89E-07	GLYCTK	-0.318	3.76E-06	SULT1A1	-0.362	9.58E-05	SHBG	-0.322	1.21E-02
DLC1	-0.371	3.28E-16	RET	0.837	2.07E-12	ITPKC	0.291	3.95E-10	BANF1	0.333	1.27E-08	GRAMD4	-0.386	2.89E-07	VIM	0.390	3.77E-06	ETFRF1	-0.363	9.61E-05	EVC	0.321	1.22E-02
PRXL2B	0.407	3.60E-16	DNMT1	0.382	2.13E-12	ANXA6	0.437	4.05E-10	MAMDC4	0.767	1.27E-08	EZH2	0.281	2.91E-07	USP34	-0.306	3.81E-06	SAA4	0.266	9.66E-05	DDT	-0.293	1.25E-02
ARHGEF4	-0.556	4.20E-16	CA12	0.916	2.19E-12	GM2A	0.286	4.08E-10	PIK3IP1	0.376	1.27E-08	JAKMIP2	-0.375	2.95E-07	ETS2	-0.319	3.91E-06	SPIC	-0.266	9.73E-05	MEP1B	0.314	1.27E-02
GINS2	0.597	4.22E-16	ABAT	-0.421	2.19E-12	ASXL3	-0.438	4.12E-10	EPB41L4B	-0.298	1.29E-08	CYP4A11	-0.354	3.01E-07	SLC17A3	-0.361	3.93E-06	ISG15	0.442	9.74E-05	CYP2A6	-0.294	1.36E-02
DIPK2A	-0.629	4.27E-16	DOK7	0.444	2.21E-12	SPN	0.426	4.14E-10	RASEF	0.522	1.29E-08	HMGCS2	-0.329	3.07E-07	DPYSL3	0.403	3.93E-06	RANBP17	-0.308	9.84E-05	CD84	0.277	1.40E-02
EMILIN2	0.536	4.39E-16	C9orf72	-0.466	2.22E-12	CYB561D2	0.291	4.34E-10	GDF2	0.396	1.30E-08	ESF1	0.372	3.13E-07	RBL2	-0.286	3.97E-06	TMEM238	-0.316	9.91E-05	MT1A	-0.317	1.43E-02
LIN7C	-0.523	4.63E-16	PACSIN2	0.324	2.30E-12	FBXO6	0.312	4.46E-10	EPHB2	0.323	1.32E-08	SP140	0.350	3.19E-07	HLA-DRB5	0.418	4.05E-06	TUBA4A	-0.282	1.00E-04	ASCL1	0.479	1.47E-02
ZNF385B	-0.480	4.67E-16	CDON	-0.395	2.31E-12	MCEE	-0.298	4.50E-10	MBNL3	-0.457	1.34E-08	USH2A	-0.552	3.20E-07	IRS1	-0.276	4.19E-06	EFCAB12	-0.284	1.01E-04	IGFALS	-0.323	1.47E-02
PLSCR4	-0.619	5.00E-16	ACHE	0.669	2.32E-12	RASGRP2	-0.496	4.64E-10	RPS6KA6	-0.308	1.34E-08	CHST7	-0.303	3.29E-07	ZNF511	-0.312	4.23E-06	IGSF23	-0.326	1.04E-04	GSTA2	-0.457	1.65E-02
ZNF507	-0.363	5.01E-16	CITED2	-0.432	2.35E-12	MRPS30	-0.278	4.70E-10	UGT1A6	0.657	1.34E-08	GPRC5A	-0.312	3.30E-07	SLC51B	0.299	4.25E-06	SLC3A1	-0.672	1.04E-04	THBS1	0.301	1.72E-02
SERPINH1	0.414	5.02E-16	WIPI1	0.365	2.35E-12	GPHN	-0.293	4.72E-10	SPSB4	-0.348	1.37E-08	BRD1	-0.269	3.31E-07	EPSTI1	0.378	4.25E-06	RNPEPL1	0.280	1.06E-04	NPW	-0.294	1.77E-02
RUNDC3B	-0.511	5.03E-16	SLC43A3	-0.364	2.35E-12	SYBU	-0.603	4.78E-10	FBN1	0.477	1.37E-08	RAB42	0.266	3.35E-07	RTP3	0.364	4.30E-06	CLCN7	0.291	1.07E-04	MLXIPL	-0.305	1.96E-02
MTMR10	-0.327	5.09E-16	CLPX	-0.302	2.39E-12	PKD2	-0.481	4.83E-10	ABCC9	-0.460	1.41E-08	GSN	0.538	3.38E-07	C11orf80	0.265	4.36E-06	BACH2	-0.342	1.07E-04	LSS	0.281	2.00E-02
NEURL1B	0.469	5.19E-16	FOXN4	0.686	2.41E-12	RETREG1	-0.594	4.84E-10	HMGA1	0.337	1.42E-08	LILRB5	-0.503	3.47E-07	CCDC88A	0.354	4.37E-06	DPT	0.545	1.07E-04	ACSL4	0.448	2.01E-02
TUBGCP3	-0.293	5.23E-16	SLC9A7	-0.296	2.43E-12	C6	-0.373	4.89E-10	ATP8A1	-0.372	1.45E-08	ANXA13	0.663	3.52E-07	COX5B	-0.343	4.42E-06	CLDN10	0.365	1.08E-04	ACSS2	0.264	2.20E-02
CCR5	0.516	5.44E-16	CDCA5	0.365	2.47E-12	EEF1A2	0.882	4.90E-10	ORMDL2	0.301	1.47E-08	PLEKHA6	-0.343	3.55E-07	SOX4	0.385	4.47E-06	GCAT	-0.283	1.09E-04	TNC	0.276	2.22E-02
RPS6KA1	0.528	5.99E-16	R3HDM2	-0.284	2.55E-12	ZNF638	-0.317	4.90E-10	ZFAND1	-0.284	1.49E-08	CALR	0.326	3.57E-07	TREH	0.552	4.54E-06	PC	-0.275	1.10E-04	TMC4	0.309	2.35E-02
KCNMA1	-0.704	6.19E-16	EXO1	0.390	2.59E-12	AQP1	0.770	4.92E-10	ENOSF1	-0.330	1.50E-08	RFX1	-0.346	3.58E-07	SLC49A3	0.385	4.56E-06	CTSK	0.279	1.10E-04	HEPACAM	-0.322	2.38E-02
HDAC11	0.398	7.10E-16	CRYM	0.448	2.60E-12	MYL6	0.380	5.11E-10	EMILIN1	0.489	1.53E-08	HAUS4	-0.279	3.58E-07	DOP1A	-0.308	4.57E-06	NDST3	-0.385	1.10E-04	KCNB1	0.294	2.51E-02
PPP1R15B	-0.348	7.22E-16	GRIA3	0.418	2.61E-12	VCAN	0.611	5.11E-10	MVK	0.487	1.53E-08	A1CF	-0.379	3.63E-07	TRIP6	0.279	4.57E-06	PLIN2	0.393	1.10E-04	NYNRIN	0.324	2.93E-02
NHSL1	-0.442	7.24E-16	LGALS4	1.153	2.61E-12	C11orf54	-0.329	5.12E-10	C8B	-0.312	1.55E-08	SLC2A14	0.368	3.63E-07	PDZK1IP1	0.444	4.58E-06	APOLD1	-0.290	1.12E-04	S100A9	0.270	2.97E-02
P4HA1	-1.003	7.30E-16	SERTAD2	-0.276	2.66E-12	GPR137	0.336	5.24E-10	NIPAL1	-0.500	1.57E-08	ZNF224	-0.324	3.70E-07	NDUFC2	-0.316	4.60E-06	PREX2	-0.280	1.14E-04	SMOC2	0.274	3.15E-02
GRTP1	-0.465	7.71E-16	POGZ	-0.420	2.70E-12	CROT	-0.338	5.36E-10	FAM76B	-0.412	1.60E-08	LRRC32	0.344	3.71E-07	CD3D	0.306	4.68E-06	UGT1A9	0.423	1.20E-04	CDH15	0.267	3.49E-02
THBS2	0.848	8.12E-16	TRIM24	-0.288	2.74E-12	ELOVL5	0.448	5.37E-10	P3H2	-0.285	1.64E-08	MOV10L1	-0.327	3.73E-07	NDUFA2	-0.338	4.68E-06	UTRN	-0.291	1.22E-04	SCUBE1	0.334	3.49E-02
GAS6	0.642	8.58E-16	SPTBN4	-0.368	2.74E-12	GCH1	-0.412	5.50E-10	EML4	-0.293	1.64E-08	RAD54B	-0.287	3.83E-07	MT1F	-0.725	4.71E-06	CAVIN1	0.287	1.22E-04	GSTM2	-0.757	3.50E-02
CREBBP	-0.401	8.83E-16	CHEK1	0.415	2.83E-12	TMEM138	0.319	5.58E-10	CCND1	0.428	1.71E-08	ARHGEF16	0.360	3.83E-07	C5	-0.448	4.78E-06	TMEM200B	0.271	1.22E-04	NCAM2	-0.350	3.73E-02
CA13	-0.342	8.89E-16	BIRC3	0.631	2.90E-12	TXNRD1	0.344	5.59E-10	TIMD4	-0.887	1.72E-08	KAT6A	-0.270	3.84E-07	NID1	-0.297	4.86E-06	RDH11	0.276	1.24E-04	TBL1Y	0.369	3.85E-02
ADGRA3	-0.531	9.09E-16	DTL	0.516	2.90E-12	MCC	-0.397	5.71E-10	DGKE	-0.284	1.72E-08	FLNA	0.511	3.86E-07	RBBP9	0.307	4.88E-06	SHANK3	-0.348	1.25E-04	HLA-DQA2	0.309	4.22E-02
ARSK	-0.427	9.38E-16	CYB561A3	0.353	2.94E-12	HS3ST3A1	-0.398	5.71E-10	CTBS	-0.368	1.72E-08	ADD3	-0.333	3.97E-07	SLC7A5	0.278	4.89E-06	SNTG1	-0.278	1.28E-04	SERTM2	-0.341	4.52E-02
SS18	-0.292	9.52E-16	SCN9A	-0.598	2.97E-12	GCFC2	-0.359	5.71E-10	MED13	-0.404	1.72E-08	PGP	0.314	3.99E-07	BCL2A1	0.291	4.97E-06	ANKRD36C	0.373	1.30E-04	NR1D1	-0.268	4.66E-02
GAREM1	-0.479	9.80E-16	CEP120	-0.325	3.05E-12	AP5B1	0.304	5.98E-10	ABCA8	-0.458	1.77E-08	SLC6A8	0.305	3.99E-07	RNF125	-0.325	5.22E-06	MICU3	-0.334	1.33E-04	A2M	0.311	4.87E-02
STMN2	0.939	9.80E-16	FAIM	0.325	3.05E-12	BNIP3	-0.355	6.06E-10	GLRX5	-0.326	1.77E-08	TMEM45B	0.650	4.02E-07	BTG2	0.292	5.25E-06	CHIT1	0.367	1.34E-04	CHRD	-0.284	4.92E-02
TUT7	-0.625	9.80E-16	CLSPN	0.423	3.10E-12	ZNF710	0.449	6.38E-10	CENPM	0.308	1.82E-08	GRAMD2B	-0.315	4.06E-07	CCNA2	0.268	5.36E-06	MT1B	-0.372	1.39E-04			

Supplementary Table 5: Enriched pathways in NAFL vs Control

	ID	Description	setSize	enrichmentScore	NES	pvalue	p.adjust
1	GO:0007005	mitochondrion organization	439	0.330278549	1.859170854	2.73E-10	1.31E-06
2	GO:0048667	cell morphogenesis involved in neuron differentiation	458	-0.370570017	-1.84698388	4.50E-10	1.31E-06
3	GO:0006119	oxidative phosphorylation	113	0.504309618	2.366615344	1.06E-09	1.98E-06
4	GO:0009060	aerobic respiration	158	0.436834595	2.185727971	1.49E-09	1.98E-06
5	GO:0042254	ribosome biogenesis	230	0.388085269	2.041902675	1.70E-09	1.98E-06
6	GO:0007610	behavior	463	-0.363741471	-1.8138472	3.61E-09	3.52E-06
7	GO:0048638	regulation of developmental growth	260	-0.413334519	-1.97770412	5.01E-09	4.18E-06
8	GO:0042773	ATP synthesis coupled electron transport	73	0.559047414	2.42939154	1.23E-08	7.99E-06
9	GO:0042775	mitochondrial ATP synthesis coupled electron transport	73	0.559047414	2.42939154	1.23E-08	7.99E-06
10	GO:0019646	aerobic electron transport chain	65	0.562938552	2.402119902	3.65E-08	2.07E-05
11	GO:0006364	rRNA processing	168	0.40983457	2.067607889	3.90E-08	2.07E-05
12	GO:0050678	regulation of epithelial cell proliferation	275	-0.394252914	-1.89244513	8.16E-08	3.85E-05
13	GO:0050673	epithelial cell proliferation	323	-0.380738919	-1.84926647	8.57E-08	3.85E-05
14	GO:0022900	electron transport chain	144	0.428039774	2.101440465	1.14E-07	4.77E-05
15	GO:0034329	cell junction assembly	362	-0.363758014	-1.77961123	1.81E-07	7.05E-05
16	GO:0046034	ATP metabolic process	225	0.363069997	1.899953194	2.00E-07	7.30E-05
17	GO:0008015	blood circulation	425	-0.346813921	-1.71954234	2.37E-07	8.14E-05
18	GO:0042255	ribosome assembly	51	0.582652228	2.382172699	3.52E-07	0.000114126
19	GO:0090150	establishment of protein localization to membrane	209	0.365299976	1.869195634	3.99E-07	0.000116845
20	GO:0022613	ribonucleoprotein complex biogenesis	318	0.323065222	1.782940643	4.00E-07	0.000116845
21	GO:0003013	circulatory system process	500	-0.330797224	-1.66147208	5.41E-07	0.000150429
22	GO:0006457	protein folding	165	0.384273325	1.94540052	6.52E-07	0.000165552
23	GO:0007409	axonogenesis	350	-0.358822234	-1.75600315	6.25E-07	0.000165552
24	GO:0070585	protein localization to mitochondrion	105	0.450113342	2.095438247	7.56E-07	0.000176714
25	GO:0061564	axon development	390	-0.346437766	-1.70805262	7.45E-07	0.000176714
26	GO:0007156	homophilic cell adhesion via plasma membrane adhesion molecules	143	-0.448207192	-1.98489447	8.33E-07	0.000187252
27	GO:0098742	cell-cell adhesion via plasma-membrane adhesion molecules	239	-0.391929168	-1.85679916	1.02E-06	0.0002201
28	GO:1903522	regulation of blood circulation	211	-0.403132245	-1.88794737	1.21E-06	0.000247346
29	GO:0071417	cellular response to organonitrogen compound	497	-0.326135489	-1.63705107	1.23E-06	0.000247346
30	GO:0033108	mitochondrial respiratory chain complex assembly	81	0.489974622	2.20759771	1.52E-06	0.000295455
31	GO:0098727	maintenance of cell number	115	-0.465359129	-1.99732315	1.78E-06	0.000335889
32	GO:0007416	synapse assembly	151	-0.44249592	-1.96941955	2.06E-06	0.000353276
33	GO:0016072	rRNA metabolic process	201	0.364816939	1.866508685	2.01E-06	0.000353276
34	GO:0007423	sensory organ development	414	-0.334073978	-1.65240464	1.98E-06	0.000353276
35	GO:0046620	regulation of organ growth	73	-0.527027227	-2.10999729	2.39E-06	0.000389231
36	GO:0045333	cellular respiration	196	0.361427382	1.848623897	2.53E-06	0.000389231
37	GO:0007507	heart development	484	-0.32914329	-1.64804448	2.40E-06	0.000389231
38	GO:0006091	generation of precursor metabolites and energy	428	0.283811712	1.603360146	2.53E-06	0.000389231
39	GO:0060419	heart growth	69	-0.539729967	-2.12227959	2.85E-06	0.000427253
40	GO:0006839	mitochondrial transport	147	0.38874744	1.908503148	3.01E-06	0.000440274
41	GO:0007389	pattern specification process	309	-0.359712866	-1.74286522	3.48E-06	0.000495492
42	GO:0019827	stem cell population maintenance	113	-0.460000453	-1.96177357	3.98E-06	0.000551254
43	GO:0003205	cardiac chamber development	141	-0.428009515	-1.88384736	4.06E-06	0.000551254
44	GO:0051205	protein insertion into membrane	52	0.547920872	2.235636405	4.31E-06	0.000565346
45	GO:0003170	heart valve development	59	-0.550298539	-2.08751293	4.45E-06	0.000565346
46	GO:0003279	cardiac septum development	87	-0.492792815	-2.01854691	4.37E-06	0.000565346
47	GO:0022904	respiratory electron transport chain	92	0.45484097	2.069015619	5.41E-06	0.000672939
48	GO:0051960	regulation of nervous system development	353	-0.338738074	-1.65827805	5.97E-06	0.000726209
49	GO:0010631	epithelial cell migration	270	-0.362918036	-1.73792305	6.60E-06	0.000787457
50	GO:0007178	transmembrane receptor protein serine/threonine kinase signaling pathway	297	-0.35517491	-1.72036894	6.89E-06	0.000805464
51	GO:1901342	regulation of vasculature development	255	-0.367015006	-1.74899995	7.14E-06	0.000818056
52	GO:0044057	regulation of system process	452	-0.322266421	-1.60325293	8.09E-06	0.000908357
53	GO:0072655	establishment of protein localization to mitochondrion	102	0.431438222	2.008411245	8.78E-06	0.000932772
54	GO:1901888	regulation of cell junction assembly	178	-0.402969443	-1.83764056	8.71E-06	0.000932772
55	GO:0001667	ameboidal-type cell migration	370	-0.337432195	-1.65525418	8.75E-06	0.000932772
56	GO:0001525	angiogenesis	465	-0.314720873	-1.57049801	9.41E-06	0.000981488
57	GO:0090130	tissue migration	275	-0.358949903	-1.72298789	9.58E-06	0.000982132

Supplementary Table 6: Enriched pathways in Borderline vs Control

	ID	Description	setSize	enrichmentScore	NES	pvalue	p.adjust
1	GO:0002495	antigen processing and presentation of peptide antigen via MHC class II	31	0.76630162	2.62335252	1.2E-09	1.26564E-06
2	GO:0002504	antigen processing and presentation of peptide or polysaccharide antigen via M	33	0.748299614	2.580592155	1.30E-09	1.27E-06
3	GO:0019882	antigen processing and presentation	95	0.558599627	2.399501121	9.95E-10	1.27E-06
4	GO:0030198	extracellular matrix organization	268	0.402234792	2.020076632	1.28E-09	1.27E-06
5	GO:0045229	external encapsulating structure organization	271	0.401619646	2.016946279	9.18E-10	1.27E-06
6	GO:0043062	extracellular structure organization	269	0.401322181	2.016907399	1.09E-09	1.27E-06
7	GO:0002478	antigen processing and presentation of exogenous peptide antigen	36	0.726418023	2.560023481	4.17E-09	3.05E-06
8	GO:0019884	antigen processing and presentation of exogenous antigen	44	0.688558023	2.538907615	3.77E-09	3.05E-06
9	GO:0002396	MHC protein complex assembly	18	0.857587543	2.525806561	6.13E-09	3.42E-06
10	GO:0002501	peptide antigen assembly with MHC protein complex	18	0.857587543	2.525806561	6.13E-09	3.42E-06
11	GO:0048002	antigen processing and presentation of peptide antigen	58	0.636206213	2.524785812	6.44E-09	3.42E-06
12	GO:0019886	antigen processing and presentation of exogenous peptide antigen via MHC cla	28	0.754776054	2.518267885	3.59E-08	1.75E-05
13	GO:0050900	leukocyte migration	321	0.36597969	1.86502515	1.10E-07	4.92E-05
14	GO:0002399	MHC class II protein complex assembly	14	0.869565508	2.361102943	1.77E-07	6.89E-05
15	GO:0002503	peptide antigen assembly with MHC class II protein complex	14	0.869565508	2.361102943	1.77E-07	6.89E-05
16	GO:0001959	regulation of cytokine-mediated signaling pathway	129	0.455652952	2.042874364	3.35E-07	1.22E-04
17	GO:0061687	detoxification of inorganic compound	14	-0.859678463	-2.3174345	3.64E-07	1.25E-04
18	GO:0010273	detoxification of copper ion	11	-0.895473536	-2.26587586	4.23E-07	1.30E-04
19	GO:1990169	stress response to copper ion	11	-0.895473536	-2.26587586	4.23E-07	0.000130145
20	GO:0032963	collagen metabolic process	78	0.530278195	2.197132065	6.96E-07	0.000203336
21	GO:0042178	xenobiotic catabolic process	24	-0.742504857	-2.30340667	7.74E-07	0.00021535
22	GO:0071674	mononuclear cell migration	168	0.413409778	1.927939867	1.00E-06	0.000266614
23	GO:0071294	cellular response to zinc ion	17	-0.802523203	-2.2598256	1.17E-06	0.000295921
24	GO:0097190	apoptotic signaling pathway	493	0.300253941	1.58377462	1.28E-06	0.000310678
25	GO:0002687	positive regulation of leukocyte migration	125	0.454022681	2.034280223	1.40E-06	0.000327766
26	GO:0022411	cellular component disassembly	389	0.319869364	1.657006724	1.59E-06	0.000356488
27	GO:0043903	regulation of biological process involved in symbiotic interaction	45	0.61168729	2.253108781	2.10E-06	0.000453582
28	GO:0007249	I-kappaB kinase/NF-kappaB signaling	254	0.367367034	1.822975936	2.84E-06	0.000592459
29	GO:0006636	unsaturated fatty acid biosynthetic process	44	0.603572624	2.225542483	3.51E-06	0.000700818
30	GO:0071357	cellular response to type I interferon	46	0.597788632	2.215920743	3.60E-06	0.000700818

Supplementary Table 7: Enriched pathways in NASH vs Control

	ID	Description	setSize	enrichmentScore	NES	pvalue	p.adjust
1	GO:0045229	external encapsulating structure organization	271	0.493207548	2.316296891	1.189E-14	6.94556E-11
2	GO:0030198	extracellular matrix organization	268	0.49457252	2.319928427	2.92E-14	8.54E-11
3	GO:0043062	extracellular structure organization	269	0.493521969	2.314700503	4.46E-14	8.68E-11
4	GO:0016054	organic acid catabolic process	226	-0.481771911	-2.1932296	2.35E-12	3.43E-09
5	GO:0046395	carboxylic acid catabolic process	223	-0.484507575	-2.20122868	4.23E-12	4.94E-09
6	GO:0050900	leukocyte migration	321	0.431197251	2.06160722	8.31E-11	8.09E-08
7	GO:0010043	response to zinc ion	40	-0.739049882	-2.51241694	2.08E-10	1.74E-07
8	GO:0002687	positive regulation of leukocyte migration	125	0.551800792	2.322846942	4.45E-10	3.25E-07
9	GO:1901605	alpha-amino acid metabolic process	178	-0.479332974	-2.13235467	1.16E-09	7.53E-07
10	GO:0019882	antigen processing and presentation	95	0.59125349	2.384116522	1.63E-09	9.50E-07
11	GO:0009063	cellular amino acid catabolic process	105	-0.55678854	-2.29037035	2.58E-09	1.37E-06
12	GO:0071677	positive regulation of mononuclear cell migration	57	0.654637897	2.442456305	3.43E-09	1.67E-06
13	GO:0071294	cellular response to zinc ion	17	-0.874808574	-2.4414497	5.47E-09	2.46E-06
14	GO:0002504	antigen processing and presentation of peptide or polysaccharide antigen via M	33	0.760752052	2.470989302	1.27E-08	4.94E-06
15	GO:0071674	mononuclear cell migration	168	0.484319412	2.119705099	1.21E-08	4.94E-06
16	GO:0002685	regulation of leukocyte migration	186	0.468920955	2.085457335	1.65E-08	6.04E-06
17	GO:0030199	collagen fibril organization	57	0.635693487	2.371774643	1.99E-08	6.85E-06
18	GO:2000147	positive regulation of cell motility	478	0.355825603	1.75042844	2.33E-08	7.58E-06
19	GO:0002495	antigen processing and presentation of peptide antigen via MHC class II	31	0.759027809	2.454050311	3.47E-08	9.88824E-06
20	GO:0030595	leukocyte chemotaxis	201	0.441981559	1.992091702	3.53E-08	9.88824E-06
21	GO:0050867	positive regulation of cell activation	318	0.393632815	1.882650707	3.56E-08	9.88824E-06
22	GO:0002696	positive regulation of leukocyte activation	306	0.393079322	1.873865001	3.72E-08	9.88824E-06
23	GO:0051272	positive regulation of cellular component movement	487	0.35527306	1.756486271	3.98E-08	9.94179E-06
24	GO:0030335	positive regulation of cell migration	466	0.353751197	1.739743447	4.08E-08	9.94179E-06
25	GO:0002478	antigen processing and presentation of exogenous peptide antigen	36	0.721152171	2.398445848	4.32E-08	1.00997E-05
26	GO:0040017	positive regulation of locomotion	491	0.353554368	1.751346659	5.04E-08	1.13299E-05
27	GO:0032963	collagen metabolic process	78	0.587437154	2.29050591	6.90E-08	1.49322E-05
28	GO:0071675	regulation of mononuclear cell migration	99	0.55541829	2.250601294	7.39E-08	1.5416E-05
29	GO:0048002	antigen processing and presentation of peptide antigen	58	0.624806882	2.335634343	8.38E-08	1.6882E-05
30	GO:0061687	detoxification of inorganic compound	14	-0.877288522	-2.33573502	9.82E-08	1.79249E-05
31	GO:0010273	detoxification of copper ion	11	-0.913138545	-2.27488746	9.79E-08	1.79249E-05
32	GO:1990169	stress response to copper ion	11	-0.913138545	-2.27488746	9.79E-08	1.79249E-05
33	GO:0042178	xenobiotic catabolic process	24	-0.777658629	-2.36073257	1.48E-07	2.53864E-05
34	GO:0044282	small molecule catabolic process	338	-0.378907355	-1.81724718	1.46E-07	2.53864E-05
35	GO:0097529	myeloid leukocyte migration	194	0.434743481	1.951197143	1.75E-07	2.92023E-05
36	GO:0045785	positive regulation of cell adhesion	397	0.359522452	1.74332453	2.28E-07	3.69584E-05
37	GO:1901606	alpha-amino acid catabolic process	86	-0.538553838	-2.1471829	2.36E-07	3.71874E-05
38	GO:0007229	integrin-mediated signaling pathway	101	0.523657425	2.125983027	2.44E-07	3.7561E-05
39	GO:0019884	antigen processing and presentation of exogenous antigen	44	0.681760099	2.365131457	2.59E-07	3.88521E-05
40	GO:0019886	antigen processing and presentation of exogenous peptide antigen via MHC cla	28	0.742989995	2.363753885	5.94E-07	8.68263E-05
41	GO:0006520	cellular amino acid metabolic process	242	-0.401724723	-1.84379797	6.29E-07	8.96232E-05
42	GO:0060326	cell chemotaxis	258	0.396921829	1.8456835	6.91E-07	9.60888E-05
43	GO:0002399	MHC class II protein complex assembly	14	0.864904513	2.247216857	7.85E-07	0.000101966
44	GO:0002503	peptide antigen assembly with MHC class II protein complex	14	0.864904513	2.247216857	7.85E-07	0.000101966
45	GO:0048144	fibroblast proliferation	74	0.566235447	2.199340974	7.72E-07	0.000101966
46	GO:0002690	positive regulation of leukocyte chemotaxis	81	0.543277775	2.12493955	1.29E-06	0.000161353
47	GO:0071621	granulocyte chemotaxis	111	0.50180423	2.069760458	1.30E-06	0.000161353
48	GO:0097530	granulocyte migration	131	0.473884278	2.008572198	1.54E-06	0.000186929
49	GO:0009611	response to wounding	485	0.330858719	1.632725882	1.75E-06	0.000209013
50	GO:0035987	endodermal cell differentiation	39	0.663053517	2.253497039	2.16E-06	0.000252342
51	GO:0097190	apoptotic signaling pathway	493	0.330778632	1.638839388	2.20E-06	0.000252342
52	GO:0001959	regulation of cytokine-mediated signaling pathway	129	0.471679587	2.000724307	2.26E-06	0.000253841
53	GO:1903039	positive regulation of leukocyte cell-cell adhesion	222	0.398061789	1.817651654	2.60E-06	0.000286492
54	GO:0006636	unsaturated fatty acid biosynthetic process	44	0.644992599	2.2375793	3.42E-06	0.000369488
55	GO:0002396	MHC protein complex assembly	18	0.79861887	2.233992511	3.77E-06	0.000390086
56	GO:0002501	peptide antigen assembly with MHC protein complex	18	0.79861887	2.233992511	3.77E-06	0.000390086
57	GO:0043122	regulation of I-kappaB kinase/NF-kappaB signaling	224	0.38754774	1.76837752	3.81E-06	0.000390086
58	GO:0044262	cellular carbohydrate metabolic process	245	-0.37788285	-1.73596856	4.42E-06	0.000437828
59	GO:0032103	positive regulation of response to external stimulus	362	0.346903865	1.672146353	4.40E-06	0.000437828
60	GO:0085029	extracellular matrix assembly	43	0.627219972	2.174172285	5.10E-06	0.000497006
61	GO:0001706	endoderm formation	46	0.620051059	2.173514619	5.35E-06	0.000512831
62	GO:0043903	regulation of biological process involved in symbiotic interaction	45	0.616188086	2.146726005	5.74E-06	0.000540679
63	GO:0060759	regulation of response to cytokine stimulus	139	0.444251969	1.891751269	6.60E-06	0.000612413
64	GO:0043068	positive regulation of programmed cell death	437	0.328839581	1.608836606	6.77E-06	0.000618401
65	GO:0042060	wound healing	378	0.340041527	1.645227937	7.66E-06	0.000688848
66	GO:0006805	xenobiotic metabolic process	91	-0.502575555	-2.02929625	8.02E-06	0.000699153
67	GO:0072329	monocarboxylic acid catabolic process	116	-0.460443966	-1.90913982	7.98E-06	0.000699153
68	GO:0050870	positive regulation of T cell activation	201	0.391808399	1.765952095	9.27E-06	0.000796301
69	GO:0030574	collagen catabolic process	32	0.684112288	2.205779383	9.92E-06	0.000827438
70	GO:0006986	response to unfolded protein	117	0.463804714	1.937853599	9.98E-06	0.000827438
71	GO:0007249	I-kappaB kinase/NF-kappaB signaling	254	0.382621894	1.778980147	1.01E-05	0.000827438
72	GO:0048146	positive regulation of fibroblast proliferation	45	0.605675315	2.11010076	1.10E-05	0.000889271
73	GO:0002694	regulation of leukocyte activation	481	0.318311784	1.570893481	1.11E-05	0.000891861
74	GO:0051251	positive regulation of lymphocyte activation	267	0.373046996	1.749199205	1.17E-05	0.000923537
75	GO:0048145	regulation of fibroblast proliferation	72	0.542244717	2.095087792	1.19E-05	0.000930693
76	GO:0030593	neutrophil chemotaxis	94	0.488399069	1.965762146	1.24E-05	0.000941042
77	GO:0002440	production of molecular mediator of immune response	183	0.413745366	1.832314512	1.24E-05	0.000941042

Supplementary Table 8: Differentially abundant hepatic microbes

Comparison	Name	Log2FC	FDR
NAFL vs Control	Cloacibacterium normanense	-1.303	1.75E-11
	Fulvia fulva	2.730	7.27E-10
	Streptomyces sp. T12	2.971	7.27E-10
	Cloacibacterium caeni	-1.345	9.04E-08
	Plantactinospora sp. BC1	-2.694	1.20E-07
	Plantactinospora sp. BB1	-2.296	2.43E-07
	Methyloversatilis sp. RAC08	-1.217	2.43E-07
	Stenotrophomonas maltophilia	2.632	3.78E-07
	Corynebacterium kefirresidentii	1.084	2.47E-05
	Pseudomonas sp. CIP-10	1.742	4.96E-05
	Cutibacterium acnes	-1.514	1.11E-04
	Acinetobacter baumannii	1.548	5.43E-04
	Moraxella osloensis	1.826	6.08E-04
	Corynebacterium tuberculostearicum	1.184	2.88E-03
	Escherichia coli	-1.481	4.79E-03
	Ralstonia insidiosa	-1.279	7.85E-03
	Colletotrichum lupini	-1.088	1.65E-02
	Puccinia triticina	1.162	3.53E-02
Borderline vs Control	Cloacibacterium normanense	-1.013	9.75E-07
	Streptomyces sp. T12	2.142	3.98E-05
	Methyloversatilis sp. RAC08	-1.012	7.16E-05
	Stenotrophomonas maltophilia	2.028	3.08E-04
	Plantactinospora sp. BB1	-1.726	3.08E-04
	Plantactinospora sp. BC1	-1.920	4.33E-04
	Malassezia vespertilionis	-1.568	5.54E-04
	Ralstonia insidiosa	-1.573	1.70E-03
	Fulvia fulva	1.490	1.70E-03
	Staphylococcus epidermidis	-1.096	1.57E-02
	Acinetobacter baumannii	1.025	3.34E-02
	Bacillus velezensis	1.416	4.09E-02
NASH vs Control	Dolosigranulum pigrum	1.316	7.38E-12
	Streptomyces sp. T12	2.588	7.03E-09
	Cloacibacterium normanense	-1.016	7.03E-09
	Methyloversatilis sp. RAC08	-1.256	7.14E-09
	Cloacibacterium caeni	-1.076	2.80E-06
	Malassezia vespertilionis	-1.719	1.12E-05
	Plantactinospora sp. BB1	-1.767	1.67E-05
	Cutibacterium acnes	-1.363	1.30E-04
	Stenotrophomonas maltophilia	1.809	1.61E-04
	Plantactinospora sp. BC1	-1.737	2.01E-04
	Staphylococcus epidermidis	-1.413	2.12E-04
	Acinetobacter johnsonii	1.089	3.33E-04
	Acinetobacter baumannii	1.387	5.38E-04
	Rothia mucilaginosa	1.068	5.38E-04
	Ralstonia insidiosa	-1.270	2.69E-03
	Bacillus velezensis	1.645	4.60E-03
	Escherichia coli	-1.048	2.79E-02

Supplementary Table 9. Hepatic microbiota in independent bulk RNA-seq validation data

Eukaryota	Leptospira	Nocardiaceae	Anatilimnocola aggregata
Bacteria	Turneriella	Mycobacteriaceae	Lacipirellula parvula
Archaea	Paludibaculum	Dietziaceae	Thermogutta terrifontis
Viruses	Luteitalea	Lawsonellaceae	Telmatocola sphagniphila
Fungi	Geothrix	Propionibacteriaceae	Gemmata obscuriglobus
Orthornavirae	Chloracidobacterium	Nocardioidaceae	Tuwongella immobilis
Heunggongvirae	Fusobacterium	Candidatus Nanopelagicaceae	Frigoriglobus tundricola
Bamfordvirae	Leptotrichia	Actinomycetaceae	Fuerstiella marisgermanici
Ascomycota	Chelatococcus	Micromonosporaceae	Planctomyces sp. SH-PL14
Basidiomycota	Hankyongella	Pseudonocardiaceae	Polystyrenella longa
Pseudomonadota	Porphyrobacter	Bifidobacteriaceae	Rubinisphaera brasiliensis
Bacteroidota	Ferribacterium	Nocardiopsaceae	Singulisphaera acidiphila
Ignavibacteriota	Luteibacter	Geodermatophilaceae	Isosphaera pallida
Rhodothermota	Proteiniphilum	Nakamurellaceae	Humisphaera borealis
Gemmatimonadota	Exiguobacterium	Jatrophihabitantaceae	Verrucomicrobium spinosum
Actinomycetota	Faecalibacterium	Iamiaceae	Roseimicrobium sp. ORNL1
Bacillota	Mucisphaera	Ilumatobacteraceae	Sulfuriroseicoccus oceanibius
Cyanobacteriota	Caldithrix	Atopobiaceae	Fontisphaera persica
Mycoplasmatota	Pulveribacter	Eggerthellaceae	Lacunisphaera limnophila
Chloroflexota	Paenacidovorax	Rubrobacteraceae	Nibricoccus aquaticus
Deinococcota	Chitiniphilus	Bacillaceae	Opitutus terrae
Armatimonadota	Methyloradius	Staphylococcaceae	Horticoccus luteus
Planctomycetota	Nitrogeniibacter	Paenibacillaceae	Ruficoccus sp. ZRK36
Verrucomicrobiota	Quatrionicoccus	Planococcaceae	Candidatus Xiphinematobacter sp. Idaho Grape
Chlamydiota	Pectobacterium	Streptococcaceae	Parachlamydia acanthamoebae
Kiritimatiellota	Stutzerimonas	Lactobacillaceae	Candidatus Protochlamydia naegleriophila
Campylobacterota	Nitrobacter	Enterococcaceae	Waddlia chondrophila
Myxococcota	Rhodomicrobium	Carnobacteriaceae	Tichowtungia aerotolerans
Bdellovibrionota	Tardibacter	Aerococcaceae	Sorangium cellulosum
Thermodesulfobacteriota	Neotabrizicola	Clostridiaceae	Polyangium aurulentum
Candidatus Saccharibacteria	Rhodovastum	Oscillospiraceae	Chondromyces crocatus
Candidatus Absconditabacteria	Elioraea	Lachnospiraceae	Labilithrix luteola
Spirochaetota	Candidatus Viadribacter	Veillonellaceae	Sandaracinus amylolyticus
Acidobacteriota	Ornithobacterium	Peptoniphilaceae	Nannocystis poenicansa
Nitrospirota	Rhodocytophaga	Erysipelotrichaceae	Pseudobdellovibrio exovorus
Thermotogota	Pseudocnuella	Microcoleaceae	Bdellovibrio sp. NC01
Fusobacteriota	Segatella	Oscillatoriaceae	Bdellovibrio sp. 22V
Elusimicrobiota	Hoylesella	Gomontiellaceae	Bdellovibrio bacteriovorus
Synergistota	Cryobacterium	Chroococcaceae	Bdellovibrio reynosensis
Aquificota	Protaetiibacter	Synechococcaceae	Micavibrio aeruginosavorus
Euryarchaeota	Dermabacter	Prochlorococcaceae	Bacteriovorax stolpii
Uroviricota	Phycicoccus	Merismopediaceae	Pigmentibacter sp. JX0631
Peploviricota	Kytococcus	Nostocaceae	Candidatus Saccharimonas aalborgensis
Nitrososphaerota	Arachnia	Calotrichaceae	Candidatus Mycosynbacter amalyticus
Chrysiogenota	Jeotgalicoccus	Pseudanabaenaceae	Candidatus Chromulinivorax destructor
Chlorobiota	Abyssicoccus	Leptolyngbyaceae	Candidatus Babela massiliensis
Deferribacterota	Aerococcus	Gloeobacteraceae	Candidatus Absconditicoccus praedator
Saccharomycetes	Fastidiosipila	Mycoplasmataceae	Entomospira culicis
Sordariomycetes	Subdoligranulum	Spiroplasmataceae	Turneriella parva
Leotiomycetes	Anaeropeptidivorans	Acholeplasmataceae	Paludibaculum fermentans
Eurotiomycetes	Anaerostipes	Anaerolineaceae	Geothrix sp. 21YS21S-2
Malasseziomycetes	Mediterraneibacter	Caldilineaceae	Methylobacterium sp. FF17
Pucciniomycetes	Roseburia	Tepidiformaceae	Sphingopyxis sp. MG
Alphaproteobacteria	Stomatobaculum	Thermaceae	Sphingomonas sp. SUN019
Betaproteobacteria	Lachnoanaerobaculum	Deinococcaceae	Sphingomonas cannabina
Gammaproteobacteria	Agathobacter	Fimbriimonadaceae	Sphingomonas sanxanigenens
Hydrogenophilia	Dorea	Pirellulaceae	Novosphingobium pentaromativorans
Flavobacteriia	Thermoanaerobacterium	Lacipirellulaceae	Novosphingobium resinovorum
Cytophagia	Megamonas	Thermoguttaceae	Hankyongella ginsenosidimutans
Chitinophagia	Pelosinus	Gemmataceae	Asticcacaulis excentricus
Bacteroidia	Parvimonas	Planctomycetaceae	Polynucleobacter sp. KF022
Sphingobacteriia	Planktothrix	Isosphaeraceae	Ferribacterium limneticum
Saprospiria	Gloeocapsa	Phycisphaeraceae	Legionella waltersii
Ignavibacteria	Capsulimonas	Tepidisphaeraceae	Corynebacterium coyleae
Rhodothermia	Symmachiella	Verrucomicrobiaceae	Mycolicibacterium aubagnense
Gemmatimonadetes	Lentisphaera	Akkermansiaceae	Iamia sp. SCSIO 61187
Actinomycetes	Leptospirillum	Fontisphaeraceae	Geobacillus stearothermophilus
Acidimicrobiia	Fulvia	Opitutaceae	Anaerococcus sp. Marseille-Q7828
Coriobacteriia	Rhizoctonia	Cerasicoccaceae	Mucisphaera calidilacus
Rubrobacteria	Pahexavirus	Methylacidiphilaceae	Candidatus Sulfuricurvum sp. RIFRC-1
Thermoleophilia	Sphingosinithalassobacter	Parachlamydiaceae	Acidovorax radicis
Bacilli	Roseococcus	Rhabdochlamydiaceae	Rhodoferax saidenbachensis
Clostridia	Clavibacter	Waddliaceae	Pulveribacter suum
Negativicutes	Collinsella	Chlamydiaceae	Paenacidovorax monticola
Tissierellia	Ruminococcus	Campylobacteraceae	Roseateles sp. BIM B-1768
Erysipelotrichia	Neochlamydia	Arcobacteraceae	Janthinobacterium tructae
Cyanophyceae	Haematobacter	Helicobacteraceae	Chitiniphilus sp. CD1
Mollicutes	Pseudoroseomonas	Sulfurimonadaceae	Methyloradius palustris
Anaerolineae	Candidatus Phycorickettsia	Sulfurovaceae	Quatrionicoccus australiensis
Caldilineae	Flagellatimonas	Polyangiaceae	Moraxella nonliquefaciens
Chloroflexia	Tannerella	Labilitrichaceae	Moraxella catarrhalis
Tepidiformia	Tardiphaga	Sandaracinaceae	Legionella antarctica
Deinococci	Ezakiella	Kofleriaceae	Bradyrhizobium sp. 200
Fimbriimonadia	Winkia	Nannocystaceae	Bradyrhizobium sp. CIAT3101
Planctomycetia	Anaerobutyricum	Anaeromyxobacteraceae	Bradyrhizobium sp. CB3481
Phycisphaerae	Fenollaria	Archangiaceae	Bradyrhizobium sp. CCBAU 51765
Verrucomicrobiae	Immundisolibacter	Myxococcaceae	Bradyrhizobium sp. CB82
Opitutae	Niveibacterium	Pseudobdellovibrionaceae	Bradyrhizobium sp. CB1650
Spartobacteria	Varunaivibrio	Halobacteriovoraceae	Bradyrhizobium sp. CB2312
Methylacidiphilae	Paludisphaera	Bacteriovoracaceae	Bradyrhizobium sp. 195
Chlamydiia	Succinivibrio	Silvanigrellaceae	Bradyrhizobium sp. 192
Epsilonproteobacteria	Stella	Desulfovibrionaceae	Bradyrhizobium sediminis
Myxococcia	Selenomonas	Desulfomicrobiaceae	Bradyrhizobium elkanii
Bdellovibrionia	Blastomonas	Geobacteraceae	Bradyrhizobium quebecense
Bacteriovoracia	Ligilactobacillus	Desulfobulbaceae	Nitrobacter hamburgensis
Oligoflexia	Truepera	Candidatus Nanosynbacteraceae	Shinella zoogloeoides
Desulfovibrionia	Nocardioides	Candidatus Saccharimonadaceae	Methylobacterium sp. WL1
Desulfobacteria	Alistipes	Candidatus Chromulinivoraceae	Methylobacterium organophilum
Desulfuromonadia	Lichenihabitans	Candidatus Babeliaceae	Methylorubrum sp. B1-46
Desulfobulbia	Candidatus Fokinia	Candidatus Absconditicoccaceae	Hyphomicrobium sp. DMF-1
Candidatus Saccharimonadia	Glutamicibacter	Spirochaetaceae	Sphingopyxis sp. GC21
Candidatus Babeliae	Aquisphaera	Treponemataceae	Sphingomonas sp.
Spirochaetia	Desulfurispirillum	Leptospiraceae	Sphingomonas sp. NIBR02145
Terriglobia	Noviherbaspirillum	Acidobacteriaceae	Sphingomonas sp. CL5.1
Vicinamibacteria	Eleftheria	Bryobacteraceae	Sphingomonas sp. R1

Holophagae	Caenibius	Vicinamibacteraceae	Sphingomonas sp. AP4-R1
Blastocatellia	Methanothrix	Holophagaceae	Sphingomonas donggukensis
Nitrospiria	Tsuneonella	Nitrospiraceae	Sphingomonas taxi
Thermotogae	Peptostreptococcus	Fusobacteriaceae	Sphingomonas aliaeris
Fusobacteriia	Thomasclavelia	Leptotrichiaceae	Sphingomonas suaedae
Deltaproteobacteria	Lautropia	Haloferacaceae	Sphingomonas radiodurans
Synergistia	Usitatibacter	Methanobacteriaceae	Sphingomonas aerolata
Aquificae	Thiothrix	Baculoviridae	Sphingomonas alpina
Halobacteria	Aneurinibacillus	Orthoherpesviridae	Sphingomonas naphthae
Methanomicrobia	Ethanoligenens	Chelatococcaceae	Sphingomonas bisphenolicum
Methanobacteria	Micropruina	Syntrophomonadaceae	Sphingomonas glaciei
Naldaviricetes	Frigoribacterium	Kytococcaceae	Sphingomonas panacis
Caudoviricetes	Mageeibacillus	Streptomycetaceae	Sphingobium sp. RAC03
Herviviricetes	Nitrospina	Thermoanaerobacteraceae	Sphingobium sp. TKS
Armatimonadia	Sulfitobacter	Selenomonadaceae	Sphingobium sp. JS3065
Lentisphaeria	Homoserinibacter	Sporomusaceae	Sphingobium sp. PAMC28499
Dothideomycetes	Rhodoplanes	Capsulimonadaceae	Sphingobium sp. CFD-1
Agaricomycetes	Methylocystis	Mycosphaerellaceae	Novosphingobium sp. Gsoil 351
Chrysiogenetes	Candidatus Hydrogenosomobacter	Ceratobasidiaceae	Novosphingobium sp. ES2-1
Chlorobiia	Phycisphaera	Coriobacteriaceae	Novosphingobium sp. PP1Y
Deferribacteres	Aminobacter	Sulfuricellaceae	Novosphingobium sp. P6W
Ustilaginomycetes	Chenggangzhangella	Listeriaceae	Novosphingobium ginsenosidimutans
Schizosaccharomycetes	Candidatus Endolissoclinum	Eubacteriales Family XIII. Incertae Sedis	Novosphingobium aromaticivorans
Saccharomycetales	Thiomonas	Succinivibrionaceae	Rhizorhabdus dicambivorans
Hypocreales	Poriferisphaera	Stellaceae	Rhizorhabdus wittichii
Glomerellales	Candidatus Nucleicultrix	Peptostreptococcaceae	Tardibacter chloracetimidivorans
Sordariales	Defluviicoccus	Trueperaceae	Croceicoccus sp. YJ47
Helotiales	Paracidovorax	Rikenellaceae	Fuscovulum blasticum
Eurotiales	Agrococcus	Parvibaculaceae	Neotabrizicola shimadae
Malasseziales	Skermanella	Chrysiogenaceae	Rhodovastum atsumiense
Pucciniales	Candidatus Promineifilum	Coprobacillaceae	Elioraea tepida
Hyphomicrobiales	Acidobacterium	Bartonellaceae	Caulobacter sp. FWC26
Sphingomonadales	Parvibaculum	Beijerinckiaceae	Caulobacter segnis
Rhodobacterales	Roseovarius	Thiotrichaceae	Phenylobacterium parvum
Rhodospirillales	Sulfuricella	Maricaulaceae	Flavobacterium sangjuense
Caulobacterales	Agreia	Aphanothecaceae	Elizabethkingia sp. JS20170427COW
Rickettsiales	Catenibacterium	Pseudoalteromonadaceae	Cloacibacterium caeni
Holosporales	Naasia	Chlorobiaceae	Rhodocytophaga rosea
Hyphomonadales	Halorhodospira	Promicromonosporaceae	Segatella copri
Candidatus Pelagibacterales	Laribacter	Streptosporangiaceae	Hoylesella buccalis
Burkholderiales	Martelella	Caedimonadaceae	Porphyromonas gingivalis
Neisseriales	Sandaracinobacteroides	Francisellaceae	Porphyromonas endodontalis
Nitrosomonadales	Acidocella	Saprospiraceae	Microbacterium sp. 10M-3C3
Rhodocyclales	Rhodospirillum	Frankiaceae	Microbacterium sediminis
Moraxellales	Falsirhodobacter	Ornithinimicrobiaceae	Microcella sp. MMS21-STM10
Xanthomonadales	Minwuia	Minwuiaceae	Rothia kristinae
Enterobacterales	Subtercola	Sneathiellaceae	Rothia aeria
Pseudomonadales	Candidatus Phytoplasma	Ustilaginaceae	Dermabacter vaginalis
Chromatiales	Sneathiella	Thermotogaceae	Kytococcus sedentarius
Legionellales	Herbiconiux	Schizosaccharomycetaceae	Gordonia rubripertincta
Cellvibrionales	Marisediminicola	Parvicellaceae	Corynebacterium kefirresidentii
Oceanospirillales	Aurantiacibacter	Candida/Metschnikowiaceae	Corynebacterium freneyi
Thiotrichales	Aristophania	Brettanomyces	Corynebacterium afermentans
Pasteurellales	Sporisorium	Pichia	Corynebacterium singulare
Alteromonadales	Pelagibacterium	Ustilaginoidea	Corynebacterium aurimucosum
Aeromonadales	Pelagerythrobacter	Purpureocillium	Corynebacterium genitalium
Vibrionales	Glaciihabitans	Botrytis	Corynebacterium accolens
Methylococcales	Schizosaccharomyces	Malassezia	Corynebacterium fournieri
Nevskiales	Parvicella	Puccinia	Corynebacterium durum
Acidiferrobacterales	Pyruvatibacter	Pararhizobium	Corynebacterium minutissimum
Cardiobacteriales	[Candida] auris	Sinorhizobium	Corynebacterium simulans
Hydrogenophilales	Brettanomyces nanus	Shinella	Corynebacterium jeikeium
Flavobacteriales	Brettanomyces bruxellensis	Rhodopseudomonas	Corynebacterium massiliense
Cytophagales	Pichia kudriavzevii	Variibacter	Corynebacterium hadale
Chitinophagales	Ustilaginoidea virens	Methylorubrum	Corynebacterium amycolatum
Bacteroidales	Purpureocillium takamizusanense	Microvirga	Corynebacterium appendicis
Marinilabiliales	Botrytis cinerea	Hyphomicrobium	Corynebacterium gottingense
Sphingobacteriales	Malassezia restricta	Bosea	Mycolicibacterium sediminis
Saprospirales	Malassezia vespertilionis	Nordella	Dietzia lutea
Candidatus Sulfidibacteriales	Puccinia triticina	Pseudorhodoplanes	Actinomyces massiliensis
Ignavibacteriales	Pararhizobium sp. BT-229	Ancylobacter	Gardnerella swidsinskii
Rhodothermales	Shinella sp. XGS7	Xanthobacter	Nakamurella sp. PAMC28650
Gemmatimonadales	Bradyrhizobium sp. S12-14-2	Brucella	Nakamurella multipartita
Micrococcales	Bradyrhizobium sp. 170	Aureimonas	Abyssicoccus albus
Mycobacteriales	Bradyrhizobium cosmicum	Sphingorhabdus	Gemella sanguinis
Propionibacteriales	Bradyrhizobium betae	Parasphingopyxis	Streptococcus sp. LPB0220
Candidatus Nanopelagicales	Bradyrhizobium arachidis	Rhizorhabdus	Streptococcus sanguinis
Actinomycetales	Bradyrhizobium japonicum	Aquisediminimonas	Lactococcus lactis
Micromonosporales	Bradyrhizobium diazoefficiens	Croceicoccus	Lactococcus cremoris
Pseudonocardiales	Bradyrhizobium icense	Altererythrobacter	Lactobacillus iners
Bifidobacteriales	Bradyrhizobium guangdongense	Qipengyuania	Lactobacillus jensenii
Streptosporangiales	Bradyrhizobium paxllaeri	Sphingosinicella	Granulicatella elegans
Geodermatophilales	Rhodopseudomonas palustris	Polymorphobacter	Granulicatella adiacens
Nakamurellales	Rhodopseudomonas sp. P2A-2r	Gemmobacter	Subdoligranulum variabile
Jatrophihabitantales	Rhodopseudomonas sp. SK50-23	Fuscovulum	Blautia obeum
Acidimicrobiales	[Pseudomonas] carboxydohydrogena	Tabrizicola	Anaeropeptidivorans aminofermentans
Coriobacteriales	Afipia carboxidovorans	Sediminicoccus	Anaerostipes hadrus
Eggerthellales	Variibacter gotjawalensis	Hypericibacter	[Ruminococcus] lactaris
Rubrobacterales	Methylorubrum populi	Haematospirillum	[Ruminococcus] gnavus
Solirubrobacterales	Hyphomicrobium sp. MC1	Phenylobacterium	Stomatobaculum sp. F0698
Bacillales	Mesorhizobium terrae	Candidatus Gromoviella	Agathobacter rectalis
Lactobacillales	Bosea sp. RAC05	Candidatus Paracaedibacter	Megamonas funiformis
Eubacteriales	Bosea sp. AS-1	Hyphomonas	Pelosinus sp. UFO1
Halanaerobiales	Nordella sp. HKS 07	Limnohabitans	Veillonella rogosae
Veillonellales	Enhydrobacter sp.	Rhodoferax	Anaerococcus prevotii
Tissierellales	Pseudorhodoplanes sinuspersici	Ottowia	Peptoniphilus sp. SAHP1
Erysipelotrichales	Devosia sp. A16	Melaminivora	Parvimonas micra
Oscillatoriales	Sphingopyxis sp. USTB-05	Polynucleobacter	Gloeocapsa sp. PCC 7428
Chroococcales	Sphingopyxis sp. EG6	Caballeronia	Deinococcus sp. AJ005
Synechococcales	Sphingopyxis sp. QXT-31	Pandoraea	Capsulimonas corticalis
Nostocales	Sphingopyxis sp. OPL5	Ephemeroptericola	Gemmata sp. SH-PL17
Pseudanabaenales	Sphingopyxis sp. PAMC25046	Caldimonas	Symmachiella dynata
Gloeobacterales	Sphingopyxis sp. DBS4	Sphaerotilus	Lentisphaera profundi
Mycoplasmatales	Sphingopyxis sp. FD7	Methylibium	Luteitalea sp. TBR-22
Entomoplasmatales	Sphingopyxis sp. 113P3	Aquincola	Fulvia fulva
Acholeplasmatales	Sphingopyxis sp. YF1	Inhella	Rhizoctonia solani
Mycoplasmoidales	Sphingopyxis sp. PET50	Ideonella	Acidovorax sp. 5MLIR

Anaerolineales	Sphingopyxis fribergensis	Piscinibacter	Legionella pneumophila
Caldilineales	Sphingopyxis macrogoltabida	Roseateles	Bradyrhizobium sp. CB1717
Chloroflexales	Sphingopyxis alaskensis	Leptothrix	Bradyrhizobium sp. I71
Tepidiformales	uncultured Sphingopyxis sp.	Rugamonas	Bradyrhizobium barranii
Thermales	Sphingopyxis granuli	Collimonas	Bradyrhizobium amphicarpaeae
Deinococcales	Sphingopyxis terrae	Tepidimonas	Xanthobacter dioxanivorans
Fimbriimonadales	Sphingomonas sp. NIC1	Paucibacter	Sphingosinithalassobacter sp. CS137
Pirellulales	Sphingomonas sp. PAMB00755	Rhizobacter	Croceicoccus marinus
Gemmatales	Sphingomonas sp. KC8	Sutterella	Fuscovulum sp. YMD61
Planctomycetales	Sphingomonas sp. SUN039	Neisseria	Roseomonas sp. OT10
Isosphaerales	Sphingomonas sp. MM-1	Aquella	Roseococcus microcysteis
Phycisphaerales	Sphingomonas sp. NBWT7	Vitreoscilla	Brevibacterium sp. CS2
Tepidisphaerales	Sphingomonas sp. FARSPH	Aquaspirillum	Collinsella aerofaciens
Verrucomicrobiales	Sphingomonas paucimobilis	Chitinimonas	Streptococcus salivarius
Limisphaerales	Sphingomonas koreensis	Methylotenera	Anaerococcus mediterraneensis
Opitutales	Sphingomonas hankookensis	Candidatus Methylopumilus	Neochlamydia sp. S13
Puniceicoccales	Sphingomonas paeninsulae	Sulfuricystis	Rhodopseudomonas boonkerdii
Methylacidiphilales	Sphingomonas lacunae	Nitrosomonas	Hyphomicrobium nitrativorans
Parachlamydiales	Sphingomonas insulae	Oryzomicrobium	Sphingomonas changnyeongensis
Chlamydiales	Sphingobium amiense	Fluviibacter	Sphingomonas profundi
Campylobacterales	Sphingobium yanoikuyae	Casimicrobium	Haematobacter massiliensis
Nautiliales	Sphingobium xenophagum	Moraxella	Pseudoroseomonas cervicalis
Polyangiales	Sphingobium cloacae	Aquirhabdus	Candidatus Phycorickettsia trachydisci
Haliangiales	Sphingorhabdus lutea	Lysobacter	Methylobacter sp. S3L5C
Nannocystales	Aquisediminimonas profunda	Luteimonas	Flavihumibacter fluvii
Myxococcales	Sphingosinicella microcystinivorans	Ahniella	Corynebacterium striatum
Bdellovibrionales	Gemmobacter fulvus	Buchnera	Mycolicibacterium arabiense
Bacteriovoracales	Gemmobacter aquarius	Sodalis	Deinococcus aquaticus
Silvanigrellales	Tabrizicola piscis	Legionella	Porphyromonas asaccharolytica
Desulfovibrionales	Roseomonas gilardii	Haemophilus	Microbacterium testaceum
Desulfobacterales	Roseomonas fluvialis	Methylobacter	Corynebacterium macginleyi
Geobacterales	Sediminicoccus rosea	Steroidobacter	Mycolicibacterium phocaicum
Desulfuromonadales	Haematospirillum jordaniae	Hydrogenophilus	Actinomyces sp. oral taxon 414
Desulfobulbales	Brevundimonas sp. Bb-A	Acidithiobacillus	Actinomyces sp. oral taxon 171
Candidatus Nanosynbacterales	Caulobacter sp. NIBR1757	Capnocytophaga	Winkia neuii
Candidatus Saccharimonadales	Caulobacter sp. NIBR2454	Myroides	Anaerobutyricum hallii
Candidatus Babeliales	Caulobacter mirabilis	Fluviicola	Fenollaria massiliensis
Candidatus Absconditabacterales	Phenylobacterium sp. NIBR 498073	Epilithonimonas	Peptoniphilus harei
Spirochaetales	Phenylobacterium sp. LH3H17	Elizabethkingia	Stutzerimonas stutzeri
Leptospirales	Phenylobacterium zucineum	Cloacibacterium	Immundisolibacter cernigliae
Terriglobales	endosymbiont of Acanthamoeba sp. UWC8	Weeksella	Neisseria subflava
Bryobacterales	Candidatus Paracaedibacter acanthamoebae	Owenweeksia	Nitrosomonas ureae
Vicinamibacterales	Hyphomonas sp. KY3	Croceimicrobium	Nitrosomonas sp. sh817
Holophagales	Hyphomonas neptunium	Candidatus Karelsulcia	Varunaivibrio sulfuroxidans
Nitrospirales	Limnohabitans sp. TEGF004	Ichthyobacterium	Bacteroides uniformis
Thermotogales	Limnohabitans sp. 63ED37-2	Aquirufa	Porphyromonas somerae
Fusobacteriales	Limnohabitans sp. MORI2	Spirosoma	Paludisphaera borealis
Bradymonadales	Limnohabitans sp. INBF002	Cytophaga	Streptococcus pneumoniae
Synergistales	Limnohabitans sp. 103DPR2	Hymenobacter	Succinivibrio dextrinosolvens
Aquificales	Acidovorax temperans	Pontibacter	Bradyrhizobium sp. 186
Haloferacales	Ottowia oryzae	Emticicia	Sphingobium phenoxybenzoativorans
Methanobacteriales	Polynucleobacter sp. MWH-Aus1W21	Fulvivirga	Croceicoccus naphthovorans
Lefavirales	Polynucleobacter sp. AP-Sving-400A-A2	Chryseolinea	Qipengyuania sediminis
Herpesvirales	Polynucleobacter sp. MWH-CaK5	Marivirga	Stella humosa
Kitasatosporales	Polynucleobacter sp. MG-Unter2-18	Candidatus Cardinium	Microbacterium schleiferi
Thermoanaerobacterales	Polynucleobacter sp. AP-Ainpum-60-G11	Sediminibacterium	Dorea longicatena
Selenomonadales	Polynucleobacter difficilis	Arachidicoccus	Peptoniphilus ivorii
Capsulimonadales	Polynucleobacter asymbioticus	Chitinophaga	Ezakiella coagulans
Mycosphaerellales	Polynucleobacter corsicus	Ferruginibacter	Novosphingobium sp. EMRT-2
Cantharellales	Polynucleobacter yangtzensis	Panacibacter	Porphyrobacter sp. YT40
Trueperales	Ephemeroptericola cinctiostellae	Flavihumibacter	Brevundimonas sp.
Chrysiogenales	Schlegelella aquatica	Filimonas	Hymenobacter sp. 5317J-9
Chlorobiales	Sphaerotilus sulfidivorans	Flavisolibacter	Hymenobacter monticola
Maricaulales	Sphaerotilus sp. FB-5	Niabella	Hymenobacter sedentarius
Deferribacterales	Aquincola tertiaricarbonis	Lacibacter	Hymenobacter sp. BRD128
Frankiales	Inhella inkyongensis	Bacteroides	Ferruginibacter albus
Minwuiales	Ideonella dechloratans	Phocaeicola	Truepera radiovictrix
Sneathiellales	Piscinibacter sp. XHJ-5	Porphyromonas	Acinetobacter baumannii
Ustilaginales	Leptothrix cholodnii	Paludibacter	Bradyrhizobium sp. WD16
Schizosaccharomycetales	Oxalobacter aliiformigenes	Parabacteroides	Bradyrhizobium sp. CCBAU 051011
Metschnikowiaceae	Rugamonas sp. DEMB1	Sphingobacterium	Bradyrhizobium sp. CCBAU 51753
Debaryomycetaceae	Aquabacterium olei	Mucilaginibacter	Bradyrhizobium sp. 191
Pichiaceae	Tepidimonas taiwanensis	Haliscomenobacter	Bradyrhizobium commune
Saccharomycetaceae	Paucibacter sp. S2-9	Candidatus Sulfidibacterium	Bradyrhizobium symbiodeficiens
Trichomonascaceae	Paucibacter aquatile	Melioribacter	Sphingomonas daechungensis
Clavicipitaceae	Rhizobacter gummiphilus	Rhodothermus	Sphingobium indicum
Nectriaceae	Neisseria gonorrhoeae	Gemmatimonas	Novosphingobium sp. KA1
Ophiocordycipitaceae	Aquella oligotrophica	Gemmatirosa	Novosphingobium decolorationis
Glomerellaceae	Vitreoscilla filiformis	Dermacoccus	Blastomonas sp. RAC04
Chaetomiaceae	Aquaspirillum sp. LM1	Rhodoluna	Porphyrobacter sp. ULC335
Sclerotiniaceae	Chitinimonas koreensis	Aquiluna	Candidatus Fokinia solitaria
Trichocomaceae	Methylotenera versatilis	Aurantimicrobium	Aquisphaera giovannonii
Aspergillaceae	Methylotenera mobilis	Leucobacter	Desulfurispirillum indicum
Malasseziaceae	Methylophilus sp. TWE2	Salinibacterium	Noviherbaspirillum sp. UKPF54
Pucciniaceae	Sulfuritalea hydrogenivorans	Agromyces	Roseateles depolymerans
Rhizobiaceae	Methyloversatilis sp. RAC08	Microcella	Eleftheria terrae
Nitrobacteraceae	Oryzomicrobium terrae	Rothia	Bradyrhizobium sp. 1(2017)
Methylobacteriaceae	Fluviibacter phosphoraccumulans	Nesterenkonia	Bradyrhizobium genosp. L
Hyphomicrobiaceae	Casimicrobium huifangae	Citricoccus	Bradyrhizobium guangzhouense
Phyllobacteriaceae	Moraxella osloensis	Brachybacterium	Ancylobacter polymorphus
Boseaceae	Acinetobacter ursingii	Cellulomonas	Sphingobium sp. AntQ-1
Devosiaceae	Aquirhabdus parva	Gordonia	Brevundimonas albigilva
Xanthobacteraceae	Stenotrophomonas maltophilia	Mycolicibacterium	Asticcacaulis sp. ZE23SCel15
Methylocystaceae	Lysobacter sp. HDW10	Mycobacteroides	Bradyrhizobium sp. CCBAU 53340
Phreatobacteraceae	Lysobacter oculi	Lawsonella	Bradyrhizobium xenonodulans
Brucellaceae	Ahniella affigens	Cutibacterium	Caldimonas brevitalea
Aurantimonadaceae	Buchnera aphidicola	Tessaracoccus	Lautropia mirabilis
Stappiaceae	Legionella lytica	Candidatus Planktophila	Nocardioides aquaticus
Sphingomonadaceae	Legionella fallonii	Actinomyces	Ethanoligenens harbinense
Erythrobacteraceae	Haemophilus parainfluenzae	Plantactinospora	Micropruina glycogenica
Sphingosinicellaceae	Steroidobacter denitrificans	Gardnerella	Bosea sp. REN20
Paracoccaceae	Hydrogenophilus thermoluteolus	Nakamurella	Tsuneonella dongtanensis
Roseobacteraceae	Flavobacterium ammonificans	Jatrophihabitans	Frigoribacterium sp. NBH87
Acetobacteraceae	Flavobacterium psychrophilum	Aquihabitans	Planctomyces sp. SH-PL62
Rhodospirillaceae	Flavobacterium ammoniigenes	Iamia	Caenibius tardaugens
Azospirillaceae	Flavobacterium haoranii	Actinomarinicola	Caulobacter sp. S6
Thalassospiraceae	Flavobacterium channae	Ilumatobacter	Homoserinibacter sp. YIM 151385

Caulobacteraceae	Flavobacterium oreochromis	Rubrobacter	Corynebacterium imitans
Rickettsiaceae	Fluviicola taffensis	Geobacillus	Sphingomonas sp. S6-11
Anaplasmataceae	Weeksella virosa	Anoxybacillus	Rhodoplanes sp. Z2-YC6860
Candidatus Midichloriaceae	Owenweeksia hongkongensis	Caldibacillus	Legionella sp. PC1000
Holosporaceae	Croceimicrobium hydrocarbonivorans	Heyndrickxia	Candidatus Hydrogenosomobacter endosymbioticus
Candidatus Paracaedibacteraceae	Candidatus Karelsulcia muelleri	Gemella	Phycisphaera mikurensis
Hyphomonadaceae	Aquirufa lenticrescens	Lactococcus	Sinorhizobium meliloti
Comamonadaceae	Aquirufa antheringensis	Lactobacillus	Chenggangzhangella methanolivorans
Burkholderiaceae	Spirosoma pollinicola	Lactiplantibacillus	Sphingomonas hengshuiensis
Sphaerotilaceae	Cytophaga hutchinsonii	Weissella	Candidatus Endolissoclinum faulkneri
Oxalobacteraceae	Emticicia sp. 21SJ11W-3	Leuconostoc	Caldimonas thermodepolymerans
Alcaligenaceae	Emticicia oligotrophica	Dolosigranulum	Thiomonas intermedia
Sutterellaceae	Chryseolinea soli	Granulicatella	Streptococcus pyogenes
Neisseriaceae	Sediminibacterium sp. TEGAF015	Blautia	Poriferisphaera corsica
Aquaspirillaceae	Ferruginibacter lapsinanis	Veillonella	Brevundimonas nasdae
Chitinibacteraceae	Panacibacter ginsenosidivorans	Anaerococcus	Candidatus Nucleicultrix amoebiphila
Chromobacteriaceae	Filimonas lacunae	Peptoniphilus	Heyndrickxia coagulans
Methylophilaceae	Lacibacter sp. S13-6-22	Finegoldia	Sphingomonas panacisoli
Sterolibacteriaceae	Phocaeicola vulgatus	Erysipelothrix	Defluviicoccus vanus
Nitrosomonadaceae	Phocaeicola dorei	Oscillatoria	Acidobacterium capsulatum
Thiobacillaceae	Phocaeicola coprophilus	Crinalium	Parvibaculum lavamentivorans
Gallionellaceae	Porphyromonas sp. oral taxon 275	Chondrocystis	Sphingomonas sp. ZFBP2030
Azonexaceae	Paludibacter propionicigenes	Synechococcus	Sphingosinicella sp. BN140058
Rhodocyclaceae	Sphingobacterium multivorum	Synechocystis	Sulfuricella denitrificans
Zoogloeaceae	Haliscomenobacter hydrossis	Nostoc	Agreia sp. COWG
Fluviibacteraceae	Candidatus Sulfidibacterium hydrothermale	Calothrix	Naasia aerilata
Casimicrobiaceae	Melioribacter roseus	Pseudanabaena	Streptococcus infantis
Moraxellaceae	Gemmatimonas aurantiaca	Leptolyngbya	Laribacter hongkongensis
Xanthomonadaceae	Gemmatimonas groenlandica	Gloeobacter	Sandaracinobacteroides saxicola
Rhodanobacteraceae	Gemmatirosa kalamazoonensis	Brevefilum	Acidocella sp. MX-AZ03
Enterobacteriaceae	Dermacoccus nishinomiyaensis	Caldilinea	Protaetiibacter sp. SSC-01
Morganellaceae	Dermacoccus sp. PAMC28757	Tepidiforma	Novosphingobium sp. THN1
Erwiniaceae	Microbacterium aurum	Fimbriimonas	Falsirhodobacter sp. PG104
Yersiniaceae	Microbacterium azadirachtae	Pirellula	Sphingomonas sp. 7/4-4
Bruguierivoracaceae	Rhodoluna lacicola	Mariniblastus	Caulobacter vibrioides
Pectobacteriaceae	Candidatus Rhodoluna planktonica	Aureliella	Bosea sp. PAMC 26642
Pseudomonadaceae	Aquiluna borgnonia	Anatilimnocola	Qipengyuania flava
Chromatiaceae	Aquiluna sp. KACHI24	Bremerella	Herbiconiux sp. SALV-R1
Ectothiorhodospiraceae	Aurantimicrobium photophilum	Lacipirellula	Herbiconiux sp. L3-i23
Legionellaceae	Kocuria palustris	Thermogutta	Marisediminicola antarctica
Coxiellaceae	Kocuria rhizophila	Telmatocola	Aristophania vespae
Cellvibrionaceae	Rothia mucilaginosa	Gemmata	Caulobacter rhizosphaerae
Halomonadaceae	Brevibacterium luteolum	Tuwongella	Salinibacterium sp. ZJ70
Oceanospirillaceae	Gordonia otitidis	Frigoriglobus	Sporisorium graminicola
Piscirickettsiaceae	Gordonia bronchialis	Fuerstiella	Sphingomonas sanguinis
Pasteurellaceae	Corynebacterium tuberculostearicum	Planctomyces	Bradyrhizobium sp. CB1024
Alteromonadaceae	Corynebacterium canis	Planctopirus	Bradyrhizobium guangxiense
Shewanellaceae	Corynebacterium pseudodiphtheriticum	Polystyrenella	Qipengyuania citrea
Aeromonadaceae	Corynebacterium ureicelerivorans	Rubinisphaera	Qipengyuania spongiae
Vibrionaceae	Corynebacterium sp. SCR221107	Singulisphaera	Bradyrhizobium sp. CB1015
Methylococcaceae	Corynebacterium kroppenstedtii	Isosphaera	Sphingobium sp. WTD-1
Steroidobacteraceae	Mycobacteroides chelonae	Humisphaera	Bradyrhizobium septentrionale
Nevskiaceae	Dietzia psychralcaliphila	Verrucomicrobium	Bradyrhizobium vignae
Acidiferrobacteraceae	Dietzia sp. oral taxon 368	Roseimicrobium	Sphingomonas sp. So64.6b
Cardiobacteriaceae	Dietzia kunjamensis	Sulfuriroseicoccus	Bradyrhizobium sp. ORS 285
Hydrogenophilaceae	Lawsonella clevelandensis	Akkermansia	Bradyrhizobium sp. CCGE-LA001
Flavobacteriaceae	Cutibacterium acnes	Fontisphaera	Bradyrhizobium sp. C-145
Crocinitomicaceae	Cutibacterium granulosum	Lacunisphaera	Bradyrhizobium sp.
Weeksellaceae	Cutibacterium modestum	Nibricoccus	Bradyrhizobium sp. 62B
Schleiferiaceae	Cutibacterium avidum	Opitutus	Bradyrhizobium sp. 4
Blattabacteriaceae	Microlunatus phosphovorus	Horticoccus	Bradyrhizobium sp. 40
Cytophagaceae	Candidatus Planktophila vernalis	Ruficoccus	Bradyrhizobium sp. LCT2
Hymenobacteraceae	Candidatus Planktophila lacus	Candidatus Xiphinematobacter	Bradyrhizobium sp. 172
Cyclobacteriaceae	Plantactinospora sp. BB1	Methylacidimicrobium	Bradyrhizobium daqingense
Spirosomaceae	Plantactinospora sp. BC1	Methylacidiphilum	Bradyrhizobium ottawaense
Fulvivirgaceae	Aquihabitans sp. Kera 3	Parachlamydia	Bradyrhizobium canariense
Flammeovirgaceae	Aquihabitans sp. G128	Candidatus Protochlamydia	Tardiphaga robiniae
Marivirgaceae	Actinomarinicola tropica	Candidatus Rhabdochlamydia	Sphingomonas qomolangmaensis
Amoebophilaceae	Ilumatobacter coccineus	Waddlia	Sphingomonas psychrotolerans
Chitinophagaceae	Rubrobacter xylanophilus	Chlamydia	Sphingomonas ginsengisoli An et al. 2013
Prevotellaceae	Anoxybacillus flavithermus	Tichowtungia	Sphingobium sp. V4
Bacteroidaceae	Caldibacillus thermoamylovorans	Campylobacter	Sphingobium sp. RSMS
Porphyromonadaceae	Gemella haemolysans	Helicobacter	Sphingobium sp. YC-XJ3
Dysgonomonadaceae	Streptococcus thermophilus	Sulfuricurvum	Sphingobium fuliginis
Paludibacteraceae	Lactobacillus crispatus	Sorangium	Novosphingobium sp. KACC 22771
Tannerellaceae	Lactiplantibacillus plantarum	Polyangium	Sphingorhabdus sp. YGSMI21
Sphingobacteriaceae	Dolosigranulum pigrum	Chondromyces	Blastomonas fulva
Haliscomenobacteraceae	Blautia wexlerae	Labilithrix	Porphyrobacter sp. LM 6
Melioribacteraceae	Finegoldia magna	Sandaracinus	Croceicoccus sp. Ery5
Gemmatimonadaceae	Oscillatoria nigro-viridis	Nannocystis	Caenibius sp. WL
Dermacoccaceae	Crinalium epipsammum	Anaeromyxobacter	Protaetiibacter larvae
Microbacteriaceae	Chondrocystis sp. NIES-4102	Pseudobdellovibrio	Microcella flavibacter
Micrococcaceae	Brevefilum fermentans	Bdellovibrio	Glaciihabitans sp. INWT7
Dermabacteraceae	Caldilinea aerophila	Micavibrio	Bradyrhizobium oligotrophicum
Intrasporangiaceae	Fimbriimonas ginsengisoli	Bacteriovorax	Bradyrhizobium sp. CB3035
Brevibacteriaceae	Pirellula sp. SH-Sr6A	Pigmentibacter	Tardiphaga sp. 37S4
Cellulomonadaceae	Pirellula staleyi	Desulfomicrobium	Novosphingobium kaempferiae
Gordoniaceae	Mariniblastus fucicola	Candidatus Nanosynbacter	Sphingomonas sp. S1-29
Corynebacteriaceae	Aureliella helgolandensis	Candidatus Saccharimonas	Bradyrhizobium sp. CCBAU 53351
Candidatus Absconditicoccus	Sphingobium sp. YBL2	Candidatus Mycosynbacter	Schizosaccharomyces osmophilus
Entomospira	Croceicoccus sp. Ery15	Candidatus Chromulinivorax	Parvicella tangerina
Treponema	Bradyrhizobium sp. NC92	Candidatus Babela	
Bradyrhizobium sp. WBAH10	Protaetiibacter intestinalis	Porphyrobacter sp. HT-58-2	

Supplementary Table 10. Hepatic microbiota in independent single cell RNA-seq validation data

Bacteroidota	Streptomyces sp. A10(2020)	Pichia	Lachnospiraceae
Herbaspirillum	Lactiplantibacillus	Kluyveromyces marxianus	Eubacteriales
Homo sapiens	Mycoplasmopsis	Kluyveromyces	Clostridia
Homo	Weeksellaceae	Pichia kudriavzevii	Mycolicibacterium
Hominidae	Lacticaseibacillus	Schizosaccharomyces	Acholeplasmatales
Burkholderia cenocepacia	Eukaryota	Schizosaccharomycetaceae	Entomoplasmatales
Primates	Durnavirales	Saccharomycetaceae	Chitinophagia
Enterobacterales	Piccovirales	Saccharomycetales	Veillonellales
Bacilli	Algavirales	Saccharomycetes	Methanobacteria
Negativicutes	Megaviricetes	Ascomycota	Deinococcaceae
Phocaeicola	Revtraviricetes	Neisseriaceae	Rhodococcus
Staphylococcaceae	Duplopiviricetes	Lacticaseibacillus rhamnosus	Aspergillus chevalieri
Buchnera aphidicola	Quintoviricetes	Fungi	Mycobacterium avium
Nocardiaceae	Cossaviricota	Ophiocordycipitaceae	Mycobacterium
Microbacteriaceae	Artverviricota	Hydrogenophaga	Mycobacteriaceae
Streptosporangiales	Pisuviricota	Candidatus Purcelliella pentastirinorum	Actinomycetes
Kitasatosporales	Pararnavirae	Candidatus Purcelliella	Thermoanaerobacter
Propionibacteriales	Orthornavirae	Acinetobacter baumannii	Cutibacterium acnes
Micromonosporales	Shotokuvirae	Acinetobacter	Tissierellales
Mycobacteriales	Nucleocytoviricota	Moraxellaceae	Tissierellia
Micrococcales	Bamfordvirae	Thermoanaerobacter wiegelii	Corynebacterium
Fusobacterium	Metschnikowiaceae	Staphylococcus pasteuri	Bacteroidales
Aeromonadaceae	Caudoviricetes	Corynebacterium matruchotii	Anaerococcus
Oxalobacter	Uroviricota	Prescottella equi	Actinomyces
Microbacterium oxydans	Herviviricetes	Lysobacter capsici	Corynebacteriaceae
Rhizobiaceae	Peploviricota	Mammaliicoccus lentus	Blattabacterium punctulatus
Enterococcaceae	Heunggongvirae	Candida dubliniensis	Pucciniomycetes
Bacteroidaceae	Shewanellaceae	Sphingomonadaceae	Malasseziales
Comamonadaceae	Thermothielavioides	Nitrobacteraceae	Pararhizobium
Burkholderiales	Thermothielavioides terrestris	Brevundimonas	Lactiplantibacillus plantarum
Thermothelomyces thermophilus	Schizosaccharomyces osmophilus	Trichomonascaceae	Lactobacillus helveticus
Chordata	Klebsiella variicola	Mammalia	Fusobacterium hwasookii
Caulobacteraceae	Pseudomonas psychrotolerans	Botrytis cinerea	Lactobacillus
Malassezia restricta	Flavobacterium	Acinetobacter radioresistens	Peptoniphilaceae
Debaryomycetaceae	Brucella	Erythroparvovirus	Malasseziomycetes
Oxalobacteraceae	Shewanella	Blattabacteriaceae	Prymnesium kappa virus
Pasteurella multocida	Methanobrevibacter	Veillonella atypica	Clostridium botulinum
Pasteurella	Ortervirales	Talaromyces marneffei	Clostridium
Malasseziaceae	Methanobacteriaceae	Bradyrhizobium	Schizosaccharomycetes
Haemophilus	Methanobacteriales	Phocaeicola dorei	Sordariomycetes
Campylobacteraceae	Archaea	Chaetomiaceae	Leotiomycetes
Helicobacteraceae	Acholeplasmataceae	Hyphomicrobiales	Eurotiomycetes
Pseudomonadales	Campylobacterales	Clavicipitaceae	Dothideomycetes
Thiotrichales	Spiroplasma	Schizosaccharomycetales	Colletotrichum lupini
Pasteurellaceae	Spiroplasmataceae	Blattabacterium	Micromonospora profundi
Acinetobacter dispersus	Plantactinospora sp. BC1	Lactobacillaceae	Bradyrhizobium septentrionale
Thermoanaerobacterales	Helicobacter pylori	Candidatus Phytoplasma	Priestia megaterium
Glomerellaceae	Mesomycoplasma hyopneumoniae	Microbacterium	Bacillus
Lysobacter	Mycoplasma	Xanthomonas	Bacillales
Edwardsiella ictaluri	Mycoplasmataceae	Candidatus Karelsulcia	Gemella haemolysans
Plantactinospora	Helicobacter	Metazoa	Gemella
Vibrio harveyi	Escherichia albertii	Botrytis	Pasteurellales
Vibrio	Mycoplasmatales	Anaerococcus prevotii	Aeromonadales
Aeromonas veronii	Puccinia triticina	synthetic construct	Vibrionales
Mycoplasma wenyonii	Plantactinospora sp. BB1	Shinella	Alteromonadales
Aeromonas	Neisseriales	Kingella	Pseudomonadaceae
Vibrionaceae	Streptomycetaceae	Buchnera	Oceanospirillales
Edwardsiella	Purpureocillium takamizusanense	Fusobacteriota	Xanthomonadales
Shewanella baltica	Actinomycetaceae	Xanthomonadaceae	Enterococcus faecalis
Shigella flexneri	Candidatus Nardonella	Burkholderia	Enterococcus
Shigella	Caulobacterales	Clostridiaceae	Candidatus Annandia pinicola
Serratia marcescens	Sphingomonadales	Veillonellaceae	Bradyrhizobium sp. CCBAU 53421
Serratia	Rhodobacterales	Mollicutes	Candidatus Annandia
Chryseobacterium	Rhodospirillales	Propionibacteriaceae	Streptococcus
Salmonella	Actinomycetales	Stutzerimonas stutzeri	Streptococcaceae
Klebsiella pneumoniae	Spirochaetia	Erythroparvovirus ungulate1	Deinococcota
Klebsiella	Spirochaetota	Epsilonproteobacteria	Staphylococcus hominis
Escherichia coli	Fusobacteriaceae	Cyanophyceae	Staphylococcus epidermidis
Escherichia	Fusobacteriales	Pseudomonas sp. JBR1	Staphylococcus aureus
Malassezia	Fusobacteriia	Rhodococcus sp. JS3073	Staphylococcus
Fusarium oxysporum	Curtobacterium	Pararhizobium sp. BT-229	Micrococcus
Fusarium	Thermomonosporaceae	Prescottella	Micrococcaceae
Herpesvirales	Actinomycetota	Candida/Metschnikowiaceae	Ustilaginoidea
Candida	Flavobacteriales	Campylobacterota	Bacillota
Enterobacter	Bacteroidia	Oxalobacter aliiformigenes	Gammaproteobacteria
Colletotrichum	Bacteria	Veillonella	Pseudomonadota
Actinomyces oris	uncultured Hydrogenophaga sp.	Staphylococcus capitis	Aspergillus puulaauensis
Mycoplasmatota	Actinomadura madurae	Pseudomonas sp. I3-I5	Talaromyces rugulosus
Enterobacteriaceae	Actinomadura	Mesomycoplasma	Burkholderiaceae
Puccinia	Candidatus Nardonella dryophthoridicola	Stutzerimonas	Methylobacteriaceae
Ustilaginaceae	Aeromonas dhakensis	Sclerotiniaceae	Deinococcales
Ustilaginales	Fusarium falciforme	Mycoplasmopsis bovis	Brucellaceae
Pucciniaceae	Streptomyces peucetius	Salmonella enterica	Flavobacteriia
Pucciniales	Campylobacter	Euryarchaeota	Retroviridae
Ustilaginomycetes	Thermothelomyces	Moraxellales	Nostocales
Basidiomycota	Cutibacterium	Pseudomonas aeruginosa	Ustilaginoidea virens
Helotiales	Streptomyces fradiae	Pseudomonas	Pichiaceae
Bordetella	Morganellaceae	Trichocomaceae	Candidatus Carsonella ruddii
Sordariales	Hafniaceae	Kamchatkavirus AP45	Candidatus Carsonella
Hypocreales	Yersiniaceae	Kamchatkavirus	Aspergillaceae
Talaromyces	Pectobacteriaceae	Staphylococcus pseudintermedius	Leptotrichiaceae
Alcaligenaceae	Erwiniaceae	Halomonadaceae	Cyanobacteriota
Aspergillus	Deinococci	Betaproteobacteria	Nectriaceae
Eurotiales	Streptomyces	Alphaproteobacteria	Partitiviridae
[Candida] auris	Micromonospora	Arcobacteraceae	Parvoviridae
Psychrobacter	Carnobacteriaceae	Micromonosporaceae	Aspergillus luchuensis
Flavobacteriaceae	Lactobacillales	Mammaliicoccus	Purpureocillium
Saccharomyces cerevisiae	Bacillaceae	Streptococcus mitis	Phycodnaviridae
Saccharomyces	Thermoanaerobacteraceae	Priestia	Glomerellales
Viruses			

Supplementary Table 11: Individual level host gene-microbiota interaction results by LASSO in Control

ID	Gene	Taxa	Coeff	FDR	ID	Gene	Taxa	Coeff	FDR	ID	Gene	Taxa	Coeff	FDR	ID	Gene	Taxa	Coeff	FDR
1	AAAS	Paucibacter	-0.393	7.78E-04	1385	DUSP1	Actinomyces naeslundii	-0.410	5.84E-07	2769	MIF	Paucibacter	-0.264	3.38E-04	4153	SERPINA10	Actinomyces naeslundii	0.197	6.04E-13
2	AAAS	Ustilaginaceae	0.056	5.24E-19	1386	DUSP1	Leptotrichiaceae	0.186	3.07E-05	2770	MIGA2	Halomonadaceae	-0.327	2.15E-04	4154	SERPINA11	Actinomyces naeslundii	0.205	4.75E-14
3	AAMDC	Cutibacterium modestum	-0.778	1.49E-06	1387	DUSP1	Methylorubrum	0.448	6.75E-05	2771	MIIP	Escherichia coli	-0.396	1.62E-12	4155	SERPINA12	Kytococcaceae	0.133	6.22E-03
4	AAMDC	Nostocales	0.251	4.76E-04	1388	DUSP15	Kytococcaceae	0.384	1.19E-06	2772	MINAR1	Pseudomonas oleovorans	-0.377	4.33E-59	4156	SERPINA4	Dolosigranulum pigrum	-0.398	1.14E-04
5	AARS1	Actinomyces naeslundii	0.397	1.30E-04	1389	DUSP4	Dolosigranulum pigrum	0.272	5.04E-05	2773	MINDY2	Yersiniaceae	0.491	3.56E-03	4157	SERPINA9	Pseudomonas oleovorans	-0.269	5.23E-04
6	AASDHPPT	Escherichia coli	0.265	6.37E-03	1390	DUSP5	Cutibacterium acnes	0.336	5.37E-04	2774	MINK1	Bradyrhizobium elkanii	-0.661	4.36E-05	4158	SERPINB2	Kytococcaceae	0.238	6.82E-08
7	ABCA10	Actinomyces naeslundii	-0.231	3.25E-04	1391	DUSP7	Leotiomycetes	-0.515	2.12E-03	2775	MIP	Dolosigranulum pigrum	0.203	8.58E-06	4159	SERPINB2	Pseudomonas oleovorans	-0.263	6.27E-12
8	ABCA12	Kytococcaceae	0.203	1.34E-08	1392	DUT	Actinomyces naeslundii	-0.505	9.68E-06	2776	MKNK1	Actinomyces naeslundii	-0.261	2.26E-07	4160	SERPINC1	Actinomyces naeslundii	0.448	2.18E-08
9	ABCA12	Pseudomonas oleovorans	-0.296	9.11E-13	1393	DUT	Alphaproteobacteria	-0.597	1.01E-03	2777	MKNK1	Ustilaginaceae	0.008	5.50E-04	4161	SERPIND1	Actinomyces naeslundii	0.518	1.52E-16
10	ABCA13	Pseudomonas oleovorans	-0.289	1.64E-08	1394	DXO	Actinomyces naeslundii	-0.379	4.54E-18	2778	MLC1	Kytococcaceae	0.289	1.24E-03	4162	SERPINE1	Actinomyces naeslundii	-0.266	2.72E-06
11	ABCA13	Stenotrophomonas maltophilia	0.381	1.31E-03	1395	DYM	Actinomyces naeslundii	0.422	2.06E-04	2779	MLEC	Actinomyces naeslundii	0.373	8.58E-09	4163	SERPINE2	Rhodoferax	-0.017	3.46E-05
12	ABCA2	Escherichia coli	-0.180	4.30E-12	1396	DYNC1I1	Pseudomonas oleovorans	-0.179	1.91E-04	2780	MLH3	Ustilaginaceae	0.217	2.08E-03	4164	SERPINF2	Paucibacter	-0.379	4.42E-03
13	ABCA5	Ustilaginaceae	0.273	1.57E-04	1397	DYNLT2	Actinomyces naeslundii	-0.368	1.10E-11	2781	MLLT6	Actinomyces naeslundii	-0.354	3.52E-152	4165	SERPING1	Actinomyces naeslundii	0.425	3.24E-09
14	ABCB11	Escherichia coli	-0.368	2.53E-06	1398	DYNLT3	Escherichia coli	0.417	3.22E-78	2782	MLPH	Actinomyces naeslundii	-0.226	1.06E-11	4166	SERTAD1	Kytococcaceae	-0.107	6.57E-08
15	ABCB5	Kytococcaceae	0.292	3.22E-10	1399	DYRK4	Dolosigranulum pigrum	0.397	5.09E-04	2783	MLXIPL	Actinomyces naeslundii	-0.318	5.33E-26	4167	SERTAD3	Actinomyces naeslundii	-0.341	2.55E-04
16	ABCB5	Pseudomonas oleovorans	-0.309	8.59E-14	1400	DYSF	Cutibacterium modestum	0.711	4.83E-11	2784	MMADHC	Escherichia coli	0.192	1.08E-05	4168	SERTM1	Acinetobacter johnsonii	0.459	2.11E-04
17	ABCB6	Escherichia coli	-0.483	2.51E-07	1401	DZANK1	Kytococcaceae	0.198	6.25E-03	2785	MMEL1	Kytococcaceae	0.246	9.89E-06	4169	SERTM1	Streptomyces sp. T12	0.317	6.75E-04
18	ABCB8	Escherichia coli	-0.301	9.31E-06	1402	DZIP3	Ustilaginaceae	0.243	3.78E-03	2786	MMGT1	Actinomyces naeslundii	0.135	7.77E-13	4170	SETBP1	Kytococcaceae	0.185	7.21E-05
19	ABCC12	Kytococcaceae	0.124	2.79E-05	1403	E2F1	Deinococcaceae	0.475	9.80E-05	2787	MMGT1	Paucibacter	0.335	3.58E-03	4171	SETD4	Ustilaginaceae	0.238	2.28E-04
20	ABCC12	Pseudomonas oleovorans	-0.369	3.40E-10	1404	E2F4	Bradyrhizobium elkanii	-0.522	2.52E-04	2788	MMP24	Escherichia coli	-0.461	3.84E-05	4172	SETDB1	Staphylococcus epidermidis	-0.416	6.23E-03
21	ABCC8	Pseudomonas oleovorans	-0.311	8.71E-15	1405	E4F1	Escherichia coli	-0.276	3.48E-58	2789	MMRN1	Pseudomonadaceae	0.633	1.14E-04	4173	SETDB2	Dolosigranulum pigrum	0.421	8.59E-06
22	ABCD4	Actinomyces naeslundii	-0.289	5.52E-04	1406	EBAG9	Escherichia coli	0.231	1.37E-07	2790	MMRN2	Paucibacter	-0.395	2.87E-04	4174	SF3A1	Actinomyces naeslundii	0.227	3.57E-06
23	ABCE1	Escherichia coli	0.321	1.94E-03	1407	EBF1	Pseudomonas oleovorans	-0.498	1.13E-06	2791	MMS22L	Pseudomonas oleovorans	-0.123	1.58E-03	4175	SF3A1	Ustilaginaceae	-0.155	1.62E-05
24	ABCF1	Kytococcaceae	0.395	2.93E-04	1408	EBF2	Kytococcaceae	0.176	5.15E-04	2792	MND1	Pseudomonas oleovorans	-0.441	1.82E-04	4176	SF3A2	Bradyrhizobium elkanii	-0.441	3.68E-04
25	ABCF3	Escherichia coli	-0.324	3.33E-07	1409	EBF2	Pseudomonas oleovorans	-0.343	3.42E-09	2793	MNT	Bradyrhizobium elkanii	-0.571	8.71E-05	4177	SF3A2	Escherichia coli	-0.208	2.89E-04
26	ABCG1	Paucibacter	-0.300	9.93E-14	1410	EBF3	Pseudomonas oleovorans	-0.418	5.89E-05	2794	MOB1B	Escherichia coli	0.488	4.16E-04	4178	SF3B2	Escherichia coli	-0.334	1.63E-03
27	ABCG5	Actinomyces naeslundii	-0.209	4.45E-28	1411	EBNA1BP2	Actinomyces naeslundii	-0.431	4.62E-20	2795	MOCS1	Leotiomycetes	-0.497	6.92E-04	4179	SFPQ	Actinomyces naeslundii	-0.094	4.25E-08
28	ABCG8	Kytococcaceae	-0.141	5.74E-06	1412	EBNA1BP2	Ustilaginaceae	-0.084	8.74E-04	2796	MOCS2	Bradyrhizobium elkanii	0.596	2.80E-03	4180	SFSWAP	Actinomyces naeslundii	-0.220	1.01E-21
29	ABHD1	Halomonadaceae	-0.275	3.78E-10	1413	ECH1	Dolosigranulum pigrum	-0.464	8.87E-04	2797	MOGS	Deinococcota	0.392	8.66E-03	4181	SFXN5	Escherichia coli	-0.483	2.61E-08
30	ABHD1	Schlegelella aquatica	-0.491	5.65E-04	1414	ECHDC1	Paucibacter	0.173	1.67E-03	2798	MOK	Actinomyces naeslundii	-0.285	4.39E-03	4182	SGCB	Bradyrhizobium elkanii	0.535	2.46E-03
31	ABHD11	Actinomyces naeslundii	-0.377	1.02E-24	1415	ECHDC2	Actinomyces naeslundii	-0.174	1.36E-12	2799	MOK	Kytococcaceae	0.059	2.23E-03	4183	SGIP1	Dolosigranulum pigrum	0.222	1.87E-05
32	ABHD12B	Pseudomonas oleovorans	-0.211	3.04E-04	1416	ECHS1	Dolosigranulum pigrum	-0.286	2.44E-07	2800	MORF4L1	Paucibacter	0.457	9.15E-13	4184	SGPP1	Escherichia coli	0.467	7.09E-49
33	ABHD13	Escherichia coli	0.270	3.82E-06	1417	ECI1	Dolosigranulum pigrum	-0.415	2.09E-25	2801	MORN4	Ustilaginaceae	0.093	2.55E-18	4185	SGPP2	Actinomyces naeslundii	-0.274	1.70E-04
34	ABHD14A	Enterobacterales	-0.530	1.73E-03	1418	EDC4	Bradyrhizobium elkanii	-0.560	5.54E-08	2802	MOSPD1	Escherichia coli	0.309	3.23E-04	4186	SGSM3	Escherichia coli	-0.303	5.90E-05
35	ABHD17A	Escherichia coli	-0.202	1.22E-11	1419	EDN1	Actinomyces naeslundii	-0.277	2.72E-05	2803	MOV10	Paucibacter	-0.335	3.17E-05	4187	SGTA	Escherichia coli	-0.256	3.09E-06
36	ABHD17C	Staphylococcus hominis	-0.424	9.13E-04	1420	EDN2	Escherichia coli	-0.643	3.58E-07	2804	MPG	Actinomyces naeslundii	0.272	5.60E-28	4188	SGTB	Halomonadaceae	0.357	1.49E-05
37	ABI1	Escherichia coli	0.540	9.45E-11	1421	EDNRA	Escherichia coli	0.449	1.38E-03	2805	MPG	Leotiomycetes	-0.561	5.85E-05	4189	SH2D1A	Escherichia coli	0.427	3.00E-04
38	ABL1	Alphaproteobacteria	0.634	2.64E-07	1422	EDNRB	Escherichia coli	0.519	2.87E-08	2806	MPHOSPH6	Dolosigranulum pigrum	0.470	7.11E-04	4190	SH3BGRL	Escherichia coli	0.695	2.56E-21
39	ABLIM1	Chromatiaceae	-0.178	8.73E-05	1423	EEA1	Escherichia coli	0.553	7.83E-08	2807	MPHOSPH8	Escherichia coli	-0.650	8.50E-06	4191	SH3BGRL2	Kytococcaceae	-0.229	8.88E-14
40	ABRACL	Cutibacterium	-0.332	2.24E-05	1424	EEF1A1	Ustilaginaceae	-0.296	1.42E-17	2808	MPPED1	Ustilaginaceae	0.192	2.48E-03	4192	SH3BP1	Methylobacterium sp. FF17	-0.682	1.92E-07
41	ABRACL	Staphylococcus epidermidis	0.358	4.48E-07	1425	EEF1AKMT2	Alphaproteobacteria	-0.608	2.91E-10	2809	MPRIP	Ustilaginaceae	-0.100	9.20E-16	4193	SH3BP5L	Escherichia coli	-0.443	1.13E-03
42	ACAA1	Dolosigranulum pigrum	-0.539	9.67E-06	1426	EEF1AKMT4	Enterobacterales	-0.599	6.50E-18	2810	MPZL1	Actinomyces naeslundii	0.418	7.25E-06	4194	SH3GL1	Bradyrhizobium elkanii	-0.528	9.45E-03
43	ACAD10	Bradyrhizobium elkanii	-0.466	4.03E-03	1427	EEPD1	Escherichia coli	-0.345	2.26E-03	2811	MRC1	Paucibacter	-0.238	2.46E-04	4195	SH3GLB2	Actinomyces naeslundii	-0.355	1.15E-23
44	ACADL	Gardnerella	0.364	6.25E-05	1428	EEPD1	Kytococcaceae	0.323	1.57E-03	2812	MREG	Actinomyces naeslundii	-0.227	1.83E-11	4196	SH3PXD2A	Actinomyces naeslundii	-0.231	2.30E-08
45	ACADS	Dolosigranulum pigrum	-0.336	1.95E-11	1429	EFCAB5	Kytococcaceae	0.171	5.33E-04	2813	MRFAP1	Paucibacter	0.405	2.00E-06	4197	SH3RF1	Chromatiaceae	0.324	3.88E-06
46	ACADVL	Actinomyces naeslundii	-0.186	3.10E-03	1430	EFCAB6	Kytococcaceae	0.279	5.00E-03	2814	MRFAP1	Ustilaginaceae	-0.237	6.76E-30	4198	SH3RF2	Ustilaginaceae	0.213	3.35E-03
47	ACADVL	Ustilaginaceae	-0.084	1.70E-04	1431	EFCAB8	Dolosigranulum pigrum	0.283	2.51E-04	2815	MRFAP1L1	Paucibacter	0.332	2.60E-05	4199	SH3TC2	Kytococcaceae	0.187	2.74E-11
48	ACAN	Kytococcaceae	0.083	1.32E-18	1432	EFHC1	Actinomyces naeslundii	-0.226	5.63E-09	2816	MRGPRF	Dolosigranulum pigrum	-0.346	8.89E-05	4200	SH3YL1	Actinomyces naeslundii	-0.256	4.20E-03
49	ACAN	Pseudomonas oleovorans	-0.481	4.20E-40	1433	EFHC2	Pseudomonas oleovorans	-0.433	1.01E-06	2817	MRM1	Escherichia coli	-0.473	8.87E-04	4201	SHANK1	Pseudomonas oleovorans	-0.314	1.90E-03
50	ACAP1	Actinomyces naeslundii	-0.446	8.16E-14	1434	EFNA1	Ascomycota	-0.610	4.17E-03	2818	MROH1	Bradyrhizobium elkanii	-0.610	3.14E-05	4202	SHARPIN	Enterobacterales	-0.462	1.00E-03
51	ACAP2	Escherichia coli	0.550	5.74E-12	1435	EFNA3	Dolosigranulum pigrum	0.217	1.53E-05	2819	MROH1	Escherichia coli	-0.231	1.32E-03	4203	SHC2	Escherichia coli	-0.231	7.46E-04

52	ACAP3	Escherichia coli	-0.229	4.04E-04	1436	EFNA5	Pseudomonas oleovorans	-0.486	4.29E-06	2820	MROH2B	Haemophilus	0.390	4.00E-04	4204	SHC3	Pseudomonas oleovorans	-0.391	3.66E-05
53	ACBD3	Actinomyces naeslundii	0.493	9.02E-26	1437	EFNB1	Paucibacter	-0.429	6.95E-03	2821	MROH7	Actinomyces naeslundii	-0.178	1.24E-06	4205	SHE	Paucibacter	-0.296	1.37E-03
54	ACBD4	Escherichia coli	-0.303	1.01E-03	1438	EFR3A	Escherichia coli	0.397	2.73E-06	2822	MRPL12	Dolosigranulum pigrum	-0.395	1.76E-06	4206	SHISA9	Staphylococcus epidermidis	-0.394	1.94E-03
55	ACBD5	Kytococcaceae	-0.103	9.59E-07	1439	EFS	Dolosigranulum pigrum	0.273	1.54E-03	2823	MRPL13	Bradyrhizobium elkanii	0.687	3.35E-12	4207	SHKBP1	Deinococcota	0.539	5.38E-04
56	ACD	Bradyrhizobium elkanii	-0.485	1.24E-05	1440	EGF	Pseudomonas oleovorans	-0.268	1.13E-09	2824	MRPL14	Lawsonellaceae	-0.655	3.72E-10	4208	SHMT1	Actinomyces naeslundii	0.402	1.48E-03
57	ACD	Escherichia coli	-0.210	1.43E-03	1441	EGR2	Actinomyces naeslundii	-0.332	1.84E-04	2825	MRPL2	Actinomyces naeslundii	-0.406	4.58E-08	4209	SHMT2	Ustilaginaceae	-0.192	5.53E-03
58	ACKR1	Streptococcaceae	0.388	7.19E-03	1442	EGR3	Dolosigranulum pigrum	0.299	2.06E-03	2826	MRPL2	Rhizobiaceae	0.378	3.37E-06	4210	SHOC2	Escherichia coli	0.339	3.77E-03
59	ACMSD	Cutibacterium acnes	-0.407	2.04E-03	1443	EHD1	Actinomyces naeslundii	0.273	3.61E-03	2827	MRPL24	Bacteria	-0.433	2.31E-03	4211	SHPK	Paucibacter	-0.403	9.45E-03
60	ACO1	Actinomyces naeslundii	0.583	4.24E-14	1444	EHD3	Paucibacter	-0.514	3.05E-10	2828	MRPL37	Actinomyces naeslundii	0.253	5.30E-05	4212	SHROOM1	Actinomyces naeslundii	-0.267	7.50E-18
61	ACO1	Streptococcaceae	-0.374	4.01E-04	1445	EHHADH	Kytococcaceae	-0.012	7.91E-04	2829	MRPL38	Paucibacter	-0.490	2.80E-04	4213	SI	Kytococcaceae	0.289	2.01E-03
62	ACO2	Actinomyces naeslundii	0.383	1.58E-05	1446	EHMT1	Bradyrhizobium elkanii	-0.657	1.64E-08	2830	MRPL40	Cutibacterium modestum	-0.697	1.56E-10	4214	SIAE	Actinomyces naeslundii	0.551	5.48E-04
63	ACO2	Dolosigranulum pigrum	-0.391	3.93E-03	1447	EHMT1	Methyloversatilis sp. RAC08	0.352	1.17E-04	2831	MRPL42	Escherichia coli	0.284	1.40E-18	4215	SIAH1	Dolosigranulum pigrum	0.454	2.30E-03
64	ACO2	Hyphomicrobiales	0.563	5.75E-03	1448	EHMT2	Deinococcota	0.462	3.17E-03	2832	MRPL43	Kytococcaceae	-0.546	1.53E-10	4216	SIAH1	Hyphomicrobiales	-0.421	6.36E-03
65	ACOT12	Ceratobasidiaceae	0.549	7.29E-03	1449	EHMT2	Escherichia coli	-0.181	3.27E-12	2833	MRPL45	Ustilaginaceae	-0.098	3.21E-03	4217	SIGLEC1	Paucibacter	-0.242	8.55E-06
66	ACOT2	Actinomyces naeslundii	0.105	2.22E-09	1450	EID1	Escherichia coli	0.376	3.68E-04	2834	MRPL9	Cytophagaceae	-0.445	3.91E-03	4218	SIGLEC10	Cutibacterium acnes	0.557	1.27E-03
67	ACOT7	Actinomyces naeslundii	0.131	2.45E-03	1451	EID2	Paucibacter	0.490	9.01E-04	2835	MRPS10	Actinomyces naeslundii	0.294	4.65E-17	4219	SIGLEC11	Paucibacter	-0.334	6.73E-05
68	ACOT8	Dolosigranulum pigrum	-0.508	4.59E-06	1452	EIF1	Paucibacter	0.521	1.70E-04	2836	MRPS10	Kytococcaceae	-0.151	2.03E-04	4220	SIGLEC5	Enterobacteriaceae	0.563	6.29E-05
69	ACOT8	Leotiomycetes	-0.539	7.75E-03	1453	EIF1AX	Cutibacterium modestum	-0.676	1.50E-12	2837	MRPS16	Bradyrhizobium elkanii	0.626	4.86E-06	4221	SIGLEC6	Kytococcaceae	0.248	6.53E-04
70	ACOXL	Pseudomonas oleovorans	-0.514	1.44E-10	1454	EIF1AY	Debaryomycetaceae	0.663	2.25E-07	2838	MRPS18A	Actinomyces naeslundii	0.360	4.75E-11	4222	SIGLEC7	Thermaceae	0.609	4.38E-05
71	ACP2	Actinomyces naeslundii	0.276	1.04E-11	1455	EIF1AY	Rhodoferax	0.244	6.09E-04	2839	MRPS18B	Ustilaginaceae	-0.163	2.19E-08	4223	SINHCAF	Dolosigranulum pigrum	0.416	9.12E-03
72	ACP5	Paucibacter	-0.602	3.52E-05	1456	EIF1B	Gordonia bronchialis	-0.512	2.41E-05	2840	MRPS18C	Achromobacter	0.686	3.17E-14	4224	SIRT1	Escherichia coli	0.368	1.00E-06
73	ACP6	Actinomyces naeslundii	-0.224	8.70E-04	1457	EIF1B	Paucibacter	0.394	3.13E-04	2841	MRPS2	Deinococcota	0.441	2.03E-03	4225	SIRT5	Actinomyces naeslundii	-0.180	5.45E-10
74	ACSBG1	Pseudomonas oleovorans	-0.310	1.97E-06	1458	EIF2B4	Actinomyces naeslundii	-0.255	1.78E-06	2842	MRPS28	Oxalobacteraceae	0.453	7.39E-03	4226	SIRT6	Escherichia coli	-0.334	9.92E-09
75	ACSBG2	Kytococcaceae	0.251	7.97E-10	1459	EIF2B4	Ustilaginaceae	0.168	2.09E-04	2843	MRPS30	Escherichia coli	0.374	1.80E-06	4227	SLAIN2	Halomonadaceae	0.398	1.34E-06
76	ACSM2A	Actinomyces naeslundii	-0.209	1.20E-09	1460	EIF3A	Dolosigranulum pigrum	-0.328	2.46E-04	2844	MRRF	Actinomyces naeslundii	-0.137	9.88E-25	4228	SLAMF7	Saccharomycetaceae	0.154	2.49E-03
77	ACSM2A	Ustilaginaceae	0.256	3.84E-04	1461	EIF3B	Escherichia coli	-0.582	4.17E-17	2845	MRTFB	Cytophagaceae	0.527	2.28E-05	4229	SLC10A1	Kytococcaceae	-0.126	2.85E-04
78	ACSS3	Rhizobiaceae	-0.232	9.43E-05	1462	EIF3B	Rhizobiaceae	0.376	1.21E-03	2846	MRTFB	Rhizobiaceae	-0.402	5.23E-03	4230	SLC11A1	Actinomyces naeslundii	-0.406	6.41E-05
79	ACTN4	Alphaproteobacteria	0.569	2.03E-06	1463	EIF3C	Kytococcaceae	0.415	5.84E-03	2847	MS4A6A	Paucibacter	-0.182	4.17E-07	4231	SLC11A1	Corynebacterium tuberculostea	0.487	3.93E-03
80	ACTR2	Escherichia coli	0.381	6.38E-03	1464	EIF3J	Phyllobacteriaceae	0.693	5.83E-06	2848	MS4A7	Paucibacter	-0.104	4.01E-03	4232	SLC11A1	Dolosigranulum pigrum	0.212	1.10E-10
81	ACTR3C	Plantactinospora	-0.623	4.35E-03	1465	EIF3L	Actinomyces naeslundii	0.161	9.58E-21	2849	MSANTD1	Pseudomonas oleovorans	-0.275	5.06E-06	4233	SLC12A1	Veillonella	-0.549	6.69E-03
82	ACVR2A	Plantactinospora	0.388	1.10E-03	1466	EIF3M	Cutibacterium modestum	-0.604	4.87E-07	2850	MSANTD2	Dolosigranulum pigrum	0.204	3.05E-05	4234	SLC12A5	Pseudomonas oleovorans	-0.151	5.80E-04
83	ACVRL1	Deinococci	0.597	1.57E-03	1467	EIF4A3	Actinomyces naeslundii	0.141	7.82E-03	2851	MSL1	Ustilaginaceae	0.151	2.34E-10	4235	SLC12A7	Escherichia coli	-0.140	2.08E-05
84	ACY1	Thermaceae	0.678	1.96E-09	1468	EIF4A3	Ustilaginaceae	-0.186	9.69E-04	2852	MSL2	Escherichia coli	0.400	1.56E-04	4236	SLC12A9	Escherichia coli	-0.369	2.10E-07
85	ACYP1	Actinomyces naeslundii	-0.440	4.52E-05	1469	EIF4EBP1	Paucibacter	-0.430	2.24E-03	2853	MSN	Actinomyces naeslundii	0.326	1.67E-04	4237	SLC13A1	Pseudomonas oleovorans	-0.175	1.53E-22
86	ACYP2	Escherichia coli	0.302	2.21E-08	1470	EIF4G1	Escherichia coli	-0.379	1.16E-58	2854	MSR1	Escherichia coli	0.620	1.33E-04	4238	SLC13A5	Ustilaginaceae	0.231	3.08E-04
87	ADAM10	Escherichia coli	0.427	2.48E-09	1471	EIF4G3	Dolosigranulum pigrum	0.415	8.84E-03	2855	MSRB1	Paucibacter	-0.485	1.41E-03	4239	SLC16A6	Cutibacterium acnes	0.417	1.83E-03
88	ADAM15	Escherichia coli	-0.310	2.86E-06	1472	EIF4G3	Ustilaginaceae	0.373	7.47E-09	2856	MST1	Ustilaginaceae	0.000	5.72E-07	4240	SLC17A4	Staphylococcus epidermidis	-0.373	1.07E-03
89	ADAM28	Dolosigranulum pigrum	0.436	1.24E-05	1473	EIF4H	Actinomyces naeslundii	0.292	5.49E-13	2857	MT1B	Ascomycota	-0.468	3.51E-05	4241	SLC17A7	Dolosigranulum pigrum	0.231	6.10E-07
90	ADAM32	Kytococcaceae	0.233	7.80E-05	1474	EIF6	Nocardioides	0.336	2.85E-03	2858	MTCH2	Actinomyces naeslundii	0.419	2.31E-11	4242	SLC1A3	Dolosigranulum pigrum	0.280	3.51E-03
91	ADAM32	Paucibacter	0.579	4.37E-05	1475	EIF6	Paucibacter	-0.427	4.12E-04	2859	MTCP1	Actinomyces naeslundii	-0.181	2.51E-07	4243	SLC1A5	Leotiomycetes	-0.344	1.88E-03
92	ADAMDEC1	Dolosigranulum pigrum	0.289	7.82E-05	1476	ELAC2	Alphaproteobacteria	0.514	3.75E-06	2860	MTCP1	Ustilaginaceae	0.172	5.49E-04	4244	SLC1A6	Pseudomonas oleovorans	-0.216	6.91E-27
93	ADAMTS14	Pseudomonas oleovorans	-0.179	3.43E-06	1477	ELAVL2	Pseudomonas oleovorans	-0.228	3.30E-12	2861	MTERF2	Ustilaginaceae	0.198	3.78E-10	4245	SLC22A1	Dolosigranulum pigrum	-0.450	2.27E-03
94	ADAMTS19	Pseudomonas oleovorans	-0.558	1.35E-43	1478	ELF4	Pseudomonadaceae	0.665	1.46E-04	2862	MTERF3	Thermaceae	-0.622	2.11E-04	4246	SLC22A14	Kytococcaceae	0.026	4.04E-05
95	ADAMTS20	Pseudomonas oleovorans	-0.164	2.09E-23	1479	ELFN1	Acinetobacter ursingii	0.600	3.20E-03	2863	MTFR1L	Actinomyces naeslundii	-0.362	6.37E-04	4247	SLC22A14	Pseudomonas oleovorans	-0.434	9.99E-05
96	ADAMTS3	Pseudomonas oleovorans	-0.423	6.64E-06	1480	ELFN1	Dolosigranulum	-0.409	7.74E-03	2864	MTG1	Actinomyces naeslundii	-0.253	1.74E-67	4248	SLC22A18	Escherichia coli	-0.331	2.89E-06
97	ADAMTS6	Pseudomonas oleovorans	-0.323	4.08E-07	1481	ELFN2	Pseudomonas oleovorans	0.005	3.07E-08	2865	MTG1	Escherichia coli	-0.505	7.91E-03	4249	SLC24A2	Pseudomonas oleovorans	-0.205	7.95E-04
98	ADAMTS7	Gordonia bronchialis	0.554	9.64E-04	1482	ELK3	Escherichia coli	0.577	3.86E-05	2866	MTG1	Ustilaginaceae	-0.136	1.30E-05	4250	SLC25A14	Actinomyces naeslundii	-0.293	1.15E-09
99	ADAMTS8	Kytococcaceae	0.188	1.49E-03	1483	ELMOD2	Bradyrhizobium elkanii	0.537	2.30E-04	2867	MTG2	Escherichia coli	-0.381	4.25E-04	4251	SLC25A15	Sphingomonadaceae	-0.406	3.30E-03
100	ADAMTSL1	Pseudomonas oleovorans	-0.502	1.04E-06	1484	ELMOD3	Halomonadaceae	-0.228	1.17E-03	2868	MTHFD1	Pseudomonas oleovorans	0.455	1.54E-08	4252	SLC25A18	Actinomyces naeslundii	-0.375	2.78E-166
101	ADAMTSL2	Bradyrhizobium elkanii	-0.444	4.43E-05	1485	ELMOD3	Ustilaginaceae	0.049	5.65E-04	2869	MTHFS	Dolosigranulum pigrum	-0.597	3.06E-09	4253	SLC25A20	Kytococcaceae	-0.128	4.67E-38
102	ADAMTSL2	Escherichia coli	-0.283	1.86E-05	1486	ELOC	Cutibacterium modestum	-0.741	9.30E-08	2870	MTHFSD	Escherichia coli	-0.415	3.99E-05	4254	SLC25A21	Colletotrichum lupini	-0.442	7.74E-03
103	ADAMTSL5	Escherichia coli	-0.513	9.79E-07	1487	ELOVL7	Dolosigranulum pigrum	0.347	3.51E-06	2871	MTMR6	Escherichia coli	0.301	2.38E-03	4255	SLC25A22	Paucibacter	-0.429	6.14E-05
104	ADAT2	Ustilaginaceae	0.210	6.56E-14	1488	EMC1	Actinomyces naeslundii	0.407	1.63E-03	2872	MTPN	Escherichia coli	0.311	1.63E-03	4256	SLC25A25	Actinomyces naeslundii	-0.481	8.97E-15
105	ADCK1	Actinomyces naeslundii	-0.344	3.12E-07	1489	EMC2	Alphaproteobacteria	-0.535	5.79E-04	2873	MTPN	Ustilaginaceae	-0.017	2.26E-31	4257	SLC25A25	Leptotrichiaceae	0.131	1.78E-12

106	ADCK5	Escherichia coli	-0.217	1.31E-05	1490	EMC2	Escherichia coli	0.193	1.34E-04	2874	MTRF1	Actinomyces naeslundii	-0.174	3.75E-08	4258	SLC25A25	Methylorubrum	0.451	1.97E-20
107	ADCY2	Kytococcaceae	0.340	9.09E-05	1491	EMC3	Actinomyces naeslundii	0.457	4.39E-15	2875	MTRF1L	Bradyrhizobium elkanii	0.590	6.32E-04	4259	SLC25A26	Staphylococcus hominis	-0.416	7.75E-11
108	ADCY8	Kytococcaceae	0.290	7.31E-08	1492	EMC7	Bradyrhizobium elkanii	0.669	1.86E-09	2876	MTUS1	Helotiales	0.390	2.73E-07	4260	SLC25A27	Actinomyces naeslundii	-0.179	2.77E-08
109	ADGB	Pseudomonas oleovorans	-0.270	8.10E-09	1493	EMC8	Acinetobacter ursingii	-0.639	2.36E-08	2877	MTX2	Thermaceae	-0.711	1.56E-03	4261	SLC25A27	Ustilaginaceae	0.153	7.57E-03
110	ADGRA3	Ustilaginaceae	0.254	2.55E-08	1494	EMCN	Methyloversatilis sp. RAC08	-0.367	4.11E-05	2878	MUC16	Acinetobacter baumannii	0.374	1.94E-03	4262	SLC25A28	Actinomyces naeslundii	-0.339	2.47E-13
111	ADGRB1	Pseudomonas oleovorans	-0.318	4.56E-07	1495	EME1	Pseudomonas oleovorans	-0.202	2.26E-04	2879	MUC16	Nostocales	0.589	1.66E-03	4263	SLC25A29	Actinomyces naeslundii	-0.420	7.34E-25
112	ADGRB2	Kytococcaceae	0.217	2.69E-04	1496	EME2	Actinomyces naeslundii	-0.347	7.81E-14	2880	MUC16	Paucibacter	0.531	2.94E-04	4264	SLC25A32	Escherichia coli	0.392	2.29E-04
113	ADGRB2	Pseudomonas oleovorans	-0.349	5.10E-10	1497	EML5	Kytococcaceae	0.228	3.79E-06	2881	MUC16	Pseudomonas oleovorans	-0.386	2.53E-07	4265	SLC25A36	Actinomyces naeslundii	-0.430	4.43E-09
114	ADGRF1	Pseudomonas oleovorans	-0.264	9.44E-33	1498	ENDOG	Dolosigranulum pigrum	-0.483	5.25E-05	2882	MUC16	Streptomyces sp. T12	0.283	1.72E-03	4266	SLC25A37	Actinomyces naeslundii	-0.316	5.77E-05
115	ADGRG7	Escherichia coli	0.317	1.35E-03	1499	ENDOG	Leotiomycetes	-0.602	7.08E-04	2883	MUC17	Acinetobacter baumannii	0.565	9.13E-03	4267	SLC25A39	Paucibacter	-0.409	5.07E-03
116	ADH1B	Kytococcaceae	-0.135	3.77E-03	1500	ENG	Paucibacter	-0.348	6.36E-04	2884	MUC17	Pseudomonas oleovorans	-0.391	4.01E-16	4268	SLC25A40	Escherichia coli	0.503	4.06E-11
117	ADH6	Kytococcaceae	0.002	2.09E-10	1501	ENO1	Actinomyces naeslundii	0.456	7.55E-06	2885	MUC19	Kytococcaceae	0.172	1.98E-08	4269	SLC25A41	Alcaligenaceae	-0.359	1.94E-04
118	ADK	Bradyrhizobium elkanii	0.686	4.89E-03	1502	ENOSF1	Actinomyces naeslundii	-0.300	2.68E-24	2886	MUC19	Pseudomonas oleovorans	-0.373	5.10E-17	4270	SLC25A44	Dolosigranulum pigrum	-0.496	2.48E-03
119	ADM5	Ustilaginaceae	-0.017	3.28E-10	1503	ENOX2	Actinomyces naeslundii	0.379	2.53E-04	2887	MUC2	Kytococcaceae	0.132	5.01E-05	4271	SLC25A46	Escherichia coli	0.335	3.28E-06
120	ADPGK	Actinomyces naeslundii	-0.353	1.41E-18	1504	ENPP5	Dolosigranulum pigrum	0.306	1.97E-06	2888	MUC2	Pseudomonas oleovorans	-0.356	3.81E-13	4272	SLC25A47	Paucibacter	-0.394	2.94E-14
121	ADPRH	Dolosigranulum pigrum	0.398	1.41E-03	1505	ENSA	Cutibacterium modestum	-0.689	2.65E-08	2889	MUC4	Pseudomonas oleovorans	-0.169	1.02E-05	4273	SLC25A5	Actinomyces naeslundii	0.223	6.75E-25
122	ADPRM	Leptotrichiaceae	0.210	3.27E-04	1506	ENTPD3	Pseudomonas oleovorans	-0.512	1.47E-08	2890	MUC5AC	Kytococcaceae	0.085	6.87E-03	4274	SLC25A52	Actinomyces naeslundii	-0.098	7.54E-23
123	ADRA2A	Kytococcaceae	0.232	6.90E-03	1507	ENTPD6	Escherichia coli	-0.461	2.53E-10	2891	MUL1	Actinomyces naeslundii	0.232	1.46E-15	4275	SLC25A52	Ustilaginaceae	0.243	8.80E-04
124	ADSL	Achromobacter	0.525	2.94E-05	1508	ENTPD8	Escherichia coli	-0.459	1.75E-33	2892	MUSTN1	Ustilaginaceae	0.154	3.59E-05	4276	SLC26A11	Bradyrhizobium elkanii	-0.579	4.24E-04
125	ADSS2	Escherichia coli	0.382	1.79E-05	1509	ENY2	Cutibacterium modestum	-0.647	1.84E-05	2893	MUTYH	Actinomyces naeslundii	-0.339	7.97E-09	4277	SLC26A3	Dolosigranulum pigrum	0.178	7.50E-03
126	AEBP2	Alphaproteobacteria	-0.430	6.20E-05	1510	EP300	Acinetobacter ursingii	0.815	3.63E-19	2894	MVB12B	Escherichia coli	-0.198	5.00E-08	4278	SLC26A7	Pseudomonas oleovorans	-0.377	1.66E-05
127	AFAP1L1	Paucibacter	-0.326	2.73E-04	1511	EPB41	Yersiniaceae	-0.657	1.70E-05	2895	MVD	Escherichia coli	-0.570	1.79E-07	4279	SLC27A1	Deinococcota	0.431	3.00E-04
128	AFAP1L2	Dolosigranulum pigrum	0.271	2.46E-06	1512	EPB41L4A	Dolosigranulum pigrum	0.374	9.12E-09	2896	MXD3	Actinomyces naeslundii	-0.185	1.93E-05	4280	SLC27A1	Paucibacter	-0.292	5.06E-06
129	AFDN	Actinomyces naeslundii	0.054	8.87E-11	1513	EPB42	Pseudomonas oleovorans	-0.285	1.86E-06	2897	MXI1	Escherichia coli	0.439	1.08E-03	4281	SLC28A1	Halomonadaceae	-0.073	5.52E-03
130	AFF1	Acinetobacter ursingii	0.771	3.95E-04	1514	EPG5	Ottowia	0.563	1.93E-05	2898	MXRA8	Actinomyces naeslundii	-0.338	3.86E-08	4282	SLC28A2	Clavicipitaceae	-0.376	7.72E-03
131	AFG3L2	Actinomyces naeslundii	0.282	7.81E-04	1515	EPHA10	Acinetobacter baumannii	0.150	6.45E-03	2899	MYBBP1A	Bradyrhizobium elkanii	-0.590	9.85E-03	4283	SLC29A2	Actinomyces naeslundii	-0.270	1.06E-19
132	AGAP3	Escherichia coli	-0.201	2.45E-03	1516	EPHA10	Kytococcaceae	0.247	3.84E-07	2900	MYBBP1A	Escherichia coli	-0.197	3.57E-07	4284	SLC2A13	Escherichia coli	0.383	5.61E-09
133	AGAP4	Ustilaginaceae	0.165	2.22E-11	1517	EPHA10	Pseudomonas oleovorans	-0.200	1.23E-06	2901	MYBPC3	Pseudomonas oleovorans	-0.389	4.76E-16	4285	SLC2A2	Kytococcaceae	-0.013	2.80E-13
134	AGBL1	Pseudomonas oleovorans	-0.439	2.83E-153	1518	EPHA3	Micromonosporales	0.388	2.76E-04	2902	MYBPH	Ascomycota	-0.253	2.72E-04	4286	SLC2A8	Escherichia coli	-0.463	2.96E-03
135	AGBL2	Ustilaginaceae	0.315	2.06E-10	1519	EPHA5	Pseudomonas oleovorans	-0.316	1.63E-10	2903	MYBPH	Kytococcaceae	0.257	1.28E-06	4287	SLC30A7	Bradyrhizobium elkanii	0.505	2.79E-03
136	AGBL5	Leotiomycetes	-0.586	1.20E-03	1520	EPHA6	Pseudomonas oleovorans	-0.351	4.43E-16	2904	MYCBPAP	Kytococcaceae	0.039	8.46E-05	4288	SLC31A1	Actinomyces naeslundii	0.558	8.44E-23
137	AGER	Actinomyces naeslundii	-0.292	1.20E-47	1521	EPHA7	Kytococcaceae	0.271	4.31E-03	2905	MYCN	Schlegelella aquatica	0.314	1.95E-03	4289	SLC35A3	Escherichia coli	0.355	5.22E-07
138	AGGF1	Bradyrhizobium elkanii	0.669	8.97E-05	1522	EPHA7	Pseudomonas oleovorans	-0.192	5.33E-06	2906	MYCT1	Escherichia coli	0.573	4.96E-03	4290	SLC35B3	Cutibacterium modestum	-0.676	1.76E-08
139	AGK	Halomonadaceae	-0.252	1.91E-08	1523	EPHX1	Actinomyces naeslundii	0.403	2.54E-07	2907	MYCT1	Paucibacter	-0.261	8.72E-03	4291	SLC35B3	Leotiomycetes	0.446	4.08E-03
140	AGK	Ustilaginaceae	0.151	1.53E-07	1524	EPN2	Ceratobasidiaceae	-0.546	5.17E-03	2908	MYDGF	Actinomyces naeslundii	-0.008	6.09E-14	4292	SLC35C2	Escherichia coli	-0.482	1.24E-05
141	AGMAT	Ottowia	-0.668	2.14E-05	1525	EPOR	Actinomyces naeslundii	-0.498	1.86E-38	2909	MYH10	Hyphomicrobiaceae	0.533	9.66E-04	4293	SLC35D1	Actinomyces naeslundii	0.492	5.50E-33
142	AGMO	Escherichia coli	0.226	6.49E-15	1526	EPS15	Escherichia coli	0.529	1.33E-03	2910	MYH13	Kytococcaceae	0.231	5.04E-06	4294	SLC35F2	Dolosigranulum pigrum	0.270	5.59E-04
143	AGPAT4	Actinomyces naeslundii	-0.481	2.85E-05	1527	EPS15L1	Bradyrhizobium elkanii	-0.405	1.40E-06	2911	MYH13	Pseudomonas oleovorans	-0.336	4.62E-05	4295	SLC35F4	Kytococcaceae	0.247	4.19E-09
144	AGRN	Escherichia coli	-0.236	1.40E-06	1528	EPS15L1	Escherichia coli	-0.351	7.51E-06	2912	MYH15	Pseudomonas oleovorans	-0.436	6.13E-25	4296	SLC35F4	Pseudomonas oleovorans	-0.380	5.68E-13
145	AGXT	Escherichia coli	-0.265	1.65E-07	1529	EPS8L3	Kytococcaceae	0.223	2.51E-03	2913	MYH3	Kytococcaceae	-0.005	3.93E-03	4297	SLC35F5	Escherichia coli	0.294	2.09E-05
146	AGXT	Paucibacter	-0.370	2.48E-07	1530	ERAS	Dolosigranulum pigrum	0.218	1.60E-04	2914	MYH4	Kytococcaceae	0.324	1.89E-04	4298	SLC35G2	Pseudomonas oleovorans	-0.353	1.36E-04
147	AHI1	Actinomyces naeslundii	-0.164	5.22E-11	1531	ERC1	Actinomyces naeslundii	0.343	2.61E-27	2915	MYH7B	Actinomyces naeslundii	-0.320	1.88E-15	4299	SLC36A1	Gordonia bronchialis	0.568	4.06E-09
148	AHNAK2	Pseudomonas oleovorans	-0.327	1.34E-03	1532	ERC1	Dolosigranulum pigrum	-0.401	1.80E-37	2916	MYL12B	Cutibacterium modestum	-0.683	3.24E-10	4300	SLC36A4	Dolosigranulum pigrum	0.457	6.81E-04
149	AHR	Escherichia coli	0.451	1.24E-04	1533	ERC2	Pseudomonas oleovorans	-0.228	1.04E-46	2917	MYL5	Actinomyces naeslundii	-0.406	8.05E-104	4301	SLC37A2	Paucibacter	-0.379	2.67E-21
150	AHSG	Actinomyces naeslundii	0.297	3.57E-20	1534	ERCC6L2	Rhizobiaceae	-0.399	7.80E-08	2918	MYO15B	Actinomyces naeslundii	-0.280	7.08E-17	4302	SLC37A2	Thermaceae	0.672	4.88E-03
151	AIF1	Escherichia coli	0.558	1.72E-05	1535	ERCC8	Rhizobiaceae	-0.344	6.74E-03	2919	MYO18B	Kytococcaceae	0.267	5.37E-05	4303	SLC37A3	Actinomyces naeslundii	-0.245	1.32E-03
152	AIFM1	Actinomyces naeslundii	0.430	8.19E-28	1536	EREG	Pseudomonas oleovorans	-0.394	9.41E-06	2920	MYO18B	Pseudomonas oleovorans	-0.178	2.34E-10	4304	SLC37A3	Dolosigranulum pigrum	0.303	9.59E-03
153	AIFM2	Escherichia coli	-0.585	2.19E-05	1537	ERF	Escherichia coli	-0.203	1.39E-04	2921	MYO19	Ustilaginaceae	0.009	8.50E-03	4305	SLC37A3	Ustilaginaceae	0.241	7.72E-03
154	AIP	Helotiales	-0.545	1.07E-03	1538	ERGIC1	Actinomyces naeslundii	0.438	9.17E-05	2922	MYO1H	Pseudomonas oleovorans	-0.332	8.62E-16	4306	SLC38A10	Escherichia coli	-0.304	2.57E-06
155	AJM1	Escherichia coli	-0.368	1.61E-07	1539	ERGIC2	Bradyrhizobium elkanii	0.634	4.64E-06	2923	MYO3A	Kytococcaceae	0.258	7.98E-14	4307	SLC38A11	Actinomyces naeslundii	-0.293	1.07E-06
156	AK4	Kytococcaceae	-0.134	1.36E-03	1540	ERH	Escherichia coli	0.386	1.39E-06	2924	MYO5A	Ottowia	0.565	1.14E-03	4308	SLC38A6	Dolosigranulum pigrum	0.453	6.50E-04
157	AK6	Dolosigranulum	0.303	2.98E-03	1541	ERICH1	Actinomyces naeslundii	-0.325	2.90E-13	2925	MYO6	Thermaceae	-0.614	6.06E-05	4309	SLC39A1	Ustilaginaceae	-0.049	1.21E-05
158	AKAP11	Escherichia coli	0.532	1.24E-04	1542	ERICH5	Streptococcus oralis	0.482	1.23E-04	2926	MYO9B	Bradyrhizobium elkanii	-0.625	1.31E-05	4310	SLC39A3	Dolosigranulum pigrum	-0.424	7.18E-03
159	AKAP12	Paucibacter	-0.215	7.65E-03	1543	ERLIN2	Actinomyces naeslundii	0.343	6.99E-17	2927	MYOM3	Kytococcaceae	0.187	1.13E-04	4311	SLC39A6	Actinomyces naeslundii	0.375	4.02E-04

160	AKAP13	Chromatiaceae	-0.137	2.08E-04	1544	ERMARD	Actinomyces naeslundii	-0.186	1.34E-06	2928	MYORG	Actinomyces naeslundii	0.358	3.40E-08	4312	SLC39A9	Actinomyces naeslundii	0.438	4.57E-08
161	AKAP8L	Staphylococcus epidermidis	-0.454	1.04E-09	1545	ERMN	Pseudomonas oleovorans	-0.355	2.03E-04	2929	MYPN	Pseudomonas oleovorans	-0.366	7.58E-26	4313	SLC39A9	Kytococcaceae	-0.005	1.07E-08
162	AKNA	Actinomyces naeslundii	-0.306	3.04E-10	1546	ERN2	Kytococcaceae	0.219	7.36E-03	2930	MYRIP	Haemophilus	-0.332	5.41E-04	4314	SLC3A2	Ceratobasidiaceae	-0.326	3.84E-04
163	AKR1A1	Rhodobacterales	0.413	2.09E-03	1547	ERO1A	Escherichia coli	0.467	4.62E-07	2931	MYT1L	Kytococcaceae	0.146	3.45E-06	4315	SLC40A1	Actinomyces naeslundii	0.140	1.86E-16
164	AKR1D1	Kytococcaceae	-0.141	5.78E-05	1548	ERP29	Bradyrhizobium elkanii	0.617	4.34E-08	2932	MYT1L	Pseudomonas oleovorans	-0.287	1.60E-06	4316	SLC41A2	Escherichia coli	0.414	1.02E-06
165	AKR7A2	Actinomyces naeslundii	0.288	1.81E-06	1549	ERRFI1	Actinomyces naeslundii	-0.265	1.67E-04	2933	MYZAP	Sordariales	0.516	5.98E-03	4317	SLC41A3	Micrococcales	0.436	7.87E-06
166	AKR7A2	Dolosigranulum pigrum	-0.449	3.92E-03	1550	ERRFI1	Leptotrichiaceae	0.280	1.42E-04	2934	MZB1	Dolosigranulum pigrum	0.184	3.79E-03	4318	SLC43A2	Paucibacter	-0.353	3.98E-03
167	AKR7A3	Dolosigranulum pigrum	-0.285	4.50E-04	1551	ESD	Gordonia bronchialis	-0.494	1.09E-11	2935	MZT1	Escherichia coli	0.393	9.57E-18	4319	SLC44A2	Paucibacter	-0.368	7.53E-04
168	AKR7L	Actinomyces naeslundii	-0.157	1.74E-14	1552	ESR2	Kytococcaceae	0.178	1.83E-09	2936	MZT1	Paucibacter	0.500	1.49E-04	4320	SLC46A3	Kytococcaceae	-0.146	2.67E-04
169	AKT2	Escherichia coli	-0.309	1.33E-06	1553	ESS2	Escherichia coli	-0.363	6.78E-18	2937	N4BP2	Escherichia coli	0.351	5.96E-03	4321	SLC49A3	Escherichia coli	-0.281	4.71E-03
170	AKTIP	Bradyrhizobium elkanii	0.557	1.05E-08	1554	ETFRF1	Bradyrhizobium elkanii	0.528	4.87E-03	2938	N4BP2L1	Actinomyces naeslundii	-0.363	6.51E-09	4322	SLC49A3	Gordonia bronchialis	0.527	1.24E-05
171	ALCAM	Ustilaginaceae	0.500	1.48E-03	1555	ETFRF1	Cutibacterium modestum	-0.562	1.39E-08	2939	N4BP2L2	Actinomyces naeslundii	-0.210	4.51E-65	4323	SLC49A4	Actinomyces naeslundii	0.109	1.92E-04
172	ALDH16A1	Escherichia coli	-0.262	6.88E-03	1556	ETFRF1	Escherichia coli	0.327	4.99E-26	2940	N4BP2L2	Ustilaginaceae	0.234	9.06E-10	4324	SLC4A1	Pseudomonas oleovorans	-0.393	1.74E-08
173	ALDH1L2	Kytococcaceae	0.251	5.84E-03	1557	ETNK1	Alphaproteobacteria	-0.562	2.96E-09	2941	N4BP3	Pseudomonas oleovorans	-0.321	7.67E-07	4325	SLC4A2	Deinococcota	0.384	3.91E-03
174	ALDH3A1	Escherichia coli	-0.678	1.79E-06	1558	ETV5	Schlegelella aquatica	0.609	1.33E-05	2942	NAA11	Pseudomonas oleovorans	-0.155	4.25E-22	4326	SLC4A2	Escherichia coli	-0.395	3.62E-06
175	ALDH4A1	Actinomyces naeslundii	0.301	1.01E-04	1559	EVA1A	Cutibacterium modestum	-0.605	2.38E-17	2943	NAA20	Bradyrhizobium elkanii	0.561	8.10E-03	4327	SLC4A8	Pseudomonas oleovorans	-0.358	2.08E-03
176	ALDH7A1	Dolosigranulum pigrum	-0.372	2.27E-03	1560	EVI2A	Escherichia coli	0.436	2.13E-04	2944	NAA20	Cutibacterium modestum	-0.723	2.54E-10	4328	SLC52A2	Escherichia coli	-0.357	2.32E-03
177	ALDOB	Kytococcaceae	-0.100	1.13E-04	1561	EVI5	Acidovorax temperans	-0.206	4.42E-08	2945	NAA40	Actinomyces naeslundii	-0.362	2.63E-15	4329	SLC5A1	Kytococcaceae	0.251	4.21E-03
178	ALG1	Actinomyces naeslundii	0.320	5.44E-08	1562	EVI5L	Escherichia coli	-0.372	1.79E-27	2946	NAA60	Escherichia coli	-0.423	4.73E-82	4330	SLC5A4	Dolosigranulum pigrum	0.193	2.97E-19
179	ALG10B	Escherichia coli	0.304	5.87E-04	1563	EVPL	Kytococcaceae	0.292	3.93E-06	2947	NACAD	Pseudomonas oleovorans	-0.278	1.74E-04	4331	SLC5A6	Leptotrichiaceae	-0.240	1.19E-03
180	ALG11	Dolosigranulum pigrum	-0.306	9.38E-03	1564	EWSR1	Paucibacter	-0.496	3.67E-04	2948	NADK2	Escherichia coli	0.294	2.64E-07	4332	SLC5A7	Pseudomonas oleovorans	-0.441	3.06E-06
181	ALG13	Actinomyces naeslundii	-0.262	1.30E-04	1565	EXOC3	Escherichia coli	-0.281	8.84E-10	2949	NADSYN1	Ustilaginaceae	0.145	2.20E-18	4333	SLC66A2	Escherichia coli	-0.358	1.24E-05
182	ALG3	Actinomyces naeslundii	0.371	4.87E-04	1566	EXOC4	Actinomyces naeslundii	0.396	1.66E-04	2950	NAGLU	Nostocales	-0.406	2.46E-03	4334	SLC66A3	Escherichia coli	0.256	1.58E-12
183	ALG6	Dolosigranulum pigrum	0.552	1.75E-05	1567	EXOC8	Escherichia coli	0.468	1.79E-07	2951	NAGLU	Paucibacter	-0.501	1.44E-03	4335	SLC6A16	Actinomyces naeslundii	-0.417	9.54E-14
184	ALK	Escherichia coli	-0.598	2.46E-13	1568	EXOSC5	Actinomyces	0.204	1.70E-04	2952	NAGPA	Deinococcota	0.414	1.98E-03	4336	SLC6A2	Escherichia coli	-0.495	3.11E-50
185	ALKBH2	Leptotrichiaceae	0.248	1.00E-03	1569	EXOSC8	Actinomyces naeslundii	-0.366	4.61E-05	2953	NALF1	Pseudomonas oleovorans	-0.444	2.93E-04	4337	SLC6A4	Pseudomonas oleovorans	-0.499	5.62E-15
186	ALKBH4	Dolosigranulum pigrum	-0.460	1.97E-04	1570	EYA1	Pseudomonas oleovorans	-0.373	8.88E-14	2954	NALF2	Kytococcaceae	0.276	1.12E-03	4338	SLC6A9	Pseudomonas oleovorans	-0.243	5.74E-03
187	ALKBH5	Actinomyces naeslundii	0.178	2.37E-12	1571	EYS	Pseudomonas oleovorans	-0.222	9.46E-07	2955	NANS	Actinomyces naeslundii	0.312	1.04E-08	4339	SLC6A9	Streptomyces sp. T12	0.401	1.88E-03
188	ALKBH6	Actinomyces naeslundii	-0.252	1.33E-10	1572	F13B	Kytococcaceae	-0.191	3.36E-04	2956	NAPRT	Escherichia coli	-0.182	5.27E-06	4340	SLC7A1	Ottowia	0.534	4.31E-03
189	ALS2CL	Ustilaginaceae	-0.034	3.91E-13	1573	F2	Actinomyces naeslundii	0.147	4.73E-03	2957	NAPRT	Paucibacter	-0.368	6.05E-03	4341	SLC7A11	Dolosigranulum pigrum	0.193	6.82E-03
190	ALX3	Ceratobasidiaceae	-0.275	1.08E-23	1574	F2	Dolosigranulum pigrum	-0.400	1.17E-03	2958	NAPSA	Ustilaginaceae	0.227	1.93E-04	4342	SLC7A11	Pseudomonas oleovorans	-0.371	4.55E-04
191	AMBP	Actinomyces naeslundii	0.188	4.06E-06	1575	F3	Actinomyces naeslundii	-0.436	9.53E-04	2959	NASP	Actinomyces naeslundii	-0.382	2.63E-15	4343	SLC7A14	Pseudomonas oleovorans	-0.090	1.86E-07
192	AMBRA1	Alphaproteobacteria	0.412	7.98E-09	1576	F8	Actinomyces naeslundii	0.368	1.78E-03	2960	NAT14	Staphylococcaceae	0.538	3.33E-03	4344	SLC7A6	Ottowia	0.495	9.46E-04
193	AMBRA1	Escherichia coli	-0.348	2.70E-11	1577	F8	Oscillatoriales	0.302	9.20E-05	2961	NAT9	Ustilaginaceae	0.150	7.07E-04	4345	SLC7A8	Paucibacter	-0.302	7.71E-19
194	AMBRA1	Paucibacter	-0.420	6.02E-13	1578	FA2H	Kytococcaceae	0.261	1.67E-10	2962	NATD1	Leotiomycetes	-0.617	1.76E-03	4346	SLC9A2	Paucibacter	0.504	1.40E-04
195	AMFR	Actinomyces naeslundii	0.447	2.38E-21	1579	FAAP100	Escherichia coli	-0.228	8.59E-76	2963	NAV3	Pseudomonas oleovorans	-0.365	1.74E-05	4347	SLC9A2	Pseudomonas oleovorans	-0.306	5.82E-06
196	AMH	Escherichia coli	-0.302	8.53E-04	1580	FAAP20	Escherichia coli	-0.355	3.35E-07	2964	NAXD	Actinomyces naeslundii	-0.162	3.05E-15	4348	SLC9A3	Escherichia coli	-0.232	3.06E-06
197	AMH	Kytococcaceae	0.455	5.64E-70	1581	FAF1	Actinomyces naeslundii	0.344	4.09E-04	2965	NAXE	Ustilaginaceae	-0.139	2.34E-06	4349	SLC9A5	Kytococcaceae	0.174	4.66E-05
198	AMIGO3	Escherichia coli	-0.307	8.85E-04	1582	FAH	Dolosigranulum pigrum	-0.427	1.38E-07	2966	NBAS	Actinomyces naeslundii	0.548	1.08E-15	4350	SLC9A9	Pseudomonadaceae	0.654	4.20E-19
199	AMPD2	Halomonadaceae	-0.251	1.69E-06	1583	FAHD1	Bradyrhizobium elkanii	0.555	2.08E-03	2967	NBPF15	Actinomyces	-0.426	1.63E-04	4351	SLC9B2	Kytococcaceae	-0.086	3.36E-04
200	AMPD2	Paucibacter	-0.456	3.51E-05	1584	FAHD2A	Halomonadaceae	-0.232	6.06E-03	2968	NBPF26	Chromatiaceae	-0.385	1.12E-07	4352	SLC9C1	Kytococcaceae	0.130	1.05E-09
201	AMY2B	Actinomyces naeslundii	-0.125	2.82E-11	1585	FAHD2A	Ustilaginaceae	0.113	7.89E-04	2969	NBPF3	Caudoviricetes	0.404	3.30E-15	4353	SLC9C1	Pseudomonas oleovorans	-0.454	8.98E-17
202	ANAPC1	Acinetobacter ursingii	0.678	2.44E-07	1586	FAIM2	Pseudomonas oleovorans	-0.297	1.86E-04	2970	NCAN	Pseudomonas oleovorans	-0.189	5.97E-04	4354	SLCO1B3-SL(	Actinomyces naeslundii	-0.414	8.11E-07
203	ANAPC4	Actinomyces naeslundii	-0.210	4.28E-03	1587	FAM110C	Dolosigranulum pigrum	0.399	1.58E-05	2971	NCAPD3	Chromatiaceae	-0.189	3.70E-03	4355	SLCO1C1	Pseudomonas oleovorans	-0.337	1.29E-13
204	ANAPC4	Ustilaginaceae	0.221	2.82E-03	1588	FAM111A	Rhizobiaceae	-0.425	2.73E-03	2972	NCAPD3	Cutibacterium modestum	0.590	1.47E-08	4356	SLCO2A1	Staphylococcus hominis	0.446	8.70E-03
205	ANGPT4	Pseudomonas oleovorans	-0.254	6.24E-06	1589	FAM117B	Escherichia coli	0.397	1.03E-03	2973	NCAPG	Pseudomonas oleovorans	-0.188	2.24E-05	4357	SLCO2B1	Chromatiaceae	-0.137	3.99E-04
206	ANGPTL6	Thermaceae	0.675	1.99E-03	1590	FAM118A	Actinomyces naeslundii	-0.310	2.67E-16	2974	NCAPH2	Actinomyces naeslundii	-0.332	4.49E-10	4358	SLCO4A1	Kytococcaceae	0.219	1.76E-04
207	ANKDD1A	Actinomyces naeslundii	-0.284	1.82E-07	1591	FAM131A	Actinomyces naeslundii	-0.562	2.13E-08	2975	NCAPH2	Escherichia coli	-0.333	3.13E-04	4359	SLCO5A1	Pseudomonas oleovorans	-0.293	1.48E-07
208	ANKDD1A	Thermaceae	-0.767	2.40E-07	1592	FAM13C	Rhodoferax	0.302	7.60E-06	2976	NCF1	Thermaceae	0.669	1.97E-08	4360	SLF1	Thermaceae	-0.694	1.47E-03
209	ANKDD1B	Pseudomonas oleovorans	-0.302	4.14E-04	1593	FAM161A	Dolosigranulum pigrum	0.379	2.24E-04	2977	NCF4	Cutibacterium acnes	0.444	3.00E-03	4361	SLFNL1	Escherichia coli	-0.366	8.90E-03
210	ANKFN1	Pseudomonas oleovorans	-0.253	8.02E-11	1594	FAM163B	Rhizobiaceae	0.386	7.37E-04	2978	NCK1	Escherichia coli	0.329	2.00E-07	4362	SLIT1	Kytococcaceae	0.069	7.47E-07
211	ANKHD1	Actinomyces naeslundii	-0.336	6.66E-11	1595	FAM166B	Actinomyces naeslundii	-0.404	7.31E-05	2979	NCK2	Ottowia	0.504	1.83E-04	4363	SMAD5	Escherichia coli	0.399	8.16E-06
212	ANKHD1-EIF4	Ustilaginaceae	0.330	1.98E-07	1596	FAM168A	Actinomyces naeslundii	0.430	9.59E-05	2980	NCKAP1	Escherichia coli	0.281	6.20E-10	4364	SMAD6	Thermales	0.599	6.79E-04
213	ANKIB1	Escherichia coli	0.423	5.18E-29	1597	FAM174A	Escherichia coli	0.357	3.65E-04	2981	NCKAP5L	Escherichia coli	-0.210	5.89E-04	4365	SMAP1	Escherichia coli	-0.367	2.40E-04

214	ANKK1	Kytococcaceae	0.143	8.14E-04	1598	FAM177B	Kytococcaceae	-0.031	6.66E-10	2982	NCKIPSD	Bradyrhizobium elkanii	-0.502	1.99E-07	4366	SMARCA2	Pseudomonas oleovorans	0.427	2.43E-03
215	ANKMY1	Rothia mucilaginosa	0.211	9.35E-05	1599	FAM185A	Gordonia bronchialis	-0.578	1.81E-04	2983	NCLN	Escherichia coli	-0.399	2.56E-10	4367	SMARCC1	Paucibacter	0.371	3.81E-09
216	ANKRA2	Malassezia restricta	0.326	3.81E-03	1600	FAM186A	Kytococcaceae	0.062	1.21E-04	2984	NCOA3	Acinetobacter ursingii	0.816	1.20E-32	4368	SMARCD2	Nostocales	-0.408	6.50E-03
217	ANKRA2	Thermaceae	-0.626	8.41E-04	1601	FAM186A	Pseudomonas oleovorans	-0.553	4.05E-08	2985	NCOA4	Actinomyces naeslundii	0.616	1.16E-25	4369	SMC1B	Pseudomonas oleovorans	-0.178	1.00E-04
218	ANKRD10	Actinomyces naeslundii	-0.322	2.67E-08	1602	FAM193B	Actinomyces naeslundii	-0.414	5.57E-73	2986	NCOA7	Escherichia coli	0.387	5.01E-04	4370	SMC4	Dolosigranulum pigrum	0.393	8.94E-03
219	ANKRD12	Thermaceae	-0.771	2.82E-03	1603	FAM193B	Ustilaginaceae	-0.083	7.37E-03	2987	NCOR2	Cutibacterium modestum	0.637	2.20E-04	4371	SMC4	Thermaceae	-0.719	1.36E-03
220	ANKRD18A	Schlegelella aquatica	-0.487	4.00E-03	1604	FAM199X	Streptococcaceae	0.439	3.35E-04	2988	NCS1	Lactobacillales	-0.368	4.99E-03	4372	SMC6	Ustilaginaceae	0.422	9.79E-07
221	ANKRD18B	Kytococcaceae	0.142	7.37E-03	1605	FAM20A	Actinomyces naeslundii	-0.134	1.00E-04	2989	NCSTN	Actinomyces naeslundii	0.465	1.35E-22	4373	SMCO2	Dolosigranulum pigrum	0.311	1.39E-05
222	ANKRD20A1	Fusobacterium	0.092	1.44E-04	1606	FAM210B	Plantactinospora	0.317	7.00E-04	2990	NDC80	Pseudomonas oleovorans	-0.294	5.02E-03	4374	SMG5	Escherichia coli	-0.328	1.13E-04
223	ANKRD20A1	Plantactinospora	-0.479	9.87E-03	1607	FAM222B	Dolosigranulum pigrum	0.235	4.29E-06	2991	NDEL1	Bacillus velezensis	0.245	7.46E-03	4375	SMIM10	Dolosigranulum pigrum	0.255	4.81E-04
224	ANKRD23	Actinomyces naeslundii	-0.219	1.08E-17	1608	FAM227A	Kytococcaceae	0.185	9.41E-05	2992	NDFIP1	Escherichia coli	0.327	4.42E-05	4376	SMIM13	Bradyrhizobium elkanii	0.487	1.42E-06
225	ANKRD24	Actinomyces naeslundii	-0.329	1.53E-11	1609	FAM227A	Pseudomonas oleovorans	-0.232	4.38E-08	2993	NDFIP2	Escherichia coli	0.312	6.12E-03	4377	SMIM13	Paucibacter	0.359	5.46E-04
226	ANKRD26	Thermaceae	-0.739	4.01E-13	1610	FAM228B	Actinomyces naeslundii	-0.448	6.36E-10	2994	NDOR1	Escherichia coli	-0.225	1.19E-11	4378	SMIM14	Escherichia coli	0.358	8.26E-03
227	ANKRD30A	Pseudomonas oleovorans	-0.439	4.60E-10	1611	FAM229B	Cutibacterium modestum	-0.620	5.87E-10	2995	NDST1	Acinetobacter ursingii	0.653	2.21E-12	4379	SMIM15	Escherichia coli	0.302	5.34E-07
228	ANKRD30B	Pseudomonas oleovorans	-0.300	2.24E-07	1612	FAM234A	Deinococcota	0.474	8.19E-03	2996	NDST3	Paucibacter	-0.253	7.90E-06	4380	SMIM18	Pseudomonas oleovorans	-0.308	1.14E-08
229	ANKRD33B	Bacillus velezensis	0.388	3.27E-04	1613	FAM234A	Dolosigranulum pigrum	-0.397	9.76E-03	2997	NDUFA1	Paucibacter	0.485	6.29E-04	4381	SMIM18	Thermales	-0.634	5.68E-03
230	ANKRD36C	Thermaceae	-0.715	1.03E-08	1614	FAM234A	Escherichia coli	-0.193	2.18E-04	2998	NDUFA12	Bradyrhizobium elkanii	0.608	1.82E-08	4382	SMIM19	Gordonia bronchialis	-0.629	6.97E-06
231	ANKRD37	Actinomyces naeslundii	-0.296	2.27E-03	1615	FAM246C	Dolosigranulum pigrum	-0.538	1.89E-13	2999	NDUFA4L2	Oxalobacteraceae	0.346	7.94E-03	4383	SMIM22	Dolosigranulum pigrum	0.369	7.41E-04
232	ANKRD39	Enterobacterales	-0.442	1.08E-04	1616	FAM32A	Dolosigranulum pigrum	-0.276	2.06E-06	3000	NDUFA5	Cutibacterium modestum	-0.483	7.85E-09	4384	SMIM24	Aspergillaceae	0.479	7.73E-03
233	ANKRD45	Pseudomonas oleovorans	-0.304	1.67E-04	1617	FAM3B	Kytococcaceae	0.166	7.01E-10	3001	NDUFA5	Escherichia coli	0.358	8.19E-12	4385	SMIM27	Dolosigranulum pigrum	0.525	1.30E-21
234	ANKRD46	Escherichia coli	0.278	9.68E-08	1618	FAM3D	Pseudomonas oleovorans	-0.308	8.03E-07	3002	NDUFA9	Actinomyces naeslundii	0.514	4.49E-08	4386	SMIM30	Escherichia coli	0.169	1.32E-05
235	ANKRD49	Dolosigranulum pigrum	0.379	6.48E-04	1619	FAM47E	Actinomyces naeslundii	-0.281	2.28E-05	3003	NDUFAB1	Paucibacter	0.342	1.49E-03	4387	SMIM36	Pseudomonas oleovorans	0.019	2.12E-05
236	ANKRD52	Yersiniaceae	-0.538	9.92E-04	1620	FAM50A	Actinomyces naeslundii	-0.454	2.52E-05	3004	NDUFAF3	Paucibacter	-0.425	1.57E-03	4388	SMIM6	Micromonosporales	-0.661	4.56E-03
237	ANKRD55	Paucibacter	-0.359	7.50E-03	1621	FAM50A	Kytococcaceae	-0.094	2.30E-05	3005	NDUFAF6	Ustilaginaceae	0.301	2.73E-04	4389	SMNDC1	Dolosigranulum pigrum	0.426	7.96E-07
238	ANKRD62	Pseudomonas oleovorans	-0.471	4.67E-12	1622	FAM50B	Cutibacterium acnes	-0.388	6.95E-03	3006	NDUFAF7	Dolosigranulum pigrum	0.345	1.65E-04	4390	SMNDC1	Escherichia coli	0.224	9.81E-34
239	ANKRD65	Dolosigranulum pigrum	-0.391	1.35E-05	1623	FAM53B	Bradyrhizobium elkanii	-0.593	8.39E-11	3007	NDUFB1	Paucibacter	0.474	4.18E-03	4391	SMO	Paucibacter	-0.570	2.60E-03
240	ANKRD9	Escherichia coli	-0.427	4.47E-04	1624	FAM76A	Actinomyces naeslundii	-0.360	1.65E-07	3008	NDUFB5	Bradyrhizobium elkanii	0.532	6.08E-05	4392	SMPD3	Gordonia bronchialis	0.512	1.44E-03
241	ANKS1A	Bradyrhizobium elkanii	-0.628	2.84E-08	1625	FAM76B	Actinomyces naeslundii	-0.309	4.76E-13	3009	NDUFB7	Actinomyces	0.308	1.56E-03	4393	SMPD4	Escherichia coli	-0.314	5.10E-09
242	ANKS6	Escherichia coli	-0.240	7.49E-03	1626	FAM83G	Escherichia coli	-0.371	2.78E-04	3010	NDUFC1	Cutibacterium modestum	-0.717	2.61E-09	4394	SMPDL3A	Oscillatoriales	0.232	6.72E-03
243	ANKZF1	Ustilaginaceae	0.019	9.81E-13	1627	FAM89A	Escherichia coli	0.300	6.67E-15	3011	NDUFS1	Actinomyces naeslundii	0.549	2.38E-59	4395	SMTN	Bradyrhizobium elkanii	-0.782	3.14E-06
244	ANO10	Actinomyces naeslundii	0.424	3.00E-07	1628	FAM8A1	Escherichia coli	0.431	3.55E-13	3012	NDUFS2	Actinomyces naeslundii	0.432	1.78E-09	4396	SMURF2	Dolosigranulum pigrum	0.495	1.22E-04
245	ANO2	Kytococcaceae	-0.008	5.16E-07	1629	FAM90A1	Dolosigranulum pigrum	0.198	7.39E-04	3013	NDUFS7	Escherichia coli	-0.161	2.28E-72	4397	SMYD1	Kytococcaceae	0.382	6.32E-05
246	ANO2	Pseudomonas oleovorans	-0.527	3.70E-12	1630	FAM91A1	Escherichia coli	0.525	3.85E-18	3014	NDUFV1	Escherichia coli	-0.477	3.37E-10	4398	SNAI3	Kytococcaceae	0.062	4.59E-08
247	ANO4	Pseudomonas oleovorans	-0.376	4.26E-09	1631	FAM98C	Escherichia coli	-0.359	1.11E-06	3015	NECAP2	Ustilaginaceae	-0.283	2.85E-07	4399	SNAP47	Dolosigranulum pigrum	-0.342	6.78E-08
248	ANO6	Actinomyces naeslundii	0.516	1.72E-86	1632	FAM98C	Paucibacter	-0.369	2.43E-04	3016	NEIL1	Actinomyces naeslundii	-0.265	1.29E-18	4400	SNAP91	Pseudomonas oleovorans	-0.339	3.86E-73
249	ANO9	Actinomyces naeslundii	-0.361	9.17E-12	1633	FAM9B	Actinomyces naeslundii	-0.445	5.56E-17	3017	NEK11	Kytococcaceae	0.000	2.75E-03	4401	SNAPC2	Ceratobasidiaceae	-0.388	1.03E-04
250	ANTXRL	Kytococcaceae	0.191	5.26E-06	1634	FANCA	Escherichia coli	-0.500	2.16E-05	3018	NEK3	Actinomyces naeslundii	-0.276	2.58E-09	4402	SNAPC4	Escherichia coli	-0.184	4.70E-08
251	ANTXRL	Pseudomonas oleovorans	-0.251	3.53E-11	1635	FANCB	Pseudomonas oleovorans	-0.127	3.54E-04	3019	NEK4	Rhizobiaceae	-0.420	2.71E-05	4403	SNCAIP	Dolosigranulum pigrum	0.226	2.61E-06
252	ANXA6	Actinomyces naeslundii	0.523	3.63E-07	1636	FANCL	Actinomyces naeslundii	-0.165	3.79E-13	3020	NEK5	Pseudomonas oleovorans	-0.297	8.25E-07	4404	SND1	Actinomyces naeslundii	0.207	4.73E-16
253	AOPEP	Halomonadaceae	-0.362	1.23E-03	1637	FANK1	Kytococcaceae	0.090	3.53E-03	3021	NEK7	Escherichia coli	0.464	1.21E-21	4405	SNORC	Escherichia coli	-0.588	1.46E-03
254	AOPEP	Ustilaginaceae	0.116	3.29E-05	1638	FARP1	Yersiniaceae	-0.605	6.92E-04	3022	NELFB	Escherichia coli	-0.410	2.20E-31	4406	SNRNP48	Fusobacteriaceae	0.309	9.25E-03
255	AP1AR	Escherichia coli	0.465	9.05E-13	1639	FARP2	Yersiniaceae	-0.685	1.11E-09	3023	NETO1	Pseudomonas oleovorans	-0.358	4.09E-95	4407	SNRNP70	Ustilaginaceae	-0.125	6.50E-03
256	AP1M1	Alphaproteobacteria	0.517	1.37E-06	1640	FASTK	Escherichia coli	-0.401	1.63E-07	3024	NETO2	Pseudomonas oleovorans	-0.380	1.19E-08	4408	SNRPA	Enterobacterales	-0.511	3.35E-04
257	AP1M2	Kytococcaceae	0.314	2.04E-05	1641	FASTKD1	Ustilaginaceae	0.469	4.57E-11	3025	NEU4	Paucibacter	-0.409	8.40E-04	4409	SNRPA	Fusobacterium	-0.299	7.82E-03
258	AP1S2	Escherichia coli	0.636	9.17E-08	1642	FAT2	Kytococcaceae	0.266	3.27E-04	3026	NEUROD6	Enterococcaceae	0.367	2.07E-03	4410	SNRPC	Actinomyces naeslundii	0.249	1.24E-07
259	AP2A1	Deinococcota	0.545	2.63E-03	1643	FAT3	Kytococcaceae	0.123	1.42E-03	3027	NF2	Actinomyces naeslundii	0.280	4.48E-10	4411	SNRPE	Paucibacter	0.463	3.83E-09
260	AP2A2	Paucibacter	-0.357	1.44E-12	1644	FAT3	Pseudomonas oleovorans	-0.393	6.24E-07	3028	NFATC2IP	Actinomyces naeslundii	-0.354	1.80E-17	4412	SNRPG	Escherichia coli	0.216	1.48E-05
261	AP3B1	Actinomyces naeslundii	0.387	7.87E-14	1645	FAXC	Acinetobacter johnsonii	0.392	1.17E-03	3029	NFATC2IP	Ustilaginaceae	0.268	1.84E-03	4413	SNTG2	Kytococcaceae	0.188	8.41E-06
262	AP3B2	Nevskiales	0.515	2.35E-03	1646	FAXC	Pseudomonas oleovorans	-0.251	2.26E-15	3030	NFATC4	Actinomyces naeslundii	-0.459	6.91E-22	4414	SNTG2	Pseudomonas oleovorans	-0.346	9.07E-12
263	AP3B2	Spirochaetota	0.355	4.58E-08	1647	FBLIM1	Actinomyces naeslundii	-0.532	5.91E-05	3031	NFATC4	Kytococcaceae	0.044	3.06E-08	4415	SNX13	Escherichia coli	0.317	1.58E-04
264	AP3D1	Escherichia coli	-0.338	1.33E-11	1648	FBN3	Kytococcaceae	0.217	3.11E-07	3032	NFE2L3	Actinomyces naeslundii	-0.057	2.30E-03	4416	SNX16	Alphaproteobacteria	-0.457	5.85E-06
265	AP3D1	Fusobacterium	-0.256	1.08E-04	1649	FBXL12	Actinomyces naeslundii	-0.469	3.30E-16	3033	NFIL3	Actinomyces naeslundii	-0.352	4.12E-04	4417	SNX16	Escherichia coli	0.247	2.60E-04
266	AP3D1	Gordonia bronchialis	0.441	5.29E-04	1650	FBXL14	Actinomyces naeslundii	0.207	3.14E-16	3034	NFKBIA	Leptotrichiaceae	0.180	1.89E-04	4418	SNX21	Sordariomycetes	-0.583	4.22E-15
267	AP3M1	Ascomycota	0.615	9.23E-03	1651	FBXL16	Kytococcaceae	0.029	1.99E-04	3035	NFKBID	Dolosigranulum pigrum	0.293	7.47E-06	4419	SNX27	Sordariomycetes	0.576	2.04E-03

268	AP3S1	Escherichia coli	0.348	1.76E-07	1652	FBXL2	Pseudomonas oleovorans	-0.414	8.62E-06	3036	NFKBIL1	Actinomyces naeslundii	-0.387	8.10E-06	4420	SNX3	Sordariales	0.366	9.24E-03
269	AP4B1	Actinomyces naeslundii	-0.307	2.65E-11	1653	FBXL3	Alphaproteobacteria	-0.433	5.09E-07	3037	NFRKB	Staphylococcus epidermidis	-0.370	7.50E-03	4421	SNX31	Pseudomonas oleovorans	-0.410	4.01E-06
270	AP5B1	Chaetomiaceae	-0.450	2.98E-03	1654	FBXL3	Escherichia coli	0.360	4.88E-11	3038	NGEF	Escherichia coli	-0.283	5.45E-14	4422	SNX6	Actinomyces naeslundii	0.250	3.72E-12
271	AP5B1	Escherichia coli	-0.427	1.93E-05	1655	FBXL6	Escherichia coli	-0.380	2.92E-06	3039	NGFR	Ustilaginaceae	-0.339	4.04E-06	4423	SNX7	Staphylococcus hominis	-0.337	3.94E-04
272	AP5Z1	Escherichia coli	-0.264	6.06E-07	1656	FBXO11	Dolosigranulum pigrum	0.631	8.26E-03	3040	NHLRC2	Escherichia coli	0.648	2.49E-10	4424	SOBP	Dolosigranulum pigrum	0.450	2.31E-03
273	APBA3	Escherichia coli	-0.346	4.71E-19	1657	FBXO15	Dolosigranulum pigrum	0.226	1.37E-06	3041	NHP2	Ustilaginaceae	-0.139	8.44E-12	4425	SOCS4	Dolosigranulum pigrum	0.362	6.09E-03
274	APBB3	Actinomyces naeslundii	-0.181	1.05E-20	1658	FBXO22	Achromobacter	0.638	2.65E-03	3042	NICN1	Ustilaginaceae	0.233	3.84E-04	4426	SOCS5	Escherichia coli	0.253	1.98E-03
275	APC2	Escherichia coli	-0.416	3.72E-03	1659	FBXO22	Bradyrhizobium elkanii	0.645	3.19E-03	3043	NINJ1	Actinomyces naeslundii	0.299	4.91E-05	4427	SORCS1	Pseudomonas oleovorans	-0.373	1.80E-06
276	APLNR	Actinomyces naeslundii	0.091	9.47E-07	1660	FBXO27	Deinococci	-0.490	3.80E-11	3044	NINJ1	Paucibacter	-0.542	4.76E-08	4428	SORCS2	Escherichia coli	-0.315	3.11E-03
277	APLNR	Ustilaginaceae	-0.344	1.93E-04	1661	FBXO28	Escherichia coli	0.573	5.22E-13	3045	NIPA2	Ascomycota	0.574	7.07E-06	4429	SOX12	Dolosigranulum pigrum	-0.527	1.88E-05
278	APLP1	Streptococcaceae	-0.459	3.27E-07	1662	FBXO30	Escherichia coli	0.489	7.77E-03	3046	NIPAL1	Pseudomonas oleovorans	0.278	8.70E-04	4430	SOX15	Deinococcota	0.359	3.30E-03
279	APLP2	Actinomyces naeslundii	0.248	2.24E-06	1663	FBXO43	Pseudomonas oleovorans	-0.336	4.50E-04	3047	NIPSNAP1	Actinomyces naeslundii	0.369	4.98E-10	4431	SOX17	Dolosigranulum pigrum	0.345	1.31E-07
280	APLP2	Plantactinospora	0.559	5.98E-03	1664	FBXO44	Ustilaginaceae	0.035	3.07E-03	3048	NIPSNAP3B	Actinomyces naeslundii	-0.271	3.47E-05	4432	SOX9	Dolosigranulum pigrum	0.198	1.62E-04
281	APLP2	Ustilaginaceae	-0.160	2.24E-06	1665	FBXW10	Kytococcaceae	0.248	8.43E-06	3049	NKAIN3	Pseudomonas oleovorans	-0.258	3.07E-06	4433	SP1	Acinetobacter ursingii	0.769	3.97E-05
282	APOBEC2	Clavicipitaceae	-0.413	6.44E-04	1666	FBXW10B	Kytococcaceae	0.134	4.01E-06	3050	NKAP	Acinetobacter ursingii	-0.753	1.57E-16	4434	SP140	Pseudomonas oleovorans	-0.418	1.93E-06
283	APOBEC2	Deinococcaceae	0.563	8.74E-06	1667	FBXW11	Actinomyces naeslundii	0.520	2.16E-16	3051	NKIRAS2	Leotiomycetes	-0.495	7.76E-03	4435	SP140L	Ustilaginaceae	0.212	8.80E-03
284	APOBEC3A	Dolosigranulum pigrum	0.196	2.67E-04	1668	FBXW5	Deinococcota	0.485	4.93E-03	3052	NKPD1	Kytococcaceae	0.251	3.48E-07	4436	SPACA6	Actinomyces naeslundii	-0.409	9.89E-07
285	APOC4-APO	Actinomyces naeslundii	-0.177	8.79E-08	1669	FBXW5	Escherichia coli	-0.200	3.22E-06	3053	NKRF	Actinomyces naeslundii	-0.215	8.05E-07	4437	SPAG17	Pseudomonas oleovorans	-0.226	7.01E-12
286	APOC4-APO	Ustilaginaceae	0.171	9.45E-03	1670	FBXW5	Paucibacter	-0.451	7.62E-03	3054	NKTR	Actinomyces naeslundii	-0.168	2.05E-05	4438	SPAG4	Sordariomycetes	-0.448	8.84E-04
287	APOE	Dolosigranulum pigrum	-0.364	2.79E-03	1671	FBXW7	Thermaceae	-0.696	1.29E-04	3055	NLGN1	Pseudomonas oleovorans	-0.334	5.17E-10	4439	SPAG5	Escherichia coli	-0.594	1.36E-07
288	APOF	Ustilaginaceae	-0.226	5.78E-04	1672	FCGR2B	Cutibacterium acnes	0.335	6.18E-04	3056	NLGN2	Actinomyces naeslundii	-0.204	1.36E-11	4440	SPAG5	Verrucomicrobiota	0.433	4.74E-04
289	APOL2	Sclerotiniaceae	0.510	3.87E-06	1673	FCGR2B	Paucibacter	-0.418	8.16E-03	3057	NLGN4Y	Rhodoferax	0.430	1.53E-07	4441	SPARCL1	Methyloversatilis sp. RAC08	-0.406	8.16E-03
290	APOL6	Actinomyces naeslundii	0.383	1.68E-07	1674	FCGRT	Ustilaginaceae	-0.361	8.30E-04	3058	NLN	Actinomyces naeslundii	0.470	1.80E-05	4442	SPART	Dolosigranulum pigrum	0.327	2.18E-04
291	APOO	Alphaproteobacteria	-0.562	1.27E-07	1675	FCHO2	Escherichia coli	0.574	1.11E-10	3059	NLRP1	Actinomyces naeslundii	-0.425	2.23E-09	4443	SPAST	Escherichia coli	0.325	1.72E-04
292	APOO	Bacteria	-0.465	1.44E-03	1676	FCHSD1	Actinomyces naeslundii	-0.366	2.19E-08	3060	NLRP2	Dolosigranulum pigrum	0.302	5.93E-03	4444	SPATA13	Actinomyces naeslundii	-0.202	3.25E-07
293	APOO	Bradyrhizobium elkanii	0.476	4.01E-06	1677	FCN1	Kitasatosporales	-0.417	9.99E-04	3061	NMD3	Kytococcaceae	-0.210	6.87E-10	4445	SPATA2L	Escherichia coli	-0.325	6.05E-07
294	APPBP2	Escherichia coli	0.524	3.06E-09	1678	FCN2	Paucibacter	-0.435	4.61E-07	3062	NME3	Escherichia coli	-0.256	1.96E-04	4446	SPATA6L	Actinomyces naeslundii	-0.313	2.23E-21
295	APPL1	Escherichia coli	0.565	9.25E-08	1679	FCN3	Paucibacter	-0.450	1.28E-05	3063	NME7	Actinomyces naeslundii	0.455	5.40E-97	4447	SPDYE12	Escherichia coli	-0.279	9.37E-27
296	APRT	Dolosigranulum pigrum	-0.418	5.15E-03	1680	FCRL5	Pseudomonas oleovorans	-0.515	1.26E-11	3064	NME8	Kytococcaceae	0.082	4.47E-08	4448	SPDYE12	Oxalobacteraceae	0.251	2.32E-70
297	APTX	Thermaceae	-0.616	8.91E-03	1681	FDX2	Actinomycetales	0.330	2.45E-03	3065	NME9	Pseudomonas oleovorans	-0.228	7.34E-05	4449	SPDYE16	Actinomyces naeslundii	-0.235	1.40E-03
298	AQP7	Actinomyces naeslundii	-0.473	2.42E-04	1682	FDX2	Enterobacterales	-0.571	4.87E-03	3066	NMI	Bradyrhizobium elkanii	0.568	3.83E-04	4450	SPEF2	Ustilaginaceae	0.202	1.11E-03
299	ARAP1	Paucibacter	-0.324	5.94E-04	1683	FDXR	Escherichia coli	-0.446	3.84E-05	3067	NMNAT1	Bradyrhizobium elkanii	0.592	1.05E-07	4451	SPIDR	Actinomyces naeslundii	-0.598	4.42E-11
300	ARCN1	Actinomyces naeslundii	0.430	3.62E-10	1684	FECH	Actinomyces naeslundii	0.467	1.42E-21	3068	NMNAT2	Pseudomonas oleovorans	-0.425	1.46E-07	4452	SPIN1	Escherichia coli	0.373	7.09E-05
301	ARFGAP1	Escherichia coli	-0.363	1.04E-05	1685	FEM1B	Enterobacterales	0.520	3.02E-03	3069	NMT2	Veillonella	-0.554	1.31E-05	4453	SPIN2A	Pseudomonas oleovorans	-0.462	3.61E-13
302	ARFGEF1	Acinetobacter ursingii	0.684	1.46E-06	1686	FEM1B	Escherichia coli	0.443	3.85E-04	3070	NNT	Actinomyces naeslundii	0.421	3.42E-19	4454	SPINK5	Kytococcaceae	0.081	9.74E-04
303	ARFIP1	Actinomyces naeslundii	0.362	3.16E-08	1687	FEM1B	Staphylococcus epidermidis	0.410	9.18E-04	3071	NOB1	Ceratobasidiaceae	-0.244	3.58E-04	4455	SPINT1	Kytococcaceae	0.223	9.42E-03
304	ARFIP2	Actinomyces naeslundii	-0.350	1.53E-05	1688	FER1L5	Kytococcaceae	0.152	1.44E-04	3072	NOB1	Dolosigranulum pigrum	-0.550	6.98E-05	4456	SPNS1	Escherichia coli	-0.306	2.73E-56
305	ARFIP2	Plantactinospora	-0.452	1.56E-03	1689	FER1L5	Pseudomonas oleovorans	-0.300	4.88E-09	3073	NOC2L	Escherichia coli	-0.335	6.86E-06	4457	SPON2	Escherichia coli	-0.484	1.47E-05
306	ARFRP1	Comamonadaceae	0.091	6.18E-03	1690	FER1L6	Kytococcaceae	0.030	1.95E-06	3074	NOC4L	Escherichia coli	-0.319	7.39E-08	4458	SPOPL	Halomonadaceae	0.198	3.31E-07
307	ARFRP1	Escherichia coli	-0.556	1.04E-11	1691	FERMT2	Escherichia coli	0.477	3.15E-03	3075	NOD1	Actinomyces naeslundii	-0.399	5.94E-07	4459	SPOUT1	Escherichia coli	-0.321	3.61E-05
308	ARGLU1	Actinomyces naeslundii	-0.330	6.25E-12	1692	FEZ1	Oscillatoriales	0.287	3.62E-03	3076	NOL3	Actinomyces naeslundii	-0.056	1.52E-26	4460	SPPL2B	Bradyrhizobium elkanii	-0.606	4.79E-06
309	ARHGAP22	Pseudomonas oleovorans	-0.396	7.81E-07	1693	FEZ1	Paucibacter	-0.477	6.35E-03	3077	NOL4	Chromatiales	-0.392	5.02E-04	4461	SPPL2B	Escherichia coli	-0.141	4.89E-03
310	ARHGAP27	Bradyrhizobium elkanii	-0.708	7.48E-06	1694	FGA	Actinomyces naeslundii	0.203	4.68E-12	3078	NOL4	Ottowia	-0.332	1.23E-07	4462	SPRED1	Escherichia coli	0.630	5.60E-10
311	ARHGAP32	Staphylococcus epidermidis	-0.442	2.77E-04	1695	FGA	Halomonadaceae	0.300	1.97E-05	3079	NOL8	Ustilaginaceae	0.406	5.68E-07	4463	SPRING1	Actinomyces naeslundii	0.256	2.48E-06
312	ARHGAP33	Ustilaginaceae	-0.173	9.27E-03	1696	FGA	Oxalobacteraceae	-0.427	3.05E-10	3080	NOMO1	Actinomyces naeslundii	0.407	2.99E-10	4464	SPRYD4	Bradyrhizobium elkanii	0.567	2.48E-07
313	ARHGAP35	Ceratobasidiaceae	-0.323	2.55E-04	1697	FGB	Actinomyces naeslundii	0.150	1.73E-14	3081	NOMO2	Actinomyces naeslundii	0.389	9.55E-05	4465	SPSB1	Plantactinospora sp. BB1	-0.427	6.46E-03
314	ARHGAP35	Dolosigranulum pigrum	-0.377	3.32E-03	1698	FGD2	Achromobacter	-0.706	2.70E-07	3082	NOP10	Bradyrhizobium elkanii	0.557	5.15E-06	4466	SPSB3	Deinococcota	0.552	8.64E-03
315	ARHGAP35	Pseudomonas oleovorans	0.474	2.71E-03	1699	FGD4	Escherichia coli	0.464	9.25E-04	3083	NOP58	Actinomyces naeslundii	-0.221	5.63E-22	4467	SPSB4	Paucibacter	-0.156	6.30E-14
316	ARHGAP4	Bradyrhizobium elkanii	-0.678	1.89E-04	1700	FGD5	Paucibacter	-0.287	7.97E-05	3084	NOP58	Thermaceae	-0.722	8.44E-04	4468	SPTA1	Kytococcaceae	0.210	2.85E-03
317	ARHGAP45	Bradyrhizobium elkanii	-0.534	1.65E-03	1701	FGF10	Kytococcaceae	0.079	4.13E-04	3085	NOS1	Haemophilus	0.469	2.00E-04	4469	SPTA1	Pseudomonas oleovorans	-0.290	2.34E-09
318	ARHGDIA	Escherichia coli	-0.294	2.15E-06	1702	FGF13	Pseudomonas oleovorans	-0.457	6.92E-05	3086	NOS1	Pseudomonas oleovorans	-0.223	1.95E-23	4470	SPTAN1	Alphaproteobacteria	0.524	9.46E-06
319	ARHGDIB	Escherichia coli	0.593	2.10E-04	1703	FGF17	Kytococcaceae	0.083	1.27E-18	3087	NOS1AP	Escherichia coli	-0.331	3.73E-03	4471	SPTB	Pseudomonas oleovorans	-0.242	9.70E-04
320	ARHGDIB	Ustilaginaceae	-0.264	1.98E-03	1704	FGF2	Escherichia coli	0.266	1.01E-08	3088	NOS1AP	Methyloversatilis sp. RAC08	0.371	4.13E-03	4472	SPTBN1	Pseudomonas oleovorans	0.419	6.78E-05
321	ARHGEF15	Sphingomonadaceae	0.326	1.70E-18	1705	FGF2	Mycosphaerellaceae	0.460	3.86E-03	3089	NOSIP	Escherichia coli	-0.429	1.80E-08	4473	SPTLC2	Thermaceae	0.568	2.98E-04

322	ARHGEF17	Bradyrhizobium elkanii	-0.549	6.80E-03	1706	FGF2	Streptococcaceae	0.508	6.12E-04	3090	NOSTRIN	Paucibacter	-0.085	8.23E-03	4474	SPTSSB	Gordoniaceae	0.297	6.55E-04
323	ARHGEF18	Alphaproteobacteria	0.633	1.03E-05	1707	FGFR1	Methylobacterium sp. FF17	-0.623	1.53E-04	3091	NOTCH1	Cutibacterium modestum	0.636	9.79E-04	4475	SPTSSB	Lactobacillales	-0.435	4.91E-09
324	ARHGEF18	Yersiniaceae	-0.666	3.92E-03	1708	FGFRL1	Thermaceae	0.610	2.33E-03	3092	NOTCH2NLB	Sphingomonadaceae	-0.261	1.57E-03	4476	SPX	Actinomyces naeslundii	-0.218	1.90E-27
325	ARHGEF2	Bradyrhizobium elkanii	-0.691	1.41E-03	1709	FGG	Actinomyces naeslundii	0.297	1.20E-12	3093	NOTCH4	Paucibacter	-0.109	9.48E-03	4477	SPX	Dolosigranulum pigrum	0.436	9.59E-04
326	ARHGEF25	Actinomyces naeslundii	-0.531	9.14E-20	1710	FGL2	Escherichia coli	0.727	5.69E-10	3094	NOX4	Pseudomonas oleovorans	-0.420	8.56E-05	4478	SQOR	Actinomyces naeslundii	0.359	1.46E-19
327	ARHGEF26	Actinomyces naeslundii	-0.122	7.64E-05	1711	FGL2	Glomerellaceae	0.313	2.54E-03	3095	NPAS1	Kytococcaceae	0.278	1.29E-10	4479	SRC	Bradyrhizobium elkanii	-0.572	1.67E-04
328	ARHGEF38	Pseudomonas oleovorans	-0.231	1.08E-05	1712	FHIT	Dolosigranulum pigrum	0.390	6.08E-03	3096	NPC2	Actinomyces naeslundii	0.253	9.89E-05	4480	SRCAP	Bradyrhizobium elkanii	-0.649	1.41E-05
329	ARHGEF9	Ustilaginaceae	0.107	1.17E-03	1713	FIGNL2	Glomerellales	-0.370	4.25E-04	3097	NPC2	Ustilaginaceae	-0.183	7.60E-07	4481	SREK1	Actinomyces naeslundii	-0.199	7.03E-08
330	ARID3B	Cutibacterium modestum	0.553	2.63E-06	1714	FIS1	Dolosigranulum pigrum	-0.367	4.82E-03	3098	NPFF	Actinomyces naeslundii	-0.248	6.01E-05	4482	SREK1	Thermaceae	-0.719	5.85E-03
331	ARID4A	Thermales	-0.677	5.89E-04	1715	FIZ1	Actinomyces naeslundii	-0.630	1.38E-13	3099	NPFF	Ustilaginaceae	0.209	3.23E-06	4483	SREK1	Ustilaginaceae	0.263	3.57E-03
332	ARID4B	Thermaceae	-0.804	7.14E-11	1716	FKBP11	Alphaproteobacteria	-0.644	7.06E-04	3100	NPFFR1	Pseudomonas oleovorans	-0.409	5.89E-23	4484	SREK1IP1	Alphaproteobacteria	-0.550	5.47E-07
333	ARIH2	Actinomyces naeslundii	-0.182	1.91E-09	1717	FKBP1A	Escherichia coli	0.372	6.31E-03	3101	NPHP1	Kytococcaceae	0.234	5.12E-03	4485	SREK1IP1	Chromatiaceae	0.172	6.02E-05
334	ARIH2	Ustilaginaceae	0.246	8.74E-03	1718	FKBP8	Escherichia coli	-0.280	2.83E-05	3102	NPHP3	Actinomyces naeslundii	-0.333	3.98E-08	4486	SRL	Pseudomonas oleovorans	-0.359	1.08E-03
335	ARL13B	Thermaceae	-0.613	9.90E-04	1719	FLG2	Pseudomonas oleovorans	-0.312	1.20E-07	3103	NPIPA2	Kytococcaceae	0.243	6.89E-04	4487	SRMS	Escherichia coli	-0.333	1.45E-03
336	ARL14	Kytococcaceae	0.188	7.73E-08	1720	FLII	Alphaproteobacteria	0.550	5.71E-06	3104	NPIPB13	Actinomyces naeslundii	-0.180	5.60E-04	4488	SRP14	Cutibacterium modestum	-0.687	2.20E-17
337	ARL15	Escherichia coli	0.457	3.55E-04	1721	FLNC	Kytococcaceae	0.132	2.45E-03	3105	NPIPB7	Dolosigranulum pigrum	0.096	8.97E-03	4489	SRP14	Escherichia coli	0.122	1.09E-06
338	ARL16	Actinomyces naeslundii	-0.334	5.79E-21	1722	FLRT1	Kytococcaceae	0.249	9.23E-03	3106	NPLOC4	Escherichia coli	-0.283	7.06E-08	4490	SRP9	Cutibacterium modestum	-0.651	8.56E-05
339	ARL17A	Paucibacter	-0.279	2.43E-05	1723	FLT3LG	Actinomyces naeslundii	-0.488	1.34E-12	3107	NPLOC4	Yersiniaceae	-0.562	1.11E-03	4491	SRPRB	Bacillus velezensis	-0.460	5.36E-04
340	ARL17B	Brucella	0.357	9.61E-03	1724	FLT4	Achromobacter	-0.709	6.30E-05	3108	NPRL2	Paucibacter	-0.321	4.09E-04	4492	SRRD	Dolosigranulum pigrum	-0.487	9.68E-05
341	ARL2	Enterobacterales	-0.491	5.70E-06	1725	FLT4	Bradyrhizobium elkanii	-0.716	5.87E-03	3109	NPRL3	Bradyrhizobium elkanii	-0.558	1.94E-12	4493	SRRM3	Pseudomonas oleovorans	-0.219	8.00E-07
342	ARL2BP	Bradyrhizobium elkanii	0.570	1.16E-05	1726	FLT4	Paucibacter	-0.224	7.42E-07	3110	NPRL3	Escherichia coli	-0.184	5.65E-03	4494	SRRM4	Kytococcaceae	0.280	6.37E-46
343	ARL5A	Escherichia coli	0.417	2.89E-67	1727	FLVCR1	Thermaceae	-0.531	1.98E-03	3111	NPTX2	Dolosigranulum pigrum	0.282	7.35E-05	4495	SRSF11	Actinomyces naeslundii	-0.280	1.37E-20
344	ARL6IP4	Escherichia coli	-0.327	3.58E-06	1728	FMN2	Pseudomonas oleovorans	-0.387	1.98E-06	3112	NPY1R	Escherichia coli	0.596	3.69E-03	4496	SRSF2	Actinomyces naeslundii	-0.180	2.17E-08
345	ARL6IP4	Paucibacter	-0.453	6.87E-06	1729	FMNL1	Methylobacterium sp. FF17	-0.694	1.86E-04	3113	NQO2	Dolosigranulum pigrum	-0.384	7.66E-04	4497	SRSF2	Ustilaginaceae	0.184	4.89E-04
346	ARL8B	Escherichia coli	0.299	7.43E-06	1730	FMNL2	Achromobacter	-0.660	1.80E-06	3114	NR0B2	Leptotrichiaceae	0.094	2.87E-04	4498	SRSF4	Actinomyces naeslundii	-0.196	3.88E-37
347	ARMC10	Escherichia coli	0.381	1.79E-10	1731	FMO5	Actinomyces naeslundii	-0.218	3.16E-12	3115	NR1H2	Escherichia coli	-0.279	9.33E-09	4499	SRSF4	Staphylococcus epidermidis	-0.299	4.18E-09
348	ARMC12	Actinomyces naeslundii	-0.358	2.56E-16	1732	FMR1	Escherichia coli	0.482	1.05E-07	3116	NR1H2	Leotiomycetes	-0.521	4.14E-04	4500	SRSF4	Ustilaginaceae	-0.002	3.21E-04
349	ARMC12	Kytococcaceae	0.103	1.11E-06	1733	FNBP1	Achromobacter	-0.750	2.63E-06	3117	NR1I2	Actinomyces naeslundii	-0.030	3.25E-14	4501	SRSF5	Actinomyces naeslundii	-0.145	6.54E-19
350	ARMC9	Herbaspirillum huttiense	-0.565	6.24E-04	1734	FNBP4	Actinomyces naeslundii	-0.166	2.70E-06	3118	NR2C1	Actinomyces naeslundii	-0.103	2.32E-04	4502	SRSF6	Actinomyces naeslundii	-0.325	5.66E-10
351	ARMC9	Pseudomonas oleovorans	-0.500	2.47E-06	1735	FNBP4	Thermaceae	-0.572	3.10E-03	3119	NR2E3	Pseudomonas oleovorans	-0.358	1.40E-04	4503	SRSF7	Actinomyces naeslundii	-0.381	1.20E-07
352	ARMCX4	Actinomyces naeslundii	-0.304	2.53E-13	1736	FNBP4	Ustilaginaceae	0.210	7.52E-04	3120	NR2F1	Achromobacter	-0.660	7.10E-08	4504	SRSF9	Actinomyces naeslundii	0.156	7.25E-08
353	ARMH1	Actinomyces naeslundii	-0.209	1.85E-20	1737	FNDC3A	Escherichia coli	0.502	8.18E-06	3121	NR2F2	Sordariales	0.639	7.12E-03	4505	SRSF9	Ustilaginaceae	-0.228	1.13E-04
354	ARMT1	Bradyrhizobium elkanii	0.688	1.04E-10	1738	FNDC3B	Schlegelella aquatica	0.288	7.69E-10	3122	NR4A1	Actinomyces naeslundii	-0.399	1.37E-03	4506	SSBP1	Bradyrhizobium elkanii	0.517	2.16E-07
355	ARNT	Pseudomonas oleovorans	0.588	3.77E-10	1739	FNIP1	Rhodobacterales	-0.365	7.73E-11	3123	NR5A2	Pseudomonas oleovorans	0.333	6.34E-03	4507	SSBP2	Pseudomonas oleovorans	-0.480	5.57E-06
356	ARPC1A	Ustilaginaceae	-0.365	6.07E-05	1740	FOLR2	Paucibacter	-0.379	5.35E-08	3124	NRAS	Escherichia coli	0.367	5.53E-04	4508	SSBP4	Escherichia coli	-0.354	2.30E-06
357	ARPC4-TTLL3	Paucibacter	-0.203	5.27E-06	1741	FOS	Leptotrichiaceae	0.181	1.37E-14	3125	NRBF2	Bradyrhizobium elkanii	0.689	1.01E-45	4509	SSH1	Bacillaceae	0.630	1.60E-03
358	ARPC5	Escherichia coli	0.159	2.58E-09	1742	FOS	Methylorubrum	0.597	4.24E-10	3126	NRBF2	Streptococcaceae	0.420	2.92E-21	4510	SSH1	Cytophagaceae	0.274	9.85E-03
359	ARPP19	Escherichia coli	0.556	2.42E-05	1743	FOSB	Actinomyces naeslundii	-0.463	5.84E-04	3127	NRBP2	Ustilaginaceae	-0.016	3.85E-03	4511	SSH2	Sphingobacterium	0.776	1.19E-03
360	ARPP19	Plantactinospora	0.565	1.46E-03	1744	FOSB	Leptotrichiaceae	0.226	1.48E-16	3128	NRF1	Actinomyces naeslundii	-0.232	1.37E-16	4512	SSH3	Deinococcota	0.510	6.55E-03
361	ARPP21	Pseudomonas oleovorans	-0.151	1.28E-03	1745	FOXN2	Acidovorax temperans	-0.415	7.30E-03	3129	NRP1	Escherichia coli	0.600	3.51E-04	4513	SSR3	Actinomyces naeslundii	0.181	3.52E-66
362	ARRDC2	Actinomyces naeslundii	-0.451	1.09E-17	1746	FOXN3	Pseudomonas oleovorans	0.296	9.62E-06	3130	NRP2	Paucibacter	-0.286	3.71E-16	4514	SSTR1	Kytococcaceae	-0.451	6.66E-06
363	ARSA	Escherichia coli	-0.423	4.97E-11	1747	FOXO4	Leotiomycetes	-0.696	1.45E-05	3131	NRROS	Leotiomycetes	-0.521	2.68E-07	4515	SSUH2	Dolosigranulum pigrum	0.259	1.16E-04
364	ART3	Pseudomonas oleovorans	-0.301	1.41E-04	1748	FOXP4	Bradyrhizobium elkanii	-0.605	6.60E-06	3132	NRTN	Escherichia coli	-0.517	1.11E-05	4516	ST13	Ustilaginaceae	-0.094	3.59E-15
365	ASAH2B	Brucellaceae	0.568	1.30E-12	1749	FRAT1	Dolosigranulum pigrum	-0.413	1.12E-03	3133	NRXN1	Pseudomonas oleovorans	-0.289	2.13E-04	4517	ST14	Escherichia coli	-0.449	5.48E-05
366	ASB14	Pseudomonas oleovorans	-0.214	5.34E-04	1750	FRAT2	Actinomyces naeslundii	0.381	5.40E-07	3134	NRXN3	Pseudomonas oleovorans	-0.342	1.88E-05	4518	ST18	Pseudomonas oleovorans	-0.234	1.39E-06
367	ASB4	Cellvibrionales	-0.404	1.08E-03	1751	FREM1	Kytococcaceae	0.163	2.73E-03	3135	NSMCE4A	Actinomyces naeslundii	-0.313	6.33E-03	4519	ST3GAL1	Pseudomonas oleovorans	0.373	1.08E-05
368	ASB6	Corynebacterium tuberculostear	0.404	2.44E-03	1752	FRK	Acinetobacter ursingii	0.654	3.94E-06	3136	NSMF	Escherichia coli	-0.316	6.69E-03	4520	ST3GAL3	Bradyrhizobium elkanii	-0.403	4.11E-04
369	ASCC2	Escherichia coli	-0.438	2.08E-87	1753	FRMD4A	Yersiniaceae	-0.757	2.38E-04	3137	NSRP1	Thermaceae	-0.707	2.02E-05	4521	ST3GAL3	Cutibacterium acnes	-0.449	2.07E-06
370	ASCC3	Actinomyces naeslundii	0.505	5.40E-05	1754	FRMD5	Pseudomonas oleovorans	-0.165	9.40E-11	3138	NSUN5	Escherichia coli	-0.365	1.16E-06	4522	ST6GAL1	Actinomyces naeslundii	0.362	1.99E-77
371	ASGR1	Escherichia coli	-0.333	1.44E-05	1755	FRMPD2	Kytococcaceae	0.220	5.01E-04	3139	NSUN6	Actinomyces naeslundii	-0.127	7.40E-05	4523	ST8SIA1	Pseudomonas oleovorans	-0.479	4.99E-07
372	ASIP	Kytococcaceae	0.122	1.20E-03	1756	FRMPD2	Pseudomonas oleovorans	-0.329	1.40E-10	3140	NSUN7	Dolosigranulum pigrum	0.173	6.19E-03	4524	ST8SIA2	Kytococcaceae	0.251	4.04E-05
373	ASL	Escherichia coli	-0.379	9.77E-06	1757	FRMPD4	Pseudomonas oleovorans	-0.317	1.02E-14	3141	NT5C	Escherichia coli	-0.177	2.12E-03	4525	ST8SIA2	Pseudomonas oleovorans	-0.298	1.46E-10
374	ASPA	Actinomyces naeslundii	-0.409	3.21E-09	1758	FRRS1L	Pseudomonas oleovorans	-0.257	3.29E-06	3142	NT5C1A	Pseudomonas oleovorans	-0.471	5.11E-09	4526	ST8SIA5	Pseudomonas oleovorans	-0.296	3.33E-06
375	ASPH	Actinomyces naeslundii	0.280	1.12E-41	1759	FSD1	Kytococcaceae	-0.028	2.35E-12	3143	NT5C3A	Acidovorax temperans	-0.133	2.05E-03	4527	STAB1	Bradyrhizobium elkanii	-0.671	1.29E-07

376	ASPN	Escherichia coli	0.494	8.29E-07	1760	FSTL4	Pseudomonas oleovorans	-0.224	4.20E-33	3144	NTM	Pseudomonas oleovorans	-0.423	8.07E-07	4528	STAB1	Paucibacter	-0.215	2.17E-04
377	ASPRV1	Ustilaginaceae	0.046	3.20E-05	1761	FTCD	Escherichia coli	-0.173	6.88E-06	3145	NTN1	Deinococcota	0.459	2.32E-03	4529	STAB2	Acinetobacter ursingii	0.671	1.17E-14
378	ASPSCR1	Escherichia coli	-0.351	3.97E-38	1762	FTO	Escherichia coli	-0.223	1.21E-05	3146	NTN3	Dolosigranulum pigrum	-0.400	2.64E-03	4530	STAB2	Paucibacter	-0.347	1.07E-06
379	ASS1	Streptococcaceae	-0.440	1.27E-04	1763	FUNDC1	Bradyrhizobium elkanii	0.566	1.16E-03	3147	NTN4	Escherichia coli	0.515	2.72E-04	4531	STAC	Pseudomonas oleovorans	-0.371	6.45E-03
380	ASTN2	Escherichia coli	-0.507	4.98E-03	1764	FURIN	Hyphomicrobiales	0.501	5.50E-05	3148	NTN4	Paucibacter	-0.288	2.48E-04	4532	STAC3	Ustilaginaceae	0.097	1.96E-05
381	ASXL1	Actinomyces naeslundii	-0.280	1.07E-25	1765	FUS	Actinomyces naeslundii	-0.334	1.50E-09	3149	NTNG1	Paucibacter	0.481	7.34E-03	4533	STAG2	Escherichia coli	0.447	4.45E-28
382	ASXL2	Actinomyces naeslundii	0.530	3.20E-11	1766	FUT3	Dolosigranulum pigrum	0.315	1.14E-14	3150	NTNG1	Pseudomonas oleovorans	-0.425	4.66E-09	4534	STAM	Actinomyces naeslundii	0.370	1.76E-05
383	ASXL3	Pseudomonas oleovorans	-0.088	1.66E-03	1767	FUT9	Pseudomonas oleovorans	-0.302	3.10E-18	3151	NTRK3	Kytococcaceae	0.100	1.39E-03	4535	STAM2	Escherichia coli	0.398	2.26E-04
384	ATAD1	Escherichia coli	0.252	6.30E-06	1768	FXYD1	Sordariomycetes	-0.425	3.74E-13	3152	NTRK3	Pseudomonas oleovorans	-0.389	1.21E-04	4536	STARD3	Bradyrhizobium elkanii	-0.430	1.13E-04
385	ATAD3A	Escherichia coli	-0.520	1.33E-12	1769	FXYD5	Cutibacterium acnes	0.421	6.77E-03	3153	NTSR1	Escherichia coli	-0.416	1.03E-35	4537	STARD3	Escherichia coli	-0.317	9.22E-27
386	ATAT1	Ustilaginaceae	-0.002	2.28E-04	1770	FYB2	Thermaceae	-0.670	3.23E-03	3154	NTSR1	Leptotrichiaceae	-0.215	1.16E-07	4538	STARD8	Alphaproteobacteria	0.601	6.80E-06
387	ATF1	Actinomyces naeslundii	0.185	8.03E-04	1771	FZD5	Actinomyces naeslundii	0.247	6.47E-08	3155	NUBP1	Actinomyces naeslundii	0.306	3.49E-10	4539	STARD9	Staphylococcus hominis	0.369	3.41E-03
388	ATF2	Escherichia coli	0.445	3.21E-12	1772	FZR1	Escherichia coli	-0.286	1.16E-34	3156	NUBP2	Escherichia coli	-0.370	5.06E-50	4540	STC1	Kytococcaceae	0.106	7.18E-05
389	ATF3	Methylorubrum	0.255	8.77E-03	1773	G2E3	Escherichia coli	0.423	7.44E-03	3157	NUDCD2	Cutibacterium modestum	-0.653	1.71E-04	4541	STEAP1B	Pseudomonas oleovorans	-0.327	8.94E-06
390	ATF4	Actinomyces naeslundii	-0.360	9.36E-12	1774	G3BP2	Actinomyces naeslundii	0.397	3.22E-10	3158	NUDCD2	Escherichia coli	0.122	3.81E-09	4542	STIM1	Streptococcaceae	-0.340	1.08E-03
391	ATG10	Acinetobacter ursingii	-0.584	3.57E-06	1775	GAA	Deinococcota	0.504	2.97E-04	3159	NUDT13	Actinomyces naeslundii	-0.261	5.67E-03	4543	STIM2	Dolosigranulum pigrum	0.458	1.45E-13
392	ATG16L1	Halomonadaceae	-0.145	1.61E-04	1776	GAB2	Bacillus velezensis	0.503	8.15E-04	3160	NUDT15	Gardnerella	-0.337	9.29E-05	4544	STK11	Escherichia coli	-0.455	4.02E-15
393	ATG16L2	Actinomyces naeslundii	-0.280	7.07E-18	1777	GAB2	Leotiomycetes	-0.414	1.56E-05	3161	NUDT22	Deinococcota	0.407	4.68E-05	4545	STK25	Escherichia coli	-0.251	2.02E-10
394	ATG2B	Dolosigranulum pigrum	0.403	4.69E-03	1778	GAB2	Paucibacter	-0.280	9.17E-05	3162	NUDT22	Escherichia coli	-0.417	1.63E-35	4546	STK26	Comamonadaceae	-0.343	5.14E-03
395	ATG4A	Methylobacterium sp. FF17	0.611	8.97E-10	1779	GABARAP	Halomonadaceae	0.002	1.37E-03	3163	NUDT4	Escherichia coli	0.380	1.53E-03	4547	STK26	Escherichia coli	0.411	1.15E-11
396	ATG9A	Escherichia coli	-0.275	6.34E-05	1780	GABARAP	Ustilaginaceae	-0.308	3.22E-09	3164	NUDT6	Dolosigranulum pigrum	-0.513	7.02E-11	4548	STK32A	Pseudomonas oleovorans	-0.327	9.18E-04
397	ATIC	Paucibacter	0.411	8.11E-03	1781	GABBR1	Actinomyces naeslundii	-0.410	9.14E-12	3165	NUMBL	Ustilaginaceae	-0.035	4.65E-14	4549	STK32B	Pseudomonas oleovorans	-0.338	3.32E-04
398	ATN1	Bradyrhizobium elkanii	-0.655	1.35E-07	1782	GABPA	Escherichia coli	0.542	1.99E-07	3166	NUP107	Actinomyces naeslundii	-0.136	2.18E-04	4550	STK32C	Pseudomonas oleovorans	-0.539	7.15E-13
399	ATP10D	Schlegelella aquatica	0.451	8.22E-03	1783	GABPB1	Dolosigranulum pigrum	0.653	2.48E-04	3167	NUP107	Ustilaginaceae	0.166	7.92E-03	4551	STK36	Ustilaginaceae	-0.056	9.37E-05
400	ATP11A	Acinetobacter ursingii	0.722	5.17E-09	1784	GABPB2	Actinomyces naeslundii	-0.283	1.48E-05	3168	NUP205	Cutibacterium modestum	0.710	2.35E-10	4552	STK38	Ustilaginaceae	0.286	8.10E-05
401	ATP13A2	Cutibacterium modestum	0.624	9.91E-11	1785	GABRA2	Pseudomonas oleovorans	-0.498	3.87E-27	3169	NUP210L	Kytococcaceae	0.041	1.50E-09	4553	STK39	Pseudomonas oleovorans	-0.350	3.63E-09
402	ATP13A2	Paucibacter	-0.376	3.75E-03	1786	GABRA4	Pseudomonas oleovorans	-0.242	8.75E-18	3170	NUP35	Bradyrhizobium elkanii	0.575	3.55E-03	4554	STK40	Ceratobasidiaceae	-0.294	2.23E-03
403	ATP13A5	Spirochaetota	0.241	9.16E-04	1787	GABRB2	Pseudomonas oleovorans	-0.362	1.03E-06	3171	NUP42	Dolosigranulum pigrum	0.423	4.35E-04	4555	STOML1	Escherichia coli	-0.565	1.57E-08
404	ATP2A2	Leptotrichiaceae	-0.096	9.79E-03	1788	GABRQ	Kytococcaceae	0.252	2.70E-14	3172	NUP43	Actinomyces naeslundii	-0.171	5.43E-16	4556	STRADA	Actinomyces naeslundii	-0.233	7.69E-04
405	ATP5F1A	Actinomyces naeslundii	0.476	1.78E-22	1789	GADD45B	Methylorubrum	0.525	2.40E-04	3173	NUP85	Ustilaginaceae	0.009	8.61E-06	4557	STRADA	Staphylococcus epidermidis	-0.435	3.66E-04
406	ATP5F1B	Actinomyces naeslundii	0.483	9.08E-108	1790	GADD45G	Leptotrichiaceae	0.004	1.63E-03	3174	NUS1	Escherichia coli	0.202	1.60E-06	4558	STRC	Kytococcaceae	0.214	5.02E-03
407	ATP5F1C	Bradyrhizobium elkanii	0.700	2.00E-06	1791	GAK	Bradyrhizobium elkanii	-0.628	2.65E-38	3175	NUSAP1	Dolosigranulum pigrum	0.424	4.62E-04	4559	STS	Actinomyces naeslundii	0.357	1.62E-24
408	ATP5MC3	Bradyrhizobium elkanii	0.492	5.35E-03	1792	GAK	Escherichia coli	-0.110	4.59E-95	3176	NWD1	Pseudomonas oleovorans	-0.178	1.31E-12	4560	STT3A	Actinomyces naeslundii	0.528	1.89E-23
409	ATP5MC3	Ustilaginaceae	-0.039	6.40E-06	1793	GAL3ST2	Ustilaginaceae	0.162	1.27E-03	3177	NXPE2	Pseudomonas oleovorans	-0.225	8.94E-07	4561	STT3B	Escherichia coli	0.280	8.09E-05
410	ATP5MF	Malassezia restricta	0.496	1.24E-09	1794	GALE	Paucibacter	-0.356	1.23E-08	3178	NXT2	Escherichia coli	0.424	9.84E-13	4562	STUB1	Deinococcota	0.484	2.99E-03
411	ATP5MF	Methyloversatilis sp. RAC08	-0.496	5.08E-12	1795	GALK2	Actinomyces naeslundii	0.437	1.33E-08	3179	NXT2	Plantactinospora	0.399	6.98E-25	4563	STUB1	Dolosigranulum pigrum	-0.565	4.04E-07
412	ATP5MF	Micromonosporales	-0.518	7.45E-03	1796	GALNT12	Paucibacter	0.585	1.77E-29	3180	NXT2	Ustilaginaceae	-0.119	2.12E-68	4564	STX10	Ustilaginaceae	-0.194	5.05E-06
413	ATP5MJ	Cutibacterium modestum	-0.764	5.47E-09	1797	GALNT12	Pseudomonas oleovorans	-0.371	1.15E-28	3181	NYAP2	Pseudomonas oleovorans	-0.172	3.96E-37	4565	STX16	Actinomyces naeslundii	-0.352	2.12E-13
414	ATP5PB	Bradyrhizobium elkanii	0.746	4.98E-09	1798	GALNT13	Pseudomonas oleovorans	-0.469	4.74E-10	3182	OAZ1	Ustilaginaceae	-0.401	1.09E-05	4566	STX16	Thermaceae	-0.645	9.65E-04
415	ATP5PF	Paucibacter	0.196	2.16E-04	1799	GALNT17	Pseudomonas oleovorans	-0.467	7.35E-13	3183	OCIAD1	Escherichia coli	0.309	8.18E-06	4567	STX17	Kytococcaceae	-0.002	1.51E-06
416	ATP6AP2	Escherichia coli	0.458	7.09E-11	1800	GALNT3	Actinomyces naeslundii	-0.234	3.49E-03	3184	OCIAD2	Bradyrhizobium elkanii	0.630	2.42E-04	4568	STXBP2	Escherichia coli	-0.179	3.20E-06
417	ATP6V0A1	Escherichia coli	-0.466	4.64E-17	1801	GALNT5	Pseudomonas oleovorans	-0.331	3.39E-08	3185	OCIAD2	Gordonia bronchialis	-0.717	4.23E-08	4569	STXBP4	Pseudomonas oleovorans	0.043	1.46E-03
418	ATP6V0A1	Kytococcaceae	0.239	8.01E-06	1802	GALNT8	Kytococcaceae	0.088	1.57E-05	3186	OCLN	Actinomyces naeslundii	0.462	5.72E-10	4570	STXBP5L	Kytococcaceae	0.198	2.80E-05
419	ATP6V0A1	Rhizobiaceae	0.304	5.35E-08	1803	GALNT9	Pseudomonas oleovorans	-0.215	5.00E-17	3187	OCRL	Ustilaginaceae	0.058	5.97E-06	4571	STXBP5L	Paucibacter	0.541	7.50E-07
420	ATP6V0C	Leotiomycetes	-0.502	4.45E-04	1804	GALT	Halomonadaceae	-0.309	3.99E-07	3188	ODAD1	Dolosigranulum pigrum	0.258	2.27E-11	4572	STXBP5L	Pseudomonas oleovorans	-0.373	2.18E-37
421	ATP6V0D1	Actinomyces naeslundii	0.365	2.02E-05	1805	GANC	Actinomyces naeslundii	-0.061	7.68E-03	3189	ODAD2	Pseudomonas oleovorans	-0.452	1.71E-10	4573	STYK1	Dolosigranulum pigrum	0.310	7.74E-04
422	ATP6V0E2	Actinomyces naeslundii	0.204	3.04E-03	1806	GAPDH	Actinomyces naeslundii	0.362	1.01E-09	3190	ODAD4	Pseudomonas oleovorans	-0.312	8.75E-03	4574	STYX	Escherichia coli	0.539	2.85E-06
423	ATP6V1A	Actinomyces naeslundii	0.368	6.95E-16	1807	GARNL3	Ustilaginaceae	0.183	1.84E-03	3191	ODF2L	Actinomyces naeslundii	-0.190	3.01E-04	4575	SUDS3	Actinomyces naeslundii	-0.241	4.55E-04
424	ATP7B	Acinetobacter ursingii	0.772	3.10E-16	1808	GAS6	Escherichia coli	-0.351	3.25E-10	3192	ODF3B	Escherichia coli	-0.276	3.35E-45	4576	SUDS3	Ustilaginaceae	0.114	1.72E-03
425	ATP8A2	Kytococcaceae	0.240	2.35E-16	1809	GATA3	Pseudomonadaceae	0.392	3.71E-03	3193	ODR4	Ustilaginaceae	0.450	3.50E-09	4577	SULT1C2	Staphylococcus hominis	0.349	2.82E-04
426	ATP8A2	Pseudomonas oleovorans	-0.278	3.57E-16	1810	GATA4	Paucibacter	-0.579	8.52E-04	3194	OFD1	Ustilaginaceae	0.343	3.61E-06	4578	SULT1C4	Dolosigranulum pigrum	0.414	8.29E-03
427	ATRN	Acinetobacter ursingii	0.773	4.92E-11	1811	GATAD1	Actinomyces naeslundii	-0.266	1.23E-06	3195	OGDH	Actinomyces naeslundii	0.421	3.60E-04	4579	SUMO1	Escherichia coli	0.191	1.54E-07
428	ATRN	Actinomyces naeslundii	0.442	4.45E-04	1812	GATAD1	Thermaceae	-0.731	6.29E-04	3196	OGDH	Dolosigranulum pigrum	-0.327	6.37E-03	4580	SUMO2	Escherichia coli	0.187	6.50E-19
429	ATXN1L	Actinomyces naeslundii	0.432	1.10E-03	1813	GATD1	Escherichia coli	-0.336	1.54E-04	3197	OGFOD2	Escherichia coli	-0.366	8.57E-08	4581	SUMO3	Actinomyces naeslundii	0.126	8.64E-26

430	ATXN1L	Dolosigranulum pigrum	-0.337	8.56E-03	1814	GATM	Escherichia coli	0.380	9.06E-04	3198	OGFOD3	Escherichia coli	-0.396	8.82E-05	4582	SUMO4	Actinomyces	-0.539	3.05E-03
431	AUP1	Actinomyces naeslundii	-0.279	1.25E-06	1815	GBP3	Basidiomycota	0.179	1.60E-03	3199	OGFR	Escherichia coli	-0.427	7.45E-28	4583	SUN2	Escherichia coli	-0.274	3.69E-04
432	AURKB	Pseudomonas oleovorans	-0.436	1.07E-05	1816	GBP6	Pseudomonas oleovorans	-0.270	7.00E-05	3200	OGFRL1	Escherichia coli	0.433	3.37E-03	4584	SUOX	Paucibacter	-0.550	1.88E-03
433	AURKC	Actinomyces naeslundii	-0.299	1.31E-04	1817	GC	Actinomyces naeslundii	0.595	6.09E-47	3201	OGN	Methyloversatilis sp. RAC08	-0.366	3.42E-03	4585	SUPT3H	Dolosigranulum pigrum	0.295	3.83E-04
434	AVIL	Actinomyces naeslundii	-0.207	3.60E-07	1818	GCC2	Thermaceae	-0.588	3.40E-05	3202	OIP5	Dolosigranulum pigrum	0.341	7.72E-04	4586	SUPT7L	Ustilaginaceae	0.251	5.88E-03
435	AXDND1	Kytococcaceae	0.234	3.25E-03	1819	GCGR	Escherichia coli	-0.219	2.30E-11	3203	OLFM2	Prevotella	0.500	2.07E-03	4587	SURF2	Kytococcaceae	0.020	3.80E-04
436	AXDND1	Pseudomonas oleovorans	-0.182	6.21E-06	1820	GCGR	Paucibacter	-0.312	1.97E-03	3204	OLFML2B	Dolosigranulum pigrum	0.315	1.44E-03	4588	SURF4	Actinomyces naeslundii	0.369	3.06E-199
437	AXL	Thermaceae	0.617	1.11E-05	1821	GCHFR	Leotiomycetes	-0.564	2.97E-03	3205	OPCML	Pseudomonas oleovorans	-0.411	4.98E-07	4589	SURF4	Ustilaginaceae	0.018	1.88E-24
438	B3GALT1	Kytococcaceae	0.067	3.22E-04	1822	GCSH	Ustilaginaceae	-0.062	1.69E-06	3206	OPLAH	Bradyrhizobium elkanii	-0.479	3.05E-03	4590	SURF6	Escherichia coli	-0.463	8.88E-03
439	B3GALT1	Pseudomonas oleovorans	-0.491	1.23E-12	1823	GDF2	Actinomyces naeslundii	0.200	1.02E-17	3207	OPLAH	Escherichia coli	-0.244	4.19E-37	4591	SUSD1	Thermales	0.696	6.46E-07
440	B3GALT2	Pseudomonas oleovorans	-0.385	2.49E-05	1824	GDI1	Actinomyces naeslundii	-0.285	5.18E-24	3208	OPN3	Actinomyces naeslundii	0.325	1.30E-06	4592	SUSD4	Fusobacteriota	0.515	1.33E-03
441	B3GAT1	Streptococcaceae	-0.486	3.92E-05	1825	GDI2	Actinomyces naeslundii	0.205	9.98E-21	3209	OPRD1	Pseudomonas oleovorans	-0.446	1.27E-09	4593	SUZ12	Alphaproteobacteria	-0.445	5.70E-04
442	B3GAT3	Escherichia coli	-0.466	7.77E-29	1826	GDPD4	Ustilaginaceae	0.104	8.90E-15	3210	OPRM1	Kytococcaceae	-0.024	2.35E-05	4594	SV2B	Bacilli	0.620	4.57E-08
443	B3GNT2	Escherichia coli	0.404	3.06E-10	1827	GET1	Escherichia coli	0.387	6.82E-10	3211	OPRM1	Pseudomonas oleovorans	-0.559	1.69E-18	4595	SV2C	Pseudomonas oleovorans	-0.211	4.82E-03
444	B3GNTL1	Kytococcaceae	0.137	1.16E-03	1828	GET3	Actinomyces naeslundii	0.298	2.07E-05	3212	OPTN	Kytococcaceae	-0.144	8.06E-03	4596	SVBP	Chromatiaceae	0.270	8.10E-03
445	B4GAT1	Dolosigranulum pigrum	-0.439	7.50E-05	1829	GFI1	Dolosigranulum pigrum	0.497	3.42E-20	3213	OR51E1	Leotiomycetes	0.478	4.00E-03	4597	SVIL	Staphylococcus hominis	0.382	3.86E-03
446	BACH1	Escherichia coli	0.489	4.62E-07	1830	GFM1	Actinomyces naeslundii	0.483	1.40E-25	3214	OR56A1	Pseudomonas oleovorans	-0.056	2.23E-04	4598	SVIP	Cutibacterium modestum	-0.633	1.03E-09
447	BAD	Dolosigranulum pigrum	-0.395	1.67E-03	1831	GFM2	Pseudomonas oleovorans	0.571	6.00E-08	3215	OR6A2	Pseudomonas oleovorans	-0.112	7.50E-17	4599	SWSAP1	Enterobacterales	-0.456	1.47E-03
448	BAG4	Bradyrhizobium elkanii	0.463	8.35E-05	1832	GFOD2	Escherichia coli	-0.412	2.29E-04	3216	ORAI1	Ustilaginaceae	-0.252	4.13E-04	4600	SWSAP1	Ustilaginaceae	0.219	1.89E-05
449	BAG4	Escherichia coli	0.249	5.54E-03	1833	GFPT2	Streptomyces sp. T12	0.286	5.08E-04	3217	ORAI3	Paucibacter	-0.440	8.32E-07	4601	SWT1	Ustilaginaceae	0.317	2.06E-04
450	BAHCC1	Bradyrhizobium elkanii	-0.611	1.53E-03	1834	GFRA1	Rhodoferax	-0.104	5.57E-03	3218	ORC1	Kytococcaceae	0.146	5.99E-04	4602	SYCP1	Pseudomonas oleovorans	-0.185	4.03E-05
451	BAHCC1	Xanthomonadales	0.357	6.50E-04	1835	GFRA2	Deinococci	0.512	7.73E-05	3219	ORM1	Actinomyces naeslundii	0.429	1.34E-24	4603	SYF2	Cutibacterium modestum	-0.627	1.64E-10
452	BAIAP2	Bradyrhizobium elkanii	-0.458	9.24E-04	1836	GFRA2	Paucibacter	-0.334	1.07E-05	3220	ORMDL1	Actinomyces naeslundii	-0.185	4.38E-05	4604	SYMPK	Escherichia coli	-0.271	2.86E-12
453	BAIAP2	Escherichia coli	-0.329	1.07E-23	1837	GFUS	Paucibacter	-0.507	8.73E-03	3221	ORMDL1	Thermaceae	-0.609	7.93E-03	4605	SYN2	Pseudomonas oleovorans	-0.429	1.04E-34
454	BAIAP2L2	Escherichia coli	-0.307	4.05E-03	1838	GGA1	Ustilaginaceae	-0.229	1.92E-27	3222	ORMDL1	Ustilaginaceae	0.190	4.61E-03	4606	SYNDIG1	Kytococcaceae	0.261	7.35E-06
455	BAK1	Kitasatosporales	-0.349	4.22E-03	1839	GGA3	Bradyrhizobium elkanii	-0.608	8.46E-04	3223	ORMDL3	Dolosigranulum pigrum	-0.366	7.81E-07	4607	SYNE2	Achromobacter	-0.621	9.15E-04
456	BATF3	Actinomyces naeslundii	-0.355	5.29E-04	1840	GGCT	Cutibacterium modestum	-0.705	1.20E-06	3224	OSBP	Actinomyces naeslundii	0.449	3.69E-09	4608	SYNE4	Halomonadaceae	-0.296	5.55E-06
457	BATF3	Dolosigranulum pigrum	0.295	9.56E-03	1841	GGCX	Actinomyces naeslundii	0.478	8.30E-10	3225	OSBPL8	Escherichia coli	0.575	1.35E-12	4609	SYNE4	Ustilaginaceae	0.010	2.65E-07
458	BAZ1B	Dolosigranulum pigrum	-0.320	3.48E-04	1842	GGH	Escherichia coli	0.221	6.10E-10	3226	OSCAR	Dolosigranulum pigrum	0.335	1.41E-04	4610	SYPL1	Bradyrhizobium elkanii	0.594	5.90E-11
459	BAZ1B	Methyloversatilis sp. RAC08	0.562	7.67E-03	1843	GGT1	Alphaproteobacteria	0.529	2.00E-05	3227	OSGEP	Actinomyces naeslundii	-0.431	3.51E-04	4611	SYT13	Pseudomonas oleovorans	-0.178	4.45E-05
460	BAZ2B	Thermaceae	-0.640	6.69E-18	1844	GGTA1	Escherichia coli	0.652	3.15E-06	3228	OSGIN1	Escherichia coli	-0.369	1.11E-06	4612	SYT14	Kytococcaceae	0.270	2.60E-10
461	BBC3	Escherichia coli	-0.614	4.22E-07	1845	GHR	Kytococcaceae	-0.020	1.36E-03	3229	OSGIN2	Escherichia coli	0.346	1.49E-04	4613	SYT14	Paucibacter	0.502	1.91E-07
462	BBS2	Caulobacterales	-0.443	7.42E-15	1846	GIGYF2	Cutibacterium modestum	0.713	1.47E-07	3230	OSMR	Ottowia	0.343	1.49E-03	4614	SYT14	Pseudomonas oleovorans	-0.299	6.09E-24
463	BBS9	Pseudomonas oleovorans	-0.164	6.36E-05	1847	GIMAP2	Escherichia coli	0.378	1.70E-04	3231	OSR1	Pseudomonas oleovorans	-0.257	6.35E-07	4615	SYT16	Kytococcaceae	0.314	2.10E-28
464	BCAM	Escherichia coli	-0.264	2.81E-06	1848	GIPC1	Escherichia coli	-0.431	4.42E-03	3232	OSTC	Paucibacter	0.276	1.34E-04	4616	SYT16	Pseudomonas oleovorans	-0.222	2.03E-51
465	BCAR1	Escherichia coli	-0.326	2.56E-08	1849	GJA1	Methyloversatilis sp. RAC08	-0.392	6.75E-06	3233	OSTF1	Actinomyces naeslundii	0.205	4.72E-10	4617	SYT6	Dolosigranulum pigrum	0.174	1.13E-03
466	BCAS2	Bradyrhizobium elkanii	0.683	2.37E-05	1850	GJB1	Actinomyces naeslundii	0.364	3.05E-04	3234	OSTF1	Ustilaginaceae	-0.154	4.73E-04	4618	SYT9	Cutibacterium granulosum	-0.294	1.79E-03
467	BCAS2	Escherichia coli	0.111	1.11E-04	1851	GJB1	Dolosigranulum pigrum	-0.402	1.39E-06	3235	OSTM1	Escherichia coli	0.309	8.57E-04	4619	SYTL4	Methyloversatilis sp. RAC08	0.459	1.42E-03
468	BCAS3	Actinomyces naeslundii	0.457	1.20E-18	1852	GJB2	Chromatiaceae	0.294	4.58E-04	3236	OTOG	Kytococcaceae	0.265	6.37E-09	4620	SZRD1	Ustilaginaceae	-0.135	2.32E-15
469	BCAS4	Pseudomonas oleovorans	-0.410	2.49E-10	1853	GJB5	Kytococcaceae	0.404	8.10E-05	3237	OTOG	Pseudomonas oleovorans	-0.369	1.34E-20	4621	TAB1	Deinococcota	0.575	7.93E-04
470	BCAT2	Escherichia coli	-0.268	5.58E-03	1854	GK	Escherichia coli	0.380	1.18E-04	3238	OTUB1	Escherichia coli	-0.300	2.30E-04	4622	TAB1	Dolosigranulum	-0.471	1.33E-03
471	BCDIN3D	Thermales	-0.589	4.14E-03	1855	GKAP1	Actinomyces naeslundii	-0.279	1.28E-11	3239	OTUD1	Escherichia coli	0.570	1.12E-03	4623	TACC3	Kytococcaceae	0.203	1.39E-05
472	BCHE	Escherichia coli	0.300	4.09E-05	1856	GLB1	Actinomyces naeslundii	0.523	2.12E-09	3240	OTUD5	Escherichia coli	-0.244	2.45E-03	4624	TACSTD2	Dolosigranulum pigrum	0.168	3.83E-14
473	BCKDHA	Actinomyces naeslundii	0.255	8.88E-10	1857	GLB1L2	Kytococcaceae	0.085	6.49E-08	3241	OTUD6B	Comamonadaceae	-0.374	2.77E-26	4625	TADA2A	Actinomyces naeslundii	-0.191	4.03E-09
474	BCKDHA	Paucibacter	-0.537	1.98E-20	1858	GLDN	Pseudomonas oleovorans	-0.431	2.66E-09	3242	OTUD7B	Dolosigranulum pigrum	-0.376	2.91E-04	4626	TADA3	Corynebacterium tuberculostea	0.372	2.21E-03
475	BCKDK	Hyphomicrobiales	0.474	2.03E-05	1859	GLG1	Acinetobacter ursingii	0.744	1.21E-13	3243	OVCA2	Escherichia coli	-0.278	7.23E-53	4627	TAF10	Fusobacterium	-0.187	3.97E-03
476	BCL10	Acidovorax temperans	-0.171	1.90E-03	1860	GLI2	Kytococcaceae	0.205	1.42E-06	3244	OVCH1	Pseudomonas oleovorans	-0.258	2.75E-07	4628	TAF13	Actinomyces naeslundii	0.413	6.07E-24
477	BCL10	Rhizobiaceae	-0.285	2.26E-10	1861	GLI3	Kytococcaceae	0.121	5.49E-03	3245	OXLD1	Escherichia coli	-0.294	4.26E-04	4629	TAF1C	Actinomyces naeslundii	-0.244	1.77E-09
478	BCL2L10	Ottowia	-0.431	1.48E-03	1862	GLIS1	Pseudomonas oleovorans	-0.279	2.75E-05	3246	OXSM	Corynebacterium accolens	0.643	2.04E-07	4630	TAF1D	Thermaceae	-0.665	6.02E-05
479	BCL2L12	Escherichia coli	-0.516	2.16E-03	1863	GLIS3	Kytococcaceae	0.259	7.26E-05	3247	OXTR	Moraxellaceae	-0.280	2.79E-06	4631	TAF6L	Escherichia coli	-0.316	4.14E-06
480	BCL2L14	Pseudomonas oleovorans	-0.373	2.70E-04	1864	GLRX5	Escherichia coli	0.310	5.10E-07	3248	P2RX4	Thermaceae	0.537	5.73E-05	4632	TAF9B	Comamonadaceae	-0.367	1.15E-12
481	BCL6	Actinomyces naeslundii	-0.339	4.21E-44	1865	GLS2	Acinetobacter ursingii	0.815	2.12E-16	3249	P2RX5	Actinomyces naeslundii	-0.397	7.31E-08	4633	TAF9B	Escherichia coli	0.398	7.92E-32
482	BCLAF3	Actinomyces naeslundii	-0.225	7.08E-18	1866	GLUD1	Kytococcaceae	-0.144	5.75E-08	3250	P2RY1	Escherichia coli	0.459	1.33E-24	4634	TAF9B	Staphylococcus saprophyticus	-0.203	4.81E-18
483	BCOR	Acinetobacter ursingii	0.594	1.16E-05	1867	GLUD2	Actinomyces naeslundii	0.374	1.24E-16	3251	P2RY12	Rothia mucilaginosa	-0.114	3.36E-03	4635	TAFAZZIN	Actinomyces naeslundii	-0.373	3.53E-16

484	BCR	Actinomyces	0.341	7.30E-07	1868	GLYATL1	Dolosigranulum pigrum	-0.241	1.51E-04	3252	P4HA1	Schlegelella aquatica	0.337	8.84E-03	4636	TAL1	Actinomyces naeslundii	-0.311	1.89E-03
485	BDH1	Escherichia coli	-0.424	3.67E-03	1869	GM2A	Actinomyces naeslundii	0.332	1.13E-13	3253	P4HB	Actinomyces naeslundii	0.388	1.43E-08	4637	TANGO2	Nocardioides	0.388	9.77E-07
486	BDH2	Actinomyces naeslundii	-0.301	8.97E-20	1870	GMCL1	Bradyrhizobium elkanii	0.317	8.86E-09	3254	PABIR2	Actinomyces naeslundii	-0.409	1.40E-09	4638	TANGO6	Actinomyces naeslundii	0.402	3.54E-07
487	BDKRB2	Pseudomonas oleovorans	-0.246	7.09E-05	1871	GMCL1	Escherichia coli	0.401	2.44E-07	3255	PABIR2	Dolosigranulum pigrum	0.434	1.73E-05	4639	TAOK2	Escherichia coli	-0.200	1.40E-07
488	BDNF	Pseudomonas oleovorans	-0.267	9.36E-05	1872	GMEB2	Escherichia coli	-0.349	6.55E-07	3256	PABIR3	Actinomyces naeslundii	-0.251	7.79E-04	4640	TAPBP	Acinetobacter ursingii	0.792	8.20E-15
489	BDNF	Streptomyces sp. T12	0.325	8.29E-05	1873	GMPPB	Leptotrichiaceae	-0.411	6.75E-03	3257	PABPC1L	Actinomyces naeslundii	-0.306	8.03E-12	4641	TAPBPL	Veillonella parvula	0.417	1.52E-03
490	BEAN1	Escherichia coli	-0.353	2.80E-10	1874	GNA12	Actinomyces naeslundii	0.153	2.72E-03	3258	PABPC4L	Streptomyces sp. T12	0.238	1.26E-07	4642	TARBP1	Ustilaginaceae	0.171	1.70E-05
491	BECN1	Actinomyces naeslundii	0.425	4.33E-07	1875	GNA13	Escherichia coli	0.627	1.14E-14	3259	PABPN1	Escherichia coli	-0.388	2.58E-05	4643	TARS2	Paucibacter	-0.457	8.80E-03
492	BEND6	Pseudomonas oleovorans	-0.327	2.15E-05	1876	GNAI2	Actinomyces naeslundii	0.390	4.50E-03	3260	PACRGL	Dolosigranulum pigrum	0.403	4.23E-03	4644	TAS2R20	Acidovorax temperans	-0.151	4.31E-05
493	BEST4	Kytococcaceae	0.277	4.41E-35	1877	GNAQ	Alphaproteobacteria	-0.439	1.33E-05	3261	PACS1	Ottowia	0.598	4.20E-03	4645	TAS2R20	Dolosigranulum pigrum	0.214	3.16E-03
494	BEX4	Escherichia coli	0.721	5.58E-06	1878	GNAZ	Kytococcaceae	0.018	6.26E-05	3262	PACS1	Thermales	0.607	5.26E-06	4646	TASOR	Escherichia coli	0.396	6.13E-04
495	BHLHE41	Dolosigranulum pigrum	0.388	9.99E-03	1879	GNAZ	Pseudomonas oleovorans	-0.485	3.57E-11	3263	PACS2	Bradyrhizobium elkanii	-0.633	1.75E-04	4647	TASOR	Rhizobiaceae	-0.470	7.69E-05
496	BID	Actinomyces naeslundii	0.269	7.37E-24	1880	GNB1	Actinomyces naeslundii	0.329	1.85E-07	3264	PAF1	Escherichia coli	-0.282	6.17E-03	4648	TASP1	Pseudomonas oleovorans	-0.273	1.78E-20
497	BIK	Debaryomycetaceae	-0.311	8.10E-04	1881	GNB1L	Kytococcaceae	0.197	5.25E-04	3265	PAGE4	Pseudomonas oleovorans	0.253	8.49E-42	4649	TATDN1	Paucibacter	0.291	6.77E-03
498	BIN1	Escherichia coli	-0.300	2.33E-04	1882	GNB3	Kytococcaceae	0.130	8.73E-04	3266	PAIP2	Paucibacter	0.342	1.04E-11	4650	TBC1D10B	Paucibacter	-0.355	8.10E-03
499	BIRC3	Streptococcaceae	0.406	4.73E-03	1883	GNB4	Escherichia coli	0.654	5.09E-07	3267	PAIP2B	Actinomyces naeslundii	0.452	6.71E-05	4651	TBC1D12	Dolosigranulum pigrum	0.436	3.05E-06
500	BIRC6	Acinetobacter ursingii	0.787	5.99E-16	1884	GNG11	Methyloversatilis sp. RAC08	-0.494	3.09E-04	3268	PAIP2B	Caldimonas	0.383	2.23E-03	4652	TBC1D14	Acinetobacter ursingii	0.732	1.19E-12
501	BIVM	Thermales	-0.709	2.09E-04	1885	GNG12	Actinomyces naeslundii	0.320	2.06E-28	3269	PAK2	Escherichia coli	0.491	2.46E-03	4653	TBC1D17	Bradyrhizobium elkanii	-0.587	9.37E-09
502	BLACAT1	Pseudomonas oleovorans	-0.139	4.05E-03	1886	GNG2	Escherichia coli	0.660	2.29E-06	3270	PALM2AKAP2	Ustilaginaceae	-0.110	5.37E-05	4654	TBC1D17	Escherichia coli	-0.200	7.89E-05
503	BLVRA	Escherichia coli	0.520	2.16E-05	1887	GNG7	Phyllobacteriaceae	-0.520	1.70E-04	3271	PALS1	Actinomyces naeslundii	0.523	9.76E-04	4655	TBC1D19	Pseudomonas oleovorans	-0.211	6.32E-05
504	BMI1	Acinetobacter ursingii	0.798	2.57E-14	1888	GNMT	Streptococcaceae	-0.368	7.73E-03	3272	PAM	Escherichia coli	0.512	2.27E-04	4656	TBC1D23	Escherichia coli	0.333	1.52E-07
505	BMP10	Actinomyces naeslundii	0.316	3.51E-04	1889	GNPDA2	Acidovorax temperans	-0.514	8.06E-04	3273	PAN2	Ustilaginaceae	0.017	5.16E-14	4657	TBC1D3E	Empedobacter	-0.242	1.69E-03
506	BMP10	Ustilaginaceae	-0.251	2.25E-03	1890	GNPDA2	Escherichia coli	0.422	3.46E-04	3274	PAN3	Actinomyces naeslundii	-0.308	6.11E-07	4658	TBC1D3E	Ustilaginaceae	0.121	1.26E-03
507	BMP2	Escherichia coli	0.503	4.30E-05	1891	GNRHR	Pseudomonas oleovorans	-0.184	1.27E-04	3275	PANK1	Campylobacterales	0.346	8.02E-03	4659	TBC1D8	Actinomyces naeslundii	-0.293	1.64E-53
508	BMP2K	Actinomyces naeslundii	0.582	6.27E-05	1892	GNS	Actinomyces naeslundii	0.414	4.18E-58	3276	PANK1	Escherichia coli	0.242	5.13E-03	4660	TBC1D9	Corynebacteriaceae	-0.399	2.30E-05
509	BMP3	Paucibacter	0.594	3.52E-04	1893	GOLGA4	Ustilaginaceae	0.305	7.64E-04	3277	PANK4	Escherichia coli	-0.239	6.49E-04	4661	TBC1D9	Escherichia coli	0.433	8.31E-04
510	BMP3	Streptomyces sp. T12	0.419	3.75E-05	1894	GOLGA5	Corynebacterium tuberculostea	-0.436	7.24E-03	3278	PANX1	Enterobacterales	0.527	1.16E-03	4662	TBC1D9B	Acinetobacter ursingii	0.690	1.24E-08
511	BMP8A	Actinomyces naeslundii	-0.198	3.73E-09	1895	GOLGA6L4	Chromatiales	-0.324	4.36E-03	3279	PAOX	Escherichia coli	-0.389	3.25E-04	4663	TBCB	Paucibacter	-0.206	9.28E-04
512	BMPR2	Escherichia coli	0.457	6.64E-06	1896	GOLGA6L4	Ustilaginaceae	0.212	2.42E-09	3280	PAPLN	Actinomyces naeslundii	-0.280	6.29E-07	4664	TBCCD1	Actinomyces naeslundii	0.461	3.03E-05
513	BMT2	Escherichia coli	0.328	9.11E-15	1897	GOLGA6L9	Actinomyces naeslundii	-0.274	5.21E-03	3281	PAPPA	Pseudomonas oleovorans	-0.245	4.02E-03	4665	TBCD	Escherichia coli	-0.257	1.61E-14
514	BNC2	Pseudomonas oleovorans	-0.344	1.50E-08	1898	GOLGA6L9	Caldimonas	-0.420	5.22E-04	3282	PAPSS1	Escherichia coli	0.532	1.39E-08	4666	TBCE	Dolosigranulum pigrum	-0.242	6.55E-05
515	BNIP2	Escherichia coli	0.272	5.93E-04	1899	GOLGA8A	Actinomyces naeslundii	-0.179	1.90E-07	3283	PAQR5	Deinococcaceae	0.473	8.38E-03	4667	TBCK	Ustilaginaceae	0.400	1.24E-04
516	BNIP3	Escherichia coli	0.252	1.05E-12	1900	GOLGA8A	Ustilaginaceae	0.120	4.69E-03	3284	PARD3	Kytococcaceae	0.368	3.55E-19	4668	TBL1X	Pseudomonas oleovorans	0.336	3.79E-06
517	BNIP3L	Escherichia coli	0.437	1.62E-18	1901	GOLGA8B	Actinomyces naeslundii	-0.243	3.14E-43	3285	PARD6G	Escherichia coli	0.535	5.77E-03	4669	TBL1XR1	Escherichia coli	0.715	1.01E-07
518	BOC	Pseudomonas oleovorans	-0.331	1.04E-03	1902	GOLGA8M	Actinomyces naeslundii	-0.283	2.86E-08	3286	PARG	Actinomyces naeslundii	0.378	7.65E-05	4670	TBL1Y	Rhodoferax	0.323	2.34E-05
519	BOD1L1	Actinomyces naeslundii	-0.086	1.44E-67	1903	GOLGA8N	Actinomyces naeslundii	-0.266	8.53E-21	3287	PARK7	Paucibacter	0.317	1.47E-04	4671	TBL3	Escherichia coli	-0.327	8.28E-13
520	BOD1L1	Thermaceae	-0.541	2.99E-06	1904	GOLGA8S	Dolosigranulum pigrum	0.301	8.17E-10	3288	PARM1	Dolosigranulum pigrum	0.332	9.88E-03	4672	TBRG1	Ustilaginaceae	0.340	5.53E-07
521	BOLA3	Bradyrhizobium elkanii	0.534	2.37E-03	1905	GOLIM4	Escherichia coli	0.429	1.81E-29	3289	PARP10	Escherichia coli	-0.385	1.07E-30	4673	TBRG4	Escherichia coli	-0.364	3.31E-06
522	BOP1	Escherichia coli	-0.335	1.43E-14	1906	GOLIM4	Rhizobiaceae	-0.391	1.73E-50	3290	PARP14	Acinetobacter ursingii	0.704	3.98E-09	4674	TBRG4	Paucibacter	-0.436	1.61E-05
523	BPGM	Bradyrhizobium elkanii	0.616	4.89E-05	1907	GOLM2	Escherichia coli	0.462	1.45E-08	3291	PARP15	Actinomyces naeslundii	-0.208	5.90E-05	4675	TBX1	Dolosigranulum pigrum	0.167	7.35E-08
524	BPNT2	Escherichia coli	0.375	9.29E-04	1908	GOLPH3L	Actinomyces naeslundii	0.332	9.85E-09	3292	PARP3	Halomonadaceae	-0.205	1.42E-05	4676	TBX19	Actinomyces naeslundii	-0.357	5.01E-08
525	BRAT1	Escherichia coli	-0.309	4.68E-05	1909	GORASP1	Escherichia coli	-0.404	1.17E-03	3293	PARP4	Acinetobacter ursingii	0.815	1.46E-18	4677	TCEA2	Escherichia coli	-0.287	2.63E-03
526	BRF1	Escherichia coli	-0.252	1.24E-06	1910	GORASP2	Actinomyces naeslundii	0.252	6.57E-09	3294	PARS2	Actinomyces naeslundii	0.378	1.32E-04	4678	TCEA3	Leptotrichiaceae	0.184	1.05E-03
527	BRI3	Ustilaginaceae	-0.399	1.33E-06	1911	GPAA1	Deinococcota	0.343	1.45E-03	3295	PAXBP1	Actinomyces naeslundii	-0.122	2.29E-06	4679	TCEAL4	Alphaproteobacteria	-0.564	3.74E-03
528	BRI3BP	Paucibacter	0.120	2.73E-07	1912	GPAA1	Escherichia coli	-0.446	3.45E-09	3296	PAXBP1	Ustilaginaceae	0.254	9.03E-03	4680	TCEAL4	Paucibacter	0.420	1.41E-03
529	BRK1	Gordonia bronchialis	-0.680	7.76E-06	1913	GPALPP1	Leotiomycetes	0.571	3.94E-04	3297	PAXIP1	Staphylococcus epidermidis	-0.390	7.41E-05	4681	TCEAL8	Escherichia coli	0.419	4.69E-16
530	BROX	Escherichia coli	0.344	1.53E-06	1914	GPATCH4	Escherichia coli	-0.678	2.13E-05	3298	PBDC1	Acinetobacter ursingii	-0.662	4.18E-09	4682	TCERG1	Actinomyces naeslundii	-0.183	1.15E-06
531	BROX	Rhizobiaceae	-0.384	7.49E-07	1915	GPBP1L1	Kytococcaceae	0.072	1.47E-05	3299	PBLD	Escherichia coli	0.509	7.86E-04	4683	TCF20	Acinetobacter ursingii	0.800	1.37E-30
532	BRPF3	Acinetobacter ursingii	0.804	2.57E-09	1916	GPC6	Bradyrhizobium elkanii	0.639	2.26E-07	3300	PBLD	Kytococcaceae	-0.334	2.09E-05	4684	TCF20	Bacillaceae	0.663	2.18E-09
533	BRSK2	Kytococcaceae	0.205	2.30E-10	1917	GPD1	Dolosigranulum pigrum	-0.251	2.52E-05	3301	PBX1	Spirochaetia	0.560	6.66E-06	4685	TCF20	Yersiniaceae	-0.723	2.15E-05
534	BSN	Escherichia coli	-0.335	4.07E-03	1918	GPD1	Paucibacter	-0.608	1.02E-03	3302	PBX4	Kytococcaceae	0.053	3.12E-03	4686	TCF25	Escherichia coli	-0.368	7.62E-09
535	BSND	Pseudomonas oleovorans	-0.455	5.45E-13	1919	GPD1L	Escherichia coli	0.514	1.16E-03	3303	PCBP1	Ustilaginaceae	-0.281	9.45E-05	4687	TCIM	Actinomyces naeslundii	-0.366	1.19E-07
536	BTBD16	Dolosigranulum pigrum	0.261	5.92E-06	1920	GPER1	Paucibacter	-0.485	1.32E-04	3304	PCBP3	Kytococcaceae	0.238	1.56E-04	4688	TCIM	Leptotrichiaceae	0.155	3.92E-05
537	BTBD2	Deinococcota	0.574	8.40E-04	1921	GPHA2	Dolosigranulum pigrum	0.253	1.97E-04	3305	PCBP4	Actinomyces naeslundii	-0.299	1.17E-29	4689	TCP10L	Kytococcaceae	-0.238	5.10E-06

538	BTBD3	Dolosigranulum pigrum	0.296	8.09E-21	1922	GPHA2	Staphylococcus epidermidis	-0.387	1.78E-06	3306	PCCA	Actinomyces naeslundii	0.450	1.73E-08	4690	TCTN3	Actinomyces naeslundii	0.420	2.06E-11
539	BTBD8	Nitrobacteraceae	-0.468	1.31E-03	1923	GPHA2	Ustilaginaceae	0.070	3.99E-06	3307	PCCB	Actinomyces naeslundii	0.423	1.55E-22	4691	TDP2	Escherichia coli	0.263	1.73E-04
540	BTD	Actinomyces naeslundii	0.470	2.12E-10	1924	GPI	Hyphomicrobiales	0.534	1.34E-03	3308	PCDH1	Actinomyces naeslundii	0.302	2.20E-04	4692	TDRD10	Actinomyces naeslundii	-0.366	9.19E-30
541	BTF3L4	Bradyrhizobium elkanii	0.487	3.67E-03	1925	GPM6A	Escherichia coli	0.657	3.14E-08	3309	PCDH12	Paucibacter	-0.481	6.07E-06	4693	TDRD12	Pseudomonas oleovorans	-0.383	2.09E-06
542	BTG3	Escherichia coli	0.401	6.09E-03	1926	GPR108	Paucibacter	-0.487	2.78E-03	3310	PCDH15	Kytococcaceae	0.155	2.94E-04	4694	TDRD3	Ustilaginaceae	0.164	3.95E-05
543	BTK	Escherichia coli	0.544	1.13E-03	1927	GPR135	Dolosigranulum pigrum	0.269	1.36E-04	3311	PCDHA10	Kytococcaceae	-0.038	8.64E-07	4695	TDRD6	Ustilaginaceae	0.296	5.53E-03
544	BTN2A1	Ustilaginaceae	0.035	1.80E-03	1928	GPR137	Escherichia coli	-0.374	4.87E-04	3312	PCDHA4	Pseudomonas oleovorans	-0.437	5.46E-09	4696	TDRP	Actinomyces naeslundii	-0.259	5.83E-37
545	BTNL9	Actinomyces naeslundii	-0.362	1.86E-13	1929	GPR137C	Acidovorax temperans	-0.315	7.53E-07	3313	PCDHB16	Ottowia	0.536	1.06E-04	4697	TDRP	Dolosigranulum pigrum	0.338	2.18E-04
546	C10orf105	Escherichia coli	-0.311	6.42E-04	1930	GPR137C	Kytococcaceae	0.155	9.54E-08	3314	PCDHB2	Dolosigranulum pigrum	0.328	3.86E-03	4698	TDRP	Ustilaginaceae	0.051	1.12E-10
547	C10orf67	Pseudomonas oleovorans	-0.193	6.50E-04	1931	GPR141	Kytococcaceae	0.140	7.92E-04	3315	PCDHGB1	Acidimicrobiales	0.334	8.25E-03	4699	TEAD3	Actinomyces naeslundii	-0.221	8.74E-03
548	C10orf90	Pseudomonas oleovorans	-0.225	8.06E-24	1932	GPR160	Escherichia coli	0.571	1.10E-07	3316	PCDHGB7	Thermales	0.644	3.23E-03	4700	TECR	Actinomyces naeslundii	0.313	3.62E-03
549	C11orf71	Kytococcaceae	-0.416	1.98E-03	1933	GPR173	Dolosigranulum pigrum	0.198	3.41E-04	3317	PCED1B	Dolosigranulum pigrum	0.380	2.18E-07	4701	TECR	Paucibacter	-0.577	2.55E-03
550	C11orf91	Dolosigranulum pigrum	0.218	9.55E-08	1934	GPR179	Kytococcaceae	0.154	3.78E-07	3318	PCGF3	Actinomyces naeslundii	-0.188	3.62E-36	4702	TECTB	Escherichia coli	-0.408	9.26E-03
551	C12orf29	Escherichia coli	0.344	1.28E-05	1935	GPR182	Paucibacter	-0.542	2.56E-08	3319	PCGF5	Escherichia coli	0.338	5.99E-09	4703	TEDC1	Escherichia coli	-0.456	1.11E-05
552	C12orf56	Pseudomonas oleovorans	-0.410	7.02E-05	1936	GPR26	Kytococcaceae	0.230	2.26E-07	3320	PCK2	Actinomyces	0.232	2.99E-03	4704	TEK	Paucibacter	-0.298	6.55E-22
553	C12orf60	Actinomyces naeslundii	-0.139	2.76E-08	1937	GPR34	Escherichia coli	0.585	5.55E-08	3321	PCK2	Dolosigranulum pigrum	-0.507	2.82E-11	4705	TEKT3	Kytococcaceae	0.081	6.43E-05
554	C12orf75	Dolosigranulum pigrum	0.274	1.70E-03	1938	GPR35	Actinomyces naeslundii	-0.289	5.97E-03	3322	PCMTD2	Actinomyces naeslundii	-0.433	1.76E-08	4706	TEKT5	Kytococcaceae	0.280	8.31E-04
555	C12orf76	Actinomyces naeslundii	-0.354	7.34E-05	1939	GPR35	Kytococcaceae	0.098	1.50E-03	3323	PCNP	Escherichia coli	0.369	2.85E-91	4707	TELO2	Escherichia coli	-0.273	7.81E-19
556	C13orf46	Kytococcaceae	0.242	1.45E-07	1940	GPR37	Actinomyces naeslundii	0.341	1.08E-06	3324	PCNT	Bradyrhizobium elkanii	-0.658	1.08E-04	4708	TENM1	Ustilaginaceae	0.283	4.21E-04
557	C13orf46	Pseudomonas oleovorans	-0.316	6.98E-10	1941	GPR39	Actinomyces naeslundii	0.304	2.24E-12	3325	PCNX1	Acinetobacter ursingii	0.724	1.24E-13	4709	TENM2	Haemophilus	-0.376	2.70E-13
558	C14orf28	Dolosigranulum pigrum	0.433	5.74E-04	1942	GPR63	Pseudomonas oleovorans	-0.263	1.41E-05	3326	PCSK1N	Mycobacteriaceae	0.396	7.54E-03	4710	TENM4	Pseudomonas oleovorans	-0.324	3.85E-14
559	C14orf39	Paucibacter	0.404	3.41E-03	1943	GPR65	Escherichia coli	0.605	7.77E-07	3327	PCSK5	Kytococcaceae	0.074	3.86E-04	4711	TENT4A	Actinomyces naeslundii	-0.365	3.47E-22
560	C14orf39	Pseudomonas oleovorans	-0.309	2.58E-13	1944	GPR68	Dolosigranulum pigrum	0.165	1.04E-07	3328	PCSK7	Yersiniaceae	-0.584	1.01E-03	4712	TENT5C	Lactobacillales	-0.335	1.56E-03
561	C14orf93	Ustilaginaceae	0.194	8.31E-04	1945	GPR89B	Actinomyces naeslundii	-0.428	1.80E-07	3329	PCSK9	Escherichia coli	-0.378	5.94E-04	4713	TERB1	Pseudomonas oleovorans	-0.246	7.61E-10
562	C16orf86	Escherichia coli	-0.253	3.47E-03	1946	GPRASP1	Actinomyces naeslundii	-0.432	3.01E-19	3330	PCYOX1	Actinomyces naeslundii	0.470	6.61E-09	4714	TERT	Pseudomonas oleovorans	0.030	6.40E-40
563	C16orf87	Escherichia coli	0.352	2.24E-05	1947	GPRC5C	Escherichia coli	-0.307	1.71E-03	3331	PCYOX1L	Dolosigranulum pigrum	0.507	2.30E-10	4715	TES	Dolosigranulum pigrum	0.330	7.51E-05
564	C16orf96	Kytococcaceae	0.247	1.33E-04	1948	GPRIN2	Kytococcaceae	0.206	5.53E-06	3332	PCYT1B	Pseudomonas oleovorans	-0.218	1.73E-05	4716	TET3	Actinomyces naeslundii	-0.260	8.93E-11
565	C17orf113	Dolosigranulum pigrum	0.364	6.12E-06	1949	GPRIN3	Dolosigranulum pigrum	0.412	5.44E-03	3333	PCYT2	Escherichia coli	-0.431	4.76E-04	4717	TEX12	Dolosigranulum pigrum	0.239	8.18E-03
566	C19orf18	Actinomyces naeslundii	-0.374	2.25E-08	1950	GPS1	Escherichia coli	-0.296	2.66E-09	3334	PDCD11	Escherichia coli	-0.326	3.23E-10	4718	TEX2	Pseudomonas oleovorans	0.524	1.55E-05
567	C19orf18	Dolosigranulum pigrum	0.227	2.33E-03	1951	GPS1	Paucibacter	-0.399	4.44E-04	3335	PDCD4	Corynebacterium tuberculostea	-0.262	4.50E-03	4719	TEX29	Actinomyces naeslundii	-0.324	1.56E-06
568	C19orf25	Escherichia coli	-0.595	8.61E-09	1952	GPS2	Paucibacter	-0.432	9.95E-06	3336	PDE10A	Pseudomonas oleovorans	-0.107	5.01E-05	4720	TEX30	Thermaceae	-0.709	4.60E-04
569	C1D	Ustilaginaceae	-0.208	1.07E-03	1953	GPT	Escherichia coli	-0.285	8.12E-15	3337	PDE1C	Kytococcaceae	0.155	5.19E-06	4721	TFCP2L1	Staphylococcus epidermidis	-0.422	6.46E-04
570	C1orf159	Escherichia coli	-0.301	3.92E-06	1954	GPX1	Leotiomycetes	-0.555	1.50E-04	3338	PDE1C	Pseudomonas oleovorans	-0.443	2.40E-11	4722	TFEB	Escherichia coli	-0.452	2.77E-04
571	C1orf159	Ustilaginaceae	0.055	2.44E-03	1955	GRAP	Paucibacter	-0.458	1.70E-10	3339	PDE2A	Paucibacter	-0.283	1.13E-08	4723	TFEC	Escherichia coli	0.513	1.08E-03
572	C1orf162	Dolosigranulum pigrum	0.427	5.35E-04	1956	GRB14	Actinomyces naeslundii	0.014	1.20E-07	3340	PDE4C	Kytococcaceae	0.014	1.22E-04	4724	TG	Pseudomonas oleovorans	-0.326	2.43E-11
573	C1orf43	Bradyrhizobium elkanii	0.624	3.17E-11	1957	GRB2	Ustilaginaceae	-0.267	1.10E-08	3341	PDE4D	Pseudomonas oleovorans	-0.329	9.44E-06	4725	TGFA	Streptococcaceae	0.444	1.86E-03
574	C1orf52	Actinomyces naeslundii	-0.389	1.15E-04	1958	GREP1	Escherichia coli	-0.358	1.17E-06	3342	PDE6A	Pseudomonas oleovorans	-0.283	3.84E-07	4726	TGFBR3	Alcaligenaceae	0.339	6.38E-04
575	C1orf74	Actinomyces naeslundii	0.413	1.80E-06	1959	GRHL1	Actinomyces naeslundii	-0.225	4.82E-05	3343	PDE6B	Dolosigranulum pigrum	0.311	6.72E-11	4727	TGFBR3	Escherichia coli	0.554	6.57E-04
576	C1QA	Paucibacter	-0.306	5.47E-09	1960	GRHL2	Kytococcaceae	0.190	1.26E-03	3344	PDE7A	Actinomyces naeslundii	-0.239	3.69E-03	4728	TGM2	Actinomyces naeslundii	0.346	7.84E-10
577	C1QB	Paucibacter	-0.362	1.13E-13	1961	GRHPR	Dolosigranulum pigrum	-0.318	1.39E-03	3345	PDE8A	Halomonadaceae	-0.072	2.48E-05	4729	TGOLN2	Actinomyces naeslundii	0.421	1.03E-03
578	C1QBP	Actinomyces naeslundii	0.240	2.94E-12	1962	GRIA1	Pseudomonas oleovorans	-0.271	3.68E-09	3346	PDE8B	Ottowia	0.451	3.48E-03	4730	THAP11	Ustilaginaceae	-0.295	1.06E-06
579	C1QBP	Ustilaginaceae	0.037	1.39E-03	1963	GRIA4	Kytococcaceae	0.156	1.46E-07	3347	PDE9A	Kytococcaceae	0.142	1.22E-58	4731	THAP7	Escherichia coli	-0.274	6.74E-11
580	C1QC	Actinomyces naeslundii	0.300	7.17E-22	1964	GRIA4	Paucibacter	0.525	4.04E-03	3348	PDF	Cloacibacterium normanense	0.521	3.30E-16	4732	THAP7	Paucibacter	-0.449	2.02E-04
581	C1QC	Paucibacter	-0.392	3.86E-07	1965	GRIA4	Pseudomonas oleovorans	-0.414	2.39E-21	3349	PDGFC	Escherichia coli	0.405	2.49E-04	4733	THBS3	Actinomyces naeslundii	-0.422	2.50E-27
582	C1QTNF3	Dolosigranulum pigrum	0.342	2.35E-03	1966	GRID2	Pseudomonas oleovorans	-0.394	2.55E-34	3350	PDHA1	Verrucomicrobiae	-0.382	7.33E-03	4734	THEM4	Actinomyces naeslundii	-0.243	1.72E-05
583	C1QTNF3	Hyphomicrobiales	-0.478	7.27E-03	1967	GRID2	Spirochaetota	0.316	7.35E-18	3351	PDIA4	Actinomyces naeslundii	0.356	4.08E-45	4735	THEM4	Ustilaginaceae	0.225	3.16E-04
584	C1QTNF3-AI	Malassezia vespertilionis	0.218	5.84E-04	1968	GRIK1	Pseudomonas oleovorans	-0.531	6.70E-10	3352	PDIA6	Actinomyces naeslundii	0.402	1.89E-30	4736	THEM5	Chromatiaceae	-0.240	1.90E-07
585	C1QTNF3-AI	Micromonosporales	0.356	1.53E-05	1969	GRIK2	Pseudomonas oleovorans	-0.222	2.10E-05	3353	PDK1	Paucibacter	0.523	6.02E-03	4737	THEM6	Dolosigranulum pigrum	-0.509	1.24E-05
586	C1S	Rhizobiaceae	0.443	7.18E-03	1970	GRIK2	Staphylococcus hominis	0.276	3.70E-05	3354	PDK2	Dolosigranulum pigrum	-0.321	3.19E-03	4738	THEM6	Paucibacter	-0.548	1.61E-03
587	C2	Actinomyces naeslundii	0.257	2.46E-07	1971	GRIK3	Pseudomonas oleovorans	-0.231	7.78E-05	3355	PDK2	Leotiomycetes	-0.588	2.77E-03	4739	THEMIS	Pseudomonas oleovorans	-0.274	2.53E-06
588	C20orf203	Kytococcaceae	0.117	5.66E-12	1972	GRIN2A	Kytococcaceae	0.112	5.08E-12	3356	PDK2	Paucibacter	-0.612	5.11E-04	4740	THEMIS2	Cutibacterium acnes	0.561	2.56E-03
589	C20orf203	Pseudomonas oleovorans	-0.502	1.84E-25	1973	GRIN2A	Pseudomonas oleovorans	-0.463	3.21E-13	3357	PDPN	Acinetobacter johnsonii	0.356	3.54E-03	4741	THNSL1	Kytococcaceae	-0.169	2.18E-03
590	C20orf96	Ustilaginaceae	-0.016	4.44E-11	1974	GRIN2B	Pseudomonas oleovorans	-0.381	1.02E-11	3358	PDPN	Streptomyces sp. T12	0.409	5.74E-09	4742	THOC1	Actinomyces naeslundii	-0.236	2.65E-03
591	C21orf62	Streptomyces sp. T12	0.410	3.13E-03	1975	GRIN3B	Escherichia coli	-0.604	5.40E-03	3359	PDRG1	Plantactinospora	-0.504	7.52E-03	4743	THOC1	Ustilaginaceae	0.053	4.00E-05

592	C2CD2L	Escherichia coli	-0.255	8.88E-03	1976	GRIP1	Dolosigranulum pigrum	0.299	3.13E-04	3360	PDSS2	Actinomyces naeslundii	0.474	7.25E-12	4744	THOC2	Actinomyces naeslundii	0.001	4.76E-24
593	C2CD6	Dolosigranulum pigrum	0.264	1.42E-03	1977	GRIPAP1	Actinomyces naeslundii	-0.137	1.70E-09	3361	PDXK	Escherichia coli	-0.452	1.14E-07	4745	THOC3	Ustilaginaceae	-0.149	3.95E-04
594	C2orf69	Escherichia coli	0.364	1.07E-07	1978	GRK2	Deinococcota	0.547	3.89E-04	3362	PDZD11	Micromonosporales	-0.494	6.84E-07	4746	THOC5	Ustilaginaceae	0.040	3.45E-15
595	C3	Actinomyces naeslundii	0.337	7.06E-10	1979	GRK4	Actinomyces naeslundii	-0.213	1.31E-18	3363	PDZD4	Actinomyces naeslundii	-0.404	6.51E-185	4747	THOC7	Bacillaceae	-0.533	1.63E-06
596	C3orf20	Micromonosporales	0.372	7.00E-03	1980	GRK6	Deinococcota	0.476	4.42E-03	3364	PDZD7	Kytococcaceae	0.122	5.23E-06	4748	THPO	Ustilaginaceae	0.059	1.85E-164
597	C3orf62	Actinomyces naeslundii	-0.252	1.57E-03	1981	GRK7	Pseudomonas oleovorans	-0.386	8.90E-06	3365	PDZK1	Halomonadaceae	-0.276	2.16E-05	4749	THRSP	Kytococcaceae	-0.202	2.18E-07
598	C3orf70	Aeromonadales	0.230	2.01E-03	1982	GRM6	Dolosigranulum pigrum	0.382	7.86E-12	3366	PDZRN4	Dolosigranulum pigrum	0.269	4.75E-03	4750	THSD7B	Corynebacterium tuberculostea	-0.125	2.46E-03
599	C4B	Actinomyces naeslundii	0.355	2.01E-03	1983	GRM7	Pseudomonas oleovorans	-0.183	5.06E-03	3367	PEBP1	Cutibacterium modestum	-0.677	2.08E-11	4751	THUMPD1	Ustilaginaceae	0.415	6.45E-07
600	C4BPA	Actinomyces naeslundii	0.295	1.01E-12	1984	GRN	Actinomyces naeslundii	0.296	9.16E-44	3368	PEMT	Actinomyces naeslundii	0.325	5.46E-07	4752	TIA1	Actinomyces naeslundii	-0.273	1.56E-10
601	C4orf47	Dolosigranulum pigrum	0.273	2.74E-13	1985	GRN	Leotiomycetes	-0.557	3.18E-03	3369	PEMT	Dolosigranulum pigrum	-0.405	5.88E-04	4753	TICRR	Pseudomonas oleovorans	-0.377	2.34E-05
602	C4orf54	Kytococcaceae	0.254	3.66E-04	1986	GRN	Paucibacter	-0.478	1.17E-08	3370	PEMT	Paucibacter	-0.547	3.13E-05	4754	TIE1	Paucibacter	-0.323	1.12E-04
603	C4orf54	Pseudomonas oleovorans	-0.303	2.19E-11	1987	GRPEL1	Actinomyces naeslundii	0.201	1.78E-48	3371	PEPD	Actinomyces naeslundii	0.380	2.46E-05	4755	TIGD1	Ustilaginaceae	0.213	1.93E-22
604	C5	Acinetobacter ursingii	0.817	1.16E-16	1988	GSAP	Ustilaginaceae	0.250	5.04E-05	3372	PES1	Escherichia coli	-0.501	6.77E-04	4756	TIGD7	Actinomyces naeslundii	-0.248	7.69E-03
605	C5AR2	Paucibacter	-0.196	9.33E-04	1989	GSDMB	Actinomyces naeslundii	-0.324	1.55E-26	3373	PEX11B	Kytococcaceae	-0.402	2.10E-03	4757	TIMM13	Dolosigranulum pigrum	-0.475	1.49E-03
606	C5orf15	Escherichia coli	0.521	5.97E-13	1990	GSDMC	Kytococcaceae	0.294	3.07E-08	3374	PEX11G	Dolosigranulum pigrum	-0.498	1.88E-04	4758	TIMM17A	Bradyrhizobium elkanii	0.683	2.49E-03
607	C5orf24	Escherichia coli	0.415	1.01E-04	1991	GSDMC	Pseudomonas oleovorans	-0.197	2.40E-36	3375	PEX13	Escherichia coli	0.349	2.00E-04	4759	TIMM17B	Gammaproteobacteria	-0.594	1.17E-04
608	C5orf63	Leotiomycetes	0.473	4.74E-03	1992	GSDMD	Escherichia coli	-0.462	6.50E-53	3376	PEX14	Escherichia coli	-0.435	2.03E-09	4760	TIMM22	Actinomyces naeslundii	0.187	2.79E-04
609	C5orf63	Ustilaginaceae	0.295	1.23E-05	1993	GSDMD	Paucibacter	-0.390	5.36E-05	3377	PEX16	Comamonadaceae	0.224	9.87E-06	4761	TIMM23	Bradyrhizobium elkanii	0.715	5.72E-03
610	C6orf52	Pseudomonas oleovorans	-0.261	5.68E-04	1994	GSG1L	Pseudomonas oleovorans	-0.488	9.76E-09	3378	PEX16	Escherichia coli	-0.355	1.00E-09	4762	TIMM29	Ustilaginaceae	-0.253	9.57E-03
611	C6orf62	Rhizobiaceae	-0.389	1.86E-06	1995	GSN	Kytococcaceae	0.223	3.21E-04	3379	PEX3	Bradyrhizobium elkanii	0.733	1.45E-09	4763	TIMM44	Escherichia coli	-0.387	2.20E-41
612	C7orf50	Escherichia coli	-0.424	4.42E-04	1996	GSS	Nostocales	-0.354	7.80E-03	3380	PEX5L	Paucibacter	0.558	2.11E-05	4764	TIMM50	Escherichia coli	-0.420	3.61E-06
613	C8G	Escherichia coli	-0.340	1.27E-05	1997	GSTA4	Schlegelella aquatica	-0.460	1.57E-03	3381	PEX5L	Pseudomonas oleovorans	-0.396	5.07E-51	4765	TIMP2	Pseudomonadaceae	0.675	1.47E-10
614	C8orf34	Pseudomonas oleovorans	-0.349	4.97E-10	1998	GSTZ1	Ottowia	-0.635	6.13E-03	3382	PEX5L	Streptomyces sp. T12	0.335	1.58E-08	4766	TIPARP	Leptotrichiaceae	0.336	3.55E-06
615	C8orf76	Thermales	-0.730	5.65E-05	1999	GTF2A1	Escherichia coli	0.593	1.76E-07	3383	PFDN5	Acinetobacter ursingii	-0.824	1.47E-18	4767	TIRAP	Bacillaceae	0.564	2.62E-04
616	C9	Escherichia coli	0.334	5.52E-03	2000	GTF2A1	Plantactinospora	0.525	3.70E-03	3384	PFDN6	Thermales	-0.772	6.87E-03	4768	TJAP1	Actinomyces naeslundii	-0.364	1.35E-33
617	C9orf78	Cutibacterium modestum	-0.703	1.06E-06	2001	GTF2A1	Ustilaginaceae	-0.100	3.83E-05	3385	PFKFB1	Staphylococcus epidermidis	-0.426	4.39E-03	4769	TJP3	Escherichia coli	-0.203	7.87E-14
618	CA8	Streptomyces sp. T12	0.327	4.70E-06	2002	GTF2A2	Escherichia coli	0.314	6.58E-09	3386	PFKFB2	Gordonia bronchialis	0.453	2.48E-03	4770	TKT	Actinomyces naeslundii	0.348	5.91E-04
619	CA9	Dolosigranulum pigrum	0.315	5.33E-04	2003	GTF2I	Acinetobacter ursingii	0.827	1.73E-14	3387	PFKL	Alphaproteobacteria	0.515	3.52E-05	4771	TLCD4	Escherichia coli	0.370	1.44E-04
620	CAAP1	Ceratobasidiaceae	0.405	5.07E-03	2004	GTF2IRD2	Actinomyces naeslundii	-0.218	4.99E-04	3388	PFKP	Kytococcaceae	0.170	4.77E-04	4772	TLK1	Escherichia coli	0.389	2.33E-05
621	CABIN1	Escherichia coli	-0.054	8.08E-03	2005	GTF2IRD2B	Actinomyces naeslundii	-0.528	1.17E-08	3389	PFN1	Ustilaginaceae	-0.211	2.23E-03	4773	TLK1	Rhizobiaceae	-0.334	4.14E-03
622	CABP1	Pseudomonas oleovorans	-0.343	5.99E-05	2006	GTF3C1	Escherichia coli	-0.174	4.11E-05	3390	PGAM1	Actinomyces naeslundii	0.416	1.92E-14	4774	TLL1	Pseudomonas oleovorans	-0.492	4.59E-04
623	CABP4	Paucibacter	-0.173	1.09E-12	2007	GTF3C5	Paucibacter	-0.356	2.44E-03	3391	PGAM1	Thermaceae	0.677	4.26E-04	4775	TLN2	Kytococcaceae	0.301	3.51E-04
624	CACFD1	Escherichia coli	-0.638	2.21E-26	2008	GTF3C6	Paucibacter	0.310	5.12E-09	3392	PGAM5	Nostocales	-0.486	8.34E-03	4776	TLNRD1	Escherichia coli	0.522	1.16E-27
625	CACNA1A	Kytococcaceae	0.015	1.89E-03	2009	GTPBP2	Actinomyces naeslundii	-0.213	5.54E-05	3393	PGAP2	Actinomyces naeslundii	-0.253	9.15E-10	4777	TLR10	Dolosigranulum pigrum	0.316	7.59E-05
626	CACNA1A	Pseudomonas oleovorans	-0.411	1.06E-11	2010	GTPBP6	Bradyrhizobium elkanii	-0.452	8.17E-04	3394	PGAP3	Dolosigranulum pigrum	-0.370	4.08E-04	4778	TLR2	Cellvibrionales	0.477	7.23E-03
627	CACNA1B	Pseudomonas oleovorans	-0.117	2.05E-06	2011	GTPBP8	Actinomyces naeslundii	0.110	6.13E-04	3395	PGAP6	Deinococcota	0.483	6.47E-04	4779	TLR5	Dolosigranulum pigrum	0.292	1.84E-04
628	CACNA1C	Kytococcaceae	0.073	6.49E-03	2012	GUCA1B	Actinomyces naeslundii	-0.377	3.52E-08	3396	PGBD2	Actinomyces naeslundii	-0.249	5.44E-07	4780	TM2D3	Bacillales	-0.520	2.43E-04
629	CACNA1D	Kytococcaceae	0.310	8.43E-10	2013	GUCA2B	Dolosigranulum pigrum	-0.320	1.30E-03	3397	PGC	Bacillaceae	-0.608	5.83E-16	4781	TM2D3	Yersiniaceae	0.566	4.17E-07
630	CACNA1E	Pseudomonas oleovorans	-0.324	1.77E-14	2014	GULP1	Pseudomonas oleovorans	-0.390	2.61E-06	3398	PGGHG	Ustilaginaceae	-0.019	1.59E-03	4782	TM4SF18	Paucibacter	-0.318	4.33E-03
631	CACNA1F	Kytococcaceae	0.213	6.61E-10	2015	GVQW3	Staphylococcus epidermidis	-0.363	2.10E-03	3399	PGGT1B	Bradyrhizobium elkanii	0.553	1.11E-03	4783	TM9SF1	Actinomyces naeslundii	0.294	2.15E-21
632	CACNA1F	Pseudomonas oleovorans	-0.264	1.45E-13	2016	H1-0	Actinomyces naeslundii	0.308	1.66E-06	3400	PGK1	Actinomyces naeslundii	0.356	3.24E-22	4784	TM9SF2	Actinomyces naeslundii	0.356	3.90E-34
633	CACNA1G	Pseudomonas oleovorans	-0.392	2.23E-16	2017	H1-3	Pseudomonas oleovorans	-0.467	7.22E-05	3401	PGLYRP2	Dolosigranulum pigrum	-0.485	1.49E-08	4785	TM9SF3	Escherichia coli	0.321	7.21E-09
634	CACNA1I	Pseudomonas oleovorans	-0.359	2.62E-72	2018	H1-5	Cutibacterium acnes	0.474	1.95E-49	3402	PGLYRP2	Paucibacter	-0.603	2.61E-03	4786	TM9SF3	Paucibacter	0.373	2.04E-10
635	CACNA1S	Kytococcaceae	0.300	2.10E-04	2019	H1-5	Fusobacterium	-0.052	8.91E-15	3403	PGM1	Actinomyces naeslundii	0.322	1.64E-14	4787	TMBIM1	Actinomyces naeslundii	0.449	8.13E-09
636	CACNB2	Actinomyces naeslundii	-0.225	6.40E-10	2020	H1-5	Paucibacter	-0.013	7.93E-09	3404	PHACTR2	Escherichia coli	0.588	9.11E-06	4788	TMBIM6	Actinomyces naeslundii	0.409	2.95E-14
637	CACNB3	Pseudomonas oleovorans	-0.297	9.44E-07	2021	H2AC6	Actinomyces naeslundii	0.322	1.89E-06	3405	PHACTR3	Dolosigranulum pigrum	0.075	6.69E-18	4789	TMC1	Kytococcaceae	0.193	3.76E-03
638	CACNB4	Pseudomonas oleovorans	-0.181	9.62E-04	2022	H2BC10	Cellvibrionales	0.190	1.57E-05	3406	PHB1	Actinomyces naeslundii	0.318	7.03E-12	4790	TMC2	Pseudomonas oleovorans	-0.367	1.16E-17
639	CACNG8	Pseudomonas oleovorans	-0.333	1.21E-07	2023	H2BC10	Paucibacter	-0.199	8.56E-04	3407	PHC1	Dolosigranulum pigrum	0.095	8.28E-06	4791	TMC5	Pseudomonas oleovorans	-0.315	5.62E-07
640	CACTIN	Bradyrhizobium elkanii	-0.502	1.57E-03	2024	H2BC15	Leotiomycetes	-0.477	5.52E-05	3408	PHC3	Escherichia coli	0.568	2.18E-06	4792	TMC6	Achromobacter	-0.717	7.24E-07
641	CACUL1	Bradyrhizobium elkanii	0.418	1.95E-04	2025	H2BC6	Paucibacter	-0.431	1.07E-08	3409	PHEX	Pseudomonas oleovorans	-0.141	6.87E-05	4793	TMC7	Pseudomonas oleovorans	-0.306	7.93E-04
642	CADPS	Pseudomonas oleovorans	-0.491	1.84E-07	2026	H2BC9	Cutibacterium acnes	0.381	2.97E-03	3410	PHF1	Actinomyces naeslundii	-0.569	2.13E-184	4794	TMC8	Cutibacterium acnes	0.473	8.36E-04
643	CALB1	Leotiomycetes	0.447	1.78E-04	2027	H3-3A	Ustilaginaceae	-0.035	4.25E-03	3411	PHF10	Cutibacterium acnes	-0.438	2.36E-05	4795	TMC8	Methylobacterium sp. FF17	-0.727	1.16E-08
644	CALB1	Pseudomonas oleovorans	-0.302	1.48E-24	2028	H4C15	Ustilaginaceae	0.336	2.19E-09	3412	PHF19	Kytococcaceae	0.370	8.51E-03	4796	TMCO3	Actinomyces naeslundii	0.291	2.18E-14
645	CALCR	Dolosigranulum pigrum	0.406	4.04E-03	2029	H4C4	Nocardioides	0.438	9.10E-04	3413	PHF21B	Kytococcaceae	0.189	7.44E-04	4797	TMED10	Actinomyces naeslundii	0.250	2.58E-111

646	CALCRL	Escherichia coli	0.582	7.00E-07	2030	HAAO	Paucibacter	-0.398	3.28E-52	3414	PHF21B	Pseudomonas oleovorans	-0.311	6.05E-12	4798	TMED7	Escherichia coli	0.452	1.50E-44
647	CALHM6	Escherichia coli	0.552	3.42E-04	2031	HABP2	Actinomyces naeslundii	0.418	4.58E-11	3415	PHF5A	Bradyrhizobium elkanii	0.476	5.47E-03	4799	TMEM100	Cutibacterium	-0.475	2.43E-04
648	CALM1	Ustilaginaceae	-0.279	6.25E-03	2032	HACD2	Actinomyces naeslundii	0.536	1.76E-05	3416	PHF7	Actinomyces naeslundii	-0.277	4.24E-03	4800	TMEM102	Dolosigranulum pigrum	-0.504	9.75E-18
649	CALM3	Actinomyces naeslundii	0.279	5.23E-04	2033	HACD3	Actinomyces naeslundii	0.480	1.25E-44	3417	PHGDH	Dolosigranulum pigrum	-0.529	3.92E-06	4801	TMEM104	Escherichia coli	-0.414	1.09E-12
650	CALML3	Escherichia coli	-0.341	4.25E-03	2034	HACD3	Ottowia	-0.498	5.37E-05	3418	PHIP	Thermaceae	-0.747	6.60E-06	4802	TMEM106B	Escherichia coli	0.490	3.06E-07
651	CALML3	Gordonia bronchialis	0.492	5.68E-03	2035	HACL1	Actinomyces naeslundii	0.414	1.42E-04	3419	PHKA1	Paucibacter	-0.214	1.54E-05	4803	TMEM109	Actinomyces naeslundii	0.170	7.18E-30
652	CALML6	Halomonadaceae	-0.181	9.08E-04	2036	HADH	Actinomyces naeslundii	0.420	1.24E-08	3420	PHKG1	Pseudomonas oleovorans	-0.395	7.59E-04	4804	TMEM120B	Ustilaginaceae	-0.194	2.37E-04
653	CALML6	Ustilaginaceae	-0.007	2.23E-12	2037	HADHA	Actinomyces naeslundii	0.324	8.77E-04	3421	PHLDB1	Bradyrhizobium elkanii	-0.678	1.14E-03	4805	TMEM121B	Pseudomonas oleovorans	-0.292	1.09E-04
654	CALN1	Kytococcaceae	0.122	5.12E-04	2038	HAGHL	Ustilaginaceae	-0.111	4.62E-05	3422	PHOSPHO1	Fusobacteriaceae	-0.315	5.48E-26	4806	TMEM123	Escherichia coli	0.255	2.32E-03
655	CALR	Actinomyces naeslundii	0.319	5.98E-26	2039	HAL	Kytococcaceae	0.285	1.61E-04	3423	PHPT1	Halomonadaceae	-0.332	6.14E-05	4807	TMEM125	Escherichia coli	-0.428	6.43E-03
656	CAMK1D	Streptococcaceae	0.483	8.41E-15	2040	HAPLN4	Halomonadaceae	-0.195	3.16E-05	3424	PHRF1	Bradyrhizobium elkanii	-0.526	2.24E-05	4808	TMEM127	Actinomyces naeslundii	0.235	5.88E-06
657	CAMK1G	Rhodocyclales	0.264	1.46E-04	2041	HARS1	Actinomyces naeslundii	-0.282	9.90E-12	3425	PHRF1	Escherichia coli	-0.214	2.92E-13	4809	TMEM131	Cutibacterium modestum	0.776	1.76E-16
658	CAMK2B	Escherichia coli	-0.358	1.17E-04	2042	HAS2	Dolosigranulum pigrum	0.418	2.19E-05	3426	PHTF1	Actinomyces naeslundii	0.299	6.71E-03	4810	TMEM132B	Pseudomonas oleovorans	-0.361	1.14E-08
659	CAMK2D	Escherichia coli	0.513	1.22E-07	2043	HAUS4	Leptotrichiaceae	0.261	2.41E-03	3427	PHYKPL	Ustilaginaceae	0.075	2.02E-07	4811	TMEM132C	Kytococcaceae	0.248	1.04E-07
660	CAMLG	Cutibacterium modestum	-0.656	3.27E-03	2044	HAUS4	Veillonella parvula	-0.445	4.18E-03	3428	PI4K2B	Gordonia bronchialis	-0.468	2.26E-03	4812	TMEM132D	Pseudomonas oleovorans	-0.166	4.12E-04
661	CAMSAP2	Escherichia coli	0.588	1.27E-04	2045	HAUS7	Actinomyces naeslundii	-0.426	1.82E-08	3429	PI4KA	Cutibacterium modestum	0.713	4.21E-14	4813	TMEM14B	Escherichia coli	0.152	8.76E-07
662	CAMSAP3	Escherichia coli	-0.191	2.25E-14	2046	HBB	Lawsonellaceae	-0.342	8.48E-03	3430	PIAS3	Bradyrhizobium elkanii	-0.497	6.58E-06	4814	TMEM14C	Escherichia coli	0.220	2.95E-14
663	CANX	Actinomyces naeslundii	0.385	1.47E-73	2047	HBD	Pseudomonas oleovorans	-0.165	6.12E-04	3431	PIDD1	Actinomyces naeslundii	-0.165	8.57E-58	4815	TMEM154	Bradyrhizobium elkanii	0.434	3.97E-03
664	CAPN12	Ustilaginaceae	0.140	3.85E-25	2048	HBP1	Pseudomonas oleovorans	0.469	1.04E-06	3432	PIDD1	Escherichia coli	-0.409	8.01E-23	4816	TMEM156	Dolosigranulum pigrum	0.359	9.26E-03
665	CAPN13	Pseudomonas oleovorans	-0.231	3.45E-07	2049	HBS1L	Actinomyces naeslundii	0.370	1.33E-82	3433	PIDD1	Lawsonellaceae	0.341	1.49E-06	4817	TMEM158	Kytococcaceae	0.168	8.57E-03
666	CAPN14	Pseudomonas oleovorans	-0.266	4.40E-09	2050	HCFC1	Cutibacterium modestum	0.709	1.12E-25	3434	PIEZO1	Bradyrhizobium elkanii	-0.623	1.72E-07	4818	TMEM160	Dolosigranulum pigrum	-0.422	1.48E-05
667	CAPN6	Paucibacter	0.355	4.20E-03	2051	HCFC2	Escherichia coli	0.392	4.50E-03	3435	PIEZO1	Escherichia coli	-0.139	1.08E-13	4819	TMEM161A	Bradyrhizobium elkanii	-0.436	2.34E-05
668	CAPN8	Kytococcaceae	0.171	8.57E-06	2052	HCN1	Pseudomonas oleovorans	-0.273	2.33E-07	3436	PIF1	Dolosigranulum pigrum	0.435	1.09E-05	4820	TMEM161A	Deinococcota	0.445	3.48E-03
669	CAPRIN2	Ustilaginaceae	0.295	2.04E-04	2053	HCRTR1	Kytococcaceae	0.236	4.42E-04	3437	PIGBOS1	Methyloversatilis sp. RAC08	-0.469	9.16E-03	4821	TMEM161A	Escherichia coli	-0.324	4.01E-34
670	CAPS	Ustilaginaceae	-0.018	5.15E-05	2054	HDAC10	Escherichia coli	-0.105	1.73E-06	3438	PIGK	Escherichia coli	0.294	6.53E-03	4822	TMEM164	Escherichia coli	-0.187	3.75E-03
671	CAPZA2	Escherichia coli	0.290	1.59E-16	2055	HDAC5	Bradyrhizobium elkanii	-0.482	8.88E-03	3439	PIGL	Actinomyces naeslundii	-0.276	1.61E-16	4823	TMEM165	Escherichia coli	0.357	1.18E-03
672	CAPZA3	Actinomyces naeslundii	-0.334	9.93E-07	2056	HDC	Dolosigranulum pigrum	0.415	5.03E-04	3440	PIGO	Dolosigranulum pigrum	-0.488	5.56E-03	4824	TMEM170B	Escherichia coli	0.308	2.40E-07
673	CAPZB	Ustilaginaceae	-0.188	4.48E-05	2057	HDGF	Lautropia	0.521	1.36E-03	3441	PIGP	Escherichia coli	0.258	7.32E-06	4825	TMEM175	Escherichia coli	-0.253	1.61E-10
674	CARD10	Bradyrhizobium elkanii	-0.525	1.29E-03	2058	HDGFL2	Escherichia coli	-0.296	1.54E-05	3442	PIGQ	Escherichia coli	-0.456	1.52E-19	4826	TMEM176A	Ustilaginaceae	-0.062	7.14E-03
675	CARD10	Deinococcota	0.450	1.90E-04	2059	HEATR1	Alphaproteobacteria	0.656	8.14E-03	3443	PIGR	Hyphomicrobiales	0.611	4.21E-05	4827	TMEM178A	Actinomyces naeslundii	-0.296	3.12E-17
676	CARD10	Escherichia coli	-0.251	5.10E-19	2060	HEBP2	Alphaproteobacteria	-0.595	1.36E-04	3444	PIGT	Actinomyces naeslundii	0.482	1.05E-08	4828	TMEM178B	Pseudomonas oleovorans	-0.366	3.41E-05
677	CARD19	Escherichia coli	-0.337	1.54E-03	2061	HECTD1	Acinetobacter ursingii	0.712	5.72E-03	3445	PIK3AP1	Actinomyces naeslundii	0.203	7.85E-11	4829	TMEM179B	Actinomyces naeslundii	0.318	5.60E-03
678	CARD8	Actinomyces naeslundii	-0.370	9.46E-06	2062	HECW1	Kytococcaceae	0.289	2.34E-05	3446	PIK3C2A	Escherichia coli	0.544	1.54E-08	4830	TMEM181	Paucibacter	0.420	8.39E-03
679	CARD9	Bradyrhizobium elkanii	-0.548	2.13E-08	2063	HECW2	Paucibacter	-0.264	5.87E-03	3447	PIK3CA	Escherichia coli	0.491	1.57E-06	4831	TMEM184A	Escherichia coli	-0.318	5.09E-05
680	CARNMT1	Escherichia coli	0.467	2.04E-06	2064	HECW2	Pseudomonadaceae	0.530	6.37E-03	3448	PIK3CB	Halomonadaceae	0.366	4.07E-04	4832	TMEM184C	Actinomyces naeslundii	0.581	8.98E-05
681	CASD1	Escherichia coli	0.565	5.40E-21	2065	HELQ	Actinomyces naeslundii	-0.206	2.98E-04	3449	PIK3R2	Paucibacter	-0.576	5.98E-03	4833	TMEM19	Actinomyces naeslundii	0.362	9.01E-41
682	CASP5	Dolosigranulum pigrum	0.319	4.63E-04	2066	HEMK1	Actinomyces naeslundii	-0.432	4.66E-18	3450	PIK3R5	Cutibacterium acnes	0.478	4.92E-03	4834	TMEM200A	Pseudomonas oleovorans	-0.529	6.95E-08
683	CASP7	Bradyrhizobium elkanii	0.635	7.61E-06	2067	HEPHL1	Escherichia coli	-0.450	1.34E-04	3451	PIK3R6	Dolosigranulum pigrum	0.273	1.06E-05	4835	TMEM201	Escherichia coli	-0.296	3.68E-03
684	CASP8AP2	Carnobacteriaceae	0.588	1.94E-11	2068	HERC3	Actinomyces naeslundii	0.316	2.38E-07	3452	PIM3	Cutibacterium acnes	0.377	3.79E-04	4836	TMEM203	Gardnerella	-0.560	2.01E-03
685	CASP9	Actinomyces naeslundii	-0.263	8.96E-09	2069	HEXB	Actinomyces naeslundii	0.391	3.84E-19	3453	PINK1	Hyphomicrobiales	0.619	1.97E-03	4837	TMEM209	Escherichia coli	0.410	2.61E-04
686	CASZ1	Kytococcaceae	0.372	1.62E-03	2070	HEXD	Actinomyces naeslundii	-0.206	1.26E-50	3454	PINX1	Escherichia coli	-0.482	8.21E-04	4838	TMEM220	Escherichia coli	0.401	8.67E-03
687	CAT	Kytococcaceae	-0.094	8.99E-07	2071	HEXIM1	Paucibacter	0.284	4.45E-03	3455	PIP4K2C	Actinomyces naeslundii	0.447	1.87E-07	4839	TMEM220	Kytococcaceae	-0.411	3.00E-03
688	CATSPERG	Kytococcaceae	0.213	2.24E-10	2072	HFM1	Pseudomonas oleovorans	-0.250	3.22E-08	3456	PIP4P1	Plantactinospora	-0.468	2.60E-03	4840	TMEM230	Escherichia coli	0.185	7.59E-03
689	CAVIN4	Pseudomonas oleovorans	-0.312	1.03E-03	2073	HGF	Escherichia coli	0.418	1.24E-08	3457	PIP5K1A	Thermaceae	-0.657	1.04E-04	4841	TMEM232	Pseudomonas oleovorans	-0.325	1.02E-03
690	CBFA2T2	Staphylococcus epidermidis	-0.490	5.38E-03	2074	HGF	Ottowia	0.451	2.02E-04	3458	PIP5K1B	Paucibacter	0.562	3.11E-03	4842	TMEM234	Actinomyces naeslundii	-0.454	2.12E-07
691	CBFB	Escherichia coli	0.615	8.55E-23	2075	HGFAC	Escherichia coli	-0.233	7.92E-07	3459	PIPOX	Dolosigranulum pigrum	-0.340	1.60E-08	4843	TMEM245	Enterobacterales	0.409	9.90E-03
692	CBFB	Streptococcus oralis	-0.494	6.88E-04	2076	HGS	Bradyrhizobium elkanii	-0.529	6.58E-03	3460	PIR	Paucibacter	0.385	1.73E-09	4844	TMEM249	Ustilaginaceae	0.128	7.65E-04
693	CBLL1	Escherichia coli	0.472	9.53E-03	2077	HHEX	Leptotrichiaceae	-0.159	1.13E-04	3461	PITPNM1	Escherichia coli	-0.170	3.83E-34	4845	TMEM255B	Paucibacter	-0.201	3.02E-04
694	CBX3	Paucibacter	0.486	1.01E-12	2078	HHIP	Escherichia coli	0.421	2.61E-03	3462	PITPNM1	Yersiniaceae	-0.584	4.95E-15	4846	TMEM256	Thermales	-0.739	6.39E-05
695	CBX5	Actinomyces naeslundii	0.424	1.55E-20	2079	HIGD2A	Ustilaginaceae	-0.389	1.70E-16	3463	PJA2	Escherichia coli	0.400	3.45E-03	4847	TMEM259	Escherichia coli	-0.230	6.00E-10
696	CC2D1A	Escherichia coli	-0.192	6.43E-07	2080	HINT2	Cutibacterium modestum	-0.727	7.72E-08	3464	PJVK	Actinomyces naeslundii	-0.103	2.48E-03	4848	TMEM259	Paucibacter	-0.339	1.64E-04
697	CC2D2B	Pseudomonas oleovorans	-0.320	1.98E-07	2081	HINT3	Paucibacter	0.240	3.90E-08	3465	PKD1	Escherichia coli	-0.160	3.26E-09	4849	TMEM263	Alphaproteobacteria	-0.543	3.05E-04
698	CCAR2	Escherichia coli	-0.308	1.32E-05	2082	HIP1R	Escherichia coli	-0.295	1.06E-08	3466	PKD1L1	Actinomyces naeslundii	-0.379	1.96E-03	4850	TMEM265	Actinomyces naeslundii	-0.357	2.42E-17
699	CCDC106	Escherichia coli	-0.398	8.07E-12	2083	HIPK2	Acinetobacter ursingii	0.763	2.12E-13	3467	PKHD1L1	Streptomyces sp. T12	0.327	3.47E-04	4851	TMEM265	Ustilaginaceae	0.100	3.84E-03

700	CCDC107	Escherichia coli	-0.536	3.76E-31	2084	HIPK2	Actinomyces naeslundii	0.388	3.18E-08	3468	PKLR	Dolosigranulum pigrum	-0.383	6.18E-03	4852	TMEM266	Escherichia coli	-0.684	2.63E-03
701	CCDC112	Dolosigranulum pigrum	0.437	7.65E-25	2085	HIPK3	Escherichia coli	0.666	1.40E-10	3469	PKN3	Mycobacteriaceae	0.354	7.45E-03	4853	TMEM30B	Escherichia coli	0.365	4.06E-03
702	CCDC115	Bradyrhizobium elkanii	0.552	1.53E-06	2086	HK1	Thermales	0.578	5.08E-04	3470	PKP2	Actinomyces naeslundii	0.509	7.68E-04	4854	TMEM35B	Escherichia coli	0.425	4.77E-08
703	CCDC117	Paucibacter	0.253	4.65E-10	2087	HK3	Gordonia bronchialis	0.590	1.84E-05	3471	PLA2G6	Actinomyces naeslundii	-0.236	2.50E-13	4855	TMEM38A	Actinomyces naeslundii	0.330	6.03E-05
704	CCDC124	Escherichia coli	-0.339	3.02E-20	2088	HLA-A	Actinomyces naeslundii	0.147	2.62E-11	3472	PLA2G7	Escherichia coli	0.513	2.10E-05	4856	TMEM47	Escherichia coli	0.458	4.30E-18
705	CCDC136	Kytococcaceae	0.333	2.89E-05	2089	HLA-A	Nocardioides	0.337	1.70E-05	3473	PLAC8	Escherichia coli	0.535	3.48E-31	4857	TMEM50A	Escherichia coli	0.348	4.22E-08
706	CCDC14	Ustilaginaceae	0.301	4.11E-04	2090	HLA-DPA1	Ustilaginaceae	-0.177	2.58E-04	3474	PLAC8L1	Halomonadaceae	-0.230	8.94E-04	4858	TMEM53	Ustilaginaceae	-0.218	9.16E-03
707	CCDC144A	Staphylococcus hominis	0.421	1.78E-03	2091	HLA-DPB1	Escherichia coli	0.479	9.90E-03	3475	PLAC8L1	Ustilaginaceae	0.176	1.74E-04	4859	TMEM54	Escherichia coli	-0.475	6.08E-04
708	CCDC148	Chitinophagaceae	0.388	6.15E-23	2092	HLA-DQA2	Bacteroidales	-0.482	3.12E-08	3476	PLBD2	Thermaceae	0.608	1.76E-05	4860	TMEM60	Gordonia bronchialis	-0.609	5.85E-48
709	CCDC148	Kytococcaceae	0.100	1.37E-06	2093	HLA-DRA	Escherichia coli	0.480	1.23E-03	3477	PLCB2	Bradyrhizobium elkanii	-0.694	1.02E-05	4861	TMEM65	Escherichia coli	0.616	5.13E-07
710	CCDC150	Actinomyces naeslundii	-0.107	6.73E-05	2094	HLA-E	Ustilaginaceae	-0.227	7.42E-05	3478	PLCB3	Cutibacterium modestum	0.561	8.82E-06	4862	TMEM67	Dolosigranulum pigrum	0.349	1.09E-03
711	CCDC150	Staphylococcus epidermidis	-0.302	1.17E-03	2095	HLTF	Actinomyces naeslundii	0.567	2.14E-06	3479	PLCD1	Paucibacter	-0.538	2.12E-04	4863	TMEM71	Dolosigranulum pigrum	0.391	1.75E-06
712	CCDC150	Ustilaginaceae	0.198	1.16E-04	2096	HLTF	Bacillaceae	0.486	2.21E-05	3480	PLCE1	Streptomyces sp. T12	0.398	6.67E-03	4864	TMEM74B	Actinomyces naeslundii	-0.317	1.79E-09
713	CCDC159	Actinomyces naeslundii	-0.280	6.49E-113	2097	HMG20B	Escherichia coli	-0.365	4.03E-21	3481	PLCG1	Bradyrhizobium elkanii	-0.577	1.99E-06	4865	TMEM82	Dolosigranulum pigrum	-0.373	8.87E-04
714	CCDC159	Ustilaginaceae	-0.009	1.09E-03	2098	HMGA2	Pseudomonas oleovorans	-0.189	2.70E-09	3482	PLCH1	Kytococcaceae	0.188	8.58E-05	4866	TMEM82	Xanthomonadales	0.288	9.68E-04
715	CCDC168	Kytococcaceae	0.257	2.15E-04	2099	HMGN1	Acinetobacter ursingii	-0.688	5.39E-11	3483	PLCL1	Pseudomonas oleovorans	-0.476	1.52E-04	4867	TMEM91	Actinomyces naeslundii	-0.519	1.37E-47
716	CCDC17	Actinomyces naeslundii	-0.127	1.15E-12	2100	HMGN2	Novosphingobium sp. EMRT-2	-0.639	6.79E-45	3484	PLCZ1	Pseudomonas oleovorans	-0.322	4.10E-08	4868	TMOD3	Actinomyces naeslundii	0.298	1.32E-14
717	CCDC170	Dolosigranulum pigrum	0.316	4.12E-03	2101	HMGN3	Actinomyces naeslundii	-0.277	1.00E-09	3485	PLD3	Alphaproteobacteria	0.555	2.46E-05	4869	TMPRSS2	Actinomyces naeslundii	-0.284	2.90E-03
718	CCDC171	Pseudomonas oleovorans	-0.213	3.17E-06	2102	HMGN3	Thermaceae	-0.737	2.58E-05	3486	PLD3	Leotiomycetes	-0.551	1.94E-03	4870	TMPRSS9	Escherichia coli	-0.380	1.84E-04
719	CCDC178	Pseudomonas oleovorans	-0.329	8.56E-11	2103	HMGN4	Dolosigranulum pigrum	0.463	9.53E-04	3487	PLD5	Pseudomonas oleovorans	-0.221	2.01E-04	4871	TMSB4X	Escherichia coli	0.328	1.74E-04
720	CCDC187	Kytococcaceae	0.205	3.82E-06	2104	HMGN5	Thermales	-0.667	3.06E-04	3488	PLEC	Deinococcota	0.554	2.75E-10	4872	TMSB4Y	Debaryomycetaceae	0.601	6.59E-05
721	CCDC187	Pseudomonas oleovorans	-0.240	3.00E-12	2105	HMGXB3	Paucibacter	-0.161	6.30E-03	3489	PLEK	Escherichia coli	0.589	6.06E-04	4873	TMSB4Y	Rhodoferax	0.243	3.52E-06
722	CCDC188	Dolosigranulum pigrum	0.257	4.43E-74	2106	HMGXB3	Ustilaginaceae	-0.034	5.04E-03	3490	PLEKHA1	Escherichia coli	0.341	3.99E-05	4874	TMTC3	Escherichia coli	0.382	5.62E-03
723	CCDC198	Ustilaginaceae	0.226	4.39E-03	2107	HNMT	Gordonia bronchialis	-0.513	1.19E-03	3491	PLEKHA3	Escherichia coli	0.356	6.07E-06	4875	TMTC3	Kytococcaceae	-0.248	3.68E-05
724	CCDC22	Escherichia coli	-0.219	4.82E-05	2108	HNRNPA0	Paucibacter	0.407	1.56E-03	3492	PLEKHA4	Kytococcaceae	0.136	8.20E-05	4876	TMUB1	Escherichia coli	-0.291	1.22E-12
725	CCDC24	Ustilaginaceae	0.213	1.33E-20	2109	HNRNPA1	Paucibacter	0.257	2.92E-03	3493	PLEKHA7	Acinetobacter baumannii	0.271	2.40E-07	4877	TMX1	Escherichia coli	0.349	3.67E-04
726	CCDC28B	Actinomyces naeslundii	-0.221	2.66E-05	2110	HNRNPA1L2	Ustilaginaceae	0.046	1.32E-07	3494	PLEKHD1	Pseudomonas oleovorans	-0.281	2.37E-08	4878	TMX3	Actinomyces naeslundii	-0.249	2.73E-11
727	CCDC3	Ustilaginaceae	-0.164	2.80E-03	2111	HNRNPAB	Campylobacterota	0.397	4.65E-03	3495	PLEKHF2	Escherichia coli	0.429	2.44E-06	4879	TMX4	Plantactinospora	0.334	1.19E-04
728	CCDC30	Pseudomonas oleovorans	-0.318	7.39E-04	2112	HNRNPD	Hyphomicrobiales	-0.630	3.30E-41	3496	PLEKHG1	Paucibacter	-0.080	6.46E-03	4880	TNFAIP3	Actinomyces naeslundii	-0.379	2.67E-06
729	CCDC47	Actinomyces naeslundii	0.454	4.97E-45	2113	HNRNPDL	Actinomyces naeslundii	-0.287	2.35E-33	3497	PLEKHG4B	Kytococcaceae	0.115	1.79E-06	4881	TNFAIP8L1	Ottowia	-0.708	6.05E-04
730	CCDC47	Paucibacter	0.118	3.39E-03	2114	HNRNPH3	Actinomyces naeslundii	-0.383	1.73E-05	3498	PLEKHG4B	Pseudomonas oleovorans	-0.368	9.25E-07	4882	TNFRSF10B	Actinomyces naeslundii	-0.260	4.73E-20
731	CCDC59	Methyloversatilis sp. RAC08	-0.445	6.69E-03	2115	HNRNPH3	Hyphomicrobiales	-0.550	9.20E-05	3499	PLEKHG5	Bradyrhizobium elkanii	-0.675	5.49E-05	4883	TNFRSF12A	Kytococcaceae	0.046	2.30E-11
732	CCDC6	Plantactinospora	0.648	8.62E-03	2116	HNRNPLL	Escherichia coli	0.318	2.33E-05	3500	PLEKHH1	Leptotrichiaceae	-0.151	1.48E-03	4884	TNFRSF14	Actinomyces naeslundii	-0.330	1.70E-25
733	CCDC61	Escherichia coli	-0.301	1.44E-06	2117	HNRNPUL1	Thermaceae	0.747	2.76E-08	3501	PLEKHJ1	Escherichia coli	-0.365	1.45E-18	4885	TNFRSF17	Dolosigranulum pigrum	0.350	1.05E-03
734	CCDC68	Actinomyces naeslundii	-0.277	4.72E-03	2118	HOXA13	Pseudomonas oleovorans	-0.264	1.44E-06	3502	PLEKHM3	Acinetobacter ursingii	0.728	2.41E-13	4886	TNFRSF17	Paucibacter	0.453	3.60E-03
735	CCDC69	Actinomyces naeslundii	0.504	7.64E-04	2119	HOXA3	Dolosigranulum pigrum	0.222	1.45E-03	3503	PLEKHS1	Kytococcaceae	0.238	2.24E-08	4887	TNFRSF21	Escherichia coli	0.593	1.31E-03
736	CCDC7	Pseudomonas oleovorans	-0.260	1.78E-06	2120	HOXA3	Paucibacter	0.533	5.50E-04	3504	PLG	Actinomyces naeslundii	0.447	1.32E-08	4888	TNK1	Acidovorax temperans	0.353	7.44E-04
737	CCDC71	Dolosigranulum pigrum	-0.309	3.20E-04	2121	HOXB7	Dolosigranulum pigrum	0.412	1.14E-07	3505	PLG	Dolosigranulum pigrum	-0.290	2.33E-03	4889	TNK1	Escherichia coli	-0.410	1.28E-07
738	CCDC82	Dolosigranulum pigrum	0.387	7.21E-03	2122	HPGD	Bradyrhizobium elkanii	0.527	4.47E-03	3506	PLGRKT	Bradyrhizobium elkanii	0.587	1.16E-06	4890	TNKS2	Escherichia coli	0.543	1.83E-05
739	CCDC87	Kytococcaceae	0.179	1.75E-04	2123	HPS3	Bacillus velezensis	0.434	2.50E-03	3507	PLIN3	Actinomyces naeslundii	0.110	6.23E-06	4891	TNNI2	Paenibacillaceae	0.235	4.60E-03
740	CCDC88A	Thermaceae	-0.714	2.27E-08	2124	HPS6	Actinomyces naeslundii	0.145	4.38E-05	3508	PLIN4	Escherichia coli	-0.495	2.21E-04	4892	TNNT2	Ustilaginaceae	0.131	2.88E-04
741	CCDC88C	Bradyrhizobium elkanii	-0.778	4.96E-04	2125	HR	Pseudomonas oleovorans	-0.184	1.77E-06	3509	PLIN5	Actinomyces naeslundii	-0.401	5.74E-29	4893	TNR	Schlegelella aquatica	0.201	9.42E-03
742	CCDC90B	Bradyrhizobium elkanii	0.652	7.10E-05	2126	HRH4	Pseudomonas oleovorans	-0.244	2.86E-03	3510	PLK1	Pseudomonas oleovorans	-0.279	4.53E-06	4894	TNRC6A	Actinomyces naeslundii	0.007	7.02E-04
743	CCDC90B	Cutibacterium modestum	-0.683	4.52E-04	2127	HS3ST1	Pseudomonas oleovorans	-0.404	1.97E-03	3511	PLK3	Kytococcaceae	-0.091	3.89E-07	4895	TNS1	Bradyrhizobium elkanii	-0.522	4.01E-04
744	CCDC92	Bradyrhizobium elkanii	-0.418	4.37E-03	2128	HS6ST2	Paucibacter	0.511	2.85E-04	3512	PLOD2	Escherichia coli	0.361	5.27E-06	4896	TOB1	Escherichia coli	0.483	3.25E-05
745	CCDC96	Kytococcaceae	0.121	1.61E-04	2129	HS6ST2	Xanthomonadales	-0.361	8.21E-03	3513	PLPP5	Ceratobasidiaceae	0.420	1.33E-03	4897	TOB1	Rhodobacterales	-0.310	6.46E-03
746	CCER2	Ustilaginaceae	0.053	1.96E-03	2130	HSBP1	Cutibacterium modestum	-0.609	5.83E-05	3514	PLPP5	Dolosigranulum pigrum	0.265	6.35E-03	4898	TOGARAM2	Achromobacter deleyi	-0.552	2.37E-08
747	CCHCR1	Actinomyces naeslundii	-0.349	3.19E-12	2131	HSD11B1L	Halomonadaceae	-0.408	2.91E-04	3515	PLPP7	Dolosigranulum pigrum	0.207	1.31E-16	4899	TOLLIP	Dolosigranulum pigrum	-0.556	2.74E-07
748	CCL2	Dolosigranulum pigrum	0.489	5.23E-03	2132	HSD17B11	Ustilaginaceae	-0.135	9.48E-03	3516	PLPPR2	Ustilaginaceae	-0.033	2.92E-03	4900	TOM1	Escherichia coli	-0.329	3.77E-08
749	CCL20	Dolosigranulum pigrum	0.216	1.09E-04	2133	HSD17B8	Lawsonellaceae	-0.627	1.35E-06	3517	PLPPR4	Streptomyces sp. T12	0.325	3.60E-04	4901	TOM1L1	Actinomyces naeslundii	0.499	2.20E-04
750	CCL24	Thermaceae	0.584	3.56E-05	2134	HSD3B2	Actinomyces naeslundii	-0.246	7.23E-03	3518	PLS3	Actinomyces naeslundii	0.443	4.83E-58	4902	TOM1L2	Bradyrhizobium elkanii	-0.504	7.20E-03
751	CCM2	Escherichia coli	-0.555	2.51E-16	2135	HSD3B7	Actinomyces naeslundii	0.196	7.52E-06	3519	PLSCR1	Schlegelella aquatica	0.264	4.41E-05	4903	TOMM20	Paucibacter	0.293	2.49E-05
752	CCN6	Actinomyces naeslundii	-0.408	3.99E-11	2136	HSD3B7	Nocardioides	0.192	2.73E-04	3520	PLSCR2	Pseudomonas oleovorans	-0.137	2.03E-04	4904	TOMM22	Ustilaginaceae	-0.271	9.34E-29
753	CCN6	Spirochaetota	0.292	2.73E-03	2137	HSD3B7	Paucibacter	-0.564	1.52E-05	3521	PLSCR4	Escherichia coli	0.578	6.94E-04	4905	TOMM40	Enterobacterales	-0.469	2.01E-03

754	CCNC	Cutibacterium modestum	-0.604	1.49E-10	2138	HSDL2	Escherichia coli	0.307	4.01E-03	3522	PLSCR4	Halomonadaceae	0.147	7.24E-03	4906	TOMM40L	Ustilaginaceae	0.274	3.01E-04
755	CCNE2	Dolosigranulum pigrum	0.357	1.86E-06	2139	HSF1	Escherichia coli	-0.203	2.32E-22	3523	PLXDC1	Actinomyces naeslundii	-0.375	5.51E-03	4907	TOMM7	Paucibacter	0.279	7.29E-12
756	CCNG2	Escherichia coli	0.350	4.51E-06	2140	HSF5	Pseudomonas oleovorans	-0.245	1.40E-03	3524	PLXNA1	Cutibacterium modestum	0.645	1.58E-09	4908	TOMM70	Plantactinospora	0.313	8.56E-09
757	CCNI2	Pseudomonas oleovorans	-0.014	7.13E-08	2141	HSH2D	Actinomyces naeslundii	-0.424	4.50E-08	3525	PLXNB2	Cutibacterium modestum	0.620	3.02E-05	4909	TONSL	Escherichia coli	-0.359	2.11E-06
758	CCNK	Thermaceae	0.675	9.46E-03	2142	HSPA2	Dolosigranulum pigrum	0.258	7.76E-04	3526	PLXNB2	Deinococcota	0.582	4.91E-19	4910	TOP1MT	Escherichia coli	-0.320	8.14E-06
759	CCNK	Yersiniaceae	-0.642	1.23E-03	2143	HSPB6	Dolosigranulum pigrum	0.255	1.84E-04	3527	PLXNB3	Kytococcaceae	0.171	5.01E-04	4911	TOP1MT	Halomonadaceae	-0.373	4.08E-09
760	CCNL1	Actinomyces naeslundii	-0.143	5.00E-05	2144	HSPBAP1	Actinomyces naeslundii	-0.227	1.96E-03	3528	PLXND1	Paucibacter	-0.296	1.39E-03	4912	TOP3B	Escherichia coli	-0.296	2.49E-06
761	CCNL2	Actinomyces naeslundii	-0.203	4.03E-35	2145	HSPD1	Actinomyces naeslundii	0.404	1.73E-08	3529	PMFBP1	Pseudomonas oleovorans	-0.444	5.21E-05	4913	TOP3B	Yersiniaceae	-0.548	6.72E-05
762	CCNQ	Enterobacterales	-0.499	3.40E-03	2146	HSPG2	Cutibacterium modestum	0.704	5.15E-11	3530	PMPCA	Bradyrhizobium elkanii	-0.520	2.70E-18	4914	TOR1A	Actinomyces naeslundii	0.221	1.41E-11
763	CCPG1	Alphaproteobacteria	-0.454	1.13E-07	2147	HTATIP2	Bradyrhizobium elkanii	0.497	1.14E-05	3531	PMPCA	Escherichia coli	-0.250	3.70E-15	4915	TOX	Dolosigranulum pigrum	0.238	5.35E-05
764	CCPG1	Escherichia coli	0.267	1.34E-09	2148	HTR2A	Acinetobacter johnsonii	0.496	3.00E-03	3532	PMS1	Actinomyces naeslundii	-0.137	2.64E-06	4916	TP53AIP1	Kytococcaceae	0.093	1.31E-28
765	CCR1	Escherichia coli	0.593	1.32E-04	2149	HTR2A	Pseudomonas oleovorans	-0.357	1.36E-12	3533	PNISR	Actinomyces naeslundii	-0.152	9.70E-10	4917	TP53AIP1	Pseudomonas oleovorans	-0.276	2.23E-06
766	CCR10	Actinomyces naeslundii	-0.332	1.41E-04	2150	HTR7	Dolosigranulum pigrum	0.262	4.06E-03	3534	PNISR	Thermaceae	-0.648	9.49E-05	4918	TP53I13	Escherichia coli	-0.304	9.52E-04
767	CCS	Actinomyces naeslundii	-0.392	4.55E-10	2151	HTT	Escherichia coli	-0.263	6.15E-07	3535	PNISR	Ustilaginaceae	0.254	5.32E-06	4919	TP53TG5	Bacillaceae	0.445	1.41E-116
768	CCSAP	Actinomyces naeslundii	-0.325	5.54E-11	2152	HTT	Paucibacter	-0.377	1.03E-03	3536	PNMA2	Dolosigranulum pigrum	0.198	7.28E-03	4920	TP53TG5	Plantactinospora sp. BC1	-0.345	2.08E-73
769	CCSER1	Pseudomonas oleovorans	-0.214	1.92E-09	2153	HTT	Staphylococcus epidermidis	-0.371	7.40E-03	3537	PNMA3	Kytococcaceae	0.020	6.29E-03	4921	TP63	Pseudomonas oleovorans	-0.200	2.74E-03
770	CCSER2	Dolosigranulum	0.280	5.49E-03	2154	HUWE1	Acinetobacter ursingii	0.826	4.62E-14	3538	PNMA6A	Chitinophagaceae	-0.486	2.96E-05	4922	TP73	Pseudomonas oleovorans	-0.300	1.60E-04
771	CCT3	Propionibacteriaceae	-0.595	1.09E-04	2155	HYOU1	Leptotrichiaceae	-0.294	2.90E-04	3539	PNMT	Moraxellaceae	-0.394	2.13E-03	4923	TPBG	Dolosigranulum pigrum	0.207	4.98E-03
772	CCT4	Bradyrhizobium elkanii	0.632	3.63E-14	2156	IARS2	Actinomyces naeslundii	0.500	3.74E-40	3540	PNMT	Ustilaginaceae	0.221	2.76E-03	4924	TPD52	Alphaproteobacteria	-0.521	2.65E-04
773	CCT7	Actinomyces naeslundii	0.230	4.44E-08	2157	ICA1L	Streptomyces sp. T12	0.443	1.17E-03	3541	PNN	Ustilaginaceae	0.260	2.38E-03	4925	TPI1	Ustilaginaceae	-0.142	4.71E-03
774	CCZ1B	Dolosigranulum pigrum	0.420	1.94E-03	2158	ICMT	Actinomyces naeslundii	0.438	5.49E-06	3542	PNO1	Methylobacterium sp. FF17	0.551	1.96E-03	4926	TPM1	Actinomyces naeslundii	-0.235	3.62E-03
775	CD163	Paucibacter	-0.306	4.83E-06	2159	ID2	Leptotrichiaceae	0.173	2.82E-04	3543	PNPLA2	Bradyrhizobium elkanii	-0.559	4.16E-05	4927	TPM3	Ustilaginaceae	-0.213	2.04E-07
776	CD164	Escherichia coli	0.418	3.15E-27	2160	ID3	Ustilaginaceae	-0.385	3.03E-04	3544	PNPLA3	Bacillaceae	0.380	1.32E-03	4928	TPP1	Actinomyces naeslundii	0.355	3.68E-09
777	CD180	Veillonella parvula	0.395	8.36E-03	2161	ID4	Dolosigranulum pigrum	0.364	1.64E-03	3545	PNPLA3	Escherichia coli	-0.309	1.16E-04	4929	TPP1	Thermaceae	0.633	9.13E-04
778	CD1B	Anaerococcus	0.333	6.25E-09	2162	IDH2	Actinomyces naeslundii	0.395	2.55E-19	3546	PNPLA4	Rhodoferax	-0.355	1.67E-03	4930	TPPP2	Actinomyces naeslundii	-0.179	3.35E-08
779	CD1B	Pseudomonas oleovorans	-0.225	3.30E-27	2163	IDH3A	Yersiniaceae	-0.580	8.67E-48	3547	PNPO	Dolosigranulum pigrum	-0.306	2.00E-07	4931	TPPP3	Actinomyces naeslundii	-0.454	1.34E-08
780	CD209	Thermaceae	0.559	1.19E-05	2164	IDH3B	Gardnerella	0.354	1.21E-04	3548	POC1B	Escherichia coli	0.383	3.75E-03	4932	TPRA1	Enterobacterales	-0.554	5.41E-04
781	CD2AP	Escherichia coli	0.540	3.80E-08	2165	IDO2	Dermacoccaceae	-0.269	3.98E-04	3549	PODNL1	Kytococcaceae	0.132	7.64E-05	4933	TPRA1	Escherichia coli	-0.378	1.57E-04
782	CD320	Escherichia coli	-0.274	9.67E-05	2166	IDS	Leptotrichiaceae	0.307	6.48E-03	3550	PODNL1	Pseudomonas oleovorans	-0.248	1.02E-11	4934	TPRA1	Paucibacter	-0.466	2.66E-04
783	CD36	Paucibacter	-0.442	1.29E-04	2167	IDUA	Actinomyces naeslundii	-0.210	0.00E+00	3551	POFUT2	Ustilaginaceae	-0.163	1.71E-03	4935	TPRKB	Cutibacterium modestum	-0.687	4.48E-08
784	CD37	Saccharomycetaceae	0.126	6.15E-03	2168	IER3IP1	Escherichia coli	0.152	2.81E-06	3552	POGLUT2	Actinomyces naeslundii	0.461	4.17E-05	4936	TPSD1	Mycobacteriaceae	0.371	5.70E-03
785	CD3E	Bacteroidota	-0.093	1.82E-03	2169	IER5L	Kytococcaceae	0.044	2.98E-07	3553	POLB	Oscillospiraceae	0.461	5.09E-06	4937	TPTE2	Pseudomonas oleovorans	-0.119	2.15E-14
786	CD3E	Glomerellaceae	0.475	2.30E-03	2170	IFI30	Actinomyces naeslundii	0.252	1.24E-20	3554	POLD1	Cutibacterium modestum	0.471	1.83E-06	4938	TRA2A	Actinomyces naeslundii	-0.427	2.25E-09
787	CD47	Escherichia coli	0.257	1.53E-09	2171	IFI44	Staphylococcus hominis	-0.449	2.80E-04	3555	POLD1	Escherichia coli	-0.360	6.35E-16	4939	TRAF1	Actinomyces naeslundii	-0.322	1.15E-09
788	CD53	Escherichia coli	0.567	1.79E-03	2172	IFI44L	Methyloversatilis sp. RAC08	-0.276	5.20E-06	3556	POLDIP2	Actinomyces naeslundii	0.296	1.70E-05	4940	TRAF1	Ustilaginaceae	0.037	5.23E-03
789	CD5L	Paucibacter	-0.309	1.79E-06	2173	IFI6	Bradyrhizobium elkanii	0.390	2.42E-05	3557	POLDIP2	Dolosigranulum pigrum	-0.408	8.56E-03	4941	TRAF3IP2	Brucella	0.424	6.92E-03
790	CD68	Actinomyces naeslundii	0.338	7.10E-13	2174	IFI6	Micromonosporales	-0.456	2.15E-03	3558	POLG	Alphaproteobacteria	0.520	1.15E-04	4942	TRAF4	Actinomyces naeslundii	-0.331	1.57E-12
791	CD68	Paucibacter	-0.457	5.47E-09	2175	IFIT5	Escherichia coli	0.433	6.06E-06	3559	POLG2	Actinomyces naeslundii	-0.293	9.65E-06	4943	TRAF4	Plantactinospora	-0.544	2.60E-06
792	CD81	Escherichia coli	-0.396	2.63E-03	2176	IFITM10	Actinomyces naeslundii	-0.368	8.63E-66	3560	POLK	Escherichia coli	0.446	3.27E-03	4944	TRAF5	Actinomyces naeslundii	-0.467	9.20E-21
793	CD82	Ceratobasidiaceae	-0.372	3.63E-21	2177	IFNAR1	Paucibacter	0.265	2.78E-05	3561	POLL	Actinomyces naeslundii	-0.248	6.07E-18	4945	TRAK1	Actinomyces naeslundii	-0.238	6.65E-60
794	CD82	Leotiomycetes	-0.520	1.02E-09	2178	IFRD1	Actinomyces naeslundii	-0.261	1.67E-07	3562	POLN	Escherichia coli	-0.446	5.39E-04	4946	TRAK1	Staphylococcus epidermidis	-0.324	4.42E-10
795	CD82	Ustilaginaceae	-0.245	8.78E-42	2179	IFT27	Pseudomonas oleovorans	-0.586	2.64E-10	3563	POLR1A	Methyloversatilis sp. RAC08	0.353	2.54E-05	4947	TRAM1	Escherichia coli	0.338	5.29E-06
796	CDC123	Actinomyces naeslundii	0.465	6.97E-10	2180	IFT43	Acinetobacter ursingii	-0.774	3.11E-08	3564	POLR1D	Actinomyces naeslundii	-0.294	9.76E-12	4948	TRAP1	Dolosigranulum pigrum	-0.508	1.22E-21
797	CDC16	Halomonadaceae	-0.204	5.43E-03	2181	IFT74	Dolosigranulum pigrum	0.404	1.48E-03	3565	POLR1D	Ottowia	-0.413	9.64E-03	4949	TRAPPC12	Thermaceae	0.642	6.65E-04
798	CDC16	Ustilaginaceae	0.209	1.45E-06	2182	IFT81	Actinomyces naeslundii	-0.175	1.42E-03	3566	POLR2E	Leotiomycetes	-0.644	7.08E-05	4950	TRAPPC3	Bradyrhizobium elkanii	0.647	4.73E-09
799	CDC34	Escherichia coli	-0.366	2.08E-04	2183	IFT88	Actinomyces naeslundii	0.093	5.66E-11	3567	POLR2H	Actinomyces naeslundii	-0.463	1.89E-62	4951	TRAPPC3	Cutibacterium modestum	-0.712	1.68E-03
800	CDC37L1	Thermaceae	-0.700	8.03E-03	2184	IGDCC4	Pseudomonas oleovorans	-0.162	2.33E-03	3568	POLR2K	Bradyrhizobium elkanii	0.538	4.28E-04	4952	TRAT1	Micromonosporales	0.321	3.71E-03
801	CDC40	Actinomyces naeslundii	0.368	4.46E-07	2185	IGF2	Thermaceae	0.573	9.59E-03	3569	POLR2M	Acinetobacter ursingii	0.770	1.54E-05	4953	TRDMT1	Pseudomonas oleovorans	-0.257	3.19E-03
802	CDC42BPB	Bradyrhizobium elkanii	-0.599	4.54E-04	2186	IGFALS	Dolosigranulum pigrum	-0.393	1.92E-05	3570	POLR3G	Veillonella	-0.616	6.70E-03	4954	TRDN	Pseudomonas oleovorans	-0.398	8.56E-04
803	CDC42BPB	Cutibacterium modestum	0.730	4.94E-17	2187	IGFALS	Veillonella	0.428	2.42E-03	3571	POLR3GL	Acinetobacter ursingii	-0.713	4.98E-08	4955	TRIB1	Leptotrichiaceae	0.204	5.28E-03
804	CDC42EP1	Leotiomycetes	-0.580	6.14E-03	2188	IGFBP2	Kytococcaceae	0.235	3.85E-04	3572	POM121	Bradyrhizobium elkanii	-0.712	7.03E-05	4956	TRIM14	Veillonella parvula	0.510	8.28E-03
805	CDC42EP4	Actinomyces naeslundii	0.186	3.73E-05	2189	IGFBP3	Escherichia coli	0.487	1.28E-31	3573	POMK	Acinetobacter ursingii	0.744	4.96E-16	4957	TRIM15	Kytococcaceae	0.084	5.33E-03
806	CDC42EP4	Ceratobasidiaceae	-0.468	1.18E-04	2190	IGFBP7	Escherichia coli	0.407	3.00E-07	3574	POMK	Phyllobacteriaceae	-0.692	6.56E-03	4958	TRIM16	Ustilaginaceae	0.079	6.25E-04
807	CDC42EP4	Ustilaginaceae	-0.314	3.03E-08	2191	IGFL2	Kytococcaceae	0.157	1.86E-04	3575	POMP	Paucibacter	0.292	2.66E-08	4959	TRIM21	Kytococcaceae	-0.100	1.82E-03

808	CDC42SE2	Escherichia coli	0.509	4.16E-07	2192	IGFL2	Pseudomonas oleovorans	-0.171	2.86E-08	3576	POMT1	Escherichia coli	-0.225	1.66E-09	4960	TRIM23	Escherichia coli	0.340	4.33E-05
809	CDC45	Escherichia coli	-0.515	3.61E-03	2193	IGFLR1	Kytococcaceae	0.042	5.57E-05	3577	POMT1	Paucibacter	-0.368	5.49E-05	4961	TRIM28	Escherichia coli	-0.269	3.93E-11
810	CDC6	Leptotrichiaceae	-0.262	5.57E-03	2194	IGFN1	Kytococcaceae	0.285	2.91E-03	3578	POMT2	Escherichia coli	-0.451	1.53E-03	4962	TRIM41	Escherichia coli	-0.343	9.17E-03
811	CDC7	Dolosigranulum pigrum	0.464	5.19E-09	2195	IGFN1	Pseudomonas oleovorans	-0.302	3.80E-05	3579	POMT2	Ustilaginaceae	0.147	1.98E-03	4963	TRIM45	Ustilaginaceae	-0.113	6.11E-07
812	CDC73	Escherichia coli	0.464	1.30E-05	2196	IGHMBP2	Actinomyces naeslundii	-0.259	1.04E-11	3580	POMZP3	Actinomyces naeslundii	-0.228	2.25E-03	4964	TRIM52	Actinomyces naeslundii	-0.242	1.73E-10
813	CDCA3	Pseudomonas oleovorans	-0.283	1.33E-05	2197	IGSF5	Pseudomonas oleovorans	-0.180	1.90E-05	3581	POMZP3	Ustilaginaceae	0.294	1.49E-04	4965	TRIM52	Ustilaginaceae	0.274	2.35E-04
814	CDCA7	Dolosigranulum pigrum	0.274	1.40E-03	2198	IGSF9B	Kytococcaceae	0.107	9.25E-05	3582	POP4	Achromobacter	0.559	2.13E-04	4966	TRIM56	Actinomyces naeslundii	-0.169	1.48E-03
815	CDH16	Kytococcaceae	0.234	3.95E-04	2199	IGSF9B	Pseudomonas oleovorans	-0.363	1.11E-05	3583	POPDC2	Actinomyces naeslundii	-0.407	1.78E-07	4967	TRIM56	Escherichia coli	-0.494	4.27E-03
816	CDH23	Bradyrhizobium elkanii	-0.533	1.44E-07	2200	IKBKB	Bradyrhizobium elkanii	-0.606	1.66E-04	3584	POR	Escherichia coli	-0.454	2.54E-04	4968	TRIM59	Dolosigranulum pigrum	0.372	7.22E-03
817	CDH23	Escherichia coli	-0.284	4.80E-04	2201	IKBKG	Escherichia coli	-0.362	5.65E-26	3585	POT1	Escherichia coli	0.344	6.04E-05	4969	TRIM67	Pseudomonas oleovorans	-0.286	6.13E-06
818	CDH26	Kytococcaceae	0.149	2.67E-06	2202	IKZF1	Pseudomonadaceae	0.572	1.29E-03	3586	POU5F1	Actinomyces naeslundii	-0.338	4.42E-21	4970	TRIM73	Actinomyces naeslundii	-0.353	2.43E-04
819	CDH3	Pseudomonas oleovorans	-0.463	1.33E-06	2203	IKZF5	Escherichia coli	0.383	5.24E-06	3587	POU6F1	Actinomyces naeslundii	-0.375	1.32E-27	4971	TRIM73	Ustilaginaceae	0.072	5.04E-13
820	CDH3	Streptomyces sp. T12	0.364	7.48E-04	2204	IL11RA	Actinomyces naeslundii	-0.333	9.97E-09	3588	PPA2	Bradyrhizobium elkanii	0.572	8.76E-09	4972	TRIM74	Actinomyces naeslundii	-0.264	1.21E-18
821	CDH5	Paucibacter	-0.283	1.54E-08	2205	IL13RA2	Paucibacter	-0.433	9.00E-04	3589	PPARGC1B	Moraxella	-0.566	6.97E-05	4973	TRIP10	Bradyrhizobium elkanii	-0.620	4.80E-06
822	CDH7	Kytococcaceae	0.264	7.32E-03	2206	IL13RA2	Ustilaginaceae	-0.176	4.38E-09	3590	PPFIA1	Sphingobacterium	0.613	2.67E-03	4974	TRIQK	Escherichia coli	0.341	6.49E-08
823	CDH7	Pseudomonas oleovorans	-0.260	4.19E-25	2207	IL15RA	Kytococcaceae	0.170	6.04E-31	3591	PPFIA3	Escherichia coli	-0.627	7.75E-06	4975	TRIT1	Actinomyces naeslundii	-0.329	2.04E-05
824	CDH8	Kytococcaceae	0.176	4.73E-09	2208	IL17REL	Pseudomonas oleovorans	-0.239	1.29E-15	3592	PPFIBP1	Micromonospora	0.711	1.65E-03	4976	TRMO	Ustilaginaceae	0.299	4.68E-06
825	CDH8	Pseudomonas oleovorans	-0.299	5.60E-08	2209	IL18RAP	Actinomyces naeslundii	-0.369	3.41E-05	3593	PPIB	Actinomyces naeslundii	0.194	7.60E-06	4977	TRMT1	Actinomyces naeslundii	-0.306	1.83E-05
826	CDHR5	Escherichia coli	-0.347	7.94E-06	2210	IL1R1	Escherichia coli	0.458	3.19E-04	3594	PPIB	Ustilaginaceae	-0.138	7.92E-03	4978	TRMT1	Ustilaginaceae	-0.090	1.18E-04
827	CDIN1	Dolosigranulum pigrum	0.420	7.36E-07	2211	IL1RL2	Escherichia coli	-0.458	4.34E-06	3595	PPIH	Acinetobacter ursingii	-0.738	5.38E-09	4979	TRMT10A	Mycosphaerellales	0.434	5.37E-04
828	CDK10	Actinomyces naeslundii	-0.323	7.18E-28	2212	IL1RN	Methylorubrum	0.241	7.99E-04	3596	PPIL6	Pseudomonas oleovorans	-0.454	1.41E-03	4980	TRMT10C	Cutibacterium modestum	-0.633	2.32E-07
829	CDK11A	Actinomyces naeslundii	-0.236	4.61E-107	2213	IL3RA	Kytococcaceae	0.002	1.43E-03	3597	PPIP5K1	Ustilaginaceae	0.247	1.27E-08	4981	TRMT11	Thermaceae	-0.687	4.05E-06
830	CDK11A	Kytococcaceae	0.179	2.16E-28	2214	IL5RA	Pseudomonas oleovorans	-0.186	5.77E-06	3598	PPM1F	Paucibacter	-0.350	1.58E-10	4982	TRMT13	Ustilaginaceae	0.250	4.52E-04
831	CDK11B	Escherichia coli	-0.514	2.25E-17	2215	IL6ST	Escherichia coli	0.553	5.91E-10	3599	PPM1H	Actinomyces naeslundii	0.258	7.78E-04	4983	TRMT1L	Escherichia coli	0.346	1.52E-05
832	CDK11B	Kytococcaceae	0.346	7.55E-20	2216	ILKAP	Actinomyces naeslundii	-0.276	2.66E-51	3600	PPOX	Actinomyces naeslundii	-0.345	1.12E-09	4984	TRMT44	Escherichia coli	-0.386	5.94E-03
833	CDK17	Escherichia coli	0.541	8.80E-08	2217	ILRUN	Actinomyces naeslundii	0.350	3.17E-05	3601	PPP1CA	Actinomyces naeslundii	0.261	9.86E-05	4985	TRMT61A	Deinococcota	0.378	6.21E-06
834	CDK2AP1	Escherichia coli	0.425	1.81E-08	2218	IMMP2L	Pseudomonas oleovorans	-0.380	3.20E-07	3602	PPP1CA	Dolosigranulum pigrum	-0.408	5.64E-03	4986	TRMT61A	Escherichia coli	-0.417	6.00E-20
835	CDK5RAP1	Halomonadaceae	-0.254	3.41E-04	2219	IMPA1	Actinomyces naeslundii	-0.299	4.98E-05	3603	PPP1CB	Escherichia coli	0.296	9.44E-07	4987	TRMU	Escherichia coli	-0.567	2.52E-16
836	CDK5RAP1	Ustilaginaceae	0.242	5.78E-03	2220	IMPG2	Kytococcaceae	0.163	6.31E-04	3604	PPP1CB	Rhizobiaceae	-0.274	1.27E-03	4988	TRNT1	Ustilaginaceae	0.278	3.92E-05
837	CDK5RAP3	Actinomyces naeslundii	-0.441	3.16E-35	2221	IMPG2	Pseudomonas oleovorans	-0.195	2.26E-06	3605	PPP1R10	Actinomyces naeslundii	-0.226	2.55E-45	4989	TRO	Actinomyces naeslundii	-0.282	4.33E-12
838	CDKL2	Pseudomonas oleovorans	-0.528	5.41E-24	2222	INAVA	Escherichia coli	-0.306	6.30E-35	3606	PPP1R12A	Thermaceae	-0.712	3.35E-06	4990	TRPA1	Pseudomonas oleovorans	-0.333	2.88E-06
839	CDKL3	Pseudomonas oleovorans	-0.450	9.65E-03	2223	INF2	Bradyrhizobium elkanii	-0.498	2.06E-06	3607	PPP1R13B	Bradyrhizobium elkanii	-0.643	2.30E-13	4991	TRPC4	Pseudomonas oleovorans	-0.307	1.17E-04
840	CDKL4	Pseudomonas oleovorans	-0.119	8.98E-05	2224	INF2	Escherichia coli	-0.272	2.21E-27	3608	PPP1R15A	Actinomyces naeslundii	-0.320	5.44E-09	4992	TRPC6	Pseudomonas oleovorans	-0.372	1.18E-08
841	CDKN1A	Kytococcus	0.457	1.03E-03	2225	ING1	Escherichia coli	0.454	2.17E-03	3609	PPP1R18	Cutibacterium acnes	0.418	1.33E-03	4993	TRPM1	Pseudomonas oleovorans	-0.337	5.53E-07
842	CDKN1B	Escherichia coli	0.324	5.00E-55	2226	ING5	Actinomyces naeslundii	-0.462	1.07E-20	3610	PPP1R1C	Escherichia coli	0.287	1.38E-03	4994	TRPM2	Pseudomonas oleovorans	-0.183	1.91E-03
843	CDR2L	Mycosphaerellales	-0.533	6.75E-03	2227	INHBC	Actinomyces naeslundii	0.369	2.77E-05	3611	PPP1R1C	Schlegelella aquatica	-0.423	4.75E-05	4995	TRPM3	Kytococcaceae	0.126	9.05E-03
844	CEACAM1	Actinomyces naeslundii	0.369	9.06E-08	2228	INHBC	Kytococcaceae	-0.028	6.04E-05	3612	PPP1R2	Escherichia coli	0.422	8.61E-10	4996	TRPT1	Actinomyces naeslundii	-0.491	7.95E-10
845	CEACAM19	Actinomyces naeslundii	-0.151	5.77E-10	2229	INHBE	Actinomyces naeslundii	0.483	1.04E-07	3613	PPP1R21	Ustilaginaceae	0.163	8.51E-03	4997	TRPV1	Actinomyces naeslundii	-0.186	6.48E-14
846	CEACAM20	Cutibacterium granulosum	-0.221	6.77E-03	2230	INKA2	Debaryomycetaceae	-0.504	5.43E-04	3614	PPP1R27	Halomonadaceae	-0.187	6.71E-23	4998	TRPV6	Kytococcaceae	0.066	3.01E-09
847	CEACAM20	Dolosigranulum pigrum	0.371	1.60E-05	2231	INO80	Oxalobacteraceae	0.533	8.73E-04	3615	PPP1R35	Actinomyces naeslundii	-0.401	1.13E-25	4999	TRRAP	Acinetobacter ursingii	0.719	8.53E-06
848	CEACAM20	Pseudomonas oleovorans	-0.366	4.96E-03	2232	INO80E	Comamonadaceae	0.437	4.32E-04	3616	PPP1R35	Enterobacterales	-0.563	3.48E-03	5000	TRRAP	Cutibacterium modestum	0.699	2.19E-03
849	CEACAM21	Paucibacter	-0.321	1.27E-04	2233	INO80E	Escherichia coli	-0.258	2.11E-04	3617	PPP1R37	Escherichia coli	-0.329	3.63E-14	5001	TRUB1	Escherichia coli	0.428	5.66E-06
850	CEBPA	Dolosigranulum pigrum	-0.366	4.62E-04	2234	INO80E	Ustilaginaceae	-0.105	1.49E-08	3618	PPP1R3E	Actinomyces naeslundii	-0.226	7.30E-10	5002	TSC2	Escherichia coli	-0.125	1.04E-05
851	CELF2	Glomerellaceae	0.403	2.89E-05	2235	INPP1	Actinomyces naeslundii	-0.257	1.78E-40	3619	PPP1R3G	Streptococcus oralis	-0.550	4.29E-07	5003	TSC22D2	Rhizobiaceae	-0.527	1.13E-04
852	CELF4	Kytococcaceae	0.129	3.26E-07	2236	INPP5B	Actinomyces naeslundii	-0.171	2.83E-43	3620	PPP1R9B	Paucibacter	-0.434	7.59E-03	5004	TSC22D3	Leptotrichiaceae	0.218	1.09E-03
853	CELSR3	Kytococcaceae	0.143	1.00E-14	2237	INPP5D	Cutibacterium acnes	0.536	2.33E-04	3621	PPP2R1B	Actinomyces naeslundii	-0.285	1.70E-05	5005	TSG101	Bradyrhizobium elkanii	0.667	1.74E-11
854	CEMIP	Pseudomonas oleovorans	-0.249	1.57E-07	2238	INPP5E	Actinomyces naeslundii	-0.342	1.10E-52	3622	PPP2R2B	Pseudomonas oleovorans	-0.323	1.03E-03	5006	TSHR	Actinomyces naeslundii	-0.291	9.31E-05
855	CENATAC	Actinomyces naeslundii	-0.323	9.52E-32	2239	INPP5J	Acinetobacter baumannii	0.263	3.15E-03	3623	PPP2R2C	Paenibacillaceae	0.167	1.78E-03	5007	TSHZ1	Escherichia coli	0.510	8.03E-03
856	CENPE	Pseudomonas oleovorans	-0.294	1.22E-04	2240	INPP5J	Kytococcaceae	0.315	1.37E-07	3624	PPP2R2C	Pseudomonas oleovorans	-0.263	7.50E-07	5008	TSLP	Escherichia coli	0.246	1.32E-04
857	CENPK	Pseudomonas oleovorans	-0.490	3.36E-07	2241	INSC	Pseudomonas oleovorans	-0.405	1.17E-08	3625	PPP2R2D	Staphylococcus epidermidis	-0.436	4.12E-20	5009	TSNARE1	Bradyrhizobium elkanii	-0.551	1.18E-05
858	CENPP	Paucibacter	-0.318	3.79E-03	2242	INSIG2	Cutibacterium modestum	-0.605	6.50E-07	3626	PPP2R5A	Staphylococcus epidermidis	0.460	8.58E-04	5010	TSPAN11	Paucibacter	-0.392	2.90E-03
859	CENPT	Actinomyces naeslundii	-0.286	3.80E-53	2243	INSYN2A	Pseudomonas oleovorans	-0.454	5.16E-06	3627	PPP3CA	Escherichia coli	0.274	5.65E-03	5011	TSPAN12	Escherichia coli	0.329	2.51E-03
860	CENPV	Actinomyces naeslundii	-0.385	2.85E-79	2244	INTS1	Escherichia coli	-0.275	1.87E-13	3628	PPP4C	Enterobacterales	-0.456	9.04E-03	5012	TSPAN15	Veillonella	0.465	1.22E-03
861	CENPV	Moraxella	0.480	3.10E-85	2245	INTS11	Escherichia coli	-0.211	1.06E-06	3629	PPP4C	Nocardioides	0.395	4.61E-05	5013	TSPAN7	Ustilaginaceae	-0.222	7.78E-04

862	CENPW	Kytococcaceae	-0.104	5.13E-07	2246	INTS15	Escherichia coli	-0.367	7.62E-04	3630	PPP4R3A	Actinomyces naeslundii	-0.111	5.39E-12	5014	TSPAN9	Escherichia coli	-0.470	6.60E-16
863	CENPX	Enterobacterales	-0.523	8.26E-03	2247	INTS5	Actinomyces naeslundii	0.352	2.84E-06	3631	PPP4R4	Artverviricota	0.310	3.95E-03	5015	TSPOAP1	Actinomyces naeslundii	-0.305	3.41E-05
864	CEP104	Actinomyces naeslundii	0.317	9.17E-03	2248	INTS6	Ustilaginaceae	0.372	5.65E-03	3632	PPP6C	Escherichia coli	0.524	3.03E-09	5016	TSPOAP1	Ustilaginaceae	-0.146	1.62E-03
865	CEP128	Dolosigranulum pigrum	0.256	2.64E-04	2249	INTS6L	Actinomyces naeslundii	-0.435	6.13E-18	3633	PPP6R1	Escherichia coli	-0.186	1.35E-15	5017	TSPYL1	Rhizobiaceae	-0.354	1.47E-23
866	CEP162	Ustilaginaceae	0.224	2.39E-04	2250	IP6K2	Actinomyces naeslundii	-0.251	5.17E-14	3634	PPRC1	Kytococcaceae	0.223	1.58E-06	5018	TSPYL2	Actinomyces naeslundii	-0.478	9.65E-47
867	CEP164	Actinomyces naeslundii	-0.193	4.71E-16	2251	IPMK	Escherichia coli	0.398	1.79E-04	3635	PPT1	Actinomyces naeslundii	0.192	9.45E-26	5019	TSPYL4	Kytococcaceae	-0.273	5.55E-05
868	CEP170B	Escherichia coli	-0.210	4.60E-06	2252	IPO11	Actinomyces naeslundii	0.543	1.15E-14	3636	PPWD1	Ustilaginaceae	0.253	5.50E-05	5020	TSR1	Escherichia coli	-0.422	3.88E-04
869	CEP250	Bradyrhizobium elkanii	-0.507	2.33E-03	2253	IPO8	Actinomyces naeslundii	0.446	5.64E-07	3637	PRAG1	Bradyrhizobium elkanii	-0.663	1.24E-17	5021	TSR1	Methyloversatilis sp. RAC08	0.486	1.51E-05
870	CEP250	Escherichia coli	-0.227	3.72E-05	2254	IQCE	Escherichia coli	-0.365	7.32E-04	3638	PRAMEF33	Actinomyces naeslundii	0.173	1.89E-04	5022	TSR3	Ustilaginaceae	-0.286	8.68E-03
871	CEP295	Acidovorax temperans	0.140	9.92E-03	2255	IQCM	Pseudomonas oleovorans	-0.092	5.59E-04	3639	PRCP	Actinomyces naeslundii	0.307	6.31E-224	5023	TSSC4	Actinomyces naeslundii	-0.170	4.65E-07
872	CEP295	Sclerotiniaceae	0.538	2.38E-03	2256	IQCN	Actinomyces naeslundii	-0.257	4.03E-33	3640	PRDM11	Escherichia coli	-0.322	7.15E-03	5024	TSSC4	Escherichia coli	-0.600	1.64E-03
873	CEP295	Thermaceae	-0.603	3.17E-05	2257	IQGAP1	Thermales	0.672	1.58E-04	3641	PRDM15	Kytococcaceae	0.245	2.64E-03	5025	TSSK4	Empedobacter	-0.321	2.06E-03
874	CEP295	Ustilaginaceae	0.232	1.84E-03	2258	IQGAP2	Escherichia coli	0.279	1.16E-11	3642	PRDM6	Dolosigranulum pigrum	0.302	7.93E-03	5026	TTC1	Bradyrhizobium elkanii	0.641	7.72E-09
875	CEP295NL	Dolosigranulum pigrum	0.201	1.68E-23	2259	IQGAP3	Pseudomonas oleovorans	-0.235	2.91E-07	3643	PRDX1	Actinomyces naeslundii	0.332	3.20E-20	5027	TTC12	Escherichia coli	-0.395	3.87E-03
876	CEP78	Ceratobasidiaceae	0.603	2.38E-05	2260	IQUB	Pseudomonas oleovorans	-0.297	2.70E-09	3644	PRDX2	Actinomyces naeslundii	0.137	1.50E-22	5028	TTC13	Halomonadaceae	-0.096	3.69E-03
877	CEP95	Actinomyces naeslundii	-0.124	4.42E-33	2261	IRAK4	Ustilaginaceae	0.232	1.83E-03	3645	PRDX3	Escherichia coli	0.210	3.72E-03	5029	TTC13	Ustilaginaceae	0.273	1.46E-04
878	CERS2	Actinomyces naeslundii	0.308	5.91E-28	2262	IRF2BP2	Leptotrichiaceae	0.073	1.14E-03	3646	PREB	Enterobacterales	-0.582	3.03E-04	5030	TTC14	Ustilaginaceae	0.322	8.71E-04
879	CERS4	Dolosigranulum pigrum	-0.403	3.32E-03	2263	IRF3	Actinomyces naeslundii	-0.331	2.69E-164	3647	PREB	Plantactinospora	-0.537	4.89E-03	5031	TTC21B	Dolosigranulum pigrum	0.326	3.02E-03
880	CERS4	Eubacteriales	0.500	8.84E-03	2264	IRF5	Paucibacter	-0.138	2.87E-03	3648	PRELID1	Ustilaginaceae	-0.293	3.20E-06	5032	TTC23	Dolosigranulum pigrum	-0.304	2.37E-04
881	CERS6	Micromonosporales	0.561	7.58E-05	2265	IRF7	Enterobacterales	-0.424	1.24E-03	3649	PREX1	Deinococci	0.590	4.69E-03	5033	TTC24	Dolosigranulum pigrum	0.368	6.34E-04
882	CERT1	Gordonia bronchialis	-0.358	1.68E-13	2266	IRF7	Plantactinospora sp. BB1	-0.382	6.90E-04	3650	PRH2	Pseudomonas oleovorans	-0.228	9.07E-03	5034	TTC30A	Escherichia coli	0.361	1.80E-03
883	CES1	Dolosigranulum pigrum	-0.241	7.19E-03	2267	IRGQ	Cutibacterium modestum	0.668	4.37E-11	3651	PRICKLE4	Actinomyces naeslundii	-0.184	2.37E-19	5035	TTC31	Halomonadaceae	-0.219	2.21E-06
884	CES2	Dolosigranulum pigrum	-0.418	6.55E-05	2268	IRS1	Kytococcaceae	-0.178	5.79E-05	3652	PRIM1	Actinomyces naeslundii	0.438	1.22E-05	5036	TTC32	Dolosigranulum pigrum	0.253	3.67E-04
885	CES4A	Ustilaginaceae	0.171	1.15E-09	2269	ISCA1	Cutibacterium modestum	-0.660	7.69E-15	3653	PRIM1	Anaerococcus	-0.468	8.78E-03	5037	TTC33	Paucibacter	0.323	2.08E-03
886	CETN2	Bradyrhizobium elkanii	0.504	2.11E-03	2270	ISCA1	Escherichia coli	0.123	1.91E-04	3654	PRKAA1	Cyanophyceae	-0.550	1.38E-03	5038	TTC34	Pseudomonas oleovorans	-0.279	5.88E-04
887	CETN3	Cutibacterium modestum	-0.675	1.09E-08	2271	ISCU	Escherichia coli	0.370	5.84E-04	3655	PRKACA	Streptomyces sp. T12	-0.510	3.41E-03	5039	TTC36	Dolosigranulum pigrum	-0.323	8.80E-18
888	CETP	Paucibacter	-0.300	5.18E-03	2272	ISG20	Kytococcaceae	0.061	9.69E-06	3656	PRKACB	Bradyrhizobium elkanii	0.284	1.46E-05	5040	TTC39A	Pseudomonas oleovorans	-0.089	4.61E-04
889	CFAP157	Escherichia coli	-0.445	5.40E-13	2273	ISOC1	Cutibacterium modestum	-0.613	2.38E-08	3657	PRKACB	Escherichia coli	0.478	1.13E-09	5041	TTC6	Actinomyces naeslundii	-0.217	1.29E-04
890	CFAP157	Moraxellaceae	-0.558	1.31E-04	2274	ISOC2	Paucibacter	-0.508	3.27E-08	3658	PRKAR1A	Escherichia coli	0.679	3.69E-21	5042	TTLL12	Ceratobasidiaceae	-0.341	3.03E-03
891	CFAP251	Actinomyces naeslundii	-0.245	5.52E-21	2275	ISYNA1	Bradyrhizobium elkanii	-0.660	2.92E-08	3659	PRKAR1A	Plantactinospora	0.653	2.48E-04	5043	TTLL13	Pseudomonas oleovorans	-0.224	5.71E-04
892	CFAP298	Actinomyces naeslundii	-0.291	4.11E-20	2276	ITFG1	Actinomyces naeslundii	0.432	1.34E-36	3660	PRKAR1A	Staphylococcus epidermidis	0.347	3.08E-03	5044	TTLL2	Actinomyces naeslundii	-0.381	3.87E-07
893	CFAP299	Pseudomonas oleovorans	-0.264	1.89E-16	2277	ITFG2	Actinomyces naeslundii	-0.427	1.08E-17	3661	PRKAR1B	Rhizobiaceae	0.501	9.20E-05	5045	TTLL4	Kytococcaceae	0.297	1.54E-04
894	CFAP410	Escherichia coli	-0.400	3.76E-06	2278	ITGA10	Actinomyces naeslundii	-0.335	1.25E-09	3662	PRKAR2B	Escherichia coli	0.538	3.34E-10	5046	TTLL5	Yersiniaceae	-0.519	1.09E-03
895	CFAP43	Kytococcaceae	0.014	1.80E-07	2279	ITGA11	Kytococcaceae	0.142	1.86E-03	3663	PRKCZ	Alphaproteobacteria	0.582	9.15E-07	5047	TTLL7	Dolosigranulum pigrum	0.463	1.49E-04
896	CFAP44	Actinomyces naeslundii	-0.129	7.18E-04	2280	ITGA4	Escherichia coli	0.506	4.40E-03	3664	PRKD2	Escherichia coli	-0.293	1.95E-03	5048	TTPA	Kytococcaceae	-0.074	2.34E-10
897	CFAP46	Escherichia coli	-0.250	4.14E-03	2281	ITGA5	Paucibacter	-0.577	8.95E-05	3665	PRKD3	Escherichia coli	0.431	1.59E-04	5049	TUBA1B	Actinomyces naeslundii	0.198	1.04E-04
898	CFAP52	Pseudomonas oleovorans	-0.238	2.77E-05	2282	ITGAD	Rhizobiaceae	0.294	2.76E-04	3666	PRKD3	Kytococcaceae	-0.100	5.07E-03	5050	TUBA3D	Kytococcaceae	0.091	1.69E-07
899	CFAP53	Kytococcaceae	0.168	2.65E-08	2283	ITGAE	Hyphomicrobiales	-0.616	7.01E-04	3667	PRKRA	Actinomyces naeslundii	-0.223	9.86E-05	5051	TUBB	Actinomyces naeslundii	0.379	4.89E-06
900	CFAP54	Kytococcaceae	0.246	3.89E-05	2284	ITGAV	Halomonadaceae	0.399	3.16E-07	3668	PRLR	Escherichia coli	0.517	5.30E-03	5052	TUBG2	Ustilaginaceae	0.098	5.83E-05
901	CFAP57	Actinomyces naeslundii	-0.303	4.49E-09	2285	ITGAX	Dolosigranulum pigrum	0.468	2.77E-03	3669	PRMT7	Escherichia coli	-0.307	9.81E-05	5053	TUBGCP2	Escherichia coli	-0.207	6.00E-07
902	CFAP61	Pseudomonas oleovorans	-0.153	1.54E-03	2286	ITGB1	Actinomyces naeslundii	0.260	1.13E-14	3670	PROC	Escherichia coli	-0.391	2.29E-11	5054	TUBGCP3	Acinetobacter ursingii	0.646	4.26E-08
903	CFAP61	Streptomyces sp. T12	0.181	3.44E-06	2287	ITGB7	Leotiomycetes	-0.537	6.94E-04	3671	PRODH2	Escherichia coli	-0.298	3.07E-08	5055	TVP23A	Pseudomonas oleovorans	-0.381	3.88E-05
904	CFAP65	Pseudomonas oleovorans	-0.362	6.25E-10	2288	ITIH2	Actinomyces naeslundii	0.424	5.94E-09	3672	PROS1	Actinomyces naeslundii	0.346	5.58E-18	5056	TVP23B	Paucibacter	0.325	6.32E-04
905	CFAP69	Actinomyces naeslundii	-0.254	1.75E-04	2289	ITIH4	Escherichia coli	-0.302	1.34E-05	3673	PROX1	Escherichia coli	0.447	6.39E-03	5057	TVP23C	Actinomyces naeslundii	-0.484	1.12E-07
906	CFAP99	Pseudomonas oleovorans	-0.333	1.73E-04	2290	ITM2A	Escherichia coli	0.452	3.49E-07	3674	PROZ	Yersiniaceae	-0.502	3.03E-03	5058	TVP23C	Verrucomicrobiae	0.373	6.04E-03
907	CFB	Oxalobacteraceae	-0.296	5.14E-03	2291	ITM2B	Escherichia coli	0.556	5.93E-18	3675	PRPF3	Ustilaginaceae	0.094	5.25E-14	5059	TWF1	Escherichia coli	0.357	9.82E-16
908	CFI	Oxalobacteraceae	-0.358	1.35E-05	2292	ITM2C	Actinomyces naeslundii	0.275	1.14E-07	3676	PRPF31	Escherichia coli	-0.264	6.65E-08	5060	TWF2	Dolosigranulum pigrum	-0.414	9.64E-03
909	CFL1	Actinomyces naeslundii	0.260	2.78E-10	2293	ITPA	Actinomyces naeslundii	-0.461	3.27E-06	3677	PRPF38B	Actinomyces naeslundii	-0.261	7.46E-14	5061	TWIST2	Pseudomonas oleovorans	-0.594	4.30E-06
910	CFP	Paucibacter	-0.382	6.34E-06	2294	ITPKB	Pseudomonadaceae	0.675	3.89E-05	3678	PRPF39	Actinomyces naeslundii	-0.165	1.68E-04	5062	TWSG1	Escherichia coli	0.408	7.61E-10
911	CGAS	Dolosigranulum pigrum	0.309	1.13E-05	2295	ITPKC	Kytococcaceae	0.053	7.57E-07	3679	PRPF4B	Ustilaginaceae	0.346	2.86E-05	5063	TXLNB	Rhodobacterales	-0.420	7.80E-03
912	CH25H	Lactobacillaceae	-0.423	1.83E-03	2296	ITPR2	Pseudomonas oleovorans	0.557	7.24E-04	3680	PRPF6	Escherichia coli	-0.510	1.39E-08	5064	TXN	Staphylococcaceae	0.408	3.60E-10
913	CHAF1A	Escherichia coli	-0.422	1.28E-04	2297	ITPRID1	Pseudomonas oleovorans	-0.161	5.50E-06	3681	PRR13	Ustilaginaceae	-0.157	2.90E-03	5065	TXNL4A	Ustilaginaceae	-0.424	2.73E-09
914	CHAF1B	Kytococcaceae	0.107	1.44E-09	2298	ITPRID2	Escherichia coli	0.192	4.38E-03	3682	PRR14	Fusobacterium	-0.323	1.36E-06	5066	TXNRD1	Actinomyces naeslundii	0.441	1.00E-10
915	CHAMP1	Actinomyces naeslundii	0.422	7.56E-03	2299	ITSN2	Ustilaginaceae	0.315	9.09E-03	3683	PRR14L	Acinetobacter ursingii	0.822	4.64E-14	5067	TXNRD2	Escherichia coli	-0.352	1.97E-06

916	CHCHD1	Cutibacterium modestum	-0.683	4.33E-06	2300	IVNS1ABP	Actinomyces naeslundii	-0.348	2.99E-04	3684	PRR22	Actinomyces naeslundii	-0.041	1.04E-39	5068	TXNRD3	Actinomyces naeslundii	0.341	1.57E-03
917	CHCHD2	Ustilaginaceae	-0.171	1.43E-10	2301	IWS1	Actinomyces naeslundii	-0.118	1.54E-06	3685	PRR3	Actinomyces naeslundii	-0.323	1.29E-14	5069	TYROBP	Ustilaginaceae	-0.290	1.33E-04
918	CHD1L	Ustilaginaceae	0.418	6.44E-05	2302	IWS1	Nostocales	0.394	4.11E-03	3686	PRR33	Escherichia coli	-0.563	6.33E-03	5070	TYSND1	Escherichia coli	-0.452	6.24E-09
919	CHD4	Escherichia coli	-0.295	1.76E-03	2303	IWS1	Paucibacter	0.468	4.79E-03	3687	PRR33	Plantactinospora sp. BC1	-0.605	1.50E-05	5071	TYSND1	Nocardioides	0.258	3.07E-03
920	CHD5	Pseudomonas oleovorans	-0.415	7.93E-08	2304	IYD	Bacillaceae	0.404	2.54E-04	3688	PRR7	Actinomyces naeslundii	-0.206	5.32E-09	5072	U2AF1L4	Actinomyces naeslundii	-0.205	2.23E-07
921	CHD5	Spirochaetota	0.391	2.39E-21	2305	IZUMO4	Actinomyces naeslundii	-0.233	5.67E-19	3689	PRRC2C	Thermaceae	-0.507	2.07E-08	5073	U2AF1L4	Ustilaginaceae	-0.007	2.40E-03
922	CHD9	Carnobacteriaceae	0.572	1.16E-05	2306	JAG2	Actinomyces naeslundii	-0.318	4.46E-08	3690	PRRC2C	Ustilaginaceae	0.419	8.91E-09	5074	U2AF2	Paucibacter	-0.389	7.17E-04
923	CHIC2	Escherichia coli	0.381	3.18E-05	2307	JAGN1	Cutibacterium modestum	-0.663	5.88E-10	3691	PRRG1	Escherichia coli	0.357	4.48E-03	5075	U2SURP	Actinomyces naeslundii	-0.182	8.27E-05
924	CHKA	Actinomyces naeslundii	-0.268	1.43E-27	2308	JAK2	Microbacteriaceae	-0.329	1.46E-03	3692	PRRG4	Escherichia coli	0.293	1.93E-03	5076	U2SURP	Ustilaginaceae	0.343	1.18E-04
925	CHM	Escherichia coli	0.407	1.44E-04	2309	JAKMIP3	Kytococcaceae	0.139	6.95E-08	3693	PRRT3	Pseudomonas oleovorans	-0.324	1.53E-05	5077	UACA	Actinomyces naeslundii	-0.244	1.52E-21
926	CHMP1A	Escherichia coli	-0.448	1.21E-07	2310	JAM2	Methyloversatilis sp. RAC08	-0.431	3.83E-04	3694	PRSS22	Escherichia coli	-0.257	2.20E-03	5078	UBA1	Escherichia coli	-0.440	1.02E-07
927	CHMP2B	Escherichia coli	0.231	1.87E-16	2311	JAM3	Thermales	0.606	9.93E-04	3695	PRSS50	Ustilaginaceae	-0.093	5.93E-04	5079	UBA7	Paucibacter	-0.396	3.94E-05
928	CHMP4B	Paucibacter	0.285	6.83E-14	2312	JAML	Actinomyces naeslundii	-0.257	7.94E-03	3696	PRSS51	Halomonadaceae	-0.204	4.12E-08	5080	UBA7	Ustilaginaceae	0.123	1.17E-05
929	CHMP5	Cutibacterium modestum	-0.675	1.07E-06	2313	JARID2	Methylobacterium sp. FF17	-0.599	1.04E-04	3697	PRSS8	Escherichia coli	-0.433	2.42E-05	5081	UBAC1	Escherichia coli	-0.375	3.57E-03
930	CHMP6	Escherichia coli	-0.336	2.15E-03	2314	JKAMP	Kytococcaceae	-0.284	2.35E-05	3698	PRTG	Pseudomonas oleovorans	-0.221	4.25E-05	5082	UBAP1L	Actinomyces naeslundii	-0.143	3.35E-09
931	CHPF2	Escherichia coli	-0.492	5.68E-06	2315	JOSD2	Escherichia coli	-0.448	1.26E-28	3699	PSAP	Actinomyces naeslundii	0.350	1.19E-13	5083	UBAP2	Escherichia coli	-0.448	2.60E-06
932	CHPF2	Kytococcaceae	0.150	4.14E-03	2316	JPH2	Pseudomonas oleovorans	-0.390	9.83E-06	3700	PSAP	Ustilaginaceae	-0.085	8.64E-03	5084	UBAP2L	Cutibacterium modestum	0.680	4.89E-10
933	CHRAC1	Escherichia coli	0.620	7.61E-03	2317	JRK	Ustilaginaceae	0.196	1.63E-04	3701	PSD2	Pseudomonas oleovorans	-0.532	2.46E-05	5085	UBB	Actinomyces naeslundii	0.231	2.35E-10
934	CHRD	Actinomyces naeslundii	-0.303	3.64E-15	2318	JTB	Cutibacterium modestum	-0.728	6.70E-10	3702	PSMA2	Bradyrhizobium elkanii	0.673	2.57E-06	5086	UBB	Ustilaginaceae	-0.205	7.33E-03
935	CHRDL2	Dolosigranulum pigrum	0.382	1.43E-15	2319	JUN	Leptotrichiaceae	0.166	4.88E-04	3703	PSMC1	Actinomyces naeslundii	0.466	7.16E-18	5087	UBD	Deinococcales	-0.149	1.32E-04
936	CHRNA10	Debaryomycetaceae	-0.508	6.12E-03	2320	JUNB	Methylorubrum	0.498	1.78E-04	3704	PSMC1	Cutibacterium acnes	-0.449	3.84E-06	5088	UBE2D1	Escherichia coli	0.524	7.00E-05
937	CHRNA10	Kytococcaceae	-0.010	4.57E-05	2321	JUP	Deinococcota	0.519	3.98E-05	3705	PSMC2	Actinomyces naeslundii	0.328	3.20E-10	5089	UBE2D2	Cutibacterium modestum	-0.671	1.06E-07
938	CHRNA4	Actinomyces naeslundii	-0.290	3.03E-11	2322	KANK2	Nostocales	-0.474	4.35E-03	3706	PSMD10	Escherichia coli	0.241	1.29E-05	5090	UBE2D3	Escherichia coli	0.347	6.69E-06
939	CHRNB4	Pseudomonas oleovorans	-0.282	1.16E-28	2323	KANK3	Bradyrhizobium elkanii	-0.623	7.53E-07	3707	PSMD13	Acinetobacter ursingii	-0.707	9.54E-32	5091	UBE2E3	Cutibacterium modestum	-0.751	7.29E-20
940	CHST14	Methyloversatilis sp. RAC08	-0.493	2.16E-08	2324	KANSL2	Thermaceae	-0.582	1.76E-03	3708	PSMD3	Paucibacter	-0.595	2.96E-04	5092	UBE2J2	Enterobacterales	-0.531	4.67E-03
941	CHST4	Dolosigranulum pigrum	0.165	3.22E-04	2325	KAT2A	Actinomyces naeslundii	-0.437	8.60E-21	3709	PSMD8	Actinomyces naeslundii	0.413	8.32E-06	5093	UBE2L3	Actinomyces naeslundii	0.370	7.47E-04
942	CHST6	Kytococcaceae	0.136	2.43E-04	2326	KAT8	Actinomyces naeslundii	-0.347	8.58E-07	3710	PSMD8	Paucibacter	-0.626	5.84E-03	5094	UBE2L3	Ustilaginaceae	-0.084	5.53E-03
943	CHST6	Pseudomonas oleovorans	-0.337	9.29E-08	2327	KATNB1	Escherichia coli	-0.288	1.26E-08	3711	PSME1	Lawsonellaceae	-0.561	3.74E-03	5095	UBE2N	Escherichia coli	0.169	4.02E-03
944	CHST7	Ustilaginaceae	-0.235	1.38E-04	2328	KATNB1	Leotiomycetes	-0.559	3.61E-04	3712	PSME1	Ustilaginaceae	-0.145	9.06E-06	5096	UBE2U	Pseudomonas oleovorans	-0.217	2.52E-07
945	CHTF18	Bradyrhizobium elkanii	-0.527	4.32E-03	2329	KBTBD12	Pseudomonas oleovorans	-0.392	4.79E-06	3713	PSME3IP1	Actinomyces naeslundii	-0.111	3.52E-13	5097	UBE2W	Escherichia coli	0.457	1.26E-06
946	CHTF18	Escherichia coli	-0.235	8.54E-03	2330	KCNA6	Pseudomonas oleovorans	-0.308	5.52E-05	3714	PSMG2	Cutibacterium modestum	-0.647	2.78E-29	5098	UBE3D	Pseudomonas oleovorans	-0.417	3.21E-04
947	CHTF18	Ustilaginaceae	-0.146	1.52E-09	2331	KCNAB2	Gordonia bronchialis	0.538	1.37E-03	3715	PSPN	Dolosigranulum pigrum	-0.279	5.34E-03	5099	UBE4B	Cutibacterium modestum	0.708	2.87E-03
948	CIAO2A	Bradyrhizobium elkanii	0.523	1.41E-03	2332	KCND2	Pseudomonas oleovorans	-0.192	8.18E-04	3716	PTBP3	Escherichia coli	0.639	4.90E-13	5100	UBL3	Escherichia coli	0.399	7.03E-55
949	CIAO2A	Escherichia coli	0.141	2.62E-03	2333	KCNE4	Leptotrichiaceae	0.165	1.68E-03	3717	PTCH2	Staphylococcus epidermidis	-0.510	7.37E-03	5101	UBL4A	Actinomyces naeslundii	0.205	2.07E-04
950	CIAO3	Escherichia coli	-0.427	2.03E-11	2334	KCNG2	Kytococcaceae	0.231	1.57E-08	3718	PTCHD4	Pseudomonas oleovorans	-0.257	2.94E-03	5102	UBL7	Actinomyces naeslundii	0.282	3.66E-06
951	CIB1	Acinetobacter ursingii	-0.663	1.94E-03	2335	KCNH1	Kytococcaceae	0.192	1.27E-03	3719	PTDSS1	Actinomyces naeslundii	0.401	2.40E-12	5103	UBL7	Dolosigranulum pigrum	-0.426	3.94E-03
952	CIBAR1	Ustilaginaceae	0.232	1.57E-03	2336	KCNH1	Pseudomonas oleovorans	-0.223	1.97E-07	3720	PTDSS2	Escherichia coli	-0.405	6.69E-33	5104	UBQLN3	Pseudomonas oleovorans	-0.071	5.71E-07
953	CIC	Bradyrhizobium elkanii	-0.659	1.43E-05	2337	KCNH2	Kytococcaceae	0.176	8.98E-04	3721	PTEN	Escherichia coli	0.374	4.96E-13	5105	UBQLN3	Verrucomicrobiae	0.449	2.42E-03
954	CIDEB	Paucibacter	-0.578	3.15E-05	2338	KCNH3	Kytococcaceae	0.194	1.06E-12	3722	PTEN	Gordonia bronchialis	-0.402	2.81E-03	5106	UBQLNL	Actinomyces naeslundii	-0.167	1.56E-03
955	CILK1	Escherichia coli	0.514	6.22E-09	2339	KCNH5	Acinetobacter baumannii	0.333	7.09E-03	3723	PTGDR	Pseudomonas oleovorans	-0.215	7.05E-03	5107	UBR1	Actinomyces naeslundii	0.557	2.31E-09
956	CIR1	Thermaceae	-0.753	5.16E-03	2340	KCNH5	Paucibacter	0.577	4.20E-04	3724	PTGER1	Dolosigranulum pigrum	-0.315	1.10E-09	5108	UBR3	Escherichia coli	0.521	8.53E-06
957	CIRBP	Actinomyces naeslundii	-0.483	1.34E-22	2341	KCNH5	Pseudomonas oleovorans	-0.348	1.97E-11	3725	PTGES3	Paucibacter	0.332	5.24E-04	5109	UBXN11	Ustilaginaceae	-0.138	1.53E-128
958	CISD3	Ustilaginaceae	-0.403	6.55E-04	2342	KCNH5	Streptomyces sp. T12	0.321	1.51E-04	3726	PTGFRN	Actinomyces naeslundii	0.410	2.16E-10	5110	UBXN4	Leotiomycetes	0.562	7.24E-06
959	CIT	Kytococcaceae	0.283	9.13E-03	2343	KCNH8	Dolosigranulum pigrum	0.192	3.40E-11	3727	PTGIS	Rhodocyclales	0.085	5.93E-03	5111	UBXN6	Thermaceae	0.749	1.41E-03
960	CITED2	Caldimonas	0.563	7.11E-03	2344	KCNH8	Rothia	0.397	8.65E-03	3728	PTK2	Pseudomonas oleovorans	0.249	1.08E-05	5112	UBXN8	Achromobacter	0.608	8.36E-04
961	CLASRP	Actinomyces naeslundii	-0.330	8.16E-54	2345	KCNIP3	Escherichia coli	-0.309	8.30E-09	3729	PTN	Escherichia coli	0.525	8.20E-03	5113	UCHL5	Escherichia coli	0.277	9.23E-17
962	CLCF1	Actinomyces naeslundii	-0.177	7.96E-39	2346	KCNJ11	Kytococcaceae	0.241	3.19E-03	3730	PTOV1	Actinomyces naeslundii	-0.417	7.38E-07	5114	UCK1	Dolosigranulum pigrum	-0.513	2.17E-06
963	CLCN1	Pseudomonas oleovorans	-0.284	2.23E-18	2347	KCNJ13	Thermaceae	-0.678	8.29E-04	3731	PTPN11	Actinomyces naeslundii	0.458	4.94E-54	5115	UCN	Microbacteriaceae	0.400	1.42E-03
964	CLCN3	Actinomyces naeslundii	0.432	3.90E-04	2348	KCNJ14	Saccharomycetaceae	-0.306	1.34E-06	3732	PTPN2	Dolosigranulum pigrum	0.492	1.20E-07	5116	UCP2	Ustilaginaceae	-0.219	1.02E-03
965	CLCN7	Bradyrhizobium elkanii	-0.452	4.27E-05	2349	KCNJ3	Lactobacillales	0.401	8.79E-06	3733	PTPN20	Pseudomonas oleovorans	-0.281	1.62E-03	5117	UCP3	Clavicipitaceae	-0.449	7.60E-03
966	CLCN7	Escherichia coli	-0.326	1.11E-09	2350	KCNJ3	Ottowia	-0.516	1.63E-07	3734	PTPN6	Cutibacterium acnes	0.375	7.11E-04	5118	UFL1	Escherichia coli	0.517	6.54E-08
967	CLDN15	Bradyrhizobium elkanii	-0.512	6.94E-05	2351	KCNJ4	Escherichia coli	-0.412	3.82E-10	3735	PTPRG	Escherichia coli	0.474	1.90E-04	5119	UFM1	Escherichia coli	0.346	7.81E-08
968	CLDN16	Caldimonas	0.356	3.95E-03	2352	KCNJ6	Kytococcaceae	0.217	1.45E-06	3736	PTPRJ	Acinetobacter ursingii	0.745	1.12E-12	5120	UFSP2	Actinomyces naeslundii	0.371	1.20E-07
969	CLDN20	Kytococcaceae	-0.367	6.10E-07	2353	KCNJ6	Pseudomonas oleovorans	-0.325	1.77E-14	3737	PTPRQ	Pseudomonas oleovorans	-0.364	1.33E-06	5121	UGCG	Escherichia coli	0.525	5.38E-04

970	CLDN3	Dolosigranulum pigrum	-0.485	3.34E-04	2354	KCNK5	Ceratobasidiaceae	-0.422	2.01E-06	3738	PTPRQ	Streptomyces sp. T12	0.322	7.91E-04	5122	UGDH	Actinomyces naeslundii	0.390	1.06E-07
971	CLDN7	Enterobacteriaceae	-0.660	7.78E-04	2355	KCNMB1	Pseudomonas oleovorans	-0.280	7.01E-04	3739	PTPRR	Pseudomonas oleovorans	-0.334	6.73E-07	5123	UGGT1	Actinomyces naeslundii	0.446	7.06E-04
972	CLDND2	Actinomyces naeslundii	-0.431	1.03E-08	2356	KCNMB3	Thermaceae	-0.587	1.21E-03	3740	PTPRZ1	Pseudomonas oleovorans	-0.306	2.28E-05	5124	UGP2	Actinomyces naeslundii	0.397	1.32E-05
973	CLEC11A	Escherichia coli	-0.312	4.11E-03	2357	KCNQ2	Kytococcaceae	0.269	5.34E-17	3741	PTRH1	Staphylococcus saprophyticus	-0.020	3.11E-03	5125	UGT1A1	Actinomyces naeslundii	0.507	3.58E-09
974	CLEC16A	Escherichia coli	-0.210	4.08E-03	2358	KCNQ2	Pseudomonas oleovorans	-0.344	1.50E-22	3742	PTTG1IP2	Dolosigranulum pigrum	0.228	1.20E-03	5126	UGT1A3	Thermaceae	0.510	1.38E-03
975	CLEC1B	Paucibacter	-0.484	8.56E-15	2359	KCNQ3	Kytococcaceae	0.203	1.51E-09	3743	PUF60	Escherichia coli	-0.297	2.70E-05	5127	UGT1A4	Actinomyces naeslundii	0.347	1.36E-09
976	CLEC2D	Actinomyces naeslundii	-0.300	6.53E-03	2360	KCNQ3	Pseudomonas oleovorans	-0.457	5.75E-13	3744	PUM2	Escherichia coli	0.426	1.53E-05	5128	UGT1A6	Actinomyces naeslundii	0.544	1.47E-11
977	CLEC4A	Actinomyces naeslundii	-0.294	7.60E-06	2361	KCNT1	Kytococcaceae	0.071	8.91E-06	3745	PURB	Actinomyces naeslundii	-0.212	7.59E-03	5129	UGT1A9	Actinomyces naeslundii	0.529	1.72E-04
978	CLEC4F	Dolosigranulum pigrum	0.215	8.14E-04	2362	KCNU1	Kytococcaceae	0.198	6.00E-11	3746	PURB	Dolosigranulum pigrum	0.564	1.36E-04	5130	UGT2B10	Dolosigranulum pigrum	-0.297	2.04E-05
979	CLEC4G	Paucibacter	-0.328	7.71E-18	2363	KCTD11	Cyanophyceae	0.543	8.51E-05	3747	PUS1	Plantactinospora	-0.441	1.11E-10	5131	UGT2B15	Kytococcaceae	-0.213	1.24E-04
980	CLIC2	Escherichia coli	0.623	1.13E-06	2364	KCTD12	Escherichia coli	0.554	4.23E-20	3748	PUSL1	Escherichia coli	-0.410	5.01E-06	5132	UGT3A1	Oxalobacteraceae	-0.278	9.46E-03
981	CLIC4	Actinomyces naeslundii	0.269	2.47E-12	2365	KCTD13	Dolosigranulum pigrum	0.201	2.41E-04	3749	PVR	Escherichia coli	-0.454	5.98E-04	5133	UHMK1	Escherichia coli	0.304	5.56E-04
982	CLIC4	Plantactinospora	0.487	4.88E-03	2366	KCTD5	Ustilaginaceae	-0.273	8.17E-03	3750	PVRIG	Achromobacter	-0.669	1.76E-05	5134	UHRF2	Actinomyces naeslundii	-0.283	4.20E-05
983	CLK1	Actinomyces naeslundii	-0.223	8.60E-07	2367	KDM3A	Actinomyces naeslundii	-0.200	1.07E-03	3751	PWWP2A	Dolosigranulum pigrum	0.441	2.53E-03	5135	ULK3	Actinomyces naeslundii	-0.195	1.50E-113
984	CLK2	Actinomyces naeslundii	-0.113	1.95E-15	2368	KDM3A	Schlegelella aquatica	0.367	5.33E-04	3752	PWWP3A	Escherichia coli	-0.397	3.21E-06	5136	UMODL1	Pseudomonas oleovorans	-0.246	1.71E-13
985	CLK3	Ustilaginaceae	-0.066	3.02E-04	2369	KDM4C	Ustilaginaceae	0.216	1.17E-07	3753	PWWP3B	Dolosigranulum pigrum	0.420	1.13E-03	5137	UNC13A	Kytococcaceae	0.235	8.60E-06
986	CLK4	Actinomyces naeslundii	-0.281	3.82E-45	2370	KDM5B	Micromonospora	0.572	5.52E-03	3754	PXMP2	Actinomyces naeslundii	0.048	1.88E-06	5138	UNC13A	Pseudomonas oleovorans	-0.333	2.04E-14
987	CLMN	Chromatiaceae	-0.240	4.56E-03	2371	KDM5C	Paucibacter	-0.417	8.12E-05	3755	PXN	Actinomyces naeslundii	-0.593	4.05E-35	5139	UNC13C	Pseudomonas oleovorans	-0.391	2.32E-08
988	CLNK	Pseudomonas oleovorans	-0.402	5.13E-07	2372	KDM5D	Debaryomycetaceae	0.662	2.90E-09	3756	PYCR2	Paucibacter	-0.327	7.20E-05	5140	UNC45B	Pseudomonas oleovorans	-0.064	1.86E-03
989	CLPP	Ustilaginaceae	-0.437	2.29E-05	2373	KDM5D	Rhodoferax	0.188	8.23E-09	3757	PYCR3	Escherichia coli	-0.513	1.83E-05	5141	UNC5D	Pseudomonas oleovorans	-0.510	3.15E-11
990	CLPTM1	Actinomyces naeslundii	0.439	2.02E-04	2374	KDM6A	Paucibacter	-0.264	2.55E-03	3758	PYM1	Cutibacterium modestum	-0.600	1.96E-29	5142	UNC93B1	Cutibacterium modestum	0.633	1.67E-11
991	CLPTM1	Hyphomicrobiales	0.521	9.44E-04	2375	KDM6B	Bradyrhizobium elkanii	-0.699	1.94E-05	3759	QKI	Actinomyces naeslundii	0.052	7.21E-07	5143	UNC93B1	Paucibacter	-0.288	1.76E-03
992	CLTA	Ustilaginaceae	-0.218	4.30E-21	2376	KDM7A	Schlegelella aquatica	0.373	1.15E-03	3760	QKI	Escherichia coli	0.627	7.17E-10	5144	UNK	Ottowia	0.402	1.02E-03
993	CLTB	Escherichia coli	-0.362	1.21E-04	2377	KDR	Paucibacter	-0.366	9.66E-05	3761	QPRT	Dolosigranulum pigrum	-0.375	1.75E-03	5145	UPF3A	Actinomyces naeslundii	-0.311	5.25E-24
994	CLTC	Actinomyces naeslundii	0.571	3.29E-22	2378	KDSR	Escherichia coli	0.251	5.15E-05	3762	QRICH2	Actinomyces naeslundii	-0.183	5.24E-03	5146	UPF3B	Thermaceae	-0.623	9.57E-06
995	CLTRN	Escherichia coli	0.318	2.09E-03	2379	KEL	Kytococcaceae	0.349	4.98E-03	3763	QTRT1	Escherichia coli	-0.188	2.17E-26	5147	UPP1	Kytococcaceae	0.054	9.21E-06
996	CLU	Actinomyces naeslundii	0.231	1.15E-06	2380	KHDC4	Ustilaginaceae	0.036	2.24E-14	3764	QTRT1	Ustilaginaceae	-0.147	3.51E-11	5148	UQCR11	Ustilaginaceae	-0.364	5.11E-04
997	CLUH	Deinococcota	0.547	1.15E-06	2381	KHDRBS2	Dolosigranulum pigrum	0.107	2.32E-04	3765	R3HCC1	Dolosigranulum pigrum	-0.528	8.11E-16	5149	UQCRB	Paucibacter	0.368	2.01E-13
998	CLVS1	Pseudomonas oleovorans	-0.351	4.05E-05	2382	KIAA0319	Haemophilus	0.508	2.76E-05	3766	R3HCC1	Nocardioides	0.143	4.44E-03	5150	UQCRFS1	Paucibacter	0.153	2.27E-05
999	CLVS2	Haemophilus parainfluenzae	0.283	6.78E-31	2383	KIAA0319L	Escherichia coli	-0.435	3.93E-11	3767	R3HCC1L	Plantactinospora sp. BC1	-0.479	8.15E-12	5151	URB1	Dolosigranulum pigrum	-0.338	2.35E-04
1000	CLVS2	Kytococcaceae	0.237	1.47E-07	2384	KIAA0825	Pseudomonas oleovorans	-0.210	1.71E-05	3768	RAB10	Escherichia coli	0.241	7.36E-03	5152	UROD	Plantactinospora	-0.526	7.22E-04
1001	CLVS2	Pseudomonas oleovorans	-0.235	4.88E-20	2385	KIAA1328	Pseudomonas oleovorans	-0.143	1.45E-04	3769	RAB14	Actinomyces naeslundii	0.252	7.84E-38	5153	USF1	Actinomyces naeslundii	-0.334	6.58E-06
1002	CMBL	Kytococcaceae	-0.359	2.30E-28	2386	KIAA1522	Dermacoccaceae	-0.229	6.72E-03	3770	RAB14	Plantactinospora	0.242	4.95E-06	5154	USF2	Mycosphaerellaceae	-0.638	2.41E-03
1003	CMPK1	Escherichia coli	0.229	3.68E-05	2387	KIAA1522	Escherichia coli	-0.496	6.25E-06	3771	RAB17	Corynebacterium tuberculostea	0.247	1.52E-08	5155	USP14	Leotiomycetes	0.462	7.60E-04
1004	CMSS1	Acinetobacter ursingii	-0.797	1.07E-13	2388	KIAA1586	Escherichia coli	0.346	5.19E-04	3772	RAB17	Plantactinospora	-0.465	5.12E-08	5156	USP15	Actinomyces naeslundii	0.043	1.08E-03
1005	CMTM8	Kytococcaceae	-0.359	2.37E-04	2389	KIAA2026	Thermaceae	-0.671	1.74E-07	3773	RAB18	Bradyrhizobium elkanii	0.519	7.48E-03	5157	USP15	Ustilaginaceae	0.424	1.29E-05
1006	CNBD2	Pseudomonas oleovorans	-0.324	1.74E-08	2390	KIF13A	Dolosigranulum pigrum	-0.280	5.13E-04	3774	RAB18	Escherichia coli	0.316	4.76E-16	5158	USP16	Thermaceae	-0.523	6.26E-04
1007	CNBP	Escherichia coli	0.379	6.41E-08	2391	KIF13B	Cutibacterium modestum	0.719	8.56E-08	3775	RAB1B	Paucibacter	-0.601	7.76E-05	5159	USP31	Actinomyces naeslundii	-0.201	6.13E-04
1008	CNBP	Gordonia bronchialis	-0.478	8.75E-08	2392	KIF15	Kytococcaceae	0.142	4.03E-05	3776	RAB21	Escherichia coli	0.268	1.36E-08	5160	USP32	Chromatiaceae	-0.167	1.07E-03
1009	CNGB1	Kytococcaceae	0.179	6.05E-10	2393	KIF16B	Corynebacterium tuberculostea	-0.369	8.86E-03	3777	RAB22A	Rhizobiaceae	-0.345	1.22E-04	5161	USP32	Methyloversatilis sp. RAC08	0.545	6.65E-05
1010	CNGB1	Pseudomonas oleovorans	-0.372	2.80E-14	2394	KIF18A	Pseudomonas oleovorans	-0.388	3.09E-06	3778	RAB23	Actinomyces naeslundii	0.429	2.37E-09	5162	USP32	Schlegelella aquatica	0.419	9.39E-04
1011	CNIH2	Kytococcaceae	-0.058	9.03E-08	2395	KIF18B	Pseudomonas oleovorans	-0.155	1.19E-04	3779	RAB24	Actinomyces naeslundii	-0.583	2.19E-25	5163	USP33	Escherichia coli	0.490	3.62E-04
1012	CNN2	Helotiales	-0.273	1.28E-03	2396	KIF1B	Acinetobacter ursingii	0.761	5.83E-09	3780	RAB26	Halomonadaceae	-0.331	3.11E-08	5164	USP37	Yersiniaceae	-0.680	3.02E-06
1013	CNNM2	Actinomyces naeslundii	0.392	1.15E-09	2397	KIF23	Kytococcaceae	0.385	1.43E-04	3781	RAB2A	Escherichia coli	0.352	2.48E-14	5165	USP40	Ustilaginaceae	0.206	1.73E-03
1014	CNOT1	Acinetobacter ursingii	0.891	3.53E-17	2398	KIF3A	Pseudomonas oleovorans	-0.316	1.15E-04	3782	RAB2B	Actinomyces naeslundii	-0.266	9.19E-05	5166	USP44	Dolosigranulum pigrum	0.340	1.83E-04
1015	CNOT3	Bradyrhizobium elkanii	-0.581	8.16E-04	2399	KIF4B	Anaerococcus	-0.494	8.82E-04	3783	RAB38	Pseudomonas oleovorans	-0.230	3.07E-03	5167	USP48	Ustilaginaceae	0.366	6.40E-05
1016	CNOT6L	Escherichia coli	0.532	2.87E-05	2400	KIF5A	Kytococcaceae	0.292	3.80E-09	3784	RAB3D	Achromobacter	-0.714	2.81E-10	5168	USP50	Dolosigranulum pigrum	0.403	3.16E-03
1017	CNP	Actinomyces naeslundii	0.147	2.95E-04	2401	KIF5C	Pseudomonas oleovorans	-0.368	1.65E-06	3785	RAB40B	Actinomyces naeslundii	-0.528	2.26E-04	5169	USP6	Pseudomonas oleovorans	-0.330	6.75E-10
1018	CNPPD1	Dolosigranulum pigrum	-0.468	5.81E-04	2402	KIF6	Pucciniaceae	-0.226	9.18E-03	3786	RAB40C	Escherichia coli	-0.439	6.46E-21	5170	USP9X	Pseudomonas oleovorans	0.672	2.55E-04
1019	CNR1	Pseudomonas oleovorans	-0.330	1.33E-05	2403	KISS1	Escherichia coli	-0.360	3.36E-03	3787	RAB4A	Gordonia bronchialis	-0.433	1.40E-03	5171	USP9Y	Debaryomycetaceae	0.593	9.94E-06
1020	CNR2	Pseudomonas oleovorans	-0.249	1.68E-11	2404	KIZ	Actinomyces naeslundii	-0.179	1.50E-03	3788	RAB5C	Actinomyces naeslundii	0.383	2.89E-07	5172	USP9Y	Rhodoferax	0.400	6.52E-09
1021	CNTN2	Pseudomonas oleovorans	-0.038	3.73E-10	2405	KLC1	Bradyrhizobium elkanii	-0.544	3.13E-03	3789	RAB6A	Escherichia coli	0.522	1.57E-06	5173	UTP23	Bradyrhizobium elkanii	0.456	6.40E-03
1022	CNTN3	Dermacoccaceae	-0.202	3.11E-07	2406	KLF4	Actinomyces naeslundii	-0.350	9.11E-03	3790	RAB6C	Debaryomycetaceae	-0.562	3.47E-03	5174	UTP25	Actinomyces naeslundii	0.523	1.15E-13
1023	CNTN6	Gemella	0.480	2.06E-05	2407	KLF4	Dolosigranulum pigrum	0.471	3.68E-03	3791	RAB7A	Stenotrophomonas maltophilia	-0.390	3.76E-06	5175	UTS2B	Pseudomonas oleovorans	-0.271	1.69E-06

1024	CNTN6	Pseudomonas oleovorans	-0.419	4.45E-09	2408	KLF4	Leptotrichiaceae	0.164	6.72E-05	3792	RAB7A	Ustilaginaceae	-0.274	3.36E-21	5176	UTY	Debaryomycetaceae	0.537	2.24E-04
1025	CNTNAP3C	Pseudomonas oleovorans	-0.189	2.14E-06	2409	KLF5	Actinomyces naeslundii	-0.278	1.84E-06	3793	RAB8B	Escherichia coli	0.674	1.08E-07	5177	UXT	Cutibacterium modestum	-0.581	9.11E-09
1026	CNTNAP5	Kytococcaceae	0.269	2.78E-10	2410	KLF6	Leptotrichiaceae	0.206	9.04E-06	3794	RABEP2	Bradyrhizobium elkanii	-0.436	1.36E-05	5178	VAC14	Escherichia coli	-0.407	1.58E-10
1027	COA1	Thermaceae	-0.635	3.20E-06	2411	KLF6	Methylorubrum	0.382	7.10E-03	3795	RABEP2	Escherichia coli	-0.226	2.30E-15	5179	VAMP4	Escherichia coli	0.481	1.11E-05
1028	COA7	Fusobacteriia	-0.434	1.10E-05	2412	KLF7	Actinomyces naeslundii	-0.083	1.15E-07	3796	RABGAP1L	Escherichia coli	0.475	4.96E-03	5180	VAMP7	Malasseziaceae	-0.507	8.55E-04
1029	COASY	Deinococcota	0.447	2.01E-05	2413	KLF9	Leptotrichiaceae	0.212	8.25E-05	3797	RABL2A	Actinomyces naeslundii	-0.271	1.59E-08	5181	VAMP7	Pseudomonas oleovorans	0.371	2.19E-05
1030	COASY	Escherichia coli	-0.280	4.82E-04	2414	KLHDC1	Actinomyces naeslundii	-0.355	1.86E-78	3798	RABL2B	Actinomyces naeslundii	-0.349	1.26E-16	5182	VANGL1	Actinomyces naeslundii	0.279	1.42E-03
1031	COG4	Escherichia coli	-0.470	2.98E-06	2415	KLHDC10	Kytococcaceae	0.134	8.71E-06	3799	RABL6	Escherichia coli	-0.242	9.28E-09	5183	VARS1	Escherichia coli	-0.331	1.26E-33
1032	COG5	Thermales	0.679	1.02E-03	2416	KLHDC7A	Actinomyces naeslundii	0.312	3.12E-03	3800	RAD21	Bradyrhizobium elkanii	0.290	5.79E-09	5184	VAT1	Helotiales	-0.499	4.97E-03
1033	COG8	Actinomyces naeslundii	0.385	1.65E-04	2417	KLHL11	Escherichia coli	0.468	6.92E-04	3801	RAD21	Escherichia coli	0.465	1.17E-39	5185	VAV2	Escherichia coli	-0.420	3.64E-07
1034	COL11A1	Kytococcaceae	0.285	3.95E-08	2418	KLHL15	Escherichia coli	0.433	9.55E-16	3802	RAD51D	Pseudomonas oleovorans	-0.374	9.18E-05	5186	VCP	Actinomyces naeslundii	0.439	2.60E-33
1035	COL11A1	Pseudomonas oleovorans	-0.218	2.74E-11	2419	KLHL24	Escherichia coli	0.505	3.43E-09	3803	RAD52	Actinomyces naeslundii	-0.284	1.60E-06	5187	VCPIP1	Escherichia coli	0.638	2.71E-23
1036	COL13A1	Kytococcaceae	0.126	3.56E-06	2420	KLHL25	Kytococcaceae	0.263	5.24E-04	3804	RAD54B	Dolosigranulum pigrum	0.389	7.03E-03	5188	VCPKMT	Actinomyces naeslundii	-0.289	2.57E-03
1037	COL16A1	Actinomyces naeslundii	-0.317	3.74E-08	2421	KLHL28	Escherichia coli	0.443	3.86E-06	3805	RADIL	Pseudomonas oleovorans	-0.439	5.90E-08	5189	VEGFA	Actinomyces naeslundii	-0.328	4.10E-12
1038	COL17A1	Pseudomonas oleovorans	-0.272	4.71E-06	2422	KLHL29	Kytococcaceae	0.154	6.43E-04	3806	RAET1E	Pseudomonas oleovorans	-0.434	7.38E-05	5190	VENTX	Actinomyces naeslundii	-0.474	8.04E-05
1039	COL18A1	Escherichia coli	-0.173	3.56E-36	2423	KLHL9	Escherichia coli	0.325	1.84E-05	3807	RAI1	Cutibacterium modestum	0.605	1.88E-06	5191	VEPH1	Dolosigranulum pigrum	0.224	4.40E-03
1040	COL20A1	Kytococcaceae	0.208	4.33E-08	2424	KLRD1	Bifidobacteriales	0.397	1.31E-04	3808	RALBP1	Dolosigranulum pigrum	-0.264	1.03E-03	5192	VEZT	Rhizobiaceae	-0.226	4.18E-06
1041	COL20A1	Pseudomonas oleovorans	-0.396	6.24E-19	2425	KNG1	Pseudomonas oleovorans	0.575	9.83E-08	3809	RALGAPB	Empedobacter falsenii	0.638	2.37E-05	5193	VGLL3	Pseudomonas oleovorans	-0.406	3.06E-03
1042	COL22A1	Kytococcaceae	0.185	3.06E-04	2426	KPNA3	Acidimicrobiales	-0.325	6.24E-05	3810	RALYL	Pseudomonas oleovorans	-0.200	4.72E-06	5194	VIP	Actinomyces naeslundii	-0.492	1.71E-03
1043	COL24A1	Pseudomonas oleovorans	-0.362	1.19E-06	2427	KPNA4	Escherichia coli	0.319	5.18E-03	3811	RANBP3	Bradyrhizobium elkanii	-0.571	2.76E-05	5195	VIP	Dolosigranulum pigrum	0.291	2.16E-05
1044	COL27A1	Ustilaginaceae	0.044	3.80E-04	2428	KPNA5	Dolosigranulum pigrum	0.531	1.51E-06	3812	RANBP3L	Escherichia coli	0.341	1.24E-03	5196	VIT	Pseudomonas oleovorans	-0.334	1.10E-03
1045	COL2A1	Pseudomonas oleovorans	-0.357	1.18E-30	2429	KPNA6	Chaetomiaceae	-0.353	6.97E-03	3813	RANBP9	Escherichia coli	0.301	3.77E-03	5197	VKORC1	Nocardioides	0.272	3.46E-04
1046	COL4A4	Dolosigranulum pigrum	0.237	3.05E-03	2430	KPTN	Escherichia coli	-0.269	7.73E-03	3814	RANGAP1	Escherichia coli	-0.540	8.89E-10	5198	VMA21	Escherichia coli	0.342	3.48E-13
1047	COL6A1	Bradyrhizobium elkanii	-0.572	7.17E-06	2431	KPTN	Ustilaginaceae	-0.141	1.81E-03	3815	RANGAP1	Kytococcaceae	0.159	2.37E-19	5199	VMP1	Actinomyces naeslundii	-0.369	1.58E-16
1048	COL6A2	Bradyrhizobium elkanii	-0.474	5.39E-05	2432	KRBOX4	Actinomyces naeslundii	-0.270	1.18E-04	3816	RAP1A	Escherichia coli	0.247	9.49E-07	5200	VPS16	Deinococcota	0.462	1.28E-04
1049	COL6A2	Escherichia coli	-0.196	1.62E-03	2433	KRT10	Alphaproteobacteria	-0.520	2.20E-07	3817	RAP1B	Escherichia coli	0.540	1.51E-07	5201	VPS29	Cutibacterium modestum	-0.684	9.29E-12
1050	COL6A5	Pseudomonas oleovorans	-0.250	1.62E-11	2434	KRT10	Chromatiaceae	0.165	1.11E-04	3818	RAP2A	Escherichia coli	0.327	8.30E-03	5202	VPS29	Escherichia coli	0.142	1.16E-13
1051	COL8A2	Actinomyces naeslundii	-0.285	2.20E-03	2435	KRT4	Pseudomonas oleovorans	-0.001	5.50E-13	3819	RAP2B	Escherichia coli	0.448	9.06E-03	5203	VPS35	Actinomyces naeslundii	0.236	4.45E-08
1052	COL9A2	Dolosigranulum pigrum	0.162	1.12E-14	2436	KRT7	Escherichia coli	-0.431	5.44E-04	3820	RAP2B	Ustilaginaceae	-0.285	9.82E-04	5204	VPS36	Aspergillaceae	0.446	5.66E-06
1053	COL9A3	Escherichia coli	-0.455	1.03E-09	2437	KRT73	Pseudomonas oleovorans	-0.248	5.97E-06	3821	RAPSN	Escherichia coli	-0.257	1.32E-05	5205	VPS37D	Halomonadaceae	-0.215	7.23E-04
1054	COLEC10	Actinomyces naeslundii	0.168	6.14E-06	2438	KRT8	Escherichia coli	-0.539	5.78E-05	3822	RASA3	Ottowia	0.605	4.60E-03	5206	VPS37D	Ustilaginaceae	0.115	2.05E-04
1055	COLGALT2	Pseudomonas oleovorans	-0.316	8.21E-04	2439	KRT80	Kytococcaceae	0.220	2.26E-05	3823	RASAL1	Kytococcaceae	0.210	9.66E-07	5207	VPS39	Staphylococcus epidermidis	-0.383	4.67E-03
1056	COMMD1	Cutibacterium modestum	-0.666	1.07E-03	2440	KRT81	Dolosigranulum pigrum	0.175	3.75E-23	3824	RASGRF1	Pseudomonas oleovorans	-0.381	1.38E-09	5208	VPS45	Ustilaginaceae	0.290	4.52E-05
1057	COMMD10	Escherichia coli	0.099	1.69E-05	2441	KRTAP5-10	Actinomyces naeslundii	-0.390	3.95E-15	3825	RASGRF2	Acinetobacter johnsonii	0.432	7.90E-04	5209	VPS4B	Escherichia coli	0.535	2.26E-06
1058	COMMD3	Leotiomycetes	0.539	4.53E-03	2442	KRTAP5-10	Ustilaginaceae	0.193	1.19E-05	3826	RASGRP1	Ottowia	0.476	1.65E-11	5210	VPS51	Escherichia coli	-0.267	1.53E-05
1059	COMMD8	Escherichia coli	0.269	3.15E-04	2443	KRTCAP2	Micromonosporales	-0.439	4.86E-03	3827	RASGRP2	Bradyrhizobium elkanii	-0.732	7.59E-04	5211	VPS53	Escherichia coli	-0.344	7.48E-04
1060	COMTD1	Escherichia coli	-0.344	5.26E-05	2444	KRTCAP3	Moraxellaceae	0.535	4.66E-04	3828	RASIP1	Methylobacterium sp. FF17	-0.654	8.74E-06	5212	VPS9D1	Actinomyces naeslundii	-0.310	8.07E-229
1061	COP1	Bradyrhizobium elkanii	0.517	6.95E-03	2445	KSR1	Ustilaginaceae	-0.057	4.52E-06	3829	RASL10A	Dolosigranulum pigrum	0.143	1.99E-05	5213	VRK1	Bacillaceae	-0.502	1.40E-04
1062	COPB1	Methylobacterium sp. FF17	0.557	3.20E-05	2446	KSR2	Kytococcaceae	0.289	4.65E-04	3830	RASL11A	Dolosigranulum pigrum	0.240	1.50E-04	5214	VSIG1	Dolosigranulum pigrum	0.124	3.64E-06
1063	COPRS	Campylobacterota	0.406	2.56E-05	2447	KSR2	Pseudomonas oleovorans	-0.290	1.61E-19	3831	RASSF7	Escherichia coli	-0.502	3.12E-114	5215	VSIG4	Paucibacter	-0.366	9.56E-04
1064	COPZ1	Actinomyces naeslundii	0.375	6.85E-13	2448	KY	Pseudomonas oleovorans	-0.275	1.55E-10	3832	RB1	Escherichia coli	0.555	1.96E-08	5216	VSIG4	Thermaceae	0.664	1.22E-07
1065	COQ10A	Halomonadaceae	-0.172	1.76E-03	2449	L1CAM	Pseudomonas oleovorans	-0.305	6.90E-11	3833	RB1CC1	Carnobacteriaceae	0.569	7.84E-03	5217	VSTM2L	Mycobacteriaceae	0.672	1.16E-06
1066	COQ2	Actinomyces naeslundii	0.103	3.69E-21	2450	L1TD1	Kytococcaceae	0.127	1.94E-04	3834	RB1CC1	Dolosigranulum	0.410	3.40E-03	5218	VSX1	Kytococcaceae	0.250	1.59E-06
1067	COQ4	Actinomyces naeslundii	-0.507	2.63E-23	2451	L1TD1	Pseudomonas oleovorans	-0.433	1.34E-06	3835	RBAK	Ustilaginaceae	0.497	2.24E-05	5219	VSX1	Pseudomonas oleovorans	-0.363	1.52E-15
1068	COQ8B	Escherichia coli	-0.418	4.69E-03	2452	L3HYPDH	Ustilaginaceae	0.299	5.08E-05	3836	RBBP4	Oxalobacteraceae	0.447	1.58E-05	5220	VTI1B	Actinomyces naeslundii	0.212	1.63E-72
1069	CORIN	Pseudomonas oleovorans	-0.183	7.86E-07	2453	LACTB	Bradyrhizobium elkanii	0.586	2.02E-08	3837	RBBP5	Actinomyces naeslundii	0.489	1.70E-04	5221	VTN	Actinomyces naeslundii	0.204	1.17E-10
1070	CORO1B	Leotiomycetes	-0.561	2.79E-05	2454	LACTB2	Bradyrhizobium elkanii	0.580	8.14E-05	3838	RBBP6	Actinomyces naeslundii	-0.138	1.33E-18	5222	VWA3A	Kytococcaceae	0.202	1.44E-04
1071	CORO2B	Dolosigranulum pigrum	0.290	1.07E-05	2455	LACTB2	Escherichia coli	0.356	9.06E-06	3839	RBCK1	Actinomyces naeslundii	-0.445	1.76E-10	5223	VWA3A	Pseudomonas oleovorans	-0.384	7.15E-08
1072	CORO7	Actinomyces naeslundii	-0.310	5.32E-05	2456	LAMB1	Lawsonellaceae	0.385	5.84E-04	3840	RBFOX1	Kytococcaceae	0.269	1.05E-09	5224	VWA3B	Kytococcaceae	0.309	6.19E-04
1073	COTL1	Ustilaginaceae	-0.344	2.09E-03	2457	LAMB2	Alphaproteobacteria	0.441	3.57E-06	3841	RBFOX1	Pseudomonas oleovorans	-0.237	3.20E-15	5225	VWA5B1	Pseudomonas oleovorans	-0.218	1.41E-21
1074	COX20	Leotiomycetes	0.495	3.78E-05	2458	LAMB2	Escherichia coli	-0.233	3.59E-05	3842	RBFOX3	Pseudomonas oleovorans	-0.310	6.69E-09	5226	VWC2	Kytococcaceae	0.190	1.25E-04
1075	COX5A	Paucibacter	0.277	1.24E-04	2459	LAMB4	Pseudomonas oleovorans	-0.269	3.33E-05	3843	RBIS	Yersiniaceae	0.713	9.97E-03	5227	VWC2	Pseudomonas oleovorans	-0.280	8.83E-07
1076	COX5A	Ustilaginaceae	-0.068	5.33E-09	2460	LAMC1	Thermales	0.715	2.66E-04	3844	RBM10	Paucibacter	-0.365	2.67E-04	5228	VWDE	Kytococcaceae	0.199	6.32E-03
1077	COX6A1	Bradyrhizobium elkanii	0.607	2.62E-05	2461	LAMP1	Actinomyces naeslundii	0.314	1.79E-08	3845	RBM10	Ustilaginaceae	-0.014	4.95E-08	5229	VXN	Actinomyces naeslundii	-0.186	6.65E-18

1078	COX6A1	Cutibacterium modestum	-0.726	6.44E-03	2462	LAMTOR1	Ustilaginaceae	-0.314	3.35E-04	3846	RBM19	Escherichia coli	-0.238	4.02E-05	5230	WAC	Pseudomonas oleovorans	0.389	5.95E-03
1079	CPA3	Caudoviricetes	0.417	4.94E-03	2463	LAMTOR3	Escherichia coli	0.216	1.29E-08	3847	RBM20	Pseudomonas oleovorans	-0.280	1.64E-06	5231	WASHC1	Actinomyces naeslundii	-0.078	4.21E-09
1080	CPD	Actinomyces naeslundii	0.232	5.10E-07	2464	LAMTOR4	Chromatiales	0.353	5.45E-03	3848	RBM24	Kytococcaceae	0.052	5.14E-04	5232	WASHC4	Escherichia coli	0.474	4.48E-06
1081	CPEB1	Pseudomonas oleovorans	-0.326	1.70E-04	2465	LANCL3	Kytococcaceae	0.114	6.24E-05	3849	RBM25	Actinomyces naeslundii	-0.180	2.65E-26	5233	WASL	Escherichia coli	0.549	1.81E-11
1082	CPEB2	Escherichia coli	0.452	1.87E-06	2466	LANCL3	Pseudomonas oleovorans	-0.384	2.78E-08	3850	RBM26	Ceratobasidiaceae	0.556	5.05E-03	5234	WASL	Kytococcaceae	-0.125	1.22E-21
1083	CPEB4	Leptotrichiaceae	0.218	4.95E-03	2467	LAP3	Actinomyces naeslundii	0.411	6.87E-15	3851	RBM26	Thermaceae	-0.726	6.60E-06	5235	WBP1L	Ceratobasidiaceae	-0.252	9.62E-03
1084	CPLANE2	Dolosigranulum pigrum	-0.477	3.34E-06	2468	LAPTM4A	Actinomyces naeslundii	0.110	5.09E-281	3852	RBM27	Escherichia coli	0.398	1.87E-04	5236	WBP2NL	Pseudomonas oleovorans	-0.362	2.78E-03
1085	CPN1	Actinomyces naeslundii	0.287	8.27E-16	2469	LAPTM4A	Ustilaginaceae	0.032	1.22E-78	3853	RBM28	Actinomyces naeslundii	-0.163	6.66E-26	5237	WDR11	Gardnerella	0.526	4.09E-04
1086	CPN2	Actinomyces naeslundii	0.450	1.10E-16	2470	LARGE1	Cutibacterium acnes	-0.506	4.41E-04	3854	RBM3	Actinomyces naeslundii	-0.356	2.04E-04	5238	WDR18	Escherichia coli	-0.428	2.39E-11
1087	CPNE3	Escherichia coli	0.384	1.59E-03	2471	LARP4	Escherichia coli	0.371	2.08E-03	3855	RBM38	Escherichia coli	-0.249	9.40E-04	5239	WDR19	Ustilaginaceae	0.263	4.34E-08
1088	CPNE7	Kytococcaceae	-0.089	1.22E-04	2472	LARP7	Thermaceae	-0.771	5.45E-05	3856	RBM39	Actinomyces naeslundii	-0.293	4.88E-14	5240	WDR24	Deinococcota	0.599	1.59E-05
1089	CPO	Actinomyces naeslundii	-0.267	6.64E-06	2473	LASP1	Actinomyces naeslundii	0.206	3.26E-04	3857	RBM41	Ustilaginaceae	0.356	1.28E-07	5241	WDR24	Escherichia coli	-0.239	1.47E-14
1090	CPPED1	Actinomyces naeslundii	0.318	1.72E-38	2474	LAT	Actinomyces naeslundii	-0.384	4.18E-43	3858	RBM44	Dolosigranulum pigrum	0.453	2.99E-06	5242	WDR44	Escherichia coli	0.383	1.17E-03
1091	CPS1	Pseudomonas oleovorans	0.511	9.56E-06	2475	LBP	Actinomyces naeslundii	0.274	9.80E-33	3859	RBM5	Actinomyces naeslundii	-0.161	8.44E-24	5243	WDR45	Actinomyces naeslundii	-0.333	1.84E-04
1092	CPSF2	Actinomyces naeslundii	0.473	2.44E-04	2476	LCAT	Paucibacter	-0.523	5.70E-03	3860	RBM6	Actinomyces naeslundii	-0.248	1.47E-35	5244	WDR46	Escherichia coli	-0.443	1.06E-09
1093	CPSF7	Actinomyces naeslundii	-0.277	1.62E-20	2477	LCLAT1	Actinomyces naeslundii	0.366	7.37E-04	3861	RBMS3	Escherichia coli	0.514	5.67E-03	5245	WDR49	Pseudomonas oleovorans	-0.274	1.13E-05
1094	CPT2	Actinomyces naeslundii	0.447	1.10E-06	2478	LCOR	Escherichia coli	0.374	7.41E-06	3862	RBP4	Rhizobiaceae	0.470	1.27E-06	5246	WDR5	Escherichia coli	-0.438	1.84E-11
1095	CPT2	Dolosigranulum pigrum	-0.223	2.61E-15	2479	LDB2	Paucibacter	-0.184	6.87E-28	3863	RBP5	Paucibacter	-0.506	5.33E-15	5247	WDR53	Ustilaginaceae	0.235	2.20E-05
1096	CPTP	Dolosigranulum pigrum	-0.469	9.48E-05	2480	LDB3	Pseudomonas oleovorans	-0.348	6.79E-04	3864	RBPJ	Escherichia coli	0.496	4.85E-15	5248	WDR59	Actinomyces naeslundii	-0.295	4.94E-25
1097	CR2	Pseudomonas oleovorans	-0.326	7.22E-07	2481	LDHAL6A	Pseudomonas oleovorans	-0.239	9.20E-03	3865	RBPMS	Actinomyces naeslundii	-0.457	5.60E-05	5249	WDR5B	Ceratobasidiaceae	0.392	5.80E-03
1098	CRACDL	Pseudomonas oleovorans	-0.488	1.15E-04	2482	LDHD	Leotiomycetes	-0.545	4.16E-04	3866	RCBTB1	Escherichia coli	0.410	2.15E-03	5250	WDR62	Escherichia coli	-0.395	8.22E-04
1099	CRAT	Paucibacter	-0.647	7.79E-07	2483	LDHD	Paucibacter	-0.487	9.36E-06	3867	RCC1L	Actinomyces naeslundii	0.219	1.51E-04	5251	WDR62	Kytococcaceae	0.351	6.93E-03
1100	CRB2	Pseudomonas oleovorans	-0.556	1.38E-08	2484	LDLR	Actinomyces naeslundii	-0.360	2.21E-07	3868	RCCD1	Actinomyces naeslundii	-0.426	6.47E-07	5252	WDR64	Pseudomonas oleovorans	-0.146	4.04E-04
1101	CRB3	Actinomyces naeslundii	0.241	3.38E-03	2485	LDLRAD3	Dolosigranulum pigrum	0.292	2.87E-04	3869	RCHY1	Escherichia coli	0.258	1.55E-04	5253	WDR7	Actinomyces naeslundii	0.445	3.56E-05
1102	CRBN	Escherichia coli	0.242	2.61E-04	2486	LDLRAP1	Escherichia coli	-0.395	4.44E-04	3870	RCL1	Leptotrichiaceae	0.080	3.35E-05	5254	WDR73	Actinomyces naeslundii	-0.360	1.06E-21
1103	CREB3L2	Comamonadaceae	-0.324	4.80E-04	2487	LECT2	Cutibacterium modestum	-0.654	1.84E-10	3871	RCOR1	Escherichia coli	0.531	5.86E-03	5255	WDR75	Actinomyces naeslundii	-0.114	6.60E-12
1104	CREBRF	Escherichia coli	0.486	1.60E-06	2488	LEKR1	Dolosigranulum pigrum	0.261	2.94E-03	3872	RCSD1	Escherichia coli	0.644	6.74E-03	5256	WDR86	Pseudomonas oleovorans	-0.238	1.79E-07
1105	CREBZF	Actinomyces naeslundii	-0.338	2.98E-10	2489	LEMD3	Escherichia coli	0.359	3.25E-06	3873	RCSD1	Pseudomonadaceae	0.745	8.78E-03	5257	WDR87	Pseudomonas oleovorans	-0.373	1.81E-17
1106	CRELD2	Leptotrichiaceae	-0.254	2.50E-03	2490	LFNG	Enterobacterales	-0.459	7.29E-06	3874	RDH10	Escherichia coli	0.379	2.09E-04	5258	WDTC1	Hyphomicrobiales	0.478	9.02E-06
1107	CRHBP	Paucibacter	-0.481	3.72E-10	2491	LGALS3BP	Actinomyces naeslundii	0.283	5.13E-05	3875	RDH13	Escherichia coli	-0.430	4.10E-08	5259	WDTC1	Leotiomycetes	-0.613	3.70E-04
1108	CRIP2	Escherichia coli	-0.196	2.37E-04	2492	LGI4	Actinomyces naeslundii	-0.160	5.64E-08	3876	RDH5	Escherichia coli	-0.426	9.74E-08	5260	WFDC3	Pseudomonas oleovorans	-0.182	5.74E-24
1109	CRIPT	Escherichia coli	0.305	9.68E-09	2493	LGMN	Ustilaginaceae	-0.323	2.40E-06	3877	REC8	Actinomyces naeslundii	-0.482	8.03E-25	5261	WIPF1	Escherichia coli	0.609	3.84E-07
1110	CRLS1	Cutibacterium modestum	-0.683	2.30E-06	2494	LGR4	Escherichia coli	0.374	4.52E-03	3878	RECQL	Malassezia vespertilionis	-0.417	4.26E-03	5262	WNK1	Acinetobacter ursingii	0.749	8.26E-03
1111	CRMP1	Pseudomonas oleovorans	-0.290	6.97E-10	2495	LHPP	Dolosigranulum pigrum	-0.374	1.67E-04	3879	RECQL4	Escherichia coli	-0.533	1.84E-15	5263	WNK4	Pseudomonas oleovorans	-0.276	3.72E-07
1112	CROCC2	Escherichia coli	-0.351	3.45E-04	2496	LIFR	Escherichia coli	0.523	1.59E-13	3880	REELD1	Dolosigranulum pigrum	0.178	1.90E-03	5264	WNT7B	Escherichia coli	-0.228	6.93E-09
1113	CRTC1	Bradyrhizobium elkanii	-0.665	2.29E-08	2497	LILRA2	Paucibacter	-0.262	1.13E-04	3881	REEP3	Escherichia coli	0.212	3.13E-20	5265	WRAP53	Escherichia coli	-0.395	3.10E-05
1114	CRTC1	Escherichia coli	-0.181	2.05E-03	2498	LILRA4	Pseudomonas oleovorans	-0.284	3.15E-08	3882	REEP5	Bradyrhizobium elkanii	0.579	8.57E-07	5266	WRAP53	Rhizobiaceae	0.358	1.29E-04
1115	CRTC2	Actinomyces naeslundii	-0.387	1.24E-23	2499	LILRA5	Rhodocyclales	-0.464	6.42E-03	3883	REL	Actinomyces naeslundii	-0.186	4.22E-20	5267	WSB1	Actinomyces naeslundii	-0.348	1.42E-06
1116	CRX	Actinomyces naeslundii	-0.321	3.93E-48	2500	LILRA6	Gordonia bronchialis	0.438	8.17E-04	3884	REL	Dolosigranulum pigrum	0.556	9.91E-07	5268	WT1	Paucibacter	0.568	1.90E-06
1117	CRX	Ustilaginaceae	0.158	2.76E-13	2501	LILRB2	Cutibacterium acnes	0.527	1.52E-04	3885	RELCH	Ustilaginaceae	0.310	5.86E-04	5269	WT1	Pseudomonas oleovorans	-0.500	2.36E-09
1118	CRYAB	Dolosigranulum pigrum	0.398	9.42E-03	2502	LILRB5	Paucibacter	-0.299	3.88E-13	3886	RELN	Actinomyces naeslundii	0.203	7.55E-04	5270	WTAP	Thermaceae	-0.639	1.97E-05
1119	CRYBB3	Ustilaginaceae	0.120	9.69E-18	2503	LILRB5	Sphingobacterium	0.669	6.04E-03	3887	RERE	Bradyrhizobium elkanii	-0.729	2.35E-06	5271	WWOX	Actinomyces naeslundii	0.367	9.69E-09
1120	CRYGS	Actinomyces naeslundii	-0.134	9.60E-06	2504	LIMCH1	Sphingobacterium	0.652	6.00E-05	3888	REST	Acidovorax temperans	-0.326	5.10E-03	5272	WWTR1	Escherichia coli	0.424	3.02E-05
1121	CRYL1	Dolosigranulum pigrum	-0.425	1.98E-03	2505	LIMD1	Escherichia coli	-0.377	2.08E-05	3889	REV1	Ustilaginaceae	0.299	2.11E-04	5273	XAB2	Bradyrhizobium elkanii	-0.529	2.68E-03
1122	CSAD	Actinomyces naeslundii	-0.435	4.97E-13	2506	LIME1	Escherichia coli	-0.246	2.64E-17	3890	REX1BD	Escherichia coli	-0.182	6.12E-04	5274	XAB2	Deinococcota	0.527	2.41E-08
1123	CSDE1	Actinomyces naeslundii	0.355	5.80E-10	2507	LIMS1	Escherichia coli	0.519	1.64E-23	3891	REXO4	Deinococcota	0.394	3.31E-03	5275	XAB2	Escherichia coli	-0.157	2.33E-11
1124	CSE1L	Actinomyces naeslundii	0.502	1.59E-05	2508	LIMS1	Staphylococcus epidermidis	0.424	1.88E-03	3892	RFC3	Pseudomonas oleovorans	-0.254	5.31E-05	5276	XAF1	Actinomyces naeslundii	-0.237	4.77E-05
1125	CSF1R	Paucibacter	-0.352	1.87E-13	2509	LIMS2	Escherichia coli	-0.236	1.23E-07	3893	RFK	Escherichia coli	0.271	2.16E-20	5277	XAF1	Plantactinospora	-0.530	4.32E-03
1126	CSMD1	Kytococcaceae	0.169	2.82E-09	2510	LIN54	Escherichia coli	0.491	2.07E-06	3894	RFK	Streptococcaceae	0.473	1.32E-09	5278	XAF1	Ustilaginaceae	0.214	1.80E-05
1127	CSMD1	Pseudomonas oleovorans	-0.342	4.07E-15	2511	LIN7B	Pseudomonas oleovorans	-0.303	3.48E-04	3895	RFNG	Paucibacter	-0.369	3.83E-04	5279	XDH	Haemophilus parainfluenzae	0.461	5.92E-03
1128	CSMD2	Pseudomonas oleovorans	-0.330	8.65E-08	2512	LIN7C	Escherichia coli	0.442	9.54E-05	3896	RFX2	Kytococcaceae	0.017	4.52E-04	5280	XKR4	Pseudomonas oleovorans	-0.157	6.49E-03
1129	CSMD3	Kytococcaceae	0.206	1.46E-05	2513	LINGO2	Escherichia coli	-0.389	5.83E-03	3897	RFX8	Pseudomonas oleovorans	-0.337	1.65E-05	5281	XKR6	Pseudomonas oleovorans	-0.326	1.48E-05
1130	CSNK1A1	Actinomyces naeslundii	0.483	3.70E-50	2514	LINGO2	Pseudomonas oleovorans	-0.247	2.90E-11	3898	RFXANK	Paucibacter	-0.280	9.98E-03	5282	XNDC1N	Pseudomonas oleovorans	-0.265	2.77E-04
1131	CSNK1G2	Escherichia coli	-0.238	3.36E-23	2515	LIPM	Pseudomonas oleovorans	-0.225	1.98E-03	3899	RFXAP	Dolosigranulum pigrum	0.374	1.59E-10	5283	XPO5	Kytococcaceae	0.461	1.12E-05

1132	CSNK1G3	Escherichia coli	0.348	8.68E-07	2516	LIPN	Pseudomonas oleovorans	-0.045	9.96E-04	3900	RGL2	Paucibacter	-0.280	6.49E-03	5284	XRCC3	Escherichia coli	-0.385	2.84E-08
1133	CSRNP1	Methylorubrum	0.437	1.72E-05	2517	LIX1L	Sclerotiniaceae	-0.741	1.48E-03	3901	RGL3	Actinomyces naeslundii	-0.331	3.94E-24	5285	XRCC3	Lawsonellaceae	0.305	6.78E-03
1134	CSRNP2	Actinomyces naeslundii	-0.418	1.81E-09	2518	LIX1L	Sordariales	0.672	7.90E-03	3902	RGMA	Pseudomonas oleovorans	-0.286	5.41E-06	5286	XRCC3	Paucibacter	-0.409	4.42E-03
1135	CSRP2	Bradyrhizobium elkanii	0.463	1.48E-03	2519	LLGL2	Bradyrhizobium elkanii	-0.496	9.82E-04	3903	RGPD2	Actinomyces naeslundii	-0.236	3.51E-03	5287	XRCC6	Actinomyces naeslundii	0.513	1.77E-12
1136	CTBP1	Bradyrhizobium elkanii	-0.676	5.76E-03	2520	LLGL2	Escherichia coli	-0.161	2.16E-09	3904	RGPD3	Actinomyces naeslundii	-0.254	3.23E-05	5288	XRN1	Escherichia coli	0.651	7.30E-06
1137	CTBP2	Dolosigranulum pigrum	0.430	2.67E-04	2521	LMAN2	Actinomyces naeslundii	0.238	2.60E-04	3905	RGPD5	Actinomyces naeslundii	-0.284	1.53E-17	5289	XXYLT1	Dolosigranulum pigrum	0.277	1.75E-07
1138	CTBS	Escherichia coli	0.479	1.09E-04	2522	LMBR1	Actinomyces naeslundii	0.234	5.16E-15	3906	RGS1	Streptococcus oralis	-0.490	3.02E-11	5290	YBX2	Halomonadaceae	-0.221	7.38E-06
1139	CTCFL	Pseudomonas oleovorans	-0.372	1.77E-06	2523	LMBR1L	Actinomyces naeslundii	-0.429	7.37E-16	3907	RGS11	Actinomyces naeslundii	-0.331	1.89E-21	5291	YES1	Actinomyces naeslundii	0.304	2.93E-06
1140	CTDSP1	Enterobacterales	-0.543	1.92E-03	2524	LMBRD2	Bradyrhizobium elkanii	0.569	1.23E-06	3908	RGS12	Escherichia coli	-0.416	9.77E-27	5292	YIF1A	Actinomyces naeslundii	0.332	5.75E-03
1141	CTDSP1	Paucibacter	-0.362	1.70E-03	2525	LMBRD2	Escherichia coli	0.239	1.47E-04	3909	RGS13	Kytococcaceae	0.183	2.40E-04	5293	YIF1A	Paucibacter	-0.649	3.64E-04
1142	CTDSP2	Pseudomonas oleovorans	0.320	1.45E-12	2526	LMF1	Escherichia coli	-0.438	1.18E-10	3910	RGS17	Dolosigranulum pigrum	0.211	4.06E-05	5294	YIF1B	Escherichia coli	-0.322	7.52E-06
1143	CTDSPL2	Escherichia coli	0.523	4.99E-17	2527	LMF2	Escherichia coli	-0.271	5.45E-09	3911	RGS18	Escherichia coli	0.633	1.12E-03	5295	YIPF2	Dolosigranulum pigrum	-0.463	5.05E-03
1144	CTHRC1	Dolosigranulum pigrum	0.326	5.92E-03	2528	LMF2	Paucibacter	-0.326	1.44E-03	3912	RGS19	Thermaceae	0.582	3.13E-04	5296	YIPF2	Leotiomycetes	-0.578	9.96E-03
1145	CTIF	Escherichia coli	-0.242	3.87E-14	2529	LMNA	Escherichia coli	-0.358	3.09E-25	3913	RGS22	Kytococcaceae	0.079	3.90E-13	5297	YKT6	Escherichia coli	-0.588	2.05E-06
1146	CTLA4	Dolosigranulum pigrum	0.274	3.12E-03	2530	LMNA	Kytococcaceae	0.339	7.25E-49	3914	RGS22	Pseudomonas oleovorans	-0.515	4.87E-39	5298	YLPM1	Cutibacterium modestum	0.607	1.16E-10
1147	CTNNA2	Pseudomonas oleovorans	-0.350	9.04E-06	2531	LMNB2	Escherichia coli	-0.328	1.35E-07	3915	RGS5	Escherichia coli	0.576	1.25E-03	5299	YPEL5	Escherichia coli	0.391	5.52E-23
1148	CTNNB1	Pseudomonas oleovorans	0.553	3.85E-04	2532	LMNTD2	Escherichia coli	-0.186	5.89E-03	3916	RGS6	Pseudomonas oleovorans	-0.507	9.82E-28	5300	YWHAE	Actinomyces naeslundii	0.284	2.74E-18
1149	CTNND1	Yersiniaceae	-0.637	7.63E-03	2533	LMO3	Pseudomonas oleovorans	-0.486	2.38E-04	3917	RHBDD1	Actinomyces naeslundii	0.462	4.96E-05	5301	YWHAG	Actinomyces naeslundii	0.284	3.52E-05
1150	CTNS	Ustilaginaceae	0.194	2.38E-03	2534	LMTK2	Acinetobacter ursingii	0.775	5.19E-16	3918	RHBDD3	Escherichia coli	-0.683	2.17E-15	5302	YWHAZ	Escherichia coli	0.425	5.32E-08
1151	CTPS2	Actinomyces naeslundii	-0.124	8.28E-03	2535	LMTK3	Kytococcaceae	0.170	1.11E-04	3919	RHBDL1	Escherichia coli	-0.329	9.14E-18	5303	ZAN	Kytococcaceae	0.110	1.47E-11
1152	CTR9	Actinomyces naeslundii	0.575	3.57E-11	2536	LNPEP	Propionibacteriaceae	0.464	2.19E-03	3920	RHD	Pseudomonas oleovorans	-0.269	1.33E-06	5304	ZAN	Pseudomonas oleovorans	-0.408	1.17E-12
1153	CTRL	Enterobacterales	-0.557	2.14E-05	2537	LNPK	Escherichia coli	0.216	2.10E-04	3921	RHEBL1	Dolosigranulum pigrum	0.267	2.22E-04	5305	ZBBX	Kytococcaceae	0.201	5.63E-05
1154	CTSA	Actinomyces naeslundii	0.396	8.36E-18	2538	LONP1	Escherichia coli	-0.189	7.70E-10	3922	RHOA	Actinomyces naeslundii	0.254	3.35E-30	5306	ZBBX	Pseudomonas oleovorans	-0.298	6.03E-09
1155	CTSA	Paucibacter	-0.594	5.02E-08	2539	LOXHD1	Pseudomonas oleovorans	-0.409	4.25E-15	3923	RHOB	Leptotrichiaceae	0.105	4.51E-05	5307	ZBED6	Acinetobacter ursingii	0.814	1.41E-17
1156	CTSB	Ustilaginaceae	-0.279	3.61E-10	2540	LPAR2	Dolosigranulum pigrum	0.343	1.42E-11	3924	RHOC	Paucibacter	-0.533	7.24E-03	5308	ZBTB1	Actinomyces naeslundii	-0.183	1.11E-05
1157	CTSD	Actinomyces naeslundii	0.243	2.47E-04	2541	LPAR4	Acinetobacter johnsonii	0.339	6.96E-04	3925	RHOQ	Dolosigranulum pigrum	0.465	1.90E-18	5309	ZBTB11	Dolosigranulum pigrum	0.485	2.81E-06
1158	CTSF	Dolosigranulum pigrum	-0.376	1.09E-04	2542	LPAR4	Pseudomonas oleovorans	-0.481	1.65E-04	3926	RHOQ	Methyloversatilis sp. RAC08	-0.387	6.97E-06	5310	ZBTB12	Nostocales	0.322	7.62E-09
1159	CTSH	Dolosigranulum pigrum	-0.358	3.40E-09	2543	LPAR6	Ceratobasidiaceae	0.021	2.81E-04	3927	RHOT1	Escherichia coli	0.338	5.75E-03	5311	ZBTB44	Escherichia coli	0.315	2.80E-03
1160	CTSL	Ustilaginaceae	-0.384	7.01E-07	2544	LPAR6	Escherichia coli	0.523	3.58E-04	3928	RHOT2	Escherichia coli	-0.149	3.03E-10	5312	ZBTB46	Bradyrhizobium elkanii	-0.676	9.06E-09
1161	CTSS	Actinomyces naeslundii	0.191	1.99E-11	2545	LPCAT2	Escherichia coli	0.573	1.33E-08	3929	RHOXF1	Ustilaginaceae	0.251	1.36E-03	5313	ZBTB48	Comamonadaceae	0.466	3.72E-04
1162	CTSV	Kytococcaceae	0.261	2.70E-05	2546	LPCAT3	Actinomyces naeslundii	0.468	7.78E-12	3930	RIC3	Actinomyces naeslundii	-0.211	4.64E-03	5314	ZBTB8B	Kytococcaceae	0.206	1.90E-19
1163	CTSZ	Actinomyces naeslundii	0.277	1.50E-19	2547	LPIN1	Ustilaginaceae	0.344	4.44E-08	3931	RIDA	Cutibacterium modestum	-0.648	1.09E-05	5315	ZBTB8B	Pseudomonas oleovorans	-0.272	1.07E-59
1164	CTTN	Oxalobacteraceae	0.235	4.41E-03	2548	LPP	Carnobacteriaceae	0.608	7.03E-05	3932	RIMKLA	Pseudomonas oleovorans	-0.389	1.05E-29	5316	ZC3H12A	Kytococcaceae	0.175	7.79E-05
1165	CTTNBP2	Paucibacter	-0.276	5.49E-03	2549	LPXN	Actinomyces naeslundii	-0.259	7.54E-03	3933	RIMKLB	Actinomyces naeslundii	-0.346	7.15E-04	5317	ZC3H12B	Kytococcaceae	0.287	3.16E-03
1166	CTU2	Escherichia coli	-0.503	6.31E-07	2550	LRCH4	Bradyrhizobium elkanii	-0.576	2.59E-07	3934	RIMOC1	Plantactinospora	0.677	4.37E-03	5318	ZC3H12D	Rhodobacterales	-0.338	9.77E-03
1167	CUBN	Kytococcaceae	0.115	1.78E-05	2551	LRFN4	Escherichia coli	-0.381	5.66E-16	3935	RIMS1	Pseudomonas oleovorans	-0.266	1.18E-17	5319	ZC3H13	Actinomyces naeslundii	-0.034	6.14E-31
1168	CUBN	Pseudomonas oleovorans	-0.411	3.90E-07	2552	LRFN5	Cutibacterium	-0.369	5.88E-04	3936	RIMS2	Kytococcaceae	0.165	5.25E-09	5320	ZC3H18	Cutibacterium modestum	0.635	1.24E-05
1169	CUEDC1	Escherichia coli	-0.395	2.54E-09	2553	LRIF1	Carnobacteriaceae	0.622	3.80E-04	3937	RIMS2	Pseudomonas oleovorans	-0.329	3.30E-11	5321	ZC3HAV1	Actinomyces naeslundii	0.610	1.55E-17
1170	CUL3	Nocardioides	-0.425	3.47E-03	2554	LRP1	Cutibacterium modestum	0.604	5.05E-08	3938	RING1	Actinomyces naeslundii	-0.295	5.15E-45	5322	ZCCHC24	Actinomyces naeslundii	0.229	1.03E-03
1171	CUTA	Ustilaginaceae	-0.402	3.24E-05	2555	LRP1	Deinococcota	0.601	4.38E-03	3939	RING1	Paucibacter	-0.213	2.85E-10	5323	ZCCHC7	Thermaceae	-0.671	9.55E-04
1172	CUX1	Ceratobasidiaceae	-0.445	3.68E-06	2556	LRP2BP	Actinomyces naeslundii	-0.370	7.65E-05	3940	RIPK4	Alcaligenaceae	0.349	2.74E-03	5324	ZCWPW1	Actinomyces naeslundii	-0.300	1.45E-14
1173	CWC25	Actinomyces naeslundii	-0.275	2.47E-25	2557	LRP5	Cutibacterium modestum	0.599	5.89E-05	3941	RIPOR2	Escherichia coli	0.574	9.55E-04	5325	ZDBF2	Escherichia coli	0.320	3.73E-04
1174	CWH43	Sphingobacteriales	0.442	6.69E-03	2558	LRP8	Dolosigranulum pigrum	0.262	8.78E-65	3942	RIPOR3	Actinomyces naeslundii	-0.419	2.42E-08	5326	ZDHHC1	Kytococcaceae	0.234	5.71E-06
1175	CX3CL1	Leotiomycetes	-0.484	3.99E-12	2559	LRPAP1	Dolosigranulum pigrum	-0.503	1.60E-03	3943	RNASE4	Bradyrhizobium elkanii	0.524	3.01E-05	5327	ZDHHC12	Dolosigranulum pigrum	-0.449	8.22E-03
1176	CXADR	Escherichia coli	0.318	1.26E-03	2560	LRPAP1	Ustilaginaceae	-0.331	4.22E-04	3944	RNASE4	Escherichia coli	0.154	2.32E-04	5328	ZDHHC15	Kytococcaceae	0.267	1.32E-03
1177	CXCL10	Bradyrhizobium elkanii	0.546	3.35E-03	2561	LRPPRC	Actinomyces naeslundii	0.384	8.02E-17	3945	RNASE6	Ustilaginaceae	-0.221	5.34E-10	5329	ZDHHC18	Actinomyces naeslundii	-0.478	3.71E-06
1178	CXCL12	Ustilaginaceae	-0.301	1.13E-03	2562	LRRC23	Acinetobacter ursingii	-0.671	6.10E-11	3946	RNASEH2B	Bacillaceae	-0.610	8.06E-04	5330	ZDHHC21	Dolosigranulum pigrum	0.371	2.31E-04
1179	CXCL16	Ustilaginaceae	-0.258	1.27E-04	2563	LRRC3	Ceratobasidiaceae	-0.146	8.76E-03	3947	RNASEL	Halomonadaceae	0.309	5.24E-06	5331	ZDHHC23	Staphylococcus hominis	-0.259	5.70E-03
1180	CXCL2	Actinomyces naeslundii	-0.485	1.12E-10	2564	LRRC3	Dolosigranulum pigrum	-0.586	5.32E-03	3948	RND2	Helotiales	-0.368	9.75E-03	5332	ZDHHC7	Bradyrhizobium elkanii	-0.578	3.33E-03
1181	CXCL5	Kytococcaceae	0.200	8.12E-04	2565	LRRC36	Kytococcaceae	0.202	3.83E-06	3949	RNF11	Escherichia coli	0.295	1.07E-11	5333	ZDHHC8	Bradyrhizobium elkanii	-0.603	2.08E-05
1182	CXCR4	Leptotrichiaceae	0.283	3.42E-03	2566	LRRC37A3	Kytococcaceae	0.156	1.99E-03	3950	RNF11	Paucibacter	0.309	4.15E-05	5334	ZEB2	Escherichia coli	0.622	4.15E-05
1183	CXCR5	Pseudomonas oleovorans	-0.260	2.45E-04	2567	LRRC37B	Dolosigranulum pigrum	0.357	7.28E-07	3951	RNF122	Cutibacterium acnes	0.438	1.93E-03	5335	ZER1	Escherichia coli	-0.135	1.95E-09
1184	CXorf65	Kytococcaceae	0.070	4.61E-03	2568	LRRC39	Actinomyces naeslundii	-0.187	2.50E-06	3952	RNF128	Escherichia coli	0.259	1.37E-07	5336	ZFAND2B	Escherichia coli	-0.306	7.59E-04
1185	CXXC1	Escherichia coli	-0.188	1.49E-06	2569	LRRC4	Thermaceae	0.587	4.62E-04	3953	RNF13	Dolosigranulum	0.318	5.76E-03	5337	ZFAND3	Actinomyces naeslundii	0.477	7.78E-09

1186	CYB561D2	Plantactinospora	-0.567	1.06E-05	2570	LRRC40	Escherichia coli	0.347	7.90E-05	3954	RNF130	Ottowia	-0.545	2.66E-11	5338	ZFAND5	Actinomyces naeslundii	-0.332	2.05E-05
1187	CYB5R1	Bradyrhizobium elkanii	0.498	3.35E-07	2571	LRRC45	Escherichia coli	-0.592	2.17E-12	3955	RNF138	Escherichia coli	0.379	1.74E-28	5339	ZFAND5	Leptotrichiaceae	0.264	2.77E-03
1188	CYB5R3	Actinomyces naeslundii	0.233	1.30E-03	2572	LRRC4C	Pseudomonas oleovorans	-0.338	4.80E-10	3956	RNF149	Hyphomicrobiales	-0.622	6.86E-03	5340	ZFC3H1	Ustilaginaceae	0.338	2.92E-06
1189	CYB5R4	Streptococcaceae	-0.361	3.45E-06	2573	LRRC56	Actinomyces naeslundii	-0.232	1.04E-14	3957	RNF150	Paucibacter	0.486	9.80E-03	5341	ZFP36L2	Leptotrichiaceae	0.067	8.14E-04
1190	CYBRD1	Escherichia coli	0.436	4.15E-12	2574	LRRC63	Pseudomonas oleovorans	-0.237	6.32E-03	3958	RNF166	Bradyrhizobium elkanii	-0.627	9.26E-08	5342	ZFP62	Ustilaginaceae	0.333	3.22E-06
1191	CYC1	Dolosigranulum pigrum	-0.367	8.20E-04	2575	LRRC69	Dolosigranulum pigrum	0.268	1.85E-08	3959	RNF168	Campylobacterales	0.504	1.92E-03	5343	ZFR	Actinomyces naeslundii	0.434	1.54E-05
1192	CYC1	Leotiomycetes	-0.549	2.50E-03	2576	LRRC70	Dolosigranulum pigrum	0.570	8.45E-06	3960	RNF175	Pseudomonas oleovorans	-0.357	4.80E-20	5344	ZFR2	Kytococcaceae	0.238	2.04E-04
1193	CYC1	Paucibacter	-0.487	4.93E-03	2577	LRRC8C	Escherichia coli	0.689	2.25E-03	3961	RNF185	Actinomyces naeslundii	0.362	1.62E-04	5345	ZFY	Debaryomycetaceae	0.636	2.91E-04
1194	CYFIP1	Thermaceae	0.750	4.80E-07	2578	LRRC8E	Staphylococcus saprophyticus	0.399	5.38E-03	3962	RNF2	Halomonadaceae	0.265	1.24E-03	5346	ZFY	Rhodoferax	0.361	6.14E-06
1195	CYP19A1	Kytococcaceae	0.269	5.59E-17	2579	LRRC9	Pseudomonas oleovorans	-0.065	5.70E-08	3963	RNF2	Ustilaginaceae	-0.206	2.24E-03	5347	ZFYVE21	Dolosigranulum pigrum	-0.459	1.48E-06
1196	CYP1B1	Cellvibrionales	0.193	1.12E-03	2580	LRRD1	Pseudomonas oleovorans	-0.279	5.60E-05	3964	RNF207	Actinomyces naeslundii	-0.283	1.61E-17	5348	ZFYVE21	Nostocales	-0.559	1.79E-08
1197	CYP20A1	Thermales	-0.676	8.96E-07	2581	LRRIQ1	Kytococcaceae	0.006	2.23E-05	3965	RNF208	Escherichia coli	-0.308	1.37E-05	5349	ZFYVE26	Paucibacter	-0.287	1.23E-05
1198	CYP24A1	Pseudomonas oleovorans	-0.296	3.13E-27	2582	LRRIQ1	Pseudomonas oleovorans	-0.421	1.25E-12	3966	RNF212B	Escherichia coli	-0.529	1.31E-06	5350	ZFYVE26	Yersiniaceae	-0.583	6.60E-04
1199	CYP27A1	Paucibacter	-0.482	4.34E-13	2583	LRRN4	Pseudomonas oleovorans	-0.297	3.25E-03	3967	RNF215	Actinomyces naeslundii	-0.266	1.57E-12	5351	ZFYVE27	Actinomyces naeslundii	-0.398	4.76E-14
1200	CYP27C1	Kytococcaceae	0.156	1.25E-04	2584	LRRN4	Streptomyces sp. T12	0.323	4.14E-03	3968	RNF216	Escherichia coli	-0.409	1.54E-13	5352	ZFYVE9	Actinomyces naeslundii	0.429	1.70E-06
1201	CYP2A7	Paucibacter	-0.361	4.60E-03	2585	LRRTM3	Kytococcaceae	-0.020	6.48E-06	3969	RNF25	Escherichia coli	-0.435	5.39E-03	5353	ZGPAT	Dolosigranulum pigrum	-0.502	6.59E-07
1202	CYP2C18	Chromatiales	-0.423	5.11E-10	2586	LRSAM1	Escherichia coli	-0.418	2.32E-06	3970	RNF26	Actinomyces naeslundii	0.175	1.26E-12	5354	ZMAT1	Ustilaginaceae	0.298	2.72E-06
1203	CYP2C9	Chromatiales	-0.467	5.49E-03	2587	LSM1	Bradyrhizobium elkanii	0.577	4.29E-03	3971	RNF7	Cutibacterium modestum	-0.780	3.75E-13	5355	ZMIZ2	Ustilaginaceae	-0.046	4.20E-07
1204	CYP2E1	Kytococcaceae	-0.133	2.97E-04	2588	LSM3	Ustilaginaceae	-0.132	4.57E-06	3972	RNFT2	Pseudomonas oleovorans	-0.368	9.79E-10	5356	ZMPSTE24	Actinomyces naeslundii	0.336	3.45E-10
1205	CYP2J2	Lautropia	0.435	8.42E-04	2589	LSM5	Alphaproteobacteria	-0.615	1.35E-03	3973	RNH1	Deinococcota	0.460	2.07E-05	5357	ZMPSTE24	Plantactinospora	0.496	3.31E-03
1206	CYP3A43	Actinomyces naeslundii	-0.196	4.69E-04	2590	LSMEM1	Ustilaginaceae	0.260	3.54E-04	3974	RNH1	Escherichia coli	-0.282	1.47E-18	5358	ZMYM3	Paucibacter	-0.341	2.92E-06
1207	CYP4A11	Rhizobiaceae	0.462	7.75E-03	2591	LSR	Paucibacter	-0.490	7.33E-04	3975	RO60	Escherichia coli	0.504	1.47E-11	5359	ZMYND11	Escherichia coli	0.514	9.11E-15
1208	CYP4B1	Pseudomonas oleovorans	-0.301	2.28E-05	2592	LTB4R	Ustilaginaceae	-0.029	1.80E-29	3976	ROBO1	Escherichia coli	0.651	1.73E-03	5360	ZMYND11	Plantactinospora	0.459	3.79E-06
1209	CYP4F11	Actinomyces naeslundii	0.497	9.17E-12	2593	LTBP4	Bradyrhizobium elkanii	-0.596	1.95E-04	3977	ROBO3	Ustilaginaceae	0.099	1.74E-03	5361	ZMYND8	Actinomyces naeslundii	-0.304	7.68E-11
1210	CYRIB	Colletotrichum lupini	0.471	7.39E-03	2594	LTO1	Actinomyces naeslundii	-0.344	4.56E-11	3978	ROBO4	Paucibacter	-0.285	9.44E-06	5362	ZNF10	Actinomyces naeslundii	-0.255	1.15E-05
1211	CYRIB	Escherichia coli	0.484	2.38E-05	2595	LUC7L	Actinomyces naeslundii	-0.342	2.74E-17	3979	ROR2	Pseudomonas oleovorans	-0.250	3.05E-06	5363	ZNF10	Ustilaginaceae	0.260	4.89E-03
1212	CYTH2	Actinomyces naeslundii	-0.407	4.93E-05	2596	LUC7L2	Acinetobacter ursingii	0.670	4.25E-05	3980	RORA	Rhodobacterales	-0.451	3.78E-03	5364	ZNF101	Actinomyces naeslundii	-0.375	2.17E-12
1213	CYTH4	Paucibacter	-0.169	1.23E-09	2597	LUC7L3	Actinomyces naeslundii	-0.245	1.15E-25	3981	RORB	Pseudomonas oleovorans	-0.340	4.77E-11	5365	ZNF106	Actinomyces naeslundii	0.601	1.19E-07
1214	D2HGDH	Escherichia coli	-0.316	2.34E-04	2598	LVRN	Pseudomonas oleovorans	-0.139	4.64E-05	3982	RP1	Kytococcaceae	0.168	7.67E-04	5366	ZNF121	Actinomyces naeslundii	-0.202	2.53E-17
1215	DAB2	Paucibacter	-0.303	1.08E-03	2599	LY6G5B	Actinomyces naeslundii	-0.233	2.77E-10	3983	RP1L1	Pseudomonas oleovorans	-0.301	1.87E-05	5367	ZNF131	Actinomyces naeslundii	-0.128	2.62E-03
1216	DAB2IP	Escherichia coli	-0.365	2.13E-06	2600	LY86	Escherichia coli	0.534	9.67E-03	3984	RPA1	Actinomyces naeslundii	0.274	5.29E-25	5368	ZNF131	Ustilaginaceae	0.283	9.24E-04
1217	DAO	Kytococcaceae	-0.101	3.85E-05	2601	LY86	Ustilaginaceae	-0.271	5.42E-04	3985	RPAP1	Escherichia coli	-0.283	7.44E-06	5369	ZNF133	Ustilaginaceae	0.255	5.38E-04
1218	DAP	Actinomyces naeslundii	0.204	7.52E-06	2602	LY96	Escherichia coli	0.354	4.00E-06	3986	RPAP2	Ustilaginaceae	0.399	2.30E-05	5370	ZNF135	Dolosigranulum pigrum	0.259	8.76E-04
1219	DAPK3	Escherichia coli	-0.383	1.52E-07	2603	LYG1	Actinomyces naeslundii	-0.229	2.32E-15	3987	RPGR	Actinomyces naeslundii	-0.113	1.74E-11	5371	ZNF138	Dolosigranulum pigrum	0.479	3.41E-03
1220	DARS2	Actinomyces naeslundii	0.521	1.41E-17	2604	LYG2	Brucellaceae	0.431	2.10E-04	3988	RPGRIP1L	Pseudomonas oleovorans	-0.231	2.51E-07	5372	ZNF140	Dolosigranulum pigrum	-0.293	3.73E-07
1221	DAXX	Nostocales	-0.486	1.33E-03	2605	LYG2	Pseudomonas oleovorans	-0.210	1.60E-06	3989	RPH3A	Pseudomonas oleovorans	-0.176	1.00E-07	5373	ZNF142	Escherichia coli	-0.238	4.86E-04
1222	DBI	Bradyrhizobium elkanii	0.649	2.17E-03	2606	LYPD1	Kytococcaceae	0.140	5.45E-03	3990	RPL11	Cutibacterium modestum	-0.692	4.52E-05	5374	ZNF146	Ustilaginaceae	0.389	5.53E-06
1223	DCAF10	Bradyrhizobium elkanii	0.573	1.19E-03	2607	LYPD6	Kytococcaceae	0.158	7.32E-05	3991	RPL22L1	Alphaproteobacteria	-0.577	1.57E-03	5375	ZNF160	Actinomyces naeslundii	-0.255	1.69E-05
1224	DCAF13	Corynebacterium accolens	0.635	9.36E-06	2608	LYPLA1	Escherichia coli	0.445	2.57E-09	3992	RPL24	Acinetobacter ursingii	-0.835	1.13E-13	5376	ZNF165	Dolosigranulum pigrum	0.375	2.74E-03
1225	DCAF4L1	Dolosigranulum pigrum	0.245	5.58E-04	2609	LYRM1	Cutibacterium modestum	-0.661	2.32E-03	3993	RPL26L1	Ustilaginaceae	-0.261	1.32E-04	5377	ZNF18	Actinomyces naeslundii	-0.266	3.46E-10
1226	DCC	Kytococcaceae	0.104	2.72E-07	2610	LYRM7	Escherichia coli	0.283	8.41E-04	3994	RPL30	Paucibacter	0.269	3.83E-12	5378	ZNF18	Ustilaginaceae	0.295	2.98E-07
1227	DCDC2C	Pseudomonas oleovorans	-0.324	7.07E-15	2611	LYSMD3	Escherichia coli	0.333	1.44E-03	3995	RPL31	Paucibacter	0.460	8.37E-25	5379	ZNF180	Leotiomycetes	0.587	4.69E-03
1228	DCHS1	Paucibacter	-0.226	2.30E-04	2612	LYVE1	Paucibacter	-0.316	1.98E-03	3996	RPL32	Cutibacterium modestum	-0.697	6.68E-03	5380	ZNF180	Thermaceae	-0.579	8.61E-03
1229	DCK	Escherichia coli	0.418	9.72E-07	2613	M1AP	Kytococcaceae	0.175	4.29E-06	3997	RPL34	Paucibacter	0.490	6.11E-04	5381	ZNF189	Actinomyces naeslundii	-0.175	4.72E-06
1230	DCLK1	Pseudomonas oleovorans	-0.477	1.70E-03	2614	MACC1	Dolosigranulum pigrum	0.180	1.34E-05	3998	RPL35A	Paucibacter	0.401	1.86E-28	5382	ZNF189	Thermaceae	-0.667	5.40E-05
1231	DCLRE1C	Thermaceae	-0.594	1.18E-03	2615	MACO1	Pseudomonas oleovorans	0.463	5.62E-06	3999	RPL39	Escherichia coli	0.635	3.60E-03	5383	ZNF195	Actinomyces naeslundii	-0.124	1.92E-05
1232	DCP2	Rhizobiaceae	-0.387	8.15E-03	2616	MACROD1	Escherichia coli	-0.201	3.07E-05	4000	RPL6	Cutibacterium modestum	-0.775	4.60E-15	5384	ZNF195	Ustilaginaceae	0.272	1.79E-03
1233	DCPS	Actinomyces naeslundii	-0.207	7.11E-80	2617	MADCAM1	Ustilaginaceae	0.271	1.60E-03	4001	RPL9	Paucibacter	0.354	3.44E-03	5385	ZNF205	Escherichia coli	-0.254	2.31E-19
1234	DCST2	Actinomyces naeslundii	-0.139	1.79E-04	2618	MAFG	Escherichia coli	-0.552	6.84E-09	4002	RPLP2	Chromatiales	0.428	9.40E-05	5386	ZNF211	Actinomyces naeslundii	-0.179	1.98E-05
1235	DCST2	Ustilaginaceae	0.076	2.65E-20	2619	MAFK	Kytococcaceae	0.346	7.00E-08	4003	RPN1	Actinomyces naeslundii	0.332	7.53E-36	5387	ZNF215	Pseudomonas oleovorans	-0.353	2.93E-03
1236	DCT	Gemella	0.427	3.42E-05	2620	MAGED1	Actinomyces naeslundii	0.249	4.42E-04	4004	RPN2	Actinomyces naeslundii	0.519	1.41E-08	5388	ZNF217	Micromonospora	0.599	4.46E-04
1237	DCUN1D4	Actinomyces naeslundii	-0.231	5.51E-06	2621	MAGI2	Pseudomonas oleovorans	-0.332	3.66E-07	4005	RPS12	Paucibacter	0.302	1.85E-11	5389	ZNF219	Dolosigranulum	-0.427	7.66E-04
1238	DCX	Pseudomonas oleovorans	-0.388	2.14E-05	2622	MAGIX	Escherichia coli	-0.396	1.19E-03	4006	RPS13	Cutibacterium modestum	-0.686	2.54E-04	5390	ZNF221	Carnobacteriaceae	0.654	2.76E-05
1239	DDB1	Actinomyces naeslundii	0.441	2.83E-09	2623	MAGT1	Actinomyces naeslundii	0.398	2.01E-16	4007	RPS14	Cutibacterium modestum	-0.756	4.41E-04	5391	ZNF224	Actinomyces naeslundii	-0.217	1.23E-07

1240	DDB2	Escherichia coli	-0.471	1.10E-03	2624	MAK	Pseudomonas oleovorans	-0.416	1.39E-07	4008	RPS23	Campylobacterales	0.366	8.55E-03	5392	ZNF226	Ustilaginaceae	0.183	8.56E-05
1241	DDIT3	Enterobacterales	-0.600	2.38E-03	2625	MAK16	Dolosigranulum pigrum	0.227	2.86E-04	4009	RPS23	Ustilaginaceae	-0.375	1.19E-05	5393	ZNF23	Ustilaginaceae	0.152	2.49E-08
1242	DDOST	Actinomyces naeslundii	0.340	7.63E-26	2626	MALL	Thermales	0.611	1.17E-04	4010	RPS24	Alphaproteobacteria	-0.609	4.36E-11	5394	ZNF239	Halomonadaceae	-0.239	1.27E-03
1243	DDX21	Kytococcaceae	0.223	1.61E-05	2627	MALRD1	Kytococcaceae	0.209	4.88E-06	4011	RPS24	Bacillaceae	-0.565	1.48E-09	5395	ZNF251	Ustilaginaceae	-0.128	8.53E-09
1244	DDX25	Pseudomonas oleovorans	-0.268	4.26E-03	2628	MALRD1	Pseudomonas oleovorans	-0.268	1.71E-20	4012	RPS24	Paucibacter	0.533	1.13E-32	5396	ZNF254	Ustilaginaceae	0.430	8.85E-05
1245	DDX3Y	Debaryomycetaceae	0.656	1.22E-05	2629	MALT1	Actinomyces naeslundii	-0.118	5.57E-33	4013	RPS26	Acinetobacter ursingii	-0.723	9.92E-13	5397	ZNF26	Actinomyces naeslundii	-0.235	7.41E-06
1246	DDX3Y	Rhodoferax	0.251	1.89E-04	2630	MALT1	Ustilaginaceae	0.241	4.97E-06	4014	RPS27	Paucibacter	0.349	3.96E-03	5398	ZNF263	Actinomyces naeslundii	-0.404	1.04E-11
1247	DDX46	Ustilaginaceae	0.372	6.11E-04	2631	MAN1A1	Escherichia coli	0.453	2.47E-07	4015	RPS27A	Paucibacter	0.303	1.97E-03	5399	ZNF263	Ustilaginaceae	0.084	3.78E-03
1248	DDX5	Actinomyces naeslundii	0.042	5.11E-08	2632	MAN1B1	Escherichia coli	-0.506	1.31E-19	4016	RPS27L	Cutibacterium modestum	-0.675	9.34E-05	5400	ZNF266	Actinomyces naeslundii	-0.359	2.60E-12
1249	DDX5	Ustilaginaceae	0.389	4.02E-04	2633	MAN1C1	Streptococcaceae	-0.346	3.22E-03	4017	RPS3A	Paucibacter	0.460	3.36E-05	5401	ZNF266	Ustilaginaceae	0.184	3.46E-03
1250	DDX51	Actinomyces naeslundii	-0.291	5.41E-68	2634	MAN2A1	Escherichia coli	0.437	2.53E-04	4018	RPS4X	Cutibacterium modestum	-0.706	7.68E-11	5402	ZNF268	Bradyrhizobium elkanii	0.692	1.04E-11
1251	DDX54	Alphaproteobacteria	0.483	7.42E-04	2635	MAN2A2	Ustilaginaceae	0.122	2.74E-09	4019	RPS4Y1	Debaryomycetaceae	0.645	9.05E-06	5403	ZNF281	Sphingomonadaceae	-0.512	3.86E-03
1252	DDX54	Ceratobasidiaceae	-0.408	3.77E-06	2636	MAN2B1	Alphaproteobacteria	0.566	1.00E-04	4020	RPS5	Ustilaginaceae	-0.394	6.81E-04	5404	ZNF284	Rhizobiaceae	-0.485	3.33E-03
1253	DEAF1	Bradyrhizobium elkanii	-0.549	1.38E-04	2637	MAN2B2	Actinomyces naeslundii	0.437	2.93E-08	4021	RPS6KA2	Staphylococcus hominis	0.382	3.13E-11	5405	ZNF287	Pseudomonas oleovorans	-0.425	7.30E-07
1254	DECR2	Enterobacterales	-0.461	2.21E-03	2638	MANBAL	Kytococcaceae	-0.379	7.25E-03	4022	RPS6KA2	Streptococcaceae	-0.450	1.97E-13	5406	ZNF300	Actinomyces naeslundii	-0.411	9.84E-10
1255	DEGS1	Actinomyces naeslundii	0.159	8.17E-03	2639	MAOA	Actinomyces naeslundii	0.380	3.63E-07	4023	RPS6KB2	Escherichia coli	-0.507	4.05E-07	5407	ZNF302	Ustilaginaceae	0.428	1.64E-15
1256	DEGS1	Escherichia coli	0.520	1.97E-03	2640	MAP1A	Pseudomonas oleovorans	-0.475	2.76E-04	4024	RPS7	Cutibacterium modestum	-0.754	4.12E-03	5408	ZNF311	Dolosigranulum pigrum	0.289	2.63E-05
1257	DENND1A	Escherichia coli	-0.354	8.02E-07	2641	MAP2K1	Escherichia coli	0.435	3.15E-03	4025	RPTOR	Deinococcota	0.588	3.06E-03	5409	ZNF316	Actinomyces naeslundii	-0.351	6.24E-10
1258	DENND1B	Alphaproteobacteria	-0.472	7.43E-04	2642	MAP2K2	Escherichia coli	-0.393	3.09E-10	4026	RPUSD1	Escherichia coli	-0.276	1.34E-05	5410	ZNF337	Actinomyces naeslundii	-0.134	2.78E-32
1259	DENND4A	Schlegelella aquatica	0.356	1.47E-06	2643	MAP2K5	Escherichia coli	-0.540	6.88E-05	4027	RRAD	Escherichia coli	-0.296	9.20E-03	5411	ZNF37A	Ustilaginaceae	0.429	8.06E-07
1260	DENND4C	Escherichia coli	0.571	2.42E-08	2644	MAP2K7	Bradyrhizobium elkanii	-0.452	1.48E-04	4028	RRAD	Ustilaginaceae	0.135	1.46E-03	5412	ZNF385B	Actinomyces naeslundii	-0.297	2.23E-04
1261	DENND5A	Thermales	0.688	3.58E-04	2645	MAP2K7	Deinococcota	0.428	2.43E-12	4029	RRAGB	Actinomyces naeslundii	-0.351	6.08E-03	5413	ZNF385D	Pseudomonas oleovorans	-0.287	7.61E-04
1262	DENND6A	Escherichia coli	0.395	2.41E-06	2646	MAP3K10	Escherichia coli	-0.203	5.07E-03	4030	RRAS	Nocardioides	0.441	8.44E-04	5414	ZNF394	Actinomyces naeslundii	-0.398	5.25E-06
1263	DEPDC1	Brucellaceae	0.491	9.77E-03	2647	MAP3K11	Deinococcota	0.396	6.57E-07	4031	RRM1	Actinomyces naeslundii	0.620	3.70E-12	5415	ZNF398	Methylorubrum	-0.497	3.02E-03
1264	DEPDC5	Bradyrhizobium elkanii	-0.515	1.29E-03	2648	MAP3K11	Escherichia coli	-0.258	8.00E-20	4032	RRP1	Escherichia coli	-0.444	3.31E-05	5416	ZNF404	Actinomyces naeslundii	-0.293	9.15E-05
1265	DEPDC5	Cutibacterium modestum	0.582	3.79E-05	2649	MAP3K14	Bradyrhizobium elkanii	-0.568	1.12E-20	4033	RRP12	Kytococcaceae	0.190	6.47E-14	5417	ZNF407	Cutibacterium modestum	0.700	1.49E-07
1266	DEXI	Fusobacterium	-0.195	5.58E-10	2650	MAP3K19	Pseudomonas oleovorans	-0.194	3.28E-04	4034	RRS1	Kytococcaceae	0.185	3.00E-05	5418	ZNF414	Escherichia coli	-0.271	2.12E-04
1267	DFFA	Bradyrhizobium elkanii	0.655	2.22E-04	2651	MAP3K9	Escherichia coli	-0.615	2.70E-03	4035	RSBN1L	Phyllobacteriaceae	0.514	1.67E-05	5419	ZNF419	Ustilaginaceae	0.376	3.12E-12
1268	DFFA	Ottowia	-0.662	7.10E-03	2652	MAP6	Pseudomonas oleovorans	-0.302	4.76E-04	4036	RSL24D1	Cutibacterium modestum	-0.629	2.92E-10	5420	ZNF432	Actinomyces naeslundii	-0.177	3.40E-06
1269	DGCR6	Escherichia coli	-0.294	1.16E-04	2653	MAP7	Escherichia coli	0.643	1.28E-53	4037	RSPH1	Kytococcaceae	0.077	2.70E-08	5421	ZNF433	Dolosigranulum pigrum	0.263	9.64E-03
1270	DGCR6L	Leotiomycetes	-0.455	6.93E-17	2654	MAP7D1	Paucibacter	-0.187	2.14E-39	4038	RSPH4A	Ustilaginaceae	0.334	5.06E-04	5422	ZNF439	Dolosigranulum pigrum	0.251	2.03E-03
1271	DGKA	Ottowia	0.519	1.72E-04	2655	MAPK1	Actinomyces naeslundii	0.560	4.61E-37	4039	RSPO4	Ustilaginaceae	0.088	7.88E-08	5423	ZNF44	Thermaceae	-0.571	5.64E-04
1272	DGKB	Pasteurellales	0.448	7.93E-05	2656	MAPK15	Kytococcaceae	0.212	1.16E-12	4040	RSPRY1	Pseudomonas oleovorans	0.532	8.29E-06	5424	ZNF44	Ustilaginaceae	0.308	1.80E-05
1273	DGKI	Pseudomonas oleovorans	-0.255	8.51E-31	2657	MAPK1IP1L	Rhizobiaceae	-0.456	1.20E-03	4041	RSRC1	Bradyrhizobium elkanii	0.573	1.11E-03	5425	ZNF440	Ustilaginaceae	0.402	3.32E-07
1274	DGKZ	Escherichia coli	-0.229	1.56E-07	2658	MAPK3	Leotiomycetes	-0.542	1.34E-03	4042	RSRC2	Actinomyces naeslundii	-0.066	1.64E-10	5426	ZNF444	Escherichia coli	-0.422	1.66E-04
1275	DHH	Pseudomonas oleovorans	-0.383	5.84E-11	2659	MAPK3	Ustilaginaceae	-0.248	3.85E-03	4043	RSU1	Actinomyces naeslundii	0.256	5.69E-05	5427	ZNF445	Bradyrhizobium elkanii	-0.563	2.57E-03
1276	DHRS1	Actinomyces naeslundii	-0.362	3.83E-34	2660	MAPK6	Staphylococcus hominis	-0.336	2.79E-05	4044	RTCB	Actinomyces naeslundii	0.395	1.44E-10	5428	ZNF45	Sordariales	-0.485	8.14E-04
1277	DHRS12	Actinomyces naeslundii	-0.332	2.81E-06	2661	MAPK8IP1	Dolosigranulum pigrum	-0.457	3.09E-03	4045	RTN3	Ustilaginaceae	0.060	9.52E-09	5429	ZNF454	Pseudomonas oleovorans	-0.196	3.21E-07
1278	DHRS4L2	Empedobacter	0.143	3.16E-03	2662	MAPK8IP2	Escherichia coli	-0.447	4.58E-05	4046	RTN4IP1	Bradyrhizobium elkanii	0.692	3.23E-03	5430	ZNF460	Sphingobacterium	0.641	8.30E-05
1279	DHRS7	Actinomyces naeslundii	0.325	6.00E-06	2663	MAPKAPK2	Escherichia coli	-0.310	1.13E-03	4047	RUFY4	Dolosigranulum pigrum	0.293	3.11E-04	5431	ZNF462	Pseudomonas oleovorans	-0.369	1.73E-06
1280	DHRS7B	Rhodobacterales	0.405	5.32E-22	2664	MAPRE3	Actinomyces naeslundii	-0.413	7.63E-20	4048	RUNDC3B	Escherichia coli	0.347	2.38E-04	5432	ZNF467	Bradyrhizobium elkanii	-0.513	1.21E-03
1281	DHX30	Deinococcota	0.476	4.44E-04	2665	MARCHF3	Saccharomycetales	-0.360	8.28E-03	4049	RUSC2	Corynebacterium tuberculostea	0.373	2.25E-04	5433	ZNF480	Gardnerella	0.598	2.31E-05
1282	DHX30	Escherichia coli	-0.338	4.65E-13	2666	MARCHF7	Escherichia coli	0.337	5.55E-04	4050	RWDD3	Actinomyces naeslundii	-0.409	2.89E-04	5434	ZNF487	Actinomyces naeslundii	-0.180	5.63E-06
1283	DHX37	Escherichia coli	-0.258	2.48E-04	2667	MARCHF9	Actinomyces naeslundii	-0.566	9.91E-08	4051	RXRA	Dolosigranulum	-0.424	9.27E-03	5435	ZNF490	Veillonella parvula	0.315	4.75E-08
1284	DHX37	Kytococcaceae	0.348	1.26E-04	2668	MARCKS	Chromatiales	0.467	4.04E-03	4052	RXRG	Veillonellaceae	0.330	4.45E-03	5436	ZNF500	Escherichia coli	-0.356	4.36E-03
1285	DHX38	Bradyrhizobium elkanii	-0.500	3.29E-04	2669	MARCKS	Escherichia coli	0.594	8.03E-15	4053	RYR2	Pseudomonas oleovorans	-0.359	1.88E-19	5437	ZNF511	Leptotrichiaceae	0.053	1.02E-04
1286	DHX38	Escherichia coli	-0.160	3.25E-08	2670	MARCO	Actinomyces naeslundii	0.345	4.80E-33	4054	RYR3	Pseudomonas oleovorans	-0.369	1.64E-09	5438	ZNF514	Actinomyces naeslundii	-0.219	1.68E-05
1287	DIABLO	Actinomyces naeslundii	0.456	8.71E-05	2671	MARK2	Bradyrhizobium elkanii	-0.482	4.80E-06	4055	S100A13	Actinomyces naeslundii	-0.525	2.82E-09	5439	ZNF514	Ustilaginaceae	0.162	4.19E-07
1288	DIAPH1	Dolosigranulum pigrum	-0.397	6.92E-04	2672	MARK2	Escherichia coli	-0.198	1.16E-04	4056	S100A6	Methyloversatilis sp. RAC08	-0.478	1.60E-03	5440	ZNF517	Escherichia coli	-0.345	6.62E-03
1289	DIAPH3	Pseudomonas oleovorans	-0.324	5.78E-38	2673	MARK3	Actinomyces naeslundii	-0.136	3.43E-12	4057	S100PBP	Actinomyces naeslundii	-0.363	1.45E-09	5441	ZNF518A	Actinomyces naeslundii	-0.043	6.75E-03
1290	DIPK2A	Escherichia coli	0.514	2.57E-06	2674	MARS2	Dolosigranulum pigrum	-0.418	5.85E-05	4058	S1PR1	Ustilaginaceae	-0.285	1.05E-05	5442	ZNF518A	Ustilaginaceae	0.281	1.94E-03
1291	DIS3L2	Escherichia coli	-0.279	5.25E-03	2675	MARVELD2	Actinomyces naeslundii	0.420	6.68E-11	4059	S1PR2	Mycosphaerellales	-0.482	3.72E-04	5443	ZNF524	Escherichia coli	-0.462	2.01E-26
1292	DISC1	Pseudomonas oleovorans	-0.229	5.10E-05	2676	MAS1	Haemophilus	0.386	2.78E-04	4060	SAMD3	Dolosigranulum pigrum	0.455	2.58E-10	5444	ZNF534	Pseudomonas oleovorans	-0.202	7.61E-07
1293	DLC1	Escherichia coli	0.723	3.41E-04	2677	MASP1	Pseudomonas oleovorans	0.483	3.35E-09	4061	SAMD4B	Bradyrhizobium elkanii	-0.445	6.08E-06	5445	ZNF536	Pseudomonas oleovorans	-0.275	4.66E-47

1294	DLD	Actinomyces naeslundii	0.465	8.16E-72	2678	MAST2	Actinomyces naeslundii	-0.232	1.28E-11	4062	SAMD4B	Escherichia coli	-0.297	7.30E-05	5446	ZNF540	Actinomyces naeslundii	-0.227	1.47E-03
1295	DLEC1	Actinomyces naeslundii	-0.314	1.02E-08	2679	MAT2B	Escherichia coli	0.441	3.74E-07	4063	SAMD8	Escherichia coli	0.507	2.47E-04	5447	ZNF540	Ustilaginaceae	0.183	6.93E-07
1296	DLG3	Mycosphaerellales	-0.368	4.71E-03	2680	MAT2B	Ustilaginaceae	-0.189	1.50E-04	4064	SAMD9L	Neisseria	0.532	7.36E-03	5448	ZNF543	Enterococcaceae	0.090	1.95E-03
1297	DLG5	Bradyrhizobium elkanii	-0.621	1.05E-07	2681	MAU2	Actinomyces naeslundii	-0.334	3.83E-13	4065	SAMM50	Actinomyces naeslundii	0.386	5.11E-05	5449	ZNF546	Ustilaginaceae	0.289	8.34E-05
1298	DLGAP1	Pseudomonas oleovorans	-0.275	2.74E-06	2682	MBD3	Escherichia coli	-0.429	6.44E-49	4066	SAP18	Bradyrhizobium elkanii	0.578	4.08E-05	5450	ZNF548	Actinomyces naeslundii	-0.373	7.22E-12
1299	DLGAP2	Dolosigranulum pigrum	0.150	1.94E-05	2683	MBL2	Gemmatales	-0.325	2.93E-04	4067	SAP18	Escherichia coli	0.204	8.70E-05	5451	ZNF558	Actinomyces naeslundii	-0.021	8.81E-04
1300	DLK1	Lautropia	-0.578	2.19E-03	2684	MBNL1	Escherichia coli	0.505	9.60E-07	4068	SAP30BP	Actinomyces naeslundii	-0.161	2.56E-25	5452	ZNF558	Ustilaginaceae	0.363	1.65E-07
1301	DMBT1	Bacillaceae	-0.376	5.95E-05	2685	MBTPS2	Enterobacterales	0.424	1.17E-03	4069	SARS1	Actinomyces naeslundii	0.444	1.30E-10	5453	ZNF559	Ustilaginaceae	0.140	7.09E-06
1302	DMC1	Pseudomonas oleovorans	-0.346	1.36E-03	2686	MC1R	Actinomyces naeslundii	-0.264	2.29E-05	4070	SART1	Escherichia coli	-0.299	3.54E-06	5454	ZNF574	Alphaproteobacteria	0.525	1.21E-05
1303	DMKN	Kytococcaceae	0.108	2.94E-04	2687	MCF2	Pseudomonas oleovorans	-0.192	1.20E-03	4071	SART1	Negativicutes	0.385	1.45E-03	5455	ZNF574	Deinococcota	0.488	9.76E-04
1304	DMTF1	Actinomyces naeslundii	-0.177	8.27E-04	2688	MCF2L2	Kytococcaceae	0.204	1.87E-06	4072	SAT1	Actinomyces naeslundii	-0.412	2.52E-10	5456	ZNF574	Escherichia coli	-0.224	3.08E-05
1305	DMXL2	Paucibacter	-0.133	5.40E-04	2689	MCF2L2	Pseudomonas oleovorans	-0.421	1.84E-11	4073	SATB1	Actinomyces naeslundii	-0.260	1.25E-11	5457	ZNF577	Actinomyces naeslundii	-0.133	1.74E-04
1306	DNAAF1	Kytococcaceae	0.157	4.22E-05	2690	MCHR1	Acinetobacter baumannii	-0.357	6.11E-03	4074	SATB2	Thermodesulfobacteriota	-0.310	1.70E-08	5458	ZNF577	Ustilaginaceae	0.239	5.12E-03
1307	DNAH1	Actinomyces naeslundii	-0.294	2.04E-10	2691	MCM3AP	Halomonadaceae	-0.220	1.92E-05	4075	SAXO1	Pseudomonas oleovorans	-0.337	2.79E-04	5459	ZNF580	Actinomyces naeslundii	-0.380	3.76E-17
1308	DNAH10	Anaerococcus	0.341	4.18E-03	2692	MCMDC2	Lawsonella	-0.270	9.51E-03	4076	SBNO1	Oxalobacteraceae	0.464	4.21E-08	5460	ZNF587	Ustilaginaceae	0.210	3.48E-03
1309	DNAH12	Pseudomonas oleovorans	-0.450	3.43E-14	2693	MCOLN1	Deinococcota	0.598	1.72E-03	4077	SCAF1	Escherichia coli	-0.219	6.88E-05	5461	ZNF587B	Ustilaginaceae	0.307	2.14E-08
1310	DNAH17	Kytococcaceae	0.227	2.48E-06	2694	MCOLN1	Paucibacter	-0.302	4.09E-03	4078	SCAMP1	Escherichia coli	0.667	1.41E-23	5462	ZNF593	Chromatiales	0.333	7.05E-03
1311	DNAH17	Pseudomonas oleovorans	-0.335	1.04E-14	2695	MCOLN2	Pseudomonas oleovorans	-0.428	1.30E-03	4079	SCAND1	Escherichia coli	-0.334	1.75E-07	5463	ZNF595	Ustilaginaceae	0.296	6.86E-04
1312	DNAH2	Kytococcaceae	0.162	2.43E-06	2696	MCOLN3	Streptomyces sp. T12	0.422	3.73E-03	4080	SCAP	Deinococcota	0.446	4.14E-03	5464	ZNF596	Thermaceae	-0.613	3.19E-03
1313	DNAH3	Kytococcaceae	0.037	2.03E-11	2697	MCRIP1	Enterobacterales	-0.524	8.91E-03	4081	SCAP	Escherichia coli	-0.360	1.35E-13	5465	ZNF596	Ustilaginaceae	0.292	1.06E-07
1314	DNAH3	Pseudomonas oleovorans	-0.382	3.58E-29	2698	MCRIP1	Escherichia coli	-0.480	3.98E-13	4082	SCARB1	Paucibacter	-0.561	1.83E-03	5466	ZNF598	Escherichia coli	-0.182	9.99E-04
1315	DNAH7	Kytococcaceae	0.171	8.76E-05	2699	MCRIP2	Enterobacterales	-0.610	4.37E-04	4083	SCART1	Actinomyces naeslundii	-0.256	1.68E-03	5467	ZNF605	Ustilaginaceae	0.331	1.26E-06
1316	DNAH7	Pseudomonas oleovorans	-0.300	4.85E-09	2700	MCRIP2	Escherichia coli	-0.444	6.81E-13	4084	SCIMP	Thermaceae	0.628	7.75E-09	5468	ZNF622	Ustilaginaceae	-0.129	3.53E-03
1317	DNAH8	Pseudomonas oleovorans	-0.400	1.68E-18	2701	MDFIC	Escherichia coli	0.387	6.78E-09	4085	SCIN	Kytococcaceae	0.123	1.31E-04	5469	ZNF623	Methyloversatilis sp. RAC08	0.497	2.11E-04
1318	DNAH9	Kytococcaceae	0.208	1.63E-06	2702	MDGA2	Pseudomonas oleovorans	-0.504	1.12E-08	4086	SCIN	Pseudomonas oleovorans	-0.344	3.31E-07	5470	ZNF624	Escherichia coli	0.344	3.07E-03
1319	DNAH9	Pseudomonas oleovorans	-0.403	5.73E-26	2703	MDH1	Bradyrhizobium elkanii	0.670	1.14E-03	4087	SCMH1	Actinomyces naeslundii	-0.245	1.87E-69	5471	ZNF628	Achromobacter xylosoxidans	-0.632	1.14E-08
1320	DNAI7	Pseudomonas oleovorans	-0.397	5.72E-05	2704	MDH1B	Dolosigranulum pigrum	0.260	1.05E-07	4088	SCML1	Actinomyces naeslundii	-0.277	9.40E-11	5472	ZNF638	Actinomyces naeslundii	0.095	3.63E-17
1321	DNAJA4	Dolosigranulum pigrum	0.206	2.67E-20	2705	MDH2	Actinomyces naeslundii	0.322	3.22E-16	4089	SCN1A	Pseudomonas oleovorans	-0.337	6.00E-11	5473	ZNF639	Escherichia coli	0.245	4.87E-16
1322	DNAJB11	Leptotrichiaceae	-0.288	9.20E-04	2706	MDM4	Actinomyces naeslundii	-0.289	1.21E-06	4090	SCN3A	Pseudomonas oleovorans	-0.427	4.93E-08	5474	ZNF654	Bradyrhizobium elkanii	0.411	4.98E-03
1323	DNAJB13	Paucibacter	0.490	9.45E-03	2707	ME2	Actinomyces naeslundii	0.318	2.38E-33	4091	SCN3B	Pseudomonas oleovorans	-0.403	8.12E-07	5475	ZNF662	Actinomyces naeslundii	-0.187	3.71E-03
1324	DNAJB14	Escherichia coli	0.540	4.73E-142	2708	ME3	Actinomyces naeslundii	-0.355	5.51E-09	4092	SCN5A	Kytococcaceae	0.258	6.42E-08	5476	ZNF664	Actinomyces naeslundii	0.333	1.26E-34
1325	DNAJB2	Enterobacterales	-0.492	1.19E-03	2709	MED1	Chromatiales	-0.309	7.16E-03	4093	SCO2	Nocardioides	0.350	5.58E-03	5477	ZNF668	Escherichia coli	-0.300	8.21E-03
1326	DNAJB2	Escherichia coli	-0.467	1.43E-10	2710	MED12	Cutibacterium modestum	0.737	1.02E-10	4094	SCOC	Cutibacterium modestum	-0.580	2.94E-07	5478	ZNF676	Pseudomonas oleovorans	-0.337	2.03E-05
1327	DNAJB9	Escherichia coli	0.172	4.03E-04	2711	MED12L	Pseudomonas oleovorans	-0.206	7.77E-07	4095	SCOC	Escherichia coli	0.178	7.57E-07	5479	ZNF677	Dolosigranulum pigrum	0.302	6.55E-13
1328	DNAJC10	Ustilaginaceae	0.298	2.47E-03	2712	MED15	Bradyrhizobium elkanii	-0.673	1.67E-08	4096	SCRG1	Pseudomonas oleovorans	-0.352	9.05E-20	5480	ZNF687	Paucibacter	-0.491	9.31E-04
1329	DNAJC11	Alphaproteobacteria	0.715	1.89E-08	2713	MED16	Deinococcota	0.525	1.14E-05	4097	SCRIB	Escherichia coli	-0.262	1.64E-10	5481	ZNF69	Actinomyces naeslundii	-0.077	6.09E-12
1330	DNAJC11	Yersiniaceae	-0.624	1.46E-03	2714	MED21	Dolosigranulum pigrum	0.487	1.26E-03	4098	SCUBE1	Gordonia bronchialis	0.482	7.43E-03	5482	ZNF691	Ustilaginaceae	0.272	3.55E-31
1331	DNAJC12	Escherichia coli	0.370	1.40E-06	2715	MED21	Leotiomycetes	0.599	8.55E-03	4099	SCUBE3	Pseudomonas oleovorans	-0.467	2.80E-08	5483	ZNF692	Actinomyces naeslundii	-0.482	9.38E-21
1332	DNAJC13	Acinetobacter ursingii	0.804	1.09E-12	2716	MED22	Escherichia coli	-0.368	1.28E-03	4100	SCX	Ustilaginaceae	-0.157	3.56E-19	5484	ZNF7	Actinomyces naeslundii	-0.212	1.54E-08
1333	DNAJC14	Actinomyces naeslundii	0.364	3.25E-10	2717	MED24	Deinococcota	0.519	7.68E-03	4101	SCYL1	Leotiomycetes	-0.545	2.63E-03	5485	ZNF7	Ustilaginaceae	0.218	1.52E-03
1334	DNAJC14	Dolosigranulum pigrum	-0.503	1.09E-06	2718	MED24	Escherichia coli	-0.192	1.98E-07	4102	SDC1	Actinomyces naeslundii	0.269	1.54E-04	5486	ZNF700	Actinomyces naeslundii	-0.112	3.30E-15
1335	DNAJC15	Yersiniaceae	0.630	4.29E-03	2719	MED25	Bradyrhizobium elkanii	-0.632	6.98E-06	4103	SDC2	Escherichia coli	0.349	9.05E-14	5487	ZNF707	Escherichia coli	-0.427	2.66E-03
1336	DNAJC19	Escherichia coli	0.300	1.79E-07	2720	MED25	Escherichia coli	-0.174	8.86E-09	4104	SDC3	Paucibacter	-0.366	1.73E-26	5488	ZNF710	Acinetobacter ursingii	0.699	1.24E-14
1337	DNAJC22	Escherichia coli	-0.532	4.50E-05	2721	MED26	Dolosigranulum pigrum	0.303	4.13E-03	4105	SDCBP	Escherichia coli	0.470	9.88E-09	5489	ZNF710	Dolosigranulum	-0.405	5.43E-10
1338	DNAJC30	Gardnerella	-0.534	1.98E-03	2722	MED4	Alphaproteobacteria	-0.646	1.11E-06	4106	SDCBP	Ustilaginaceae	-0.097	4.65E-03	5490	ZNF711	Actinomyces naeslundii	-0.136	1.69E-03
1339	DNAJC5	Deinococcota	0.526	1.48E-03	2723	MED4	Cutibacterium modestum	-0.693	2.20E-13	4107	SDCCAG8	Gardnerella	0.394	6.28E-04	5491	ZNF711	Dolosigranulum pigrum	0.411	5.25E-03
1340	DNAJC6	Pseudomonas oleovorans	-0.320	1.03E-05	2724	MED9	Oxalobacteraceae	0.485	5.18E-03	4108	SDF4	Deinococcota	0.418	2.66E-03	5492	ZNF721	Ustilaginaceae	0.285	2.64E-04
1341	DNAJC6	Pseudomonas sp. CIP-10	-0.079	2.23E-03	2725	MEF2C	Escherichia coli	0.599	3.60E-04	4109	SDHA	Actinomyces naeslundii	0.278	6.02E-11	5493	ZNF723	Kytococcaceae	0.273	2.92E-04
1342	DNAL1	Pseudomonas oleovorans	-0.258	2.06E-04	2726	MEFV	Thermaceae	0.512	9.75E-03	4110	SDHA	Dolosigranulum pigrum	-0.395	1.38E-08	5494	ZNF730	Kytococcaceae	0.102	1.09E-04
1343	DNASE1	Actinomyces naeslundii	-0.315	2.23E-11	2727	MEGF11	Pseudomonas oleovorans	-0.300	1.05E-10	4111	SDHAF1	Enterobacteriaceae	-0.560	2.54E-04	5495	ZNF730	Pseudomonas oleovorans	-0.279	8.44E-14
1344	DNASE1L3	Paucibacter	-0.416	2.57E-09	2728	MEGF6	Actinomyces naeslundii	-0.375	1.26E-05	4112	SDHAF3	Escherichia coli	0.382	3.94E-04	5496	ZNF75A	Actinomyces naeslundii	-0.288	1.45E-12
1345	DNASE2	Actinomyces naeslundii	0.217	1.91E-04	2729	MEGF8	Deinococcota	0.570	5.46E-03	4113	SDHC	Actinomyces naeslundii	0.325	6.63E-09	5497	ZNF75A	Ustilaginaceae	0.301	2.60E-03
1346	DND1	Escherichia coli	-0.275	2.32E-07	2730	MEGF9	Actinomyces naeslundii	0.295	6.61E-06	4114	SDHC	Dolosigranulum pigrum	-0.286	2.49E-03	5498	ZNF76	Actinomyces naeslundii	-0.345	1.44E-53
1347	DNHD1	Actinomyces naeslundii	-0.129	5.69E-12	2731	MEI1	Kytococcaceae	0.069	7.03E-05	4115	SDK1	Pseudomonas oleovorans	-0.220	1.24E-03	5499	ZNF768	Escherichia coli	-0.315	5.30E-14

1348	DNLZ	Pseudomonas oleovorans	-0.350	8.74E-57	2732	MEI1	Pseudomonas oleovorans	-0.440	2.18E-08	4116	SEC11A	Paucibacter	0.296	6.87E-08	5500	ZNF77	Pseudomonas oleovorans	-0.330	2.02E-03
1349	DNM2	Escherichia coli	-0.187	5.59E-06	2733	MEIS1	Actinomyces naeslundii	-0.371	4.88E-04	4117	SEC11A	Ustilaginaceae	-0.293	6.43E-09	5501	ZNF783	Actinomyces naeslundii	-0.414	2.43E-21
1350	DNM3	Pseudomonas oleovorans	-0.270	7.14E-06	2734	MEIS2	Dolosigranulum pigrum	0.447	2.79E-03	4118	SEC14L1	Thermales	0.592	3.14E-03	5502	ZNF789	Actinomyces naeslundii	-0.271	1.50E-05
1351	DOC2A	Dolosigranulum pigrum	0.247	7.03E-07	2735	MEIS3	Kytococcaceae	0.193	1.06E-04	4119	SEC14L2	Dolosigranulum pigrum	-0.381	1.68E-06	5503	ZNF789	Ustilaginaceae	0.171	1.88E-04
1352	DOCK10	Schlegelella aquatica	0.587	2.56E-03	2736	METRN	Dolosigranulum pigrum	-0.426	5.06E-03	4120	SEC14L4	Bacillaceae	0.256	7.75E-03	5504	ZNF79	Methyloversatilis sp. RAC08	0.393	8.47E-04
1353	DOCK2	Ottowia	0.656	5.11E-15	2737	METTL16	Escherichia coli	-0.423	9.95E-08	4121	SEC14L6	Dolosigranulum pigrum	0.270	3.95E-03	5505	ZNF791	Carnobacteriaceae	0.561	2.87E-03
1354	DOCK3	Pseudomonas oleovorans	-0.450	8.00E-10	2738	METTL25B	Actinomyces naeslundii	-0.388	9.43E-13	4122	SEC22A	Corynebacterium accolens	0.634	5.31E-05	5506	ZNF793	Pseudomonas oleovorans	-0.390	1.23E-03
1355	DOK7	Escherichia coli	-0.592	9.01E-04	2739	METTL27	Ceratobasidiaceae	-0.399	2.34E-03	4123	SEC23B	Actinomyces naeslundii	0.355	2.72E-05	5507	ZNF813	Pucciniomycetes	-0.406	8.83E-03
1356	DOLK	Actinomyces naeslundii	0.463	1.32E-07	2740	METTL3	Ustilaginaceae	0.114	4.63E-12	4124	SEC24D	Actinomyces naeslundii	0.344	1.26E-16	5508	ZNF814	Ustilaginaceae	0.343	2.42E-08
1357	DOT1L	Escherichia coli	-0.194	9.28E-03	2741	MEX3C	Escherichia coli	0.261	1.03E-03	4125	SEC61A1	Escherichia coli	-0.566	7.29E-06	5509	ZNF827	Pseudomonas oleovorans	-0.286	1.98E-05
1358	DPEP2	Cutibacterium acnes	0.418	8.75E-04	2742	MEX3D	Ustilaginaceae	-0.398	2.74E-07	4126	SEC61B	Bradyrhizobium elkanii	0.507	1.20E-05	5510	ZNF83	Actinomyces naeslundii	-0.392	1.23E-09
1359	DPH7	Actinomyces naeslundii	-0.444	2.95E-18	2743	MFAP1	Actinomyces naeslundii	0.480	1.87E-08	4127	SECISBP2L	Escherichia coli	0.428	1.62E-27	5511	ZNF836	Deinococcaceae	0.549	3.64E-06
1360	DPM2	Actinomyces naeslundii	-0.217	1.67E-04	2744	MFAP3	Actinomyces naeslundii	0.559	3.25E-08	4128	SEL1L	Actinomyces naeslundii	0.314	3.74E-27	5512	ZNF837	Bradyrhizobium elkanii	-0.517	5.48E-05
1361	DPM3	Escherichia coli	-0.339	4.27E-05	2745	MFAP3L	Escherichia coli	0.244	8.86E-04	4129	SEL1L3	Thermales	0.628	6.90E-04	5513	ZNF837	Escherichia coli	-0.276	4.17E-08
1362	DPP6	Pseudomonas oleovorans	-0.285	2.84E-86	2746	MFSD13A	Escherichia coli	-0.452	1.37E-03	4130	SELENOF	Escherichia coli	0.170	6.46E-04	5514	ZNF839	Actinomyces naeslundii	-0.180	2.87E-05
1363	DPP7	Escherichia coli	-0.368	1.33E-12	2747	MFSD3	Escherichia coli	-0.527	8.22E-11	4131	SELENOO	Bradyrhizobium elkanii	-0.512	1.63E-05	5515	ZNF839	Ustilaginaceae	0.055	2.18E-03
1364	DPP9	Escherichia coli	-0.259	6.45E-05	2748	MGAM2	Pseudomonas oleovorans	-0.230	2.77E-03	4132	SELENOO	Escherichia coli	-0.151	2.60E-11	5516	ZNF84	Actinomyces naeslundii	-0.148	7.59E-05
1365	DPY19L1	Actinomyces naeslundii	0.246	1.12E-20	2749	MGAT3	Cellvibrionales	0.406	7.81E-09	4133	SELENOP	Actinomyces naeslundii	0.369	2.85E-87	5517	ZNF843	Dolosigranulum pigrum	-0.269	3.55E-04
1366	DPY19L4	Escherichia coli	0.407	1.58E-05	2750	MGAT4B	Corynebacterium tuberculostea	0.197	1.21E-06	4134	SELENOS	Acinetobacter ursingii	-0.712	4.09E-11	5518	ZNF846	Thermaceae	-0.561	1.89E-03
1367	DPY30	Cutibacterium modestum	-0.716	1.29E-07	2751	MGAT4B	Escherichia coli	-0.416	4.21E-45	4135	SELENOW	Acinetobacter ursingii	-0.709	1.01E-11	5519	ZNF85	Pseudomonas oleovorans	-0.274	2.46E-03
1368	DPYSL4	Chitinophagaceae	0.498	3.05E-03	2752	MGAT4C	Pseudomonas oleovorans	-0.375	1.99E-08	4136	SELPLG	Thermaceae	0.546	1.42E-03	5520	ZNF860	Pseudomonas oleovorans	-0.312	1.90E-06
1369	DRAM2	Yersiniaceae	0.477	9.15E-03	2753	MGAT4C	Streptomyces sp. T12	0.408	1.08E-06	4137	SEM1	Cutibacterium modestum	-0.787	3.08E-07	5521	ZNF862	Actinomyces naeslundii	-0.185	5.62E-09
1370	DRC7	Pseudomonas oleovorans	-0.287	1.34E-08	2754	MGAT5	Ottowia	0.441	2.61E-04	4138	SEMA3A	Streptomyces sp. T12	0.406	2.14E-03	5522	ZNF865	Bradyrhizobium elkanii	-0.561	2.67E-03
1371	DRG2	Halomonadaceae	-0.267	4.09E-05	2755	MGAT5B	Kytococcaceae	0.228	2.77E-05	4139	SEMA3B	Ustilaginaceae	0.020	1.34E-03	5523	ZNF875	Actinomyces naeslundii	-0.186	2.26E-12
1372	DROSHA	Ustilaginaceae	0.160	1.08E-20	2756	MGLL	Actinomyces naeslundii	0.435	4.75E-12	4140	SEMA3C	Streptomyces sp. T12	0.382	2.07E-03	5524	ZNF90	Pseudomonas oleovorans	-0.226	1.49E-03
1373	DSC2	Actinomyces naeslundii	0.314	5.21E-04	2757	MGP	Methyloversatilis sp. RAC08	-0.349	2.59E-03	4141	SEMA3E	Kytococcaceae	0.181	1.87E-03	5525	ZNF91	Thermaceae	-0.654	3.72E-06
1374	DSG4	Dolosigranulum pigrum	0.220	1.68E-03	2758	MGRN1	Bradyrhizobium elkanii	-0.517	1.88E-05	4142	SEMA4D	Achromobacter	-0.697	1.38E-05	5526	ZNF99	Pseudomonas oleovorans	-0.226	1.56E-03
1375	DSN1	Thermaceae	-0.583	9.31E-05	2759	MGST3	Ustilaginaceae	-0.332	4.58E-05	4143	SEMA4D	Ottowia	0.716	3.16E-03	5527	ZNFX1	Cellvibrionales	0.450	2.89E-04
1376	DSP	Pseudomonas oleovorans	0.480	1.11E-03	2760	MIB1	Escherichia coli	0.376	9.29E-06	4144	SEMA4D	Propionibacteriaceae	0.655	4.09E-03	5528	ZNRF2	Escherichia coli	0.388	1.06E-14
1377	DTNB	Lactococcus	-0.233	8.48E-10	2761	MICA	Actinomyces naeslundii	-0.304	4.06E-09	4145	SEMA4F	Pseudomonas oleovorans	-0.286	2.29E-04	5529	ZPBP	Pseudomonas oleovorans	-0.246	1.02E-07
1378	DTX3	Dolosigranulum pigrum	0.150	3.83E-05	2762	MICAL1	Actinomyces naeslundii	-0.440	4.23E-16	4146	SEMA6A	Paucibacter	-0.170	2.08E-03	5530	ZPLD1	Pseudomonas oleovorans	-0.129	3.94E-04
1379	DTX3L	Actinomyces naeslundii	0.336	2.19E-03	2763	MICAL3	Staphylococcaceae	-0.348	2.86E-03	4147	SEMA6C	Actinomyces naeslundii	-0.342	1.07E-15	5531	ZPR1	Acinetobacter baumannii	0.366	3.72E-07
1380	DUOX1	Pseudomonas oleovorans	-0.438	4.52E-59	2764	MICOS10	Paucibacter	0.358	2.46E-03	4148	SENP3	Nostocales	-0.407	3.32E-03	5532	ZRSR2	Actinomyces naeslundii	-0.146	1.32E-10
1381	DUOX1	Rothia mucilaginosa	0.255	4.11E-17	2765	MICU3	Thermales	-0.659	2.95E-04	4149	SEPTIN1	Achromobacter deleyi	-0.634	2.97E-04	5533	ZSCAN30	Ustilaginaceae	0.270	5.35E-07
1382	DUS1L	Deinococcota	0.496	1.11E-03	2766	MIER1	Escherichia coli	0.563	1.68E-19	4150	SEPTIN10	Kytococcaceae	-0.025	1.25E-06	5534	ZSWIM9	Kitasatosporales	0.480	5.19E-05
1383	DUS3L	Bradyrhizobium elkanii	-0.475	5.20E-04	2767	MIER2	Gordonia bronchialis	0.425	6.45E-05	4151	SEPTIN7	Escherichia coli	0.566	3.01E-32	5535	ZW10	Actinomyces naeslundii	0.675	1.14E-08
1384	DUS3L	Escherichia coli	-0.276	4.47E-07	2768	MIER3	Escherichia coli	0.279	3.81E-05	4152	SEPTIN9	Escherichia coli	-0.253	1.96E-07	5536	ZXDA	Escherichia coli	0.334	4.18E-03
															5537	ZZEF1	Cutibacterium modestum	0.731	1.20E-04

Supplementary Table 12: Individual level host gene-microbiota interaction results by LASSO in NAFL

ID	Gene	Taxa	Coeff	FDR	ID	Gene	Taxa	Coeff	FDR	ID	Gene	Taxa	Coeff	FDR	ID	Gene	Taxa	Coeff	FDR
1	A1CF	Comamonadaceae	-0.264	7.28E-07	496	EDA	Cloacibacterium normanense	0.293	1.12E-04	991	MYO9B	Ralstonia insidiosa	0.321	1.57E-05	1486	SLC25A28	Plantactinospora sp. BC1	0.205	4.39E-03
2	A1CF	Malassezia vespertilionis	0.290	9.56E-04	497	EEF1B2	Lactococcus	0.335	1.48E-04	992	MYOZ1	Brevibacteriaceae	0.032	1.07E-06	1487	SLC25A32	Comamonadaceae	-0.209	2.02E-15
3	AARS1	Nocardioidaceae	-0.371	4.56E-05	498	EEPD1	Sphingomonadales	0.307	1.29E-03	993	MYPOP	Bacillota	-0.546	4.43E-03	1488	SLC25A32	Viruses	0.261	4.06E-05
4	AASDH	Ralstonia insidiosa	-0.200	3.32E-03	499	EFCAB7	Cloacibacterium caeni	-0.236	1.14E-03	994	MZF1	Bacillales	-0.494	7.76E-04	1489	SLC25A40	Ralstonia insidiosa	-0.178	2.71E-03
5	AASDHPPT	Bacillota	0.435	2.94E-03	500	EFNA2	Sphingomonadales	0.439	5.93E-03	995	NAA11	Cellvibrionales	-0.060	2.32E-04	1490	SLC25A40	Sphingomonadales	-0.339	3.23E-07
6	AASDHPPT	Ralstonia insidiosa	-0.311	1.67E-05	501	EFNB1	Campylobacterota	-0.156	2.61E-07	996	NAA30	Comamonadaceae	-0.368	8.52E-05	1491	SLC25A46	Comamonadaceae	-0.303	2.16E-40
7	ABCA12	Streptococcus sanguinis	-0.044	9.16E-06	502	EFNB1	Ralstonia insidiosa	0.139	2.01E-07	997	NAA30	Ralstonia insidiosa	-0.189	2.40E-03	1492	SLC25A46	Ralstonia insidiosa	-0.190	5.19E-28
8	ABCA2	Ralstonia insidiosa	0.177	4.52E-13	503	EFR3A	Ralstonia insidiosa	-0.175	6.65E-05	998	NAA50	Ralstonia insidiosa	-0.311	2.63E-11	1493	SLC26A11	Ralstonia insidiosa	0.287	8.33E-03
9	ABCB8	Sphingomonadales	0.263	2.28E-03	504	EID1	Comamonadaceae	-0.410	5.46E-03	999	NAALADL2	Comamonadaceae	-0.281	1.31E-03	1494	SLC2A13	Sphingomonadales	-0.304	7.68E-03
10	ABCC1	Ralstonia insidiosa	0.344	4.23E-05	505	EID3	Rothia mucilaginosa	-0.356	2.62E-04	1000	NAALADL2	Diaphorobacter	0.379	2.99E-04	1495	SLC2A2	Bacillota	0.405	5.42E-05
11	ABCD1	Bacillota	-0.333	1.17E-03	506	EIF3E	Ralstonia insidiosa	-0.393	1.69E-04	1001	NAB1	Comamonadaceae	-0.306	8.70E-03	1496	SLC2A2	Ralstonia insidiosa	-0.257	2.81E-06
12	ABCD1	Ralstonia insidiosa	0.285	4.61E-03	507	EIF4A2	Ralstonia insidiosa	-0.376	5.40E-05	1002	NAB1	Ralstonia insidiosa	-0.210	2.48E-04	1497	SLC30A5	Ralstonia insidiosa	-0.243	2.59E-06
13	ABCD3	Ralstonia insidiosa	-0.148	3.83E-05	508	EIF4G1	Nocardioidaceae	-0.330	8.60E-06	1003	NABP1	Chromatiaceae	0.170	1.90E-03	1498	SLC30A7	Ralstonia insidiosa	-0.080	1.64E-03
14	ABCD4	Oceanospirillales	-0.411	5.35E-03	509	ELMOD2	Ralstonia insidiosa	-0.196	3.76E-09	1004	NAT14	Shinella	-0.260	2.90E-04	1499	SLC30A9	Ralstonia insidiosa	-0.258	5.65E-05
15	ABCE1	Ralstonia insidiosa	-0.362	9.13E-05	510	ELMOD2	Xanthomonadaceae	-0.251	3.72E-03	1005	NBEAL1	Comamonadaceae	-0.314	4.27E-03	1500	SLC35A1	Ralstonia insidiosa	-0.404	3.59E-06
16	ABCE1	Saccharomycetales	0.441	7.35E-03	511	ELMOD3	Plantactinospora sp. BC1	0.230	2.91E-03	1006	NBEAL2	Ralstonia insidiosa	0.294	6.19E-20	1501	SLC35A3	Comamonadaceae	-0.321	3.09E-03
17	ABHD13	Ralstonia insidiosa	-0.169	3.20E-10	512	ELOVL2	Ralstonia insidiosa	-0.240	8.88E-03	1007	NBPF15	Anaerococcus	-0.349	5.23E-03	1502	SLC35B3	Ralstonia insidiosa	-0.401	1.37E-05
18	ABHD3	Ralstonia insidiosa	-0.196	7.54E-03	513	EMC2	Ralstonia insidiosa	-0.344	6.13E-05	1008	NCBP1	Ralstonia insidiosa	-0.253	1.50E-05	1503	SLC35F5	Ralstonia insidiosa	-0.263	8.01E-04
19	ABHD5	Ralstonia insidiosa	-0.128	2.14E-05	514	EML4	Ralstonia insidiosa	-0.240	3.41E-03	1009	NCKIPSD	Ralstonia insidiosa	0.231	7.40E-03	1504	SLC36A1	Viruses	0.312	5.11E-03
20	ABI3	Ralstonia insidiosa	0.260	2.90E-03	515	ENG	Ralstonia insidiosa	0.201	2.15E-03	1010	NCL	Nocardioidaceae	-0.269	8.85E-03	1505	SLC37A3	Corynebacterium durum	0.131	3.91E-03
21	ABITRAM	Ralstonia insidiosa	-0.203	4.27E-03	516	ENTPD5	Viruses	0.385	3.88E-03	1011	NCOR2	Ralstonia insidiosa	0.303	3.69E-04	1506	SLC38A10	Ralstonia insidiosa	0.144	8.32E-04
22	ABTB1	Bacillota	-0.324	5.91E-03	517	ENY2	Lactococcus	0.283	6.39E-03	1012	NDUFA5	Ralstonia insidiosa	-0.191	1.37E-03	1507	SLC38A10	Sphingomonadales	0.291	1.26E-10
23	ACAN	Cellvibrionales	0.040	9.87E-03	518	EPAS1	Cloacibacterium normanense	0.497	9.73E-03	1013	NDUFAB1	Tepidimonas taiwanensis	0.166	7.65E-03	1508	SLC38A4	Sphingomonadales	-0.355	5.64E-03
24	ACBD5	Sphingomonadales	-0.370	4.66E-14	519	EPC1	Chromatiaceae	0.322	1.90E-04	1014	NDUFAB1	Viruses	-0.374	4.54E-03	1509	SLC38A9	Bacillota	0.594	4.18E-04
25	ACSM5	Azospirillaceae	-0.168	3.40E-03	520	EPHA1	Chromatiaceae	-0.234	2.24E-03	1015	NDUFAF2	Lactococcus	0.356	7.32E-04	1510	SLC38A9	Viruses	0.446	4.79E-03
26	ACTR6	Ralstonia insidiosa	-0.358	1.35E-03	521	EPHA5	Paucibacter	0.307	6.98E-03	1016	NDUFB1	Viruses	-0.382	7.94E-03	1511	SLC39A6	Sphingomonadales	-0.375	9.41E-03
27	ADAMDEC1	Nevskiales	0.241	2.36E-03	522	EPOR	Plantactinospora sp. BC1	0.312	8.86E-03	1017	NECTIN3	Bacillota	0.316	6.04E-04	1512	SLC39A8	Ralstonia insidiosa	-0.342	3.86E-09
28	ADAMTS7	Ralstonia insidiosa	0.347	6.86E-03	523	EPOR	Ralstonia insidiosa	0.311	8.48E-05	1018	NECTIN3	Ralstonia insidiosa	-0.257	6.08E-03	1513	SLC39A8	Sphingomonadales	-0.320	7.91E-03
29	ADAMTSL1	Cloacibacterium caeni	0.213	6.25E-03	524	EPX	Corynebacterium durum	-0.259	8.81E-03	1019	NEDD4	Bacillales	0.474	4.76E-03	1514	SLC39A9	Ralstonia insidiosa	-0.308	2.65E-08
30	ADAT3	Nitrobacteraceae	0.271	2.75E-06	525	ERGIC2	Ralstonia insidiosa	-0.115	7.65E-04	1020	NEK3	Saccharomycetaceae	-0.278	7.79E-03	1515	SLC44A2	Cloacibacterium normanense	0.331	7.11E-04
31	ADGRG6	Bacillota	0.474	1.24E-03	526	ERICH1	Cloacibacterium normanense	-0.230	3.68E-04	1021	NEK7	Ralstonia insidiosa	-0.206	4.06E-03	1516	SLC46A3	Ralstonia insidiosa	-0.352	2.47E-03
32	ADH1A	Bacillus velezensis	-0.293	7.39E-04	527	ERO1A	Ralstonia insidiosa	-0.314	1.15E-03	1022	NELFA	Sphingomonadales	0.355	3.75E-03	1517	SLC47A2	Bacteria	-0.433	1.30E-05
33	ADH1B	Coriobacteriia	-0.249	3.82E-03	528	ERO1A	Saccharomycetales	0.431	6.67E-03	1023	NENF	Viruses	-0.263	8.23E-04	1518	SLC48A1	Malassezia vespertilionis	-0.317	9.68E-05
34	ADIPOR1	Bacillales	0.455	1.04E-03	529	ERO1B	Ralstonia insidiosa	-0.219	8.00E-07	1024	NFAM1	Ralstonia insidiosa	0.244	5.47E-04	1519	SLC4A2	Sphingomonadales	0.295	3.76E-05
35	ADPGK	Corynebacterium durum	0.233	2.97E-03	530	ERVW-1	Corynebacterium durum	-0.107	2.78E-03	1025	NFIC	Ralstonia insidiosa	0.462	3.39E-07	1520	SLC4A4	Ralstonia insidiosa	-0.280	2.13E-05
36	ADSS2	Ralstonia insidiosa	-0.225	5.13E-03	531	ESCO1	Chromatiaceae	0.237	8.53E-11	1026	NFKBIB	Archaea	0.467	7.92E-03	1521	SLC4A4	Sphingomonadales	-0.352	7.24E-03
37	AFDN	Nitrobacteraceae	0.231	7.82E-04	532	ESCO1	Ralstonia insidiosa	-0.199	1.60E-35	1027	NFKBIB	Nitrobacteraceae	0.122	3.80E-03	1522	SLC6A6	Cloacibacterium normanense	0.293	1.30E-03
38	AFF1	Cloacibacterium normanense	0.390	9.57E-04	533	ESPN	Ralstonia insidiosa	0.253	7.94E-05	1028	NFYB	Ralstonia insidiosa	-0.323	2.44E-06	1523	SLC9A7	Ralstonia insidiosa	-0.249	4.27E-17
39	AFF4	Comamonadaceae	-0.252	2.75E-05	534	ESPN	Sphingomonadales	0.356	9.92E-04	1029	NHLRC2	Comamonadaceae	-0.311	1.69E-04	1524	SLC9C1	Streptococcus sanguinis	0.097	8.31E-06
40	AFMID	Bacillota	-0.448	3.80E-03	535	ETAA1	Ralstonia insidiosa	-0.229	4.43E-05	1030	NHLRC3	Ralstonia insidiosa	-0.222	3.01E-03	1525	SLCO1B1	Bacillota	0.343	3.25E-04
41	AFTPH	Comamonadaceae	-0.283	2.27E-22	536	ETFRF1	Comamonadaceae	-0.485	2.37E-09	1031	NHSL2	Cloacibacterium normanense	0.316	4.15E-04	1526	SLCO1B3	Bacillus velezensis	-0.314	4.68E-04
42	AFTPH	Ralstonia insidiosa	-0.155	7.34E-11	537	ETFRF1	Malasseziales	0.128	3.90E-05	1032	NKAP	Cloacibacterium normanense	-0.383	1.25E-09	1527	SLCO1B3-SL	Microbacteriaceae	-0.291	7.68E-03
43	AFTPH	Sphingomonadales	-0.351	9.92E-06	538	ETNK1	Ralstonia insidiosa	-0.070	7.38E-04	1033	NKTR	Cloacibacterium caeni	-0.302	4.08E-03	1528	SLIRP	Viruses	-0.294	9.03E-04
44	AGFG1	Ralstonia insidiosa	-0.118	2.79E-03	539	ETV7	Pichiaceae	0.157	1.04E-05	1034	NKTR	Corynebacterium accolens	0.056	9.39E-05	1529	SLIT1	Staphylococcus hominis	0.391	1.36E-07
45	AGPS	Ralstonia insidiosa	-0.290	2.98E-08	540	EVI5L	Ralstonia insidiosa	0.238	1.33E-20	1035	NLN	Neisseria	0.397	7.76E-05	1530	SLN	Brevibacteriaceae	-0.192	5.30E-05
46	AGRN	Bacillota	-0.333	6.96E-04	541	EVL	Ralstonia insidiosa	0.184	1.68E-03	1036	NMD3	Ralstonia insidiosa	-0.296	3.53E-03	1531	SMAD2	Ralstonia insidiosa	-0.220	7.33E-03
47	AGRN	Ralstonia insidiosa	0.269	1.36E-07	542	EXD3	Ralstonia insidiosa	0.208	4.74E-04	1037	NMD3	Sphingomonadales	-0.434	1.75E-03	1532	SMAD4	Malassezia vespertilionis	0.339	9.49E-03
48	AGRN	Sphingomonadaceae	0.282	1.52E-04	543	EXD3	Sphingomonadales	0.358	7.31E-04	1038	NOD1	Epilithonimonas	-0.308	3.71E-03	1533	SMARCA4	Ralstonia insidiosa	0.323	1.59E-05
49	AIDA	Ralstonia insidiosa	-0.367	8.30E-06	544	EXOC3L2	Ralstonia insidiosa	0.364	4.08E-06	1039	NOL12	Ralstonia insidiosa	0.284	1.84E-03	1534	SMARCA4	Sphingomonadales	0.277	6.53E-04
50	AK4	Ralstonia insidiosa	-0.353	1.48E-04	545	EXOC4	Lactococcus	-0.317	4.68E-03	1040	NOP53	Archaea	0.502	3.25E-05	1535	SMARCAD1	Ralstonia insidiosa	-0.194	2.21E-05
51	AKAP9	Nitrobacteraceae	0.462	3.02E-05	546	EXOC5	Ralstonia insidiosa	-0.208	7.17E-06	1041	NOP53	Sphingomonadaceae	0.357	4.26E-03	1536	SMARCC2	Ralstonia insidiosa	0.270	7.99E-03

52	ALG10	Uroviricota	0.209	2.80E-04	547	EXOSC6	Mycobacteroides	0.225	5.41E-03	1042	NOTCH1	Ralstonia insidiosa	0.310	1.59E-03	1537	SMC4	Microbacteriaceae	-0.428	4.58E-03
53	ALG10B	Comamonadaceae	-0.339	8.47E-04	548	EXTL2	Ralstonia insidiosa	-0.249	3.03E-05	1043	NOXA1	Sphingomonadales	0.344	9.41E-06	1538	SMG6	Viruses	0.292	3.77E-03
54	ALG12	Sordariomycetes	0.261	1.22E-06	549	F11	Archaea	-0.403	1.33E-04	1044	NPC1	Sphaerotilaceae	-0.378	9.23E-03	1539	SMG9	Ralstonia insidiosa	0.301	3.25E-03
55	ALG12	Sphingomonadales	0.350	1.34E-06	550	F11	Corynebacterium tuberculostea	-0.292	2.99E-03	1045	NPM1	Ralstonia insidiosa	-0.365	2.37E-03	1540	SMIM13	Ralstonia insidiosa	-0.199	1.73E-04
56	ALG6	Ralstonia insidiosa	-0.225	6.86E-04	551	F5	Bacillota	0.438	2.00E-04	1046	NPR1	Ralstonia insidiosa	0.195	5.39E-04	1541	SMIM15	Ralstonia insidiosa	-0.122	9.19E-03
57	AMDHD2	Ralstonia insidiosa	0.125	6.74E-03	552	FAAP20	Bacillota	-0.402	8.02E-14	1047	NPTXR	Enterococcaceae	0.184	5.05E-03	1542	SMIM26	Viruses	-0.337	3.17E-03
58	AMDHD2	Sphingomonadaceae	0.306	5.35E-04	553	FAM114A1	Nitrobacteraceae	0.474	5.99E-04	1048	NQO2	Debaryomycetaceae	-0.274	8.52E-03	1543	SMTN	Ralstonia insidiosa	0.300	9.15E-04
59	AMIGO3	Sphingomonadales	0.390	8.65E-03	554	FAM133A	Corynebacterium durum	-0.081	4.00E-05	1049	NR2C1	Ralstonia insidiosa	-0.201	3.15E-03	1544	SNAPC2	Sphingomonadales	0.337	6.34E-04
60	ANAPC2	Ralstonia insidiosa	0.246	4.84E-06	555	FAM136A	Paenibacillaceae	-0.460	1.99E-03	1050	NR2C2AP	Diaphorobacter	-0.401	3.68E-04	1545	SNED1	Ralstonia insidiosa	0.372	8.80E-04
61	ANGPTL8	Glomerellales	0.408	4.35E-03	556	FAM13A	Ralstonia insidiosa	-0.207	9.76E-03	1051	NR5A2	Bacillota	0.411	4.39E-03	1546	SNRPG	Comamonas testosteroni	0.370	7.43E-03
62	ANKDD1A	Brucella	-0.296	5.05E-03	557	FAM168A	Nitrobacteraceae	-0.158	1.80E-06	1052	NR5A2	Malassezia vespertilionis	0.225	4.67E-03	1547	SNX10	Gordonia	-0.227	8.82E-03
63	ANKRD11	Anoxybacillus	-0.444	6.47E-03	558	FAM184B	Veillonella	-0.422	7.43E-03	1053	NRP2	Rothia mucilaginosa	-0.205	9.73E-03	1548	SNX14	Ralstonia insidiosa	-0.457	1.85E-16
64	ANKRD11	Dolosigranulum	0.337	9.32E-04	559	FAM193B	Ralstonia insidiosa	0.320	8.85E-04	1054	NSA2	Lactococcus	0.283	6.27E-03	1549	SNX14	Sphaerotilaceae	-0.375	1.51E-05
65	ANKRD11	Paucibacter	0.439	8.45E-03	560	FAM199X	Comamonadaceae	-0.223	9.12E-04	1055	NSUN5	Bacillota	-0.438	4.81E-03	1550	SNX21	Bacillota	-0.599	7.83E-04
66	ANKRD11	Ralstonia insidiosa	0.424	9.04E-05	561	FAM199X	Ralstonia insidiosa	-0.193	2.46E-04	1056	NSUN5	Sphingomonadales	0.297	1.10E-04	1551	SNX4	Bacillota	0.364	3.71E-12
67	ANKRD13D	Bacillota	-0.452	3.28E-03	562	FAM200B	Ralstonia insidiosa	-0.240	8.54E-03	1057	NTSR1	Acinetobacter baumannii	0.179	1.50E-05	1552	SNX4	Ralstonia insidiosa	-0.237	5.82E-05
68	ANKRD36C	Corynebacterium accolens	0.299	7.40E-03	563	FAM3C	Ralstonia insidiosa	-0.130	8.27E-03	1058	NUBP2	Bacillota	-0.417	5.77E-03	1553	SNX8	Gemella	-0.212	5.17E-36
69	ANKRD36C	Enterobacteriaceae	-0.389	4.17E-03	564	FAM3C	Xanthomonadales	-0.321	7.03E-03	1059	NUCB1	Ralstonia insidiosa	0.076	8.46E-03	1554	SOCS1	Retroviridae	-0.262	4.26E-03
70	ANKRD46	Comamonadaceae	-0.293	3.41E-03	565	FAM83H	Dermacoccaceae	-0.259	4.75E-05	1060	NUCKS1	Comamonadaceae	-0.383	2.73E-06	1555	SOS2	Chromatiaceae	0.280	1.59E-04
71	ANKRD46	Ralstonia insidiosa	-0.241	4.67E-06	566	FAM83H	Sphingomonadales	0.302	2.28E-04	1061	NUDT12	Ralstonia insidiosa	-0.161	6.21E-03	1556	SOS2	Viruses	0.301	2.16E-10
72	ANKRD49	Ralstonia insidiosa	-0.225	1.27E-05	567	FAM8A1	Ralstonia insidiosa	-0.342	4.25E-14	1062	NUDT14	Mycobacteroides	0.303	2.45E-03	1557	SOWAHC	Comamonadaceae	-0.419	5.91E-05
73	ANKS3	Ralstonia insidiosa	0.248	5.12E-03	568	FAM98B	Ralstonia insidiosa	-0.236	1.34E-03	1063	NUDT21	Ralstonia insidiosa	-0.379	1.05E-10	1558	SOX18	Ralstonia insidiosa	0.314	1.67E-04
74	ANO6	Bacillota	0.495	5.76E-03	569	FARP1	Cloacibacterium normanense	0.424	4.22E-03	1064	NUDT21	Sphingomonadales	-0.350	2.01E-03	1559	SP140	Corynebacterium accolens	0.208	5.99E-04
75	ANO8	Ralstonia insidiosa	0.314	6.83E-03	570	FASTK	Bacillota	-0.398	3.54E-08	1065	NUFIP2	Viruses	0.379	9.43E-03	1560	SP2	Plantactinospora sp. BC1	0.316	6.22E-03
76	ANP32A	Mycosphaerellaceae	0.365	8.46E-03	571	FASTK	Gordonia	0.298	3.27E-03	1066	NUP133	Bacillota	0.453	4.83E-03	1561	SPATA2L	Sphingomonadales	0.335	2.56E-08
77	ANP32E	Comamonadaceae	-0.450	6.85E-04	572	FASTK	Sphingomonadales	0.295	1.05E-04	1067	NUP155	Bacillota	0.536	3.42E-05	1562	SPI1	Ralstonia insidiosa	0.321	2.25E-04
78	ANXA5	Nevskiales	0.313	5.88E-03	573	FASTKD2	Bacillota	0.482	5.06E-04	1068	NUP43	Cloacibacterium normanense	-0.296	4.74E-06	1563	SPIN1	Ralstonia insidiosa	-0.160	9.82E-03
79	AOC3	Cloacibacterium normanense	0.185	9.62E-03	574	FASTKD2	Ralstonia insidiosa	-0.296	9.91E-05	1069	NUP54	Ralstonia insidiosa	-0.298	9.12E-06	1564	SPIRE2	Ralstonia insidiosa	0.223	6.46E-03
80	AP1AR	Comamonadaceae	-0.316	7.14E-12	575	FASTKD2	Sphaerotilaceae	-0.375	2.59E-03	1070	NUP88	Archaea	-0.317	9.20E-03	1565	SPPL2A	Sphaerotilaceae	-0.298	7.46E-03
81	AP1AR	Ralstonia insidiosa	-0.146	3.00E-08	576	FASTKD3	Ralstonia insidiosa	-0.327	3.78E-04	1071	NUS1	Ralstonia insidiosa	-0.176	5.22E-06	1566	SPRY4	Enterobacterales	0.322	5.72E-03
82	AP1B1	Ralstonia insidiosa	0.153	5.51E-04	577	FAXDC2	Coriobacteriia	-0.273	2.51E-03	1072	NXT1	Bacillota	-0.304	1.14E-03	1567	SREBF1	Sphingomonadales	0.392	1.39E-05
83	AP1B1	Sordariomycetes	0.340	5.64E-03	578	FBF1	Ralstonia insidiosa	0.286	5.80E-03	1073	NYAP2	Lactobacillus	0.149	4.22E-03	1568	SREK1IP1	Comamonadaceae	-0.502	6.45E-04
84	AP2A2	Ralstonia insidiosa	0.221	7.42E-03	579	FBRS	Ralstonia insidiosa	0.243	2.22E-03	1074	OCIAD1	Ralstonia insidiosa	-0.328	3.55E-04	1569	SRP9	Comamonadaceae	-0.405	3.76E-12
85	AP3M1	Bacillota	0.540	6.00E-04	580	FBRSL1	Ralstonia insidiosa	0.332	2.28E-05	1075	ODF2	Plantactinospora sp. BC1	0.238	5.46E-03	1570	SRP9	Ralstonia insidiosa	-0.267	2.28E-12
86	AP3M1	Ralstonia insidiosa	-0.265	8.33E-05	581	FBXL15	Nitrobacteraceae	0.066	2.78E-04	1076	ODF3B	Bacillales	-0.446	6.80E-05	1571	SRP9	Xanthomonadaceae	-0.310	6.84E-04
87	AP5M1	Ralstonia insidiosa	-0.167	1.43E-25	582	FBXL17	Comamonadaceae	-0.354	2.19E-03	1077	ODF3B	Sphingomonadales	0.271	9.23E-07	1572	SRRM1	Nitrobacteraceae	0.377	9.44E-08
88	APBA3	Sphingomonadales	0.278	1.77E-03	583	FBXL18	Ralstonia insidiosa	0.350	7.76E-04	1078	ODR4	Bacillota	0.467	2.85E-06	1573	SRSF1	Ralstonia insidiosa	-0.167	9.21E-04
89	APBB1IP	Porphyromonadaceae	0.279	2.47E-05	584	FBXL20	Nitrobacteraceae	0.196	4.52E-03	1079	ODR4	Ralstonia insidiosa	-0.228	5.47E-03	1574	SRSF10	Chromatiaceae	0.240	8.62E-03
90	APH1A	Nitrobacteraceae	-0.308	2.78E-04	585	FBXL4	Ralstonia insidiosa	-0.146	3.05E-04	1080	OMA1	Ralstonia insidiosa	-0.351	2.78E-04	1575	SRSF10	Comamonadaceae	-0.338	2.97E-03
91	APLNR	Rothia mucilaginosa	-0.230	6.71E-03	586	FBXL5	Bacillota	0.439	6.70E-03	1081	OR2M3	Gemmatales	-0.366	4.15E-03	1576	SRSF10	Ralstonia insidiosa	-0.223	9.86E-08
92	APOBEC2	Corynebacterium accolens	0.168	2.96E-03	587	FBXL5	Ralstonia insidiosa	-0.409	9.94E-05	1082	OR2M3	Lactobacillus	0.347	5.70E-03	1577	SRXN1	Nitrobacteraceae	-0.217	2.10E-05
93	APOBR	Ralstonia insidiosa	0.247	6.30E-03	588	FBXL8	Nitrobacteraceae	0.186	9.17E-03	1083	OR52H1	Haemophilus	0.221	4.92E-04	1578	SSR1	Ralstonia insidiosa	-0.261	4.29E-05
94	AQP9	Coriobacteriia	-0.293	9.90E-04	589	FBXO17	Bacillota	-0.403	9.73E-03	1084	OR52H1	Thermodesulfobacteriota	-0.260	5.63E-07	1579	SSX2IP	Comamonadaceae	-0.264	2.42E-04
95	ARFGAP1	Bacillota	-0.367	3.06E-03	590	FBXO3	Bacillota	0.387	9.47E-03	1085	OR5AN1	Ottowia	-0.496	1.24E-04	1580	SSX2IP	Sphingomonadales	-0.264	7.89E-03
96	ARFGAP1	Sphingomonadales	0.304	4.40E-04	591	FERMT2	Bacillota	0.451	3.57E-03	1086	ORAI2	Ralstonia insidiosa	0.344	7.10E-03	1581	STAB1	Ralstonia insidiosa	0.230	1.55E-03
97	ARHGAP1	Sordariomycetes	0.307	8.42E-03	592	FERMT2	Malassezia vespertilionis	0.348	1.84E-03	1087	ORC4	Ralstonia insidiosa	-0.304	3.35E-09	1582	STAG2	Bacillota	0.336	1.01E-03
98	ARHGAP12	Comamonadaceae	-0.206	5.72E-18	593	FEZ2	Ralstonia insidiosa	-0.147	6.54E-05	1088	OSGIN1	Burkholderiaceae	0.366	3.38E-05	1583	STAG2	Ralstonia insidiosa	-0.214	2.54E-03
99	ARHGAP12	Ralstonia insidiosa	-0.101	5.11E-07	594	FGD2	Ralstonia insidiosa	0.273	2.58E-04	1089	OSGIN2	Ralstonia insidiosa	-0.315	2.84E-03	1584	STAG2	Sphingomonadales	-0.417	1.27E-04
100	ARHGAP17	Ralstonia insidiosa	0.293	6.87E-03	595	FGF2	Ralstonia insidiosa	-0.279	4.96E-03	1090	OTOG	Ustilaginoidea virens	0.194	7.09E-03	1585	STAU2	Shinella	0.385	7.37E-05
101	ARHGAP29	Microbacteriaceae	-0.314	6.07E-03	596	FGF21	Micromonospora	0.249	8.06E-05	1091	OTUD1	Microbacteriaceae	-0.367	2.79E-03	1586	STK11IP	Sphingomonadales	0.275	2.05E-05
102	ARHGAP29	Ralstonia insidiosa	-0.131	9.02E-03	597	FGFR1OP2	Ralstonia insidiosa	-0.121	5.16E-03	1092	PABPC1	Archaea	0.404	1.07E-03	1587	STK3	Chromatiaceae	0.365	1.90E-03
103	ARHGAP33	Ralstonia insidiosa	0.124	2.65E-03	598	FHIP2A	Comamonadaceae	-0.293	3.91E-03	1093	PABPN1	Archaea	0.476	2.12E-05	1588	STK38L	Ralstonia insidiosa	-0.109	1.59E-03
104	ARHGAP4	Ralstonia insidiosa	0.285	1.37E-04	599	FHIP2A	Microbacteriaceae	-0.370	5.57E-03	1094	PABPN1	Enterobacteriaceae	-0.446	4.87E-03	1589	STPG2	Viruses	0.431	6.31E-05
105	ARHGAP9	Ralstonia insidiosa	0.326	1.61E-03	600	FKTN	Viruses	0.358	9.47E-03	1095	PABPN1	Nitrobacteraceae	0.100	4.84E-06	1590	STRADA	Gordoniaceae	0.266	1.76E-07

106	ARHGEF1	Bacillota	-0.405	1.10E-20	601	FLT4	Ralstonia insidiosa	0.265	2.07E-04	1096	PACS1	Mycobacteroides	-0.181	5.90E-03	1591	STRN3	Comamonadaceae	-0.293	1.76E-03
107	ARHGEF1	Ralstonia insidiosa	0.273	1.26E-21	602	FNDC3A	Bacillota	0.416	2.59E-03	1097	PACS2	Ralstonia insidiosa	0.283	5.99E-09	1592	STT3B	Ralstonia insidiosa	-0.332	7.80E-05
108	ARHGEF18	Ralstonia insidiosa	0.219	3.14E-03	603	FOXK1	Nitrobacteraceae	0.339	8.96E-05	1098	PACS2	Sphingomonadales	0.321	1.43E-03	1593	STX17	Malassezia vespertilionis	0.246	4.52E-03
109	ARID1B	Planctomycetia	0.466	2.80E-04	604	FOXP2	Malassezia vespertilionis	0.318	7.18E-03	1099	PAK4	Bacillota	-0.424	2.87E-28	1594	STX17	Ralstonia insidiosa	-0.222	1.33E-03
110	ARID4B	Corynebacterium accolens	0.397	6.28E-06	605	FOXP4	Ralstonia insidiosa	0.243	1.72E-03	1100	PAK4	Ralstonia insidiosa	0.274	4.04E-16	1595	STX1B	Ralstonia insidiosa	0.140	4.22E-03
111	ARID4B	Nitrobacteraceae	0.471	5.38E-16	606	FPGT	Ralstonia insidiosa	-0.192	4.03E-05	1101	PAK4	Sphingomonadales	0.348	3.51E-19	1596	STX7	Chromatiaceae	0.254	3.51E-03
112	ARIH1	Ralstonia insidiosa	-0.320	5.97E-04	607	FRA10AC1	Cloacibacterium normanense	-0.391	2.44E-05	1102	PANK3	Sphingomonadales	-0.322	4.85E-03	1597	STXBP2	Ralstonia insidiosa	0.251	1.15E-03
113	ARL10	Caulobacterales	-0.288	2.01E-03	608	FRAT1	Colletotrichum lupini	0.257	2.47E-03	1103	PANK4	Ralstonia insidiosa	0.246	1.63E-04	1598	STYX	Comamonadaceae	-0.353	5.18E-03
114	ARL2	Gordonia	0.336	2.38E-05	609	FRG1	Viruses	-0.475	7.66E-03	1104	PAPOLA	Ralstonia insidiosa	-0.290	2.17E-10	1599	SUB1	Ralstonia insidiosa	-0.310	2.99E-03
115	ARL5B	Ralstonia insidiosa	-0.145	7.41E-04	610	FRK	Sphingomonadales	-0.308	1.83E-04	1105	PARD6B	Ceratobasidiaceae	0.381	1.65E-03	1600	SUCO	Ralstonia insidiosa	-0.221	6.44E-05
116	ARL6IP1	Coriobacteriia	-0.331	1.47E-06	611	FRMD4A	Cloacibacterium normanense	0.461	1.76E-03	1106	PARD6B	Comamonadaceae	-0.371	3.34E-03	1601	SUGT1	Ralstonia insidiosa	-0.244	1.47E-03
117	ARL6IP1	Ralstonia insidiosa	-0.383	2.02E-06	612	FRMD5	Streptococcus sanguinis	0.026	9.08E-03	1107	PARP10	Gordonia	0.225	6.47E-03	1602	SUPT5H	Ralstonia insidiosa	0.172	1.63E-03
118	ARL8B	Ralstonia insidiosa	-0.217	1.17E-15	613	FRMPD2	Lactobacillus	0.250	8.98E-03	1108	PARP10	Ralstonia insidiosa	0.167	2.07E-03	1603	SUZ12	Chromatiaceae	0.252	1.19E-07
119	ARMC1	Comamonadaceae	-0.366	1.61E-03	614	FRRS1	Pasteurellaceae	0.386	4.66E-03	1109	PARP10	Sphingomonadales	0.282	2.36E-04	1604	SWSAP1	Bacillota	-0.342	9.59E-03
120	ARMC10	Comamonadaceae	-0.405	7.82E-03	615	FRS2	Viruses	0.314	2.08E-03	1110	PAXBP1	Chromatiaceae	0.254	1.13E-03	1605	SYCE2	Micromonospora	-0.404	1.91E-04
121	ARMC8	Ralstonia insidiosa	-0.188	2.12E-03	616	FSCN1	Ralstonia insidiosa	0.240	6.96E-04	1111	PAXBP1	Microbacteriaceae	-0.298	1.26E-04	1606	SYMPK	Ralstonia insidiosa	0.272	1.94E-07
122	ARMT1	Ralstonia insidiosa	-0.322	1.64E-03	617	FTH1	Actinomycetota	-0.403	4.52E-03	1112	PAXBP1	Ralstonia insidiosa	-0.086	2.34E-04	1607	SYNCRIP	Ralstonia insidiosa	-0.224	7.87E-05
123	ARPC5	Ralstonia insidiosa	-0.235	3.45E-04	618	FUCA1	Agaricomycetes	-0.382	1.13E-06	1113	PCDH12	Cloacibacterium normanense	0.370	1.98E-03	1608	SYPL1	Ralstonia insidiosa	-0.438	7.70E-06
124	ARPP19	Ralstonia insidiosa	-0.134	5.57E-09	619	FUT10	Ralstonia insidiosa	-0.277	5.16E-03	1114	PCDHA12	Corynebacteriaceae	-0.285	8.13E-03	1609	SYVN1	Sphingomonadales	0.317	1.95E-03
125	ASAH2B	Ralstonia insidiosa	-0.201	2.97E-03	620	FUT9	Cellvibrionales	0.457	3.40E-03	1115	PCDHA4	Myxococcota	0.189	1.27E-03	1610	TAB2	Ralstonia insidiosa	-0.219	2.48E-04
126	ASF1A	Ralstonia insidiosa	-0.293	7.65E-03	621	FYTTD1	Ralstonia insidiosa	-0.267	7.72E-03	1116	PCDHA4	Paucibacter	0.223	4.58E-03	1611	TAB3	Ralstonia insidiosa	-0.111	3.87E-03
127	ASMTL	Sphingomonadaceae	0.264	6.82E-04	622	GAA	Ralstonia insidiosa	0.189	5.43E-03	1117	PCDHGA4	Cloacibacterium normanense	0.439	2.43E-03	1612	TAF1C	Sphingomonadales	0.322	4.33E-15
128	ATAD1	Bacillota	0.477	1.63E-03	623	GABBR2	Pichiaceae	0.319	8.44E-03	1118	PCDHGB5	Viruses	0.442	1.81E-03	1613	TAF2	Bacillota	0.459	8.63E-04
129	ATAD1	Ralstonia insidiosa	-0.294	1.78E-06	624	GABPA	Comamonadaceae	-0.392	3.59E-08	1119	PCGF1	Bacillus velezensis	-0.405	4.65E-03	1614	TAF2	Sphingomonadales	-0.260	9.16E-03
130	ATAD1	Sphingomonadales	-0.316	1.74E-03	625	GABRA2	Paucibacter	0.215	8.67E-04	1120	PCNP	Dermacoccaceae	0.409	1.57E-03	1615	TAFAZZIN	Sphingomonadales	0.294	3.40E-03
131	ATAD2B	Cloacibacterium caeni	0.010	5.42E-03	626	GABRQ	Lactobacillus	0.180	4.91E-03	1121	PCNP	Ralstonia insidiosa	-0.154	2.06E-06	1616	TANK	Ralstonia insidiosa	-0.209	3.55E-04
132	ATE1	Sphingomonadales	-0.399	1.67E-03	627	GAK	Ralstonia insidiosa	0.206	9.73E-04	1122	PCNX2	Cloacibacterium caeni	-0.147	1.60E-03	1617	TAOK2	Ralstonia insidiosa	0.323	2.75E-05
133	ATF2	Ralstonia insidiosa	-0.137	1.91E-03	628	GALR3	Ascomycota	-0.383	2.99E-04	1123	PCTP	Ralstonia insidiosa	-0.356	9.20E-03	1618	TAPT1	Comamonadaceae	-0.394	1.55E-03
134	ATG12	Comamonadaceae	-0.296	9.79E-03	629	GAS2L1	Bacillota	-0.382	4.39E-03	1124	PDCD6IP	Bacillota	0.471	2.85E-03	1619	TAPT1	Ralstonia insidiosa	-0.180	4.17E-03
135	ATG12	Ralstonia insidiosa	-0.153	1.97E-03	630	GAS2L1	Ralstonia insidiosa	0.205	6.84E-03	1125	PDCD7	Cloacibacterium normanense	-0.321	1.43E-09	1620	TARDBP	Ralstonia insidiosa	-0.306	8.73E-03
136	ATIC	Mycobacteroides	0.010	1.49E-03	631	GATAD2A	Ralstonia insidiosa	0.315	7.97E-03	1126	PDE4A	Fusobacterium	0.211	8.38E-03	1621	TAS2R20	Bacteria	0.340	2.14E-03
137	ATL2	Ralstonia insidiosa	-0.220	2.00E-03	632	GATAD2A	Sphingomonadales	0.327	3.36E-07	1127	PDE4A	Ralstonia insidiosa	0.248	7.84E-03	1622	TAS2R43	Nevskiales	-0.099	7.84E-03
138	ATN1	Ralstonia insidiosa	0.279	6.76E-03	633	GATD1	Ralstonia insidiosa	0.272	1.60E-03	1128	PDF	Nitrobacteraceae	0.149	1.30E-04	1623	TAS2R50	Nevskiales	-0.427	4.27E-06
139	ATP11B	Ralstonia insidiosa	-0.249	8.53E-03	634	GDAP2	Ralstonia insidiosa	-0.158	3.76E-05	1129	PDHA1	Colletotrichum lupini	0.278	1.88E-03	1624	TBC1D10A	Ralstonia insidiosa	0.276	6.49E-03
140	ATP13A2	Ralstonia insidiosa	0.288	1.99E-04	635	GDF6	Brucella	-0.264	4.04E-04	1130	PDK2	Archaea	-0.310	4.28E-03	1625	TBC1D15	Ralstonia insidiosa	-0.353	9.88E-06
141	ATP2A3	Comamonadaceae	0.332	5.33E-03	636	GEN1	Chromatiaceae	0.290	7.68E-03	1131	PDLIM2	Ralstonia insidiosa	0.214	2.47E-03	1626	TBC1D23	Ralstonia insidiosa	-0.186	2.62E-11
142	ATP2C1	Ralstonia insidiosa	-0.314	3.29E-04	637	GET1	Comamonadaceae	-0.443	9.24E-03	1132	PERP	Ralstonia insidiosa	-0.333	3.76E-03	1627	TBC1D23	Sphingomonadales	-0.282	6.56E-07
143	ATP2C1	Sphingomonadales	-0.464	2.71E-04	638	GET3	Pasteurellaceae	-0.422	4.10E-03	1133	PEX3	Ralstonia insidiosa	-0.228	9.95E-13	1628	TBCA	Viruses	-0.366	7.62E-03
144	ATP6V0C	Ralstonia insidiosa	0.306	1.49E-03	639	GIN1	Ralstonia insidiosa	-0.271	7.29E-04	1134	PGAM5	Bifidobacteriales	-0.408	4.09E-05	1629	TBCK	Ralstonia insidiosa	-0.169	4.44E-04
145	ATP6V1C1	Bacillota	0.522	1.64E-03	640	GIT2	Actinomyces naeslundii	0.400	2.28E-03	1135	PGAM5	Ottowia	-0.288	4.72E-03	1630	TBKBP1	Ralstonia insidiosa	0.216	8.12E-07
146	ATP6V1C2	Anaerococcus	-0.294	4.87E-03	641	GLMN	Ralstonia insidiosa	-0.148	6.84E-03	1136	PGAP6	Sphingomonadaceae	0.188	1.07E-03	1631	TBL1XR1	Comamonadaceae	-0.350	2.57E-09
147	ATP8B2	Colletotrichum lupini	-0.162	6.26E-04	642	GLRX5	Tepidimonas taiwanensis	0.242	6.99E-03	1137	PGM3	Ralstonia insidiosa	-0.274	1.41E-05	1632	TBL1XR1	Microbacteriaceae	-0.320	4.88E-09
148	ATP8B2	Methylobacterium sp. FF17	-0.140	1.90E-04	643	GMFB	Comamonadaceae	-0.286	4.04E-03	1138	PHF20L1	Corynebacterium accolens	0.358	3.57E-03	1633	TBL1XR1	Ralstonia insidiosa	-0.183	6.07E-05
149	ATRX	Bacteria	-0.463	7.86E-03	644	GMFB	Ralstonia insidiosa	-0.221	3.31E-05	1139	PHF3	Bacillota	0.384	9.69E-03	1634	TBL2	Thermaceae	-0.350	1.78E-03
150	ATXN2L	Ralstonia insidiosa	0.247	7.08E-04	645	GNAI1	Comamonadaceae	-0.296	9.28E-05	1140	PHIP	Nitrobacteraceae	0.309	4.32E-03	1635	TBL3	Sphingomonadales	0.285	1.11E-06
151	AZIN1	Ralstonia insidiosa	-0.345	4.03E-03	646	GNAI1	Ralstonia insidiosa	-0.210	6.15E-04	1141	PHKG2	Campylobacteraceae	-0.379	3.26E-05	1636	TBRG1	Enterobacteriaceae	-0.381	4.27E-03
152	B3GALT6	Malassezia vespertilionis	-0.319	1.34E-05	647	GNAI3	Ralstonia insidiosa	-0.317	8.68E-03	1142	PHRF1	Ralstonia insidiosa	0.302	3.42E-04	1637	TBX18	Sordariales	-0.273	7.88E-03
153	B4GALT2	Malassezia vespertilionis	-0.334	5.62E-04	648	GNAQ	Ralstonia insidiosa	-0.094	7.52E-07	1143	PI4K2B	Comamonadaceae	-0.279	6.42E-03	1638	TBXA2R	Ralstonia insidiosa	0.303	4.66E-04
154	BAG1	Viruses	-0.457	6.69E-03	649	GNE	Bacillota	0.430	9.09E-07	1144	PIAS4	Ralstonia insidiosa	0.231	1.66E-03	1639	TCEA1	Comamonadaceae	-0.304	5.63E-06
155	BAG6	Sphingomonadales	0.312	3.69E-03	650	GNG12	Sphingomonadales	-0.308	4.18E-05	1145	PICALM	Bacillota	0.512	3.00E-04	1640	TCEA1	Ralstonia insidiosa	-0.257	1.49E-05
156	BAHCC1	Ralstonia insidiosa	0.270	2.40E-03	651	GNG7	Ralstonia insidiosa	0.267	6.17E-04	1146	PICALM	Ralstonia insidiosa	-0.291	7.73E-03	1641	TCF12	Ralstonia insidiosa	-0.256	8.27E-04
157	BAZ1A	Corynebacterium accolens	0.396	1.35E-04	652	GNMT	Sphingomonadaceae	0.272	4.82E-03	1147	PIGA	Chromatiaceae	0.276	2.57E-03	1642	TCF3	Bacillota	-0.402	5.94E-03
158	BAZ2B	Corynebacterium accolens	0.389	2.20E-03	653	GNPDA1	Gordonia bronchialis	0.363	1.12E-04	1148	PIGK	Bacillota	0.417	6.99E-03	1643	TCN1	Anoxybacillus	0.010	8.28E-04
159	BBS10	Ralstonia insidiosa	-0.300	5.77E-03	654	GOLGA2	Nitrobacteraceae	0.349	6.70E-05	1149	PIGN	Bacillota	0.429	2.27E-06	1644	TCOF1	Ralstonia insidiosa	0.398	3.76E-04

160	BCAM	Ralstonia insidiosa	0.189	2.42E-04	655	GOLGA3	Ralstonia insidiosa	0.365	8.11E-03	1150	PIGN	Sphingomonadales	-0.343	8.91E-04	1645	TCTN3	Nitrobacteraceae	-0.091	1.43E-03
161	BCAT2	Ralstonia insidiosa	0.335	1.98E-12	656	GOLPH3L	Aerococcaceae	0.347	2.70E-05	1151	PIK3CA	Ralstonia insidiosa	-0.191	1.08E-07	1646	TECPR1	Ralstonia insidiosa	0.295	1.56E-07
162	BCKDHA	Ralstonia insidiosa	0.181	3.04E-03	657	GOLT1B	Ralstonia insidiosa	-0.191	4.99E-03	1152	PIK3CA	Sphingomonadales	-0.311	8.03E-03	1647	TECPR1	Sphingomonadales	0.257	4.38E-06
163	BCLAF1	Chromatiaceae	0.213	1.84E-03	658	GON4L	Cloacibacterium caeni	-0.157	4.27E-03	1153	PILRA	Ralstonia insidiosa	0.186	3.71E-03	1648	TECR	Ralstonia insidiosa	0.137	3.71E-03
164	BCLAF1	Microbacteriaceae	-0.382	1.10E-03	659	GOPC	Ralstonia insidiosa	-0.190	2.86E-03	1154	PILRB	Cyanophyceae	0.371	1.66E-03	1649	TEKTIP1	Ralstonia insidiosa	0.353	3.37E-06
165	BCLAF1	Ralstonia insidiosa	-0.187	1.06E-05	660	GPATCH2	Chromatiaceae	0.410	6.37E-03	1155	PILRB	Ralstonia insidiosa	0.311	6.66E-03	1650	TENT2	Ralstonia insidiosa	-0.201	2.46E-05
166	BCR	Ralstonia insidiosa	0.141	8.78E-03	661	GPBP1	Ralstonia insidiosa	-0.357	7.39E-08	1156	PIP5K1C	Ralstonia insidiosa	0.446	1.54E-08	1651	TENT4B	Comamonadaceae	-0.355	7.24E-06
167	BCR	Sphingomonadales	0.339	3.75E-03	662	GPR173	Mycobacteroides	-0.270	3.49E-03	1157	PITPNM2	Ralstonia insidiosa	0.254	5.15E-05	1652	TEX261	Archaea	-0.390	1.05E-03
168	BEX2	Pseudomonas sp. CIP-10	-0.303	4.35E-03	663	GPR89B	Bacteria	0.407	2.53E-03	1158	PITPNM2	Sphingomonadales	0.400	2.20E-12	1653	TEX261	Nitrobacteraceae	-0.142	6.06E-07
169	BICRA	Ralstonia insidiosa	0.282	1.25E-03	664	GRAMD1C	Ralstonia insidiosa	-0.266	6.85E-03	1159	PIWIL1	Lactobacillus	0.186	2.90E-03	1654	TFAM	Ralstonia insidiosa	-0.222	1.07E-03
170	BIRC2	Chromatiaceae	0.112	1.49E-03	665	GRAMD1C	Sphingomonadales	-0.330	1.74E-04	1160	PKD1	Ralstonia insidiosa	0.241	1.51E-07	1655	TFAP4	Mycobacteroides	0.292	3.32E-04
171	BIRC2	Microbacteriaceae	-0.149	6.71E-03	666	GRIA4	Shinella	-0.301	2.26E-03	1161	PLA2G1B	Prevotella	-0.345	2.39E-04	1656	TFE3	Ralstonia insidiosa	0.197	4.50E-03
172	BIRC2	Ralstonia insidiosa	-0.281	8.08E-11	667	GRK2	Ralstonia insidiosa	0.177	4.11E-05	1162	PLAC8L1	Cloacibacterium normanense	-0.253	1.02E-03	1657	TGFB1	Ralstonia insidiosa	0.309	3.92E-05
173	BLID	Cloacibacterium	-0.333	2.38E-03	668	GSDMD	Gordonia	0.311	7.26E-03	1163	PLCB2	Ralstonia insidiosa	0.285	6.58E-05	1658	THAP12	Ralstonia insidiosa	-0.338	7.87E-03
174	BMP6	Mycobacteroides	-0.155	7.44E-03	669	GSDMD	Ottowia	-0.301	4.80E-06	1164	PLCB3	Ralstonia insidiosa	0.268	7.09E-04	1659	THAP7	Ottowia	-0.482	4.46E-03
175	BMP8A	Ralstonia insidiosa	0.283	2.22E-03	670	GTF2A1	Comamonadaceae	-0.270	7.82E-03	1165	PLCE1	Paucibacter	0.214	1.38E-03	1660	THAP7	Sphingomonadales	0.413	1.11E-04
176	BNIP2	Chromatiaceae	0.338	4.20E-07	671	GTF2F1	Ralstonia insidiosa	0.158	1.81E-03	1166	PLD3	Ralstonia insidiosa	0.144	2.64E-05	1661	THOC2	Nitrobacteraceae	0.374	8.67E-03
177	BNIP2	Ralstonia insidiosa	-0.135	9.65E-16	672	GTF3C3	Ralstonia insidiosa	-0.234	1.74E-03	1167	PLEKHF2	Ralstonia insidiosa	-0.170	3.91E-03	1662	THRAP3	Nitrobacteraceae	0.253	7.25E-04
178	BNIP3L	Sphingomonadales	-0.380	7.68E-03	673	GTF3C5	Gordonia	0.259	1.87E-03	1168	PLEKHG5	Ralstonia insidiosa	0.287	1.98E-03	1663	TIGD5	Bacillota	-0.426	9.37E-04
179	BOD1L1	Bacteria	-0.457	5.22E-03	674	GTPBP2	Aerococcaceae	-0.280	4.50E-03	1169	PLG	Cloacibacterium normanense	0.396	3.91E-03	1664	TIMM44	Ralstonia insidiosa	0.252	2.66E-05
180	BOD1L1	Corynebacterium accolens	0.342	9.15E-03	675	GTPBP3	Ralstonia insidiosa	0.260	7.76E-03	1170	PLIN5	Ralstonia insidiosa	0.326	7.42E-06	1665	TINAGL1	Cloacibacterium normanense	0.253	3.73E-06
181	BORCS7	Cloacibacterium normanense	-0.286	9.98E-04	676	GTPBP6	Prevotellaceae	-0.361	7.38E-03	1171	PLOD2	Ralstonia insidiosa	-0.267	3.58E-03	1666	TIPRL	Ralstonia insidiosa	-0.308	6.35E-09
182	BORCS7	Comamonadaceae	-0.342	9.27E-03	677	GTPBP6	Sphingomonadaceae	0.271	6.53E-03	1172	PLPBP	Actinomyces	-0.166	9.24E-03	1667	TIPRL	Saccharomycetales	0.377	5.01E-03
183	BRAT1	Ralstonia insidiosa	0.185	9.47E-03	678	GUF1	Ralstonia insidiosa	-0.356	3.14E-04	1173	PLPBP	Ralstonia insidiosa	-0.270	4.89E-04	1668	TJAP1	Sphingomonadales	0.282	1.17E-03
184	BRD3	Nitrobacteraceae	0.471	7.85E-04	679	H2BC10	Nevskiales	0.385	1.36E-05	1174	PLXND1	Ralstonia insidiosa	0.250	6.53E-03	1669	TLCD2	Ralstonia insidiosa	0.403	1.67E-03
185	BRD4	Ralstonia insidiosa	0.412	5.28E-04	680	H2BC3	Myxococcota	-0.383	6.81E-05	1175	PML	Sphingomonadales	0.327	5.79E-04	1670	TLE3	Planctomycetia	0.375	8.65E-03
186	BRF1	Bacillota	-0.369	5.65E-03	681	H2BC3	Rhodocyclales	-0.369	2.70E-03	1176	PNISR	Corynebacterium accolens	0.037	5.79E-07	1671	TM2D3	Ralstonia insidiosa	-0.289	5.61E-03
187	BRF1	Ralstonia insidiosa	0.212	5.33E-03	682	H2BC7	Archaea	0.316	4.64E-04	1177	PNPLA8	Ralstonia insidiosa	-0.171	1.56E-04	1672	TM4SF18	Shinella	0.352	1.58E-03
188	BRMS1L	Ralstonia insidiosa	-0.228	5.81E-03	683	H4C2	Gemella	-0.237	6.94E-03	1178	PNRC2	Ralstonia insidiosa	-0.378	1.42E-06	1673	TM6SF1	Bacillus velezensis	0.405	9.49E-03
189	BRWD1	Ralstonia insidiosa	-0.216	5.92E-06	684	H4C6	Rothia mucilaginosa	-0.132	4.34E-03	1179	POC1B	Ralstonia insidiosa	-0.246	3.01E-03	1674	TM9SF3	Ralstonia insidiosa	-0.243	8.18E-21
190	BSCL2	Cloacibacterium normanense	0.218	9.13E-09	685	HACD3	Ralstonia insidiosa	-0.380	4.29E-03	1180	POGLUT3	Ralstonia insidiosa	-0.355	1.82E-04	1675	TM9SF3	Sphingomonadales	-0.304	2.20E-07
191	BSCL2	Viruses	0.269	1.24E-03	686	HACE1	Chromatiaceae	0.343	6.21E-04	1181	POLD1	Ralstonia insidiosa	0.189	6.53E-05	1676	TMC6	Ralstonia insidiosa	0.212	2.10E-03
192	BTBD1	Ralstonia insidiosa	-0.253	4.30E-13	687	HAT1	Ralstonia insidiosa	-0.335	1.26E-14	1182	POLE3	Ralstonia insidiosa	-0.348	9.34E-03	1677	TMC8	Ralstonia insidiosa	0.267	5.74E-03
193	BTBD9	Lactococcus	-0.246	6.15E-03	688	HDAC10	Bacillota	-0.412	6.52E-07	1183	POLI	Ralstonia insidiosa	-0.240	1.91E-04	1678	TMCO1	Ralstonia insidiosa	-0.407	2.30E-05
194	BTD	Bacillota	0.481	5.70E-09	689	HDAC10	Ralstonia insidiosa	0.170	4.31E-08	1184	POLM	Sphingomonadales	0.357	9.60E-03	1679	TMED10	Ralstonia insidiosa	-0.355	4.37E-04
195	BTF3L4	Ralstonia insidiosa	-0.284	2.08E-04	690	HDAC10	Sphingomonadales	0.297	9.14E-07	1185	POLR3GL	Caulobacteraceae	0.438	4.66E-09	1680	TMED2	Ralstonia insidiosa	-0.318	1.17E-03
196	BZW1	Ralstonia insidiosa	-0.364	8.23E-07	691	HDAC7	Ralstonia insidiosa	0.291	1.11E-05	1186	POMK	Cloacibacterium normanense	0.336	1.19E-03	1681	TMED5	Ralstonia insidiosa	-0.194	4.77E-03
197	C12orf29	Ralstonia insidiosa	-0.189	5.32E-08	692	HDGFL2	Gordoniaceae	0.195	9.12E-03	1187	POP4	Viruses	-0.216	2.30E-04	1682	TMEM106B	Ralstonia insidiosa	-0.143	3.30E-03
198	C12orf56	Bacteria	-0.334	1.05E-03	693	HDGFL2	Ralstonia insidiosa	0.156	1.08E-12	1188	POT1	Bacillota	0.492	7.77E-04	1683	TMEM131	Bacillota	0.513	2.13E-03
199	C12orf76	Viruses	-0.326	9.33E-03	694	HESX1	Cloacibacterium caeni	-0.388	2.72E-03	1189	POT1	Ralstonia insidiosa	-0.405	7.98E-09	1684	TMEM131L	Archaea	-0.392	9.91E-03
200	C15orf39	Enterobacteriaceae	0.344	2.09E-03	695	HIC1	Ralstonia insidiosa	0.294	4.37E-03	1190	POU2F1	Nitrobacteraceae	0.292	6.32E-03	1685	TMEM132A	Ralstonia insidiosa	0.345	2.16E-03
201	C15orf40	Cloacibacterium normanense	-0.347	5.25E-04	696	HK3	Ralstonia insidiosa	0.191	5.88E-03	1191	POU2F2	Cyanophyceae	0.283	4.69E-03	1686	TMEM132E	Ralstonia insidiosa	0.203	4.75E-03
202	C16orf86	Bacillota	-0.374	1.90E-03	697	HMG20A	Ralstonia insidiosa	-0.285	1.75E-03	1192	POU2F2	Dolosigranulum	0.379	1.08E-03	1687	TMEM135	Comamonadaceae	-0.169	5.94E-05
203	C17orf80	Ralstonia insidiosa	-0.269	1.22E-05	698	HMGB1	Ralstonia insidiosa	-0.167	6.13E-05	1193	POU2F2	Nitrobacteraceae	0.345	2.88E-04	1688	TMEM135	Ralstonia insidiosa	-0.197	2.13E-06
204	C19orf25	Bacillota	-0.315	5.49E-06	699	HMGCLL1	Brucella	-0.249	8.60E-03	1194	POU2F2	Ralstonia insidiosa	0.530	1.38E-08	1689	TMEM143	Gordonia	0.316	1.84E-03
205	C1GALT1	Comamonadaceae	-0.331	4.44E-07	700	HMGN2	Archaea	0.474	1.02E-04	1195	PPCS	Ralstonia insidiosa	-0.361	7.75E-03	1690	TMEM161B	Ralstonia insidiosa	-0.169	9.56E-03
206	C1GALT1	Malassezia vespertilionis	0.341	3.12E-06	701	HMGN4	Ralstonia insidiosa	-0.292	4.54E-03	1196	PPDPF	Ralstonia insidiosa	0.242	8.65E-07	1691	TMEM167A	Ralstonia insidiosa	-0.275	3.61E-03
207	C1GALT1	Ralstonia insidiosa	-0.185	2.73E-09	702	HNRNPA0	Archaea	0.351	3.19E-03	1197	PPDPF	Saccharomycetales	-0.417	1.25E-18	1692	TMEM168	Comamonadaceae	-0.382	1.38E-03
208	C1GALT1C1	Ralstonia insidiosa	-0.412	4.24E-04	703	HNRNPA2B1	Campylobacterota	0.168	2.74E-03	1198	PPFIA1	Viruses	0.367	4.72E-04	1693	TMEM170B	Ralstonia insidiosa	-0.146	5.74E-03
209	C1orf159	Ralstonia insidiosa	0.237	3.76E-06	704	HNRNPC	Ralstonia insidiosa	-0.481	3.00E-06	1199	PPIL2	Sordariomycetes	0.324	4.81E-03	1694	TMEM181	Ralstonia insidiosa	-0.281	8.77E-03
210	C1orf159	Sphingomonadales	0.421	8.23E-05	705	HNRNPH1	Nitrobacteraceae	0.074	2.91E-03	1200	PPM1B	Ralstonia insidiosa	-0.161	4.78E-03	1695	TMEM181	Sphaerotilaceae	-0.323	4.52E-03
211	C1RL	Cutibacterium	0.376	2.25E-03	706	HNRNPH3	Ralstonia insidiosa	-0.254	5.35E-04	1201	PPM1M	Campylobacterota	-0.188	3.02E-03	1696	TMEM186	Spirochaetota	0.412	1.57E-03
212	C1S	Neisseria	0.388	2.19E-04	707	HNRNPM	Archaea	0.494	8.35E-05	1202	PPP1CB	Comamonadaceae	-0.410	5.14E-03	1697	TMEM192	Sphingomonadales	-0.311	2.52E-03
213	C2orf69	Comamonadaceae	-0.301	3.45E-03	708	HOOK1	Ralstonia insidiosa	-0.229	3.72E-03	1203	PPP1CC	Ralstonia insidiosa	-0.245	2.42E-25	1698	TMEM203	Viruses	-0.423	7.72E-03

214	C3orf62	Bacillota	-0.455	9.76E-03	709	HOXA5	Cloacibacterium caeni	0.233	1.13E-05	1204	PPP1CC	Sphingomonadales	-0.376	9.15E-37	1699	TMEM230	Ralstonia insidiosa	-0.354	1.12E-05
215	C4orf48	Deinococcaceae	0.328	6.23E-03	710	HOXB5	Corynebacterium durum	-0.221	1.80E-04	1205	PPP1R11	Nitrobacteraceae	-0.350	1.02E-03	1700	TMEM245	Microbacteriaceae	-0.356	3.30E-03
216	C4orf50	Rhodocyclales	0.177	3.52E-08	711	HPRT1	Coriobacteriia	-0.373	1.81E-04	1206	PPP1R12C	Ralstonia insidiosa	0.257	2.82E-07	1701	TMEM254	Ralstonia insidiosa	-0.397	4.30E-04
217	C5orf15	Malasseziales	0.255	1.10E-09	712	HPS6	Bacillota	-0.240	1.79E-04	1207	PPP1R12C	Sphingomonadales	0.425	1.82E-07	1702	TMEM255B	Ralstonia insidiosa	0.349	4.93E-03
218	C5orf15	Ralstonia insidiosa	-0.130	2.96E-06	713	HRG	Neisseria	0.437	7.29E-03	1208	PPP1R27	Cloacibacterium caeni	0.095	3.93E-03	1703	TMEM263	Ralstonia insidiosa	-0.070	5.13E-03
219	C6orf120	Ralstonia insidiosa	-0.190	4.08E-03	714	HS2ST1	Gordonia	-0.260	1.85E-03	1209	PPP1R37	Bacillota	-0.351	8.17E-16	1704	TMEM33	Ralstonia insidiosa	-0.081	3.69E-03
220	C6orf52	Viruses	-0.357	1.89E-05	715	HS2ST1	Ralstonia insidiosa	-0.211	1.47E-06	1210	PPP1R37	Ralstonia insidiosa	0.208	7.92E-08	1705	TMEM44	Ralstonia insidiosa	0.272	2.32E-03
221	C9orf72	Chromatiaceae	0.237	2.23E-03	716	HS2ST1	Sphingomonadales	-0.325	3.65E-03	1211	PPP1R37	Sphingomonadales	0.276	9.14E-13	1706	TMEM67	Corynebacterium durum	-0.118	8.92E-04
222	C9orf72	Ralstonia insidiosa	-0.211	3.46E-05	717	HSD17B12	Saccharomycetales	0.393	5.82E-03	1212	PPP3CA	Ceratobasidiaceae	0.430	4.88E-05	1707	TMX3	Ralstonia insidiosa	-0.141	6.54E-04
223	C9orf78	Archaea	0.430	5.47E-06	718	HSD17B3	Bacillota	-0.376	1.62E-03	1213	PPP3CA	Comamonadaceae	-0.398	6.38E-05	1708	TNFAIP8L1	Bacillota	-0.357	7.18E-03
224	CABIN1	Ralstonia insidiosa	0.309	4.77E-08	719	HSD3B7	Gordonia	0.300	5.60E-03	1214	PPP4R2	Comamonadaceae	-0.426	2.19E-05	1709	TNFSF10	Malassezia vespertilionis	0.265	1.16E-03
225	CABIN1	Sphingomonadales	0.318	5.79E-03	720	HSPD1	Gordonia	-0.330	8.43E-03	1215	PPP4R3B	Ralstonia insidiosa	-0.319	3.95E-05	1710	TNKS	Ralstonia insidiosa	-0.236	6.92E-04
226	CACTIN	Ralstonia insidiosa	0.174	1.02E-03	721	HSPG2	Cloacibacterium normanense	0.297	3.98E-03	1216	PPP6C	Bacillota	0.359	7.85E-05	1711	TNKS	Viruses	0.337	7.85E-03
227	CAMK2D	Comamonadaceae	-0.318	3.66E-03	722	HSPG2	Ralstonia insidiosa	0.262	3.02E-03	1217	PPP6C	Ralstonia insidiosa	-0.374	4.20E-18	1712	TNKS2	Chromatiaceae	0.333	1.51E-05
228	CAMTA2	Ralstonia insidiosa	0.311	3.87E-03	723	IBTK	Ralstonia insidiosa	-0.266	4.34E-03	1218	PPP6R1	Ralstonia insidiosa	0.195	9.29E-06	1713	TNPO1	Ralstonia insidiosa	-0.205	2.64E-04
229	CAMTA2	Sphingomonadales	0.258	7.18E-03	724	ICAM2	Cloacibacterium normanense	0.316	1.69E-05	1219	PPRC1	Actinomyces	0.191	2.17E-05	1714	TNRC18	Ralstonia insidiosa	0.348	9.54E-05
230	CAND1	Bacillota	0.450	4.05E-05	725	ICE2	Ralstonia insidiosa	-0.255	1.90E-03	1220	PRAG1	Ralstonia insidiosa	0.284	4.94E-03	1715	TNRC6A	Nitrobacteraceae	0.325	1.30E-04
231	CAND1	Sphaerotilaceae	-0.427	5.17E-03	726	IER5L	Nitrobacteraceae	0.214	1.37E-06	1221	PRELID2	Paucibacter	0.277	8.81E-04	1716	TNRC6B	Nitrobacteraceae	0.362	1.79E-05
232	CANT1	Ralstonia insidiosa	0.170	1.23E-03	727	IFITM10	Ralstonia insidiosa	0.264	8.77E-03	1222	PRELID3B	Comamonadaceae	-0.432	7.77E-05	1717	TNRC6C	Nitrobacteraceae	0.233	3.74E-05
233	CAPN10	Sphingomonadales	0.324	1.35E-03	728	IFT74	Ralstonia insidiosa	-0.300	6.43E-04	1223	PRKACB	Ralstonia insidiosa	-0.265	3.35E-09	1718	TOE1	Plantactinospora sp. BC1	0.204	9.48E-08
234	CAPN15	Nitrobacteraceae	0.170	4.38E-04	729	IGSF8	Bacillota	-0.340	1.10E-03	1224	PRKACB	Sphingomonadales	-0.340	1.86E-06	1719	TOM1L2	Ralstonia insidiosa	0.324	2.14E-03
235	CAPN7	Chromatiaceae	0.304	7.43E-03	730	IHH	Sphingomonadales	0.314	1.40E-03	1225	PRKAR1A	Saccharomycetales	0.444	1.58E-03	1720	TOMM20	Anoxybacillus	0.195	1.55E-03
236	CAPN7	Ralstonia insidiosa	-0.240	2.26E-03	731	IKZF5	Chromatiaceae	0.146	6.09E-08	1226	PRKD3	Bacillota	0.370	8.07E-03	1721	TOMM20	Ralstonia insidiosa	-0.382	6.97E-09
237	CAPRIN1	Bacillota	0.548	4.91E-03	732	IKZF5	Comamonadaceae	-0.292	3.03E-07	1227	PRKD3	Sphingomonadales	-0.255	5.57E-03	1722	TOMM40	Bacillota	-0.345	2.79E-03
238	CAPZA1	Ralstonia insidiosa	-0.230	1.52E-04	733	IKZF5	Malassezia vespertilionis	0.294	1.56E-05	1228	PRNP	Ralstonia insidiosa	-0.319	1.17E-03	1723	TOMM40	Sphingomonadales	0.396	2.60E-03
239	CARD10	Sphingomonadales	0.329	3.28E-03	734	IL17D	Cloacibacterium normanense	-0.234	5.61E-03	1229	PROSER1	Corynebacterium durum	0.268	1.97E-05	1724	TOMM7	Brucella	-0.286	3.40E-03
240	CARM1	Ralstonia insidiosa	0.171	1.41E-03	735	IL18BP	Plantactinospora sp. BC1	0.258	6.42E-03	1230	PRPF38B	Nitrobacteraceae	0.110	2.37E-03	1725	TOMM70	Bacillota	0.402	5.53E-07
241	CARNMT1	Comamonadaceae	-0.277	2.39E-03	736	IL4R	Plantactinospora sp. BC1	0.336	5.70E-03	1231	PRPF4B	Chromatiaceae	0.300	2.97E-03	1726	TOMM70	Ralstonia insidiosa	-0.351	4.56E-05
242	CASD1	Ralstonia insidiosa	-0.199	5.05E-03	737	ILRUN	Nitrobacteraceae	-0.295	2.61E-04	1232	PRPF8	Cloacibacterium normanense	0.441	2.06E-03	1727	TONSL	Ralstonia insidiosa	0.301	2.22E-04
243	CATSPERB	Nevskiales	0.232	1.41E-04	738	IMMP2L	Cloacibacterium normanense	-0.245	1.09E-03	1233	PRR12	Ralstonia insidiosa	0.442	3.50E-05	1728	TP53	Ralstonia insidiosa	0.301	3.57E-03
244	CBLL1	Bacillota	0.263	1.53E-03	739	IMPA2	Comamonadaceae	0.249	1.69E-04	1234	PRRC1	Ralstonia insidiosa	-0.140	6.82E-04	1729	TP53INP1	Micromonospora	0.320	9.81E-03
245	CBR4	Ralstonia insidiosa	-0.335	5.37E-03	740	ING3	Burkholderiaceae	-0.258	8.94E-04	1235	PRRG4	Comamonadaceae	-0.468	8.59E-04	1730	TPGS1	Nitrobacteraceae	0.100	4.19E-24
246	CBX3	Ralstonia insidiosa	-0.228	2.86E-03	741	INHBC	Micromonosporales	0.359	5.44E-04	1236	PRRT2	Cloacibacterium normanense	-0.383	2.78E-03	1731	TPRN	Bacillota	-0.399	1.90E-15
247	CBX4	Nitrobacteraceae	0.229	4.84E-03	742	INHBE	Micromonospora	0.435	5.87E-03	1237	PRX	Sphingomonadaceae	0.304	5.40E-04	1732	TRAF2	Bacillota	-0.390	8.74E-05
248	CC2D1A	Bacillota	-0.318	5.54E-03	743	INO80E	Paracoccaceae	0.404	2.26E-03	1238	PSMA1	Ralstonia insidiosa	-0.366	3.70E-03	1733	TRAF6	Ralstonia insidiosa	-0.282	3.31E-04
249	CC2D1A	Sphingomonadales	0.281	7.41E-04	744	INPP4A	Planctomycetia	0.379	6.69E-03	1239	PSMC6	Ralstonia insidiosa	-0.371	1.04E-03	1734	TRAM1	Ralstonia insidiosa	-0.257	1.06E-06
250	CCAR2	Ralstonia insidiosa	0.387	9.53E-04	745	INPP5E	Sphingomonadales	0.375	5.23E-03	1240	PSTPIP2	Verrucomicrobiota	-0.191	1.07E-03	1735	TRAPPC11	Bacillota	0.474	4.27E-03
251	CCDC122	Neisseria	-0.348	5.10E-04	746	INSIG2	Ralstonia insidiosa	-0.332	3.09E-05	1241	PTAR1	Ralstonia insidiosa	-0.134	2.44E-03	1736	TRAPPC14	Ralstonia insidiosa	0.308	4.00E-03
252	CCDC137	Sphingomonadales	0.415	8.13E-03	747	INSR	Nitrobacteraceae	0.226	3.31E-03	1242	PTBP3	Ralstonia insidiosa	-0.150	2.18E-04	1737	TRAPPC6B	Ralstonia insidiosa	-0.310	2.82E-08
253	CCDC142	Bacillota	-0.482	8.68E-03	748	INTS6	Xanthomonadales	-0.337	1.68E-03	1243	PTCH2	Ralstonia insidiosa	0.385	3.76E-09	1738	TRAPPC8	Ralstonia insidiosa	-0.223	3.15E-03
254	CCDC18	Corynebacterium accolens	0.143	9.73E-03	749	INTS7	Corynebacterium durum	0.444	1.79E-03	1244	PTEN	Comamonadaceae	-0.444	4.11E-04	1739	TRIM13	Ralstonia insidiosa	-0.357	5.49E-05
255	CCDC194	Methyloversatilis sp. RAC08	-0.434	1.01E-03	750	INTS8	Bacillota	0.474	1.40E-03	1245	PTEN	Ralstonia insidiosa	-0.086	8.29E-05	1740	TRIM3	Ralstonia insidiosa	0.274	5.12E-03
256	CCDC198	Ralstonia insidiosa	-0.286	7.85E-03	751	IPMK	Comamonadaceae	-0.288	1.00E-09	1246	PTGER2	Neisseria	-0.273	9.25E-03	1741	TRIM33	Comamonadaceae	-0.332	5.33E-03
257	CCDC28B	Bacillota	-0.314	9.03E-03	752	IPO5	Ralstonia insidiosa	-0.387	9.61E-03	1247	PTGES3	Ralstonia insidiosa	-0.355	7.80E-05	1742	TRIM38	Anoxybacillus	-0.424	5.10E-04
258	CCDC47	Gordonia	-0.344	5.30E-03	753	IPO7	Ralstonia insidiosa	-0.341	3.67E-03	1248	PTPN23	Ralstonia insidiosa	0.277	5.93E-04	1743	TRIM38	Nitrobacteraceae	0.223	1.34E-03
259	CCDC82	Ralstonia insidiosa	-0.128	3.25E-04	754	IPO8	Ralstonia insidiosa	-0.260	4.99E-04	1249	PTPRK	Bacillota	0.419	1.58E-03	1744	TRIM59	Cloacibacterium normanense	-0.205	3.55E-03
260	CCDC85B	Nitrobacteraceae	0.085	8.94E-07	755	IQCM	Acidobacteriota	-0.164	1.86E-03	1250	PTPRS	Ralstonia insidiosa	0.331	1.71E-06	1745	TRIM74	Ralstonia insidiosa	0.287	4.62E-03
261	CCDC85B	Sphingomonadaceae	0.380	3.34E-03	756	IQCM	Verrucomicrobiota	0.051	1.05E-04	1251	PUM2	Ralstonia insidiosa	-0.143	1.63E-05	1746	TRIOBP	Ralstonia insidiosa	0.281	3.59E-04
262	CCDC88A	Comamonadaceae	-0.317	2.55E-03	757	IQGAP2	Bacillota	0.399	2.59E-04	1252	PUS1	Bacillota	-0.396	8.88E-05	1747	TRMT1	Sphingomonadales	0.312	2.94E-03
263	CCDC88B	Bacillota	-0.359	8.75E-03	758	IQSEC2	Ralstonia insidiosa	0.404	6.49E-05	1253	PUS10	Chromatiaceae	0.250	3.84E-03	1748	TRMT10B	Staphylococcus saprophyticus	-0.415	1.16E-03
264	CCDC9	Archaea	0.396	3.34E-03	759	IRAK4	Streptococcus oralis	0.043	3.67E-04	1254	PWWP2A	Staphylococcus saccharolyticus	-0.289	1.10E-03	1749	TRMT13	Chromatiaceae	0.119	8.15E-03
265	CCDC90B	Ralstonia insidiosa	-0.369	1.05E-07	760	IRF2BPL	Archaea	0.364	7.58E-04	1255	PWWP2B	Sphingomonadaceae	0.299	2.22E-10	1750	TRMT1L	Ralstonia insidiosa	-0.209	3.33E-06
266	CCDC90B	Sphingomonadales	-0.307	2.92E-03	761	IRF3	Bacillota	-0.421	3.16E-04	1256	PWWP3A	Bacillales	-0.381	4.11E-03	1751	TRPM4	Ralstonia insidiosa	0.260	2.63E-03
267	CCDC96	Nocardioidaceae	-0.267	8.49E-03	762	IRF7	Gordonia	0.246	8.10E-03	1257	QSER1	Viruses	0.422	8.13E-09	1752	TRPV1	Ralstonia insidiosa	0.347	9.76E-03

268	CCM2L	Aerococcaceae	-0.233	6.15E-04	763	IRF7	Sphingomonadales	0.335	7.24E-05	1258	RAB10	Ralstonia insidiosa	-0.134	2.64E-03	1753	TSC22D2	Xanthomonadales	-0.330	2.20E-03
269	CCM2L	Ralstonia insidiosa	0.312	5.10E-03	764	ISCA1	Ralstonia insidiosa	-0.167	9.52E-03	1259	RAB11B	Bacillota	-0.350	5.76E-04	1754	TSFM	Colletotrichum lupini	0.254	6.39E-03
270	CCNB1IP1	Fulvia fulva	0.315	4.98E-03	765	ITGA1	Bacillota	0.470	4.53E-03	1260	RAB11B	Ralstonia insidiosa	0.222	4.35E-04	1755	TSNARE1	Ralstonia insidiosa	0.232	8.35E-04
271	CCNC	Ralstonia insidiosa	-0.271	3.87E-04	766	ITPKA	Staphylococcus saccharolyticus	0.209	2.31E-10	1261	RAB11B	Sphingomonadales	0.284	7.61E-03	1756	TSPAN31	Nitrobacteraceae	-0.234	3.39E-12
272	CCNG1	Ralstonia insidiosa	-0.394	4.74E-06	767	ITPRID2	Ralstonia insidiosa	-0.135	1.15E-03	1262	RAB11FIP3	Ralstonia insidiosa	0.295	1.07E-03	1757	TSPAN6	Ralstonia insidiosa	-0.349	1.49E-04
273	CCNT2	Chromatiaceae	0.223	2.32E-03	768	ITPRID2	Sphingomonadales	-0.351	1.59E-04	1263	RAB13	Archaea	0.439	5.89E-03	1758	TSPAN6	Sphingomonadales	-0.310	4.68E-03
274	CCNT2	Ralstonia insidiosa	-0.098	1.17E-03	769	ITPRIPL2	Nitrobacteraceae	0.181	3.26E-03	1264	RAB18	Comamonadaceae	-0.295	3.88E-08	1759	TSPAN7	Rothia mucilaginosa	-0.293	5.66E-03
275	CCPG1	Comamonadaceae	-0.305	9.63E-03	770	ITSN1	Archaea	-0.507	2.65E-03	1265	RAB18	Ralstonia insidiosa	-0.191	7.73E-06	1760	TSPO2	Streptococcaceae	-0.234	9.14E-04
276	CCR10	Nitrobacteraceae	0.224	1.45E-03	771	JKAMP	Ralstonia insidiosa	-0.343	4.83E-04	1266	RAB21	Comamonadaceae	-0.337	4.03E-03	1761	TSPOAP1	Ralstonia insidiosa	0.173	1.56E-03
277	CCSER2	Chromatiaceae	0.319	1.44E-03	772	JMJD8	Gordonia	0.219	3.99E-03	1267	RAB22A	Ralstonia insidiosa	-0.221	1.71E-03	1762	TSR3	Sphingomonadaceae	0.304	4.93E-03
278	CCSER2	Sphingomonadales	-0.303	2.05E-04	773	KANK3	Ralstonia insidiosa	0.339	4.10E-04	1268	RAB28	Ralstonia insidiosa	-0.216	2.88E-03	1763	TSSK6	Ralstonia insidiosa	0.339	5.51E-05
279	CD164	Ralstonia insidiosa	-0.184	5.87E-03	774	KAT2B	Ralstonia insidiosa	-0.304	2.35E-04	1269	RAB2A	Comamonadaceae	-0.333	3.83E-03	1764	TTC14	Chromatiaceae	0.173	5.50E-03
280	CD22	Ralstonia insidiosa	0.186	5.27E-03	775	KATNBL1	Comamonadaceae	-0.366	8.58E-05	1270	RAB2A	Ralstonia insidiosa	-0.106	2.18E-03	1765	TTC21B	Archaea	-0.467	7.72E-03
281	CD2AP	Sphingomonadales	-0.307	1.55E-04	776	KATNBL1	Ralstonia insidiosa	-0.130	1.55E-03	1271	RAB33B	Comamonadaceae	-0.415	1.17E-04	1766	TTC21B	Ralstonia insidiosa	-0.217	9.66E-03
282	CD320	Bacillota	-0.324	5.15E-04	777	KAZALD1	Neisseria	0.286	3.34E-03	1272	RAB6C	Deinococci	-0.268	5.41E-03	1767	TTC32	Cloacibacterium caeni	0.061	2.25E-03
283	CD46	Bacillota	0.474	2.22E-03	778	KCNH5	Paucibacter	0.100	5.97E-03	1273	RABEP1	Mycobacteroides	-0.164	2.63E-03	1768	TTC33	Ralstonia insidiosa	-0.180	9.36E-04
284	CD46	Ralstonia insidiosa	-0.316	8.63E-04	779	KCNJ10	Pantoea agglomerans	0.410	2.02E-03	1274	RABL6	Ralstonia insidiosa	0.283	6.99E-04	1769	TTF1	Archaea	0.465	4.64E-03
285	CD47	Ralstonia insidiosa	-0.346	4.42E-05	780	KCNJ13	Archaea	0.383	2.70E-03	1275	RAD17	Ralstonia insidiosa	-0.282	1.65E-05	1770	TUBB4B	Sphingomonadales	0.266	5.46E-05
286	CD7	Ralstonia insidiosa	0.210	2.71E-03	781	KCNJ13	Plantactinospora sp. BB1	-0.379	6.28E-03	1276	RAD21	Sphingomonadales	-0.350	5.41E-03	1771	TUBE1	Comamonadaceae	-0.270	2.42E-03
287	CDC27	Bacillota	0.473	2.11E-03	782	KCNK6	Ralstonia insidiosa	0.359	3.50E-05	1277	RAD23A	Ralstonia insidiosa	0.203	9.88E-05	1772	TUG1	Ralstonia insidiosa	-0.262	4.94E-04
288	CDC27	Ralstonia insidiosa	-0.304	1.76E-04	783	KDM4C	Streptococcus oralis	0.151	1.24E-03	1278	RAI1	Ralstonia insidiosa	0.250	1.85E-05	1773	TUT1	Ralstonia insidiosa	0.149	1.61E-03
289	CDC42SE2	Saccharomycetales	0.402	9.20E-07	784	KDR	Cloacibacterium normanense	0.354	5.73E-04	1279	RAMP1	Burkholderiaceae	0.243	6.72E-04	1774	TUT4	Viruses	0.289	3.56E-03
290	CDK11B	Archaea	0.454	4.95E-04	785	KHSRP	Ralstonia insidiosa	0.235	2.85E-04	1280	RANBP6	Ralstonia insidiosa	-0.345	1.74E-07	1775	TVP23A	Paucibacter	0.242	5.87E-03
291	CDK12	Viruses	0.304	2.07E-03	786	KIAA0513	Ralstonia insidiosa	0.365	9.85E-03	1281	RANBP6	Sphaerotilaceae	-0.401	6.77E-03	1776	TWF1	Comamonadaceae	-0.352	1.11E-03
292	CDK14	Comamonadaceae	-0.249	1.44E-03	787	KLHDC8B	Viruses	-0.391	8.18E-05	1282	RANGAP1	Bacillota	-0.269	3.31E-04	1777	TXNDC5	Thermaceae	0.376	1.89E-03
293	CDKN1B	Comamonadaceae	-0.367	7.50E-03	788	KLHL18	Anoxybacillus	-0.117	4.64E-08	1283	RANGAP1	Sphingomonadales	0.275	8.56E-07	1778	TXNL1	Ralstonia insidiosa	-0.351	3.63E-04
294	CENPB	Bacillota	-0.375	4.00E-05	789	KLHL2	Sphingomonadales	-0.295	3.43E-03	1284	RAP2C	Comamonadaceae	-0.500	2.24E-08	1779	UAP1L1	Ralstonia insidiosa	0.291	1.69E-04
295	CEP170B	Ralstonia insidiosa	0.214	1.65E-12	790	KLHL26	Ralstonia insidiosa	0.245	3.46E-03	1285	RASA2	Chromatiaceae	0.279	9.37E-03	1780	UBA3	Comamonadaceae	-0.229	9.42E-05
296	CEP170B	Sphingomonadales	0.369	9.84E-33	791	KLHL28	Comamonadaceae	-0.312	8.30E-14	1286	RASAL3	Bacillota	-0.466	6.88E-03	1781	UBA3	Malasseziales	0.246	9.39E-05
297	CEP290	Cloacibacterium caeni	-0.200	5.99E-03	792	KMT2B	Ralstonia insidiosa	0.369	3.06E-04	1287	RASL11B	Uroviricota	-0.273	3.30E-03	1782	UBA3	Ralstonia insidiosa	-0.055	3.10E-03
298	CEP70	Ralstonia insidiosa	-0.308	5.50E-04	793	KMT5C	Flavobacteriales	-0.273	5.19E-04	1288	RASSF5	Paucibacter	-0.263	8.89E-03	1783	UBA6	Ralstonia insidiosa	-0.068	1.06E-03
299	CEPT1	Ralstonia insidiosa	-0.145	1.27E-06	794	KNTC1	Viruses	0.367	7.83E-04	1289	RASSF7	Sphingomonadales	0.325	2.52E-10	1784	UBD	Nevskiales	0.050	8.06E-06
300	CETN3	Xanthomonadaceae	-0.283	4.65E-03	795	KRBA1	Ralstonia insidiosa	0.284	5.36E-06	1290	RAVER1	Ralstonia insidiosa	0.315	5.76E-03	1785	UBE2D3	Ralstonia insidiosa	-0.284	1.13E-19
301	CFAP298-TC	Staphylococcus saprophyticus	-0.179	4.03E-12	796	KRTAP5-10	Viruses	-0.283	2.70E-03	1291	RAVER1	Sphingomonadales	0.400	1.39E-04	1786	UBE2D3	Xanthomonadaceae	-0.242	8.29E-03
302	CFAP410	Bacillota	-0.519	7.14E-03	797	KTN1	Sphingomonadales	-0.364	9.16E-03	1292	RBBP6	Nitrobacteraceae	0.313	2.22E-03	1787	UBE2J2	Bacillales	-0.489	5.00E-03
303	CFAP418	Cloacibacterium caeni	-0.057	5.75E-04	798	KYAT1	Oceanospirillales	-0.425	4.38E-07	1293	RBBP9	Ralstonia insidiosa	-0.228	1.43E-03	1788	UBE2N	Ralstonia insidiosa	-0.253	3.33E-12
304	CFAP97	Bacillota	0.405	5.69E-03	799	KYAT1	Ralstonia insidiosa	0.122	5.75E-04	1294	RBM10	Gordonia	0.237	4.09E-03	1789	UBE2V1	Cloacibacterium normanense	0.087	8.10E-04
305	CFL2	Ralstonia insidiosa	-0.358	1.42E-10	800	LACTB2	Comamonadaceae	-0.282	3.48E-03	1295	RBM41	Corynebacterium accolens	0.333	2.29E-05	1790	UBE4A	Saccharomycetales	0.442	1.99E-03
306	CFP	Ralstonia insidiosa	0.151	9.98E-03	801	LAMTOR2	Viruses	-0.319	9.25E-03	1296	RBMX2	Cloacibacterium normanense	-0.262	2.96E-03	1791	UBL3	Comamonadaceae	-0.260	6.43E-05
307	CGGBP1	Ralstonia insidiosa	-0.270	7.78E-11	802	LAMTOR3	Ralstonia insidiosa	-0.253	8.38E-04	1297	RBMXL1	Ralstonia insidiosa	-0.258	4.15E-03	1792	UBQLN1	Bacillota	0.492	8.14E-05
308	CHAF1A	Ralstonia insidiosa	0.300	5.09E-03	803	LANCL1	Ralstonia insidiosa	-0.328	1.54E-03	1298	RBPJ	Chromatiaceae	0.250	4.50E-03	1793	UBQLN1	Ralstonia insidiosa	-0.258	4.85E-08
309	CHCHD1	Anoxybacillus	0.028	7.67E-03	804	LARP7	Cloacibacterium normanense	-0.315	2.33E-06	1299	RBPJ	Comamonadaceae	-0.262	8.51E-04	1794	UBR3	Viruses	0.266	1.71E-16
310	CHCHD2	Viruses	-0.422	3.00E-04	805	LATS1	Viruses	0.292	6.20E-03	1300	RBX1	Viruses	-0.403	6.54E-04	1795	UBXN11	Ralstonia insidiosa	0.298	4.03E-03
311	CHD2	Nitrobacteraceae	0.327	6.63E-07	806	LBR	Microbacteriaceae	-0.280	5.14E-03	1301	RC3H2	Ralstonia insidiosa	-0.244	2.79E-03	1796	UBXN8	Ralstonia insidiosa	-0.395	1.74E-07
312	CHERP	Ralstonia insidiosa	0.274	1.53E-03	807	LBR	Ralstonia insidiosa	-0.219	1.47E-03	1302	RCBTB2	Ralstonia insidiosa	-0.288	4.50E-05	1797	UHMK1	Ralstonia insidiosa	-0.217	2.80E-05
313	CHM	Viruses	0.386	5.92E-03	808	LDB2	Cloacibacterium normanense	0.373	3.12E-03	1303	RCC1L	Bacillota	-0.306	3.57E-03	1798	UHRF2	Ralstonia insidiosa	-0.221	5.38E-05
314	CHMP1A	Bacillota	-0.310	5.87E-05	809	LDHB	Brucella	-0.277	5.20E-04	1304	RCHY1	Ralstonia insidiosa	-0.260	2.50E-04	1799	UNC13D	Gordonia	0.183	1.06E-03
315	CHMP2B	Ralstonia insidiosa	-0.193	1.02E-05	810	LENG1	Cloacibacterium normanense	-0.421	3.20E-03	1305	REEP3	Comamonadaceae	-0.280	5.23E-03	1800	UNC93B1	Ralstonia insidiosa	0.206	1.56E-08
316	CHN2	Bacilli	0.447	6.24E-04	811	LETM1	Ralstonia insidiosa	0.342	1.34E-03	1306	REEP3	Ralstonia insidiosa	-0.036	5.51E-03	1801	UNC93B1	Rheinheimera	-0.324	2.03E-06
317	CHTF18	Prevotella melaninogenica	-0.353	5.30E-06	812	LGSN	Pasteurellaceae	0.390	7.14E-03	1307	REEP5	Ralstonia insidiosa	-0.345	3.87E-05	1802	UPF2	Nitrobacteraceae	0.458	4.68E-03
318	CHTF18	Ralstonia insidiosa	0.209	4.57E-03	813	LIG3	Tepidimonas taiwanensis	-0.187	1.30E-03	1308	REEP5	Xanthomonadaceae	-0.276	9.36E-03	1803	UPF3B	Corynebacterium accolens	0.158	7.18E-06
319	CHUK	Ralstonia insidiosa	-0.256	1.88E-04	814	LILRB1	Ralstonia insidiosa	0.337	1.88E-03	1309	RELA	Ralstonia insidiosa	0.206	5.30E-03	1804	UPP1	Nectriaceae	-0.305	1.59E-10
320	CHUK	Sphaerotilaceae	-0.343	2.17E-03	815	LILRB2	Ralstonia insidiosa	0.285	9.58E-03	1310	RELCH	Chromatiaceae	0.259	5.84E-03	1805	UQCRQ	Viruses	-0.409	4.98E-03
321	CIC	Ralstonia insidiosa	0.321	2.35E-14	816	LIMK1	Ralstonia insidiosa	0.260	8.85E-03	1311	RENBP	Aerococcaceae	-0.247	2.56E-03	1806	URB1	Viruses	0.292	4.62E-03

322	CIC	Sphingomonadales	0.261	3.85E-08	817	LIMS2	Bacillota	-0.454	2.61E-03	1312	REPS2	Fusobacterium	-0.276	4.70E-05	1807	USF2	Ralstonia insidiosa	0.170	2.01E-03
323	CITED4	Sphingomonadales	0.445	1.85E-03	818	LIN54	Comamonadaceae	-0.245	2.86E-07	1313	RERE	Ralstonia insidiosa	0.354	9.70E-03	1808	USP1	Ralstonia insidiosa	-0.114	3.66E-08
324	CKAP4	Brucella	-0.196	6.02E-04	819	LIN7A	Ralstonia insidiosa	-0.261	4.16E-04	1314	REV1	Cloacibacterium caeni	-0.186	1.91E-08	1809	USP12	Ralstonia insidiosa	-0.237	7.59E-06
325	CKAP4	Staphylococcus capitis	0.274	8.47E-07	820	LIN7C	Comamonadaceae	-0.357	6.72E-03	1315	REV3L	Viruses	0.268	3.51E-03	1810	USP14	Ralstonia insidiosa	-0.200	9.39E-03
326	CLASRP	Sphingomonadales	0.328	1.06E-03	821	LLGL2	Bacillota	-0.407	6.20E-03	1316	REX1BD	Bacillales	-0.517	3.30E-14	1811	USP15	Bacillota	0.338	7.22E-09
327	CLCN4	Viruses	0.423	4.18E-03	822	LMAN1	Bacillota	0.347	1.46E-03	1317	REX1BD	Sphingomonadales	0.413	6.90E-05	1812	USP15	Chromatiaceae	0.265	1.24E-07
328	CLCN7	Ralstonia insidiosa	0.199	4.18E-04	823	LMAN1	Ralstonia insidiosa	-0.322	2.71E-04	1318	REXO1	Ralstonia insidiosa	0.332	1.07E-04	1813	USP15	Ralstonia insidiosa	-0.147	2.43E-08
329	CLDN1	Ralstonia insidiosa	-0.329	8.56E-04	824	LMBR1	Ralstonia insidiosa	-0.324	4.47E-03	1319	RFLNB	Rothia mucilaginosa	-0.355	4.69E-03	1814	USP33	Bacillota	0.423	6.50E-11
330	CLDN12	Ralstonia insidiosa	-0.342	1.14E-21	825	LMBRD1	Ralstonia insidiosa	-0.376	3.49E-05	1320	RHBDD1	Bacillota	0.497	4.41E-03	1815	USP42	Nitrobacteraceae	0.436	2.55E-04
331	CLDN12	Sphingomonadales	-0.414	5.97E-05	826	LMBRD2	Ralstonia insidiosa	-0.176	6.74E-03	1321	RHBDD1	Ralstonia insidiosa	-0.274	4.11E-04	1816	VAV1	Ralstonia insidiosa	0.290	3.17E-03
332	CLDN20	Nocardiaceae	0.232	8.78E-03	827	LNPK	Comamonadaceae	-0.298	3.25E-03	1322	RHOBTB2	Ralstonia insidiosa	0.358	5.36E-04	1817	VAV3	Mycobacteroides	-0.095	4.23E-04
333	CLDN34	Nevskiales	-0.400	1.53E-05	828	LONP1	Ralstonia insidiosa	0.223	6.37E-05	1323	RHOT1	Bacillota	0.453	5.98E-06	1818	VCP	Nocardioidaceae	-0.359	2.02E-03
334	CLDN5	Ralstonia insidiosa	0.264	4.76E-03	829	LONRF1	Ralstonia insidiosa	-0.224	2.42E-06	1324	RHOT1	Ralstonia insidiosa	-0.241	1.10E-04	1819	VEZF1	Ralstonia insidiosa	-0.110	5.61E-03
335	CLDND2	Shinella	-0.385	4.19E-03	830	LRCH4	Ralstonia insidiosa	0.316	5.83E-03	1325	RIC1	Viruses	0.415	6.97E-14	1820	VEZT	Bacillota	0.519	9.04E-03
336	CLEC16A	Dermacoccaceae	-0.414	5.38E-06	831	LRFN3	Ralstonia insidiosa	0.189	1.11E-03	1326	RICTOR	Chromatiaceae	0.276	2.07E-11	1821	VEZT	Ralstonia insidiosa	-0.279	2.63E-05
337	CLEC16A	Ralstonia insidiosa	0.272	6.15E-06	832	LRGUK	Streptococcus salivarius	0.148	3.70E-03	1327	RICTOR	Ralstonia insidiosa	-0.032	4.59E-09	1822	VIM	Brucella	-0.192	1.25E-03
338	CLEC16A	Sphingomonadales	0.260	1.67E-03	833	LRP11	Ralstonia insidiosa	-0.233	7.88E-05	1328	RIF1	Microbacteriaceae	-0.416	3.08E-04	1823	VMP1	Verrucomicrobiota	-0.325	3.10E-05
339	CLEC3B	Aerococcaceae	-0.211	8.71E-03	834	LRRC40	Ralstonia insidiosa	-0.229	3.25E-13	1329	RILPL1	Ralstonia insidiosa	0.439	4.22E-04	1824	VPS16	Gordonia	0.360	3.44E-06
340	CMIP	Ralstonia insidiosa	0.507	1.15E-04	835	LRRC42	Fulvia fulva	0.267	1.70E-03	1330	RIMOC1	Saccharomycetales	0.391	1.13E-03	1825	VPS36	Chromatiaceae	0.248	3.04E-04
341	CMPK1	Ralstonia insidiosa	-0.258	3.52E-07	836	LRRC58	Comamonadaceae	-0.304	3.28E-04	1331	RIN3	Ralstonia insidiosa	0.257	2.04E-05	1826	VPS36	Ralstonia insidiosa	-0.156	1.03E-07
342	CMTM6	Ralstonia insidiosa	-0.348	1.73E-04	837	LRRC61	Sphingomonadales	0.359	8.98E-03	1332	RINT1	Ralstonia insidiosa	-0.200	3.12E-03	1827	VPS37A	Archaea	-0.464	9.25E-04
343	CNEP1R1	Chromatiaceae	0.225	1.58E-03	838	LRRC69	Cloacibacterium normanense	-0.219	1.85E-03	1333	RIPOR1	Ralstonia insidiosa	0.294	3.39E-08	1828	VPS37C	Bacillota	-0.342	1.40E-15
344	CNIH1	Ralstonia insidiosa	-0.404	1.91E-06	839	LRRTM3	Bacteria	-0.385	8.29E-03	1334	RMDN2	Ralstonia insidiosa	-0.291	4.77E-05	1829	VPS37C	Ralstonia insidiosa	0.289	1.05E-11
345	CNIH1	Sphingomonadales	-0.352	1.77E-03	840	LRWD1	Ralstonia insidiosa	0.286	3.02E-04	1335	RMDN3	Caulobacteraceae	0.400	8.66E-03	1830	VPS45	Actinomycetota	0.441	2.87E-03
346	CNNM2	Rhodocyclales	0.115	3.65E-03	841	LSM8	Ralstonia insidiosa	-0.334	4.15E-03	1336	RNF11	Comamonadaceae	-0.324	2.17E-06	1831	VPS45	Corynebacterium durum	0.315	3.63E-07
347	CNOT3	Sphingomonadales	0.280	8.82E-05	842	LTC4S	Nitrobacteraceae	0.251	7.52E-05	1337	RNF126	Nitrobacteraceae	0.094	1.09E-04	1832	VPS50	Ralstonia insidiosa	-0.260	1.20E-03
348	CNOT6L	Ralstonia insidiosa	-0.141	1.55E-04	843	LTK	Ralstonia insidiosa	0.282	7.66E-04	1338	RNF128	Ralstonia insidiosa	-0.188	2.47E-04	1833	VPS53	Ralstonia insidiosa	0.385	3.38E-04
349	CNPY2	Lactococcus	0.286	9.06E-03	844	LTN1	Ralstonia insidiosa	-0.253	9.47E-03	1339	RNF128	Sphingobacteriales	0.327	2.03E-03	1834	VPS54	Ralstonia insidiosa	-0.148	8.80E-04
350	CNTNAP3C	Corynebacteriaceae	-0.399	7.39E-04	845	LTN1	Viruses	0.321	3.03E-03	1340	RNF128	Sphingomonadales	-0.311	3.25E-03	1835	VPS9D1	Bacillota	-0.449	3.59E-04
351	CNTNAP4	Ralstonia insidiosa	0.192	8.73E-03	846	LUC7L3	Corynebacterium accolens	-0.003	4.08E-03	1341	RNF13	Ralstonia insidiosa	-0.284	5.06E-04	1836	VRK2	Viruses	0.406	9.38E-03
352	COA8	Viruses	-0.325	1.83E-03	847	LYPLA1	Comamonadaceae	-0.402	9.01E-04	1342	RNF138	Comamonadaceae	-0.443	5.89E-07	1837	VTA1	Bacillales	0.425	1.10E-03
353	COBLL1	Malassezia vespertilionis	0.323	6.05E-03	848	LYPLAL1	Ralstonia insidiosa	-0.194	4.26E-04	1343	RNF139	Ralstonia insidiosa	-0.293	1.10E-28	1838	VTA1	Ralstonia insidiosa	-0.396	4.21E-09
354	COG5	Viruses	0.378	4.70E-03	849	LYRM7	Comamonadaceae	-0.391	1.79E-03	1344	RNF141	Ralstonia insidiosa	-0.197	5.67E-07	1839	VWA1	Sphingomonadales	0.337	9.66E-03
355	COG6	Ralstonia insidiosa	-0.234	2.99E-16	850	LYST	Chromatiaceae	0.260	3.99E-03	1345	RNF141	Sphingomonadales	-0.257	2.30E-10	1840	WAC	Bacillota	0.481	3.25E-13
356	COG6	Sphingomonadales	-0.299	1.19E-05	851	LZTFL1	Ralstonia insidiosa	-0.339	1.23E-03	1346	RNF149	Cloacibacterium normanense	-0.248	1.80E-06	1841	WAC	Ralstonia insidiosa	-0.190	7.71E-03
357	COL18A1	Ralstonia insidiosa	0.199	1.92E-03	852	MAF	Comamonadaceae	-0.345	3.04E-03	1347	RNF149	Mycobacteroides	0.210	8.80E-03	1842	WAC	Sphingomonadales	-0.283	2.58E-03
358	COL22A1	Streptococcus sanguinis	0.129	7.30E-03	853	MAF	Nitrobacteraceae	0.283	2.97E-03	1348	RNF166	Ralstonia insidiosa	0.328	2.93E-03	1843	WASL	Comamonadaceae	-0.279	9.35E-03
359	COL27A1	Ralstonia insidiosa	0.299	8.18E-03	854	MAK	Corynebacterium accolens	0.041	7.56E-06	1349	RNF181	Viruses	-0.230	7.57E-04	1844	WBP4	Cloacibacterium normanense	-0.180	2.71E-05
360	COL6A5	Streptococcus sanguinis	-0.015	2.30E-04	855	MAL	Cloacibacterium caeni	0.324	5.98E-03	1350	RNF185	Nitrobacteraceae	-0.298	6.69E-05	1845	WDR24	Bacillota	-0.306	5.95E-03
361	COL9A1	Paucibacter	0.134	6.39E-03	856	MAL2	Ralstonia insidiosa	-0.233	3.54E-08	1351	RNF187	Bacillota	-0.476	6.22E-03	1846	WDR26	Ralstonia insidiosa	-0.193	8.42E-04
362	COMMD10	Comamonadaceae	-0.301	6.46E-05	857	MAN1C1	Micromonospora	-0.251	1.57E-04	1352	RNF208	Ralstonia insidiosa	0.274	7.71E-03	1847	WDR44	Bacillota	0.511	2.28E-03
363	COMMD10	Ralstonia insidiosa	-0.193	7.05E-04	858	MAN2A1	Bacillota	0.386	1.06E-03	1353	RNF217	Comamonadaceae	-0.372	2.22E-03	1848	WDR49	Paucibacter	0.160	1.68E-06
364	COMMD7	Viruses	-0.288	6.56E-04	859	MAN2B1	Ralstonia insidiosa	0.213	1.74E-03	1354	RNF217	Sordariomycetes	-0.371	6.35E-03	1849	WDR83	Ralstonia insidiosa	0.267	2.16E-47
365	COMTD1	Bacillales	-0.509	2.29E-03	860	MAP1S	Ralstonia insidiosa	0.233	3.32E-03	1355	RNF44	Ralstonia insidiosa	0.308	2.07E-07	1850	WDR89	Ralstonia insidiosa	-0.380	3.76E-05
366	COPB1	Ralstonia insidiosa	-0.253	4.87E-03	861	MAP2	Staphylococcus saccharolyticus	-0.322	1.70E-03	1356	RNH1	Ralstonia insidiosa	0.216	5.11E-03	1851	WIPI1	Fusobacteriaceae	-0.339	7.88E-03
367	COPB2	Bacillota	0.465	7.12E-03	862	MAP3K10	Myxococcota	0.293	9.30E-03	1357	RNH1	Sphingomonadales	0.308	2.68E-04	1852	WIZ	Ralstonia insidiosa	0.337	9.32E-07
368	COPS2	Ralstonia insidiosa	-0.356	1.55E-06	863	MAP3K2	Ralstonia insidiosa	-0.150	5.31E-04	1358	RNPEPL1	Bacillota	-0.337	1.25E-33	1853	WIZ	Sphingomonadales	0.347	5.62E-05
369	COPS2	Sphingomonadales	-0.344	9.05E-04	864	MAP3K7	Chromatiaceae	0.296	8.74E-03	1359	RNPEPL1	Ralstonia insidiosa	0.144	1.53E-04	1854	WNK1	Viruses	0.407	4.94E-03
370	CORO1B	Ralstonia insidiosa	0.101	9.26E-04	865	MAP3K7	Ralstonia insidiosa	-0.115	1.59E-03	1360	RNPEPL1	Sphingomonadales	0.304	2.24E-16	1855	WNT9B	Ralstonia insidiosa	0.258	3.22E-21
371	COX11	Comamonadaceae	-0.467	1.82E-04	866	MAP4K2	Cloacibacterium caeni	-0.223	1.75E-03	1361	RNPS1	Bacillota	-0.343	1.46E-05	1856	WNT9B	Thermaceae	0.257	5.06E-06
372	COX14	Viruses	-0.370	1.33E-03	867	MAP4K3	Ralstonia insidiosa	-0.151	2.09E-03	1362	RNPS1	Malassezia vespertilionis	-0.312	2.73E-07	1857	WSCD1	Cloacibacterium normanense	0.149	6.37E-03
373	CPB2	Bacillota	0.412	7.28E-03	868	MAP4K5	Ralstonia insidiosa	-0.236	1.15E-03	1363	RNPS1	Sphingomonadales	0.316	9.23E-05	1858	WWP1	Ralstonia insidiosa	-0.219	3.45E-17
374	CPB2	Flavobacteriales	0.334	2.90E-04	869	MAP7D1	Ralstonia insidiosa	0.289	2.83E-08	1364	RO60	Comamonadaceae	-0.393	4.76E-08	1859	WWP1	Sphingomonadales	-0.330	4.55E-15
375	CPEB2	Chromatiaceae	0.283	1.46E-03	870	MAPK1IP1L	Malasseziales	0.237	2.86E-04	1365	ROS1	Brucella	0.191	3.58E-04	1860	XIRP2	Brevibacteriaceae	-0.241	1.11E-03

376	CPEB2	Comamonadaceae	-0.289	3.36E-03	871	MAPK6	Viruses	0.395	2.13E-05	1366	RPAP3	Ralstonia insidiosa	-0.236	1.04E-03	1861	XNDC1N-ZNF	Kocuria palustris	0.129	1.12E-14
377	CPLX1	Ralstonia insidiosa	0.269	5.37E-03	872	MAPK7	Ralstonia insidiosa	0.245	2.81E-03	1367	RPL23A	Brucella	-0.201	5.36E-03	1862	XPO1	Chromatiaceae	0.363	8.94E-05
378	CPN2	Archaea	-0.398	2.10E-03	873	MAPK8	Archaea	-0.422	1.49E-09	1368	RPL23A	Viruses	-0.329	6.41E-03	1863	XPO1	Ralstonia insidiosa	-0.157	2.32E-04
379	CPNE3	Ralstonia insidiosa	-0.222	9.20E-05	874	MAPK8	Ralstonia insidiosa	-0.300	2.19E-24	1369	RPL24	Viruses	-0.364	8.67E-03	1864	XPOT	Ralstonia insidiosa	-0.163	4.15E-03
380	CPSF2	Sphaerotilaceae	-0.409	3.09E-03	875	MARCHF6	Comamonadaceae	-0.360	2.57E-03	1370	RPL26	Viruses	-0.260	6.77E-03	1865	XRCC2	Acinetobacter johnsonii	0.263	1.85E-04
381	CPSF6	Ralstonia insidiosa	-0.227	8.75E-03	876	MARCHF7	Ralstonia insidiosa	-0.221	7.34E-06	1371	RPL31	Brucella	-0.299	4.25E-03	1866	XRCC6	Nitrobacteraceae	-0.078	2.57E-03
382	CPT1B	Nocardiaceae	0.252	4.90E-26	877	MARCKSL1	Ralstonia insidiosa	0.171	4.26E-03	1372	RPL31	Viruses	-0.321	5.43E-03	1867	XYLT2	Nitrosomonadales	-0.222	5.06E-20
383	CPTP	Bacillota	-0.385	8.20E-08	878	MARK2	Ralstonia insidiosa	0.382	7.94E-05	1373	RPL34	Viruses	-0.355	1.68E-03	1868	YAP1	Ralstonia insidiosa	-0.241	8.60E-03
384	CPTP	Gordonia	0.259	8.01E-04	879	MAST4	Nitrobacteraceae	0.360	5.23E-05	1374	RPL35A	Brucella	-0.281	1.59E-03	1869	YARS1	Yersiniaceae	0.293	5.41E-03
385	CPTP	Ralstonia insidiosa	0.203	3.29E-06	880	MATR3	Ralstonia insidiosa	-0.226	2.91E-03	1375	RPL35A	Viruses	-0.343	2.06E-03	1870	YBEY	Viruses	-0.372	6.44E-03
386	CPTP	Sphingomonadales	0.372	1.05E-07	881	MAZ	Actinomycetota	-0.269	5.11E-03	1376	RPL37A	Viruses	-0.354	6.00E-03	1871	YDJC	Sphingomonadales	0.359	7.14E-04
387	CRAT	Gordonia	0.276	5.91E-03	882	MBD6	Ralstonia insidiosa	0.288	8.51E-05	1377	RPL41	Lactococcus	0.395	8.06E-06	1872	YIPF2	Aerococcaceae	-0.139	2.28E-09
388	CRBN	Ralstonia insidiosa	-0.234	7.61E-42	883	MBLAC2	Malassezia vespertilionis	0.305	7.15E-03	1378	RPL5	Lactococcus	0.369	5.89E-03	1873	YJU2B	Ralstonia insidiosa	0.198	8.23E-05
389	CRBN	Sphingomonadales	-0.284	1.54E-12	884	MBNL1	Ralstonia insidiosa	-0.022	2.35E-06	1379	RPL7	Brucella	-0.234	2.67E-03	1874	YJU2B	Sphingomonadales	0.359	3.99E-03
390	CREB1	Ralstonia insidiosa	-0.110	4.03E-03	885	MBNL3	Bacillota	0.318	7.62E-03	1380	RPLP0	Lactococcus	0.378	6.38E-03	1875	YPEL5	Ralstonia insidiosa	-0.262	1.22E-05
391	CREBL2	Ralstonia insidiosa	-0.342	1.58E-11	886	MBNL3	Comamonadaceae	-0.221	2.90E-04	1381	RPRD1A	Sphingomonadales	-0.301	6.87E-03	1876	YTHDF3	Ralstonia insidiosa	-0.254	5.14E-15
392	CREBL2	Sphingomonadales	-0.322	6.40E-07	887	MCFD2	Ralstonia insidiosa	-0.341	1.16E-04	1382	RPS18	Lactococcus	0.306	3.91E-03	1877	YWHAZ	Ralstonia insidiosa	-0.235	7.65E-03
393	CREBRF	Chromatiaceae	0.257	6.32E-05	888	MCOLN1	Ralstonia insidiosa	0.277	1.50E-04	1383	RPS27A	Brucella	-0.289	2.30E-03	1878	ZBED5	Ralstonia insidiosa	-0.223	1.22E-05
394	CREM	Rhodocyclales	-0.291	9.93E-03	889	MDFIC	Ceratobasidiaceae	0.373	5.00E-03	1384	RPS29	Viruses	-0.373	1.46E-03	1879	ZBTB11	Ralstonia insidiosa	-0.155	7.37E-03
395	CRHBP	Rothia mucilaginosa	-0.275	2.73E-03	890	MED14	Bacillota	0.389	2.23E-06	1385	RPS6KA3	Viruses	0.322	1.71E-03	1880	ZBTB11	Viruses	0.327	8.80E-03
396	CRLS1	Ralstonia insidiosa	-0.299	5.96E-06	891	MED14	Viruses	0.422	4.90E-07	1386	RPS6KB1	Ralstonia insidiosa	-0.210	8.81E-03	1881	ZBTB17	Bacillota	-0.322	2.22E-28
397	CRLS1	Xanthomonadaceae	-0.260	3.43E-03	892	MED16	Gordonia	0.290	6.37E-05	1387	RPS6KC1	Ralstonia insidiosa	-0.250	1.73E-03	1882	ZBTB17	Ralstonia insidiosa	0.243	6.97E-14
398	CROCC	Bacillales	-0.444	7.77E-10	893	MED21	Ralstonia insidiosa	-0.204	4.49E-11	1388	RPS8	Lactococcus	0.388	1.82E-03	1883	ZBTB17	Sphingomonadales	0.321	1.96E-09
399	CROCC	Ralstonia insidiosa	0.292	1.60E-10	894	MED24	Sordariomycetes	0.295	7.92E-03	1389	RPSA	Lactococcus	0.316	2.56E-03	1884	ZBTB25	Chromatiaceae	0.073	2.85E-03
400	CROCC	Sphingomonadales	0.298	1.84E-07	895	MED25	Ralstonia insidiosa	0.264	3.56E-04	1390	RPSA	Viruses	-0.311	9.89E-04	1885	ZBTB33	Ralstonia insidiosa	-0.186	6.12E-03
401	CRTC1	Ralstonia insidiosa	0.321	8.51E-03	896	MED26	Sphingomonadales	0.347	5.77E-03	1391	RPUSD4	Methylorubrum populi	-0.292	8.61E-03	1886	ZBTB45	Ralstonia insidiosa	0.286	6.41E-06
402	CSDE1	Sphingomonadales	-0.382	8.02E-04	897	MED31	Flavobacteriaceae	0.214	2.05E-04	1392	RRM2B	Ralstonia insidiosa	-0.246	1.07E-03	1887	ZBTB49	Corynebacterium durum	0.169	2.04E-04
403	CSE1L	Ralstonia insidiosa	-0.274	8.09E-03	898	MED4	Ralstonia insidiosa	-0.214	3.12E-05	1393	RSF1	Bacteria	-0.499	4.50E-03	1888	ZBTB7A	Bacillota	-0.495	1.94E-03
404	CSF3R	Ralstonia insidiosa	0.298	1.97E-03	899	MEGF6	Ralstonia insidiosa	0.282	6.59E-03	1394	RSF1	Nitrobacteraceae	0.461	2.11E-07	1889	ZBTB7A	Ralstonia insidiosa	0.324	2.86E-03
405	CSKMT	Prevotella melaninogenica	-0.235	9.69E-03	900	MEGF8	Ralstonia insidiosa	0.307	7.55E-03	1395	RTN4	Ralstonia insidiosa	-0.217	6.97E-03	1890	ZC3H12A	Actinomyces	0.177	4.35E-03
406	CSNK1G2	Ralstonia insidiosa	0.225	8.98E-07	901	MEIOB	Azospirillaceae	0.337	4.40E-03	1396	RUFY3	Cloacibacterium normanense	-0.311	8.47E-04	1891	ZC3H13	Nitrobacteraceae	0.191	2.16E-03
407	CSNK1G2	Sphingomonadaceae	0.309	9.05E-13	902	MET	Sphingomonadales	-0.336	7.92E-03	1397	RUNDC3B	Comamonadaceae	-0.418	5.52E-04	1892	ZCCHC10	Cloacibacterium normanense	-0.236	3.37E-04
408	CSNK1G3	Ralstonia insidiosa	-0.188	3.90E-03	903	METRNL	Nitrobacteraceae	0.215	1.06E-03	1398	RUVBL2	Sphingomonadales	0.242	9.08E-08	1893	ZCRB1	Cloacibacterium normanense	-0.414	1.16E-03
409	CSPP1	Nitrobacteraceae	0.185	8.67E-03	904	METTL14	Ralstonia insidiosa	-0.376	5.80E-11	1399	RWDD1	Lactococcus	0.314	8.79E-07	1894	ZCWPW1	Campylobacteraceae	-0.452	3.34E-03
410	CTDSPL2	Comamonadaceae	-0.264	2.21E-03	905	METTL15	Prevotella	0.403	7.73E-03	1400	RWDD1	Xanthomonadaceae	-0.431	4.90E-03	1895	ZDHHC17	Ralstonia insidiosa	-0.165	7.38E-04
411	CTSO	Bacillota	0.394	3.05E-19	906	METTL8	Cloacibacterium caeni	-0.101	8.34E-05	1401	RWDD3	Ralstonia insidiosa	-0.249	6.65E-04	1896	ZDHHC7	Ralstonia insidiosa	0.344	4.40E-03
412	CTSO	Ralstonia insidiosa	-0.246	3.35E-08	907	METTL8	Sordariomycetes	-0.275	2.66E-04	1402	RYBP	Ralstonia insidiosa	-0.171	1.65E-06	1897	ZEB2	Rothia mucilaginosa	-0.168	3.94E-03
413	CTSO	Sphingomonadales	-0.327	3.13E-06	908	METTL9	Brucella	-0.243	9.29E-04	1403	RYBP	Xanthomonadales	-0.263	4.49E-03	1898	ZFAND1	Ralstonia insidiosa	-0.374	2.08E-05
414	CTTN	Ralstonia insidiosa	0.270	4.06E-03	909	MEX3D	Nitrobacteraceae	0.153	6.86E-04	1404	RYK	Ralstonia insidiosa	-0.323	1.26E-05	1899	ZFAT	Archaea	-0.419	7.75E-04
415	CTTNBP2NL	Cloacibacterium normanense	0.419	1.59E-04	910	MFAP3	Ralstonia insidiosa	-0.268	6.65E-04	1405	S100A6	Brucella	-0.354	5.97E-04	1900	ZFHX3	Bacteria	-0.433	9.12E-03
416	CTU1	Archaea	0.531	9.74E-03	911	MFAP3	Saccharomycetales	0.506	7.29E-03	1406	S100PBP	Corynebacterium durum	-0.033	1.91E-03	1901	ZFHX3	Nitrobacteraceae	0.389	2.63E-03
417	CUL4B	Bacillota	0.454	2.52E-15	912	MFAP3	Sphaerotilaceae	-0.401	4.79E-03	1407	SAA4	Lactococcus	0.405	1.65E-03	1902	ZFHX3	Planctomycetia	0.493	3.99E-03
418	CUL4B	Ralstonia insidiosa	-0.335	7.19E-12	913	MFSD3	Bacillota	-0.392	2.38E-07	1408	SACM1L	Ralstonia insidiosa	-0.263	7.05E-06	1903	ZFHX4	Nitrobacteraceae	0.314	2.20E-03
419	CUL4B	Sphingomonadales	-0.374	9.77E-13	914	MFSD8	Ralstonia insidiosa	-0.230	2.64E-05	1409	SACM1L	Sphingomonadales	-0.249	6.73E-03	1904	ZFP41	Ralstonia insidiosa	0.283	9.45E-03
420	CUX1	Ralstonia insidiosa	0.259	2.60E-03	915	MGAT1	Ralstonia insidiosa	0.150	1.92E-03	1410	SAFB2	Bacillales	-0.503	2.69E-03	1905	ZFP69B	Mycobacteroides	-0.180	1.80E-04
421	CXADR	Comamonadaceae	-0.309	4.22E-03	916	MGAT4A	Ralstonia insidiosa	-0.313	9.75E-03	1411	SAFB2	Nitrobacteraceae	0.126	1.54E-05	1906	ZFPM1	Ralstonia insidiosa	0.253	4.59E-03
422	CYBA	Ralstonia insidiosa	0.283	5.07E-03	917	MGAT4B	Ralstonia insidiosa	0.208	9.45E-05	1412	SAMD10	Sphingomonadales	0.362	9.32E-04	1907	ZGLP1	Bacillota	-0.444	5.44E-03
423	CYBC1	Ralstonia insidiosa	0.148	9.80E-04	918	MGRN1	Ralstonia insidiosa	0.282	8.46E-04	1413	SAP25	Ralstonia insidiosa	0.266	1.28E-08	1908	ZHX1	Comamonadaceae	-0.260	3.09E-04
424	CYP11A1	Acinetobacter lwoffii	-0.196	4.47E-03	919	MIB1	Ralstonia insidiosa	-0.171	3.76E-03	1414	SART1	Bacillota	-0.377	2.21E-06	1909	ZMAT2	Brucella	-0.303	5.09E-03
425	CYP20A1	Corynebacterium accolens	0.216	8.17E-05	920	MICAL1	Fusobacterium	0.141	1.81E-06	1415	SATB2	Brucella	0.318	2.09E-03	1910	ZMPSTE24	Bacillota	0.399	7.75E-05
426	DACH1	Rothia mucilaginosa	-0.102	8.32E-04	921	MICAL1	Ralstonia insidiosa	0.203	9.41E-04	1416	SBDS	Ralstonia insidiosa	-0.355	3.09E-06	1911	ZMPSTE24	Ralstonia insidiosa	-0.248	1.61E-03
427	DAZAP1	Bacillota	-0.469	1.01E-09	922	MICAL3	Ralstonia insidiosa	0.315	6.13E-03	1417	SBNO1	Nitrobacteraceae	0.519	4.62E-04	1912	ZMYM2	Ralstonia insidiosa	-0.160	1.93E-03
428	DAZAP1	Sphingomonadales	0.398	8.92E-10	923	MICALL1	Ralstonia insidiosa	0.285	1.16E-03	1418	SBNO2	Ralstonia insidiosa	0.296	2.13E-03	1913	ZMYM4	Ralstonia insidiosa	-0.247	1.42E-03
429	DAZAP2	Ralstonia insidiosa	-0.339	5.31E-03	924	MICOS10	Viruses	-0.454	2.55E-04	1419	SCAF1	Bacillota	-0.320	1.36E-04	1914	ZMYND11	Ralstonia insidiosa	-0.251	1.66E-15

430	DBI	Caulobacteraceae	0.387	2.43E-03	925	MICU2	Ralstonia insidiosa	-0.336	5.04E-05	1420	SCAF1	Ralstonia insidiosa	0.250	1.68E-07	1915	ZNF101	Cloacibacterium normanense	-0.231	6.81E-05
431	DCAF10	Diaphorobacter	0.408	2.31E-06	926	MICU3	Comamonadaceae	-0.423	5.92E-04	1421	SCAF1	Sphingomonadales	0.271	5.75E-04	1916	ZNF12	Ralstonia insidiosa	-0.214	5.53E-03
432	DCAF13	Ralstonia insidiosa	-0.297	5.46E-05	927	MID2	Bacillota	0.453	2.96E-03	1422	SCAMP1	Comamonadaceae	-0.318	5.21E-04	1917	ZNF121	Nitrobacteraceae	0.149	6.47E-05
433	DCAF15	Bacillota	-0.339	2.04E-03	928	MIDN	Sphingomonadales	0.408	3.57E-03	1423	SCARF1	Ralstonia insidiosa	0.210	6.89E-04	1918	ZNF132	Ottowia	0.237	7.91E-03
434	DCAF15	Sphingomonadales	0.291	1.25E-03	929	MIER3	Ralstonia insidiosa	-0.121	1.73E-14	1424	SCARF2	Ralstonia insidiosa	0.326	7.88E-03	1919	ZNF140	Viruses	0.385	4.22E-03
435	DCAF6	Archaea	-0.395	3.49E-03	930	MIF	Ottowia	-0.371	3.93E-11	1425	SCNN1D	Ralstonia insidiosa	0.224	2.31E-03	1920	ZNF141	Malassezia vespertilionis	0.366	3.80E-07
436	DCP1A	Corynebacterium accolens	0.444	4.00E-05	931	MINDY3	Chromatiaceae	0.240	1.64E-05	1426	SCOC	Ralstonia insidiosa	-0.208	6.93E-04	1921	ZNF146	Ralstonia insidiosa	-0.293	1.76E-03
437	DCP2	Ralstonia insidiosa	-0.209	1.51E-29	932	MINDY3	Ralstonia insidiosa	-0.239	3.29E-09	1427	SCT	Enterococcaceae	0.341	4.73E-04	1922	ZNF155	Uroviricota	0.092	2.84E-04
438	DCTN4	Ralstonia insidiosa	-0.237	1.27E-05	933	MINDY3	Sphingomonadaceae	-0.216	3.16E-03	1428	SCYL2	Ralstonia insidiosa	-0.162	5.11E-10	1923	ZNF181	Ralstonia insidiosa	-0.341	1.39E-08
439	DCUN1D1	Comamonadaceae	-0.295	2.21E-03	934	MINPP1	Ralstonia insidiosa	-0.305	2.52E-07	1429	SCYL2	Sphingomonadales	-0.291	5.76E-04	1924	ZNF184	Ralstonia insidiosa	-0.266	6.48E-04
440	DCUN1D3	Nitrobacteraceae	0.318	6.75E-04	935	MIOS	Ralstonia insidiosa	-0.344	5.57E-03	1430	SDF2L1	Archaea	0.502	3.40E-04	1925	ZNF189	Ralstonia insidiosa	-0.254	3.56E-03
441	DCUN1D4	Ralstonia insidiosa	-0.143	2.42E-03	936	MITD1	Prevotella melaninogenica	-0.368	3.57E-03	1431	SDR42E2	Sphingobacteriaceae	0.208	1.46E-03	1926	ZNF205	Ralstonia insidiosa	0.287	6.54E-04
442	DDA1	Ralstonia insidiosa	0.284	9.86E-03	937	MKLN1	Ralstonia insidiosa	-0.129	4.49E-03	1432	SEC22A	Ralstonia insidiosa	-0.245	1.49E-03	1927	ZNF219	Nitrobacteraceae	0.185	2.75E-05
443	DDAH1	Comamonadaceae	-0.303	6.34E-04	938	MKRN2OS	Archaea	0.282	1.55E-03	1433	SEC62	Comamonadaceae	-0.442	4.11E-06	1928	ZNF221	Corynebacterium accolens	0.187	1.26E-07
444	DDAH1	Ralstonia insidiosa	-0.115	4.15E-04	939	MKX	Corynebacterium durum	0.305	3.57E-04	1434	SELENOF	Ralstonia insidiosa	-0.313	2.89E-03	1929	ZNF233	Bacteria	-0.330	7.58E-04
445	DDIAS	Mycobacteroides	-0.187	1.50E-05	940	MLLT1	Ralstonia insidiosa	0.194	2.95E-03	1435	SELENON	Enterobacteriaceae	0.418	6.49E-03	1930	ZNF24	Ralstonia insidiosa	-0.326	7.97E-07
446	DDX49	Sphingomonadales	0.332	7.77E-11	941	MLLT1	Sphingomonadales	0.451	4.82E-05	1436	SELL	Actinomycetales	-0.397	6.50E-03	1931	ZNF268	Ralstonia insidiosa	-0.185	1.68E-15
447	DDX54	Ralstonia insidiosa	0.130	1.15E-05	942	MMADHC	Ralstonia insidiosa	-0.364	1.36E-08	1437	SEMA4B	Ralstonia insidiosa	0.144	3.11E-05	1932	ZNF280D	Chromatiaceae	0.252	1.88E-04
448	DEK	Comamonadaceae	-0.342	9.99E-03	943	MMADHC	Sphingomonadales	-0.378	7.89E-06	1438	SEMA4B	Sphingomonadales	0.255	6.41E-06	1933	ZNF316	Bacillota	-0.548	8.96E-05
449	DENND1A	Ralstonia insidiosa	0.187	6.43E-03	944	MMADHC	Xanthomonadaceae	-0.267	1.14E-21	1439	SEMA6A	Ralstonia insidiosa	0.355	7.06E-03	1934	ZNF383	Cloacibacterium normanense	-0.233	6.02E-03
450	DENND1B	Ralstonia insidiosa	-0.144	2.74E-03	945	MMGT1	Ralstonia insidiosa	-0.280	2.05E-03	1440	SEMA6B	Ralstonia insidiosa	0.289	1.28E-04	1935	ZNF420	Ralstonia insidiosa	-0.292	4.57E-03
451	DENND4A	Bacillota	0.401	9.85E-07	946	MMP24	Cloacibacterium normanense	0.074	2.93E-03	1441	SENP6	Ralstonia insidiosa	-0.205	2.74E-07	1936	ZNF428	Archaea	0.512	6.77E-07
452	DENND4A	Chromatiaceae	0.338	1.38E-05	947	MMRN1	Rothia mucilaginosa	-0.251	7.46E-04	1442	SENP7	Ralstonia insidiosa	-0.177	2.16E-04	1937	ZNF441	Ralstonia insidiosa	-0.144	4.09E-03
453	DERL1	Ralstonia insidiosa	-0.352	3.14E-03	948	MOB1B	Ralstonia insidiosa	-0.163	4.50E-03	1443	SEPSECS	Bacillota	0.448	1.94E-04	1938	ZNF443	Verrucomicrobiales	-0.322	9.26E-04
454	DGKZ	Sphaerotilaceae	0.271	4.70E-03	949	MORF4L1	Ottowia	0.481	7.73E-05	1444	SEPSECS	Ralstonia insidiosa	-0.314	6.98E-08	1939	ZNF444	Sphingomonadales	0.378	9.12E-03
455	DHFR	Viruses	0.333	3.00E-03	950	MORF4L2	Micromonospora	0.384	9.01E-04	1445	SEPTIN10	Bacillota	0.374	1.01E-03	1940	ZNF480	Archaea	-0.438	7.65E-03
456	DHX15	Bacillota	0.545	7.42E-03	951	MORF4L2	Ralstonia insidiosa	-0.208	1.44E-04	1446	SEPTIN9	Ralstonia insidiosa	0.202	1.84E-04	1941	ZNF500	Bacillota	-0.414	7.38E-03
457	DHX57	Epilithonimonas	-0.203	3.68E-03	952	MOSMO	Comamonadaceae	-0.268	1.26E-10	1447	SERBP1	Ralstonia insidiosa	-0.223	6.01E-04	1942	ZNF500	Ralstonia insidiosa	0.369	1.33E-03
458	DIAPH2	Bacillota	0.393	1.07E-06	953	MPST	Bacillota	-0.379	9.17E-03	1448	SERINC1	Bacillota	0.424	7.25E-05	1943	ZNF503	Staphylococcus saprophyticus	-0.260	6.97E-06
459	DIS3	Bacillota	0.484	7.97E-04	954	MPZL2	Ralstonia insidiosa	-0.229	1.33E-04	1449	SERPINA6	Agaricomycetes	-0.478	4.82E-03	1944	ZNF540	Pasteurellaceae	0.370	7.77E-03
460	DIS3	Ralstonia insidiosa	-0.232	1.62E-03	955	MR1	Micrococcales	0.406	7.02E-03	1450	SERPINB6	Brucella	-0.361	5.18E-03	1945	ZNF566	Malassezia vespertilionis	0.320	1.74E-05
461	DLG1	Ralstonia insidiosa	-0.245	6.15E-03	956	MRFAP1	Archaea	0.479	9.30E-04	1451	SETD5	Nitrobacteraceae	0.205	5.22E-05	1946	ZNF571	Ralstonia insidiosa	-0.362	8.56E-04
462	DLG4	Ralstonia insidiosa	0.407	1.71E-03	957	MRI1	Ralstonia insidiosa	0.334	5.05E-04	1452	SFMBT1	Viruses	0.370	5.13E-03	1947	ZNF598	Sphingomonadales	0.450	2.23E-04
463	DLL1	Ralstonia insidiosa	0.335	8.32E-03	958	MRM1	Sphingomonadales	0.359	1.46E-03	1453	SFT2D3	Rhodocyclales	0.057	4.85E-05	1948	ZNF626	Cloacibacterium caeni	-0.176	2.24E-06
464	DMTN	Ralstonia insidiosa	0.199	1.80E-04	959	MRNIP	Streptococcus oralis	-0.298	4.63E-03	1454	SGPP1	Comamonadaceae	-0.337	2.32E-08	1949	ZNF639	Ralstonia insidiosa	-0.252	7.79E-04
465	DNAJC10	Ralstonia insidiosa	-0.278	4.31E-03	960	MROH2B	Plantactinospora sp. BC1	0.294	6.74E-03	1455	SGSH	Bacillota	-0.402	4.10E-06	1950	ZNF646	Ralstonia insidiosa	0.218	2.77E-03
466	DNAJC19	Anoxybacillus	0.059	9.87E-03	961	MROH6	Bacillota	-0.451	1.42E-07	1456	SGSH	Ralstonia insidiosa	0.315	2.40E-09	1951	ZNF653	Ralstonia insidiosa	0.351	5.46E-06
467	DNAJC21	Ralstonia insidiosa	-0.080	1.04E-03	962	MRPL21	Viruses	-0.345	7.87E-03	1457	SGSM3	Sphingomonadales	0.252	5.00E-05	1952	ZNF653	Sphingomonadales	0.308	4.44E-03
468	DNM2	Ralstonia insidiosa	0.180	2.31E-04	963	MRPL27	Viruses	-0.333	1.32E-04	1458	SH3GL1	Ralstonia insidiosa	0.191	8.26E-03	1953	ZNF654	Ralstonia insidiosa	-0.131	7.99E-04
469	DNMT3A	Nitrobacteraceae	0.167	2.79E-03	964	MRPL32	Lactococcus	0.286	1.56E-03	1459	SH3GL1	Sphingomonadales	0.316	9.33E-03	1954	ZNF680	Chromatiaceae	0.349	8.58E-03
470	DOCK5	Chromatiaceae	0.228	5.47E-03	965	MRPL4	Bacillota	-0.327	6.29E-03	1460	SH3GL3	Gemella	0.278	2.53E-03	1955	ZNF710	Ralstonia insidiosa	0.359	9.12E-04
471	DOHH	Nitrobacteraceae	0.086	4.32E-03	966	MRPL4	Sphingomonadales	0.341	7.86E-03	1461	SH3GL3	Lactobacillus	0.282	1.85E-07	1956	ZNF721	Ralstonia insidiosa	-0.220	3.13E-03
472	DOK3	Ralstonia insidiosa	0.279	5.68E-03	967	MRPS5	Viruses	-0.403	3.44E-03	1462	SH3GL3	Ustilaginoidea virens	0.114	8.74E-08	1957	ZNF749	Hymenobacter	-0.186	2.43E-08
473	DOT1L	Sphingomonadales	0.373	6.25E-03	968	MRTFA	Ralstonia insidiosa	0.216	4.55E-06	1463	SH3GLB1	Ralstonia insidiosa	-0.267	4.39E-06	1958	ZNF782	Cloacibacterium caeni	-0.101	4.59E-04
474	DPP9	Bacillota	-0.332	3.76E-03	969	MRTFA	Sphingomonadales	0.254	8.45E-13	1464	SH3GLB2	Sphingomonadales	0.322	3.05E-05	1959	ZNF785	Ralstonia insidiosa	0.308	3.05E-03
475	DPP9	Sphingomonadales	0.395	1.55E-04	970	MTA1	Ralstonia insidiosa	0.219	2.01E-03	1465	SH3TC1	Sphingomonadales	0.402	7.88E-05	1960	ZNF837	Nitrobacteraceae	0.095	4.87E-06
476	DPY19L1	Saccharomycetales	0.450	2.85E-03	971	MTAP	Ralstonia insidiosa	-0.267	2.68E-28	1466	SHANK3	Ralstonia insidiosa	0.336	3.22E-03	1961	ZNF845	Sphingomonadales	-0.335	5.28E-04
477	DPY19L2	Paucibacter	0.212	7.94E-03	972	MTERF3	Ralstonia insidiosa	-0.273	6.66E-03	1467	SHISA9	Sphingobacterium	-0.278	1.91E-03	1962	ZNF865	Ralstonia insidiosa	0.411	3.00E-07
478	DPY19L3	Ralstonia insidiosa	-0.261	7.67E-04	973	MTF2	Cloacibacterium normanense	-0.146	1.28E-16	1468	SHKBP1	Ralstonia insidiosa	0.121	4.53E-03	1963	ZNF865	Sphingomonadales	0.396	9.06E-04
479	DPY19L4	Comamonadaceae	-0.265	4.70E-03	974	MTMR6	Ralstonia insidiosa	-0.256	3.75E-03	1469	SHLD1	Streptococcus oralis	-0.338	3.16E-03	1964	ZNF91	Cloacibacterium normanense	-0.143	3.63E-07
480	DPY19L4	Ralstonia insidiosa	-0.248	2.33E-03	975	MTRFR	Cloacibacterium normanense	-0.377	7.80E-05	1470	SHLD2	Ralstonia insidiosa	-0.271	3.12E-03	1965	ZNF92	Bacillota	0.398	1.71E-03
481	DPYD	Bacillota	0.407	8.97E-20	976	MTRR	Viruses	0.400	8.48E-03	1471	SHROOM1	Ralstonia insidiosa	0.312	2.66E-03	1966	ZNF92	Flavobacteriales	0.280	5.64E-04
482	DTWD1	Ralstonia insidiosa	-0.300	2.37E-06	977	MTTP	Bacillota	0.406	3.99E-03	1472	SHROOM1	Sphingomonadales	0.380	6.80E-04	1967	ZNRF2	Comamonadaceae	-0.435	3.21E-03
483	DYNC2I1	Nitrobacteraceae	0.160	6.60E-05	978	MTTP	Sphingomonadales	-0.461	9.19E-03	1473	SIGLEC11	Ralstonia insidiosa	0.218	3.92E-03	1968	ZRSR2	Streptococcus salivarius	0.058	5.45E-05

484	DYNLT3	Comamonadaceae	-0.367	8.55E-03	979	MVB12A	Sphingomonadales	0.362	4.80E-04	1474	SIGLEC12	Anaerococcus	-0.356	5.32E-03	1969	ZSCAN29	Anoxybacillus	-0.144	9.68E-03
485	DYNLT3	Ralstonia insidiosa	-0.161	1.72E-03	980	MVB12B	Cantharellales	-0.400	5.92E-03	1475	SIMC1	Brucella	0.205	2.98E-03	1970	ZSWIM1	Ralstonia insidiosa	0.370	1.52E-03
486	DYRK1B	Flavobacteriales	-0.323	5.40E-03	981	MYBBP1A	Ralstonia insidiosa	0.305	4.05E-06	1476	SIMC1	Paucibacter	0.173	1.70E-03	1971	ZSWIM4	Prevotella melaninogenica	-0.209	1.75E-04
487	DZIP3	Ralstonia insidiosa	-0.097	1.46E-04	982	MYBBP1A	Sphingomonadales	0.255	2.78E-08	1477	SIPA1	Ralstonia insidiosa	0.188	9.54E-03	1972	ZYX	Ralstonia insidiosa	0.254	1.40E-04
488	E2F4	Ralstonia insidiosa	0.312	1.02E-04	983	MYCN	Sphingomonadales	0.285	4.95E-03	1478	SIPA1L1	Viruses	0.407	5.55E-03	1973	ZYX	Sphingomonadales	0.310	7.77E-03
489	EAPP	Streptococcus oralis	-0.194	2.05E-09	984	MYH13	Acinetobacter johnsonii	0.236	4.95E-03	1479	SLC10A7	Bacillota	0.408	6.70E-03					
490	EBAG9	Lawsonellaceae	0.346	2.29E-05	985	MYH14	Ralstonia insidiosa	0.220	4.41E-03	1480	SLC11A2	Ralstonia insidiosa	-0.260	4.42E-03					
491	EBAG9	Ralstonia insidiosa	-0.320	3.30E-03	986	MYH7	Brevibacteriaceae	-0.073	1.99E-04	1481	SLC13A1	Ustilaginoidea virens	0.175	1.68E-04					
492	EBF2	Nevskiales	0.150	1.02E-03	987	MYL1	Brevibacteriaceae	-0.309	9.58E-07	1482	SLC16A10	Comamonadaceae	-0.320	2.95E-03					
493	EBI3	Prevotellaceae	-0.299	7.95E-03	988	MYL2	Brevibacteriaceae	0.004	5.38E-04	1483	SLC19A2	Chromatiaceae	0.231	8.14E-06					
494	ECPAS	Ralstonia insidiosa	-0.340	1.64E-03	989	MYNN	Ralstonia insidiosa	-0.232	8.14E-07	1484	SLC22A11	Nectriaceae	0.328	4.46E-03					
495	ECRG4	Streptococcus oralis	-0.297	3.78E-04	990	MYO1F	Ralstonia insidiosa	0.186	5.46E-03	1485	SLC22A24	Veillonella parvula	0.188	7.18E-03					

Supplementary Table 13: Individual level host gene-microbiota interaction results by LASSO in Borderline

ID	Gene	Taxa	Coeff	FDR	ID	Gene	Taxa	Coeff	FDR	ID	Gene	Taxa	Coeff	FDR	ID	Gene	Taxa	Coeff	FDR
1	AADAC	Schlegelella aquatica	-0.138	3.88E-03	367	DYNC2I2	Micromonosporaceae	-0.554	1.57E-04	733	MTERF2	Nitrobacteraceae	0.123	1.31E-04	1099	SH3PXD2B	Sphingomonadaceae	0.318	7.98E-09
2	AASDHPPT	Sphingomonadaceae	-0.569	6.83E-04	368	DYNLL1	Sphingosinicellaceae	0.232	3.39E-04	734	MTMR11	Schlegelella aquatica	0.174	9.47E-12	1100	SH3YL1	Intrasporangiaceae	0.162	5.56E-05
3	ABCB10	Micromonosporaceae	0.524	5.99E-03	369	DYNLT3	Sphingomonadaceae	-0.532	1.20E-09	735	MUC17	Brevundimonas sp.	0.135	1.24E-04	1101	SHANK1	Pseudomonas oleovorans	-0.315	1.88E-04
4	ABCE1	Micromonosporaceae	0.526	4.78E-03	370	DYRK1A	Sphingomonadaceae	-0.378	6.61E-03	736	MUC17	Pseudomonas oleovorans	-0.274	3.00E-03	1102	SHANK2	Sphingosinicellaceae	-0.401	4.77E-03
5	ABCE1	Sphingomonadaceae	-0.635	1.10E-04	371	DZANK1	Sphingomonas sp. FARSPH	-0.407	9.19E-03	737	MUC19	Pseudomonas oleovorans	-0.333	1.51E-03	1103	SHARPIN	Micromonosporaceae	-0.556	8.32E-03
6	ABHD13	Sphingomonadaceae	-0.591	4.37E-05	372	EARS2	Bacillaceae	0.373	4.79E-03	738	MUC2	Sphingomonas sp. FARSPH	-0.311	4.03E-03	1104	SHISA5	Lactobacillus	-0.255	9.16E-03
7	ABL1	Sphingomonadaceae	0.408	4.03E-03	373	ECEL1	Sphingomonas sp. FARSPH	-0.228	1.16E-10	739	MUC5AC	Pseudomonas oleovorans	-0.303	3.37E-06	1105	SHLD2	Chromatiales	-0.326	9.93E-03
8	ACAN	Pseudomonas oleovorans	-0.407	3.85E-03	374	ECHDC1	Caulobacterales	-0.423	8.11E-06	740	MUC5AC	Sphingomonas sp. FARSPH	-0.283	2.41E-06	1106	SHOC1	Sphingomonas sp. FARSPH	-0.383	5.18E-03
9	ACAT1	Methyloversatilis sp. RAC08	0.335	3.59E-07	375	ECHDC1	Sphaerotilaceae	-0.323	7.25E-13	741	MUTYH	Clostridiaceae	-0.524	4.98E-03	1107	SHOC2	Bacillales	0.489	9.98E-03
10	ACO2	Bacillaceae	0.358	8.97E-04	376	ECHS1	Methyloversatilis sp. RAC08	0.200	6.45E-03	742	MYBBP1A	Mycolicibacterium	-0.523	1.93E-04	1108	SIAH1	Bacillaceae	-0.371	1.43E-03
11	ACP5	Revtraviricetes	0.343	1.42E-04	377	EDRF1	Sphingomonadaceae	-0.313	3.63E-03	743	MYH13	Pseudomonas oleovorans	-0.235	4.48E-03	1109	SIGLEC14	Diaphorobacter	0.425	7.45E-03
12	ACTA2	Pseudomonas sp. CIP-10	0.241	4.98E-03	378	EEA1	Micromonosporaceae	0.552	5.26E-04	744	MYH15	Pseudomonas oleovorans	-0.291	6.11E-03	1110	SIPA1L1	Alcaligenaceae	-0.295	2.93E-05
13	ACTR6	Sphingomonadaceae	-0.553	5.20E-05	379	EFCAB14	Stutzerimonas	0.463	7.40E-03	745	MYH6	Pseudomonas oleovorans	-0.272	4.37E-03	1111	SIPA1L2	Lactobacillus	0.339	4.79E-03
14	ACVR2A	Sphingomonadaceae	-0.315	2.90E-03	380	EFNB1	Lactobacillus	-0.307	7.11E-05	746	MYNN	Hyphomicrobiales	0.486	8.51E-03	1112	SKP1	Cellulomonadaceae	0.503	2.66E-04
15	ADAM32	Pseudomonas oleovorans	-0.328	5.64E-04	381	EFR3B	Sphingomonas sp. FARSPH	-0.168	6.45E-03	747	MYO18B	Sphingomonas sp. FARSPH	-0.273	1.37E-03	1113	SLAIN2	Stutzerimonas	0.552	4.58E-03
16	ADAMTS5	Caulobacteraceae	-0.367	3.38E-04	382	EIF2S2	Cellulomonadaceae	0.373	6.94E-03	748	MYO3A	Sphingomonas sp. FARSPH	-0.321	4.57E-03	1114	SLAMF1	Staphylococcus capitis	-0.366	3.82E-03
17	ADAMTS5	Sphingomonadaceae	-0.383	6.36E-04	383	EIF3J	Sphingomonadaceae	-0.524	1.12E-08	749	MYO9B	Sphingomonadaceae	0.445	1.86E-03	1115	SLC22A15	Pseudomonas oleovorans	-0.217	6.43E-03
18	ADCY2	Sphingomonas sp. FARSPH	-0.309	7.56E-03	384	EIF3M	Cellulomonadaceae	0.364	4.52E-03	750	MYOM3	Pseudomonas oleovorans	-0.366	8.92E-03	1116	SLC24A1	Pichiaceae	-0.200	8.09E-03
19	ADGRB2	Pseudomonas oleovorans	-0.365	1.34E-03	385	ELFN2	Micromonospora	0.260	2.15E-12	751	MZF1	Sphaerotilaceae	0.451	2.30E-04	1117	SLC24A2	Sphingomonas sp. FARSPH	-0.178	4.84E-03
20	ADGRE1	Bacillus velezensis	0.340	3.64E-11	386	ELMOD2	Sphingomonadaceae	-0.505	4.59E-04	752	MZT2A	Micromonosporaceae	-0.520	8.62E-04	1118	SLC24A4	Sphingomonas sp. FARSPH	-0.171	1.05E-03
21	ADGRE1	Cytophagaceae	0.381	2.32E-05	387	EML4	Micromonosporaceae	0.527	6.85E-04	753	NAA15	Micromonosporaceae	0.565	4.40E-03	1119	SLC25A21	Veillonella parvula	-0.017	6.25E-04
22	ADPRHL1	Kocuria palustris	0.128	1.64E-04	388	EMP3	Corynebacterium durum	0.212	8.99E-04	754	NAA30	Caulobacterales	-0.514	6.94E-05	1120	SLC25A22	Sphingomonadaceae	0.373	8.55E-03
23	AEBP2	Caulobacterales	-0.427	9.88E-03	389	EOMES	Oxalobacteraceae	-0.235	2.29E-03	755	NAA30	Sphingomonadaceae	-0.612	2.90E-03	1121	SLC25A26	Cellulomonadaceae	0.250	8.12E-03
24	AEBP2	Sphingomonadaceae	-0.532	3.22E-03	390	EPHA5	Achromobacter deleyi	-0.330	5.88E-03	756	NAA50	Caulobacterales	-0.562	3.79E-06	1122	SLC25A32	Diaphorobacter	0.496	2.78E-03
25	AFF4	Sphingomonadaceae	-0.496	5.60E-15	391	EPHA5	Pseudomonas oleovorans	-0.368	1.44E-06	757	NAB1	Sphingomonadaceae	-0.435	9.92E-03	1123	SLC25A32	Micromonosporaceae	0.521	7.90E-03
26	AFG1L	Sphaerotilaceae	-0.314	1.17E-03	392	ERI3	Nevskiales	-0.278	4.08E-03	758	NCAPG	Acinetobacter johnsonii	0.268	4.59E-03	1124	SLC25A38	Bacillus velezensis	-0.475	8.76E-03
27	AFG3L2	Bacillaceae	0.402	4.17E-04	393	ERO1A	Caulobacterales	-0.419	8.06E-05	759	NCKAP1	Micromonosporaceae	0.602	2.80E-03	1125	SLC25A4	Cellulomonadaceae	0.364	7.69E-04
28	AFTPH	Micromonosporaceae	0.525	6.96E-04	394	ERO1A	Sphingomonadaceae	-0.454	4.67E-07	760	NDFIP2	Sphingomonadaceae	-0.618	1.76E-04	1126	SLC25A40	Sphingomonadaceae	-0.537	3.91E-04
29	AGBL1	Pseudomonas oleovorans	-0.314	1.92E-03	395	ESCO1	Sphingomonadaceae	-0.533	4.74E-05	761	NDUFA12	Cellulomonadaceae	0.413	3.87E-03	1127	SLC25A46	Caulobacterales	-0.571	2.67E-03
30	AGTR1	Sphingomonadaceae	-0.463	6.02E-04	396	ETAA1	Sphingomonadaceae	-0.509	7.87E-10	762	NDUFA4	Cellulomonadaceae	0.394	5.58E-03	1128	SLC25A46	Sphingomonadaceae	-0.623	9.35E-08
31	AHCYL2	Malassezia vespertilionis	-0.310	2.57E-05	397	ETFRF1	Sphingomonadaceae	-0.523	2.19E-06	763	NDUFA4L2	Prevotella melaninogenica	-0.415	9.76E-03	1129	SLC25A5	Cellulomonadaceae	0.398	2.52E-03
32	AHNAK	Cellulomonadaceae	-0.319	9.19E-06	398	ETNK1	Sphingomonadaceae	-0.456	2.77E-17	764	NDUFA5	Sphingomonadaceae	-0.455	5.09E-04	1130	SLC26A4	Sphingomonas sp. FARSPH	-0.113	8.29E-03
33	AJUBA	Verrucomicrobiota	0.293	4.01E-04	399	EVC	Lactobacillus	0.329	5.77E-03	765	NDUFA6	Cellulomonadaceae	0.342	3.06E-03	1131	SLC2A14	Streptococcus oralis	0.133	5.94E-03
34	AKAP11	Micromonosporaceae	0.540	3.26E-03	400	EWSR1	Streptococcus oralis	-0.044	3.61E-04	766	NDUFA8	Cellulomonadaceae	0.474	3.72E-04	1132	SLC30A1	Caulobacterales	-0.510	4.70E-04
35	ALDH16A1	Micromonosporaceae	-0.502	8.68E-03	401	EXOC5	Caulobacteraceae	-0.564	2.64E-05	767	NDUFAF4	Sphingosinicellaceae	0.277	4.42E-03	1133	SLC30A1	Sphingomonadaceae	-0.593	2.08E-04
36	ALDH18A1	Thermodesulfobacteriota	0.389	9.45E-03	402	EXOC5	Sphingomonadaceae	-0.582	6.31E-12	768	NDUFB5	Cellulomonadaceae	0.413	1.41E-03	1134	SLC30A5	Sphingomonadaceae	-0.556	1.20E-04
37	ALG5	Cellulomonadaceae	0.480	1.20E-05	403	EXOC8	Micromonosporaceae	0.451	2.66E-03	769	NDUFB6	Cellulomonadaceae	0.400	4.90E-03	1135	SLC35A1	Sphingomonadaceae	-0.386	2.35E-03
38	ALG5	Sphingosinicellaceae	0.458	1.92E-11	404	EXTL2	Sphingomonadaceae	-0.294	1.55E-04	770	NDUFC2	Sphingomonadaceae	-0.353	1.58E-04	1136	SLC35A3	Caulobacterales	-0.543	8.31E-10
39	ALG6	Hyphomicrobiales	0.418	1.25E-05	405	FAAP20	Micromonosporaceae	-0.514	1.91E-03	771	NDUFS4	Cellulomonadaceae	0.410	3.90E-03	1137	SLC35A3	Sphingomonadaceae	-0.573	5.35E-05
40	ALG6	Sphingomonadaceae	-0.567	1.39E-19	406	FAIM2	Pseudomonas oleovorans	-0.459	6.30E-03	772	NECTIN3	Sphaerotilaceae	-0.446	2.74E-04	1138	SLC35F4	Sphingomonas sp. FARSPH	-0.323	7.54E-03
41	ALKAL2	Intrasporangiaceae	0.183	2.98E-06	407	FAM107A	Thermales	-0.355	4.09E-03	773	NEXMIF	Sphingomonas sp. FARSPH	-0.269	5.19E-03	1139	SLC38A10	Sphingomonadaceae	0.387	2.86E-03
42	ANAPC13	Cellulomonadaceae	0.332	8.62E-04	408	FAM120C	Cellulomonadaceae	-0.295	9.68E-03	774	NFAT5	Cellulomonadaceae	-0.333	9.86E-11	1140	SLC38A6	Sphingomonadaceae	-0.289	5.57E-06
43	ANAPC13	Sphingomonadaceae	-0.360	3.88E-03	409	FAM149B1	Bacillaceae	0.530	9.16E-03	775	NFYB	Bacillus velezensis	-0.386	4.30E-03	1141	SLC38A7	Sphingomonadaceae	0.309	6.96E-03
44	ANKHD1	Sphingomonadaceae	-0.326	1.67E-03	410	FAM168A	Alcaligenaceae	-0.337	5.34E-07	776	NIPA2	Bacillaceae	0.554	6.54E-03	1142	SLC39A1	Nevskiales	-0.272	5.41E-04
45	ANKRD40	Stutzerimonas	0.358	3.95E-03	411	FAM168A	Chromatiaceae	0.245	2.75E-05	777	NKIRAS2	Revtraviricetes	0.272	3.12E-05	1143	SLC39A8	Sphingomonadaceae	-0.609	1.72E-15
46	ANKRD46	Sphingomonadaceae	-0.545	3.38E-03	412	FAM172A	Sphingomonadaceae	-0.492	7.56E-03	778	NKTR	Cellulomonadaceae	-0.450	3.18E-03	1144	SLC49A4	Bacillales	0.515	2.36E-03
47	ANKRD62	Staphylococcus saprophyticus	0.167	7.45E-03	413	FAM174A	Cellulomonadaceae	0.226	1.69E-03	779	NLE1	Chaetomiaceae	-0.374	4.43E-03	1145	SLC4A2	Sphingomonadaceae	0.474	3.74E-05
48	ANO4	Pseudomonas oleovorans	-0.347	1.74E-05	414	FAM174C	Micromonosporaceae	-0.510	7.32E-03	780	NLRP3	Bacillus velezensis	0.361	1.54E-03	1146	SLC5A2	Sphingomonas sp. FARSPH	-0.332	1.37E-03
49	ANP32E	Sphingomonadaceae	-0.507	1.37E-03	415	FAM186A	Sphingomonas sp. FARSPH	-0.364	1.69E-05	781	NME3	Micromonosporaceae	-0.574	2.05E-03	1147	SLC5A3	Micromonosporaceae	0.597	1.45E-03
50	ANTKMT	Micromonosporaceae	-0.510	6.17E-03	416	FAM193A	Cellulomonadaceae	-0.484	6.24E-03	782	NOC2L	Mycolicibacterium	-0.487	5.72E-03	1148	SLC5A7	Sphingomonas sp. FARSPH	-0.283	1.60E-03
51	ANTXRL	Sphingomonas sp. FARSPH	-0.267	1.75E-03	417	FAM199X	Sphingomonadaceae	-0.510	9.27E-03	783	NOC3L	Sphaerotilaceae	-0.431	5.49E-12	1149	SLC66A2	Micromonosporaceae	-0.460	5.20E-03

52	AP1AR	Sphingomonadaceae	-0.468	6.68E-03	418	FAM200B	Sphingomonadaceae	-0.471	2.59E-04	784	NOC4L	Micromonosporaceae	-0.529	1.14E-03	1150	SLC7A6OS	Rhizobiaceae	-0.378	2.01E-04
53	AP3B2	Sphingomonas sp. FARSPH	-0.363	7.65E-06	419	FAM234A	Sphingomonadaceae	0.570	2.09E-04	785	NOS1	Pseudomonas oleovorans	-0.215	7.08E-03	1151	SLC9A5	Sphingomonas sp. FARSPH	-0.444	2.59E-03
54	AP3M1	Micromonosporaceae	0.538	3.03E-03	420	FAM241B	Corynebacterium durum	0.209	6.03E-03	786	NOX1	Nocardiaceae	0.270	3.33E-03	1152	SLCO1B1	Micromonosporaceae	0.500	2.55E-04
55	AP3S1	Sphingomonadaceae	-0.491	6.49E-03	421	FAM43A	Nostocaceae	-0.300	7.09E-04	787	NOXRED1	Veillonella parvula	-0.258	7.28E-04	1153	SLCO1B3	Sphingomonadaceae	-0.450	1.20E-04
56	AP5B1	Sphingomonadaceae	0.392	3.90E-04	422	FAM53B	Pseudomonas sp. CIP-10	0.260	3.76E-04	788	NPHP3	Rhizobiaceae	0.244	4.20E-03	1154	SLCO1B3-	Malasseziales	0.378	9.74E-03
57	APBB2	Brevibacteriaceae	0.371	7.57E-03	423	FAM83B	Sphingomonas sp. FARSPH	-0.103	6.86E-03	789	NPIPB12	Cellulomonadaceae	-0.340	6.72E-03	1155	SLK	Micromonosporaceae	0.496	3.59E-03
58	APOBEC3F	Bacillus velezensis	0.285	2.83E-05	424	FAM8A1	Sphingomonadaceae	-0.475	2.06E-05	790	NPM3	Cellulomonadaceae	0.264	7.31E-03	1156	SMAD4	Chromatiales	-0.332	1.74E-04
59	APPBP2	Sphingomonadaceae	-0.571	2.56E-03	425	FAM98B	Caulobacterales	-0.488	7.42E-04	791	NR2C1	Sphingomonadaceae	-0.210	3.52E-05	1157	SMAD4	Sphingomonadaceae	-0.494	1.55E-04
60	APPL1	Micromonosporaceae	0.549	4.07E-03	426	FAM98B	Sphingomonadaceae	-0.506	3.30E-03	792	NR5A1	Pseudomonas oleovorans	-0.090	9.25E-04	1158	SMARCA4	Mycolicibacterium	-0.503	8.42E-03
61	ARFGEF1	Micromonosporaceae	0.557	8.31E-03	427	FAR1	Sphingomonadaceae	-0.334	8.92E-03	793	NSMCE1	Cellulomonadaceae	0.237	9.98E-03	1159	SMARCA5	Micromonosporaceae	0.480	2.07E-03
62	ARHGEF11	Cellulomonadaceae	-0.455	7.40E-04	428	FARP1	Alcaligenaceae	-0.385	4.13E-03	794	NSMCE4A	Bacillus velezensis	-0.475	4.42E-03	1160	SMARCAD1	Sphingomonadaceae	-0.525	2.69E-03
63	ARHGEF7	Cellulomonadaceae	-0.414	3.34E-03	429	FASTKD3	Sphingomonadaceae	-0.470	1.59E-04	795	NSRP1	Bacillus velezensis	-0.238	7.93E-03	1161	SMDT1	Cellulomonadaceae	0.237	7.91E-03
64	ARID1B	Cellulomonadaceae	-0.388	8.31E-03	430	FBRS	Sphaerotilaceae	0.382	8.67E-03	796	NSUN3	Sphingomonadaceae	-0.380	8.85E-03	1162	SMIM13	Sphingomonadales	-0.537	1.71E-03
65	ARIH1	Stutzerimonas	0.485	5.45E-04	431	FBXL17	Sphingomonadaceae	-0.560	1.31E-04	797	NT5C1A	Pseudomonas oleovorans	-0.243	3.22E-03	1163	SMIM15	Sphingomonadaceae	-0.563	2.99E-06
66	ARL15	Sphingomonadaceae	-0.485	6.53E-04	432	FBXL18	Sphingomonadaceae	0.384	2.54E-03	798	NT5C3A	Sphingomonadaceae	-0.406	1.18E-03	1164	SMNDC1	Sphingomonadaceae	-0.451	1.67E-05
67	ARL5A	Caulobacterales	-0.519	3.93E-03	433	FBXL3	Sphingomonadaceae	-0.608	1.38E-04	799	NTM	Pseudomonas oleovorans	-0.210	7.99E-04	1165	SMPD4	Gardnerella	-0.006	4.19E-03
68	ARL5A	Sphingomonadaceae	-0.609	8.42E-06	434	FBXO11	Sphingomonadaceae	-0.397	3.48E-04	800	NTN4	Lactobacillus	-0.308	7.93E-04	1166	SNAPC2	Micromonosporaceae	-0.471	1.19E-04
69	ARL5B	Hyphomicrobiales	0.358	2.41E-03	435	FBXO28	Sphingomonadaceae	-0.548	1.65E-04	801	NUCKS1	Caulobacterales	-0.584	3.48E-11	1167	SNAPC3	Sphingomonadaceae	-0.478	9.35E-03
70	ARL5B	Sphingomonadaceae	-0.595	6.92E-09	436	FCGR2B	Lactobacillus	-0.298	6.65E-03	802	NUDT21	Caulobacterales	-0.530	4.62E-03	1168	SNRNP48	Sphingomonadaceae	-0.468	3.46E-06
71	ARL6	Sphingomonadaceae	-0.481	9.14E-03	437	FCHO2	Micromonosporaceae	0.486	3.57E-03	803	NUDT21	Sphingomonadaceae	-0.631	3.95E-07	1169	SNX14	Sphingomonadaceae	-0.423	1.30E-04
72	ARL8B	Sphingomonadaceae	-0.572	8.69E-11	438	FCN2	Acinetobacter johnsonii	-0.076	1.89E-05	804	NUMA1	Bacteroidaceae	-0.342	5.63E-03	1170	SNX27	Stutzerimonas	0.449	3.72E-05
73	ARL8B	Streptococcaceae	-0.346	4.98E-03	439	FCN2	Lactobacillus	-0.344	4.24E-05	805	NUP54	Sphingomonadaceae	-0.443	5.81E-03	1171	SNX3	Sphingomonadaceae	-0.546	3.25E-03
74	ARMC1	Caulobacterales	-0.564	8.50E-07	440	FEM1B	Caulobacterales	-0.543	7.01E-03	806	NUS1	Caulobacterales	-0.511	6.24E-03	1172	SNX31	Sphingomonas sp. FARSPH	-0.263	2.60E-03
75	ARMC1	Sphingomonadaceae	-0.600	2.59E-09	441	FER1L5	Sphingomonas sp. FARSPH	-0.275	2.22E-03	807	NUTF2	Nevskiales	-0.286	5.35E-03	1173	SP1	Lautropia	0.322	1.85E-03
76	ARPP19	Micromonosporaceae	0.547	1.37E-03	442	FGF21	Micromonospora	0.241	1.11E-06	808	OAZ2	Cellulomonadaceae	0.337	1.72E-04	1174	SP140L	Cellulomonadaceae	-0.468	2.81E-04
77	ARPP19	Sphingomonadales	-0.542	5.63E-03	443	FHAD1	Sphingomonas sp. FARSPH	-0.215	4.41E-03	809	ODC1	Bacteroidaceae	0.295	5.44E-03	1175	SPACA7	Methylobacteriaceae	-0.239	7.15E-03
78	ARPP19	Stutzerimonas	0.504	4.99E-04	444	FLYWCH2	Micromonosporaceae	-0.535	3.04E-03	810	ODF3L2	Corynebacterium durum	0.084	3.40E-04	1176	SPAG9	Micromonosporaceae	0.469	7.37E-03
79	ART4	Bacillaceae	0.477	2.33E-04	445	FMN2	Sphingomonas sp. FARSPH	-0.275	1.34E-06	811	OMA1	Sphingomonadaceae	-0.489	1.24E-03	1177	SPAST	Bacillales	0.465	1.39E-04
80	ASCC2	Pseudomonas sp. CIP-10	0.118	1.24E-03	446	FMR1	Sphingomonadaceae	-0.504	8.71E-04	812	OPRM1	Pseudomonas oleovorans	-0.312	1.39E-03	1178	SPATA7	Bacillus velezensis	-0.366	2.67E-03
81	ASCC2	Sphingomonadaceae	0.475	3.20E-04	447	FNBP1L	Micromonosporaceae	0.543	1.09E-03	813	OR2M3	Archaea	0.352	1.76E-03	1179	SPCS1	Cellulomonadaceae	0.353	8.41E-03
82	ASF1A	Sphingomonadaceae	-0.496	1.62E-03	448	FOS	Pasteurellales	-0.202	8.57E-03	814	OR52H1	Nostocales	-0.286	1.21E-04	1180	SPCS3	Sphingomonadaceae	-0.482	2.20E-04
83	ATF3	Micromonospora	0.257	9.81E-03	449	FOXK1	Sphingomonadaceae	0.371	2.82E-06	815	OR6A2	Streptococcus thermophilus	-0.224	5.59E-03	1181	SPEN	Cellulomonadaceae	-0.428	2.71E-04
84	ATG12	Sphingomonadaceae	-0.509	8.21E-07	450	FOXN2	Sphingomonadaceae	-0.441	1.74E-03	816	ORC4	Sphingomonadaceae	-0.507	1.33E-05	1182	SPNS1	Sphingomonadaceae	0.387	8.56E-03
85	ATG16L1	Nostocaceae	-0.318	7.76E-03	451	FOXP4	Sphingomonadaceae	0.499	1.79E-03	817	OSBPL8	Micromonosporaceae	0.523	4.45E-03	1183	SPP2	Cellulomonadaceae	0.297	4.77E-03
86	ATG2A	Sphingomonadaceae	0.451	7.86E-03	452	FPGT	Sphingomonadaceae	-0.567	1.75E-09	818	OSTF1	Cellulomonadaceae	0.405	3.11E-04	1184	SPTAN1	Mycobacteroides	-0.306	6.58E-03
87	ATG4C	Sphingomonadaceae	-0.574	1.09E-06	453	FRMD5	Pseudomonas oleovorans	-0.372	1.57E-03	819	OSTM1	Cyanobacteriota	-0.426	4.07E-04	1185	SRCAP	Cellulomonadaceae	-0.436	9.25E-04
88	ATL2	Sphingomonadaceae	-0.514	7.11E-05	454	FRMD8	Sphingomonadaceae	0.372	7.50E-04	820	OTOG	Achromobacter deleyi	-0.326	1.01E-04	1186	SREK1	Sphingomonadaceae	-0.270	3.96E-03
89	ATP1A1	Bacillaceae	0.366	1.58E-03	455	FUT9	Pseudomonas oleovorans	-0.324	2.41E-03	821	OVGP1	Bacillus velezensis	-0.422	9.25E-04	1187	SRP9	Sphingomonadaceae	-0.537	3.62E-11
90	ATP5F1B	Cellulomonadaceae	0.462	5.18E-10	456	FXYD5	Nevskiales	-0.386	9.73E-03	822	OVOL1	Brevundimonas sp.	0.144	2.55E-05	1188	SRRD	Nostocaceae	0.413	1.90E-03
91	ATP5F1B	Schlegelella aquatica	-0.074	9.86E-04	457	FZD7	Staphylococcus capitis	-0.226	4.26E-09	823	OXLD1	Micromonosporaceae	-0.521	3.62E-03	1189	SRRM2	Cellulomonadaceae	-0.496	1.94E-03
92	ATP5MC3	Cellulomonadaceae	0.504	4.18E-05	458	G2E3	Sphingomonadaceae	-0.580	5.75E-03	824	OXNAD1	Ralstonia insidiosa	-0.395	6.75E-03	1190	SRRM4	Lautropia	-0.423	4.99E-04
93	ATP5MG	Cellulomonadaceae	0.286	1.75E-03	459	G3BP2	Micromonosporaceae	0.551	1.45E-03	825	PABIR2	Rhizobiaceae	0.272	6.43E-04	1191	SRSF1	Sphingomonadaceae	-0.457	4.74E-24
94	ATP5MK	Cellulomonadaceae	0.417	2.83E-04	460	GABARAP	Cellulomonadaceae	0.391	5.30E-03	826	PAIP1	Cellulomonadaceae	0.452	3.12E-05	1192	SRSF10	Sphingomonadaceae	-0.550	1.17E-07
95	ATP5PB	Cellulomonadaceae	0.441	4.27E-03	461	GABPB1	Sphingomonadaceae	-0.517	4.23E-04	827	PALS2	Caulobacterales	-0.465	4.57E-03	1193	SRSF2	Micrococcaceae	0.382	1.50E-17
96	ATP5PO	Cellulomonadaceae	0.462	1.67E-04	462	GABRA4	Brevundimonas sp.	0.077	1.74E-04	828	PALS2	Nevskiales	0.391	2.19E-03	1194	SSH1	Cellulomonadaceae	-0.316	6.85E-04
97	ATP6V0E1	Cellulomonadaceae	0.307	5.07E-03	463	GABRA4	Pseudomonas oleovorans	-0.258	1.93E-03	829	PAN3	Brevibacteriaceae	0.309	6.42E-14	1195	SSTR5	Methylobacterium sp. FF17	0.198	1.79E-03
98	ATP6V1G1	Sphingosinicellaceae	0.352	1.48E-19	464	GABRB3	Intrasporangiaceae	0.298	4.21E-03	830	PANX1	Bacillaceae	0.480	5.18E-03	1196	ST6GALNAC2	Plantactinospora	-0.299	4.79E-03
99	ATP9B	Malassezia vespertilionis	-0.304	3.39E-06	465	GABRQ	Sphingomonas sp. FARSPH	-0.223	5.17E-03	831	PAPOLA	Sphingomonadaceae	-0.588	4.39E-09	1197	STARD3NL	Gordonia bronchialis	0.423	2.33E-03
100	ATXN2	Cellulomonadaceae	-0.501	2.24E-03	466	GALNS	Sphingomonadaceae	0.490	1.75E-03	832	PARG	Spirosomaceae	0.387	1.86E-03	1198	STK24	Nitrobacteraceae	-0.116	3.37E-04
101	ATXN7L1	Xanthobacteraceae	-0.302	8.53E-04	467	GALNT13	Pseudomonas oleovorans	-0.283	3.22E-03	833	PARP10	Mycolicibacterium	-0.479	7.67E-03	1199	STK3	Sphingomonadaceae	-0.427	9.21E-03
102	AURKAIP1	Stutzerimonas	-0.505	2.29E-03	468	GALNT15	Mycobacteriales	-0.197	6.71E-03	834	PAWR	Sphingomonadaceae	-0.481	1.56E-04	1200	STK31	Nitrobacteraceae	0.087	1.53E-03
103	B3GALT1	Pseudomonas oleovorans	-0.336	3.08E-03	469	GALR1	Sphingomonas sp. FARSPH	-0.283	1.26E-03	835	PCDH10	Sphingomonas sp. FARSPH	-0.255	6.36E-04	1201	STK33	Pseudomonas oleovorans	-0.465	7.06E-03
104	B3GAT3	Micromonosporaceae	-0.477	5.23E-03	470	GAN	Brevibacteriaceae	0.269	6.22E-03	836	PCDH15	Achromobacter deleyi	-0.328	4.87E-04	1202	STK4	Micromonosporaceae	0.465	8.68E-03
105	B4GALNT4	Corynebacterium kefirresidenti	-0.071	7.00E-03	471	GANC	Cellulomonadaceae	-0.363	7.03E-04	837	PCDH15	Pseudomonas oleovorans	-0.293	1.26E-03	1203	STPG4	Streptococcus salivarius	0.130	9.91E-03

106	BAP1	Sphingomonadaceae	0.485	4.06E-03	472	GAR1	Sphingomonadaceae	-0.384	7.76E-03	838	PCGF5	Caulobacterales	-0.548	4.44E-04	1204	STRN3	Sphingomonadaceae	-0.470	8.47E-03
107	BAZ2A	Cellulomonadaceae	-0.452	1.39E-05	473	GARNL3	Haemophilus parainfluenzae	0.113	4.93E-03	839	PCIF1	Sphingomonadaceae	0.585	3.15E-04	1205	STRN4	Sphingomonadaceae	0.496	1.06E-03
108	BBLN	Micromonosporaceae	-0.553	1.03E-04	474	GATAD2B	Cellulomonadaceae	-0.461	6.03E-04	840	PCMT1	Sphingosinicellaceae	0.401	3.31E-04	1206	STXBP5L	Sphingomonas sp. FARSPH	-0.302	7.41E-03
109	BBOF1	Brevibacteriaceae	0.301	9.95E-06	475	GATD1	Sphingomonadaceae	0.416	3.93E-03	841	PCNP	Sphingomonadaceae	-0.526	5.04E-04	1207	STYX	Caulobacterales	-0.521	1.28E-04
110	BCAM	Sphingomonadaceae	0.409	8.62E-03	476	GATM	Pseudomonas sp. CIP-10	-0.159	4.10E-03	842	PCNT	Mycobacteroides	-0.445	2.05E-03	1208	STYX	Sphingomonadaceae	-0.607	6.85E-05
111	BCLAF1	Sphingomonadaceae	-0.598	2.52E-05	477	GBA3	Bacillaceae	0.404	5.41E-04	843	PCNX3	Sphingomonadaceae	0.373	2.74E-03	1209	SUB1	Cellulomonadaceae	0.416	2.72E-03
112	BCR	Sphingomonadaceae	0.497	3.22E-04	478	GBE1	Lactobacillus iners	0.373	7.93E-04	844	PCSK4	Sphaerotilaceae	0.442	7.86E-03	1210	SUGP1	Lactobacillus iners	-0.464	7.45E-03
113	BEND5	Pseudomonas oleovorans	-0.428	6.27E-03	479	GBF1	Cellulomonadaceae	-0.291	4.21E-03	845	PCSK6	Bacillaceae	0.426	9.41E-03	1211	SUMO3	Cellulomonadaceae	0.400	4.18E-03
114	BEST1	Corynebacterium tuberculoste	-0.379	4.66E-03	480	GCFC2	Sphingomonadaceae	-0.253	5.97E-04	846	PDE6A	Streptococcus sanguinis	0.161	1.17E-05	1212	SUPT20H	Ottowia	-0.333	8.80E-03
115	BIRC2	Sphingomonadaceae	-0.407	1.41E-06	481	GDA	Sphingomonadaceae	-0.272	3.12E-04	847	PDE6B	Pseudomonas oleovorans	-0.316	5.23E-03	1213	SUPT6H	Cellulomonadaceae	-0.338	8.02E-04
116	BMP3	Lawsonella clevelandensis	0.246	2.58E-04	482	GEN1	Chromatiales	-0.377	6.33E-03	848	PDIA6	Bacillaceae	0.418	2.45E-03	1214	SURF6	Stutzerimonas	-0.476	1.54E-03
117	BMPR1A	Sphaerotilaceae	-0.423	2.02E-04	483	GIN1	Sphingomonadaceae	-0.410	4.78E-03	849	PDPR	Cellulomonadaceae	-0.331	3.68E-03	1215	SUV39H1	Nevskiales	-0.362	3.56E-04
118	BMPR2	Stutzerimonas	0.523	2.45E-03	484	GIPC2	Caulobacterales	-0.442	9.85E-03	850	PET117	Sphingomonadaceae	-0.369	6.59E-04	1216	SUV39H2	Sphingomonadales	-0.561	1.06E-03
119	BMT2	Sphingomonadaceae	-0.594	4.67E-06	485	GIT2	Cellulomonadaceae	-0.282	1.32E-03	851	PFDN4	Cellulomonadaceae	0.392	4.58E-03	1217	SWT1	Sphingomonadaceae	-0.394	7.73E-03
120	BNIP2	Sphingomonadaceae	-0.578	1.28E-03	486	GKAP1	Achromobacter	0.223	3.91E-04	852	PGGT1B	Caulobacterales	-0.543	7.70E-04	1218	SYMPK	Mycolicibacterium	-0.488	1.94E-03
121	BNIP3	Sphingomonadaceae	-0.361	9.83E-04	487	GKAP1	Bacillus velezensis	-0.375	5.23E-04	853	PGGT1B	Sphingomonadaceae	-0.604	3.66E-06	1219	SYMPK	Sphingomonadaceae	0.528	6.07E-03
122	BOD1L1	Cellulomonadaceae	-0.186	1.97E-05	488	GLDN	Pseudomonas oleovorans	-0.320	3.36E-04	854	PGM2	Bacillaceae	0.413	2.45E-05	1220	SYNCRIP	Caulobacterales	-0.570	7.03E-03
123	BORCS7	Sphingomonadaceae	-0.457	3.39E-05	489	GLMN	Sphingomonadaceae	-0.321	9.85E-06	855	PHF21A	Sphingomonas hankookensis	-0.451	2.48E-03	1221	SYNDIG1L	Sphingomonas sp. FARSPH	-0.206	2.52E-03
124	BRF1	Mycolicibacterium	-0.440	5.17E-03	490	GM2A	Sphingomonas hankookensis	0.378	1.06E-04	856	PHF21B	Pseudomonas oleovorans	-0.319	5.40E-04	1222	SYNGR1	Sphingomonas hankookensis	-0.338	8.50E-03
125	BROX	Micromonosporaceae	0.503	8.92E-04	491	GMFB	Sphingomonadaceae	-0.664	1.48E-06	857	PHIP	Sphingomonadaceae	-0.524	7.19E-03	1223	SYNPO	Sphingomonadaceae	0.381	2.93E-03
126	BROX	Sphingomonadaceae	-0.613	2.44E-05	492	GNA11	Sphingomonadaceae	0.471	1.54E-03	858	PI4K2B	Pseudomonas sp. CIP-10	-0.153	8.10E-03	1224	SYT16	Pseudomonas oleovorans	-0.288	1.63E-04
127	BTF3	Cellulomonadaceae	0.426	1.30E-03	493	GNAI2	Nevskiales	-0.272	6.79E-03	859	PIAS1	Stutzerimonas	0.492	8.17E-03	1225	SYT16	Sphingomonas sp. FARSPH	-0.209	7.89E-03
128	BTF3L4	Sphingomonadaceae	-0.535	7.00E-04	494	GNAQ	Sphingomonadales	-0.625	1.30E-04	860	PICALM	Micromonosporaceae	0.570	6.74E-03	1226	SYT6	Pseudomonas oleovorans	-0.353	1.29E-03
129	BZW1	Caulobacterales	-0.556	4.58E-05	495	GNAZ	Achromobacter deleyi	-0.373	6.05E-03	861	PIGK	Caulobacterales	-0.464	4.14E-03	1227	TACC2	Sphingomonadaceae	0.399	1.00E-03
130	C11orf80	Streptococcus oralis	0.221	4.27E-03	496	GOLGA1	Cellulomonadaceae	-0.387	9.90E-03	862	PIGO	Bacillaceae	0.352	9.08E-03	1228	TAGLN	Pseudomonas sp. CIP-10	0.161	4.32E-03
131	C12orf29	Pseudomonas sp. CIP-10	-0.181	9.75E-03	497	GOLGA1	Gordonia bronchialis	-0.272	1.22E-06	863	PIGW	Sphaerotilaceae	-0.419	2.62E-03	1229	TAPT1	Sphingomonadaceae	-0.344	5.02E-03
132	C12orf29	Sphingomonadaceae	-0.487	1.41E-03	498	GOLGB1	Cellulomonadaceae	-0.200	2.74E-03	864	PIK3C2A	Micromonosporaceae	0.520	2.82E-03	1230	TBC1D12	Sphingomonadaceae	-0.363	3.40E-03
133	C14orf28	Sphingomonadaceae	-0.261	3.55E-06	499	GOLT1B	Sphingomonadaceae	-0.580	1.28E-07	865	PINX1	Kocuria palustris	0.106	6.49E-05	1231	TBL1XR1	Sphingomonadaceae	-0.517	2.26E-18
134	C14orf39	Brevundimonas sp.	0.167	8.76E-04	500	GON4L	Cellulomonadaceae	-0.386	5.25E-06	866	PJA2	Micromonosporaceae	0.510	2.05E-03	1232	TCEA1	Caulobacterales	-0.623	2.08E-06
135	C14orf39	Sphingomonas sp. FARSPH	-0.175	8.14E-04	501	GON7	Cellulomonadaceae	0.260	1.57E-03	867	PLA2G10	Brevundimonas sp.	0.339	6.97E-05	1233	TCEA1	Sphingomonadaceae	-0.561	4.14E-05
136	C16orf74	Sphingomonas hankookensis	-0.308	5.64E-08	502	GOPC	Sphingomonadaceae	-0.514	1.03E-03	868	PLCL2	Brevundimonas sp.	0.443	4.15E-03	1234	TCEAL8	Cellulomonadaceae	0.404	8.38E-03
137	C16orf86	Micromonosporaceae	-0.511	1.30E-03	503	GORASP2	Schlegelella aquatica	-0.236	2.26E-07	869	PLEKHF2	Sphingomonadaceae	-0.624	9.25E-14	1235	TEDC1	Micromonosporaceae	-0.555	7.72E-04
138	C16orf96	Sphingomonas sp. FARSPH	-0.313	6.48E-03	504	GPATCH4	Archaea	0.355	1.00E-03	870	PLEKHG7	Lautropia	-0.319	3.77E-03	1236	TENT2	Sphingomonadaceae	-0.538	3.31E-06
139	C17orf80	Lautropia	0.265	6.38E-03	505	GPATCH8	Cellulomonadaceae	-0.345	1.37E-14	871	PLEKHH2	Brucella	-0.261	1.53E-04	1237	TERT	Streptococcus salivarius	0.262	4.95E-10
140	C19orf18	Peptoniphilaceae	0.193	3.22E-03	506	GPD2	Streptococcaceae	-0.392	7.03E-03	872	PLEKHM2	Sphingomonadaceae	0.382	3.47E-12	1238	TEX101	Archaea	0.353	9.76E-05
141	C19orf25	Micromonosporaceae	-0.531	4.90E-03	507	GPR137	Sphingomonadaceae	0.497	1.52E-03	873	PLIN2	Micromonospora	0.308	6.54E-04	1239	TEX101	Coriobacteriia	-0.326	5.77E-03
142	C19orf53	Cellulomonadaceae	0.242	8.10E-03	508	GPR176	Staphylococcus capitis	-0.224	7.37E-03	874	PLOD1	Sphingomonadaceae	0.402	1.75E-03	1240	TFAM	Caulobacterales	-0.548	2.53E-09
143	C1GALT1	Sphingomonadaceae	-0.508	7.26E-05	509	GPR182	Ralstonia insidiosa	0.325	5.12E-03	875	PLOD1	Thermodesulfobacteriota	0.192	2.60E-03	1241	TFAM	Micromonosporaceae	0.507	1.49E-03
144	C1orf159	Micromonosporaceae	-0.575	7.41E-04	510	GRAMD1A	Sphingomonadaceae	0.502	1.76E-03	876	PLOD3	Sphingomonadaceae	0.508	9.86E-04	1242	TFAM	Sphingomonadaceae	-0.584	5.21E-04
145	C21orf58	Sphingomonas hankookensis	-0.391	3.32E-03	511	GRIA1	Pseudomonas oleovorans	-0.268	9.52E-03	877	PLPP3	Nitrobacteraceae	-0.096	3.96E-03	1243	TFAP2E	Brucella	-0.396	7.67E-03
146	C2CD5	Sphingomonadaceae	-0.509	2.97E-03	512	GRID2	Pseudomonas oleovorans	-0.430	7.38E-04	878	PLPP7	Sphingomonas sp. FARSPH	-0.387	1.44E-03	1244	TFF3	Pseudomonas oleovorans	-0.331	2.93E-03
147	C2orf49	Sphingomonadaceae	-0.494	6.15E-03	513	GRIN2B	Sphingomonas sp. FARSPH	-0.268	4.45E-03	879	PLSCR2	Pseudomonas oleovorans	-0.176	1.44E-03	1245	TG	Pseudomonas oleovorans	-0.354	8.44E-03
148	C3orf62	Bacillaceae	-0.413	7.72E-04	514	GRIP1	Sphingomonas sp. FARSPH	-0.281	1.41E-03	880	PLXNB2	Sphingomonadaceae	0.420	1.76E-03	1246	TG	Sphingomonas sp. FARSPH	-0.277	7.20E-03
149	C4orf3	Cellulomonadaceae	0.433	1.31E-04	515	GRIPAP1	Mycolicibacterium	-0.582	2.99E-03	881	PNKP	Stutzerimonas	-0.611	7.98E-03	1247	TGFBI	Bacillaceae	0.249	5.43E-03
150	C5orf24	Micromonosporaceae	0.500	5.84E-04	516	GRK4	Clostridiaceae	-0.434	6.81E-03	882	PNPLA2	Bacillaceae	-0.488	6.67E-03	1248	TGIF1	Ustilaginaceae	-0.364	2.32E-04
151	C5orf46	Pseudomonas oleovorans	-0.236	3.54E-03	517	GRM1	Sphingomonas sp. FARSPH	-0.259	4.61E-03	883	PNRC1	Sphingomonadaceae	-0.310	5.08E-03	1249	THAP2	Sphingomonadaceae	-0.479	9.52E-03
152	C6orf120	Caulobacterales	-0.546	2.22E-03	518	GRPEL1	Cellulomonadaceae	0.418	9.47E-03	884	PNRC2	Sphingomonadaceae	-0.547	1.29E-04	1250	THAP5	Sphingomonadaceae	-0.550	1.63E-05
153	C6orf120	Sphingomonadaceae	-0.573	8.58E-09	519	GSK3A	Brevibacteriaceae	-0.341	5.03E-03	885	PODNL1	Pseudomonas oleovorans	-0.394	1.28E-03	1251	THAP9	Sphingomonadaceae	-0.343	2.82E-03
154	C6orf62	Chromatiales	-0.366	2.18E-03	520	GSK3A	Sphingomonadaceae	0.375	6.49E-03	886	POGZ	Gordonia bronchialis	-0.343	5.55E-04	1252	TIAL1	Bacillus velezensis	-0.408	5.08E-07
155	C6orf62	Sphingomonadaceae	-0.431	8.17E-04	521	GSTA1	Methyloversatilis sp. RAC08	0.196	1.44E-03	887	POLD4	Micromonosporaceae	-0.496	3.28E-03	1253	TIPRL	Sphingomonadaceae	-0.614	4.84E-08
156	C8G	Micromonosporaceae	-0.501	4.42E-04	522	GSTCD	Bacillaceae	0.545	8.61E-04	888	POLQ	Acinetobacter johnsonii	0.122	8.39E-03	1254	TKT	Streptococcus thermophilus	-0.227	8.83E-03
157	CAAP1	Nitrobacteraceae	0.177	5.20E-09	523	GSTO1	Cellulomonadaceae	0.380	2.32E-03	889	POLR1F	Sphingomonadaceae	-0.563	3.21E-03	1255	TLK2	Nostocales	-0.346	5.82E-03
158	CAAP1	Sphingomonadaceae	-0.413	4.52E-19	524	GTF2A1	Caulobacterales	-0.493	1.30E-04	890	POLR2A	Cellulomonadaceae	-0.426	1.81E-03	1256	TLN1	Nitrobacteraceae	-0.290	3.68E-03
159	CABIN1	Cellulomonadaceae	-0.431	3.79E-03	525	GTF2IRD1	Sphingomonadaceae	0.429	4.18E-03	891	POMP	Cellulomonadaceae	0.380	3.93E-03	1257	TM7SF3	Nostocaceae	0.362	9.38E-03

160	CACNA1B	Brevundimonas sp.	0.185	2.10E-09	526	GTPBP10	Sphingomonadaceae	-0.416	4.74E-03	892	PPA2	Cellulomonadaceae	0.476	2.01E-03	1258	TM9SF3	Caulobacterales	-0.580	4.97E-05
161	CACNA1B	Pseudomonas oleovorans	-0.279	1.63E-04	527	GUF1	Sphingomonadaceae	-0.420	3.64E-03	893	PPDPF	Sphingomonadaceae	0.570	6.20E-04	1259	TM9SF3	Cyanobacteriota	-0.464	4.27E-05
162	CACNA1E	Pseudomonas oleovorans	-0.377	8.82E-03	528	H1-5	Acinetobacter johnsonii	0.131	1.28E-09	894	PPFIA4	Pseudomonas oleovorans	-0.394	6.51E-03	1260	TM9SF3	Sphingomonadaceae	-0.641	2.37E-07
163	CACNA1E	Sphingomonas sp. FARSPH	-0.343	5.73E-03	529	H2AZ1	Cellulomonadaceae	0.428	2.30E-03	895	PPID	Sphingomonadaceae	-0.516	8.67E-05	1261	TMA16	Sphingomonadaceae	-0.426	3.50E-03
164	CACNA1H	Intrasporangiaceae	-0.366	4.03E-03	530	H4C2	Nocardioides	0.207	5.39E-05	896	PPIL6	Lautropia	-0.195	3.78E-04	1262	TMA7	Cellulomonadaceae	0.205	9.95E-03
165	CALCR	Sphingomonas sp. FARSPH	-0.120	6.81E-05	531	HBD	Leptotrichiaceae	0.121	9.32E-03	897	PPL	Cellulomonadaceae	-0.417	8.01E-03	1263	TMBIM4	Sphingomonadaceae	-0.538	2.48E-03
166	CAMTA2	Sphingomonadaceae	0.533	7.96E-03	532	HCFC1R1	Micromonosporaceae	-0.488	6.91E-03	898	PPM1B	Nevskiales	0.377	2.52E-03	1264	TMCC3	Bacillus velezensis	0.358	3.26E-03
167	CAPN1	Sphingomonadaceae	0.396	2.37E-03	533	HCFC2	Micromonosporaceae	0.537	8.79E-03	899	PPP1CB	Caulobacterales	-0.514	9.92E-07	1265	TMED5	Sphingomonadaceae	-0.612	5.42E-05
168	CAPN14	Lautropia	-0.370	4.83E-03	534	HDAC10	Mycolicibacterium	-0.572	4.63E-04	900	PPP1CB	Sphingomonadaceae	-0.596	7.58E-08	1266	TMED7	Sphingomonadaceae	-0.589	2.32E-06
169	CAPN7	Micromonosporaceae	0.501	5.25E-03	535	HDLBP	Alcaligenaceae	-0.375	1.89E-10	901	PPP1R11	Nevskiales	-0.396	1.31E-04	1267	TMEM134	Micromonosporaceae	-0.483	4.79E-03
170	CAPZA3	Alphaproteobacteria	-0.455	7.18E-08	536	HECTD4	Cellulomonadaceae	-0.468	8.65E-04	902	PPP1R12A	Sphingomonadaceae	-0.514	8.35E-03	1268	TMEM135	Sphingomonadaceae	-0.547	2.60E-03
171	CAPZA3	Bacillus velezensis	-0.391	8.95E-03	537	HECTD4	Sphingosinicellaceae	-0.431	5.73E-03	903	PPP1R12C	Micromonosporaceae	-0.530	8.32E-06	1269	TMEM14B	Cellulomonadaceae	0.418	1.48E-03
172	CARF	Sphingomonadaceae	-0.414	3.91E-03	538	HECW1	Pseudomonas oleovorans	-0.263	9.00E-03	904	PPP1R12C	Sphaerotilaceae	0.388	3.37E-04	1270	TMEM161B	Sphingomonadaceae	-0.564	1.92E-05
173	CARM1	Sphingomonadaceae	0.226	8.44E-03	539	HEPHL1	Pseudomonas oleovorans	-0.276	5.22E-03	905	PPP1R2	Sphingomonadaceae	-0.523	1.58E-03	1271	TMEM167A	Caulobacterales	-0.555	1.92E-03
174	CARNMT1	Caulobacterales	-0.487	4.95E-03	540	HERC2	Cellulomonadaceae	-0.387	5.42E-10	906	PPP1R35	Spirosomaceae	-0.376	7.78E-03	1272	TMEM167A	Sphingomonadaceae	-0.644	1.99E-05
175	CARNMT1	Sphingomonadaceae	-0.541	8.20E-05	541	HIGD1A	Cellulomonadaceae	0.448	1.90E-03	907	PPP1R37	Sphingomonadaceae	0.523	6.01E-03	1273	TMEM168	Sphingomonadaceae	-0.491	1.48E-07
176	CASD1	Sphingomonadaceae	-0.575	4.64E-06	542	HMG20B	Micromonosporaceae	-0.587	5.49E-03	908	PPP2R5A	Sphingomonadaceae	-0.294	1.25E-05	1274	TMEM230	Sphingosinicellaceae	0.329	1.23E-03
177	CATSPERG	Sphingomonas sp. FARSPH	-0.256	2.41E-03	543	HMGB1	Sphingomonadaceae	-0.465	1.44E-04	909	PPP2R5E	Sphingomonadaceae	-0.523	3.46E-03	1275	TMEM263	Sphingomonadaceae	-0.465	1.26E-06
178	CBFB	Sphingomonadaceae	-0.500	1.36E-03	544	HNRNPA2B1	Nevskiales	0.261	1.01E-03	910	PPP3CB	Sphingomonadaceae	-0.461	1.72E-04	1276	TMEM30A	Caulobacterales	-0.537	2.17E-03
179	CBLC	Spirosomaceae	-0.359	2.21E-04	545	HNRNPA3	Sphingomonadaceae	-0.525	4.13E-04	911	PPP4R2	Caulobacterales	-0.529	6.29E-03	1277	TMEM30A	Sphingomonadales	-0.508	1.93E-03
180	CBLL1	Sphingomonadaceae	-0.536	2.72E-03	546	HOMER1	Sphingomonas sp. FARSPH	-0.163	4.15E-04	912	PPP4R2	Sphingomonadaceae	-0.578	3.09E-04	1278	TMEM33	Chromatiales	-0.454	8.88E-03
181	CBR4	Sphingomonadaceae	-0.474	7.02E-03	547	HOOK1	Sphingomonadaceae	-0.505	7.83E-03	913	PPP4R3B	Sphingomonadaceae	-0.566	7.54E-03	1279	TMEM33	Sphingomonadaceae	-0.483	2.04E-03
182	CCDC124	Micromonosporaceae	-0.490	2.42E-03	548	HOXA3	Sphingomonas hankookensis	-0.260	2.70E-03	914	PPP6R1	Sphingomonadaceae	0.438	5.98E-07	1280	TMEM40	Pseudomonas oleovorans	-0.185	9.40E-03
183	CCDC158	Lactobacillus	0.201	1.53E-03	549	HPCAL1	Micromonosporaceae	-0.487	1.99E-03	915	PPP6R2	Sphingomonadaceae	0.353	2.45E-03	1281	TMEM47	Sphingomonadaceae	-0.538	5.10E-03
184	CCDC171	Sphingomonas sp. FARSPH	-0.090	6.66E-03	550	HPGD	Sphingomonadaceae	-0.432	2.02E-17	916	PRCP	Nitrobacteraceae	0.079	4.70E-03	1282	TMEM59	Cellulomonadaceae	0.552	5.15E-04
185	CCDC22	Streptococcus thermophilus	-0.291	3.23E-04	551	HPN	Micromonosporaceae	-0.482	6.34E-03	917	PRDM2	Cellulomonadaceae	-0.397	3.63E-04	1283	TMEM64	Caulobacterales	-0.554	8.15E-03
186	CCDC50	Sphingomonadaceae	-0.495	6.82E-05	552	HPS4	Gordonia bronchialis	-0.371	6.65E-03	918	PRDM2	Lactobacillus	0.160	8.74E-06	1284	TMEM65	Sphingomonadaceae	-0.486	1.54E-04
187	CCDC82	Chromatiales	-0.401	5.09E-03	553	HS1BP3	Sphingomonadaceae	0.371	5.22E-03	919	PRDM6	Pseudomonas oleovorans	-0.315	2.07E-03	1285	TMEM70	Cellulomonadaceae	0.318	3.94E-05
188	CCDC9	Mycolicibacterium	-0.474	6.72E-03	554	HS2ST1	Caulobacterales	-0.526	1.64E-03	920	PRELID1	Cellulomonadaceae	0.353	7.83E-03	1286	TMEM80	Caulobacterales	0.416	3.09E-05
189	CCDC9	Sphingomonadaceae	0.428	3.62E-03	555	HS6ST2	Pseudomonas oleovorans	-0.265	3.83E-03	921	PRELID3B	Sphingomonadaceae	-0.487	5.95E-05	1287	TMEM82	Micromonosporaceae	-0.461	6.18E-03
190	CCDC90B	Sphingomonadaceae	-0.453	8.44E-03	556	HSD17B14	Intrasporangiaceae	-0.326	3.24E-03	922	PRKCI	Micromonosporaceae	0.503	3.18E-03	1288	TMEM9B	Gordonia bronchialis	0.316	1.95E-04
191	CCDC91	Cyanobacteriota	-0.439	9.82E-03	557	HSPA14	Sphingomonadaceae	-0.350	3.50E-03	923	PRKCSH	Sphingomonadaceae	0.471	2.00E-03	1289	TMEM9B	Sphingosinicellaceae	0.380	4.07E-03
192	CCDC97	Sphingomonadaceae	0.451	3.35E-03	558	HSPE1	Cellulomonadaceae	0.341	1.61E-04	924	PRKD2	Sphaerotilaceae	0.386	1.25E-10	1290	TMX3	Sphingomonadaceae	-0.238	2.32E-03
193	CCL5	Corynebacterium tuberculoste	-0.405	1.40E-04	559	HSPG2	Cellulomonadaceae	-0.336	7.48E-04	925	PRKG2	Pseudomonas oleovorans	-0.208	5.75E-03	1291	TNFRSF10A	Sphingomonadaceae	0.223	4.76E-03
194	CCM2	Bacillaceae	-0.463	1.49E-03	560	HUNK	Propionibacteriaceae	0.356	1.94E-03	926	PRND	Pseudomonas oleovorans	-0.309	3.67E-04	1292	TNFRSF14	Lactobacillus iners	-0.498	9.43E-03
195	CCN1	Debaryomycetaceae	0.053	5.69E-03	561	HUWE1	Cellulomonadaceae	-0.264	2.57E-03	927	PRPF3	Cellulomonadaceae	-0.492	2.99E-04	1293	TNFRSF18	Pseudomonas oleovorans	-0.363	3.17E-03
196	CCN6	Sphingomonadaceae	-0.359	2.16E-03	562	HYI	Oscillatoriales	0.254	4.67E-03	928	PRPF39	Sphingomonadaceae	-0.295	1.20E-03	1294	TNPO1	Micromonosporaceae	0.581	2.02E-03
197	CCNB1	Archaea	0.216	2.23E-07	563	HYI	Ottowia	0.106	5.32E-14	929	PRR12	Sphingomonadaceae	0.483	7.47E-03	1295	TNRC6A	Cellulomonadaceae	-0.347	5.93E-03
198	CCNC	Sphingomonadaceae	-0.445	3.74E-08	564	ICA1L	Pseudomonas oleovorans	-0.342	7.67E-03	930	PRR14	Lactobacillus iners	-0.496	1.68E-03	1296	TOGARAM1	Micromonosporaceae	0.534	3.91E-03
199	CCND2	Staphylococcus capitis	-0.252	5.02E-03	565	ICOSLG	Sphingomonadaceae	0.334	9.88E-04	931	PRR7	Corynebacterium durum	0.051	3.31E-03	1297	TOGARAM2	Sphingomonas hankookensis	-0.353	3.48E-03
200	CCNJL	Sphingomonas sp. FARSPH	-0.130	1.22E-03	566	IDH3G	Nevskiales	-0.286	6.62E-03	932	PRRC2A	Cellulomonadaceae	-0.452	3.08E-03	1298	TOM1	Sphingomonadaceae	0.451	4.48E-03
201	CCNT2	Brevibacteriaceae	0.308	1.60E-06	567	IER3IP1	Sphingomonadaceae	-0.498	9.86E-03	933	PRRC2B	Cellulomonadaceae	-0.463	4.30E-04	1299	TOM1	Stutzerimonas	-0.487	4.70E-03
202	CCNT2	Sphingomonadaceae	-0.281	3.13E-13	568	IFT140	Mycolicibacterium	-0.467	6.98E-03	934	PRRC2C	Cellulomonadaceae	-0.279	1.43E-03	1300	TOMM22	Cellulomonadaceae	0.397	3.68E-03
203	CCPG1	Sphingomonadaceae	-0.551	8.03E-08	569	IFT74	Cyanobacteriota	-0.441	7.60E-04	935	PRRG1	Sphingomonadaceae	-0.498	3.00E-03	1301	TOMM5	Cellulomonadaceae	0.376	6.11E-03
204	CCSER1	Staphylococcus saprophyticus	0.369	2.06E-03	570	IGDCC4	Pseudomonas oleovorans	-0.301	7.46E-04	936	PRSS35	Archaea	0.207	8.43E-03	1302	TOR1A	Gordonia bronchialis	0.300	8.85E-03
205	CCT5	Sphaerotilaceae	-0.272	9.44E-05	571	IGFN1	Pseudomonas oleovorans	-0.370	5.55E-03	937	PRTG	Pseudomonas oleovorans	-0.237	4.11E-04	1303	TP53	Cutibacterium	0.215	5.98E-04
206	CD164	Sphingomonadaceae	-0.605	6.95E-04	572	IGSF21	Methylobacterium sp. FF17	-0.387	1.14E-03	938	PRX	Sphingomonadaceae	0.304	6.35E-03	1304	TP53	Nevskiales	-0.458	1.99E-06
207	CD2AP	Micromonosporaceae	0.466	4.21E-03	573	IKZF5	Sphingomonadaceae	-0.454	3.34E-03	939	PSMA7	Lautropia	-0.299	2.86E-09	1305	TPD52	Sphingomonadaceae	-0.450	4.77E-20
208	CD40	Caulobacterales	0.418	6.65E-03	574	IL1RAPL1	Brevundimonas sp.	0.189	8.89E-04	940	PSMD12	Sphingomonadaceae	-0.498	2.67E-03	1306	TPR	Cellulomonadaceae	-0.218	1.93E-03
209	CD47	Sphingomonadaceae	-0.507	9.08E-04	575	ILK	Alphaproteobacteria	0.326	2.18E-03	941	PTAR1	Sphingomonadaceae	-0.498	6.40E-03	1307	TRAF2	Stutzerimonas	-0.547	2.81E-03
210	CD58	Sphingomonadaceae	-0.485	4.81E-04	576	IMPA1	Bacillus velezensis	-0.335	6.11E-03	942	PTBP2	Brevibacteriaceae	0.345	2.34E-03	1308	TRAF7	Sphingomonadaceae	0.312	1.24E-05
211	CD59	Cellulomonadaceae	0.487	5.63E-03	577	IMPACT	Caulobacterales	-0.532	1.06E-06	943	PTBP2	Sphingomonadaceae	-0.238	4.11E-04	1309	TRAM1	Sphingomonadaceae	-0.614	6.35E-03
212	CDC26	Lautropia	-0.205	5.69E-03	578	INF2	Sphingomonadaceae	0.491	1.05E-03	944	PTBP3	Micromonosporaceae	0.514	7.50E-03	1310	TRAPPC12	Actinomycetaceae	-0.295	6.22E-03
213	CDC5L	Bacillaceae	0.488	3.84E-05	579	ING5	Sphingomonas hankookensis	-0.481	9.23E-04	945	PTDSS1	Bacillaceae	0.392	1.24E-03	1311	TRAPPC12	Sphingomonadaceae	0.297	2.17E-03

214	CDH7	Lautropia	-0.425	8.69E-04	580	INPP5B	Gordonia bronchialis	-0.427	7.93E-04	946	PTDSS1	Spirochaetales	0.433	2.67E-07	1312	TRAPPC13	Sphingomonadaceae	-0.518	7.44E-38
215	CDH7	Lawsonella	0.304	6.29E-03	581	INTS1	Sphingomonadaceae	0.445	7.67E-03	947	PTEN	Sphingomonadaceae	-0.592	1.24E-07	1313	TRAPPC6B	Sphingomonadaceae	-0.511	1.15E-06
216	CDH7	Pseudomonas oleovorans	-0.300	1.31E-06	582	IP6K1	Brevibacteriaceae	-0.274	9.04E-03	948	PTGER1	Pseudomonas oleovorans	-0.270	8.73E-03	1314	TRDMT1	Bacteria	0.215	9.39E-04
217	CDKL4	Sphingomonas sp. FARSPH	-0.337	1.86E-03	583	IP6K1	Nevskiales	-0.260	5.99E-08	949	PTGES3	Sphingomonadaceae	-0.527	8.78E-05	1315	TRIAP1	Cellulomonadaceae	0.331	7.35E-03
218	CDKN1B	Sphingomonadaceae	-0.432	3.09E-05	584	IPMK	Sphingomonadaceae	-0.477	2.61E-03	950	PTOV1	Lactobacillus iners	-0.513	4.84E-04	1316	TRIO	Cellulomonadaceae	-0.341	1.52E-03
219	CELF4	Sphingomonas sp. FARSPH	-0.489	1.27E-03	585	IPO4	Intrasporangiaceae	-0.227	8.88E-03	951	PTPRR	Pseudomonas oleovorans	-0.267	3.62E-03	1317	TRIOBP	Sphingomonadaceae	0.471	7.07E-04
220	CEMIP	Sphingomonas sp. FARSPH	-0.326	1.34E-04	586	IPPK	Sphingomonas hankookensis	0.346	6.30E-07	952	PTTG1IP2	Nostocaceae	0.303	5.76E-03	1318	TRIP10	Lactobacillus iners	-0.414	6.67E-03
221	CEMIP2	Bacillaceae	0.478	5.06E-03	587	IQCE	Pseudomonas sp. CIP-10	0.095	1.54E-03	953	PUS7L	Sphingomonadaceae	-0.524	7.60E-05	1319	TRMT10C	Sphingomonadaceae	-0.372	2.96E-03
222	CENPT	Lactobacillus iners	-0.470	1.26E-03	588	IQSEC3	Sphingomonas sp. FARSPH	-0.391	7.50E-04	954	PXDC1	Micromonosporales	-0.405	1.15E-04	1320	TRMT13	Sphingomonadaceae	-0.271	7.84E-03
223	CEP128	Staphylococcus saprophyticus	0.363	1.35E-03	589	IRAK1	Sphingomonadaceae	0.392	2.45E-08	955	PXDNL	Staphylococcus saprophyticus	0.201	2.02E-04	1321	TRMT1L	Sphingomonadaceae	-0.550	4.95E-06
224	CEP290	Brevibacteriaceae	0.209	4.75E-05	590	IRAK2	Bacillus velezensis	0.300	3.07E-03	956	PXMP2	Cellulomonadaceae	0.378	4.26E-03	1322	TRNT1	Sphingomonadaceae	-0.461	9.73E-03
225	CEP70	Sphingomonadaceae	-0.410	5.88E-03	591	IRGQ	Gordonia bronchialis	-0.287	8.14E-03	957	PYROXD2	Malassezia vespertilionis	0.377	6.22E-03	1323	TRRAP	Cellulomonadaceae	-0.327	2.53E-04
226	CEPT1	Sphingomonadaceae	-0.382	2.74E-03	592	ISCA1	Sphingomonadaceae	-0.370	6.16E-03	958	PYY	Sphingomonas hankookensis	-0.205	3.92E-03	1324	TSC1	Cellulomonadaceae	-0.369	5.05E-03
227	CES1	Lactococcus lactis	-0.212	8.86E-04	593	ISCU	Cellulomonadaceae	0.236	1.39E-03	959	R3HDM2	Cellulomonadaceae	-0.398	9.65E-03	1325	TSC22D4	Sphaerotilaceae	0.395	3.85E-03
228	CFAP52	Pseudomonas oleovorans	-0.246	1.16E-03	594	ISG20	Corynebacterium durum	0.198	1.82E-03	960	R3HDM2	Gardnerella	0.108	1.39E-03	1326	TSNAX	Caulobacterales	-0.468	2.89E-03
229	CFAP92	Sphingomonas sp. FARSPH	-0.418	1.00E-02	595	ITGA11	Pseudomonas oleovorans	-0.383	8.57E-03	961	RAB10	Sphingomonadaceae	-0.624	7.62E-03	1327	TSNAX	Sphingomonadaceae	-0.495	1.41E-03
230	CFL2	Caulobacterales	-0.460	9.76E-03	596	ITGA2B	Sphingomonas sp. FARSPH	-0.454	3.75E-03	962	RAB11FIP2	Micromonosporaceae	0.499	7.97E-08	1328	TSPAN14	Bacillus velezensis	0.380	1.45E-03
231	CGGBP1	Actinomycetaceae	0.235	8.54E-03	597	ITM2B	Sphingomonadaceae	-0.494	2.93E-04	963	RAB11FIP3	Sphingomonadaceae	0.505	2.04E-04	1329	TSPAN7	Lactobacillus	-0.252	3.07E-03
232	CGGBP1	Sphingomonadaceae	-0.519	1.60E-06	598	ITPRID1	Pseudomonas oleovorans	-0.356	4.41E-03	964	RAB14	Caulobacterales	-0.472	2.21E-06	1330	TSR1	Sphingomonas hankookensis	0.403	6.34E-03
233	CHD7	Cellulomonadaceae	-0.354	4.95E-24	599	JKAMP	Sphingomonadaceae	-0.455	2.59E-13	965	RAB18	Caulobacterales	-0.580	4.43E-07	1331	TTC14	Sphingomonadaceae	-0.291	8.45E-03
234	CHD8	Cellulomonadaceae	-0.255	2.54E-03	600	JRK	Pichiaceae	-0.371	2.52E-03	966	RAB18	Sphingomonadaceae	-0.622	2.59E-08	1332	TTC17	Lautropia	0.353	4.48E-03
235	CHERP	Sphingomonadaceae	0.367	2.49E-07	601	JUND	Lactobacillus iners	-0.530	1.55E-03	967	RAB1B	Nevskiales	-0.383	2.00E-04	1333	TTC23L	Sphingomonas sp. FARSPH	-0.377	3.75E-03
236	CHMP2A	Cellulomonadaceae	0.220	9.74E-03	602	KANTR	Cellulomonadaceae	-0.223	5.05E-03	968	RAB21	Caulobacterales	-0.431	6.56E-04	1334	TWF1	Sphingomonadaceae	-0.559	1.94E-03
237	CHMP2B	Caulobacterales	-0.527	7.43E-03	603	KATNBL1	Sphingomonadaceae	-0.541	6.68E-08	969	RAB2A	Caulobacterales	-0.406	1.97E-06	1335	TXNDC16	Micromonosporaceae	0.547	2.08E-03
238	CHMP2B	Cyanobacteriota	-0.419	4.15E-04	604	KBTBD3	Sphingomonadaceae	-0.474	6.98E-03	970	RAB2A	Sphingomonadaceae	-0.482	1.17E-07	1336	TXNL4A	Cellulomonadaceae	0.333	8.18E-03
239	CHMP2B	Sphingomonadaceae	-0.564	1.82E-06	605	KBTBD7	Sphingomonadaceae	-0.506	8.89E-03	971	RAB30	Bacillaceae	0.535	2.48E-03	1337	TYW5	Sphingomonadaceae	-0.412	5.56E-03
240	CHMP5	Gordonia bronchialis	0.310	7.30E-03	606	KCNC3	Cellulomonadaceae	-0.354	1.49E-07	972	RAB30	Caulobacteraceae	-0.484	1.56E-03	1338	U2AF1L4	Lactobacillus iners	-0.488	1.44E-03
241	CHPF	Bacillales	-0.510	7.83E-03	607	KCNJ14	Mycolicibacterium	-0.376	7.72E-03	973	RAB37	Rhizobiaceae	-0.108	5.16E-03	1339	UBA3	Sphingomonadaceae	-0.482	2.14E-08
242	CHRNB4	Brevundimonas sp.	0.225	6.77E-04	608	KCNS1	Lautropia	-0.306	1.58E-04	974	RAB3B	Pseudomonas oleovorans	-0.288	5.96E-04	1340	UBAC1	Kocuria palustris	0.015	9.46E-03
243	CHST12	Dermacoccaceae	-0.344	5.85E-03	609	KCNT1	Sphingomonas sp. FARSPH	-0.332	1.56E-03	975	RAB40B	Sphingomonas hankookensis	-0.441	2.10E-03	1341	UBAP2L	Cellulomonadaceae	-0.287	7.90E-03
244	CHST6	Sphingomonas sp. FARSPH	-0.280	1.58E-03	610	KCNU1	Archaea	0.346	4.22E-03	976	RAB40C	Sphaerotilaceae	0.368	1.42E-03	1342	UBB	Cellulomonadaceae	0.371	7.15E-03
245	CHURC1	Cyanobacteriota	-0.435	8.45E-04	611	KCTD16	Lautropia	-0.364	1.74E-03	977	RAB9B	Lactobacillus	0.296	3.91E-07	1343	UBD	Gemmatales	0.180	5.30E-04
246	CILP2	Pseudomonas oleovorans	-0.296	7.21E-03	612	KDM3B	Cellulomonadaceae	-0.354	5.08E-03	978	RAD17	Actinomycetaceae	0.201	6.01E-03	1344	UBE2B	Sphingomonadaceae	-0.431	5.19E-03
247	CKS1B	Cellulomonadaceae	0.368	6.41E-03	613	KIAA1217	Gordonia bronchialis	-0.221	4.57E-04	979	RAD17	Sphingomonadaceae	-0.465	9.49E-08	1345	UBE2D1	Sphingomonadaceae	-0.494	1.07E-05
248	CKS1B	Lautropia	-0.198	5.60E-05	614	KIAA1217	Pichiaceae	-0.408	8.65E-03	980	RAD21	Micromonosporaceae	0.515	1.55E-03	1346	UBE2D2	Cellulomonadaceae	0.338	2.21E-06
249	CLASP1	Cellulomonadaceae	-0.299	2.29E-04	615	KIAA1586	Sphingomonadaceae	-0.532	1.22E-04	981	RADIL	Pseudomonas oleovorans	-0.358	9.04E-03	1347	UBE2D3	Sphingomonadaceae	-0.598	4.55E-09
250	CLASP2	Micromonosporaceae	0.471	8.89E-03	616	KIF15	Sphingomonas sp. FARSPH	-0.151	8.85E-03	982	RAI1	Sphingomonadaceae	0.435	4.46E-03	1348	UBE2G1	Sphingomonadaceae	-0.555	2.86E-04
251	CLASRP	Lactobacillus iners	-0.520	9.08E-03	617	KLHL11	Sphingomonadaceae	-0.490	6.64E-04	983	RALBP1	Rhizobiaceae	-0.317	2.87E-04	1349	UBE2K	Sphingomonadales	-0.598	1.39E-06
252	CLCN3	Micromonosporaceae	0.531	8.85E-03	618	KLHL21	Sphingomonadaceae	0.464	2.39E-03	984	RANBP3	Sphaerotilaceae	0.355	1.04E-03	1350	UBE2QL1	Bacillaceae	-0.319	2.05E-03
253	CLCN7	Sphingomonadaceae	0.478	3.46E-03	619	KLHL8	Stutzerimonas	0.506	4.92E-03	985	RAP1B	Sphingomonadaceae	-0.565	8.17E-03	1351	UBE2V2	Sphingomonadaceae	-0.532	5.96E-04
254	CLEC4D	Gordonia bronchialis	0.170	8.12E-03	620	KLK11	Clostridia	-0.291	1.23E-03	986	RAP2B	Gardnerella	-0.376	7.64E-03	1352	UBE2W	Sphingomonadaceae	-0.609	1.25E-06
255	CLEC4G	Lactobacillus	-0.327	1.40E-03	621	KMT2A	Sphingosinicellaceae	-0.251	9.41E-04	987	RAP2C	Sphingomonadaceae	-0.509	1.88E-04	1353	UBLCP1	Sphingomonadaceae	-0.554	8.27E-04
256	CLHC1	Rhizobiaceae	0.261	1.21E-03	622	KMT2C	Cellulomonadaceae	-0.264	2.31E-05	988	RASGEF1A	Sphingomonas sp. FARSPH	-0.305	2.74E-03	1354	UBR4	Cellulomonadaceae	-0.344	6.62E-03
257	CLIC5	Staphylococcus capitis	-0.226	2.66E-04	623	KMT2D	Cellulomonadaceae	-0.429	2.88E-03	989	RBBP4	Bacillaceae	0.493	7.85E-03	1355	UCHL5	Caulobacterales	-0.534	8.08E-03
258	CLINT1	Micromonosporaceae	0.460	7.73E-04	624	KPNA5	Sphingomonadaceae	-0.482	6.11E-05	990	RBBP5	Micromonosporaceae	0.510	5.16E-03	1356	UCHL5	Sphingomonadaceae	-0.630	3.35E-05
259	CLP1	Devosiaceae	0.402	1.15E-03	625	KRAS	Caulobacterales	-0.496	5.89E-03	991	RBBP8	Sphaerotilaceae	-0.431	3.77E-03	1357	UEVLD	Sphingomonadaceae	-0.472	6.75E-03
260	CLTA	Cellulomonadaceae	0.254	8.16E-03	626	KRAS	Micromonosporaceae	0.472	6.01E-05	992	RBCK1	Micromonosporaceae	-0.475	1.80E-03	1358	UFM1	Sphingomonadaceae	-0.574	6.62E-05
261	CMC1	Cellulomonadaceae	0.393	5.40E-04	627	KRAS	Sphingomonadales	-0.544	3.22E-09	993	RBFOX3	Staphylococcus saprophyticus	0.052	2.26E-03	1359	UGT1A4	Lactobacillus iners	0.489	1.61E-07
262	CMPK1	Caulobacterales	-0.510	1.49E-03	628	KRR1	Caulobacterales	-0.514	2.58E-12	994	RBM26	Sphingomonadaceae	-0.366	8.55E-07	1360	UMAD1	Sphingomonadaceae	-0.554	2.48E-04
263	CMPK1	Sphingomonadaceae	-0.535	9.65E-06	629	KRR1	Sphingomonadaceae	-0.566	7.52E-16	995	RBM42	Stutzerimonas	-0.560	1.03E-03	1361	UNC5C	Pseudomonas oleovorans	-0.251	3.20E-03
264	CMTM6	Sphingomonadales	-0.542	6.20E-04	630	KYAT3	Sphingomonadaceae	-0.463	2.69E-04	996	RBPJ	Sphingomonadaceae	-0.582	2.62E-04	1362	UNC5D	Pseudomonas oleovorans	-0.344	3.01E-04
265	CNBP	Cellulomonadaceae	0.390	2.84E-03	631	L1TD1	Sphingomonas sp. FARSPH	-0.251	9.16E-03	997	RC3H1	Sphingomonadaceae	-0.416	3.40E-04	1363	UNK	Devosiaceae	-0.506	2.01E-07
266	CNN1	Pseudomonas sp. CIP-10	0.185	5.43E-03	632	LAMB4	Sphingomonas sp. FARSPH	-0.195	3.35E-03	998	RCCD1	Klebsiella michiganensis	-0.547	5.71E-04	1364	UNKL	Sphaerotilaceae	0.231	2.07E-27
267	CNOT2	Sphingomonadaceae	-0.402	3.88E-04	633	LAMTOR5	Cellulomonadaceae	0.395	3.80E-03	999	RCHY1	Sphingomonadaceae	-0.571	1.79E-03	1365	UQCRFS1	Cellulomonadaceae	0.442	6.32E-04

268	CNR1	Sphingomonas sp. FARSPH	-0.180	8.95E-03	634	LANCL1	Micromonosporaceae	0.568	4.92E-03	1000	RCN3	Lactobacillus iners	-0.476	1.92E-03	1366	UQCRH	Cellulomonadaceae	0.351	5.15E-07
269	CNTD1	Veillonella parvula	-0.322	3.52E-06	635	LAT	Lactobacillus iners	-0.531	8.55E-03	1001	REC8	Schlegelella aquatica	0.168	1.77E-03	1367	USP1	Sphaerotilaceae	-0.463	6.89E-05
270	CNTLN	Sphingomonadaceae	-0.485	1.78E-03	636	LBHD1	Nitrobacteraceae	-0.035	3.24E-05	1002	REEP6	Intrasporangiaceae	-0.357	7.35E-03	1368	USP1	Sphingomonadaceae	-0.594	7.13E-06
271	CNTN4	Pseudomonas oleovorans	-0.239	4.95E-03	637	LCORL	Sphingomonadaceae	-0.551	1.89E-05	1003	REPS2	Pseudomonas oleovorans	0.409	7.07E-04	1369	USP15	Sphingomonadaceae	-0.447	6.17E-03
272	CNTN4	Sphingomonas sp. FARSPH	-0.260	3.61E-03	638	LGALS2	Hypocreales	0.197	2.34E-09	1004	REXO5	Pseudomonas oleovorans	-0.239	8.10E-03	1370	UVSSA	Cellulomonadaceae	-0.376	1.64E-08
273	CNTNAP5	Pseudomonas oleovorans	-0.450	3.07E-04	639	LGALS2	Sphingosinicellaceae	0.230	3.86E-10	1005	RGMA	Pseudomonas oleovorans	-0.339	7.32E-03	1371	UVSSA	Sphingosinicellaceae	-0.373	1.78E-06
274	COA6	Sphingomonadaceae	-0.362	6.92E-03	640	LIF	Pseudomonas oleovorans	-0.348	3.26E-08	1006	RGS22	Pseudomonas oleovorans	-0.204	8.68E-03	1372	VAC14	Sphingomonadaceae	0.368	9.60E-14
275	COL11A1	Pseudomonas oleovorans	-0.338	8.77E-05	641	LIG3	Clostridia	0.265	4.94E-03	1007	RHBDD3	Micromonosporaceae	-0.506	9.97E-03	1373	VAMP4	Sphingomonadaceae	-0.505	3.03E-10
276	COL13A1	Achromobacter deleyi	-0.487	1.09E-03	642	LIME1	Micromonosporaceae	-0.522	4.69E-03	1008	RHBDF2	Sphingomonadaceae	0.441	6.58E-03	1374	VARS1	Sphingomonadaceae	0.417	2.22E-06
277	COL22A1	Pseudomonas oleovorans	-0.332	4.28E-04	643	LIN7C	Sphingomonadaceae	-0.448	1.85E-04	1009	RHBDL3	Sphingomonas sp. FARSPH	-0.253	9.32E-03	1375	VENTX	Pseudomonas oleovorans	-0.455	3.85E-03
278	COL6A3	Kocuria palustris	-0.275	1.17E-08	644	LIN9	Sphaerotilaceae	-0.374	9.25E-05	1010	RHOC	Nevskiales	-0.270	2.24E-03	1376	VHL	Dermacoccaceae	-0.405	5.39E-05
279	COL9A2	Spirosomaceae	-0.373	4.54E-03	645	LIPH	Lactobacillus	0.338	1.98E-03	1011	RIMS2	Brevundimonas sp.	0.181	1.64E-03	1377	VMA21	Sphingomonadaceae	-0.401	1.13E-03
280	COMMD10	Sphingomonadaceae	-0.545	9.46E-03	646	LMBRD1	Sphingomonadaceae	-0.552	4.58E-05	1012	RIPOR2	Bacillus velezensis	0.374	9.52E-03	1378	VPS37B	Bacillaceae	-0.453	9.13E-03
281	COMMD6	Cellulomonadaceae	0.375	9.74E-03	647	LMBRD2	Micromonosporaceae	0.521	2.84E-03	1013	RMDN2	Sphingomonadaceae	-0.501	2.06E-03	1379	VPS4B	Micromonosporaceae	0.552	1.14E-04
282	COMTD1	Micromonosporaceae	-0.539	8.94E-04	648	LMF1	Micromonosporaceae	-0.418	1.21E-03	1014	RNASE4	Sphingomonadaceae	-0.458	2.08E-04	1380	VPS54	Sphingomonadaceae	-0.458	1.35E-03
283	COPS2	Caulobacterales	-0.534	1.45E-06	649	LMOD1	Pseudomonas sp. CIP-10	0.187	4.75E-03	1015	RNF11	Sphingomonadaceae	-0.574	3.70E-03	1381	VSX1	Lawsonella clevelandensis	0.269	5.58E-03
284	COPS2	Sphingomonadaceae	-0.575	3.10E-06	650	LNPK	Caulobacterales	-0.560	1.97E-03	1016	RNF13	Sphingomonadaceae	-0.558	6.50E-04	1382	VTA1	Sphingomonadaceae	-0.529	7.12E-06
285	CORIN	Sphingomonas sp. FARSPH	-0.267	1.31E-03	651	LPAR6	Sphingomonadaceae	-0.391	3.45E-03	1017	RNF138	Caulobacterales	-0.471	1.38E-07	1383	VWA1	Bacillales	-0.520	4.24E-03
286	COX11	Sphingomonadaceae	-0.509	1.16E-04	652	LRFN3	Sphingomonadaceae	0.409	4.02E-03	1018	RNF138	Sphingomonadaceae	-0.555	2.73E-08	1384	VWA3B	Staphylococcus saprophyticus	0.172	7.98E-03
287	COX20	Sphingomonadaceae	-0.364	7.97E-03	653	LRP1	Cellulomonadaceae	-0.346	2.27E-05	1019	RNF139	Caulobacterales	-0.515	2.22E-06	1385	WAPL	Micromonosporaceae	0.530	8.22E-03
288	COX6C	Cellulomonadaceae	0.339	1.56E-03	654	LRP2BP	Sphingomonas hankookensis	-0.374	4.01E-05	1020	RNF139	Sphingomonadaceae	-0.539	2.42E-08	1386	WASL	Caulobacterales	-0.542	3.20E-04
289	COX7A2L	Cellulomonadaceae	0.392	3.72E-04	655	LRP5	Sphingomonadaceae	0.307	7.58E-03	1021	RNF146	Sphingomonadaceae	-0.276	6.87E-03	1387	WDFY1	Micromonosporaceae	0.554	8.72E-03
290	CRAMP1	Cellulomonadaceae	-0.439	8.27E-03	656	LRRC40	Micromonosporaceae	0.517	1.13E-03	1022	RNF150	Gemmatales	0.192	7.27E-03	1388	WDR18	Cutibacterium	0.185	6.09E-04
291	CREB1	Sphingomonadaceae	-0.556	9.23E-03	657	LRRC40	Sphingomonadaceae	-0.601	9.69E-07	1023	RNF166	Prevotella melaninogenica	-0.487	4.10E-03	1389	WDR45B	Nevskiales	-0.274	8.95E-03
292	CREB3	Cellulomonadaceae	0.392	1.96E-04	658	LYG2	Streptococcus sanguinis	0.244	2.96E-03	1024	RNF175	Pseudomonas oleovorans	-0.330	7.36E-07	1390	WDR46	Stutzerimonas	-0.451	4.14E-03
293	CREBBP	Cellulomonadaceae	-0.423	4.93E-05	659	LYPLAL1	Sphingomonadaceae	-0.473	1.99E-06	1025	RNF208	Sphaerotilaceae	0.356	4.45E-03	1391	WRAP53	Micromonosporaceae	-0.461	5.31E-03
294	CREBZF	Sphingomonadaceae	-0.327	5.80E-09	660	LYSMD2	Cellulomonadaceae	0.322	5.47E-03	1026	RNF213	Cellulomonadaceae	-0.342	8.53E-04	1392	XPO1	Sphingomonadaceae	-0.450	8.76E-03
295	CRIP2	Micromonosporaceae	-0.544	2.63E-03	661	LYSMD3	Sphingomonadaceae	-0.584	1.23E-04	1027	RNF214	Devosiaceae	-0.438	1.14E-03	1393	YAF2	Sphingomonadaceae	-0.323	1.32E-03
296	CRIPT	Sphingomonadaceae	-0.416	8.47E-03	662	LYSMD4	Nectriaceae	-0.315	2.96E-03	1028	RNF40	Sphingomonadaceae	0.425	8.98E-03	1394	YIPF4	Cyanobacteriota	-0.390	8.14E-03
297	CRYZ	Pseudomonas sp. CIP-10	-0.123	1.99E-05	663	LZTFL1	Sphingomonadaceae	-0.464	3.52E-03	1029	RNF7	Cellulomonadaceae	0.299	1.76E-09	1395	YOD1	Sphingomonadaceae	-0.578	1.48E-03
298	CSK	Sphaerotilaceae	0.314	6.81E-06	664	M6PR	Rhizobiaceae	-0.321	5.00E-03	1030	RNFT1	Sphingomonadaceae	-0.489	7.32E-07	1396	YPEL5	Caulobacterales	-0.440	4.14E-07
299	CSK	Sphingomonadaceae	0.469	1.08E-07	665	MACF1	Cellulomonadaceae	-0.305	2.74E-06	1031	RNPEPL1	Sphingomonadaceae	0.589	3.50E-04	1397	YPEL5	Sphingomonadaceae	-0.479	9.70E-20
300	CSRP2	Myxococcota	0.305	7.35E-04	666	MAIP1	Sphingosinicellaceae	0.292	5.06E-03	1032	RO60	Sphingomonadaceae	-0.542	5.84E-04	1398	YWHAZ	Sphaerotilaceae	-0.319	4.35E-03
301	CTBP1	Lactobacillus iners	-0.472	6.29E-05	667	MAL2	Sphingomonadaceae	-0.589	5.84E-06	1033	ROBO4	Nitrobacteraceae	-0.226	2.81E-04	1399	ZBBX	Pseudomonas oleovorans	-0.243	1.90E-03
302	CTBS	Caulobacterales	-0.493	2.25E-04	668	MALSU1	Cellulomonadaceae	0.284	4.12E-03	1034	RP1	Pseudomonas oleovorans	-0.169	1.85E-03	1400	ZBED5	Sphingomonadaceae	-0.318	1.49E-09
303	CTDSP2	Nitrobacteraceae	-0.201	2.61E-03	669	MAN2B2	Alcaligenaceae	-0.135	2.32E-05	1035	RPA3	Lautropia	-0.257	5.19E-03	1401	ZBTB26	Sphingomonadaceae	-0.526	2.19E-03
304	CTF1	Schlegelella aquatica	0.293	7.46E-03	670	MAP2K2	Micromonosporaceae	-0.485	2.96E-06	1036	RPL10A	Cellulomonadaceae	0.343	7.67E-03	1402	ZBTB6	Sphingomonadaceae	-0.592	2.91E-05
305	CTNNA2	Pseudomonas oleovorans	-0.213	1.20E-05	671	MAP3K14	Bacillaceae	-0.534	1.46E-03	1037	RPL22	Cellulomonadaceae	0.393	1.47E-03	1403	ZC3H12C	Streptococcaceae	-0.304	4.44E-05
306	CTTN	Mycolicibacterium	-0.502	2.89E-04	672	MAP4	Sphingomonadaceae	0.389	5.09E-03	1038	RPS12	Cellulomonadaceae	0.376	5.65E-03	1404	ZC3H15	Sphaerotilaceae	-0.391	6.08E-04
307	CUEDC2	Lautropia	-0.332	6.30E-03	673	MAP6D1	Nostocaceae	-0.439	8.72E-03	1039	RPS14	Cellulomonadaceae	0.273	6.06E-03	1405	ZC3H15	Sphingomonadaceae	-0.515	5.31E-07
308	CUL4B	Sphingomonadaceae	-0.535	2.53E-03	674	MAPK1IP1L	Chromatiales	-0.389	1.92E-03	1040	RPS27A	Cellulomonadaceae	0.295	2.56E-03	1406	ZC3H3	Sphaerotilaceae	0.336	7.93E-07
309	CUL5	Sphingomonadaceae	-0.596	2.02E-03	675	MAPKAPK5	Bacillus velezensis	-0.387	6.19E-03	1041	RPS3	Cellulomonadaceae	0.359	5.90E-04	1407	ZC3H7B	Nitrobacteraceae	-0.281	3.36E-13
310	CXCL6	Intrasporangiaceae	0.168	2.27E-03	676	MARCHF7	Micromonosporaceae	0.514	8.68E-03	1042	RPS5	Cellulomonadaceae	0.413	4.42E-03	1408	ZCCHC8	Bacillus velezensis	-0.412	2.08E-04
311	CXXC5	Bacillaceae	-0.480	9.61E-03	677	MARS2	Sphingomonas hankookensis	0.490	1.02E-03	1043	RPS6KA4	Sphingomonadaceae	0.426	2.67E-03	1409	ZDHHC12	Micromonosporaceae	-0.516	4.60E-04
312	CYP24A1	Pseudomonas oleovorans	-0.317	1.24E-05	678	MAST2	Cellulomonadaceae	-0.469	3.56E-04	1044	RPSA	Cellulomonadaceae	0.348	3.02E-03	1410	ZDHHC17	Sphingomonadaceae	-0.453	2.45E-04
313	CYP3A7	Intrasporangiaceae	0.211	8.12E-03	679	MATR3	Micromonosporaceae	0.472	5.73E-03	1045	RPSA	Lautropia	-0.214	8.58E-03	1411	ZDHHC21	Sphingomonadaceae	-0.416	4.02E-03
314	CYP46A1	Sphingomonas hankookensis	-0.445	3.70E-03	680	MAVS	Cellulomonadaceae	-0.386	4.20E-03	1046	RPTOR	Sphingomonadaceae	0.334	1.20E-09	1412	ZER1	Sphaerotilaceae	0.305	8.35E-03
315	CYP4V2	Bacillaceae	0.490	2.03E-27	681	MBD3	Micromonosporaceae	-0.542	3.51E-04	1047	RPUSD1	Micromonosporaceae	-0.528	3.34E-03	1413	ZFAND6	Sphingomonadaceae	-0.555	5.44E-04
316	CYTH2	Stutzerimonas	-0.485	8.85E-03	682	MCM3AP	Mycobacteroides	-0.371	2.02E-03	1048	RRAGD	Xanthomonadales	-0.410	1.18E-09	1414	ZFP41	Sphingomonadaceae	0.332	8.20E-03
317	DAB2IP	Sphingomonadaceae	0.415	7.66E-04	683	MCRIP2	Micromonosporaceae	-0.506	7.97E-03	1049	RREB1	Cellulomonadaceae	-0.422	6.43E-03	1415	ZFP91	Stutzerimonas	0.570	1.77E-05
318	DCDC2C	Sphingomonas sp. FARSPH	-0.206	4.78E-03	684	MDFIC	Sphingomonadaceae	-0.420	5.32E-03	1050	RRP15	Bacteroidaceae	0.287	2.76E-03	1416	ZFR	Micromonosporaceae	0.550	1.07E-03
319	DCP2	Sphingomonadales	-0.528	1.67E-10	685	MDH1	Cellulomonadaceae	0.511	4.81E-03	1051	RSBN1	Sphingomonadaceae	-0.501	6.95E-03	1417	ZFR2	Sphingomonas hankookensis	-0.251	6.79E-08
320	DCPS	Spirosomaceae	-0.369	8.80E-03	686	MDH1	Methyloversatilis sp. RAC08	0.333	2.78E-03	1052	RSBN1L	Sphingomonadaceae	-0.488	2.14E-09	1418	ZFYVE28	Sphaerotilaceae	0.414	1.86E-03
321	DCST1	Gordonia bronchialis	-0.288	5.06E-03	687	MDH2	Lautropia	-0.200	5.28E-13	1053	RSL24D1	Cellulomonadaceae	0.402	3.92E-04	1419	ZMIZ1	Cellulomonadaceae	-0.440	9.68E-04

322	DCTN4	Caulobacteraceae	-0.565	2.90E-03	688	MEA1	Nevskiales	-0.230	9.33E-08	1054	RTF2	Cellulomonadaceae	0.426	2.28E-04	1420	ZMYM5	Sphingomonadaceae	-0.508	2.80E-03
323	DCTN4	Micromonosporaceae	0.526	2.15E-03	689	MED25	Sphaerotilaceae	0.344	6.24E-04	1055	RTL5	Yersiniaceae	-0.249	7.26E-03	1421	ZMYND8	Streptococcus oralis	-0.012	4.81E-04
324	DCUN1D1	Caulobacterales	-0.546	1.15E-03	690	MED25	Sphingomonadaceae	0.561	2.08E-04	1056	RTL8C	Stutzerimonas	-0.528	3.83E-03	1422	ZNF133	Cellulomonadaceae	-0.466	1.04E-03
325	DCUN1D1	Sphingomonadaceae	-0.602	1.34E-06	691	MED4	Sphingomonadaceae	-0.526	7.52E-05	1057	RTN4R	Acinetobacter baumannii	0.141	4.09E-03	1423	ZNF138	Sphingomonadaceae	-0.468	9.98E-03
326	DCUN1D4	Sphingomonadaceae	-0.471	2.01E-04	692	MED6	Nostocaceae	0.252	1.48E-03	1058	RWDD3	Chromatiaceae	-0.355	1.74E-04	1424	ZNF181	Sphingomonadaceae	-0.414	1.83E-03
327	DDT	Micromonosporaceae	-0.478	7.96E-03	693	MEF2A	Lautropia	0.277	3.97E-03	1059	RWDD3	Sphingomonadaceae	-0.339	8.85E-11	1425	ZNF219	Sphaerotilaceae	0.324	7.25E-03
328	DEK	Cyanobacteriota	-0.506	2.12E-03	694	MEGF11	Sphingomonas sp. FARSPH	-0.313	1.13E-03	1060	RXFP1	Sphingomonadaceae	-0.441	8.66E-03	1426	ZNF232	Corynebacterium durum	0.197	6.86E-03
329	DENND1B	Sphingomonadaceae	-0.547	5.96E-04	695	MEIOB	Dermacoccaceae	-0.385	5.03E-03	1061	RYK	Sphingomonadaceae	-0.495	2.12E-03	1427	ZNF280D	Sphingomonadaceae	-0.463	5.03E-05
330	DENND4C	Micromonosporaceae	0.545	9.80E-04	696	METAP2	Cellulomonadaceae	0.404	1.76E-03	1062	SAA4	Leuconostoc	0.245	6.24E-03	1428	ZNF282	Mycolicibacterium	-0.465	4.19E-03
331	DESI2	Sphingomonadaceae	-0.531	4.37E-03	697	METRN	Micromonosporaceae	-0.561	2.63E-04	1063	SAFB	Mycolicibacterium	-0.517	5.96E-04	1429	ZNF282	Sphingomonadaceae	0.552	1.41E-03
332	DEXI	Ottowia	0.330	1.00E-06	698	METTL3	Streptococcus oralis	0.099	2.13E-03	1064	SAFB2	Mycolicibacterium	-0.529	9.36E-03	1430	ZNF284	Leuconostoc	-0.339	7.48E-06
333	DGCR2	Sphingomonadaceae	0.471	4.11E-03	699	MFSD8	Sphingomonadaceae	-0.493	9.95E-04	1065	SC5D	Caulobacteraceae	-0.414	8.95E-03	1431	ZNF302	Sphingomonadaceae	-0.330	2.36E-03
334	DGCR6	Micromonosporaceae	-0.472	6.93E-04	700	MGAT4B	Sphingomonadaceae	0.502	2.09E-09	1066	SCAMP1	Sphingomonadaceae	-0.521	9.08E-03	1432	ZNF319	Sphaerotilaceae	0.307	1.55E-03
335	DHX57	Pichiaceae	-0.233	4.79E-03	701	MICOS10	Lautropia	-0.319	7.84E-03	1067	SCAND1	Micromonosporaceae	-0.516	5.58E-03	1433	ZNF41	Lautropia	0.323	3.09E-03
336	DIAPH3	Brevundimonas sp.	0.308	3.82E-03	702	MICU2	Sphingomonadaceae	-0.469	5.31E-12	1068	SCCPDH	Methyloversatilis sp. RAC08	0.207	3.89E-03	1434	ZNF441	Hypocreales	-0.126	6.03E-05
337	DIDO1	Bacteroidaceae	-0.372	1.14E-04	703	MICU3	Brevibacteriaceae	0.301	1.01E-05	1069	SCOC	Caulobacterales	-0.517	5.98E-04	1435	ZNF449	Brevibacteriaceae	0.333	9.98E-03
338	DIDO1	Cellulomonadaceae	-0.420	1.44E-03	704	MICU3	Sphingomonadaceae	-0.309	1.50E-12	1070	SCOC	Sphingomonadaceae	-0.581	7.86E-06	1436	ZNF502	Bacillus velezensis	-0.260	3.85E-04
339	DIPK2A	Sphingomonadaceae	-0.438	2.67E-03	705	MIER1	Caulobacterales	-0.512	1.64E-03	1071	SCYL2	Caulobacteraceae	-0.552	3.91E-03	1437	ZNF514	Cellulomonadaceae	-0.508	7.15E-03
340	DIS3	Micromonosporaceae	0.543	1.93E-03	706	MIER1	Sphingomonadaceae	-0.570	5.33E-06	1072	SDF2	Cellulomonadaceae	0.409	3.44E-04	1438	ZNF534	Sphingomonas sp. FARSPH	-0.321	6.34E-03
341	DLGAP2	Sphingomonas sp. FARSPH	-0.442	1.29E-03	707	MIF	Corynebacterium kefirresidentii	0.022	1.04E-03	1073	SDHB	Methyloversatilis sp. RAC08	0.318	3.57E-03	1439	ZNF536	Pseudomonas oleovorans	-0.263	9.36E-03
342	DLL4	Lactobacillus	-0.294	5.90E-11	708	MIIP	Micromonosporaceae	-0.536	1.18E-04	1074	SDHD	Cellulomonadaceae	0.469	8.92E-03	1440	ZNF551	Bacillaceae	0.441	5.42E-03
343	DLST	Brevibacteriaceae	-0.261	7.87E-05	709	MINPP1	Sphingomonadaceae	-0.554	5.11E-06	1075	SEC14L5	Achromobacter deleyi	-0.203	6.74E-03	1441	ZNF566	Sphingomonadaceae	-0.498	3.90E-04
344	DMRTA1	Sphingomonadaceae	-0.356	1.71E-03	710	MIOS	Sphingomonadaceae	-0.433	7.30E-03	1076	SEC16B	Alcaligenaceae	-0.426	1.01E-03	1442	ZNF575	Sphingomonas hankookensis	-0.471	1.36E-03
345	DMWD	Sphingomonadaceae	0.523	9.20E-04	711	MLH3	Gordonia bronchialis	-0.128	1.21E-09	1077	SELENOF	Cellulomonadaceae	0.449	1.89E-03	1443	ZNF621	Cellulomonadaceae	-0.486	1.17E-03
346	DNAH10	Sphingomonas sp. FARSPH	-0.281	3.91E-03	712	MMGT1	Sphingomonadales	-0.565	9.80E-04	1078	SELL	Bacillus velezensis	0.411	7.73E-04	1444	ZNF639	Sphingomonadaceae	-0.541	2.46E-27
347	DNAH12	Sphingomonas sp. FARSPH	-0.357	1.39E-04	713	MMRN2	Nevskiales	-0.282	9.79E-03	1079	SEPSECS	Sphingomonadaceae	-0.490	5.42E-04	1445	ZNF644	Sphingomonadaceae	-0.561	8.87E-04
348	DNAJB14	Sphingomonadaceae	-0.544	4.36E-05	714	MOBP	Sphingomonas sp. FARSPH	-0.283	3.44E-03	1080	SEPTIN10	Bacillales	0.467	2.62E-04	1446	ZNF654	Sphingomonadaceae	-0.569	1.55E-06
349	DNAJC21	Sphaerotilaceae	-0.358	8.24E-11	715	MPC2	Cellulomonadaceae	0.390	3.35E-03	1081	SEPTIN9	Sphingomonadaceae	0.419	1.79E-04	1447	ZNF704	Archaea	-0.358	5.89E-03
350	DNAJC24	Sphingomonadaceae	-0.463	8.66E-05	716	MPLKIP	Bacillus velezensis	-0.371	2.13E-03	1082	SERINC1	Micromonosporaceae	0.506	9.95E-03	1448	ZNF92	Sphingomonadaceae	-0.522	6.53E-03
351	DNAJC25	Sphingomonadaceae	-0.334	9.45E-03	717	MPLKIP	Pseudomonas sp. CIP-10	-0.218	6.05E-03	1083	SERINC2	Sphingomonadaceae	0.460	2.15E-03	1449	ZNHIT6	Sphingomonadaceae	-0.444	3.53E-04
352	DNAL4	Micromonosporaceae	-0.418	2.21E-03	718	MRFAP1	Cellulomonadaceae	0.306	2.46E-03	1084	SERP1	Sphingomonadaceae	-0.424	1.56E-27	1450	ZNRF2	Sphingomonadaceae	-0.515	7.27E-06
353	DNASE1L3	Lactobacillus	-0.329	1.96E-03	719	MRFAP1	Lautropia	-0.224	3.65E-03	1085	SERPINA5	Bacillaceae	0.384	8.66E-03	1451	ZRANB2	Sphingomonadaceae	-0.476	4.73E-05
354	DOCK5	Cellulomonadaceae	-0.427	2.01E-03	720	MROH1	Mycolicibacterium	-0.441	5.05E-03	1086	SETD2	Cellulomonadaceae	-0.362	7.59E-04	1452	ZSCAN29	Cellulomonadaceae	-0.270	8.52E-05
355	DOCK5	Vibrionaceae	-0.381	5.97E-03	721	MRPL15	Cellulomonadaceae	0.436	9.70E-03	1087	SETD5	Cellulomonadaceae	-0.458	9.96E-03	1453	ZSWIM1	Caulobacterales	0.497	1.83E-05
356	DPCD	Lautropia	-0.394	4.20E-03	722	MRPL4	Micromonosporaceae	-0.492	4.57E-03	1088	SETDB1	Cellulomonadaceae	-0.512	2.33E-03	1454	ZSWIM6	Cellulomonadaceae	-0.303	9.95E-03
357	DPEP1	Pseudomonas oleovorans	-0.236	1.25E-03	723	MRPL41	Micromonosporaceae	-0.458	3.71E-03	1089	SF3A2	Sphingomonadaceae	0.519	3.28E-04	1455	ZSWIM9	Mycolicibacterium	-0.468	9.56E-05
358	DPY19L3	Sphingomonadaceae	-0.416	5.03E-03	724	MRPL42	Sphingomonadaceae	-0.527	4.54E-04	1090	SFI1	Mycolicibacterium	-0.446	3.33E-03					
359	DPY30	Cellulomonadaceae	0.372	4.92E-03	725	MRPS18C	Anoxybacillus	0.372	1.72E-05	1091	SFN	Nitrobacteraceae	-0.011	2.91E-21					
360	DR1	Sphingomonadaceae	-0.554	3.60E-03	726	MRPS18C	Sphingosinicellaceae	0.312	2.36E-31	1092	SFT2D3	Lawsonella	-0.362	7.12E-71					
361	DRAM1	Gordonia bronchialis	0.289	3.57E-04	727	MRPS23	Sphingosinicellaceae	0.335	8.84E-04	1093	SFT2D3	Lawsonella clevelandensis	-0.348	7.73E-13					
362	DRP2	Pseudomonas oleovorans	-0.295	1.00E-03	728	MRPS34	Micromonosporaceae	-0.466	3.27E-03	1094	SGCZ	Sphingomonas sp. FARSPH	-0.151	5.49E-03					
363	DST	Cellulomonadaceae	-0.251	7.40E-04	729	MRTFA	Sphingomonadaceae	0.490	5.09E-03	1095	SGPP1	Sphingomonadaceae	-0.539	1.68E-06					
364	DTWD1	Sphingomonadaceae	-0.401	7.69E-04	730	MSH4	Sphingomonas sp. FARSPH	-0.198	1.37E-03	1096	SH3BGRL	Sphingomonadaceae	-0.517	8.63E-03					
365	DYNC1H1	Cellulomonadaceae	-0.205	1.77E-03	731	MTDH	Micromonosporaceae	0.516	8.46E-03	1097	SH3D19	Micromonosporaceae	0.505	5.32E-03					
366	DYNC2I1	Devosiaceae	-0.438	2.54E-05	732	MTERF1	Sphingomonadaceae	-0.463	7.22E-04	1098	SH3GL1	Sphingomonadaceae	0.430	4.70E-03					

Supplementary Table 14: Individual level host gene-microbiota interaction results by LASSO in NASH

ID	Gene	Taxa	Coeff	FDR	ID	Gene	Taxa	Coeff	FDR	ID	Gene	Taxa	Coeff	FDR	ID	Gene	Taxa	Coeff	FDR
1	AADAC	Ralstonia insidiosa	-0.308	3.55E-04	734	EEA1	Comamonadaceae	-0.305	6.75E-03	1467	MZT1	Malasseziaceae	0.275	1.00E-03	2200	SLC35A1	Malassezia vespertilionis	0.284	5.17E-03
2	AAK1	Acidovorax sp. KKS102	-0.153	1.65E-03	735	EEF1E1	Ralstonia insidiosa	-0.271	4.61E-04	1468	MZT2A	Ralstonia insidiosa	0.279	3.90E-03	2201	SLC35A1	Ralstonia insidiosa	-0.297	3.21E-07
3	AASDHPPT	Ralstonia insidiosa	-0.294	1.08E-12	736	EEF2	Alphaproteobacteria	0.371	3.19E-05	1469	N6AMT1	Alphaproteobacteria	-0.268	2.62E-04	2202	SLC35A1	Sclerotiniaceae	0.354	3.25E-03
4	ABCA1	Ralstonia insidiosa	-0.195	3.31E-03	737	EEFSEC	Ralstonia insidiosa	0.174	3.44E-03	1470	NAA11	Brucella	0.005	5.06E-03	2203	SLC35A3	Ralstonia insidiosa	-0.307	2.84E-09
5	ABCB8	Ralstonia insidiosa	0.239	1.28E-03	738	EFCAB14	Ralstonia insidiosa	-0.274	1.38E-04	1471	NAA11	Dolosigranulum pigrum	0.320	3.87E-03	2204	SLC35A4	Enterobacteriaceae	0.249	3.31E-03
6	ABCC1	Sphingobacteriaceae	-0.313	2.98E-03	739	EFCAB2	Acidovorax sp. KKS102	-0.220	3.75E-03	1472	NAA30	Ralstonia insidiosa	-0.309	7.71E-09	2205	SLC35A5	Ralstonia insidiosa	-0.287	6.62E-08
7	ABCC4	Ralstonia insidiosa	-0.147	2.79E-03	740	EFCAB7	Ralstonia insidiosa	-0.180	9.47E-04	1473	NAA40	Prevotella melaninogenica	-0.243	6.21E-06	2206	SLC35B3	Ralstonia insidiosa	-0.280	1.97E-07
8	ABCC5	Actinomyces naeslundii	0.017	2.86E-03	741	EFEMP2	Ralstonia insidiosa	0.263	6.09E-03	1474	NAA50	Microbacteriaceae	-0.359	2.19E-03	2207	SLC35C1	Debaryomycetaceae	-0.227	1.54E-03
9	ABCD1	Ralstonia insidiosa	0.215	6.06E-04	742	EFR3A	Ralstonia insidiosa	-0.258	1.26E-07	1475	NAA60	Ralstonia insidiosa	0.280	9.57E-06	2208	SLC35D1	Porphyromonadaceae	0.425	7.61E-03
10	ABCD3	Ralstonia insidiosa	-0.245	5.44E-04	743	EGLN1	Caulobacterales	-0.388	1.36E-03	1476	NAB1	Ralstonia insidiosa	-0.306	4.40E-07	2209	SLC35F5	Ralstonia insidiosa	-0.254	1.80E-10
11	ABCE1	Ralstonia insidiosa	-0.334	1.19E-07	744	EGLN1	Epilithonimonas	0.380	5.99E-03	1477	NADK2	Caulobacterales	-0.381	3.34E-03	2210	SLC36A4	Ralstonia insidiosa	-0.226	6.20E-03
12	ABCE1	Sphaerotilaceae	-0.353	2.28E-03	745	EHBP1L1	Ralstonia insidiosa	0.188	1.22E-03	1478	NAF1	Ralstonia insidiosa	-0.209	5.11E-04	2211	SLC38A2	Caulobacterales	-0.345	1.98E-04
13	ABCF1	Caulobacteraceae	0.277	4.12E-03	746	EIF1AX	Ralstonia insidiosa	-0.264	9.04E-04	1479	NAGS	Bacillales	-0.429	1.35E-03	2212	SLC38A2	Ralstonia insidiosa	-0.207	4.04E-08
14	ABCF1	Neisseriaceae	-0.363	6.67E-03	747	EIF2AK3	Ustilaginaceae	0.218	5.08E-03	1480	NAMPT	Bacillales	0.449	9.24E-03	2213	SLC38A2	Sphaerotilaceae	-0.279	7.94E-03
15	ABCF3	Malasseziales	-0.299	5.27E-04	748	EIF3E	Ralstonia insidiosa	-0.287	1.87E-05	1481	NANP	Ralstonia insidiosa	-0.320	9.53E-03	2214	SLC39A1	Sordariales	-0.306	7.50E-03
16	ABHD16A	Acidobacteriota	-0.191	1.27E-07	749	EIF3M	Leptotrichiaceae	0.179	8.14E-03	1482	NAP1L1	Betaproteobacteria	-0.274	2.48E-03	2215	SLC39A5	Bacillales	-0.321	5.78E-04
17	ABHD17A	Ralstonia insidiosa	0.258	3.57E-13	750	EIF4A2	Ralstonia insidiosa	-0.229	2.12E-04	1483	NAP1L2	Bacillales	0.385	1.31E-03	2216	SLC39A6	Ralstonia insidiosa	-0.294	2.33E-05
18	ABHD17B	Ralstonia insidiosa	-0.254	5.28E-03	751	EIF4E	Ralstonia insidiosa	-0.312	1.57E-05	1484	NAPRT	Bacillales	-0.368	9.47E-03	2217	SLC39A8	Ralstonia insidiosa	-0.334	9.55E-10
19	ABHD2	Viruses	0.297	4.61E-03	752	EIF4G1	Caulobacteraceae	0.283	1.24E-04	1485	NASP	Alphaproteobacteria	-0.411	5.34E-03	2218	SLC39A9	Ralstonia insidiosa	-0.311	4.77E-05
20	ABHD5	Ralstonia insidiosa	-0.176	3.58E-07	753	EIF4H	Acidovorax sp. KKS102	0.298	1.69E-07	1486	NASP	Saccharomycetales	-0.431	6.38E-03	2219	SLC40A1	Ralstonia insidiosa	-0.288	1.45E-03
21	ABHD8	Ralstonia insidiosa	0.293	1.16E-06	754	ELAVL2	Nevskiales	0.011	8.45E-05	1487	NAT1	Debaryomycetaceae	0.194	2.90E-03	2220	SLC41A2	Malasseziales	0.277	4.73E-07
22	ABI1	Ralstonia insidiosa	-0.306	3.00E-05	755	ELFN1	Ralstonia insidiosa	0.234	2.48E-03	1488	NAT1	Ralstonia insidiosa	-0.183	2.01E-03	2221	SLC41A2	Ralstonia insidiosa	-0.224	1.59E-07
23	ABI3	Ralstonia insidiosa	0.247	4.52E-04	756	ELK4	Ralstonia insidiosa	-0.254	1.68E-08	1489	NBEAL1	Ralstonia insidiosa	-0.169	3.60E-08	2222	SLC43A2	Ralstonia insidiosa	0.198	2.07E-03
24	ABITRAM	Ralstonia insidiosa	-0.296	1.03E-05	757	ELL	Nocardioides	0.208	2.06E-03	1490	NBN	Bacillales	0.535	3.18E-04	2223	SLC47A2	Corynebacterium accolens	0.164	2.57E-05
25	ABLIM2	Ralstonia insidiosa	0.252	8.44E-03	758	ELL2	Ralstonia insidiosa	-0.234	6.87E-06	1491	NBN	Ralstonia insidiosa	-0.267	2.89E-07	2224	SLC4A4	Ralstonia insidiosa	-0.243	2.69E-04
26	ACAD11	Nevskiales	-0.209	2.12E-03	759	ELMOD2	Ralstonia insidiosa	-0.324	7.40E-11	1492	NBPF3	Actinomycetaceae	-0.185	8.59E-03	2225	SLC7A14	Gordonia bronchialis	0.270	1.81E-03
27	ACBD4	Bacillales	-0.481	2.15E-04	760	ELP4	Ralstonia insidiosa	-0.259	3.38E-04	1493	NCAN	Gordonia bronchialis	0.106	8.59E-03	2226	SLC7A4	Nitrobacteraceae	-0.204	3.90E-04
28	ACBD4	Ralstonia insidiosa	0.165	1.34E-04	761	EMC2	Ralstonia insidiosa	-0.233	1.33E-10	1494	NCEH1	Bacillales	0.473	7.11E-03	2227	SLC9A7	Acidovorax sp. T1	-0.102	1.65E-03
29	ACD	Bacillales	-0.370	2.23E-03	762	EMC4	Acidovorax sp. KKS102	0.091	6.76E-03	1495	NCKAP5L	Ralstonia insidiosa	0.222	1.50E-08	2228	SLC9A7	Debaryomycetaceae	0.215	1.51E-03
30	ACER3	Geodermatophilales	0.460	2.41E-04	763	EMILIN1	Ralstonia insidiosa	0.295	7.67E-08	1496	NCOR1	Delftia acidovorans	0.424	5.40E-03	2229	SLC9C1	Paucibacter	0.237	3.91E-03
31	ACIN1	Alphaproteobacteria	-0.410	2.54E-04	764	EML4	Ralstonia insidiosa	-0.252	2.25E-04	1497	NCOR1	Phyllobacteriaceae	0.414	7.53E-03	2230	SLC9C1	Thermales	-0.290	1.88E-03
32	ACO2	Alphaproteobacteria	0.479	7.20E-04	765	ENAH	Comamonadaceae	-0.296	7.39E-03	1498	NCR1	Corynebacterium accolens	0.106	1.77E-03	2231	SLCO5A1	Pseudomonas oleovorans	-0.123	4.59E-03
33	ACP1	Sphingobacteriaceae	0.157	2.42E-03	766	ENG	Ralstonia insidiosa	0.223	6.30E-05	1499	NDFIP2	Ralstonia insidiosa	-0.291	7.08E-08	2232	SLF1	Enterobacteriaceae	-0.220	9.34E-03
34	ACSL5	Ascomycota	0.364	1.82E-03	767	ENKD1	Bacillales	-0.529	2.26E-03	1500	NDUFA5	Ralstonia insidiosa	-0.254	5.73E-06	2233	SLFNL1	Prevotella melaninogenica	-0.206	2.71E-04
35	ACTL10	Cloacibacterium normanense	-0.174	1.88E-04	768	ENO1	Acidovorax sp. KKS102	0.187	3.29E-03	1501	NDUFAF4	Leptotrichiaceae	0.150	6.68E-04	2234	SLK	Porphyromonadaceae	0.453	7.08E-04
36	ACTN4	Ralstonia insidiosa	0.250	3.01E-05	769	ENO1	Caulobacteraceae	0.227	6.83E-04	1502	NDUFAF6	Alphaproteobacteria	-0.374	1.02E-04	2235	SLK	Ralstonia insidiosa	-0.175	7.77E-04
37	ACTR10	Sclerotiniaceae	0.310	2.47E-03	770	ENPEP	Ascomycota	0.335	1.62E-03	1503	NDUFS4	Actinomyces naeslundii	-0.214	1.00E-02	2236	SMAD1	Brevundimonas sp.	0.261	8.86E-03
38	ACTR1B	Acidobacteriota	-0.202	6.53E-03	771	ENPP1	Ralstonia insidiosa	-0.199	1.29E-03	1504	NDUFS7	Bacillales	-0.330	3.34E-03	2237	SMARCA4	Ralstonia insidiosa	0.207	4.15E-05
39	ACTR1B	Betaproteobacteria	0.313	8.57E-04	772	ENPP7	Puccinia triticina	-0.250	2.92E-03	1505	NDUFS7	Ralstonia insidiosa	0.265	6.44E-04	2238	SMARCAD1	Ralstonia insidiosa	-0.245	6.82E-09
40	ACTR6	Ralstonia insidiosa	-0.251	1.69E-10	773	ENTPD6	Saccharomycetales	-0.184	3.95E-05	1506	NEDD4	Ralstonia insidiosa	-0.197	2.77E-03	2239	SMARCB1	Ralstonia insidiosa	0.160	1.85E-04
41	ACVR1C	Ralstonia insidiosa	-0.228	3.27E-04	774	EPB41L4B	Ralstonia insidiosa	-0.264	6.68E-04	1507	NEIL1	Prevotella melaninogenica	-0.459	1.97E-03	2240	SMC2	Enterobacteriaceae	-0.250	2.01E-03
42	ADAM10	Ralstonia insidiosa	-0.188	5.21E-05	775	EPB41L5	Ralstonia insidiosa	-0.233	3.81E-03	1508	NEK7	Caulobacteraceae	-0.395	6.58E-03	2241	SMCHD1	Ralstonia insidiosa	-0.163	3.29E-04
43	ADAM11	Acidovorax sp. KKS102	-0.249	3.27E-03	776	EPC1	Ralstonia insidiosa	-0.114	3.92E-05	1509	NEK7	Comamonadaceae	-0.291	2.69E-03	2242	SMIM11	Cloacibacterium normanense	-0.224	2.47E-03
44	ADAM20	Acidovorax sp. KKS102	-0.183	1.59E-33	777	EPG5	Porphyromonadaceae	0.354	4.02E-04	1510	NEK7	Ralstonia insidiosa	-0.246	3.56E-09	2243	SMIM14	Ralstonia insidiosa	-0.234	1.10E-03
45	ADAMTS13	Bacillales	-0.437	5.10E-03	778	EPG5	Ralstonia insidiosa	-0.151	6.78E-05	1511	NELFA	Bacillales	-0.444	4.05E-03	2244	SMIM15	Ralstonia insidiosa	-0.306	3.78E-10
46	ADAMTS13	Ralstonia insidiosa	0.337	4.24E-07	779	EPHA5	Brucella	-0.333	1.14E-07	1512	NEMP1	Ralstonia insidiosa	-0.246	4.81E-05	2245	SMIM30	Ralstonia insidiosa	-0.242	2.62E-03
47	ADAMTS7	Ralstonia insidiosa	0.282	1.06E-03	780	EPHB3	Ralstonia insidiosa	0.231	6.96E-03	1513	NEU4	Bacillales	-0.359	5.52E-04	2246	SMNDC1	Betaproteobacteria	-0.305	3.22E-07
48	ADAMTSL2	Ralstonia insidiosa	0.241	8.76E-04	781	EPM2AIP1	Betaproteobacteria	-0.277	4.92E-09	1514	NEUROD6	Ornithinimicrobiaceae	0.307	2.40E-03	2247	SMPD4	Ralstonia insidiosa	0.211	5.74E-05
49	ADCK5	Bacillales	-0.407	9.89E-04	782	EPN1	Bacillales	-0.441	2.21E-03	1515	NFAT5	Betaproteobacteria	-0.380	8.06E-05	2248	SMTN	Ralstonia insidiosa	0.252	8.64E-05
50	ADCY10	Alphaproteobacteria	-0.278	3.59E-03	783	EPN1	Ralstonia insidiosa	0.274	4.98E-06	1516	NFIA	Delftia acidovorans	0.265	1.83E-06	2249	SNAPC2	Staphylococcus saprophyticus	-0.407	5.02E-03
51	ADD3	Bacillales	0.392	9.45E-03	784	EPS15	Microbacteriaceae	-0.393	5.95E-03	1517	NFIB	Delftia acidovorans	0.236	2.77E-12	2250	SNAPC4	Prevotella melaninogenica	-0.356	2.83E-03

52	ADGB	Pseudomonas oleovorans	0.001	7.71E-03	785	EPS8L2	Bacillales	-0.374	1.42E-03	1518	NFIC	Debaryomycetaceae	-0.219	3.15E-03	2251	SNAPC4	Ralstonia insidiosa	0.230	1.12E-06
53	ADGRA2	Ralstonia insidiosa	0.229	4.94E-03	786	ERBB4	Staphylococcus saprophyticus	0.254	2.06E-03	1519	NFIC	Ralstonia insidiosa	0.211	1.40E-03	2252	SNCAIP	Streptococcus oralis	0.278	6.21E-03
54	ADGRF1	Nevskiales	0.075	8.67E-04	787	ERBIN	Ralstonia insidiosa	-0.183	5.31E-07	1520	NFKBIB	Bacillales	-0.471	7.12E-04	2253	SNRK	Microbacteriaceae	-0.343	5.53E-03
55	ADGRG6	Ralstonia insidiosa	-0.170	2.05E-04	788	ERC2	Brucella	-0.034	4.23E-04	1521	NFKBIL1	Porphyromonadaceae	-0.298	5.50E-03	2254	SNRK	Ralstonia insidiosa	-0.255	1.05E-09
56	ADGRL1	Ralstonia insidiosa	0.207	6.47E-03	789	ERC2	Herbaspirillum huttiense	-0.024	5.95E-05	1522	NFYB	Ralstonia insidiosa	-0.286	3.27E-09	2255	SNX12	Acidovorax sp. KKS102	0.286	2.90E-03
57	ADGRL4	Geodermatophilales	0.424	5.68E-03	790	ERCC4	Delftia acidovorans	0.430	1.42E-03	1523	NHLRC3	Ralstonia insidiosa	-0.295	5.92E-08	2256	SNX13	Bacillales	0.485	2.14E-03
58	ADORA2B	Acidovorax sp. KKS102	0.105	4.20E-03	791	ERCC5	Porphyromonadaceae	0.283	4.10E-03	1524	NIT2	Leptotrichiaceae	0.064	9.58E-05	2257	SNX13	Porphyromonadaceae	0.520	4.22E-04
59	ADPRM	Ralstonia insidiosa	-0.195	2.70E-03	792	ERCC6	Delftia acidovorans	0.439	2.04E-12	1525	NKAP	Cloacibacterium normanense	-0.350	6.06E-04	2258	SNX13	Ralstonia insidiosa	-0.214	4.09E-08
60	ADSS2	Ralstonia insidiosa	-0.198	6.83E-04	793	ERF	Bacillales	-0.297	1.87E-05	1526	NKIRAS1	Ralstonia insidiosa	-0.265	5.88E-07	2259	SNX14	Microbacteriaceae	-0.352	8.41E-03
61	AFDN	Delftia acidovorans	0.221	1.55E-06	794	ERF	Ralstonia insidiosa	0.212	2.24E-03	1527	NKRF	Alphaproteobacteria	-0.347	9.66E-05	2260	SNX14	Ralstonia insidiosa	-0.353	1.67E-15
62	AFF1	Cloacibacterium normanense	0.334	5.88E-10	795	ERGIC2	Enterobacteriaceae	-0.298	7.56E-04	1528	NLRX1	Ralstonia insidiosa	0.213	6.82E-04	2261	SNX18	Moraxellaceae	-0.354	7.61E-03
63	AFMID	Saccharomycetales	-0.470	8.48E-03	796	ERGIC2	Ralstonia insidiosa	-0.256	1.26E-11	1529	NMD3	Caulobacterales	-0.363	3.73E-03	2262	SNX18	Saccharomycetales	0.400	2.85E-04
64	AFTPH	Ralstonia insidiosa	-0.242	5.97E-12	797	ERI2	Ralstonia insidiosa	-0.245	7.45E-04	1530	NMD3	Debaryomycetaceae	0.427	4.92E-04	2263	SNX8	Alphaproteobacteria	0.248	5.09E-04
65	AGAP3	Bacillales	-0.532	5.98E-04	798	ERLIN2	Porphyromonadaceae	0.393	4.39E-03	1531	NME9	Corynebacterium accolens	0.128	1.75E-05	2264	SNX8	Ralstonia insidiosa	0.215	3.44E-04
66	AGAP3	Ralstonia insidiosa	0.275	8.11E-06	799	ERO1A	Ralstonia insidiosa	-0.377	4.27E-06	1532	NMRK1	Alphaproteobacteria	-0.383	2.12E-04	2265	SOCS4	Ralstonia insidiosa	-0.259	9.17E-05
67	AGL	Ralstonia insidiosa	-0.216	2.91E-06	800	ERO1B	Ralstonia insidiosa	-0.236	2.46E-03	1533	NMUR1	Chitinophagales	-0.242	4.62E-03	2266	SOCS5	Porphyromonadaceae	0.447	9.05E-03
68	AGTR1	Ralstonia insidiosa	-0.193	6.81E-03	801	ESAM	Ralstonia insidiosa	0.236	6.79E-03	1534	NOL12	Ralstonia insidiosa	0.208	4.28E-04	2267	SOCS5	Ralstonia insidiosa	-0.248	3.73E-06
69	AHDC1	Epilithonimonas	-0.434	1.20E-03	802	ESCO1	Microbacteriaceae	-0.349	9.37E-03	1535	NOP53	Malassezia vespertilionis	-0.225	6.30E-03	2268	SON	Delftia acidovorans	0.353	2.36E-05
70	AHDC1	Ralstonia insidiosa	0.284	4.42E-08	803	ESCO1	Ralstonia insidiosa	-0.286	6.38E-07	1536	NOP58	Cloacibacterium normanense	-0.204	4.91E-04	2269	SORBS2	Ralstonia insidiosa	-0.191	4.96E-03
71	AIDA	Ralstonia insidiosa	-0.202	1.87E-03	804	ESF1	Cloacibacterium normanense	0.079	2.33E-13	1537	NOTCH1	Ralstonia insidiosa	0.236	4.19E-06	2270	SORBS3	Ralstonia insidiosa	0.120	1.34E-07
72	AIF1	Verrucomicrobiota	-0.139	8.07E-03	805	ESPN	Bacillales	-0.361	1.06E-03	1538	NOTCH2	Porphyromonadaceae	0.455	1.42E-03	2271	SOX12	Bacillales	-0.453	4.22E-03
73	AIG1	Methyloversatilis sp. RAC08	0.182	4.85E-03	806	ESPN	Ralstonia insidiosa	0.207	5.06E-04	1539	NOTUM	Bacillales	-0.414	7.34E-05	2272	SOX12	Ralstonia insidiosa	0.252	2.02E-05
74	AJM1	Bacillales	-0.347	6.33E-04	807	ESS2	Ralstonia insidiosa	0.230	6.70E-05	1540	NOXA1	Bacillales	-0.448	8.85E-03	2273	SOX13	Enterobacteriaceae	0.242	8.02E-03
75	AK3	Ralstonia insidiosa	-0.258	1.53E-04	808	ETAA1	Ralstonia insidiosa	-0.252	2.57E-05	1541	NPFF	Alphaproteobacteria	-0.394	4.54E-03	2274	SOX18	Brevibacteriaceae	0.055	9.98E-06
76	AK4	Ralstonia insidiosa	-0.312	2.04E-03	809	ETFRF1	Malassezia restricta	0.310	1.69E-03	1542	NPRL3	Ralstonia insidiosa	0.199	4.36E-05	2275	SP140L	Streptococcus oralis	0.053	2.37E-03
77	AKAP7	Ralstonia insidiosa	-0.216	4.73E-03	810	ETV3	Staphylococcus saprophyticus	0.340	3.62E-04	1543	NPY1R	Caulobacteraceae	-0.344	7.10E-03	2276	SP2	Debaryomycetaceae	-0.262	7.09E-03
78	AKAP9	Delftia acidovorans	0.443	2.31E-03	811	EVI5L	Ralstonia insidiosa	0.239	4.44E-06	1544	NR1D2	Ralstonia insidiosa	-0.188	1.08E-03	2277	SP3	Caulobacteraceae	-0.374	5.94E-03
79	AKAP9	Phyllobacteriaceae	0.430	6.15E-03	812	EVPL	Ascomycota	-0.187	8.48E-03	1545	NR1H2	Ralstonia insidiosa	0.252	3.75E-04	2278	SP3	Ralstonia insidiosa	-0.219	8.60E-09
80	AKIRIN2	Alphaproteobacteria	-0.422	9.03E-04	813	EWSR1	Alphaproteobacteria	-0.367	9.00E-03	1546	NR2C1	Ralstonia insidiosa	-0.189	9.99E-04	2279	SP5	Bacillales	-0.387	1.91E-03
81	ALCAM	Ralstonia insidiosa	-0.237	1.64E-05	814	EXOC1	Ralstonia insidiosa	-0.181	5.05E-05	1547	NR2F1	Sphingobacteriaceae	-0.227	6.48E-03	2280	SPACA6	Saccharomycetales	-0.279	1.07E-03
82	ALDH8A1	Acidovorax sp. KKS102	0.362	3.21E-03	815	EXOC3	Malasseziales	-0.309	1.09E-03	1548	NRAS	Ralstonia insidiosa	-0.311	1.47E-04	2281	SPAG16	Ralstonia insidiosa	-0.213	1.03E-06
83	ALG1	Acidovorax sp. KKS102	0.206	5.79E-05	816	EXOC3	Ralstonia insidiosa	0.193	6.89E-04	1549	NRIP1	Ralstonia insidiosa	-0.188	9.65E-07	2282	SPAG9	Cloacibacterium normanense	0.390	2.43E-03
84	ALG11	Porphyromonadaceae	0.516	1.86E-03	817	EXOC3L2	Ralstonia insidiosa	0.205	1.19E-05	1550	NRIP3	Pseudomonas oleovorans	-0.111	9.75E-03	2283	SPATA2L	Bacillales	-0.342	4.15E-06
85	ALG6	Ralstonia insidiosa	-0.318	5.05E-09	818	EXOC3L2	Sclerotiniaceae	-0.293	2.97E-03	1551	NRK	Brucella	-0.057	3.92E-03	2284	SPATA2L	Ralstonia insidiosa	0.263	3.58E-07
86	ALKBH6	Bacillaceae	-0.451	4.41E-03	819	EXOC5	Ralstonia insidiosa	-0.267	2.38E-08	1552	NRROS	Ralstonia insidiosa	0.222	2.71E-03	2285	SPDYE12	Gordonia bronchialis	0.140	6.99E-03
87	ALKBH8	Ralstonia insidiosa	-0.296	1.47E-03	820	EXOC8	Porphyromonadaceae	0.401	8.65E-04	1553	NRXN2	Cloacibacterium normanense	-0.176	7.50E-03	2286	SPEGNB	Methylocystaceae	-0.234	2.51E-03
88	ALS2	Ralstonia insidiosa	-0.194	1.21E-03	821	EXOC8	Ralstonia insidiosa	-0.279	1.65E-03	1554	NSRP1	Archaea	0.301	1.01E-03	2287	SPHK2	Bacillales	-0.368	3.47E-03
89	AMDHD2	Bacillales	-0.372	3.83E-04	822	EXOG	Acidovorax sp. KKS102	-0.143	7.43E-04	1555	NT5C2	Geodermatophilaceae	0.433	8.20E-03	2288	SPI1	Ralstonia insidiosa	0.179	1.76E-04
90	AMIGO3	Ralstonia insidiosa	0.174	6.99E-06	823	EXOSC3	Betaproteobacteria	-0.330	3.89E-03	1556	NT5C3A	Betaproteobacteria	-0.330	3.18E-09	2289	SPIN1	Ralstonia insidiosa	-0.290	2.83E-11
91	AMMECR1	Malasseziales	0.299	9.35E-07	824	EXOSC8	Alphaproteobacteria	-0.441	5.10E-03	1557	NT5C3A	Ralstonia insidiosa	-0.227	1.88E-05	2290	SPIN2A	Corynebacterium accolens	0.119	2.73E-06
92	AMMECR1	Ralstonia insidiosa	-0.222	2.55E-06	825	EXTL2	Betaproteobacteria	-0.286	6.68E-03	1558	NTAQ1	Herbaspirillum huttiense	-0.214	9.90E-05	2291	SPOCK1	Brucella	-0.190	5.67E-03
93	AMY2B	Alphaproteobacteria	-0.401	2.10E-03	826	EXTL2	Ralstonia insidiosa	-0.303	4.03E-06	1559	NTHL1	Bacillales	-0.453	4.04E-03	2292	SPOPL	Porphyromonadaceae	0.455	2.32E-04
94	AMZ2	Ralstonia insidiosa	-0.217	4.28E-03	827	F13B	Ralstonia insidiosa	-0.272	9.63E-09	1560	NUBP2	Bacillales	-0.394	2.43E-03	2293	SPOPL	Ralstonia insidiosa	-0.287	3.80E-06
95	ANAPC13	Ralstonia insidiosa	-0.259	2.55E-03	828	FAAP100	Bacillales	-0.351	7.89E-03	1561	NUBP2	Ralstonia insidiosa	0.237	3.77E-04	2294	SPPL2A	Ralstonia insidiosa	-0.193	1.35E-05
96	ANAPC4	Ralstonia insidiosa	-0.252	2.71E-03	829	FAAP20	Ralstonia insidiosa	0.295	1.90E-03	1562	NUDT12	Ralstonia insidiosa	-0.255	9.35E-07	2295	SREK1	Betaproteobacteria	-0.347	1.10E-04
97	ANGEL2	Ralstonia insidiosa	-0.242	2.05E-05	830	FADS6	Erwiniaceae	0.013	3.87E-03	1563	NUDT15	Sphingobacteriaceae	0.441	7.69E-05	2296	SREK1IP1	Enterobacteriaceae	-0.321	3.69E-03
98	ANGPTL6	Ralstonia insidiosa	0.231	4.35E-03	831	FAM133B	Cloacibacterium normanense	-0.061	3.81E-05	1564	NUDT21	Caulobacteraceae	-0.432	2.21E-06	2297	SRM	Ralstonia insidiosa	0.156	1.02E-05
99	ANK3	Brucella	-0.066	3.95E-04	832	FAM135A	Ralstonia insidiosa	-0.246	5.97E-12	1565	NUDT21	Ralstonia insidiosa	-0.310	1.00E-19	2298	SRP14	Malassezia vespertilionis	0.301	2.47E-03
100	ANKK1	Alphaproteobacteria	-0.274	7.07E-03	833	FAM161A	Phyllobacteriaceae	0.351	3.05E-05	1566	NUDT22	Ralstonia insidiosa	0.225	2.40E-04	2299	SRP9	Caulobacteraceae	-0.306	3.72E-03
101	ANKRD11	Debaryomycetaceae	-0.399	3.80E-03	834	FAM167B	Ralstonia insidiosa	0.233	3.85E-04	1567	NUFIP2	Porphyromonadaceae	0.424	9.57E-03	2300	SRP9	Microbacteriaceae	-0.336	8.99E-03
102	ANKRD11	Ralstonia insidiosa	0.339	2.43E-04	835	FAM193B	Prevotella melaninogenica	-0.396	6.06E-03	1568	NUP107	Ralstonia insidiosa	-0.127	5.79E-04	2301	SRPK1	Bacillales	0.374	5.15E-03
103	ANKRD12	Cloacibacterium normanense	0.106	4.21E-06	836	FAM199X	Ralstonia insidiosa	-0.275	1.55E-05	1569	NUP133	Ralstonia insidiosa	-0.204	1.94E-06	2302	SRPK2	Phyllobacteriaceae	0.452	8.36E-03
104	ANKRD12	Phyllobacteriaceae	0.463	3.12E-05	837	FAM200B	Caulobacteraceae	-0.254	1.35E-03	1570	NUP155	Cloacibacterium normanense	0.422	3.85E-03	2303	SRRM1	Phyllobacteriaceae	0.019	6.56E-05
105	ANKRD18B	Empedobacter falsenii	0.003	3.03E-03	838	FAM200B	Ralstonia insidiosa	-0.264	4.93E-06	1571	NUP54	Ralstonia insidiosa	-0.302	2.25E-15	2304	SRSF1	Ralstonia insidiosa	-0.246	2.95E-05

106	ANKRD20A1	Corynebacterium accolens	0.267	2.16E-04	839	FAM20C	Bacillales	-0.390	4.32E-03	1572	NUP58	Ralstonia insidiosa	-0.254	9.09E-04	2305	SRSF10	Enterobacteriaceae	-0.297	6.56E-05
107	ANKRD23	Acidovorax sp. KKS102	-0.320	6.14E-03	840	FAM217B	Enterobacteriaceae	-0.274	7.10E-03	1573	NUS1	Ralstonia insidiosa	-0.277	1.15E-07	2306	SRSF6	Alphaproteobacteria	-0.387	3.32E-03
108	ANKRD26	Cloacibacterium normanense	-0.054	7.06E-04	841	FAM228B	Alphaproteobacteria	-0.342	9.81E-03	1574	NUTM2B	Shinella	-0.125	6.44E-05	2307	SS18	Ralstonia insidiosa	-0.205	1.01E-04
109	ANKRD30A	Paucibacter	0.100	4.12E-03	842	FAM50B	Ascomycota	-0.280	2.51E-03	1575	NXPE2	Pseudomonas oleovorans	-0.024	1.95E-03	2308	SSBP4	Ascomycota	-0.378	9.50E-04
110	ANKRD36B	Phyllobacteriaceae	0.327	4.35E-04	843	FAM76B	Ralstonia insidiosa	-0.205	1.10E-11	1576	NXT2	Caulobacteraceae	-0.384	2.20E-04	2309	SSR1	Ralstonia insidiosa	-0.360	1.37E-03
111	ANKRD46	Ralstonia insidiosa	-0.311	5.92E-08	844	FAM8A1	Microbacteriaceae	-0.300	6.81E-03	1577	NXT2	Dermacoccaceae	0.419	2.21E-03	2310	SSX2IP	Ralstonia insidiosa	-0.261	2.18E-06
112	ANKRD49	Ralstonia insidiosa	-0.207	1.63E-05	845	FAM8A1	Ralstonia insidiosa	-0.334	4.81E-07	1578	NXT2	Ralstonia insidiosa	-0.277	1.71E-04	2311	ST3GAL2	Debaryomycetaceae	-0.386	2.77E-03
113	ANKRD50	Ralstonia insidiosa	-0.127	1.82E-06	846	FAM91A1	Ralstonia insidiosa	-0.208	2.67E-07	1579	OARD1	Debaryomycetaceae	0.144	4.23E-03	2312	ST3GAL2	Ralstonia insidiosa	0.196	2.43E-05
114	ANKRD61	Acidovorax sp. KKS102	-0.098	6.72E-07	847	FAM98B	Ralstonia insidiosa	-0.244	2.84E-10	1580	OAZ2	Corynebacterium accolens	-0.304	3.95E-03	2313	ST3GAL6	Ralstonia insidiosa	-0.226	1.77E-03
115	ANO5	Ralstonia insidiosa	-0.210	1.48E-03	848	FAN1	Cloacibacterium normanense	0.254	6.03E-04	1581	OBI1	Ralstonia insidiosa	-0.216	2.30E-04	2314	ST6GAL1	Ascomycota	0.411	4.17E-03
116	ANO6	Porphyromonadaceae	0.439	4.71E-05	849	FANCL	Ralstonia insidiosa	-0.210	8.85E-03	1582	OCIAD1	Debaryomycetaceae	0.320	1.07E-03	2315	STAB1	Sphingobacteriaceae	-0.392	6.06E-05
117	ANP32E	Caulobacteraceae	-0.324	9.59E-04	850	FASTKD1	Malassezia vespertilionis	0.237	5.16E-03	1583	OCIAD1	Ralstonia insidiosa	-0.327	4.05E-12	2316	STAG1	Bacillales	0.445	5.24E-03
118	ANXA11	Colletotrichum lupini	0.192	6.73E-03	851	FASTKD1	Ralstonia insidiosa	-0.173	5.31E-04	1584	OCLN	Cloacibacterium normanense	0.467	8.77E-03	2317	STAG1	Ralstonia insidiosa	-0.205	6.60E-05
119	AOC2	Acidovorax sp. KKS102	-0.340	8.06E-04	852	FASTKD3	Ralstonia insidiosa	-0.248	1.94E-04	1585	ODAD2	Rhodoferax	0.188	8.04E-03	2318	STAMBPL1	Blautia	0.171	5.14E-04
120	AP1AR	Ralstonia insidiosa	-0.224	1.95E-07	853	FAT4	Cloacibacterium normanense	0.386	2.22E-05	1586	ODR4	Bacillales	0.354	9.90E-04	2319	STARD3NL	Brevibacteriaceae	-0.077	1.34E-03
121	AP1G1	Bacillales	0.441	6.09E-03	854	FBRS	Debaryomycetaceae	-0.384	1.96E-03	1587	ODR4	Ralstonia insidiosa	-0.250	3.45E-06	2320	STARD4	Ralstonia insidiosa	-0.269	6.85E-03
122	AP2A1	Ralstonia insidiosa	0.217	8.47E-06	855	FBRSL1	Debaryomycetaceae	-0.472	3.43E-05	1588	OGDH	Caulobacteraceae	0.170	6.98E-04	2321	STARD8	Cloacibacterium normanense	0.165	6.64E-03
123	AP2A2	Enterobacteriaceae	0.284	9.64E-03	856	FBXL12	Ralstonia insidiosa	0.229	1.34E-04	1589	OGFOD2	Bacillales	-0.494	2.93E-03	2322	STARD9	Acidovorax sp. KKS102	-0.128	3.51E-03
124	AP2A2	Ralstonia insidiosa	0.227	3.80E-05	857	FBXL15	Ascomycota	-0.390	6.12E-03	1590	OGFR	Ralstonia insidiosa	0.247	2.24E-06	2323	STBD1	Alphaproteobacteria	0.293	8.52E-08
125	AP2M1	Brevibacteriaceae	-0.227	1.27E-05	858	FBXL15	Bacillales	-0.411	4.51E-05	1591	OGT	Ralstonia insidiosa	-0.177	6.30E-05	2324	STIM2	Ralstonia insidiosa	-0.160	4.09E-03
126	AP2M1	Malasseziales	-0.234	2.04E-03	859	FBXL18	Debaryomycetaceae	-0.356	6.68E-07	1592	OMA1	Ralstonia insidiosa	-0.284	3.42E-11	2325	STK11	Ralstonia insidiosa	0.159	1.88E-04
127	AP3D1	Ralstonia insidiosa	0.251	5.67E-07	860	FBXL18	Ralstonia insidiosa	0.156	1.57E-03	1593	OPA1	Ralstonia insidiosa	-0.277	1.65E-07	2326	STK24	Brevibacteriaceae	-0.107	4.15E-03
128	AP3S1	Malasseziaceae	0.292	4.02E-06	861	FBXL4	Ralstonia insidiosa	-0.204	7.75E-07	1594	OR13C3	Acinetobacter johnsonii	0.053	1.63E-03	2327	STK26	Ralstonia insidiosa	-0.257	9.26E-09
129	AP3S1	Ralstonia insidiosa	-0.328	1.19E-06	862	FBXL6	Bacillales	-0.430	1.59E-03	1595	OR5AN1	Corynebacterium accolens	0.285	3.61E-03	2328	STK38L	Ralstonia insidiosa	-0.227	2.85E-09
130	AP4B1	Alphaproteobacteria	-0.438	4.25E-03	863	FBXL8	Bacillales	-0.380	6.97E-03	1596	OR5AN1	Herbaspirillum huttiense	0.090	5.26E-04	2329	STOML2	Brevibacteriaceae	-0.243	6.14E-03
131	AP5B1	Ralstonia insidiosa	0.200	4.36E-03	864	FBXO11	Betaproteobacteria	-0.296	5.16E-13	1597	ORC2	Ralstonia insidiosa	-0.155	4.07E-03	2330	STOX1	Streptococcaceae	-0.226	5.54E-03
132	AP5M1	Ralstonia insidiosa	-0.291	2.72E-08	865	FBXO11	Ralstonia insidiosa	-0.258	9.34E-05	1598	ORC4	Microbacteriaceae	-0.322	1.82E-05	2331	STRN	Ralstonia insidiosa	-0.168	2.88E-08
133	APBA3	Ralstonia insidiosa	0.280	5.02E-05	866	FBXO28	Ralstonia insidiosa	-0.231	7.53E-08	1599	ORC4	Ralstonia insidiosa	-0.276	2.56E-20	2332	STRN4	Ralstonia insidiosa	0.165	2.19E-06
134	APC	Ralstonia insidiosa	-0.149	3.76E-03	867	FBXO3	Porphyromonadaceae	0.410	5.51E-03	1600	ORMDL1	Ralstonia insidiosa	-0.264	5.94E-04	2333	STT3B	Ralstonia insidiosa	-0.347	9.20E-14
135	APC2	Ralstonia insidiosa	0.226	4.01E-04	868	FBXO38	Cloacibacterium normanense	0.405	1.19E-03	1601	OS9	Caulobacteraceae	0.319	7.11E-03	2334	STX10	Porphyromonadaceae	-0.519	3.97E-04
136	APOB	Porphyromonadaceae	0.383	2.15E-05	869	FBXO44	Bacillales	-0.499	3.32E-03	1602	OSBPL8	Caulobacteraceae	-0.421	4.24E-03	2335	STXBP2	Ralstonia insidiosa	0.188	9.69E-09
137	APOL1	Debaryomycetaceae	-0.307	4.11E-03	870	FBXO46	Bacillales	-0.498	8.32E-05	1603	OSBPL8	Ralstonia insidiosa	-0.241	2.72E-06	2336	STXBP3	Ralstonia insidiosa	-0.219	1.95E-12
138	APOOL	Streptococcus salivarius	0.092	1.48E-03	871	FBXW4	Enterobacteriaceae	0.202	8.74E-03	1604	OSGIN2	Ralstonia insidiosa	-0.263	6.19E-03	2337	STXBP5	Ralstonia insidiosa	-0.144	3.53E-04
139	APPBP2	Ralstonia insidiosa	-0.255	1.26E-06	872	FBXW4	Ralstonia insidiosa	0.161	3.95E-03	1605	OSTC	Ralstonia insidiosa	-0.298	1.88E-04	2338	STXBP5L	Methyloversatilis sp. RAC08	0.083	8.71E-03
140	APPBP2	Saccharomycetales	0.419	1.39E-05	873	FBXW5	Betaproteobacteria	0.257	6.91E-03	1606	OSTM1	Enterobacteriaceae	-0.310	1.14E-04	2339	STYX	Caulobacteraceae	-0.365	9.96E-03
141	APTX	Leptotrichiaceae	0.064	1.66E-03	874	FCHO1	Ralstonia insidiosa	0.267	2.74E-04	1607	OTUD1	Dermacoccaceae	0.312	9.06E-03	2340	SUB1	Ralstonia insidiosa	-0.288	3.99E-05
142	APTX	Thermaceae	-0.242	5.37E-03	875	FCHO2	Ralstonia insidiosa	-0.213	4.30E-05	1608	OTUD4	Microbacteriaceae	-0.375	3.71E-03	2341	SUCNR1	Ralstonia insidiosa	-0.278	2.49E-03
143	ARAF	Enterobacteriaceae	0.275	3.26E-03	876	FEM1B	Bacillales	0.387	9.30E-03	1609	OTUD4	Porphyromonadaceae	0.434	5.73E-05	2342	SUCO	Ralstonia insidiosa	-0.242	4.35E-03
144	ARAP2	Enterobacteriaceae	-0.211	7.61E-03	877	FEM1C	Ralstonia insidiosa	-0.214	2.43E-06	1610	OTUD4	Ralstonia insidiosa	-0.240	1.06E-07	2343	SUDS3	Ralstonia insidiosa	-0.228	4.68E-06
145	ARF3	Alphaproteobacteria	0.317	4.06E-03	878	FGD1	Ralstonia insidiosa	0.224	6.28E-03	1611	OXSM	Leptotrichiaceae	0.076	3.17E-03	2344	SUGP1	Bacillales	-0.524	1.19E-04
146	ARHGAP1	Enterobacteriaceae	0.324	1.27E-03	879	FGF10	Pseudomonas oleovorans	-0.188	8.35E-03	1612	P3H3	Ralstonia insidiosa	0.213	9.78E-03	2345	SUMO1	Ralstonia insidiosa	-0.300	3.90E-03
147	ARHGAP12	Ralstonia insidiosa	-0.262	1.44E-08	880	FGF17	Acidovorax sp. KKS102	-0.344	4.13E-04	1613	PACS1	Ralstonia insidiosa	0.191	1.92E-03	2346	SUMO2	Ralstonia insidiosa	-0.356	4.73E-04
148	ARHGAP29	Ralstonia insidiosa	-0.180	1.01E-04	881	FGF21	Micromonospora	0.331	3.57E-13	1614	PAH	Ralstonia insidiosa	-0.240	1.47E-05	2347	SUMO3	Epilithonimonas	0.247	3.12E-07
149	ARHGAP4	Ralstonia insidiosa	0.230	2.01E-05	882	FGFR1OP2	Enterobacteriaceae	-0.265	8.48E-03	1615	PAICS	Ralstonia insidiosa	-0.293	6.37E-05	2348	SUPT20H	Actinomyces naeslundii	0.186	2.29E-03
150	ARHGAP45	Ralstonia insidiosa	0.228	1.83E-07	883	FGFR1OP2	Ralstonia insidiosa	-0.211	5.06E-06	1616	PAK4	Bacillales	-0.436	5.31E-04	2349	SUV39H2	Ralstonia insidiosa	-0.223	8.41E-03
151	ARHGDIA	Ralstonia insidiosa	0.177	1.95E-04	884	FGFRL1	Ralstonia insidiosa	0.177	1.22E-04	1617	PAK4	Ralstonia insidiosa	0.250	6.78E-08	2350	SVOP	Alphaproteobacteria	-0.362	1.87E-04
152	ARHGDIB	Verrucomicrobiota	-0.174	8.54E-03	885	FHIP1A	Porphyromonadaceae	0.301	5.32E-03	1618	PALM	Ralstonia insidiosa	0.211	1.11E-05	2351	SWT1	Ralstonia insidiosa	-0.157	6.50E-09
153	ARHGEF1	Ralstonia insidiosa	0.247	1.12E-07	886	FIGN	Betaproteobacteria	-0.255	1.03E-03	1619	PALS1	Porphyromonadaceae	0.457	2.68E-07	2352	SYCP3	Nitrobacteraceae	0.212	9.72E-03
154	ARHGEF12	Porphyromonadaceae	0.458	2.43E-04	887	FKTN	Bacillales	0.458	9.59E-03	1620	PAN3	Betaproteobacteria	-0.390	5.78E-04	2353	SYDE1	Ralstonia insidiosa	0.226	1.17E-03
155	ARHGEF12	Ralstonia insidiosa	-0.200	1.74E-06	888	FLII	Ralstonia insidiosa	0.181	7.06E-05	1621	PAPOLA	Caulobacteraceae	-0.371	5.91E-03	2354	SYMPK	Ralstonia insidiosa	0.229	1.27E-08
156	ARHGEF18	Ralstonia insidiosa	0.174	1.24E-05	889	FMO4	Ralstonia insidiosa	-0.204	4.84E-03	1622	PAPOLA	Ralstonia insidiosa	-0.289	1.80E-19	2355	SYNJ2BP	Ralstonia insidiosa	-0.191	6.35E-05
157	ARHGEF6	Bacillales	0.379	9.98E-03	890	FMO5	Ralstonia insidiosa	-0.170	1.29E-04	1623	PARN	Methyloversatilis sp. RAC08	0.332	2.02E-03	2356	SYPL1	Ralstonia insidiosa	-0.311	4.00E-08
158	ARID1A	Oscillospiraceae	-0.287	1.72E-04	891	FN3K	Bacillales	-0.457	8.48E-03	1624	PARP4	Cloacibacterium normanense	0.497	8.75E-03	2357	SYS1	Micromonospora	0.198	6.00E-03
159	ARID4A	Betaproteobacteria	-0.172	2.76E-05	892	FN3K	Caulobacteraceae	0.346	5.97E-03	1625	PAXBP1	Ralstonia insidiosa	-0.188	1.58E-06	2358	SYT15	Corynebacterium accolens	0.140	4.05E-04

160	ARID4A	Phyllobacteriaceae	0.460	3.74E-04	893	FNDC3A	Ralstonia insidiosa	-0.271	5.21E-07	1626	PCBP1	Caulobacteraceae	0.227	8.92E-03	2359	SYT16	Paucibacter	0.139	7.81E-03
161	ARID4B	Delftia acidovorans	0.319	3.75E-06	894	FNIP1	Corynebacterium accolens	0.366	4.80E-04	1627	PCDH17	Mycolicibacterium	0.340	9.96E-03	2360	TAB3	Ralstonia insidiosa	-0.291	1.20E-05
162	ARID4B	Phyllobacteriaceae	0.412	5.48E-05	895	FOXA2	Bacillales	-0.370	6.05E-03	1628	PCDHGA5	Selenomonadaceae	0.299	3.22E-03	2361	TADA1	Ralstonia insidiosa	-0.258	4.33E-07
163	ARL13B	Enterobacteriaceae	-0.248	8.12E-04	896	FOXJ3	Ralstonia insidiosa	-0.202	5.25E-04	1629	PCDHGB6	Porphyromonadaceae	0.188	2.36E-05	2362	TADA2A	Alphaproteobacteria	-0.463	7.38E-07
164	ARL15	Caulobacteraceae	-0.330	6.42E-03	897	FOXK1	Ralstonia insidiosa	0.216	1.93E-05	1630	PCGF2	Ralstonia insidiosa	0.194	2.83E-04	2363	TAF1	Shinella	-0.138	1.05E-04
165	ARL17B	Aeromonadales	0.211	7.13E-03	898	FOXN2	Caulobacteraceae	-0.342	4.27E-03	1631	PCGF5	Comamonadaceae	-0.302	8.74E-03	2364	TAF2	Brevundimonas sp.	0.385	8.90E-03
166	ARL17B	Archaea	0.232	3.87E-06	899	FOXN2	Ralstonia insidiosa	-0.206	1.32E-05	1632	PCGF6	Ralstonia insidiosa	-0.251	6.89E-03	2365	TAF2	Ralstonia insidiosa	-0.185	6.25E-04
167	ARL2	Ralstonia insidiosa	0.302	3.18E-04	900	FOXO3B	Corynebacterium accolens	0.231	9.89E-04	1633	PCIF1	Enterobacteriaceae	0.311	8.53E-05	2366	TAF9	Malassezia vespertilionis	0.256	1.83E-03
168	ARL5B	Caulobacterales	-0.400	1.90E-04	901	FOXP4	Ralstonia insidiosa	0.150	4.03E-11	1634	PCMTD2	Ralstonia insidiosa	-0.327	5.12E-06	2367	TAF9B	Caulobacteraceae	-0.395	2.62E-04
169	ARL5B	Ralstonia insidiosa	-0.273	4.85E-07	902	FPGT	Ralstonia insidiosa	-0.308	7.51E-09	1635	PCNP	Caulobacteraceae	-0.350	2.48E-03	2368	TAF9B	Ralstonia insidiosa	-0.319	1.34E-09
170	ARL6IP1	Ralstonia insidiosa	-0.274	4.46E-05	903	FRMD3	Bacillales	0.318	8.56E-03	1636	PCNP	Malasseziales	0.325	7.93E-11	2369	TANK	Ralstonia insidiosa	-0.219	2.17E-05
171	ARL6IP5	Ralstonia insidiosa	-0.309	9.70E-08	904	FRMD5	Carnobacteriaceae	0.295	1.05E-03	1637	PCNP	Ralstonia insidiosa	-0.260	1.79E-08	2370	TAOK1	Ralstonia insidiosa	-0.219	1.45E-03
172	ARL6IP6	Caulobacteraceae	-0.353	1.42E-04	905	FRS2	Porphyromonadaceae	0.456	1.52E-03	1638	PCNX1	Porphyromonadaceae	0.424	2.66E-08	2371	TAOK2	Ralstonia insidiosa	0.218	2.11E-06
173	ARL8B	Ralstonia insidiosa	-0.321	9.70E-10	906	FRY	Ralstonia insidiosa	-0.188	5.46E-03	1639	PCNX3	Ralstonia insidiosa	0.206	1.49E-03	2372	TAP2	Methylorubrum populi	0.146	3.54E-04
174	ARMC5	Bacillales	-0.488	1.64E-03	907	FSCN1	Ralstonia insidiosa	0.222	1.34E-03	1640	PCNX4	Caulobacteraceae	-0.303	4.29E-03	2373	TAPT1	Ralstonia insidiosa	-0.262	8.37E-07
175	ARMCX1	Streptococcus thermophilus	-0.100	4.18E-03	908	FSIP2	Brucella	-0.079	9.23E-05	1641	PCNX4	Enterobacteriaceae	-0.282	1.87E-03	2374	TARDBP	Betaproteobacteria	-0.273	1.21E-03
176	ARMCX4	Alphaproteobacteria	-0.475	1.53E-03	909	FSIP2	Caulobacteraceae	-0.291	5.41E-03	1642	PCSK4	Bacillales	-0.443	3.59E-05	2375	TARDBP	Methyloversatilis sp. RAC08	0.258	7.85E-07
177	ARMT1	Ralstonia insidiosa	-0.323	2.88E-20	910	FTCD	Bacillales	-0.453	8.66E-04	1643	PDCD10	Ralstonia insidiosa	-0.301	2.90E-11	2376	TARDBP	Microbacteriaceae	-0.265	6.69E-04
178	ARPC1B	Alphaproteobacteria	0.266	7.46E-04	911	FTH1	Malassezia vespertilionis	-0.212	5.80E-03	1644	PDCD6IP	Ralstonia insidiosa	-0.210	2.97E-06	2377	TARDBP	Ralstonia insidiosa	-0.219	9.24E-06
179	ARPC4	Cloacibacterium normanense	-0.366	7.09E-03	912	FTH1	Ralstonia insidiosa	0.320	2.75E-06	1645	PDE6A	Herbaspirillum huttiense	0.002	7.01E-05	2378	TARS2	Streptococcus thermophilus	0.212	5.20E-03
180	ARPP19	Saccharomycetales	0.495	3.27E-04	913	FTO	Geodermatophilaceae	0.414	9.49E-04	1646	PDE8A	Ralstonia insidiosa	-0.184	6.75E-03	2379	TAS2R30	Methyloversatilis sp. RAC08	0.351	2.44E-03
181	ARRDC1	Bacillales	-0.473	7.83E-04	914	FUBP1	Nocardioides	-0.163	6.72E-03	1647	PDIA6	Brevibacteriaceae	-0.143	1.35E-03	2380	TAS2R43	Rhodoferax	-0.185	7.61E-03
182	ARVCF	Bacillales	-0.442	9.12E-03	915	FUCA1	Acidovorax sp. KKS102	0.256	2.35E-03	1648	PDIK1L	Ralstonia insidiosa	-0.267	3.12E-06	2381	TASOR	Ralstonia insidiosa	-0.224	2.67E-07
183	ARVCF	Ralstonia insidiosa	0.257	1.92E-04	916	FYB2	Betaproteobacteria	-0.366	5.54E-04	1649	PDLIM2	Bacillales	-0.353	7.60E-04	2382	TBC1D10A	Bacillales	-0.396	4.30E-03
184	ASCC3	Bacillales	0.525	8.61E-03	917	FZD6	Clostridiaceae	0.377	6.19E-03	1650	PDLIM2	Ralstonia insidiosa	0.244	9.49E-04	2383	TBC1D12	Ralstonia insidiosa	-0.236	4.85E-07
185	ASF1A	Ralstonia insidiosa	-0.310	1.62E-07	918	G2E3	Caulobacteraceae	-0.373	1.20E-03	1651	PDPR	Campylobacteraceae	-0.229	8.35E-05	2384	TBC1D15	Ralstonia insidiosa	-0.203	2.95E-10
186	ASL	Betaproteobacteria	0.236	2.33E-04	919	G2E3	Ralstonia insidiosa	-0.247	1.09E-06	1652	PDS5B	Enterobacteriaceae	-0.274	5.13E-03	2385	TBC1D17	Ralstonia insidiosa	0.274	1.33E-11
187	ASPSCR1	Bacillales	-0.415	4.92E-04	920	G3BP2	Porphyromonadaceae	0.501	4.95E-06	1653	PDZD4	Acidovorax sp. KKS102	-0.275	5.85E-03	2386	TBC1D2	Bacillales	-0.360	1.12E-03
188	ATAD1	Ralstonia insidiosa	-0.265	1.18E-04	921	GABPA	Ralstonia insidiosa	-0.261	2.97E-06	1654	PDZD8	Porphyromonadaceae	0.506	3.03E-04	2387	TBC1D23	Ralstonia insidiosa	-0.248	1.92E-11
189	ATAD2	Ralstonia insidiosa	-0.160	5.09E-03	922	GABPB1	Malassezia restricta	0.327	5.81E-03	1655	PECAM1	Streptococcus thermophilus	-0.091	4.30E-03	2388	TBC1D8B	Porphyromonadaceae	0.462	9.35E-03
190	ATG12	Malassezia restricta	0.330	2.06E-03	923	GAK	Ralstonia insidiosa	0.247	3.80E-11	1656	PELP1	Ralstonia insidiosa	0.284	2.32E-06	2389	TBC1D8B	Ralstonia insidiosa	-0.257	7.74E-06
191	ATG13	Dermacoccaceae	-0.312	3.90E-03	924	GALNT1	Bacillales	0.404	8.74E-03	1657	PER2	Corynebacterium accolens	0.105	3.95E-03	2390	TBC1D9	Ralstonia insidiosa	-0.178	6.27E-03
192	ATG2A	Ralstonia insidiosa	0.214	1.47E-04	925	GALNT1	Ralstonia insidiosa	-0.313	1.17E-06	1658	PERP	Ralstonia insidiosa	-0.269	6.76E-03	2391	TBCD	Malasseziales	-0.375	4.88E-05
193	ATG2B	Ralstonia insidiosa	-0.177	8.05E-09	926	GAN	Yersiniaceae	-0.186	1.25E-03	1659	PEX1	Ralstonia insidiosa	-0.217	3.51E-03	2392	TBCE	Methyloversatilis sp. RAC08	0.234	1.86E-04
194	ATG4C	Ralstonia insidiosa	-0.301	9.87E-10	927	GARIN1A	Acidovorax sp. KKS102	-0.320	2.41E-03	1660	PEX11B	Ralstonia insidiosa	-0.329	1.91E-04	2393	TBCK	Ralstonia insidiosa	-0.177	8.53E-03
195	ATL2	Ralstonia insidiosa	-0.362	2.85E-09	928	GAS2L1	Ralstonia insidiosa	0.278	1.95E-06	1661	PEX11B	Sphingobacteriaceae	0.352	1.40E-08	2394	TBK1	Ralstonia insidiosa	-0.238	4.59E-04
196	ATN1	Ralstonia insidiosa	0.218	2.24E-08	929	GASK1B	Bacillales	0.498	4.91E-03	1662	PEX14	Bacillales	-0.441	2.05E-04	2395	TBL1XR1	Caulobacteraceae	-0.339	1.04E-04
197	ATP11C	Ralstonia insidiosa	-0.206	1.93E-03	930	GASK1B	Saccharomycetales	0.458	3.27E-03	1663	PEX19	Acidovorax sp. KKS102	0.275	3.70E-03	2396	TBL1XR1	Malassezia restricta	0.359	3.43E-03
198	ATP13A1	Ralstonia insidiosa	0.179	4.72E-08	931	GATAD2A	Ralstonia insidiosa	0.253	3.18E-05	1664	PEX19	Ascomycota	0.442	9.88E-03	2397	TBL1XR1	Microbacteriaceae	-0.347	8.72E-04
199	ATP13A3	Porphyromonadaceae	0.483	4.87E-03	932	GBP3	Prevotella melaninogenica	0.103	5.04E-03	1665	PEX3	Ralstonia insidiosa	-0.292	4.09E-09	2398	TBL1XR1	Moraxellaceae	-0.286	2.49E-03
200	ATP2C1	Ralstonia insidiosa	-0.288	1.18E-08	933	GC	Ottowia	0.407	2.85E-03	1666	PEX5	Enterobacteriaceae	0.219	6.72E-03	2399	TBL1XR1	Ralstonia insidiosa	-0.266	1.11E-05
201	ATP5F1B	Acidovorax sp. KKS102	0.301	5.75E-03	934	GCC2	Phyllobacteriaceae	0.518	1.56E-05	1667	PFKL	Ralstonia insidiosa	0.214	6.55E-04	2400	TBL3	Ralstonia insidiosa	0.277	7.85E-07
202	ATP5F1D	Ralstonia insidiosa	0.268	2.65E-04	935	GCLC	Ralstonia insidiosa	-0.220	4.60E-04	1668	PGAP3	Alcaligenaceae	-0.270	1.31E-03	2401	TBRG4	Lawsonellaceae	-0.333	3.81E-03
203	ATP5F1E	Streptococcus salivarius	-0.140	4.31E-03	936	GCLM	Ralstonia insidiosa	-0.213	8.98E-04	1669	PGBD2	Alphaproteobacteria	-0.358	4.11E-03	2402	TBXA2R	Ralstonia insidiosa	0.294	2.25E-05
204	ATP5MC3	Actinomyces naeslundii	-0.146	4.58E-04	937	GCNA	Alphaproteobacteria	-0.352	4.66E-03	1670	PGBD4	Alphaproteobacteria	-0.262	7.02E-03	2403	TC2N	Bacillales	0.379	2.83E-03
205	ATP6AP1	Caulobacteraceae	0.305	5.13E-03	938	GDA	Ralstonia insidiosa	-0.283	4.23E-03	1671	PGC	Gordonia bronchialis	-0.014	6.07E-04	2404	TCF12	Ralstonia insidiosa	-0.207	1.66E-05
206	ATP6AP2	Ralstonia insidiosa	-0.367	3.65E-16	939	GDAP2	Ralstonia insidiosa	-0.248	1.79E-04	1672	PGGT1B	Enterobacteriaceae	-0.296	2.28E-06	2405	TCF25	Ralstonia insidiosa	0.223	5.17E-05
207	ATP6V0A1	Caulobacteraceae	0.341	3.84E-03	940	GDF2	Ralstonia insidiosa	0.174	4.23E-04	1673	PGK1	Acidovorax sp. KKS102	0.296	1.02E-04	2406	TCF3	Debaryomycetaceae	-0.420	2.85E-04
208	ATP6V0A1	Malasseziales	-0.273	1.29E-04	941	GET4	Bacillales	-0.489	7.33E-03	1674	PGM1	Carnobacteriaceae	-0.160	4.39E-03	2407	TCF3	Ralstonia insidiosa	0.223	4.91E-10
209	ATP6V1A	Porphyromonadaceae	0.505	1.00E-03	942	GFM2	Acidovorax sp. KKS102	0.335	3.75E-04	1675	PGRMC2	Malasseziaceae	0.304	5.81E-04	2408	TCIRG1	Bacillales	-0.404	4.01E-04
210	ATP6V1A	Ralstonia insidiosa	-0.236	4.42E-08	943	GFOD1	Delftia acidovorans	0.130	1.34E-03	1676	PHETA1	Bacillales	-0.434	6.51E-03	2409	TCOF1	Debaryomycetaceae	-0.438	2.19E-04
211	ATP6V1C1	Ralstonia insidiosa	-0.208	1.63E-06	944	GGT5	Ralstonia insidiosa	0.256	5.37E-04	1677	PHF10	Methyloversatilis sp. RAC08	0.308	5.38E-03	2410	TCOF1	Ralstonia insidiosa	0.225	2.32E-04
212	ATP8B1	Ralstonia insidiosa	-0.257	1.78E-03	945	GIMAP6	Alphaproteobacteria	0.396	6.67E-03	1678	PHF14	Cloacibacterium normanense	0.086	2.32E-05	2411	TCTN3	Brevibacteriaceae	-0.102	7.11E-04
213	ATR	Ralstonia insidiosa	-0.133	6.20E-03	946	GIN1	Betaproteobacteria	-0.265	8.63E-03	1679	PHF20L1	Enterobacteriaceae	-0.292	6.33E-03	2412	TDG	Delftia acidovorans	0.232	9.25E-04

214	ATRX	Delftia acidovorans	0.447	7.11E-05	947	GIPC2	Microbacteriaceae	-0.312	3.61E-03	1680	PHF20L1	Phyllobacteriaceae	0.358	1.31E-08	2413	TDRD3	Alphaproteobacteria	-0.426	1.66E-03
215	ATRX	Phyllobacteriaceae	0.518	1.16E-05	948	GJD3	Ralstonia insidiosa	0.168	8.88E-05	1681	PHF6	Ralstonia insidiosa	-0.272	6.75E-08	2414	TEAD3	Ralstonia insidiosa	0.186	1.26E-05
216	ATXN2	Sphingobacteriaceae	-0.359	1.53E-03	949	GKAP1	Bacilli	-0.306	1.06E-03	1682	PHIP	Betaproteobacteria	-0.205	6.38E-07	2415	TEKTIP1	Ralstonia insidiosa	0.270	7.67E-07
217	ATXN7	Corynebacterium accolens	0.242	1.07E-03	950	GLCE	Ralstonia insidiosa	-0.312	1.06E-07	1683	PHIP	Enterobacteriaceae	-0.272	1.45E-03	2416	TELO2	Bacillales	-0.475	7.59E-06
218	AXIN1	Bacillales	-0.321	2.03E-03	951	GLG1	Bacilli	0.352	1.76E-03	1684	PHKA1	Cloacibacterium normanense	0.278	3.85E-04	2417	TELO2	Ralstonia insidiosa	0.273	2.13E-10
219	AXIN1	Ralstonia insidiosa	0.254	5.28E-05	952	GLMN	Betaproteobacteria	-0.319	2.68E-05	1685	PHLDB2	Porphyromonadaceae	0.444	9.97E-03	2418	TENM1	Delftia acidovorans	0.186	6.03E-06
220	B3GALT9	Alphaproteobacteria	-0.298	6.47E-03	953	GLMN	Microbacteriaceae	-0.298	2.07E-03	1686	PHLDB2	Ralstonia insidiosa	-0.273	2.25E-07	2419	TENT2	Ralstonia insidiosa	-0.288	3.79E-12
221	B4GALNT4	Streptococcus oralis	0.020	1.64E-03	954	GLO1	Ralstonia insidiosa	-0.297	4.79E-03	1687	PHLDB3	Bacillales	-0.473	2.98E-05	2420	TET2	Ralstonia insidiosa	-0.146	7.48E-05
222	BACH1	Porphyromonadaceae	0.481	5.68E-03	955	GLO1	Sphingobacteriaceae	0.341	2.69E-03	1688	PHRF1	Ralstonia insidiosa	0.266	1.55E-11	2421	TEX101	Herbaspirillum huttiense	-0.015	1.10E-11
223	BAG4	Ralstonia insidiosa	-0.238	7.68E-08	956	GLOD5	Phyllobacteriaceae	-0.293	3.13E-04	1689	PHYKPL	Saccharomycetales	-0.481	2.74E-03	2422	TFAM	Bacillales	0.446	8.09E-03
224	BAG6	Caulobacteraceae	0.304	6.73E-03	957	GLRX2	Ralstonia insidiosa	-0.251	2.15E-04	1690	PI16	Lactococcus lactis	-0.139	5.97E-04	2423	TFE3	Enterobacteriaceae	0.289	2.56E-03
225	BAG6	Enterobacteriaceae	0.282	5.27E-04	958	GLYATL3	Acinetobacter ursingii	0.291	2.74E-03	1691	PI4K2B	Ralstonia insidiosa	-0.233	5.79E-07	2424	TFEB	Bacillales	-0.477	1.66E-03
226	BAP1	Enterobacteriaceae	0.237	7.07E-05	959	GMFB	Caulobacteraceae	-0.373	6.96E-03	1692	PIAS4	Bacillales	-0.322	7.00E-05	2425	TFEB	Ralstonia insidiosa	0.270	3.21E-04
227	BAX	Porphyromonadaceae	-0.288	1.98E-03	960	GMFB	Ralstonia insidiosa	-0.343	1.11E-09	1693	PIAS4	Ralstonia insidiosa	0.270	5.75E-07	2426	TFPT	Cloacibacterium normanense	-0.279	6.88E-03
228	BAZ1A	Phyllobacteriaceae	0.300	2.00E-07	961	GMIP	Ralstonia insidiosa	0.243	6.38E-03	1694	PICALM	Bacillales	0.503	7.63E-03	2427	TG	Pseudomonas oleovorans	-0.258	2.67E-03
229	BAZ1B	Delftia acidovorans	0.399	2.21E-06	962	GNA11	Ralstonia insidiosa	0.207	3.75E-03	1695	PICALM	Microbacteriaceae	-0.377	1.34E-03	2428	TGFB1	Ralstonia insidiosa	0.196	2.32E-03
230	BAZ1B	Phyllobacteriaceae	0.389	3.34E-04	963	GNA13	Caulobacterales	-0.382	5.62E-05	1696	PIGA	Ralstonia insidiosa	-0.218	2.17E-05	2429	TGFB1I1	Ralstonia insidiosa	0.292	1.33E-03
231	BAZ2B	Delftia acidovorans	0.434	6.45E-05	964	GNA13	Ralstonia insidiosa	-0.297	4.42E-08	1697	PIGB	Ralstonia insidiosa	-0.239	2.12E-03	2430	TGFBR1	Ralstonia insidiosa	-0.248	5.52E-04
232	BBLN	Brevibacteriaceae	0.109	3.99E-03	965	GNA13	Saccharomycetales	0.468	5.36E-06	1698	PIGK	Ralstonia insidiosa	-0.220	1.90E-03	2431	TGFBR3L	Geodermatophilaceae	-0.401	9.20E-03
233	BBLN	Ralstonia insidiosa	0.305	8.81E-08	966	GNAI1	Ralstonia insidiosa	-0.216	6.69E-05	1699	PIGP	Ralstonia insidiosa	-0.233	7.18E-04	2432	THAP12	Bacillales	0.410	9.46E-03
234	BBOX1	Carnobacteriaceae	-0.044	4.65E-03	967	GNAI2	Acidobacteriota	-0.219	2.59E-03	1700	PIGW	Epilithonimonas	0.284	3.19E-04	2433	THAP12	Ralstonia insidiosa	-0.283	3.34E-05
235	BBS10	Ralstonia insidiosa	-0.266	2.20E-06	968	GNAI2	Ralstonia insidiosa	0.142	4.95E-05	1701	PIK3AP1	Ralstonia insidiosa	-0.239	4.86E-03	2434	THAP2	Sphaerotilaceae	-0.304	2.59E-03
236	BCAM	Ralstonia insidiosa	0.185	3.10E-03	969	GNAQ	Microbacteriaceae	-0.400	9.29E-03	1702	PIK3CA	Ralstonia insidiosa	-0.196	4.85E-07	2435	THAP6	Ralstonia insidiosa	-0.238	2.32E-03
237	BCAP29	Brucella	-0.079	4.81E-03	970	GNAQ	Ralstonia insidiosa	-0.239	5.07E-12	1703	PIK3R1	Porphyromonadaceae	0.453	6.32E-06	2436	THAP7	Ralstonia insidiosa	0.212	1.34E-03
238	BCAP31	Cloacibacterium normanense	-0.206	8.76E-05	971	GNAS	Corynebacterium accolens	-0.258	1.07E-04	1704	PIK3R2	Enterobacteriaceae	0.258	3.87E-03	2437	THAP8	Ralstonia insidiosa	0.188	6.86E-03
239	BCAR1	Bacillales	-0.388	6.25E-04	972	GNAS	Ralstonia insidiosa	0.176	2.08E-03	1705	PIK3R4	Porphyromonadaceae	0.423	1.47E-04	2438	THAP9	Ralstonia insidiosa	-0.219	7.24E-06
240	BCAR1	Ralstonia insidiosa	0.290	2.56E-12	973	GNB1L	Ralstonia insidiosa	0.315	3.05E-05	1706	PIM3	Brevibacteriaceae	0.115	4.61E-03	2439	THEM4	Alphaproteobacteria	-0.323	1.49E-03
241	BCL2L12	Porphyromonadaceae	-0.439	3.84E-03	974	GNB2	Ralstonia insidiosa	0.198	7.56E-03	1707	PINK1	Alphaproteobacteria	0.372	5.46E-03	2440	THOC6	Ralstonia insidiosa	0.216	1.17E-03
242	BCL6	Alphaproteobacteria	-0.314	5.97E-04	975	GNB4	Bacillales	0.351	3.95E-03	1708	PINLYP	Herbaspirillum huttiense	-0.150	5.03E-04	2441	THOP1	Bacillales	-0.400	2.36E-04
243	BCL6B	Streptococcus thermophilus	-0.143	4.13E-03	976	GNB4	Saccharomycetales	0.371	2.22E-04	1709	PIP5K1C	Debaryomycetaceae	-0.314	1.87E-03	2442	THUMPD1	Ralstonia insidiosa	-0.152	1.27E-05
244	BCL7B	Ralstonia insidiosa	0.182	1.68E-03	977	GNL3L	Debaryomycetaceae	-0.095	1.65E-03	1710	PITPNM1	Ralstonia insidiosa	0.206	1.21E-07	2443	TIA1	Ralstonia insidiosa	-0.239	6.22E-05
245	BCLAF1	Enterobacteriaceae	-0.232	5.44E-04	978	GNL3L	Phyllobacteriaceae	0.144	5.83E-03	1711	PJA2	Porphyromonadaceae	0.488	3.12E-03	2444	TICAM1	Ralstonia insidiosa	0.252	6.51E-04
246	BCLAF1	Ralstonia insidiosa	-0.247	1.89E-09	979	GNRHR	Acidovorax sp. KKS102	-0.120	7.87E-05	1712	PKN1	Ralstonia insidiosa	0.210	1.83E-06	2445	TIMM29	Ralstonia insidiosa	0.232	4.23E-03
247	BCLAF3	Alphaproteobacteria	-0.218	6.12E-03	980	GOLGA5	Brevibacteriaceae	-0.122	1.90E-03	1713	PKN2	Ralstonia insidiosa	-0.238	1.11E-09	2446	TIPRL	Ralstonia insidiosa	-0.306	3.69E-04
248	BCR	Ralstonia insidiosa	0.228	2.41E-04	981	GOLGA8N	Sphingosinicellaceae	-0.309	1.19E-03	1714	PLA2G4A	Bacillales	0.431	1.67E-04	2447	TJP3	Ralstonia insidiosa	0.170	3.76E-04
249	BDH2	Alphaproteobacteria	-0.325	2.42E-03	982	GOLGB1	Actinomyces naeslundii	0.291	9.09E-03	1715	PLA2G6	Oscillospiraceae	-0.402	9.39E-03	2448	TKFC	Bacillales	-0.308	1.05E-03
250	BECN1	Acidovorax sp. KKS102	0.328	2.80E-03	983	GOLIM4	Streptococcus salivarius	-0.001	9.84E-03	1716	PLA2R1	Brucella	-0.111	6.01E-04	2449	TKFC	Staphylococcus capitis	-0.247	5.67E-03
251	BECN1	Brevibacteriaceae	-0.073	1.41E-05	984	GOLM2	Ralstonia insidiosa	-0.251	4.84E-05	1717	PLAC8L1	Alphaproteobacteria	-0.397	4.44E-06	2450	TLE2	Brevundimonas sp.	-0.323	4.57E-03
252	BEND4	Nocardioides	-0.214	5.74E-04	985	GOLT1B	Ralstonia insidiosa	-0.323	2.43E-06	1718	PLBD2	Caulobacteraceae	0.233	5.92E-03	2451	TLE2	Ralstonia insidiosa	0.210	4.92E-07
253	BGN	Ralstonia insidiosa	0.226	1.95E-04	986	GON4L	Sphingobacteriaceae	-0.254	9.53E-03	1719	PLCB3	Ralstonia insidiosa	0.227	5.26E-10	2452	TLR1	Bacillales	0.423	2.16E-03
254	BICRA	Ralstonia insidiosa	0.311	5.76E-13	987	GON7	Leptotrichiaceae	0.176	9.97E-03	1720	PLCD1	Betaproteobacteria	0.321	1.28E-03	2453	TLR1	Saccharomycetales	0.421	1.00E-03
255	BID	Polyangiales	-0.307	7.34E-03	988	GOPC	Ralstonia insidiosa	-0.187	2.92E-07	1721	PLCE1	Brucella	-0.140	3.00E-03	2454	TM2D1	Ralstonia insidiosa	-0.241	6.21E-03
256	BIRC2	Caulobacterales	-0.334	9.07E-03	989	GPBP1	Ralstonia insidiosa	-0.252	6.12E-07	1722	PLCE1	Polyangiales	0.307	4.02E-03	2455	TM2D3	Leptotrichiaceae	0.141	2.69E-04
257	BIRC2	Ralstonia insidiosa	-0.314	1.04E-14	990	GPC1	Ralstonia insidiosa	0.196	2.42E-03	1723	PLCL1	Gordonia bronchialis	0.246	9.94E-04	2456	TM2D3	Ralstonia insidiosa	-0.263	2.51E-03
258	BIVM	Malassezia restricta	0.340	8.13E-03	991	GPER1	Bacillales	-0.368	1.95E-03	1724	PLD1	Ralstonia insidiosa	-0.185	3.75E-03	2457	TM9SF2	Ralstonia insidiosa	-0.334	1.35E-12
259	BIVM	Malassezia vespertilionis	0.364	5.20E-03	992	GPM6A	Comamonadaceae	-0.294	8.00E-03	1725	PLEKHA8	Ralstonia insidiosa	-0.230	7.90E-04	2458	TM9SF3	Caulobacteraceae	-0.366	5.07E-04
260	BLCAP	Leptotrichiaceae	0.055	1.20E-03	993	GPR21	Herbaspirillum huttiense	-0.081	6.73E-03	1726	PLEKHF2	Caulobacteraceae	-0.377	5.46E-03	2459	TM9SF3	Microbacteriaceae	-0.378	1.19E-04
261	BLID	Brucella	-0.207	7.40E-04	994	GPR65	Saccharomycetales	0.338	5.59E-03	1727	PLEKHF2	Ralstonia insidiosa	-0.275	2.38E-08	2460	TM9SF3	Ralstonia insidiosa	-0.352	3.68E-14
262	BLOC1S2	Ralstonia insidiosa	-0.350	1.80E-03	995	GPR89B	Alphaproteobacteria	-0.346	7.31E-03	1728	PLEKHG4B	Mycobacteroides	0.074	5.37E-05	2461	TMA16	Ralstonia insidiosa	-0.226	1.82E-03
263	BMP2K	Cloacibacterium normanense	0.360	3.61E-03	996	GPRC5C	Betaproteobacteria	0.314	1.44E-04	1729	PLEKHG5	Ralstonia insidiosa	0.233	2.41E-03	2462	TMC6	Ralstonia insidiosa	0.228	1.02E-05
264	BMPR2	Saccharomycetales	0.460	7.39E-03	997	GPSM1	Epilithonimonas	-0.363	6.45E-03	1730	PLEKHH2	Brucella	-0.154	9.94E-03	2463	TMCO1	Ralstonia insidiosa	-0.299	1.30E-06
265	BMT2	Ralstonia insidiosa	-0.210	2.02E-04	998	GPX1	Alphaproteobacteria	0.238	5.61E-04	1731	PLEKHH3	Bacillales	-0.505	2.99E-04	2464	TMCO1	Sphingobacteriaceae	0.381	7.11E-04
266	BNC2	Brucella	-0.071	8.28E-03	999	GRAMD1A	Ralstonia insidiosa	0.291	6.37E-05	1732	PLEKHH3	Ralstonia insidiosa	0.266	4.44E-06	2465	TMED10	Bacillales	0.467	4.97E-03
267	BNIP2	Bacillales	0.407	5.02E-03	1000	GRAMD1C	Ralstonia insidiosa	-0.206	1.19E-06	1733	PLEKHM2	Betaproteobacteria	0.341	3.02E-03	2466	TMED2	Ralstonia insidiosa	-0.381	5.36E-06

268	BNIP2	Ralstonia insidiosa	-0.245	5.84E-08	1001	GRB14	Ralstonia insidiosa	-0.191	3.01E-04	1734	PLEKHM2	Ralstonia insidiosa	0.176	6.39E-07	2467	TMED7	Moraxellaceae	-0.314	3.19E-03
269	BNIP3L	Ralstonia insidiosa	-0.265	2.66E-06	1002	GRHL2	Rothia	0.177	6.67E-03	1735	PLK3	Nitrosomonadales	-0.171	9.86E-07	2468	TMED7	Ralstonia insidiosa	-0.309	1.21E-07
270	BOD1L1	Delftia acidovorans	0.379	1.07E-07	1003	GRIN2D	Achromobacter	-0.279	5.43E-03	1736	PLOD1	Caulobacteraceae	0.342	5.60E-05	2469	TMEM106B	Caulobacteraceae	-0.389	2.86E-03
271	BOD1L1	Phyllobacteriaceae	0.399	1.22E-04	1004	GRK2	Ralstonia insidiosa	0.190	5.23E-08	1737	PLOD2	Ralstonia insidiosa	-0.263	5.10E-03	2470	TMEM106B	Moraxellaceae	-0.334	4.19E-03
272	BOD1L1	Staphylococcus saprophyticus	0.081	9.65E-04	1005	GRK2	Sclerotiniaceae	-0.251	6.44E-04	1738	PLOD3	Caulobacteraceae	0.248	6.36E-03	2471	TMEM106B	Ralstonia insidiosa	-0.271	4.32E-09
273	BPNT2	Ralstonia insidiosa	-0.297	2.69E-04	1006	GRTP1	Bacillales	-0.462	5.04E-03	1739	PLPBP	Sphingobacteriaceae	0.405	2.92E-04	2472	TMEM123	Ralstonia insidiosa	-0.298	2.01E-06
274	BPTF	Delftia acidovorans	0.313	3.13E-05	1007	GSDMB	Alphaproteobacteria	-0.506	8.24E-03	1740	PML	Debaryomycetaceae	-0.346	1.16E-04	2473	TMEM165	Pasteurellaceae	-0.113	2.22E-03
275	BPTF	Phyllobacteriaceae	0.300	2.59E-04	1008	GSDMB	Oscillospiraceae	-0.498	2.32E-04	1741	PML	Ralstonia insidiosa	0.248	7.34E-08	2474	TMEM167A	Bacillales	0.393	3.51E-03
276	BRAT1	Bacillales	-0.425	3.64E-05	1009	GSG1L	Lachnospiraceae	0.059	3.89E-03	1742	PNPLA4	Rothia mucilaginosa	-0.191	7.85E-04	2475	TMEM167A	Ralstonia insidiosa	-0.418	1.57E-12
277	BRAT1	Ralstonia insidiosa	0.324	4.76E-06	1010	GSK3A	Enterobacteriaceae	0.184	3.70E-03	1743	PNPLA8	Bacillales	0.435	4.62E-03	2476	TMEM167B	Ralstonia insidiosa	-0.334	1.15E-08
278	BRD1	Prevotella melaninogenica	-0.302	4.50E-03	1011	GTF2A1	Ralstonia insidiosa	-0.272	1.97E-07	1744	PNPT1	Ralstonia insidiosa	-0.174	3.47E-04	2477	TMEM168	Caulobacteraceae	-0.321	5.53E-04
279	BRD4	Ralstonia insidiosa	0.273	3.31E-04	1012	GTF2F1	Malassezia vespertilionis	-0.313	6.10E-03	1745	PNRC2	Ralstonia insidiosa	-0.286	6.53E-10	2478	TMEM168	Malasseziales	0.315	1.94E-03
280	BRF1	Ralstonia insidiosa	0.267	6.29E-05	1013	GTF2H5	Leptotrichiaceae	0.065	1.59E-04	1746	POC1B	Ralstonia insidiosa	-0.312	2.15E-04	2479	TMEM168	Ralstonia insidiosa	-0.216	1.55E-11
281	BRMS1L	Ralstonia insidiosa	-0.272	6.66E-13	1014	GTF3A	Corynebacterium accolens	-0.193	8.43E-03	1747	POLD1	Ralstonia insidiosa	0.218	4.00E-03	2480	TMEM170B	Ralstonia insidiosa	-0.293	1.10E-04
282	BRWD1	Ralstonia insidiosa	-0.187	1.42E-07	1015	GTF3C3	Ralstonia insidiosa	-0.222	1.08E-08	1748	POLE3	Ralstonia insidiosa	-0.339	6.74E-05	2481	TMEM181	Porphyromonadaceae	0.461	2.08E-03
283	BSCL2	Thermaceae	0.218	1.88E-03	1016	GTPBP10	Caulobacteraceae	-0.319	6.10E-03	1749	POLE3	Sphingobacteriaceae	0.397	3.12E-06	2482	TMEM181	Ralstonia insidiosa	-0.294	4.77E-04
284	BTAF1	Ralstonia insidiosa	-0.226	8.39E-04	1017	GTPBP10	Ralstonia insidiosa	-0.235	1.41E-06	1750	POLG	Betaproteobacteria	0.405	7.62E-03	2483	TMEM182	Sphingobacteriales	0.278	2.78E-03
285	BTBD2	Ralstonia insidiosa	0.203	6.32E-05	1018	GUF1	Ralstonia insidiosa	-0.319	1.10E-07	1751	POLI	Ralstonia insidiosa	-0.178	4.43E-04	2484	TMEM184B	Debaryomycetaceae	-0.192	9.49E-05
286	BTBD3	Ralstonia insidiosa	-0.156	6.73E-03	1019	GUSB	Caulobacteraceae	0.268	5.07E-04	1752	POLR1F	Enterobacteriaceae	-0.305	1.89E-06	2485	TMEM184B	Ralstonia insidiosa	0.192	7.97E-05
287	BTRC	Actinomyces naeslundii	0.270	9.84E-03	1020	GYS2	Ralstonia insidiosa	-0.252	2.22E-04	1753	POLR2A	Debaryomycetaceae	-0.225	7.18E-04	2486	TMEM221	Pseudomonas oleovorans	-0.224	6.18E-03
288	BZW1	Caulobacterales	-0.356	9.57E-04	1021	H1-3	Herbaspirillum huttiense	0.020	5.45E-04	1754	POLR2B	Porphyromonadaceae	0.478	8.31E-03	2487	TMEM230	Ralstonia insidiosa	-0.370	2.34E-05
289	BZW1	Ralstonia insidiosa	-0.308	4.49E-09	1022	H2BC10	Methylorubrum	0.220	1.33E-10	1755	POLR2E	Ralstonia insidiosa	0.170	7.38E-04	2488	TMEM238	Porphyromonadaceae	-0.354	6.70E-05
290	C10orf88	Ralstonia insidiosa	-0.217	3.68E-03	1023	H2BC10	Methyloversatilis sp. RAC08	-0.313	3.32E-05	1756	POLR2L	Ralstonia insidiosa	0.232	5.06E-03	2489	TMEM25	Colletotrichum lupini	0.234	3.33E-03
291	C12orf29	Ralstonia insidiosa	-0.257	1.83E-07	1024	H3-3A	Actinomyces naeslundii	-0.135	3.12E-03	1757	POLRMT	Bacillales	-0.423	7.33E-04	2490	TMEM250	Ralstonia insidiosa	0.138	2.13E-04
292	C12orf4	Ralstonia insidiosa	-0.199	7.45E-03	1025	H3-3A	Brevibacteriaceae	-0.153	3.89E-04	1758	POMK	Cloacibacterium normanense	0.393	3.44E-04	2491	TMEM254	Ralstonia insidiosa	-0.328	2.08E-04
293	C14orf119	Leptotrichiaceae	0.127	1.41E-04	1026	HAT1	Ralstonia insidiosa	-0.262	2.98E-09	1759	PON2	Methyloversatilis sp. RAC08	0.199	9.84E-03	2492	TMEM260	Ralstonia insidiosa	-0.213	4.40E-06
294	C14orf119	Sclerotiniaceae	0.248	2.74E-04	1027	HAUS3	Betaproteobacteria	-0.307	6.52E-03	1760	PON2	Ralstonia insidiosa	-0.326	4.42E-06	2493	TMEM265	Alphaproteobacteria	-0.460	4.49E-03
295	C17orf80	Micromonosporaceae	0.450	7.00E-03	1028	HAUS6	Ralstonia insidiosa	-0.243	4.60E-09	1761	POR	Nocardioides	0.182	2.93E-03	2494	TMEM30A	Ralstonia insidiosa	-0.299	5.02E-06
296	C18orf25	Ralstonia insidiosa	-0.171	1.66E-04	1029	HCAR3	Viruses	0.320	6.09E-03	1762	POT1	Caulobacteraceae	-0.356	6.17E-03	2495	TMEM30B	Ralstonia insidiosa	-0.260	9.24E-03
297	C18orf32	Corynebacterium accolens	0.092	3.00E-04	1030	HDAC10	Bacillales	-0.464	1.28E-03	1763	POT1	Ralstonia insidiosa	-0.311	3.34E-09	2496	TMEM33	Malasseziales	0.362	9.18E-06
298	C1GALT1	Caulobacteraceae	-0.291	6.50E-03	1031	HDAC10	Ralstonia insidiosa	0.257	1.16E-13	1764	POU2F1	Delftia acidovorans	0.337	6.03E-07	2497	TMEM42	Leptotrichiaceae	0.052	3.42E-03
299	C1GALT1	Ralstonia insidiosa	-0.218	8.44E-05	1032	HDAC3	Phyllobacteriaceae	-0.381	1.97E-07	1765	POU5F1B	Sphingosinicellaceae	-0.246	2.84E-03	2498	TMEM45A	Ralstonia insidiosa	-0.278	5.16E-04
300	C1GALT1C1	Ralstonia insidiosa	-0.288	6.78E-07	1033	HEATR1	Porphyromonadaceae	0.461	6.25E-03	1766	POU5F2	Herbaspirillum huttiense	0.063	6.91E-10	2499	TMEM59	Sphingobacteriaceae	0.384	5.56E-03
301	C1orf56	Ralstonia insidiosa	0.239	3.04E-05	1034	HECA	Microbacteriaceae	-0.372	2.71E-03	1767	PPA2	Ralstonia insidiosa	-0.229	1.31E-03	2500	TMEM60	Dermacoccaceae	0.323	2.03E-03
302	C1QTNF1	Ralstonia insidiosa	0.236	3.37E-04	1035	HECA	Ralstonia insidiosa	-0.263	4.79E-08	1768	PPAN	Bacillales	-0.346	5.21E-03	2501	TMEM60	Ralstonia insidiosa	-0.290	3.99E-07
303	C1R	Gardnerella	-0.282	5.46E-03	1036	HECTD2	Betaproteobacteria	-0.261	3.45E-03	1769	PPARD	Ralstonia insidiosa	0.175	1.34E-04	2502	TMEM64	Ralstonia insidiosa	-0.321	9.48E-06
304	C2	Nitrobacteraceae	-0.269	4.52E-04	1037	HELQ	Ralstonia insidiosa	-0.228	7.57E-04	1770	PPCS	Ralstonia insidiosa	-0.280	4.45E-05	2503	TMEM65	Comamonadaceae	-0.376	7.91E-03
305	C21orf58	Ralstonia insidiosa	0.263	8.70E-04	1038	HERC3	Cloacibacterium normanense	0.444	3.14E-03	1771	PPDPF	Ralstonia insidiosa	0.219	1.62E-05	2504	TMEM82	Bacillales	-0.388	1.36E-03
306	C21orf91	Ralstonia insidiosa	-0.257	6.38E-07	1039	HERC4	Ralstonia insidiosa	-0.121	9.38E-07	1772	PPEF2	Methylocystaceae	-0.078	2.45E-03	2505	TMEM87B	Ralstonia insidiosa	-0.289	1.77E-06
307	C2CD5	Microbacteriaceae	-0.360	4.60E-03	1040	HEXD	Bacillales	-0.527	2.37E-03	1773	PPFIA3	Bacillales	-0.370	2.02E-04	2506	TMOD3	Colletotrichum lupini	-0.229	8.00E-03
308	C2CD5	Ralstonia insidiosa	-0.261	4.63E-08	1041	HGS	Bacillales	-0.368	2.80E-03	1774	PPFIA3	Ralstonia insidiosa	0.291	4.25E-10	2507	TMTC3	Ralstonia insidiosa	-0.215	2.12E-05
309	C2orf69	Ralstonia insidiosa	-0.198	9.47E-06	1042	HGS	Ralstonia insidiosa	0.235	5.85E-11	1775	PPID	Epilithonimonas	0.255	1.38E-03	2508	TMX1	Ralstonia insidiosa	-0.307	2.84E-10
310	C2orf88	Cellvibrionales	-0.192	2.57E-04	1043	HIPK3	Ralstonia insidiosa	-0.230	2.09E-07	1776	PPID	Ralstonia insidiosa	-0.275	1.96E-04	2509	TMX3	Ralstonia insidiosa	-0.269	1.29E-04
311	C2orf92	Acidovorax sp. KKS102	-0.276	8.67E-06	1044	HLX	Pasteurellaceae	0.148	1.50E-03	1777	PPIG	Enterobacteriaceae	-0.293	4.56E-04	2510	TMX4	Ralstonia insidiosa	-0.278	5.71E-04
312	C3orf22	Staphylococcus saprophyticus	0.252	7.68E-03	1045	HMBOX1	Delftia acidovorans	0.472	9.48E-06	1778	PPIG	Phyllobacteriaceae	0.441	4.05E-07	2511	TNFAIP1	Acidovorax sp. KKS102	0.273	5.93E-04
313	C4orf3	Actinomyces naeslundii	-0.137	1.90E-08	1046	HMCES	Caulobacteraceae	0.329	6.93E-03	1779	PPIP5K2	Ralstonia insidiosa	-0.222	5.70E-07	2512	TNFRSF1A	Ralstonia insidiosa	0.207	2.71E-03
314	C4orf46	Ralstonia insidiosa	-0.273	8.06E-04	1047	HMG20A	Ralstonia insidiosa	-0.212	4.39E-07	1780	PPP1CB	Caulobacteraceae	-0.340	1.48E-03	2513	TNFRSF25	Comamonas aquatica	-0.361	1.35E-03
315	C5	Porphyromonadaceae	0.379	2.90E-03	1048	HMGN4	Ralstonia insidiosa	-0.277	2.41E-03	1781	PPP1CB	Microbacteriaceae	-0.366	2.86E-03	2514	TNFSF12	Ralstonia insidiosa	0.273	5.35E-03
316	C5orf15	Malasseziales	0.323	3.63E-05	1049	HMGN5	Phyllobacteriaceae	0.236	1.47E-04	1782	PPP1CB	Ralstonia insidiosa	-0.253	1.06E-12	2515	TNIP1	Enterobacteriaceae	0.222	9.34E-03
317	C5orf15	Ralstonia insidiosa	-0.284	1.75E-05	1050	HMGXB4	Delftia acidovorans	0.296	7.67E-03	1783	PPP1CC	Debaryomycetaceae	0.366	5.01E-03	2516	TNIP1	Ralstonia insidiosa	0.186	1.52E-03
318	C5orf22	Ralstonia insidiosa	-0.253	5.01E-05	1051	HNF4A	Enterobacteriaceae	0.254	4.04E-03	1784	PPP1CC	Ralstonia insidiosa	-0.290	3.24E-08	2517	TNK2	Ralstonia insidiosa	0.238	1.36E-06
319	C6orf120	Caulobacteraceae	-0.411	8.73E-03	1052	HNMT	Ralstonia insidiosa	-0.217	3.09E-04	1785	PPP1R12A	Enterobacteriaceae	-0.245	1.36E-03	2518	TNKS	Ralstonia insidiosa	-0.200	5.80E-03
320	C6orf226	Bacillales	-0.333	9.85E-03	1053	HNRNPA1L3	Ralstonia insidiosa	-0.311	6.34E-04	1786	PPP1R13L	Bacillales	-0.456	1.85E-03	2519	TNKS2	Ralstonia insidiosa	-0.181	7.27E-08
321	C6orf62	Ralstonia insidiosa	-0.270	8.15E-05	1054	HNRNPA2B1	Nevskiales	0.273	7.13E-06	1787	PPP1R13L	Ralstonia insidiosa	0.324	6.97E-06	2520	TNN	Pseudomonas oleovorans	-0.102	8.91E-04

322	C9orf72	Caulobacterales	-0.361	5.92E-03	1055	HNRNPA2B1	Ralstonia insidiosa	-0.215	4.49E-06	1788	PPP1R15B	Ralstonia insidiosa	-0.231	2.97E-05	2521	TNRC18	Debaryomycetaceae	-0.326	5.00E-04
323	C9orf72	Ralstonia insidiosa	-0.249	1.04E-07	1056	HNRNPC	Debaryomycetaceae	0.341	6.79E-03	1789	PPP1R16A	Bacillales	-0.445	1.10E-03	2522	TNRC18	Ralstonia insidiosa	0.242	1.43E-05
324	C9orf78	Cloacibacterium normanense	-0.077	3.52E-03	1057	HNRNPC	Ralstonia insidiosa	-0.269	9.51E-05	1790	PPP1R2	Moraxellaceae	-0.344	6.11E-03	2523	TNRC6B	Delftia acidovorans	0.390	1.42E-18
325	CABIN1	Debaryomycetaceae	-0.291	7.98E-03	1058	HNRNPD	Alphaproteobacteria	-0.514	6.80E-04	1791	PPP1R37	Malassezia vespertilionis	-0.310	6.87E-03	2524	TNXB	Ralstonia insidiosa	0.237	1.05E-04
326	CACTIN	Microbacteriaceae	0.344	1.27E-05	1059	HNRNPLL	Haemophilus parainfluenzae	0.023	1.03E-03	1792	PPP1R3E	Alphaproteobacteria	-0.425	5.85E-04	2525	TOLLIP	Ralstonia insidiosa	0.165	2.20E-03
327	CACTIN	Ralstonia insidiosa	0.240	1.93E-14	1060	HNRNPUL2	Alphaproteobacteria	-0.484	7.59E-07	1793	PPP1R9B	Methyloversatilis sp. RAC08	-0.348	1.90E-03	2526	TOM1	Bacillales	-0.340	8.46E-03
328	CACUL1	Ralstonia insidiosa	-0.223	1.06E-04	1061	HOOK1	Porphyromonadaceae	0.410	7.97E-03	1794	PPP2R1A	Ralstonia insidiosa	0.254	1.93E-05	2527	TOMM20	Debaryomycetaceae	0.362	7.01E-03
329	CALCRL	Bacillales	0.449	1.58E-03	1062	HOOK1	Ralstonia insidiosa	-0.272	4.93E-04	1795	PPP2R5E	Microbacteriaceae	-0.352	8.74E-03	2528	TOMM20	Ralstonia insidiosa	-0.264	1.47E-04
330	CALCRL	Colletotrichum lupini	-0.256	8.31E-03	1063	HOOK2	Ralstonia insidiosa	0.279	1.02E-04	1796	PPP2R5E	Ralstonia insidiosa	-0.247	7.48E-09	2529	TONSL	Ralstonia insidiosa	0.302	1.66E-07
331	CALCRL	Saccharomycetales	0.427	2.30E-03	1064	HOOK3	Phyllobacteriaceae	0.219	2.41E-03	1797	PPP4R2	Ralstonia insidiosa	-0.264	8.56E-11	2530	TOP2B	Porphyromonadaceae	0.379	6.08E-04
332	CALM2	Malassezia vespertilionis	0.344	2.24E-04	1065	HOXB2	Pseudomonas oleovorans	-0.250	4.03E-03	1798	PPP4R3A	Ralstonia insidiosa	-0.223	5.33E-03	2531	TOPORS	Brevundimonas sp.	0.439	1.12E-04
333	CALML6	Alphaproteobacteria	-0.356	8.06E-03	1066	HRAS	Porphyromonadaceae	-0.427	6.32E-04	1799	PPP4R3B	Ralstonia insidiosa	-0.280	3.37E-12	2532	TOPORS	Ralstonia insidiosa	-0.140	1.91E-06
334	CALU	Brevibacteriaceae	0.001	5.73E-03	1067	HS2ST1	Ralstonia insidiosa	-0.319	1.70E-13	1800	PPP4R4	Malasseziales	0.387	2.92E-04	2533	TOX2	Ralstonia insidiosa	0.248	5.00E-04
335	CALU	Colletotrichum lupini	-0.239	1.00E-02	1068	HSD17B11	Ralstonia insidiosa	-0.248	2.41E-03	1801	PPP6C	Ralstonia insidiosa	-0.360	2.04E-12	2534	TP53I11	Ralstonia insidiosa	0.157	3.45E-07
336	CAMK2B	Ralstonia insidiosa	0.274	8.55E-06	1069	HSD17B13	Ralstonia insidiosa	-0.299	1.97E-03	1802	PPP6C	Saccharomycetales	0.415	1.35E-04	2535	TP53I13	Bacillales	-0.453	6.10E-03
337	CAMK2D	Ralstonia insidiosa	-0.209	1.67E-11	1070	HSF1	Ralstonia insidiosa	0.229	9.21E-10	1803	PPP6R1	Ralstonia insidiosa	0.190	4.85E-07	2536	TP53INP1	Malasseziales	0.238	2.35E-03
338	CAMK2D	Saccharomycetales	0.404	1.80E-04	1071	HSP90AA1	Enterobacteriaceae	-0.299	2.46E-03	1804	PPP6R3	Microbacteriaceae	-0.270	8.22E-03	2537	TP53INP2	Caulobacteraceae	0.280	1.55E-03
339	CAMSAP2	Ralstonia insidiosa	-0.140	2.66E-08	1072	HSPA14	Ralstonia insidiosa	-0.246	3.63E-03	1805	PPP6R3	Ralstonia insidiosa	-0.103	1.84E-09	2538	TPD52	Anoxybacillus	-0.140	6.70E-03
340	CAMSAP3	Bacillales	-0.471	3.92E-05	1073	HSPB1	Ralstonia insidiosa	0.225	3.51E-04	1806	PRCC	Bacillales	-0.464	2.60E-03	2539	TPD52	Malassezia restricta	0.350	1.47E-03
341	CAMSAP3	Ralstonia insidiosa	0.259	5.65E-11	1074	HSPBP1	Bacillales	-0.332	8.79E-03	1807	PRCC	Ralstonia insidiosa	0.204	5.04E-05	2540	TPD52	Moraxellaceae	-0.235	9.56E-03
342	CAMTA2	Enterobacteriaceae	0.264	5.67E-05	1075	HSPBP1	Ralstonia insidiosa	0.314	5.77E-04	1808	PRDM1	Dolosigranulum	0.275	6.73E-03	2541	TPMT	Ralstonia insidiosa	-0.327	3.21E-07
343	CAND1	Porphyromonadaceae	0.531	2.70E-04	1076	HUS1	Acinetobacter ursingii	0.313	3.69E-03	1809	PRDX3	Debaryomycetaceae	0.342	1.42E-03	2542	TPP2	Sphaerotilaceae	-0.338	3.18E-04
344	CAND1	Ralstonia insidiosa	-0.239	1.91E-08	1077	HUWE1	Cloacibacterium normanense	0.232	5.98E-03	1810	PRELID3B	Malassezia restricta	0.268	8.34E-03	2543	TPR	Actinomyces naeslundii	0.365	2.24E-03
345	CAPN15	Ralstonia insidiosa	0.264	1.48E-05	1078	ICE1	Ralstonia insidiosa	-0.227	2.73E-04	1811	PRELID3B	Moraxellaceae	-0.340	4.05E-04	2544	TPR	Porphyromonadaceae	0.298	5.23E-04
346	CAPN7	Ralstonia insidiosa	-0.248	8.56E-11	1079	ICMT	Ascomycota	0.308	9.20E-03	1812	PRELP	Ralstonia insidiosa	0.291	5.59E-03	2545	TPRG1	Acidovorax sp. KKS102	-0.185	1.77E-07
347	CAPS2	Malassezia vespertilionis	0.275	3.51E-03	1080	ID2	Ralstonia insidiosa	-0.222	1.65E-03	1813	PREX2	Cloacibacterium normanense	0.310	6.28E-03	2546	TPT1	Debaryomycetaceae	0.247	8.79E-03
348	CAPS2	Ralstonia insidiosa	-0.222	1.90E-03	1081	IDE	Porphyromonadaceae	0.502	1.70E-03	1814	PRG4	Caulobacteraceae	0.198	4.25E-03	2547	TRABD	Bacillales	-0.284	4.81E-03
349	CAPZA1	Ralstonia insidiosa	-0.300	7.36E-09	1082	IER2	Bacillales	-0.448	2.76E-03	1815	PRICKLE3	Ralstonia insidiosa	0.297	3.16E-06	2548	TRABD	Ralstonia insidiosa	0.216	1.07E-03
350	CAPZA2	Ralstonia insidiosa	-0.283	1.91E-06	1083	IFI44L	Veillonellaceae	0.260	4.08E-03	1816	PRIM1	Methyloversatilis sp. RAC08	0.345	6.46E-03	2549	TRAF3IP2	Prevotella melaninogenica	0.219	6.27E-04
351	CARF	Ralstonia insidiosa	-0.256	4.05E-04	1084	IFIT5	Ralstonia insidiosa	-0.185	4.94E-04	1817	PRIM2	Nevskiales	0.343	4.10E-03	2550	TRAF6	Ralstonia insidiosa	-0.288	2.13E-03
352	CARM1	Ralstonia insidiosa	0.117	2.41E-03	1085	IFNGR1	Bacillales	0.452	1.71E-05	1818	PRKAA1	Malasseziales	0.269	1.22E-04	2551	TRAF7	Alphaproteobacteria	0.347	2.14E-03
353	CARNMT1	Ralstonia insidiosa	-0.262	2.21E-07	1086	IFT46	Pasteurellaceae	0.160	7.15E-03	1819	PRKAB2	Ralstonia insidiosa	-0.264	7.61E-03	2552	TRAK2	Bacillales	0.542	1.38E-04
354	CASD1	Ralstonia insidiosa	-0.294	1.56E-08	1087	IFT74	Ralstonia insidiosa	-0.200	1.75E-06	1820	PRKACB	Ralstonia insidiosa	-0.299	3.19E-06	2553	TRAK2	Ralstonia insidiosa	-0.234	3.86E-03
355	CASKIN2	Ralstonia insidiosa	0.224	2.37E-08	1088	IFT88	Ralstonia insidiosa	-0.212	2.78E-04	1821	PRKAR1A	Saccharomycetales	0.473	5.35E-03	2554	TRAM1	Ralstonia insidiosa	-0.329	1.27E-08
356	CAVIN3	Porphyromonadaceae	-0.260	5.37E-03	1089	IGFLR1	Ralstonia insidiosa	0.200	1.92E-03	1822	PRKAR2A	Delftia acidovorans	0.427	4.43E-03	2555	TRAPPC1	Ralstonia insidiosa	0.191	1.02E-03
357	CBFA2T3	Bacillales	-0.447	6.49E-03	1090	IGSF8	Bacillales	-0.378	4.16E-04	1823	PRKCSH	Ralstonia insidiosa	0.187	2.43E-04	2556	TRAPPC11	Porphyromonadaceae	0.437	3.90E-03
358	CBLL1	Ralstonia insidiosa	-0.293	2.44E-03	1091	IKBIP	Dermacoccaceae	0.292	9.06E-03	1824	PRKCZ	Betaproteobacteria	0.290	1.74E-04	2557	TRAPPC13	Ralstonia insidiosa	-0.230	5.52E-03
359	CBR4	Enterobacteriaceae	-0.259	1.08E-03	1092	IL27	Bacillales	-0.511	9.17E-04	1825	PRKCZ	Malasseziales	-0.296	8.22E-03	2558	TRAPPC6B	Ralstonia insidiosa	-0.281	1.39E-10
360	CBR4	Ralstonia insidiosa	-0.259	1.01E-05	1093	IL6ST	Ralstonia insidiosa	-0.248	2.13E-07	1826	PROS1	Ralstonia insidiosa	-0.279	2.97E-06	2559	TRAPPC8	Ralstonia insidiosa	-0.264	1.55E-11
361	CBX3	Alcaligenaceae	0.309	1.28E-03	1094	IMMP1L	Leptotrichiaceae	0.149	3.10E-03	1827	PRPF3	Alphaproteobacteria	-0.444	2.78E-03	2560	TRAPPC9	Ralstonia insidiosa	0.199	4.41E-03
362	CBX3	Ralstonia insidiosa	-0.268	8.12E-08	1095	IMPACT	Ralstonia insidiosa	-0.324	8.06E-13	1828	PRPF39	Ralstonia insidiosa	-0.248	1.75E-05	2561	TRIAP1	Leptotrichiaceae	0.158	7.12E-03
363	CBX6	Staphylococcus saprophyticus	-0.236	5.30E-03	1096	INF2	Debaryomycetaceae	-0.387	2.78E-03	1829	PRPF40A	Enterobacteriaceae	-0.259	3.20E-05	2562	TRIM13	Betaproteobacteria	-0.281	4.81E-03
364	CC2D1A	Bacillales	-0.421	3.46E-04	1097	INF2	Ralstonia insidiosa	0.246	1.32E-08	1830	PRPS1	Acidovorax sp. KKS102	0.265	2.35E-06	2563	TRIM13	Ralstonia insidiosa	-0.216	3.63E-05
365	CC2D1A	Ralstonia insidiosa	0.180	5.39E-10	1098	INO80E	Bacillales	-0.495	3.48E-03	1831	PRPSAP1	Bacilli	-0.254	8.66E-03	2564	TRIM23	Porphyromonadaceae	0.485	1.27E-03
366	CCAR1	Phyllobacteriaceae	0.338	7.07E-04	1099	INPP5B	Streptococcus oralis	0.024	2.62E-03	1832	PRR12	Debaryomycetaceae	-0.397	1.35E-06	2565	TRIM26	Enterobacteriaceae	0.246	1.95E-03
367	CCAR2	Acinetobacter ursingii	-0.435	2.11E-03	1100	INPP5E	Bacillales	-0.505	7.01E-03	1833	PRR12	Ralstonia insidiosa	0.225	2.72E-07	2566	TRIM28	Ralstonia insidiosa	0.215	1.92E-07
368	CCAR2	Ralstonia insidiosa	0.229	3.42E-04	1101	INPP5E	Prevotella melaninogenica	-0.446	4.67E-04	1834	PRR14	Porphyromonadaceae	-0.504	4.59E-03	2567	TRIM32	Pseudomonas oleovorans	0.298	2.66E-03
369	CCDC107	Cloacibacterium normanense	-0.118	6.66E-03	1102	INSIG2	Ralstonia insidiosa	-0.180	1.28E-05	1835	PRRC1	Ralstonia insidiosa	-0.319	8.05E-05	2568	TRIM33	Delftia acidovorans	0.359	1.84E-03
370	CCDC107	Ralstonia insidiosa	0.278	3.83E-04	1103	INTS1	Ralstonia insidiosa	0.206	8.15E-11	1836	PRRC2A	Enterobacteriaceae	0.289	2.53E-03	2569	TRIM56	Debaryomycetaceae	-0.394	8.79E-04
371	CCDC112	Cloacibacterium normanense	-0.012	7.13E-03	1104	INTS1	Sclerotiniaceae	-0.339	6.67E-03	1837	PRRC2B	Enterobacteriaceae	0.286	4.40E-03	2570	TRIOBP	Ralstonia insidiosa	0.228	3.11E-06
372	CCDC117	Ralstonia insidiosa	-0.270	1.32E-04	1105	INTS8	Ralstonia insidiosa	-0.272	6.29E-07	1838	PRRC2C	Delftia acidovorans	0.391	6.68E-07	2571	TRIP12	Porphyromonadaceae	0.498	2.60E-05
373	CCDC121	Ralstonia insidiosa	-0.241	4.81E-07	1106	IPMK	Moraxellaceae	-0.356	9.52E-03	1839	PRRC2C	Phyllobacteriaceae	0.407	5.30E-05	2572	TRIP12	Ralstonia insidiosa	-0.180	6.51E-05
374	CCDC124	Ralstonia insidiosa	0.280	1.10E-04	1107	IPO5	Ralstonia insidiosa	-0.203	4.78E-03	1840	PRRG1	Ralstonia insidiosa	-0.248	1.45E-03	2573	TRIQK	Ralstonia insidiosa	-0.290	1.42E-08
375	CCDC125	Alphaproteobacteria	-0.386	7.89E-04	1108	IPO7	Ralstonia insidiosa	-0.271	8.53E-09	1841	PRRG4	Malasseziales	0.327	1.53E-04	2574	TRMT10A	Ralstonia insidiosa	-0.221	1.08E-03

376	CCDC126	Ralstonia insidiosa	-0.248	2.55E-06	1109	IPO8	Porphyromonadaceae	0.468	3.33E-07	1842	PRSS50	Geodermatophilaceae	-0.465	1.44E-03	2575	TRMT13	Betaproteobacteria	-0.392	7.85E-03
377	CCDC13	Acidovorax sp. KKS102	-0.176	3.63E-03	1110	IPO8	Ralstonia insidiosa	-0.257	1.02E-10	1843	PSKH1	Brevibacteriaceae	-0.141	1.56E-03	2576	TRMT1L	Microbacteriaceae	-0.347	6.14E-03
378	CCDC14	Ralstonia insidiosa	-0.226	3.61E-03	1111	IQCB1	Alphaproteobacteria	-0.387	2.45E-04	1844	PSMA1	Ralstonia insidiosa	-0.257	1.46E-03	2577	TRMT1L	Ralstonia insidiosa	-0.275	4.41E-11
379	CCDC24	Saccharomycetales	-0.328	4.95E-03	1112	IQCN	Epilithonimonas	-0.435	4.51E-06	1845	PSMA1	Sclerotiniaceae	0.305	5.09E-05	2578	TRMT61A	Bacillales	-0.401	9.02E-04
380	CCDC30	Acidovorax sp. KKS102	-0.189	7.58E-03	1113	IREB2	Porphyromonadaceae	0.513	6.27E-03	1846	PSMA4	Leptotrichiaceae	0.183	6.84E-06	2579	TRO	Alphaproteobacteria	-0.366	9.16E-03
381	CCDC30	Streptococcus oralis	0.118	1.20E-03	1114	IRF2BP1	Ralstonia insidiosa	0.294	3.08E-06	1847	PSMA4	Malassezia vespertilionis	0.292	4.01E-03	2580	TRPV4	Debaryomycetaceae	-0.244	1.10E-04
382	CCDC34	Staphylococcus saprophyticus	-0.190	1.07E-03	1115	IRF2BPL	Bacillales	-0.346	1.38E-03	1848	PSMB9	Plantactinospora sp. BB1	0.257	3.66E-03	2581	TSC1	Streptococcus oralis	0.194	6.12E-03
383	CCDC39	Malasseziales	0.261	5.58E-03	1116	IRF2BPL	Malassezia vespertilionis	-0.214	6.15E-03	1849	PSMC1	Brevibacteriaceae	-0.117	1.35E-04	2582	TSC22D2	Corynebacterium accolens	0.234	6.02E-03
384	CCDC50	Ralstonia insidiosa	-0.266	3.23E-06	1117	ISCU	Leptotrichiaceae	0.164	2.13E-03	1850	PSMC1	Streptococcus salivarius	-0.108	8.46E-03	2583	TSG101	Brevibacteriaceae	-0.036	5.87E-05
385	CCDC57	Bacillales	-0.456	9.98E-03	1118	ISOC1	Ralstonia insidiosa	-0.238	6.03E-06	1851	PSMC6	Ralstonia insidiosa	-0.237	1.55E-06	2584	TSNAXIP1	Streptococcus oralis	-0.093	1.15E-03
386	CCDC57	Herbaspirillum huttiense	-0.323	7.87E-05	1119	ISYNA1	Ralstonia insidiosa	0.247	6.00E-04	1852	PSMD12	Ralstonia insidiosa	-0.212	3.99E-07	2585	TSPAN31	Acidovorax sp. KKS102	0.217	4.56E-04
387	CCDC59	Cloacibacterium normanense	-0.149	6.31E-06	1120	ITCH	Porphyromonadaceae	0.448	6.65E-03	1853	PSME3IP1	Alphaproteobacteria	-0.392	1.23E-03	2586	TSPAN6	Debaryomycetaceae	0.389	1.28E-04
388	CCDC82	Ralstonia insidiosa	-0.227	3.10E-06	1121	ITFG1	Acidovorax sp. KKS102	0.411	6.05E-03	1854	PSMG1	Ralstonia insidiosa	-0.255	8.97E-03	2587	TSPAN6	Ralstonia insidiosa	-0.306	1.45E-06
389	CCDC85B	Ascomycota	-0.414	4.04E-03	1122	ITFG1	Ralstonia insidiosa	-0.285	1.92E-09	1855	PTAR1	Porphyromonadaceae	0.438	1.78E-05	2588	TSPYL4	Porphyromonadaceae	0.360	4.87E-03
390	CCDC9	Ralstonia insidiosa	0.246	3.89E-05	1123	ITGA6	Ralstonia insidiosa	-0.218	4.06E-03	1856	PTAR1	Ralstonia insidiosa	-0.227	5.47E-10	2589	TSTD2	Porphyromonadaceae	0.459	7.65E-03
391	CCDC90B	Ralstonia insidiosa	-0.305	8.81E-08	1124	ITGAV	Ralstonia insidiosa	-0.229	4.62E-04	1857	PTBP2	Betaproteobacteria	-0.373	2.80E-04	2590	TTC21A	Viruses	-0.322	6.01E-03
392	CCDC92	Ralstonia insidiosa	0.321	1.41E-05	1125	ITM2B	Malasseziales	0.369	8.06E-13	1858	PTBP2	Ralstonia insidiosa	-0.215	8.75E-04	2591	TTC32	Betaproteobacteria	-0.302	2.01E-03
393	CCDC97	Ralstonia insidiosa	0.167	1.40E-03	1126	ITM2B	Ralstonia insidiosa	-0.266	1.04E-05	1859	PTBP3	Ralstonia insidiosa	-0.266	3.97E-06	2592	TTC32	Ralstonia insidiosa	-0.222	9.01E-05
394	CCNC	Ralstonia insidiosa	-0.299	7.45E-07	1127	ITPR3	Cutibacterium	0.257	3.18E-03	1860	PTBP3	Saccharomycetales	0.427	3.03E-07	2593	TTC33	Ralstonia insidiosa	-0.290	1.55E-05
395	CCNG1	Ralstonia insidiosa	-0.326	1.55E-11	1128	ITPRID2	Ralstonia insidiosa	-0.218	3.34E-05	1861	PTCD2	Ralstonia insidiosa	-0.248	4.08E-06	2594	TTF1	Cloacibacterium normanense	-0.027	3.62E-03
396	CCNG2	Ralstonia insidiosa	-0.208	7.38E-03	1129	JADE3	Ralstonia insidiosa	-0.230	3.75E-04	1862	PTCD3	Ralstonia insidiosa	-0.144	1.35E-05	2595	TTYH3	Debaryomycetaceae	-0.191	9.83E-04
397	CCNT1	Ralstonia insidiosa	-0.216	1.32E-06	1130	JARID2	Phyllobacteriaceae	0.213	5.18E-05	1863	PTEN	Caulobacteraceae	-0.336	2.13E-04	2596	TUBGCP2	Ralstonia insidiosa	0.217	7.18E-04
398	CCNT2	Betaproteobacteria	-0.286	1.10E-04	1131	JDP2	Fusobacteriia	0.205	7.98E-03	1864	PTEN	Microbacteriaceae	-0.345	4.88E-03	2597	TUG1	Ralstonia insidiosa	-0.287	1.56E-04
399	CCNT2	Ralstonia insidiosa	-0.197	1.31E-06	1132	JDP2	Ralstonia insidiosa	0.194	4.22E-03	1865	PTER	Ralstonia insidiosa	-0.284	6.97E-07	2598	TUT7	Ralstonia insidiosa	-0.133	7.75E-03
400	CCPG1	Ralstonia insidiosa	-0.291	1.79E-04	1133	JKAMP	Ralstonia insidiosa	-0.267	5.55E-06	1866	PTPN13	Nocardioides	-0.208	8.06E-03	2599	TVP23A	Acidovorax sp. KKS102	-0.306	1.53E-05
401	CCR10	Cloacibacterium normanense	-0.059	5.98E-03	1134	JMJD8	Acidobacteriota	-0.249	3.42E-03	1867	PTPN4	Ralstonia insidiosa	-0.219	9.16E-04	2600	TVP23B	Ralstonia insidiosa	-0.294	2.33E-03
402	CD164	Ralstonia insidiosa	-0.269	2.06E-08	1135	JMJD8	Alphaproteobacteria	0.259	2.64E-03	1868	PTPRJ	Cloacibacterium normanense	0.265	1.75E-03	2601	TWSG1	Ralstonia insidiosa	-0.196	6.25E-05
403	CD248	Ralstonia insidiosa	0.268	5.35E-03	1136	JOSD2	Bacillales	-0.354	4.66E-03	1869	PTTG1IP2	Staphylococcus saccharolyticus	-0.081	9.85E-03	2602	TXNDC16	Ralstonia insidiosa	-0.229	3.40E-04
404	CD46	Ralstonia insidiosa	-0.256	1.54E-11	1137	KANK3	Ralstonia insidiosa	0.334	6.49E-09	1870	PUM2	Ralstonia insidiosa	-0.206	2.36E-10	2603	TXNDC5	Thermaceae	0.257	8.96E-05
405	CD47	Ralstonia insidiosa	-0.287	2.84E-08	1138	KARS1	Corynebacterium accolens	-0.033	2.84E-05	1871	PURA	Delftia acidovorans	0.432	2.82E-03	2604	TXNL1	Sclerotiniaceae	0.328	3.00E-04
406	CD63	Cloacibacterium normanense	-0.357	7.56E-03	1139	KAT2B	Ralstonia insidiosa	-0.289	1.66E-06	1872	PURA	Enterobacteriaceae	-0.207	4.64E-03	2605	TXNRD2	Bacillales	-0.446	2.91E-05
407	CD7	Ralstonia insidiosa	0.252	7.06E-04	1140	KAT7	Cloacibacterium normanense	0.289	5.69E-03	1873	PUS10	Ralstonia insidiosa	-0.207	1.36E-03	2606	TXNRD2	Ralstonia insidiosa	0.276	5.58E-03
408	CDC14A	Ralstonia insidiosa	-0.189	5.29E-03	1141	KATNAL1	Ralstonia insidiosa	-0.188	2.52E-03	1874	PUS7L	Ralstonia insidiosa	-0.232	3.13E-05	2607	TXNRD3	Brevibacteriaceae	-0.089	1.49E-03
409	CDC27	Ralstonia insidiosa	-0.299	6.39E-11	1142	KATNAL2	Acidovorax sp. KKS102	-0.110	1.09E-07	1875	PVRIG	Brevundimonas sp.	-0.263	9.51E-05	2608	U2SURP	Betaproteobacteria	-0.259	5.36E-03
410	CDC37L1	Caulobacterales	-0.324	4.27E-03	1143	KATNBL1	Ralstonia insidiosa	-0.260	9.27E-05	1876	PXMP2	Actinomyces naeslundii	-0.334	3.84E-03	2609	UACA	Actinomyces naeslundii	0.190	4.37E-03
411	CDC40	Geodermatophilaceae	0.543	8.29E-03	1144	KBTBD3	Ralstonia insidiosa	-0.265	5.82E-05	1877	PYCR2	Ralstonia insidiosa	0.241	7.12E-04	2610	UACA	Betaproteobacteria	-0.273	5.48E-04
412	CDC42BPA	Nostocaceae	0.229	1.47E-03	1145	KBTBD7	Enterobacteriaceae	-0.255	9.28E-04	1878	QKI	Saccharomycetales	0.381	1.80E-03	2611	UBA3	Ralstonia insidiosa	-0.244	5.25E-08
413	CDC42EP1	Ralstonia insidiosa	0.198	1.82E-05	1146	KBTBD8	Ralstonia insidiosa	-0.212	6.66E-04	1879	QTRT1	Bacillales	-0.474	9.38E-03	2612	UBA6	Caldimonas	-0.263	5.57E-03
414	CDC42EP2	Ralstonia insidiosa	0.214	1.26E-03	1147	KCNQ1	Ralstonia insidiosa	0.178	5.35E-03	1880	RAB11FIP3	Ralstonia insidiosa	0.239	1.74E-04	2613	UBA6	Ralstonia insidiosa	-0.239	3.23E-06
415	CDC42SE2	Caulobacteraceae	-0.341	3.09E-03	1148	KCTD13	Ralstonia insidiosa	0.261	9.60E-05	1881	RAB14	Saccharomycetales	0.333	7.18E-04	2614	UBA7	Acidobacteriota	-0.193	2.77E-03
416	CDC42SE2	Ralstonia insidiosa	-0.276	1.22E-05	1149	KCTD7	Brachybacterium	-0.258	8.58E-03	1882	RAB18	Ralstonia insidiosa	-0.276	1.79E-08	2615	UBALD1	Ralstonia insidiosa	0.261	5.09E-09
417	CDCA7	Cloacibacterium caeni	0.133	9.16E-03	1150	KCTD9	Ralstonia insidiosa	-0.279	5.45E-08	1883	RAB1B	Betaproteobacteria	0.279	1.79E-03	2616	UBAP2	Actinomyces naeslundii	0.153	2.98E-03
418	CDH12	Rhodoferax	0.213	5.64E-03	1151	KCTD9	Staphylococcus saprophyticus	0.347	4.45E-04	1884	RAB22A	Ralstonia insidiosa	-0.322	1.43E-05	2617	UBE2D1	Moraxellaceae	-0.346	3.95E-03
419	CDH26	Alphaproteobacteria	-0.314	1.65E-03	1152	KDM4B	Bacillales	-0.343	1.23E-04	1885	RAB28	Ralstonia insidiosa	-0.263	2.33E-03	2618	UBE2D3	Ralstonia insidiosa	-0.321	1.33E-08
420	CDH3	Lachnospiraceae	0.277	1.28E-04	1153	KDM5A	Phyllobacteriaceae	0.507	5.54E-04	1886	RAB2A	Ralstonia insidiosa	-0.326	8.51E-07	2619	UBE2E3	Actinomyces naeslundii	-0.138	1.61E-03
421	CDK11B	Ralstonia insidiosa	0.339	3.83E-05	1154	KHDRBS2	Gordonia bronchialis	0.216	6.68E-03	1887	RAB2B	Malasseziales	0.322	3.18E-03	2620	UBE2G1	Saccharomycetales	0.391	2.36E-05
422	CDK2AP1	Alcaligenaceae	0.319	6.01E-05	1155	KHSRP	Malassezia vespertilionis	-0.281	3.96E-03	1888	RAB33B	Delftia acidovorans	0.291	2.07E-03	2621	UBE2K	Malasseziales	0.337	8.75E-07
423	CDKL1	Cellvibrionales	-0.249	3.27E-04	1156	KHSRP	Ralstonia insidiosa	0.234	2.70E-08	1889	RAB33B	Enterobacteriaceae	-0.277	7.06E-03	2622	UBE2N	Malassezia vespertilionis	0.275	1.82E-05
424	CDO1	Ralstonia insidiosa	-0.228	3.39E-03	1157	KIAA0825	Corynebacterium accolens	0.226	9.46E-03	1890	RAB3IL1	Ralstonia insidiosa	0.237	6.74E-05	2623	UBE2V2	Ralstonia insidiosa	-0.254	2.58E-10
425	CDYL2	Porphyromonadaceae	0.256	5.10E-03	1158	KIAA0930	Malasseziales	-0.278	1.88E-04	1891	RAB40C	Bacillales	-0.323	6.65E-03	2624	UBE3D	Streptococcus thermophilus	-0.014	2.01E-03
426	CEACAM19	Streptococcus oralis	0.009	2.57E-03	1159	KIAA1143	Ralstonia insidiosa	-0.233	3.15E-03	1892	RAB7A	Corynebacterium accolens	0.015	2.19E-03	2625	UBE4A	Microbacteriaceae	-0.328	2.60E-03
427	CEACAM8	Verrucomicrobiota	-0.172	3.22E-04	1160	KIF18A	Cutibacterium avidum	0.172	6.59E-03	1893	RAB8B	Caulobacteraceae	-0.383	5.59E-03	2626	UBE4A	Ralstonia insidiosa	-0.252	1.06E-13
428	CEBPZOS	Betaproteobacteria	-0.301	5.72E-03	1161	KIF1C	Ralstonia insidiosa	0.100	2.13E-03	1894	RAB8B	Colletotrichum lupini	-0.205	9.40E-04	2627	UBLCP1	Enterobacteriaceae	-0.250	1.65E-04
429	CELSR3	Epilithonimonas	-0.242	7.72E-04	1162	KIF20B	Enterobacteriaceae	-0.282	3.44E-04	1895	RAB8B	Saccharomycetales	0.415	1.44E-04	2628	UBLCP1	Ralstonia insidiosa	-0.218	8.16E-09

430	CENPB	Ralstonia insidiosa	0.249	8.90E-04	1163	KIF21A	Delftia acidovorans	0.426	6.34E-07	1896	RABEP2	Ralstonia insidiosa	0.280	1.33E-09	2629	UBR1	Porphyromonadaceae	0.503	5.28E-03
431	CENPC	Cloacibacterium normanense	0.303	2.63E-07	1164	KIF3A	Phyllobacteriaceae	0.368	1.97E-08	1897	RABGAP1L	Saccharomycetales	0.333	1.17E-03	2630	UBR2	Porphyromonadaceae	0.441	1.55E-03
432	CENPC	Porphyromonadaceae	0.271	3.81E-03	1165	KIF5B	Enterobacteriaceae	-0.238	2.74E-03	1898	RABGGTA	Lawsonellaceae	-0.380	3.38E-03	2631	UBR3	Ralstonia insidiosa	-0.231	3.39E-06
433	CENPC	Ralstonia insidiosa	-0.138	5.97E-04	1166	KIFC3	Ralstonia insidiosa	0.261	1.57E-08	1899	RABL6	Ralstonia insidiosa	0.267	2.66E-11	2632	UBR5	Porphyromonadaceae	0.390	1.32E-03
434	CEP120	Ralstonia insidiosa	-0.232	1.02E-04	1167	KLF12	Ralstonia insidiosa	-0.197	2.85E-05	1900	RAD17	Debaryomycetaceae	0.324	4.35E-03	2633	UBTD1	Ralstonia insidiosa	0.211	2.53E-03
435	CEP170	Ralstonia insidiosa	-0.135	3.55E-03	1168	KLF16	Caulobacteraceae	0.319	5.24E-03	1901	RAD17	Ralstonia insidiosa	-0.344	1.79E-12	2634	UBXN11	Ralstonia insidiosa	0.232	3.92E-03
436	CEP350	Ralstonia insidiosa	-0.150	3.77E-05	1169	KLHL15	Caulobacterales	-0.382	6.95E-03	1902	RAD18	Ralstonia insidiosa	-0.213	6.72E-03	2635	UBXN2B	Ralstonia insidiosa	-0.231	1.83E-08
437	CEP44	Betaproteobacteria	-0.304	2.74E-03	1170	KLHL15	Ralstonia insidiosa	-0.245	1.03E-03	1903	RAD23A	Enterobacteriaceae	0.246	9.24E-03	2636	UBXN2B	Saccharomycetales	0.337	3.97E-04
438	CEP57	Bacillales	0.368	1.63E-04	1171	KLHL24	Ralstonia insidiosa	-0.135	1.00E-03	1904	RAD23A	Ralstonia insidiosa	0.113	4.48E-05	2637	UBXN4	Enterobacteriaceae	-0.287	9.94E-05
439	CEP57	Ralstonia insidiosa	-0.237	1.18E-04	1172	KLHL34	Corynebacterium accolens	0.146	4.83E-05	1905	RAD23B	Ralstonia insidiosa	-0.310	8.01E-07	2638	UBXN6	Alphaproteobacteria	0.303	9.66E-04
440	CEP57L1	Betaproteobacteria	-0.316	1.44E-04	1173	KLHL9	Porphyromonadaceae	0.479	5.04E-03	1906	RAI1	Ralstonia insidiosa	0.236	3.86E-04	2639	UBXN8	Sphingobacteriaceae	0.371	7.85E-04
441	CEP70	Malassezia vespertilionis	0.292	2.86E-03	1174	KMT2B	Debaryomycetaceae	-0.368	5.82E-03	1907	RALA	Sphingobacteriales	0.383	5.51E-03	2640	UFL1	Enterobacteriaceae	-0.235	5.39E-04
442	CEP70	Ralstonia insidiosa	-0.265	4.98E-12	1175	KMT2B	Ralstonia insidiosa	0.248	2.83E-06	1908	RALBP1	Veillonella	-0.163	5.68E-04	2641	UFL1	Ralstonia insidiosa	-0.214	4.15E-05
443	CEP72	Epilithonimonas	-0.257	9.54E-04	1176	KMT2E	Delftia acidovorans	0.386	2.06E-04	1909	RALGAPA1	Ralstonia insidiosa	-0.169	2.37E-07	2642	UFM1	Ralstonia insidiosa	-0.343	8.10E-06
444	CEP85L	Enterobacteriaceae	-0.300	1.18E-04	1177	KMT5B	Ralstonia insidiosa	-0.219	2.43E-05	1910	RALGAPA2	Cloacibacterium normanense	0.424	3.51E-05	2643	UGCG	Ralstonia insidiosa	-0.220	2.93E-03
445	CEP95	Alphaproteobacteria	-0.461	4.62E-04	1178	KNL1	Ralstonia insidiosa	-0.258	8.05E-05	1911	RALGPS2	Ralstonia insidiosa	-0.280	1.22E-05	2644	UGGT2	Microbacteriaceae	-0.309	1.61E-03
446	CEPT1	Ralstonia insidiosa	-0.231	3.61E-08	1179	KNOP1	Cloacibacterium normanense	-0.217	1.82E-06	1912	RALY	Ralstonia insidiosa	0.273	2.06E-13	2645	UGGT2	Ralstonia insidiosa	-0.157	1.21E-07
447	CERCAM	Ralstonia insidiosa	0.283	5.50E-03	1180	KPNA3	Ralstonia insidiosa	-0.282	2.18E-06	1913	RANBP2	Ralstonia insidiosa	-0.197	3.41E-03	2646	UGT1A7	Corynebacterium accolens	0.120	3.61E-03
448	CERS6	Bacillales	0.558	4.40E-03	1181	KPNA6	Delftia acidovorans	0.495	3.23E-06	1914	RANBP3	Bacillales	-0.369	2.72E-03	2647	UGT3A1	Ascomycota	0.282	1.49E-03
449	CETN3	Betaproteobacteria	-0.345	5.21E-06	1182	KRAS	Ralstonia insidiosa	-0.250	8.62E-04	1915	RANBP3	Ralstonia insidiosa	0.298	5.97E-12	2648	UHMK1	Ralstonia insidiosa	-0.277	2.11E-07
450	CFAP298	Alphaproteobacteria	-0.501	1.10E-03	1183	KRI1	Ralstonia insidiosa	0.241	4.50E-03	1916	RANBP6	Ralstonia insidiosa	-0.298	1.33E-11	2649	UHRF2	Ralstonia insidiosa	-0.168	2.88E-06
451	CFAP410	Ralstonia insidiosa	0.289	9.69E-07	1184	KRI1	Saccharomycetales	-0.406	2.96E-03	1917	RANBP6	Sphaerotilaceae	-0.402	3.69E-04	2650	UNC119	Rhizoctonia solani	-0.322	4.39E-03
452	CFAP70	Acidovorax sp. KKS102	-0.216	9.71E-05	1185	KRR1	Caulobacteraceae	-0.389	1.31E-04	1918	RANGAP1	Saccharomycetales	-0.316	8.54E-03	2651	UNC5CL	Comamonadaceae	0.186	4.30E-03
453	CFAP70	Streptococcus oralis	0.120	5.93E-03	1186	KRR1	Enterobacteriaceae	-0.236	9.19E-05	1919	RAP1B	Bacillales	0.416	1.38E-03	2652	UNC5D	Herbaspirillum huttiense	-0.180	7.36E-05
454	CFH	Ralstonia insidiosa	-0.194	1.02E-03	1187	KRR1	Ralstonia insidiosa	-0.245	6.34E-07	1920	RAP1GDS1	Ralstonia insidiosa	-0.191	3.53E-03	2653	UNK	Ralstonia insidiosa	0.364	4.01E-09
455	CFI	Ascomycota	0.289	6.94E-03	1188	KRTAP5-6	Brevibacteriaceae	0.117	8.20E-03	1921	RAP2A	Ralstonia insidiosa	-0.307	4.63E-05	2654	UNK	Streptococcus oralis	0.022	3.07E-03
456	CFL1	Acidobacteriota	-0.204	2.33E-03	1189	KYAT1	Saccharomycetales	-0.368	9.87E-03	1922	RAP2A	Saccharomycetales	0.414	3.00E-04	2655	UPF2	Delftia acidovorans	0.447	9.77E-06
457	CFL2	Caulobacterales	-0.307	6.04E-03	1190	KYAT3	Debaryomycetaceae	0.327	3.44E-04	1923	RAP2C	Moraxellaceae	-0.381	6.66E-03	2656	UPF3A	Cloacibacterium normanense	-0.357	2.82E-03
458	CFL2	Ralstonia insidiosa	-0.300	1.38E-09	1191	KYAT3	Ralstonia insidiosa	-0.202	5.07E-05	1924	RAP2C	Ralstonia insidiosa	-0.251	2.53E-04	2657	UPF3B	Cloacibacterium normanense	-0.190	9.09E-03
459	CGGBP1	Caulobacteraceae	-0.304	8.91E-04	1192	L3MBTL1	Oscillospiraceae	-0.421	6.87E-03	1925	RARA	Ralstonia insidiosa	0.163	8.22E-05	2658	UPF3B	Phyllobacteriaceae	0.171	2.54E-06
460	CGGBP1	Ralstonia insidiosa	-0.255	1.51E-09	1193	LACC1	Ralstonia insidiosa	-0.263	9.64E-04	1926	RASA2	Ralstonia insidiosa	-0.159	8.06E-06	2659	UPP1	Ralstonia insidiosa	0.291	1.54E-03
461	CHADL	Bacillales	-0.397	1.60E-04	1194	LACTB	Ralstonia insidiosa	-0.215	2.18E-04	1927	RASIP1	Ralstonia insidiosa	0.232	8.07E-04	2660	UQCC3	Prevotella melaninogenica	-0.228	6.66E-04
462	CHAF1A	Ralstonia insidiosa	0.236	1.49E-03	1195	LACTB2	Sphingobacteriaceae	0.324	9.05E-04	1928	RASSF8	Bacillales	0.362	2.06E-03	2661	UQCRB	Leptotrichiaceae	0.120	4.14E-03
463	CHCHD4	Brevibacteriaceae	-0.275	5.50E-04	1196	LAMTOR3	Ralstonia insidiosa	-0.282	5.31E-09	1929	RASSF8	Ralstonia insidiosa	-0.220	2.20E-03	2662	UROC1	Betaproteobacteria	0.213	1.07E-03
464	CHD7	Delftia acidovorans	0.243	4.42E-06	1197	LAMTOR5	Actinomyces naeslundii	-0.291	3.37E-03	1930	RBBP8	Sphingobacteriaceae	0.321	8.48E-03	2663	USF3	Ralstonia insidiosa	-0.189	1.72E-03
465	CHD9	Delftia acidovorans	0.397	5.81E-03	1198	LANCL1	Ralstonia insidiosa	-0.324	5.38E-08	1931	RBBP9	Ralstonia insidiosa	-0.294	8.87E-04	2664	USO1	Bacillales	0.484	3.44E-04
466	CHERP	Ralstonia insidiosa	0.249	2.20E-05	1199	LAPTM4A	Ralstonia insidiosa	-0.285	3.01E-06	1932	RBCK1	Brevundimonas sp.	-0.333	5.04E-06	2665	USO1	Ralstonia insidiosa	-0.238	5.15E-03
467	CHM	Bacillales	0.536	9.98E-03	1200	LARP1B	Enterobacteriaceae	-0.296	2.86E-03	1933	RBL2	Cloacibacterium normanense	0.426	1.05E-03	2666	USP12	Ralstonia insidiosa	-0.270	7.95E-04
468	CHM	Ralstonia insidiosa	-0.217	1.11E-04	1201	LARP7	Cloacibacterium normanense	-0.090	1.33E-07	1934	RBL2	Porphyromonadaceae	0.362	3.94E-03	2667	USP15	Ralstonia insidiosa	-0.183	2.79E-08
469	CHMP2B	Ralstonia insidiosa	-0.321	1.22E-09	1202	LBR	Ralstonia insidiosa	-0.255	3.33E-05	1935	RBM12	Bacillales	0.389	9.61E-03	2668	USP25	Porphyromonadaceae	0.498	1.33E-04
470	CHMP6	Ralstonia insidiosa	0.275	6.80E-03	1203	LCLAT1	Ralstonia insidiosa	-0.226	3.42E-03	1936	RBM12B	Porphyromonadaceae	0.430	2.89E-08	2669	USP34	Ralstonia insidiosa	-0.202	1.33E-11
471	CHORDC1	Ralstonia insidiosa	-0.210	4.61E-03	1204	LCN2	Pucciniaceae	-0.295	3.21E-03	1937	RBM14	Epilithonimonas	-0.381	8.50E-03	2670	USP37	Porphyromonadaceae	0.427	8.57E-03
472	CHRAC1	Ralstonia insidiosa	-0.337	5.74E-04	1205	LCORL	Caulobacteraceae	-0.376	8.45E-03	1938	RBM15	Ralstonia insidiosa	-0.162	4.98E-04	2671	USP40	Brevibacteriaceae	0.212	4.62E-03
473	CHRNA10	Alphaproteobacteria	-0.410	2.40E-03	1206	LCORL	Microbacteriaceae	-0.374	6.47E-03	1939	RBM26	Betaproteobacteria	-0.393	6.56E-05	2672	USP47	Ralstonia insidiosa	-0.139	5.55E-08
474	CHRNA4	Sphingobacteriaceae	-0.432	3.18E-04	1207	LCORL	Ralstonia insidiosa	-0.195	8.39E-08	1940	RBM28	Alphaproteobacteria	-0.341	6.24E-03	2673	USP49	Corynebacterium accolens	0.098	2.68E-04
475	CHTOP	Alphaproteobacteria	-0.471	3.36E-04	1208	LEMD3	Microbacteriaceae	-0.368	3.75E-03	1941	RBM28	Epilithonimonas	-0.297	1.94E-03	2674	USP49	Sphingosinicellaceae	-0.119	2.55E-03
476	CIAO3	Ralstonia insidiosa	0.242	8.30E-03	1209	LEMD3	Ralstonia insidiosa	-0.239	1.50E-06	1942	RBM38	Ralstonia insidiosa	0.199	8.01E-05	2675	USP6NL	Ralstonia insidiosa	-0.221	6.46E-03
477	CIC	Debaryomycetaceae	-0.296	7.32E-03	1210	LENG9	Ascomycota	-0.364	3.34E-03	1943	RBM41	Phyllobacteriaceae	0.312	9.36E-05	2676	USP8	Enterobacteriaceae	-0.203	9.20E-03
478	CIC	Ralstonia insidiosa	0.213	3.31E-05	1211	LGR4	Ralstonia insidiosa	-0.251	1.13E-06	1944	RBM48	Brucella	-0.182	8.04E-04	2677	USP9X	Porphyromonadaceae	0.442	1.00E-02
479	CIDEB	Methylobacteriaceae	-0.221	4.45E-03	1212	LIG3	Porphyromonadaceae	0.195	4.48E-04	1945	RBMS1	Comamonadaceae	-0.381	3.26E-03	2678	USPL1	Ralstonia insidiosa	-0.178	4.76E-06
480	CIDEB	Nevskiales	-0.240	6.93E-03	1213	LIG4	Ralstonia insidiosa	-0.224	3.39E-05	1946	RBMX	Ralstonia insidiosa	-0.255	2.01E-05	2679	UTP25	Ascomycota	0.416	5.48E-03
481	CILK1	Ralstonia insidiosa	-0.239	7.96E-07	1214	LIMK1	Ralstonia insidiosa	0.167	8.37E-04	1947	RBMX2	Cloacibacterium normanense	-0.193	2.67E-03	2680	UTP25	Porphyromonadaceae	0.465	4.24E-03
482	CIR1	Cloacibacterium normanense	-0.182	2.25E-05	1215	LIMS1	Comamonadaceae	-0.320	6.08E-03	1948	RBMXL1	Ralstonia insidiosa	-0.327	7.66E-07	2681	VAC14	Ralstonia insidiosa	0.208	8.15E-04
483	CIZ1	Ralstonia insidiosa	0.216	1.90E-05	1216	LIN7A	Bacillales	0.381	2.45E-03	1949	RBPMS2	Ralstonia insidiosa	0.159	1.65E-03	2682	VAMP4	Caulobacteraceae	-0.384	2.31E-07

484	CKS2	Sphingobacteriaceae	0.160	1.23E-03	1217	LIN7A	Ralstonia insidiosa	-0.263	1.14E-07	1950	RC3H1	Betaproteobacteria	-0.226	2.05E-05	2683	VAMP4	Ralstonia insidiosa	-0.252	5.94E-12
485	CLASP2	Ralstonia insidiosa	-0.137	9.69E-05	1218	LIN7B	Bacillales	-0.393	3.62E-04	1951	RC3H1	Ralstonia insidiosa	-0.222	3.40E-05	2684	VARS1	Ralstonia insidiosa	0.258	5.53E-06
486	CLCN3	Porphyromonadaceae	0.471	9.16E-03	1219	LINGO1	Ralstonia insidiosa	0.325	3.62E-05	1952	RC3H2	Ralstonia insidiosa	-0.198	1.94E-06	2685	VASN	Bacillales	-0.403	6.59E-03
487	CLCN3	Ralstonia insidiosa	-0.275	1.45E-05	1220	LIPF	Thermales	0.369	3.63E-07	1953	RCBTB1	Ralstonia insidiosa	-0.232	1.38E-03	2686	VASP	Ralstonia insidiosa	0.252	1.89E-03
488	CLCN7	Ralstonia insidiosa	0.221	4.66E-11	1221	LMAN1	Ralstonia insidiosa	-0.301	4.01E-09	1954	RCBTB2	Ralstonia insidiosa	-0.212	2.97E-03	2687	VEGFB	Ralstonia insidiosa	0.193	1.78E-04
489	CLDN11	Veillonellaceae	0.220	5.68E-03	1222	LMBRD1	Ralstonia insidiosa	-0.273	5.45E-08	1955	RCCD1	Alphaproteobacteria	-0.332	8.18E-05	2688	VHL	Debaryomycetaceae	-0.301	1.46E-03
490	CLDN12	Ralstonia insidiosa	-0.308	5.07E-13	1223	LMF2	Bacillales	-0.369	7.95E-04	1956	RCN2	Ralstonia insidiosa	-0.285	2.72E-06	2689	VMA21	Ralstonia insidiosa	-0.341	2.48E-03
491	CLDN3	Ralstonia insidiosa	0.252	2.86E-07	1224	LMNA	Ralstonia insidiosa	0.278	5.78E-06	1957	RECQL4	Ralstonia insidiosa	0.267	9.50E-04	2690	VMO1	Ralstonia insidiosa	0.266	8.84E-03
492	CLEC16A	Epilithonimonas	-0.268	7.02E-04	1225	LMNTD2	Bacillales	-0.462	7.18E-03	1958	REEP5	Ralstonia insidiosa	-0.341	5.10E-05	2691	VPS13A	Ralstonia insidiosa	-0.197	3.51E-05
493	CLEC16A	Ralstonia insidiosa	0.241	4.38E-03	1226	LMO2	Streptococcus thermophilus	-0.285	7.80E-04	1959	RELCH	Ralstonia insidiosa	-0.170	5.79E-05	2692	VPS26B	Corynebacterium accolens	-0.225	3.36E-03
494	CLEC3B	Alphaproteobacteria	0.201	1.69E-03	1227	LMO4	Alcaligenaceae	0.421	7.16E-03	1960	RELL1	Alcaligenaceae	0.351	5.17E-04	2693	VPS35	Prevotella melaninogenica	0.453	6.10E-03
495	CLEC3B	Ralstonia insidiosa	0.246	9.47E-04	1228	LMOD3	Rhodoferax	0.218	7.58E-03	1961	REN	Herbaspirillum huttiense	-0.113	6.66E-05	2694	VPS36	Ralstonia insidiosa	-0.219	1.03E-06
496	CLEC4D	Micromonosporales	0.302	6.85E-03	1229	LMTK2	Cloacibacterium normanense	0.298	9.35E-03	1962	REPIN1	Bacillales	-0.382	3.89E-04	2695	VPS37A	Staphylococcaceae	-0.309	9.37E-03
497	CLIC4	Saccharomycetales	0.453	2.55E-05	1230	LNPEP	Porphyromonadaceae	0.391	1.70E-08	1963	REPIN1	Ralstonia insidiosa	0.214	2.41E-04	2696	VPS37B	Ralstonia insidiosa	0.248	2.86E-06
498	CLPTM1	Betaproteobacteria	0.373	5.06E-03	1231	LONP1	Ralstonia insidiosa	0.219	2.49E-04	1964	REPS2	Ralstonia insidiosa	-0.267	1.96E-03	2697	VPS41	Bacillales	0.492	7.48E-03
499	CLSPN	Phyllobacteriaceae	0.234	6.63E-03	1232	LONRF1	Ralstonia insidiosa	-0.253	6.30E-05	1965	REST	Enterobacteriaceae	-0.246	1.50E-03	2698	VPS45	Actinomyces naeslundii	0.142	1.30E-04
500	CLSTN1	Ralstonia insidiosa	0.245	6.73E-03	1233	LPAR4	Brucella	-0.010	5.12E-03	1966	REST	Phyllobacteriaceae	0.500	9.16E-03	2699	VPS4A	Caulobacteraceae	0.296	3.82E-03
501	CLSTN3	Enterobacteriaceae	0.246	1.97E-03	1234	LPAR6	Alcaligenaceae	0.380	1.46E-03	1967	REX1BD	Bacillales	-0.549	1.58E-04	2700	VPS4A	Enterobacteriaceae	0.319	1.62E-03
502	CLTC	Porphyromonadaceae	0.443	8.73E-03	1235	LPAR6	Caulobacteraceae	-0.280	1.91E-03	1968	REXO4	Malasseziales	-0.296	1.13E-03	2701	VPS50	Ralstonia insidiosa	-0.253	1.32E-06
503	CMPK1	Ralstonia insidiosa	-0.331	1.29E-07	1236	LPXN	Betaproteobacteria	-0.302	8.14E-03	1969	RFX1	Ralstonia insidiosa	0.281	2.00E-07	2702	VPS53	Ralstonia insidiosa	0.318	2.27E-03
504	CMTM6	Bacillales	0.359	6.30E-03	1237	LRBA	Porphyromonadaceae	0.493	3.55E-03	1970	RFX3	Betaproteobacteria	-0.249	5.40E-04	2703	VPS54	Ralstonia insidiosa	-0.195	1.07E-04
505	CMTM6	Ralstonia insidiosa	-0.391	6.53E-10	1238	LRFN3	Ralstonia insidiosa	0.239	9.70E-08	1971	RFX7	Betaproteobacteria	-0.244	2.63E-04	2704	VPS9D1	Bacillales	-0.560	2.73E-03
506	CMTR2	Microbacteriaceae	-0.335	2.03E-03	1239	LRRC14	Ralstonia insidiosa	0.231	3.19E-03	1972	RGPD3	Corynebacterium accolens	0.038	8.35E-07	2705	VRK3	Caulobacteraceae	0.370	1.39E-04
507	CMTR2	Ralstonia insidiosa	-0.302	8.60E-19	1240	LRRC2	Delftia acidovorans	0.377	1.29E-03	1973	RGPD5	Nocardioides	-0.234	1.93E-03	2706	VSTM2A	Pseudomonas oleovorans	-0.107	4.08E-05
508	CNBP	Debaryomycetaceae	0.299	2.00E-03	1241	LRRC40	Ralstonia insidiosa	-0.292	1.20E-12	1974	RGS1	Actinomycetales	-0.174	3.66E-05	2707	VTA1	Ralstonia insidiosa	-0.324	6.41E-11
509	CNBP	Ralstonia insidiosa	-0.259	2.39E-04	1242	LRRC45	Bacillales	-0.439	4.55E-05	1975	RGS12	Bacillales	-0.381	7.13E-03	2708	VWA2	Campylobacteraceae	0.119	5.09E-03
510	CNEP1R1	Ralstonia insidiosa	-0.285	7.69E-05	1243	LRRC4B	Ralstonia insidiosa	0.309	9.33E-06	1976	RGS19	Ralstonia insidiosa	0.180	6.19E-03	2709	VWC2	Gordonia bronchialis	0.157	7.32E-03
511	CNGB1	Pseudomonas oleovorans	0.052	7.18E-03	1244	LRRC8B	Bacillales	0.477	3.20E-03	1977	RGS22	Brucella	-0.072	2.45E-03	2710	WAC	Saccharomycetales	0.442	1.92E-03
512	CNGB3	Brucella	0.043	1.39E-03	1245	LRRC8B	Ralstonia insidiosa	-0.232	3.76E-03	1978	RGSL1	Staphylococcus saccharolyticus	0.150	1.69E-04	2711	WAPL	Porphyromonadaceae	0.476	7.11E-03
513	CNGB3	Nevskiales	0.208	4.93E-03	1246	LRRC8C	Bacillales	0.418	1.72E-03	1979	RHBDF2	Debaryomycetaceae	-0.299	9.65E-04	2712	WAS	Ralstonia insidiosa	0.188	1.56E-03
514	CNIH1	Ralstonia insidiosa	-0.332	1.77E-05	1247	LRRC8C	Saccharomycetales	0.368	9.18E-03	1980	RHBDL1	Brevibacteriaceae	0.029	4.26E-05	2713	WASHC2C	Methylocystaceae	0.227	2.37E-03
515	CNOT1	Porphyromonadaceae	0.467	2.04E-03	1248	LRRC8D	Ralstonia insidiosa	-0.273	1.45E-07	1981	RHOG	Ralstonia insidiosa	0.230	5.06E-05	2714	WASHC4	Ralstonia insidiosa	-0.212	5.16E-07
516	CNOT2	Enterobacteriaceae	-0.200	1.93E-03	1249	LRRCC1	Enterobacteriaceae	-0.249	1.65E-03	1982	RHOQ	Alcaligenaceae	0.325	5.13E-05	2715	WBP4	Cloacibacterium normanense	0.034	9.26E-08
517	CNOT2	Ralstonia insidiosa	-0.167	2.42E-07	1250	LRSAM1	Bacillales	-0.301	2.65E-03	1983	RHOT1	Ralstonia insidiosa	-0.296	2.34E-07	2716	WDFY1	Ralstonia insidiosa	-0.263	1.14E-06
518	CNOT3	Ralstonia insidiosa	0.226	4.83E-05	1251	LRSAM1	Ralstonia insidiosa	0.198	3.31E-04	1984	RICTOR	Microbacteriaceae	-0.374	1.67E-04	2717	WDR11	Porphyromonadaceae	0.452	3.75E-09
519	CNOT6	Ralstonia insidiosa	-0.178	4.32E-04	1252	LRWD1	Bacillales	-0.243	1.24E-03	1985	RICTOR	Ralstonia insidiosa	-0.194	1.19E-12	2718	WDR18	Bacillales	-0.313	6.20E-04
520	CNPPD1	Malasseziales	-0.251	7.67E-04	1253	LSM8	Betaproteobacteria	-0.357	7.25E-03	1986	RIDA	Ralstonia insidiosa	-0.244	3.11E-03	2719	WDR18	Ralstonia insidiosa	0.284	9.21E-04
521	CNTNAP1	Sphingobacteriaceae	-0.411	1.71E-03	1254	LSM8	Caulobacteraceae	-0.255	3.48E-03	1987	RIF1	Ralstonia insidiosa	-0.206	3.05E-08	2720	WDR24	Bacillales	-0.326	6.35E-05
522	CNTROB	Enterobacteriaceae	0.276	1.50E-07	1255	LSM8	Ralstonia insidiosa	-0.228	2.40E-04	1988	RILPL1	Acidovorax sp. KKS102	-0.212	5.76E-13	2721	WDR24	Malassezia vespertilionis	-0.331	8.11E-05
523	COA5	Leptotrichiaceae	0.161	3.81E-05	1256	LTBP4	Ralstonia insidiosa	0.308	1.22E-06	1989	RILPL1	Ralstonia insidiosa	0.280	1.93E-11	2722	WDR26	Betaproteobacteria	-0.275	1.11E-09
524	COASY	Bacillales	-0.457	3.95E-04	1257	LTBR	Ralstonia insidiosa	0.136	2.80E-03	1990	RILPL1	Streptococcus oralis	0.044	9.50E-06	2723	WDR26	Ralstonia insidiosa	-0.259	1.06E-05
525	COBLL1	Ralstonia insidiosa	-0.236	5.51E-03	1258	LTC4S	Ralstonia insidiosa	0.294	3.64E-05	1991	RIMOC1	Ralstonia insidiosa	-0.263	1.65E-05	2724	WDR26	Sphaerotilaceae	-0.349	7.06E-03
526	COG4	Comamonadaceae	0.266	4.76E-03	1259	LTN1	Porphyromonadaceae	0.525	4.94E-04	1992	RIN3	Ralstonia insidiosa	0.194	9.60E-05	2725	WDR36	Ralstonia insidiosa	-0.228	3.33E-08
527	COG5	Porphyromonadaceae	0.503	7.91E-03	1260	LTN1	Ralstonia insidiosa	-0.226	4.89E-06	1993	RIPK1	Enterobacteriaceae	0.323	2.66E-03	2726	WDR44	Ralstonia insidiosa	-0.275	3.76E-05
528	COG6	Ralstonia insidiosa	-0.209	5.85E-05	1261	LUC7L2	Corynebacterium accolens	0.083	7.02E-03	1994	RIPOR1	Ralstonia insidiosa	0.183	1.04E-09	2727	WDR62	Dermacoccaceae	-0.359	8.16E-04
529	COL18A1	Ralstonia insidiosa	0.204	2.21E-07	1262	LUC7L3	Phyllobacteriaceae	0.063	3.55E-04	1995	RLF	Bacillales	0.458	2.41E-03	2728	WDR72	Ralstonia insidiosa	-0.265	5.54E-04
530	COL1A1	Ralstonia insidiosa	0.232	1.87E-03	1263	LY6G5B	Alphaproteobacteria	-0.474	1.56E-04	1996	RLF	Ralstonia insidiosa	-0.168	2.17E-05	2729	WDR74	Herbaspirillum huttiense	-0.291	2.22E-04
531	COL2A1	Brevibacteriaceae	0.231	4.08E-03	1264	LY6G5B	Oscillospiraceae	-0.475	8.27E-03	1997	RLIM	Porphyromonadaceae	0.487	5.57E-03	2730	WDR75	Ralstonia insidiosa	-0.160	7.41E-06
532	COL6A1	Ralstonia insidiosa	0.282	2.41E-08	1265	LYG1	Alphaproteobacteria	-0.428	5.03E-06	1998	RLIM	Ralstonia insidiosa	-0.207	6.90E-03	2731	WDR83	Geodermatophilaceae	-0.433	2.96E-03
533	COL6A2	Ralstonia insidiosa	0.309	2.29E-08	1266	LYG2	Lactococcus	0.259	4.81E-03	1999	RMDN2	Ralstonia insidiosa	-0.287	5.15E-09	2732	WDR86	Acidovorax sp. KKS102	-0.293	1.07E-04
534	COLQ	Alphaproteobacteria	-0.439	3.27E-03	1267	LYPLA1	Comamonadaceae	-0.311	5.09E-03	2000	RMI1	Caulobacteraceae	-0.424	8.86E-03	2733	WDR87	Brucella	-0.254	3.92E-05
535	COMMD4	Porphyromonadaceae	-0.372	1.06E-03	1268	LYPLAL1	Betaproteobacteria	-0.353	2.29E-07	2001	RNASE4	Ralstonia insidiosa	-0.296	6.38E-07	2734	WDR89	Ralstonia insidiosa	-0.323	9.50E-05
536	COMMD5	Bacillales	-0.354	9.97E-03	1269	LYPLAL1	Malassezia vespertilionis	0.405	1.06E-03	2002	RNASEH2B	Enterobacteriaceae	-0.270	6.56E-03	2735	WDR90	Bacillales	-0.450	3.07E-03
537	COP1	Malassezia vespertilionis	0.295	3.84E-03	1270	LYRM4	Nostocaceae	-0.251	2.80E-03	2003	RND3	Caulobacterales	-0.291	2.49E-03	2736	WDSUB1	Ralstonia insidiosa	-0.122	1.19E-03

538	COP1	Ralstonia insidiosa	-0.180	1.05E-04	1271	LYRM9	Brevundimonas sp.	-0.359	6.35E-03	2004	RNF11	Comamonadaceae	-0.324	1.37E-03	2737	WFIKKN1	Brevibacteriaceae	0.133	2.79E-05
539	COPB1	Bacillales	0.536	5.74E-03	1272	LYSMD3	Ralstonia insidiosa	-0.256	8.34E-07	2005	RNF114	Nostocaceae	-0.268	8.55E-03	2738	WTAP	Betaproteobacteria	-0.350	1.06E-03
540	CORIN	Acidovorax sp. KKS102	-0.131	3.56E-06	1273	LYST	Ralstonia insidiosa	-0.221	2.14E-05	2006	RNF126	Bacillales	-0.343	1.89E-04	2739	WWC2	Porphyromonadaceae	0.490	1.41E-04
541	CORO1B	Alphaproteobacteria	0.322	4.15E-06	1274	LZTFL1	Bacillales	0.371	2.12E-03	2007	RNF126	Ralstonia insidiosa	0.225	7.07E-09	2740	WWOX	Brevibacteriaceae	-0.088	5.98E-04
542	CORO1B	Ralstonia insidiosa	0.191	1.28E-04	1275	LZTFL1	Ralstonia insidiosa	-0.241	8.47E-04	2008	RNF128	Ralstonia insidiosa	-0.240	2.73E-04	2741	WWP1	Ralstonia insidiosa	-0.306	1.10E-07
543	COX11	Caulobacteraceae	-0.261	9.06E-04	1276	MACF1	Actinomyces naeslundii	0.266	1.62E-08	2009	RNF13	Bacillales	0.416	4.91E-03	2742	WWP2	Enterobacteriaceae	0.269	5.14E-03
544	COX11	Malassezia restricta	0.347	1.69E-03	1277	MACROD1	Bacillales	-0.438	3.29E-04	2010	RNF13	Ralstonia insidiosa	-0.242	6.70E-10	2743	WWTR1	Ralstonia insidiosa	-0.200	6.10E-03
545	COX20	Ralstonia insidiosa	-0.269	4.82E-03	1278	MAF	Caulobacteraceae	-0.264	6.40E-03	2011	RNF138	Moraxellaceae	-0.353	2.90E-03	2744	XAB2	Ralstonia insidiosa	0.197	4.05E-06
546	COX7A2	Actinomyces naeslundii	-0.295	5.16E-03	1279	MAF	Saccharomycetales	0.249	7.60E-04	2012	RNF138	Ralstonia insidiosa	-0.246	3.01E-06	2745	XPA	Bacilli	-0.364	3.78E-03
547	CPA3	Bacillales	0.352	5.14E-03	1280	MAFB	Methylorubrum	0.245	3.79E-03	2013	RNF139	Microbacteriaceae	-0.314	2.76E-03	2746	XPO1	Ralstonia insidiosa	-0.262	4.05E-07
548	CPB2	Epilithonimonas	0.301	7.58E-03	1281	MAFG	Delftia acidovorans	0.161	5.37E-04	2014	RNF139	Ralstonia insidiosa	-0.362	4.76E-18	2747	XPR1	Bacillales	0.570	3.69E-03
549	CPD	Porphyromonadaceae	0.417	9.60E-04	1282	MAGED1	Brevibacteriaceae	-0.163	3.22E-03	2015	RNF141	Ralstonia insidiosa	-0.316	4.02E-05	2748	XRCC3	Ralstonia insidiosa	0.217	1.50E-04
550	CPD	Ralstonia insidiosa	-0.213	4.63E-05	1283	MAGEH1	Brevibacteriaceae	-0.111	5.41E-03	2016	RNF144B	Ralstonia insidiosa	-0.198	5.71E-03	2749	XRCC4	Ralstonia insidiosa	-0.216	7.75E-03
551	CPEB2	Ralstonia insidiosa	-0.224	1.48E-05	1284	MAGI3	Porphyromonadaceae	0.389	1.47E-04	2017	RNF19A	Microbacteriaceae	-0.413	3.15E-03	2750	XRCC6	Acidovorax sp. KKS102	0.211	9.32E-03
552	CPEB4	Caulobacterales	-0.353	2.79E-03	1285	MAGIX	Ralstonia insidiosa	0.261	1.44E-03	2018	RNF19A	Ralstonia insidiosa	-0.255	1.42E-08	2751	XRCC6	Brevibacteriaceae	-0.148	2.93E-06
553	CPNE3	Ralstonia insidiosa	-0.282	4.01E-07	1286	MAL2	Caulobacteraceae	-0.295	2.39E-04	2019	RNF2	Epilithonimonas	0.414	7.36E-03	2752	XRN1	Ralstonia insidiosa	-0.197	5.30E-05
554	CPNE7	Pseudomonas oleovorans	-0.301	8.27E-03	1287	MAL2	Microbacteriaceae	-0.357	8.98E-04	2020	RNF208	Bacillales	-0.425	2.54E-03	2753	XYLT2	Bacillales	-0.340	9.54E-04
555	CPNE8	Ralstonia insidiosa	-0.167	3.48E-05	1288	MAL2	Ralstonia insidiosa	-0.327	2.88E-20	2021	RNF208	Ralstonia insidiosa	0.331	9.99E-10	2754	YAE1	Malassezia vespertilionis	0.300	9.26E-03
556	CPSF1	Ralstonia insidiosa	0.201	4.90E-06	1289	MALSU1	Leptotrichiaceae	0.013	9.95E-03	2022	RNF214	Acidovorax sp. KKS102	-0.275	4.12E-05	2755	YAF2	Cloacibacterium normanense	-0.079	6.03E-03
557	CPSF7	Alphaproteobacteria	-0.406	1.32E-03	1290	MAN1A1	Ralstonia insidiosa	-0.278	3.32E-05	2023	RNF216	Ralstonia insidiosa	0.352	2.99E-03	2756	YAF2	Malassezia restricta	0.259	1.73E-03
558	CPTP	Bacillales	-0.347	5.83E-04	1291	MAN1A2	Enterobacteriaceae	-0.221	2.07E-04	2024	RNF217	Ralstonia insidiosa	-0.210	1.36E-04	2757	YARS1	Caulobacteraceae	0.317	4.40E-03
559	CPTP	Ralstonia insidiosa	0.214	1.18E-04	1292	MAN2A1	Ralstonia insidiosa	-0.272	7.81E-14	2025	RNF38	Ralstonia insidiosa	-0.250	2.41E-05	2758	YEATS4	Ralstonia insidiosa	-0.275	5.61E-06
560	CRAT	Betaproteobacteria	0.320	7.34E-04	1293	MAN2B2	Betaproteobacteria	0.332	6.63E-03	2026	RNGTT	Ralstonia insidiosa	-0.286	2.64E-06	2759	YIF1A	Ralstonia insidiosa	0.192	2.69E-03
561	CRBN	Ralstonia insidiosa	-0.251	1.41E-10	1294	MANEA	Ralstonia insidiosa	-0.271	2.73E-06	2027	RNH1	Ralstonia insidiosa	0.224	4.76E-03	2760	YIPF2	Alphaproteobacteria	0.349	1.42E-04
562	CREB1	Caulobacteraceae	-0.356	5.71E-04	1295	MAOB	Carnobacteriaceae	-0.166	1.02E-03	2028	RNPC3	Ralstonia insidiosa	-0.163	8.17E-03	2761	YIPF3	Acidobacteriota	-0.283	9.67E-04
563	CREBL2	Ralstonia insidiosa	-0.297	1.26E-05	1296	MAP10	Ralstonia insidiosa	-0.220	2.43E-05	2029	RNPEPL1	Bacillales	-0.384	8.90E-04	2762	YIPF5	Bacillales	0.466	7.15E-03
564	CREBRF	Ralstonia insidiosa	-0.130	3.71E-08	1297	MAP1S	Ralstonia insidiosa	0.238	6.77E-06	2030	RNPEPL1	Ralstonia insidiosa	0.230	4.05E-07	2763	YJU2	Ralstonia insidiosa	0.223	1.31E-03
565	CREG1	Ralstonia insidiosa	-0.268	3.62E-05	1298	MAP2K2	Ralstonia insidiosa	0.189	3.42E-07	2031	RNPS1	Ralstonia insidiosa	0.218	9.13E-04	2764	YJU2B	Bacillales	-0.472	3.56E-03
566	CRIP2	Ralstonia insidiosa	0.250	8.53E-06	1299	MAP2K2	Staphylococcus saprophyticus	-0.299	6.53E-03	2032	ROCK1	Enterobacteriaceae	-0.237	2.55E-03	2765	YOD1	Ralstonia insidiosa	-0.271	4.39E-05
567	CRIPT	Betaproteobacteria	-0.334	4.00E-03	1300	MAP3K11	Bacillales	-0.358	5.10E-03	2033	ROGDI	Geodermatophilaceae	-0.403	4.41E-03	2766	YPEL5	Debaryomycetaceae	0.396	5.24E-03
568	CRLS1	Ralstonia insidiosa	-0.314	8.86E-07	1301	MAP3K11	Ralstonia insidiosa	0.196	2.07E-05	2034	RPA2	Sphingobacteriaceae	0.114	9.67E-03	2767	YPEL5	Ralstonia insidiosa	-0.370	1.58E-11
569	CRTC1	Ralstonia insidiosa	0.239	9.41E-06	1302	MAP3K2	Ralstonia insidiosa	-0.229	6.14E-10	2035	RPL19	Corynebacterium accolens	-0.280	8.19E-03	2768	YTHDC2	Ralstonia insidiosa	-0.223	6.17E-06
570	CSF1	Ralstonia insidiosa	0.164	4.30E-03	1303	MAP3K7	Ralstonia insidiosa	-0.181	2.50E-03	2036	RPL28	Staphylococcus saprophyticus	-0.373	2.74E-03	2769	YTHDF3	Ralstonia insidiosa	-0.256	3.08E-06
571	CSK	Ralstonia insidiosa	0.226	9.97E-08	1304	MAP4K3	Ralstonia insidiosa	-0.253	3.12E-11	2037	RPL7A	Corynebacterium accolens	-0.343	9.48E-03	2770	YWHAB	Alcaligenaceae	0.257	9.78E-05
572	CSNK1D	Bacillales	-0.412	6.50E-03	1305	MAP7D1	Debaryomycetaceae	-0.321	5.27E-03	2038	RPRD1A	Bacillales	0.424	3.51E-03	2771	YWHAZ	Ralstonia insidiosa	-0.269	2.41E-04
573	CSNK1G2	Ralstonia insidiosa	0.224	5.19E-06	1306	MAP7D1	Ralstonia insidiosa	0.228	1.06E-05	2039	RPRD1A	Ralstonia insidiosa	-0.267	1.28E-04	2772	ZBBX	Gordonia bronchialis	0.221	6.15E-03
574	CSNK1G3	Microbacteriaceae	-0.386	8.67E-03	1307	MAPK1IP1L	Malasseziales	0.360	3.58E-03	2040	RPS27	Methyloversatilis sp. RAC08	0.169	4.12E-03	2773	ZBED5	Betaproteobacteria	-0.344	1.38E-03
575	CSNK2A2	Brevibacteriaceae	-0.258	4.94E-03	1308	MAPK6	Bacillales	0.473	1.78E-04	2041	RPS6KA4	Debaryomycetaceae	-0.281	5.99E-05	2774	ZBED5	Caulobacteraceae	-0.256	9.04E-04
576	CSPG4	Acidovorax sp. KKS102	-0.249	8.24E-03	1309	MAPK6	Ralstonia insidiosa	-0.273	1.92E-07	2042	RPS6KA4	Ralstonia insidiosa	0.245	2.89E-08	2775	ZBTB1	Ralstonia insidiosa	-0.204	2.59E-07
577	CSPP1	Delftia acidovorans	0.119	4.65E-05	1310	MAPK7	Debaryomycetaceae	-0.284	2.45E-03	2043	RPS6KB1	Ralstonia insidiosa	-0.252	1.92E-09	2776	ZBTB10	Ralstonia insidiosa	-0.177	1.61E-04
578	CSRNP3	Ralstonia insidiosa	-0.202	4.71E-03	1311	MAPK7	Ralstonia insidiosa	0.194	1.06E-04	2044	RPS6KC1	Ralstonia insidiosa	-0.183	3.48E-03	2777	ZBTB18	Ralstonia insidiosa	-0.185	4.15E-04
579	CSRP1	Enterobacteriaceae	0.251	4.82E-03	1312	MAPK8	Ralstonia insidiosa	-0.274	1.30E-06	2045	RPTOR	Ralstonia insidiosa	0.190	1.76E-03	2778	ZBTB20	Delftia acidovorans	0.369	1.58E-11
580	CSTF3	Leptotrichiaceae	0.154	7.09E-03	1313	MAPKAPK2	Acidobacteriota	-0.244	2.65E-03	2046	RPUSD1	Ralstonia insidiosa	0.290	1.22E-04	2779	ZBTB26	Malassezia vespertilionis	0.304	4.68E-03
581	CTBP1	Bacillales	-0.420	5.90E-03	1314	MARCHF1	Saccharomycetales	0.352	1.05E-03	2047	RRAS2	Ralstonia insidiosa	-0.264	8.68E-07	2780	ZBTB26	Microbacteriaceae	-0.393	7.18E-03
582	CTBP1	Brevibacteriaceae	0.025	1.00E-04	1315	MARCHF6	Ralstonia insidiosa	-0.162	3.18E-09	2048	RRBP1	Cutibacterium	0.215	4.20E-03	2781	ZBTB33	Ralstonia insidiosa	-0.254	4.49E-03
583	CTDSPL2	Caulobacteraceae	-0.381	5.18E-04	1316	MARCHF7	Ralstonia insidiosa	-0.270	7.75E-14	2049	RRM1	Acidovorax sp. KKS102	0.402	1.13E-03	2782	ZBTB41	Ralstonia insidiosa	-0.250	1.05E-09
584	CTIF	Epilithonimonas	-0.350	7.37E-03	1317	MARCKS	Alcaligenaceae	0.398	3.38E-05	2050	RRM2B	Ralstonia insidiosa	-0.269	4.04E-05	2783	ZBTB45	Ralstonia insidiosa	0.199	7.81E-04
585	CTNNAL1	Ralstonia insidiosa	-0.276	8.93E-03	1318	MARCO	Alphaproteobacteria	0.422	2.93E-03	2051	RSBN1L	Delftia acidovorans	0.235	2.25E-03	2784	ZBTB6	Ralstonia insidiosa	-0.256	1.25E-07
586	CTSO	Ralstonia insidiosa	-0.260	9.69E-07	1319	MARF1	Ralstonia insidiosa	-0.106	1.02E-04	2052	RSRC2	Ralstonia insidiosa	-0.158	4.88E-03	2785	ZBTB7A	Bacillales	-0.469	1.22E-04
587	CTU2	Bacillales	-0.323	9.98E-03	1320	MARK2	Ralstonia insidiosa	0.295	1.05E-03	2053	RTCB	Brevibacteriaceae	-0.125	8.24E-06	2786	ZBTB7A	Debaryomycetaceae	-0.445	3.77E-04
588	CUEDC1	Ralstonia insidiosa	0.199	7.45E-03	1321	MARK3	Alphaproteobacteria	-0.408	5.58E-03	2054	RTCB	Empedobacter falsenii	0.204	7.18E-03	2787	ZC3H13	Brevibacteriaceae	0.158	4.63E-03
589	CUL4B	Ralstonia insidiosa	-0.289	2.83E-12	1322	MARK4	Ralstonia insidiosa	0.233	2.15E-04	2055	RTCB	Streptococcus oralis	-0.140	8.89E-05	2788	ZC3H18	Ralstonia insidiosa	0.213	2.88E-06
590	CUX1	Ralstonia insidiosa	0.214	6.68E-07	1323	MATK	Ralstonia insidiosa	0.280	9.34E-05	2056	RTF1	Phyllobacteriaceae	0.349	6.80E-05	2789	ZC3H4	Ralstonia insidiosa	0.294	3.50E-05
591	CWC22	Ralstonia insidiosa	-0.196	7.67E-07	1324	MATR3	Ralstonia insidiosa	-0.243	3.12E-10	2057	RTF1	Staphylococcus saprophyticus	0.032	4.34E-03	2790	ZC3H8	Alphaproteobacteria	-0.376	3.88E-03

592	CWF19L2	Cloacibacterium normanense	0.105	4.01E-07	1325	MAZ	Alphaproteobacteria	0.253	1.26E-03	2058	RTL6	Brevibacteriaceae	-0.135	4.07E-04	2791	ZDBF2	Ralstonia insidiosa	-0.185	1.26E-05
593	CWF19L2	Sphingobium yanoikuyae	0.353	7.12E-08	1326	MAZ	Ralstonia insidiosa	0.133	1.04E-04	2059	RTN4	Ralstonia insidiosa	-0.256	2.19E-06	2792	ZDHHC17	Caulobacteraceae	-0.342	2.34E-03
594	CXADR	Comamonadaceae	-0.346	6.28E-03	1327	MBD6	Debaryomycetaceae	-0.396	2.05E-04	2060	RTN4RL1	Ralstonia insidiosa	0.178	4.46E-03	2793	ZDHHC17	Ralstonia insidiosa	-0.234	3.05E-09
595	CXCL9	Methylorubrum populi	0.240	5.57E-05	1328	MBNL1	Caulobacteraceae	-0.353	2.57E-03	2061	RTRAF	Leptotrichiaceae	0.101	6.02E-03	2794	ZDHHC21	Caulobacteraceae	-0.321	6.22E-03
596	CXXC4	Microbacteriaceae	-0.250	3.95E-03	1329	MBNL1	Microbacteriaceae	-0.408	5.70E-03	2062	RUBCN	Acidovorax sp. KKS102	-0.200	6.51E-03	2795	ZDHHC21	Ralstonia insidiosa	-0.175	1.10E-14
597	CXXC5	Bacillales	-0.501	1.54E-03	1330	MBNL1	Ralstonia insidiosa	-0.219	3.62E-09	2063	RUNDC1	Delftia acidovorans	0.258	1.20E-03	2796	ZEB1	Caulobacteraceae	-0.408	8.68E-03
598	CYBA	Ralstonia insidiosa	0.316	2.53E-07	1331	MBOAT7	Bacillales	-0.329	3.90E-03	2064	RWDD2A	Alphaproteobacteria	-0.301	5.20E-03	2797	ZFAND1	Debaryomycetaceae	0.322	3.11E-03
599	CYBC1	Ralstonia insidiosa	0.192	1.28E-04	1332	MBOAT7	Ralstonia insidiosa	0.226	2.83E-04	2065	RWDD3	Ralstonia insidiosa	-0.247	3.61E-04	2798	ZFAND1	Ralstonia insidiosa	-0.280	5.26E-10
600	CYBRD1	Bacillales	0.375	9.68E-03	1333	MBTD1	Ralstonia insidiosa	-0.194	6.96E-07	2066	RWDD4	Ralstonia insidiosa	-0.254	2.31E-04	2799	ZFAND6	Caulobacteraceae	-0.283	3.73E-03
601	CYLD	Ralstonia insidiosa	-0.188	3.12E-05	1334	MCL1	Caulobacterales	-0.311	4.34E-05	2067	RYBP	Ralstonia insidiosa	-0.274	9.76E-05	2800	ZFC3H1	Betaproteobacteria	-0.247	3.04E-03
602	CYP24A1	Paucibacter	0.250	3.59E-03	1335	MCTS1	Betaproteobacteria	-0.363	9.20E-03	2068	RYK	Ralstonia insidiosa	-0.247	1.97E-08	2801	ZFHX4	Delftia acidovorans	0.217	4.15E-07
603	CYP27C1	Blautia	0.261	4.66E-05	1336	MDM1	Ralstonia insidiosa	-0.131	8.80E-03	2069	SACM1L	Ralstonia insidiosa	-0.284	7.40E-11	2802	ZFP1	Ralstonia insidiosa	-0.226	2.52E-06
604	CYP2A7	Streptococcus thermophilus	0.105	3.61E-03	1337	MDM4	Alphaproteobacteria	-0.442	1.10E-07	2070	SAFB	Bacillales	-0.358	3.37E-03	2803	ZFP91	Saccharomycetales	0.505	2.27E-03
605	CYP2R1	Ralstonia insidiosa	-0.142	8.10E-03	1338	MDM4	Streptococcus oralis	0.026	3.63E-03	2071	SAFB2	Brevibacteriaceae	0.075	7.19E-11	2804	ZFPL1	Ralstonia insidiosa	0.242	4.58E-04
606	CYP39A1	Ralstonia insidiosa	-0.299	3.60E-05	1339	MEAK7	Blautia	0.211	2.00E-03	2072	SAMD1	Porphyromonadaceae	-0.500	3.20E-03	2805	ZFPM1	Bacillales	-0.338	5.93E-03
607	CYP46A1	Geodermatophilaceae	-0.403	8.30E-03	1340	MED10	Porphyromonadaceae	-0.345	2.37E-03	2073	SAMD14	Ralstonia insidiosa	0.259	2.06E-03	2806	ZFPM1	Debaryomycetaceae	-0.310	9.19E-03
608	CYP4V2	Porphyromonadaceae	0.425	2.84E-03	1341	MED14	Porphyromonadaceae	0.360	6.36E-03	2074	SAMD4B	Ralstonia insidiosa	0.236	7.46E-05	2807	ZFPM1	Ralstonia insidiosa	0.217	4.97E-05
609	CYSTM1	Thermaceae	-0.321	4.67E-04	1342	MED15	Ralstonia insidiosa	0.274	1.65E-07	2075	SAMD8	Porphyromonadaceae	0.478	2.92E-03	2808	ZFR	Porphyromonadaceae	0.547	4.39E-04
610	CYYR1	Bacillales	0.341	2.22E-03	1343	MED16	Ralstonia insidiosa	0.204	1.59E-06	2076	SAMD8	Ralstonia insidiosa	-0.186	6.54E-04	2809	ZFYVE16	Ralstonia insidiosa	-0.164	4.59E-04
611	CYYR1	Saccharomycetales	0.308	8.31E-03	1344	MED17	Ralstonia insidiosa	-0.242	2.41E-03	2077	SAR1B	Ralstonia insidiosa	-0.286	1.52E-03	2810	ZFYVE28	Bacillales	-0.313	9.60E-04
612	D2HGDH	Bacillales	-0.507	2.49E-03	1345	MED18	Debaryomycetaceae	-0.290	7.59E-03	2078	SART1	Bacillales	-0.437	6.36E-03	2811	ZHX1	Caulobacteraceae	-0.391	9.59E-03
613	DAAM1	Delftia acidovorans	0.250	8.11E-04	1346	MED21	Microbacteriaceae	-0.393	7.47E-05	2079	SASS6	Ralstonia insidiosa	-0.196	1.59E-04	2812	ZKSCAN2	Uroviricota	-0.177	3.75E-03
614	DACH1	Comamonadaceae	-0.301	6.32E-05	1347	MED21	Ralstonia insidiosa	-0.282	1.96E-15	2080	SAXO2	Lautropia mirabilis	0.168	4.77E-04	2813	ZKSCAN3	Prevotella melaninogenica	-0.072	2.25E-06
615	DAGLB	Haemophilus	0.069	8.59E-03	1348	MED23	Ralstonia insidiosa	-0.168	7.37E-07	2081	SBDS	Ralstonia insidiosa	-0.265	3.84E-04	2814	ZKSCAN8	Ralstonia insidiosa	-0.236	4.72E-05
616	DALRD3	Microbacteriaceae	0.264	3.43E-03	1349	MED24	Enterobacteriaceae	0.298	4.11E-03	2082	SBNO1	Delftia acidovorans	0.522	9.20E-14	2815	ZMYM2	Microbacteriaceae	-0.342	2.37E-03
617	DAO	Acidovorax sp. KKS102	0.209	6.23E-03	1350	MED25	Bacillales	-0.388	2.47E-03	2083	SBNO2	Ralstonia insidiosa	0.210	1.50E-06	2816	ZMYM2	Ralstonia insidiosa	-0.251	1.24E-09
618	DAZAP2	Ralstonia insidiosa	-0.369	1.53E-06	1351	MED25	Malassezia vespertilionis	-0.265	3.07E-03	2084	SCAF1	Bacillales	-0.374	3.37E-04	2817	ZMYM4	Bacillales	0.331	3.05E-03
619	DCAF17	Ralstonia insidiosa	-0.246	3.92E-05	1352	MED25	Ralstonia insidiosa	0.310	1.35E-06	2085	SCAF1	Ralstonia insidiosa	0.240	1.09E-10	2818	ZMYM4	Ralstonia insidiosa	-0.214	4.20E-04
620	DCBLD2	Ralstonia insidiosa	-0.152	1.35E-06	1353	MED4	Ralstonia insidiosa	-0.275	1.54E-07	2086	SCAF4	Ralstonia insidiosa	0.252	2.53E-03	2819	ZMYM5	Betaproteobacteria	-0.239	1.40E-03
621	DCLK2	Brucella	-0.297	7.56E-04	1354	MEFV	Cloacibacterium normanense	0.154	1.33E-03	2087	SCAF8	Porphyromonadaceae	0.311	1.21E-03	2820	ZMYM6	Ralstonia insidiosa	-0.215	5.11E-03
622	DCLRE1A	Ralstonia insidiosa	-0.271	6.50E-03	1355	MEIS1	Brucella	-0.223	1.44E-03	2088	SCAMP1	Comamonadaceae	-0.286	1.65E-03	2821	ZMYND11	Ralstonia insidiosa	-0.333	7.47E-11
623	DCLRE1C	Phyllobacteriaceae	0.291	4.07E-04	1356	MEPCE	Nevskiales	-0.400	9.06E-04	2089	SCAMP1	Ralstonia insidiosa	-0.247	1.20E-07	2822	ZNF10	Alphaproteobacteria	-0.367	6.65E-04
624	DCP1A	Delftia acidovorans	0.464	6.50E-07	1357	METTL14	Ralstonia insidiosa	-0.290	2.89E-08	2090	SCAMP2	Acidobacteriota	-0.221	3.28E-06	2823	ZNF112	Ralstonia insidiosa	-0.205	2.00E-03
625	DCP1A	Phyllobacteriaceae	0.498	9.31E-03	1358	METTL15	Enterobacteriaceae	-0.241	3.44E-04	2091	SCAMP3	Acidobacteriota	-0.211	4.74E-03	2824	ZNF12	Ralstonia insidiosa	-0.178	1.75E-04
626	DCP2	Ralstonia insidiosa	-0.261	6.64E-11	1359	METTL22	Saccharomycetales	-0.460	4.26E-03	2092	SCAMP4	Ralstonia insidiosa	0.152	1.22E-08	2825	ZNF131	Ralstonia insidiosa	-0.211	4.31E-04
627	DCTD	Corynebacterium accolens	-0.126	4.22E-03	1360	METTL4	Lawsonellaceae	0.310	6.81E-03	2093	SCAND1	Brevibacteriaceae	0.092	1.73E-05	2826	ZNF136	Ralstonia insidiosa	-0.182	2.74E-03
628	DCTN4	Bacillales	0.455	7.11E-03	1361	METTL4	Ralstonia insidiosa	-0.173	1.76E-03	2094	SCAP	Ralstonia insidiosa	0.224	7.46E-04	2827	ZNF141	Lawsonellaceae	0.256	9.25E-03
629	DCTN4	Ralstonia insidiosa	-0.291	2.18E-06	1362	METTL9	Acinetobacter ursingii	0.338	8.57E-03	2095	SCARF2	Ralstonia insidiosa	0.296	1.47E-05	2828	ZNF146	Ralstonia insidiosa	-0.332	7.66E-08
630	DCUN1D1	Caulobacteraceae	-0.375	4.41E-03	1363	MEX3C	Ralstonia insidiosa	-0.238	4.45E-05	2096	SCFD1	Bacillales	0.397	9.04E-03	2829	ZNF148	Caulobacteraceae	-0.390	3.55E-03
631	DCUN1D1	Microbacteriaceae	-0.357	2.64E-03	1364	MFAP1	Brevibacteriaceae	-0.156	6.55E-05	2097	SCML1	Ralstonia insidiosa	-0.182	1.35E-05	2830	ZNF154	Corynebacterium accolens	0.058	3.04E-03
632	DCUN1D2	Prevotella melaninogenica	-0.223	2.77E-04	1365	MFAP3	Bacillales	0.590	9.11E-04	2098	SCN1A	Pseudomonas oleovorans	0.027	1.85E-05	2831	ZNF180	Ralstonia insidiosa	-0.203	2.72E-05
633	DCUN1D2	Streptococcus thermophilus	0.009	1.16E-03	1366	MFAP3L	Ralstonia insidiosa	-0.279	1.51E-03	2099	SCOC	Ralstonia insidiosa	-0.321	1.55E-09	2832	ZNF181	Ralstonia insidiosa	-0.357	3.63E-13
634	DCUN1D4	Betaproteobacteria	-0.325	1.95E-05	1367	MFSD1	Ralstonia insidiosa	-0.316	4.98E-06	2100	SCRIB	Bacillales	-0.437	1.18E-07	2833	ZNF184	Ralstonia insidiosa	-0.188	6.04E-03
635	DDA1	Bacillales	-0.327	9.61E-04	1368	MFSD13A	Bacillales	-0.358	3.28E-03	2101	SCRIB	Ralstonia insidiosa	0.273	1.13E-08	2834	ZNF189	Ralstonia insidiosa	-0.194	3.90E-03
636	DDX49	Betaproteobacteria	0.306	5.85E-03	1369	MFSD14A	Ralstonia insidiosa	-0.269	2.35E-07	2102	SCRN3	Ralstonia insidiosa	-0.265	1.05E-04	2835	ZNF205	Ralstonia insidiosa	0.310	1.76E-06
637	DDX49	Ralstonia insidiosa	0.248	1.53E-04	1370	MFSD4B	Acidovorax sp. KKS102	-0.166	1.98E-11	2103	SCUBE3	Herbaspirillum huttiense	0.035	9.36E-05	2836	ZNF213	Ralstonia insidiosa	0.260	1.84E-09
638	DDX54	Ralstonia insidiosa	0.161	2.11E-03	1371	MFSD8	Ralstonia insidiosa	-0.303	4.63E-06	2104	SCX	Brevibacteriaceae	-0.012	2.93E-03	2837	ZNF219	Ralstonia insidiosa	0.298	3.31E-05
639	DDX6	Porphyromonadaceae	0.438	2.86E-03	1372	MGLL	Betaproteobacteria	0.352	9.97E-03	2105	SCYL2	Ralstonia insidiosa	-0.243	2.72E-08	2838	ZNF219	Sclerotiniaceae	-0.328	7.06E-03
640	DDX60L	Ralstonia insidiosa	-0.186	2.14E-03	1373	MGRN1	Bacillales	-0.312	7.80E-03	2106	SDCBP	Saccharomycetales	0.420	6.66E-05	2839	ZNF23	Alphaproteobacteria	-0.361	1.15E-03
641	DEK	Enterobacteriaceae	-0.260	6.99E-06	1374	MGRN1	Ralstonia insidiosa	0.277	5.69E-11	2107	SDE2	Porphyromonadaceae	0.453	3.61E-03	2840	ZNF24	Ralstonia insidiosa	-0.247	1.49E-08
642	DENND1A	Ralstonia insidiosa	0.269	4.76E-05	1375	MIA2	Ralstonia insidiosa	-0.242	2.45E-03	2108	SDHC	Actinomyces naeslundii	-0.010	1.57E-03	2841	ZNF248	Alphaproteobacteria	-0.345	2.45E-03
643	DENND1B	Ralstonia insidiosa	-0.270	7.99E-10	1376	MIA3	Porphyromonadaceae	0.353	8.08E-03	2109	SEC14L3	Phyllobacteriaceae	-0.280	5.35E-03	2842	ZNF260	Ralstonia insidiosa	-0.243	3.65E-04
644	DENND2A	Ralstonia insidiosa	0.231	2.50E-03	1377	MIB1	Ralstonia insidiosa	-0.248	2.04E-09	2110	SEC61A1	Moraxella	0.241	8.71E-03	2843	ZNF266	Alphaproteobacteria	-0.338	9.51E-03
645	DENND2C	Mycobacteriales	0.206	5.26E-03	1378	MIB2	Bacillales	-0.421	4.30E-03	2111	SEC61G	Malassezia vespertilionis	0.223	5.04E-03	2844	ZNF267	Ralstonia insidiosa	-0.267	7.15E-03

646	DENND6A	Malasseziales	0.295	8.46E-05	1379	MICALL1	Debaryomycetaceae	-0.371	4.97E-03	2112	SEC62	Enterobacteriaceae	-0.291	3.85E-03	2845	ZNF28	Ralstonia insidiosa	-0.233	4.24E-03
647	DENND6A	Ralstonia insidiosa	-0.242	1.90E-05	1380	MICALL1	Ralstonia insidiosa	0.266	5.92E-08	2113	SECISBP2L	Ralstonia insidiosa	-0.213	5.95E-04	2846	ZNF280D	Caulobacteraceae	-0.354	2.62E-03
648	DENND6B	Ascomycota	-0.334	7.19E-03	1381	MICU2	Ralstonia insidiosa	-0.306	8.00E-11	2114	SEL1L	Ralstonia insidiosa	-0.267	4.98E-06	2847	ZNF282	Bacillales	-0.313	6.93E-03
649	DEPDC7	Ralstonia insidiosa	-0.236	2.89E-08	1382	MICU3	Betaproteobacteria	-0.315	2.30E-04	2115	SELENOF	Ralstonia insidiosa	-0.299	7.60E-08	2848	ZNF283	Corynebacterium accolens	0.066	6.68E-04
650	DERL1	Ralstonia insidiosa	-0.292	4.82E-03	1383	MID2	Bacillales	0.490	4.62E-03	2116	SELENOT	Ralstonia insidiosa	-0.327	2.48E-09	2849	ZNF302	Ralstonia insidiosa	-0.171	1.91E-08
651	DESI1	Caulobacteraceae	0.424	1.11E-03	1384	MIDEAS	Epilithonimonas	-0.277	8.89E-03	2117	SEMA3F	Ralstonia insidiosa	0.214	1.56E-04	2850	ZNF319	Ralstonia insidiosa	0.245	4.74E-03
652	DESI2	Bacillales	0.415	9.04E-04	1385	MIER1	Enterobacteriaceae	-0.278	3.96E-06	2118	SEMA6C	Streptococcus oralis	-0.124	7.80E-04	2851	ZNF322	Ralstonia insidiosa	-0.233	4.72E-04
653	DESI2	Microbacteriaceae	-0.424	9.56E-03	1386	MIER2	Debaryomycetaceae	-0.316	3.29E-03	2119	SENP5	Corynebacterium accolens	0.205	6.02E-06	2852	ZNF326	Caulobacteraceae	-0.300	7.02E-03
654	DESI2	Ralstonia insidiosa	-0.295	1.62E-06	1387	MIER3	Ralstonia insidiosa	-0.301	1.86E-15	2120	SENP6	Ralstonia insidiosa	-0.248	4.98E-06	2853	ZNF334	Alphaproteobacteria	-0.382	1.47E-04
655	DEXI	Methylorubrum	0.174	3.85E-09	1388	MIF	Brucella	0.340	5.05E-07	2121	SENP7	Caulobacteraceae	-0.370	1.84E-03	2854	ZNF33B	Bacillales	0.306	6.16E-03
656	DEXI	Neisseria	0.194	9.43E-04	1389	MINDY3	Betaproteobacteria	-0.330	2.33E-03	2122	SENP7	Ralstonia insidiosa	-0.266	1.01E-07	2855	ZNF33B	Ralstonia insidiosa	-0.331	1.29E-08
657	DGCR8	Prevotella melaninogenica	-0.285	1.88E-03	1390	MINDY3	Ralstonia insidiosa	-0.231	3.29E-10	2123	SEPSECS	Microbacteriaceae	-0.371	3.65E-03	2856	ZNF345	Acidovorax sp. KKS102	-0.236	5.19E-04
658	DGKQ	Acinetobacter ursingii	-0.440	7.53E-03	1391	MINK1	Enterobacteriaceae	0.276	8.93E-03	2124	SEPSECS	Ralstonia insidiosa	-0.257	2.93E-07	2857	ZNF385A	Debaryomycetaceae	-0.233	2.39E-04
659	DGKQ	Bacillales	-0.382	4.49E-03	1392	MINPP1	Ralstonia insidiosa	-0.295	1.55E-08	2125	SEPTIN11	Geodermatophilaceae	0.490	5.97E-04	2858	ZNF398	Gordonia bronchialis	0.225	4.28E-03
660	DGKZ	Debaryomycetaceae	-0.257	4.10E-03	1393	MIOS	Ralstonia insidiosa	-0.234	1.80E-09	2126	SEPTIN7	Ralstonia insidiosa	-0.239	3.38E-04	2859	ZNF407	Cloacibacterium normanense	0.319	6.10E-07
661	DGKZ	Ralstonia insidiosa	0.190	5.41E-07	1394	MISP3	Bacillales	-0.401	4.50E-03	2127	SEPTIN9	Ralstonia insidiosa	0.143	8.86E-07	2860	ZNF408	Bacillales	-0.356	4.80E-04
662	DHRS1	Pasteurellaceae	-0.025	9.80E-03	1395	MKLN1	Ralstonia insidiosa	-0.157	5.95E-07	2128	SERINC1	Ralstonia insidiosa	-0.230	3.22E-06	2861	ZNF408	Ralstonia insidiosa	0.191	3.27E-04
663	DHTKD1	Delftia acidovorans	0.366	1.08E-03	1396	MLLT1	Ralstonia insidiosa	0.245	1.55E-08	2129	SERPINA12	Campylobacterota	-0.137	5.85E-03	2862	ZNF414	Ralstonia insidiosa	0.308	3.42E-06
664	DHX9	Porphyromonadaceae	0.437	9.54E-04	1397	MLXIP	Debaryomycetaceae	-0.391	1.30E-04	2130	SESN3	Saccharomycetales	0.419	3.26E-03	2863	ZNF414	Sclerotiniaceae	-0.395	8.15E-05
665	DICER1	Ralstonia insidiosa	-0.178	2.88E-03	1398	MMADHC	Caulobacterales	-0.288	9.24E-03	2131	SETD1A	Debaryomycetaceae	-0.357	2.15E-03	2864	ZNF420	Ralstonia insidiosa	-0.256	3.90E-05
666	DIO3	Rhizobiaceae	0.178	6.69E-03	1399	MMADHC	Debaryomycetaceae	0.415	9.15E-03	2132	SETD1A	Epilithonimonas	-0.402	5.93E-03	2865	ZNF433	Actinomyces naeslundii	-0.026	1.86E-05
667	DIPK1A	Ralstonia insidiosa	-0.323	5.50E-04	1400	MMGT1	Ralstonia insidiosa	-0.334	3.08E-03	2133	SETD1A	Ralstonia insidiosa	0.211	3.75E-06	2866	ZNF449	Ralstonia insidiosa	-0.271	1.85E-04
668	DIS3	Ralstonia insidiosa	-0.231	1.08E-08	1401	MMGT1	Saccharomycetales	0.402	5.44E-06	2134	SETD2	Actinomyces naeslundii	0.195	4.59E-04	2867	ZNF451	Ralstonia insidiosa	-0.072	1.13E-08
669	DISC1	Empedobacter falsenii	-0.230	5.56E-03	1402	MNT	Ralstonia insidiosa	0.195	5.82E-05	2135	SETD7	Porphyromonadaceae	0.502	1.75E-04	2868	ZNF461	Alphaproteobacteria	-0.270	9.75E-03
670	DLG1	Ralstonia insidiosa	-0.290	2.28E-06	1403	MOB1A	Bacillales	0.464	5.28E-03	2136	SETD9	Debaryomycetaceae	0.268	3.70E-03	2869	ZNF480	Bacillales	0.373	1.04E-03
671	DLGAP3	Pseudomonas oleovorans	-0.156	6.60E-03	1404	MOB1A	Ralstonia insidiosa	-0.288	1.88E-04	2137	SETX	Porphyromonadaceae	0.387	9.15E-03	2870	ZNF487	Alphaproteobacteria	-0.362	3.41E-03
672	DLGAP4	Ralstonia insidiosa	0.224	4.75E-07	1405	MOBP	Brucella	-0.132	3.49E-03	2138	SF3A1	Ralstonia insidiosa	0.186	2.91E-03	2871	ZNF507	Porphyromonadaceae	0.296	2.86E-03
673	DLST	Acidovorax sp. KKS102	0.308	2.14E-05	1406	MOCS1	Nitrobacteraceae	-0.246	1.02E-03	2139	SF3A2	Ralstonia insidiosa	0.220	1.46E-08	2872	ZNF510	Staphylococcus saprophyticus	0.286	7.15E-03
674	DMTN	Ralstonia insidiosa	0.141	7.06E-03	1407	MOGS	Caulobacteraceae	0.340	9.54E-03	2140	SF3B1	Ralstonia insidiosa	-0.212	5.43E-07	2873	ZNF512	Ralstonia insidiosa	-0.257	4.82E-03
675	DMXL1	Porphyromonadaceae	0.522	4.44E-03	1408	MON2	Ralstonia insidiosa	-0.189	7.22E-08	2141	SF3B2	Caulobacteraceae	0.319	6.32E-03	2874	ZNF518A	Ralstonia insidiosa	-0.181	4.37E-05
676	DMXL1	Ralstonia insidiosa	-0.205	2.01E-08	1409	MORC3	Ralstonia insidiosa	-0.158	2.23E-05	2142	SF3B4	Ralstonia insidiosa	0.180	2.96E-03	2875	ZNF524	Bacillales	-0.401	4.02E-03
677	DNA2	Alphaproteobacteria	-0.299	3.49E-03	1410	MORF4L1	Haemophilus parainfluenzae	-0.139	4.41E-03	2143	SFPQ	Alphaproteobacteria	-0.384	5.34E-03	2876	ZNF543	Cloacibacterium normanense	0.220	2.31E-03
678	DNAH9	Pseudomonas oleovorans	-0.230	1.63E-03	1411	MORF4L2	Ralstonia insidiosa	-0.307	3.96E-03	2144	SFT2D3	Nitrosomonadales	0.286	6.50E-04	2877	ZNF562	Corynebacterium accolens	0.300	1.63E-03
679	DNAI7	Corynebacterium durum	0.194	1.58E-03	1412	MORN4	Acidovorax sp. KKS102	-0.253	1.18E-03	2145	SGPP1	Moraxellaceae	-0.351	5.79E-04	2878	ZNF566	Enterobacteriaceae	-0.279	3.55E-03
680	DNAJB14	Ralstonia insidiosa	-0.224	2.22E-06	1413	MOSMO	Ralstonia insidiosa	-0.216	8.64E-08	2146	SGPP1	Ralstonia insidiosa	-0.295	6.17E-07	2879	ZNF569	Ralstonia insidiosa	-0.299	1.14E-07
681	DNAJB2	Cloacibacterium normanense	-0.294	2.83E-05	1414	MOSPD1	Ralstonia insidiosa	-0.259	5.86E-03	2147	SGSH	Epilithonimonas	-0.458	1.51E-03	2880	ZNF574	Ralstonia insidiosa	0.196	5.12E-03
682	DNAJB4	Ralstonia insidiosa	-0.243	3.19E-03	1415	MPC1	Micromonospora	0.288	4.51E-07	2148	SGTA	Ralstonia insidiosa	0.190	3.61E-03	2881	ZNF577	Alphaproteobacteria	-0.342	1.17E-03
683	DNAJC10	Ralstonia insidiosa	-0.298	1.77E-07	1416	MPG	Alphaproteobacteria	0.171	6.02E-03	2149	SH3BGRL	Caulobacteraceae	-0.361	4.95E-03	2882	ZNF579	Ascomycota	-0.430	3.03E-03
684	DNAJC2	Cloacibacterium normanense	-0.126	2.45E-05	1417	MPHOSPH8	Phyllobacteriaceae	0.377	6.81E-06	2150	SH3BP1	Ralstonia insidiosa	0.237	2.56E-05	2883	ZNF596	Alphaproteobacteria	-0.398	2.55E-03
685	DNAJC24	Ralstonia insidiosa	-0.395	2.16E-08	1418	MREG	Alphaproteobacteria	-0.551	4.43E-08	2151	SH3BP4	Lactobacillus iners	0.015	4.67E-06	2884	ZNF598	Bacillales	-0.459	5.61E-04
686	DNAJC8	Corynebacterium accolens	-0.178	5.83E-03	1419	MRNIP	Brachybacterium	-0.327	1.44E-03	2152	SH3BP5	Alphaproteobacteria	0.325	5.85E-03	2885	ZNF608	Brucella	-0.176	2.10E-03
687	DNMBP	Cloacibacterium normanense	0.067	6.46E-03	1420	MROH1	Ralstonia insidiosa	0.293	7.62E-07	2153	SH3BP5L	Dermacoccaceae	-0.347	6.08E-03	2886	ZNF608	Nocardioides	-0.175	3.36E-04
688	DNMT1	Debaryomycetaceae	-0.272	5.57E-03	1421	MRPL12	Bacillales	-0.449	4.20E-03	2154	SH3GL1	Bacillales	-0.359	5.19E-04	2887	ZNF626	Malasseziales	0.298	2.78E-03
689	DNMT1	Ralstonia insidiosa	0.257	5.20E-05	1422	MRPL18	Porphyromonadaceae	-0.245	5.82E-04	2155	SH3GL1	Ralstonia insidiosa	0.168	4.07E-05	2888	ZNF628	Bacillales	-0.393	3.52E-05
690	DOCK3	Nectriaceae	-0.427	8.75E-03	1423	MRPL39	Sclerotiniaceae	0.331	4.73E-03	2156	SH3GLB1	Bacillales	0.437	2.52E-03	2889	ZNF628	Ralstonia insidiosa	0.308	2.31E-06
691	DOCK5	Sphingosinicellaceae	-0.205	5.55E-04	1424	MRPL4	Ralstonia insidiosa	0.242	5.12E-03	2157	SHF	Bacillales	-0.406	6.33E-03	2890	ZNF628	Sclerotiniaceae	-0.368	5.09E-03
692	DOCK6	Ralstonia insidiosa	0.259	2.54E-05	1425	MRPL42	Ralstonia insidiosa	-0.279	2.13E-09	2158	SHKBP1	Betaproteobacteria	0.245	2.72E-03	2891	ZNF638	Actinomyces naeslundii	0.247	4.34E-03
693	DOHH	Ralstonia insidiosa	0.275	4.37E-03	1426	MRPL49	Epilithonimonas	0.210	9.88E-03	2159	SHKBP1	Ralstonia insidiosa	0.194	3.50E-05	2892	ZNF638	Ralstonia insidiosa	-0.088	1.01E-04
694	DOLK	Shinella	0.228	1.67E-03	1427	MRPL50	Ralstonia insidiosa	-0.240	3.38E-03	2160	SHOC2	Bacillales	0.474	4.94E-03	2893	ZNF639	Ralstonia insidiosa	-0.275	1.05E-04
695	DONSON	Alphaproteobacteria	-0.280	8.40E-04	1428	MRPS10	Epilithonimonas	0.405	1.75E-06	2161	SHPRH	Corynebacterium accolens	0.422	4.63E-04	2894	ZNF641	Actinomyces naeslundii	0.130	7.32E-04
696	DOP1A	Ralstonia insidiosa	-0.189	1.19E-03	1429	MRPS18A	Epilithonimonas	0.191	9.15E-04	2162	SHQ1	Nevskiales	0.295	5.40E-03	2895	ZNF644	Bacillales	0.450	2.11E-03
697	DPM1	Ralstonia insidiosa	-0.239	1.35E-03	1430	MRRF	Alphaproteobacteria	-0.283	1.44E-03	2163	SHROOM1	Ralstonia insidiosa	0.227	5.89E-05	2896	ZNF644	Ralstonia insidiosa	-0.204	2.63E-10
698	DPM2	Acidobacteriota	-0.257	1.24E-05	1431	MRTFA	Debaryomycetaceae	-0.366	1.42E-03	2164	SIGLEC7	Alphaproteobacteria	0.377	5.00E-03	2897	ZNF654	Ralstonia insidiosa	-0.224	3.74E-10
699	DPM3	Bacillales	-0.436	2.76E-03	1432	MRTFA	Ralstonia insidiosa	0.241	2.54E-08	2165	SIKE1	Ralstonia insidiosa	-0.252	1.64E-06	2898	ZNF668	Ralstonia insidiosa	0.315	8.16E-05

700	DPP3	Caulobacteraceae	0.324	3.05E-03	1433	MS4A4E	Brucella	-0.232	9.32E-04	2166	SIN3A	Cloacibacterium normanense	0.297	9.55E-03	2899	ZNF678	Caulobacteraceae	-0.336	3.34E-03
701	DPY19L3	Microbacteriaceae	-0.319	1.90E-03	1434	MSANTD2	Alphaproteobacteria	-0.418	5.41E-03	2167	SIN3B	Prevotella melaninogenica	-0.301	2.69E-03	2900	ZNF680	Ralstonia insidiosa	-0.210	1.89E-05
702	DPY19L3	Ralstonia insidiosa	-0.259	2.34E-06	1435	MSH3	Geodermatophilaceae	0.440	9.15E-03	2168	SIPA1	Ralstonia insidiosa	0.235	3.58E-12	2901	ZNF684	Ralstonia insidiosa	-0.193	2.83E-03
703	DPY19L4	Caulobacteraceae	-0.371	5.69E-03	1436	MSL1	Alphaproteobacteria	-0.383	2.74E-03	2169	SIPA1	Sphaerotilaceae	0.355	8.38E-04	2902	ZNF688	Ascomycota	-0.425	4.64E-03
704	DPY19L4	Ralstonia insidiosa	-0.286	2.25E-07	1437	MTDH	Enterobacteriaceae	-0.294	1.28E-04	2170	SIPA1L3	Ralstonia insidiosa	0.253	1.35E-05	2903	ZNF691	Alphaproteobacteria	-0.407	7.15E-03
705	DRAM2	Ralstonia insidiosa	-0.283	1.92E-05	1438	MTERF3	Ralstonia insidiosa	-0.200	1.14E-03	2171	SIRT1	Ralstonia insidiosa	-0.133	3.27E-04	2904	ZNF7	Prevotella melaninogenica	-0.353	9.90E-04
706	DSC2	Bacillales	0.453	4.62E-04	1439	MTHFS	Bacillales	-0.327	2.84E-04	2172	SIX4	Aspergillaceae	-0.264	4.95E-03	2905	ZNF700	Alphaproteobacteria	-0.459	5.90E-04
707	DSC2	Ralstonia insidiosa	-0.274	1.43E-04	1440	MTHFS	Malassezia vespertilionis	-0.232	4.85E-03	2173	SIX4	Mycobacteriaceae	0.167	1.99E-03	2906	ZNF700	Oscillospiraceae	-0.362	8.62E-04
708	DST	Actinomyces naeslundii	0.274	1.18E-04	1441	MTLN	Bacillales	-0.443	4.62E-03	2174	SIX5	Bacillales	-0.425	3.81E-03	2907	ZNF728	Brucella	-0.073	8.14E-03
709	DSTN	Ralstonia insidiosa	-0.221	5.07E-03	1442	MTMR10	Ralstonia insidiosa	-0.154	3.84E-03	2175	SIX5	Ralstonia insidiosa	0.268	5.83E-07	2908	ZNF768	Bacillales	-0.313	4.18E-03
710	DSTYK	Delftia acidovorans	0.284	5.82E-03	1443	MTMR12	Porphyromonadaceae	0.404	5.25E-03	2176	SKI	Ralstonia insidiosa	0.212	2.32E-03	2909	ZNF770	Ralstonia insidiosa	-0.250	5.32E-09
711	DTWD1	Caulobacteraceae	-0.307	6.24E-05	1444	MTMR2	Bacillales	0.434	1.76E-04	2177	SKIL	Betaproteobacteria	-0.221	6.13E-04	2910	ZNF771	Bacillales	-0.447	3.45E-04
712	DTWD1	Malassezia vespertilionis	0.329	2.12E-03	1445	MTPN	Saccharomycetales	0.434	7.36E-05	2178	SLC16A3	Ralstonia insidiosa	0.196	4.80E-03	2911	ZNF776	Ralstonia insidiosa	-0.225	8.65E-03
713	DTX2	Ralstonia insidiosa	0.282	8.39E-06	1446	MTRFR	Cloacibacterium normanense	-0.337	2.56E-03	2179	SLC17A2	Methyloversatilis sp. RAC08	0.300	2.39E-03	2912	ZNF777	Ralstonia insidiosa	0.147	1.65E-07
714	DUS1L	Bacillales	-0.369	7.97E-03	1447	MTUS1	Porphyromonadaceae	0.375	8.11E-04	2180	SLC17A5	Brevibacteriaceae	0.024	6.86E-04	2913	ZNF780A	Ralstonia insidiosa	-0.226	1.97E-07
715	DUS1L	Nocardioides	0.123	5.29E-03	1448	MTX2	Leptotrichiaceae	0.171	3.40E-10	2181	SLC19A2	Ralstonia insidiosa	-0.257	7.81E-05	2914	ZNF787	Bacillales	-0.412	8.37E-03
716	DUS3L	Bacillales	-0.317	6.51E-04	1449	MTX3	Ralstonia insidiosa	-0.274	2.72E-06	2182	SLC22A18	Ralstonia insidiosa	0.243	7.63E-03	2915	ZNF799	Brevibacteriaceae	0.096	6.77E-03
717	DUS3L	Ralstonia insidiosa	0.254	5.22E-05	1450	MVB12A	Bacillales	-0.405	2.48E-03	2183	SLC22A24	Acidovorax sp. KKS102	-0.169	3.83E-03	2916	ZNF805	Acidovorax sp. KKS102	-0.176	1.63E-05
718	DVL1	Bacillales	-0.326	1.57E-05	1451	MVB12A	Ralstonia insidiosa	0.253	1.71E-03	2184	SLC25A10	Bacillales	-0.343	3.15E-04	2917	ZNF837	Bacillales	-0.407	7.47E-03
719	DYNC2LI1	Malassezia vespertilionis	0.274	6.14E-03	1452	MVB12A	Staphylococcus saprophyticus	-0.367	9.01E-03	2185	SLC25A10	Ralstonia insidiosa	0.222	5.24E-03	2918	ZNF837	Ralstonia insidiosa	0.284	2.28E-03
720	DYNC2LI1	Ralstonia insidiosa	-0.210	1.96E-04	1453	MXD4	Ralstonia insidiosa	0.173	2.84E-04	2186	SLC25A23	Betaproteobacteria	0.263	3.10E-03	2919	ZNF84	Ralstonia insidiosa	-0.196	3.34E-04
721	DYNLT3	Ralstonia insidiosa	-0.254	1.05E-10	1454	MYBBP1A	Ralstonia insidiosa	0.203	9.11E-05	2187	SLC25A27	Alphaproteobacteria	-0.433	9.85E-03	2920	ZNF850	Corynebacterium accolens	0.243	1.15E-05
722	DYRK1A	Cyanobacteriota	-0.191	2.81E-03	1455	MYBL1	Betaproteobacteria	-0.224	4.52E-03	2188	SLC25A28	Alphaproteobacteria	-0.454	8.11E-03	2921	ZNF852	Alphaproteobacteria	-0.381	5.03E-04
723	DYRK1A	Ralstonia insidiosa	-0.173	4.30E-06	1456	MYH14	Ralstonia insidiosa	0.224	9.99E-04	2189	SLC25A28	Colletotrichum lupini	0.339	9.21E-03	2922	ZNF92	Caulobacteraceae	-0.445	1.97E-04
724	DZANK1	Streptococcus oralis	0.084	4.53E-03	1457	MYH9	Ralstonia insidiosa	0.224	5.48E-03	2190	SLC25A40	Ralstonia insidiosa	-0.245	1.19E-06	2923	ZNF92	Ralstonia insidiosa	-0.291	2.45E-07
725	EARS2	Alphaproteobacteria	0.418	5.95E-04	1458	MYL12B	Sclerotiniaceae	0.200	1.68E-03	2191	SLC25A45	Ralstonia insidiosa	0.183	3.10E-05	2924	ZNHIT2	Ralstonia insidiosa	0.302	9.43E-03
726	EBAG9	Ralstonia insidiosa	-0.285	1.45E-07	1459	MYL9	Ralstonia insidiosa	0.224	7.48E-03	2192	SLC25A46	Ralstonia insidiosa	-0.292	2.59E-11	2925	ZRANB2	Betaproteobacteria	-0.327	5.94E-04
727	EBF4	Ralstonia insidiosa	0.189	1.49E-04	1460	MYLK	Cloacibacterium normanense	0.426	8.53E-03	2193	SLC29A3	Caulobacteraceae	0.232	4.97E-03	2926	ZRANB2	Enterobacteriaceae	-0.294	3.43E-03
728	EBF4	Staphylococcaceae	0.405	5.64E-03	1461	MYLK	Porphyromonadaceae	0.445	3.01E-03	2194	SLC2A4RG	Bacillales	-0.456	9.16E-03	2927	ZSCAN26	Ralstonia insidiosa	-0.285	9.55E-04
729	EBLN2	Corynebacterium accolens	0.218	8.97E-03	1462	MYNN	Caulobacteraceae	-0.350	1.84E-03	2195	SLC30A5	Ralstonia insidiosa	-0.304	8.31E-10	2928	ZSCAN30	Actinomyces naeslundii	0.090	5.97E-04
730	EBNA1BP2	Alphaproteobacteria	-0.441	8.65E-03	1463	MYNN	Ralstonia insidiosa	-0.248	1.13E-10	2196	SLC30A6	Ralstonia insidiosa	-0.245	3.11E-03	2929	ZXDA	Saccharomycetales	0.338	7.67E-03
731	ECHDC3	Acidovorax temperans	-0.078	8.84E-03	1464	MYO15A	Streptococcus oralis	-0.025	6.80E-04	2197	SLC30A9	Ralstonia insidiosa	-0.288	6.21E-06	2930	ZYG11B	Ralstonia insidiosa	-0.207	2.49E-05
732	EDC4	Ralstonia insidiosa	0.231	6.00E-06	1465	MYO18A	Enterobacteriaceae	0.229	6.13E-03	2198	SLC31A1	Ascomycota	0.450	7.97E-03	2931	ZYX	Debaryomycetaceae	-0.331	1.83E-05
733	EDEM3	Ralstonia insidiosa	-0.248	9.84E-06	1466	MYSM1	Ralstonia insidiosa	-0.179	4.35E-03	2199	SLC34A2	Corynebacteriaceae	0.285	3.06E-03	2932	ZYX	Ralstonia insidiosa	0.180	2.74E-07

ZZZ3 Ralstonia insidi ##

Supplementary Table 15. Multi-variable regression of interaction strength on various clinical variables.

Model				Coefficient											
				Linear Variable					Non-Linear Variable						
ID	Formula	R^2	P	NAS	Fibrosis stage	Sex	Age	Sequencing Instrument	NAS^2	NAS^3	Fibrosis $stage^2$	Fibrosis $stage^3$	Age^2	Age^3
1	Interaction strength ~ NAS + Fibrosis stage + Age + Sex + Instrument	0.1474	*	*	ns	ns	ns	*	-	-	-	-	-	-
2	Interaction strength ~ NAS + NAS^2 + NAS^3+ Fibrosis stage + Age + Sex + Instrument	0.3056	*	*	ns	ns	ns	ns	*	*	-	-	-	-
3	Interaction strength ~ NAS + NAS^2 + NAS^3+ Fibrosis stage + Fibrosis $stage^2$+ Fibrosis $stage^3$ + Age + Sex + Instrument	0.3058	*	*	ns	ns	ns	*	*	*	ns	ns	-	-
4	Interaction strength ~ NAS + NAS^2 + NAS^3 + Fibrosis stage + Fibrosis $stage^2$ + Fibrosis $stage^3$ + Age + Age^2 + Age^3 + Sex + Instrument	0.3021	*	*	ns	ns	ns	ns	*	*	ns	ns	ns	ns

NAS, NAFLD Activity Score; P: p-value

ns: not significant (p value > 0.05)； *: significant (p value < 0.05); -: not included in the model

Supplementary Table 16: Functional annotation of host-microbiota interactions by SparceCCA in all stages in discovery data

	category	subcategory	ID	Description	GeneRatio	BgRatio	pvalue	FDR	Cluster	stage
1	Human Diseases	Cardiovascular disease	hsa05415	Diabetic cardiomyopathy	135/3878	203/8644	2.71E-10	9.17E-08	gene_taxa_component_4	Control
2	Metabolism	Energy metabolism	hsa00190	Oxidative phosphorylation	92/3878	134/8644	1.82E-08	1.09E-06	gene_taxa_component_4	Control
3	Genetic Information Processing	Translation	hsa03010	Ribosome	112/3935	167/8644	1.20E-08	1.13E-06	gene_taxa_component_10	Control
4	Human Diseases	Endocrine and metabolic disease	hsa04932	Non-alcoholic fatty liver disease	102/3878	155/8644	9.47E-08	4.57E-06	gene_taxa_component_4	Control
5	Organismal Systems	Environmental adaptation	hsa04714	Thermogenesis	142/3878	232/8644	2.93E-07	9.00E-06	gene_taxa_component_4	Control
6	Metabolism	Lipid metabolism	hsa00071	Fatty acid degradation	35/3494	43/8644	4.27E-08	1.44E-05	gene_taxa_component_9	Control
7	Cellular Processes	Cell motility	hsa04810	Regulation of actin cytoskeleton	143/3889	229/8644	5.60E-08	1.89E-05	gene_taxa_component_1	Control
8	Organismal Systems	Endocrine system	hsa04919	Thyroid hormone signaling pathway	81/3878	121/8644	6.82E-07	1.92E-05	gene_taxa_component_4	Control
9	Organismal Systems	Immune system	hsa04611	Platelet activation	82/3878	124/8644	1.26E-06	3.28E-05	gene_taxa_component_4	Control
10	Human Diseases	Endocrine and metabolic disease	hsa04933	AGE-RAGE signaling pathway in diabetic complications	68/3878	100/8644	2.26E-06	5.10E-05	gene_taxa_component_4	Control
11	Cellular Processes	Transport and catabolism	hsa04140	Autophagy - animal	103/3878	165/8644	3.52E-06	7.00E-05	gene_taxa_component_4	Control
12	Human Diseases	Cancer: overview	hsa05208	Chemical carcinogenesis - reactive oxygen species	133/3878	223/8644	5.04E-06	9.47E-05	gene_taxa_component_4	Control
13	Cellular Processes	Cellular community - eukaryotes	hsa04510	Focal adhesion	124/3935	203/8644	4.74E-06	0.00014572	gene_taxa_component_10	Control
14	Cellular Processes	Transport and catabolism	hsa04144	Endocytosis	146/3878	250/8644	8.94E-06	0.00015906	gene_taxa_component_4	Control
15	Environmental Information Processing	Signal transduction	hsa04015	Rap1 signaling pathway	125/3759	210/8644	1.64E-06	0.00018655	gene_taxa_component_3	Control
16	Human Diseases	Infectious disease: bacterial	hsa05130	Pathogenic Escherichia coli infection	121/3889	198/8644	2.93E-06	0.00041756	gene_taxa_component_1	Control
17	Human Diseases	Cancer: specific types	hsa05223	Non-small cell lung cancer	51/3889	72/8644	7.57E-06	0.00045181	gene_taxa_component_1	Control
18	Cellular Processes	Cell growth and death	hsa04110	Cell cycle	102/4000	157/8644	1.51E-06	0.00050919	gene_taxa_component_6	Control
19	Human Diseases	Cancer: specific types	hsa05216	Thyroid cancer	29/3878	37/8644	3.23E-05	0.0005462	gene_taxa_component_4	Control
20	Metabolism	Amino acid metabolism	hsa00310	Lysine degradation	43/3494	63/8644	6.90E-06	0.00068912	gene_taxa_component_9	Control
21	Organismal Systems	Immune system	hsa04610	Complement and coagulation cascades	55/3494	86/8644	8.16E-06	0.00068912	gene_taxa_component_9	Control
22	Environmental Information Processing	Signal transduction	hsa04010	MAPK signaling pathway	171/3889	301/8644	1.86E-05	0.00069745	gene_taxa_component_1	Control
23	Genetic Information Processing	Folding, sorting and degradation	hsa04141	Protein processing in endoplasmic reticulum	102/3878	170/8644	4.43E-05	0.00071356	gene_taxa_component_4	Control
24	Human Diseases	Cancer: overview	hsa05205	Proteoglycans in cancer	120/3878	205/8644	4.78E-05	0.00072105	gene_taxa_component_4	Control
25	Environmental Information Processing	Signal transduction	hsa04070	Phosphatidylinositol signaling system	63/3878	97/8644	4.91E-05	0.00072105	gene_taxa_component_4	Control
26	Cellular Processes	Cellular community - eukaryotes	hsa04530	Tight junction	103/3889	170/8644	2.65E-05	0.00081471	gene_taxa_component_1	Control
27	Genetic Information Processing	Folding, sorting and degradation	hsa03050	Proteasome	34/3878	46/8644	5.88E-05	0.00082846	gene_taxa_component_4	Control
28	Environmental Information Processing	Signaling molecules and interaction	hsa04514	Cell adhesion molecules	101/4000	158/8644	5.02E-06	0.00084895	gene_taxa_component_6	Control
29	Cellular Processes	Cellular community - eukaryotes	hsa04520	Adherens junction	64/4000	93/8644	8.49E-06	0.00095625	gene_taxa_component_6	Control
30	Metabolism	Global and overview maps	hsa01212	Fatty acid metabolism	39/3494	57/8644	1.69E-05	0.00104802	gene_taxa_component_9	Control
31	Metabolism	Amino acid metabolism	hsa00280	Valine, leucine and isoleucine degradation	34/3494	48/8644	1.86E-05	0.00104802	gene_taxa_component_9	Control
32	Cellular Processes	Cell motility	hsa04814	Motor proteins	115/3935	193/8644	5.06E-05	0.00113975	gene_taxa_component_10	Control
33	Human Diseases	Infectious disease: bacterial	hsa05131	Shigellosis	140/3878	247/8644	0.0001028	0.00133646	gene_taxa_component_4	Control
34	Organismal Systems	Endocrine system	hsa04921	Oxytocin signaling pathway	92/3759	154/8644	3.12E-05	0.00150765	gene_taxa_component_3	Control
35	Environmental Information Processing	Signal transduction	hsa04668	TNF signaling pathway	71/3759	114/8644	3.71E-05	0.0015684	gene_taxa_component_3	Control
36	Human Diseases	Cancer: specific types	hsa05212	Pancreatic cancer	51/3889	76/8644	7.82E-05	0.00188732	gene_taxa_component_1	Control
37	Cellular Processes	Cell growth and death	hsa04115	p53 signaling pathway	52/4000	74/8644	2.38E-05	0.0019339	gene_taxa_component_6	Control
38	Metabolism	Carbohydrate metabolism	hsa00520	Amino sugar and nucleotide sugar metabolism	37/4000	49/8644	2.86E-05	0.0019339	gene_taxa_component_6	Control
39	Cellular Processes	Transport and catabolism	hsa04142	Lysosome	80/3878	132/8644	0.00017992	0.00212636	gene_taxa_component_4	Control
40	Organismal Systems	Immune system	hsa04062	Chemokine signaling pathway	111/3878	192/8644	0.00018244	0.00212636	gene_taxa_component_4	Control
41	Human Diseases	Cancer: specific types	hsa05210	Colorectal cancer	56/3889	86/8644	0.00012577	0.00223768	gene_taxa_component_1	Control
42	Genetic Information Processing	Folding, sorting and degradation	hsa03060	Protein export	19/3878	23/8644	0.00023153	0.00260856	gene_taxa_component_4	Control
43	Cellular Processes	Cell growth and death	hsa04218	Cellular senescence	92/3878	156/8644	0.00024604	0.00267154	gene_taxa_component_4	Control
44	Metabolism	Lipid metabolism	hsa00120	Primary bile acid biosynthesis	15/3494	17/8644	6.50E-05	0.00313981	gene_taxa_component_9	Control
45	Metabolism	Global and overview maps	hsa01200	Carbon metabolism	70/3759	115/8644	0.00011969	0.00314092	gene_taxa_component_3	Control
46	Environmental Information Processing	Signal transduction	hsa04064	NF-kappa B signaling pathway	64/3759	104/8644	0.00014733	0.00327035	gene_taxa_component_3	Control
47	Organismal Systems	Immune system	hsa04625	C-type lectin receptor signaling pathway	64/3759	104/8644	0.00014733	0.00327035	gene_taxa_component_3	Control
48	Environmental Information Processing	Signal transduction	hsa04022	cGMP-PKG signaling pathway	97/3878	167/8644	0.00036219	0.00370966	gene_taxa_component_4	Control
49	Genetic Information Processing	Folding, sorting and degradation	hsa04120	Ubiquitin mediated proteolysis	84/3878	142/8644	0.00039215	0.00389842	gene_taxa_component_4	Control
50	Organismal Systems	Immune system	hsa04670	Leukocyte transendothelial migration	70/3889	115/8644	0.00041233	0.00464562	gene_taxa_component_1	Control
51	Environmental Information Processing	Signal transduction	hsa04371	Apelin signaling pathway	81/3759	139/8644	0.00028932	0.0048439	gene_taxa_component_3	Control
52	Metabolism	Lipid metabolism	hsa01040	Biosynthesis of unsaturated fatty acids	21/3759	27/8644	0.0003007	0.0048439	gene_taxa_component_3	Control
53	Environmental Information Processing	Signal transduction	hsa04071	Sphingolipid signaling pathway	73/3889	121/8644	0.00045621	0.00497421	gene_taxa_component_1	Control
54	Organismal Systems	Immune system	hsa04666	Fc gamma R-mediated phagocytosis	63/4000	97/8644	0.00015083	0.00509806	gene_taxa_component_6	Control
55	Human Diseases	Cardiovascular disease	hsa05416	Viral myocarditis	46/4000	67/8644	0.00017016	0.00522844	gene_taxa_component_6	Control
56	Metabolism	Glycan biosynthesis and metabolism	hsa00510	N-Glycan biosynthesis	36/3878	53/8644	0.00057424	0.00539147	gene_taxa_component_4	Control
57	Genetic Information Processing	Replication and repair	hsa03410	Base excision repair	31/3889	44/8644	0.00055071	0.00576678	gene_taxa_component_1	Control

58	Human Diseases	Infectious disease: bacterial	hsa05135	Yersinia infection	81/3889	137/8644	0.00056303	0.00576678	gene taxa component 1	Control
59	Human Diseases	Drug resistance: antineoplastic	hsa01522	Endocrine resistance	60/3678	98/8644	0.00013951	0.00589413	gene taxa component 7	Control
60	Organismal Systems	Immune system	hsa04662	B cell receptor signaling pathway	52/3759	84/8644	0.00048945	0.00663788	gene taxa component 3	Control
61	Human Diseases	Cardiovascular disease	hsa05412	Arrhythmogenic right ventricular cardiomyopathy	52/3759	84/8644	0.00048945	0.00663788	gene taxa component 3	Control
62	Human Diseases	Cancer: specific types	hsa05222	Small cell lung cancer	57/3889	92/8644	0.00074232	0.00716869	gene taxa component 1	Control
63	Human Diseases	Infectious disease: bacterial	hsa05132	Salmonella infection	137/3889	249/8644	0.00080483	0.00744247	gene taxa component 1	Control
64	Human Diseases	Infectious disease: viral	hsa05163	Human cytomegalovirus infection	125/3889	225/8644	0.00081471	0.00744247	gene taxa component 1	Control
65	Organismal Systems	Endocrine system	hsa04928	Parathyroid hormone synthesis, secretion and action	63/3759	106/8644	0.00064541	0.00794786	gene taxa component 3	Control
66	Environmental Information Processing	Signal transduction	hsa04330	Notch signaling pathway	40/3759	62/8644	0.0006584	0.00794786	gene taxa component 3	Control
67	Environmental Information Processing	Signal transduction	hsa04072	Phospholipase D signaling pathway	84/3759	148/8644	0.00072392	0.00843741	gene taxa component 3	Control
68	Human Diseases	Infectious disease: viral	hsa05167	Kaposi sarcoma-associated herpesvirus infection	109/3889	194/8644	0.00100734	0.00873026	gene taxa component 1	Control
69	Metabolism	Carbohydrate metabolism	hsa00020	Citrate cycle (TCA cycle)	22/3494	30/8644	0.00025983	0.00890459	gene taxa component 9	Control
70	Human Diseases	Cardiovascular disease	hsa05410	Hypertrophic cardiomyopathy	58/3759	97/8644	0.00084528	0.00892832	gene taxa component 3	Control
71	Cellular Processes	Transport and catabolism	hsa04136	Autophagy - other	24/3935	32/8644	0.00067246	0.00901834	gene taxa component 10	Control
72	Human Diseases	Cardiovascular disease	hsa05414	Dilated cardiomyopathy	61/3759	103/8644	0.00088723	0.00908737	gene taxa component 3	Control
73	Metabolism	Lipid metabolism	hsa00100	Steroid biosynthesis	16/3759	20/8644	0.00096354	0.0092309	gene taxa component 3	Control
74	Organismal Systems	Endocrine system	hsa04926	Relaxin signaling pathway	74/3759	129/8644	0.00097298	0.0092309	gene taxa component 3	Control
75	Human Diseases	Infectious disease: viral	hsa05169	Epstein-Barr virus infection	110/3759	202/8644	0.00098317	0.0092309	gene taxa component 3	Control
76	Organismal Systems	Immune system	hsa04621	NOD-like receptor signaling pathway	102/3759	186/8644	0.00107968	0.00960345	gene taxa component 3	Control
77	Metabolism	Carbohydrate metabolism	hsa00010	Glycolysis / Gluconeogenesis	42/3759	67/8644	0.00115926	0.00991774	gene taxa component 3	Control
78	Cellular Processes	Cell growth and death	hsa04210	Apoptosis	79/3889	136/8644	0.00135139	0.01015043	gene taxa component 1	Control
79	Metabolism	Carbohydrate metabolism	hsa00562	Inositol phosphate metabolism	46/3878	73/8644	0.00131329	0.01138182	gene taxa component 4	Control
80	Cellular Processes	Transport and catabolism	hsa04146	Peroxisome	50/3759	83/8644	0.00149594	0.01149154	gene taxa component 3	Control
81	Environmental Information Processing	Signaling molecules and interaction	hsa04512	ECM-receptor interaction	53/3759	89/8644	0.00158334	0.01189264	gene taxa component 3	Control
82	Environmental Information Processing	Signal transduction	hsa04068	FoxO signaling pathway	76/3889	131/8644	0.00173319	0.01220455	gene taxa component 1	Control
83	Environmental Information Processing	Signal transduction	hsa04066	HIF-1 signaling pathway	63/3759	109/8644	0.00174216	0.01252875	gene taxa component 3	Control
84	Human Diseases	Cardiovascular disease	hsa05418	Fluid shear stress and atherosclerosis	80/3889	139/8644	0.00182142	0.01256409	gene taxa component 1	Control
85	Human Diseases	Cancer: overview	hsa05230	Central carbon metabolism in cancer	44/3878	70/8644	0.00178511	0.0131167	gene taxa component 4	Control
86	Human Diseases	Immune disease	hsa05323	Rheumatoid arthritis	56/3889	93/8644	0.00214577	0.01368434	gene taxa component 1	Control
87	Human Diseases	Cancer: specific types	hsa05220	Chronic myeloid leukemia	47/3889	76/8644	0.00221969	0.01389364	gene taxa component 1	Control
88	Organismal Systems	Circulatory system	hsa04270	Vascular smooth muscle contraction	77/3878	134/8644	0.00212174	0.01525845	gene taxa component 4	Control
89	Metabolism	Lipid metabolism	hsa00600	Sphingolipid metabolism	35/3889	54/8644	0.00256617	0.01530887	gene taxa component 1	Control
90	Organismal Systems	Immune system	hsa04658	Th1 and Th2 cell differentiation	54/3759	92/8644	0.00226389	0.01536646	gene taxa component 3	Control
91	Human Diseases	Infectious disease: parasitic	hsa05142	Chagas disease	59/3759	102/8644	0.00234368	0.01536646	gene taxa component 3	Control
92	Organismal Systems	Immune system	hsa04659	Th17 cell differentiation	62/3759	108/8644	0.00236407	0.01536646	gene taxa component 3	Control
93	Human Diseases	Cancer: specific types	hsa05221	Acute myeloid leukemia	41/3759	67/8644	0.00256467	0.01576107	gene taxa component 3	Control
94	Environmental Information Processing	Signal transduction	hsa04390	Hippo signaling pathway	92/4000	157/8644	0.00117654	0.01590686	gene taxa component 6	Control
95	Organismal Systems	Endocrine system	hsa04924	Renin secretion	42/3759	69/8644	0.00263781	0.01592109	gene taxa component 3	Control
96	Environmental Information Processing	Membrane transport	hsa02010	ABC transporters	30/3878	45/8644	0.00256595	0.01636397	gene taxa component 4	Control
97	Metabolism	Carbohydrate metabolism	hsa00630	Glyoxylate and dicarboxylate metabolism	21/3759	30/8644	0.00299913	0.0177843	gene taxa component 3	Control
98	Organismal Systems	Environmental adaptation	hsa04713	Circadian entrainment	56/3759	97/8644	0.00316892	0.0178516	gene taxa component 3	Control
99	Human Diseases	Cancer: specific types	hsa05214	Glioma	46/3889	75/8644	0.00311507	0.01815333	gene taxa component 1	Control
100	Human Diseases	Infectious disease: parasitic	hsa05140	Leishmaniasis	49/4000	77/8644	0.00155275	0.01908334	gene taxa component 6	Control
101	Human Diseases	Infectious disease: bacterial	hsa05120	Epithelial cell signaling in Helicobacter pylori infection	42/3759	70/8644	0.00385148	0.02027336	gene taxa component 3	Control
102	Metabolism	Carbohydrate metabolism	hsa00620	Pyruvate metabolism	30/3759	47/8644	0.00386784	0.02027336	gene taxa component 3	Control
103	Metabolism	Carbohydrate metabolism	hsa00640	Propanoate metabolism	23/3935	32/8644	0.00228229	0.02142821	gene taxa component 10	Control
104	Organismal Systems	Sensory system	hsa04750	Inflammatory mediator regulation of TRP channels	56/3759	98/8644	0.00430194	0.0217023	gene taxa component 3	Control
105	Human Diseases	Infectious disease: bacterial	hsa05133	Pertussis	46/3889	76/8644	0.00448798	0.02446671	gene taxa component 1	Control
106	Genetic Information Processing	Chromosome	hsa03083	Polycomb repressive complex	50/3878	84/8644	0.00469037	0.02520475	gene taxa component 4	Control
107	Genetic Information Processing	Replication and repair	hsa03420	Nucleotide excision repair	39/3878	63/8644	0.00469793	0.02520475	gene taxa component 4	Control
108	Organismal Systems	Endocrine system	hsa04927	Cortisol synthesis and secretion	39/3759	65/8644	0.00525866	0.02539183	gene taxa component 3	Control
109	Metabolism	Glycan biosynthesis and metabolism	hsa00513	Various types of N-glycan biosynthesis	28/3678	42/8644	0.00135685	0.02540085	gene taxa component 7	Control
110	Human Diseases	Infectious disease: bacterial	hsa05110	Vibrio cholerae infection	32/3878	50/8644	0.00489933	0.02547653	gene taxa component 4	Control
111	Human Diseases	Cancer: specific types	hsa05211	Renal cell carcinoma	42/3678	69/8644	0.00159317	0.02564247	gene taxa component 7	Control
112	Metabolism	Global and overview maps	hsa01250	Biosynthesis of nucleotide sugars	26/4000	37/8644	0.00269079	0.02598533	gene taxa component 6	Control
113	Organismal Systems	Immune system	hsa04640	Hematopoietic cell lineage	56/3759	99/8644	0.00576527	0.02604444	gene taxa component 3	Control
114	Metabolism	Amino acid metabolism	hsa00380	Tryptophan metabolism	27/3494	42/8644	0.00148976	0.02787876	gene taxa component 9	Control
115	Metabolism	Lipid metabolism	hsa00062	Fatty acid elongation	19/3494	27/8644	0.00156715	0.02787876	gene taxa component 9	Control
116	Metabolism	Nucleotide metabolism	hsa00230	Purine metabolism	70/3759	128/8644	0.00667665	0.02893214	gene taxa component 3	Control
117	Metabolism	Glycan biosynthesis and metabolism	hsa00604	Glycosphingolipid biosynthesis - ganglio series	12/3889	15/8644	0.00627617	0.03153409	gene taxa component 1	Control

118	Human Diseases	Infectious disease: bacterial	hsa05100	Bacterial invasion of epithelial cells	46/3889	77/8644	0.00634414	0.03153409	gene taxa component 1	Control
119	Human Diseases	Cancer: specific types	hsa05213	Endometrial cancer	36/3889	58/8644	0.0064499	0.03159515	gene taxa component 1	Control
120	Metabolism	Global and overview maps	hsa01232	Nucleotide metabolism	50/3878	85/8644	0.00648883	0.03178588	gene taxa component 4	Control
121	Organismal Systems	Endocrine system	hsa04925	Aldosterone synthesis and secretion	55/3759	98/8644	0.00767819	0.03238448	gene taxa component 3	Control
122	Organismal Systems	Endocrine system	hsa04911	Insulin secretion	49/3759	86/8644	0.00785659	0.03238448	gene taxa component 3	Control
123	Cellular Processes	Transport and catabolism	hsa04145	Phagosome	87/4000	152/8644	0.00403412	0.03408835	gene taxa component 6	Control
124	Organismal Systems	Endocrine system	hsa04929	GnRH secretion	39/3889	64/8644	0.00728663	0.03468848	gene taxa component 1	Control
125	Metabolism	Amino acid metabolism	hsa00220	Arginine biosynthesis	16/3878	22/8644	0.00764006	0.03557579	gene taxa component 4	Control
126	Organismal Systems	Endocrine system	hsa04910	Insulin signaling pathway	76/3878	137/8644	0.00768353	0.03557579	gene taxa component 4	Control
127	Organismal Systems	Digestive system	hsa04979	Cholesterol metabolism	32/3678	51/8644	0.00282733	0.03581302	gene taxa component 7	Control
128	Human Diseases	Infectious disease: parasitic	hsa05145	Toxoplasmosis	62/3678	111/8644	0.00307271	0.03581302	gene taxa component 7	Control
129	Human Diseases	Immune disease	hsa05330	Allograft rejection	26/4000	38/8644	0.00479308	0.03600134	gene taxa component 6	Control
130	Organismal Systems	Endocrine system	hsa04935	Growth hormone synthesis, secretion and action	66/3678	120/8644	0.00380913	0.03801255	gene taxa component 7	Control
131	Environmental Information Processing	Signal transduction	hsa04014	Ras signaling pathway	121/3678	236/8644	0.00382375	0.03801255	gene taxa component 7	Control
132	Human Diseases	Endocrine and metabolic disease	hsa04930	Type II diabetes mellitus	29/3678	46/8644	0.00397959	0.03843149	gene taxa component 7	Control
133	Human Diseases	Cardiovascular disease	hsa05417	Lipid and atherosclerosis	111/3678	215/8644	0.0041119	0.03860614	gene taxa component 7	Control
134	Environmental Information Processing	Signal transduction	hsa04020	Calcium signaling pathway	133/3889	253/8644	0.00844316	0.03909298	gene taxa component 1	Control
135	Organismal Systems	Endocrine system	hsa04912	GnRH signaling pathway	52/3759	93/8644	0.01031077	0.03987199	gene taxa component 3	Control
136	Human Diseases	Substance dependence	hsa05032	Morphine addiction	51/3759	91/8644	0.01038087	0.03987199	gene taxa component 3	Control
137	Metabolism	Glycan biosynthesis and metabolism	hsa00603	Glycosphingolipid biosynthesis - globo and isoglobo series	12/3759	16/8644	0.010808	0.04014399	gene taxa component 3	Control
138	Cellular Processes	Cellular community - eukaryotes	hsa04540	Gap junction	51/3878	88/8644	0.00895179	0.04034275	gene taxa component 4	Control
139	Environmental Information Processing	Signal transduction	hsa04370	VEGF signaling pathway	36/3889	59/8644	0.00946532	0.04323348	gene taxa component 1	Control
140	Organismal Systems	Aging	hsa04213	Longevity regulating pathway - multiple species	37/3889	61/8644	0.00979804	0.0441565	gene taxa component 1	Control
141	Human Diseases	Cancer: overview	hsa05231	Choline metabolism in cancer	56/3889	98/8644	0.01006992	0.04478465	gene taxa component 1	Control
142	Cellular Processes	Cell growth and death	hsa04114	Oocyte meiosis	75/4000	131/8644	0.00718812	0.04584121	gene taxa component 6	Control
143	Human Diseases	Infectious disease: parasitic	hsa05144	Malaria	31/3889	50/8644	0.01135951	0.04799392	gene taxa component 1	Control
144	Organismal Systems	Circulatory system	hsa04261	Adrenergic signaling in cardiomyocytes	81/3759	154/8644	0.01353933	0.0481715	gene taxa component 3	Control
145	Human Diseases	Drug resistance: antineoplastic	hsa01521	EGFR tyrosine kinase inhibitor resistance	46/3878	79/8644	0.01131599	0.04841524	gene taxa component 4	Control
146	Genetic Information Processing	Translation	hsa03010	Ribosome	22/205	167/8644	4.76E-11	1.19E-08	gene taxa component 4	NAFL
147	Cellular Processes	Transport and catabolism	hsa04144	Endocytosis	21/214	250/8644	8.89E-07	0.0002302	gene taxa component 3	NAFL
148	Human Diseases	Infectious disease: bacterial	hsa05135	Yersinia infection	13/214	137/8644	3.26E-05	0.00234308	gene taxa component 3	NAFL
149	Human Diseases	Cancer: overview	hsa05208	Chemical carcinogenesis - reactive oxygen species	17/214	223/8644	3.62E-05	0.00234308	gene taxa component 3	NAFL
150	Genetic Information Processing	Folding, sorting and degradation	hsa04120	Ubiquitin mediated proteolysis	13/214	142/8644	4.75E-05	0.00246244	gene taxa component 3	NAFL
151	Genetic Information Processing	Chromosome	hsa03083	Polycomb repressive complex	10/202	84/8644	2.38E-05	0.00277535	gene taxa component 7	NAFL
152	Environmental Information Processing	Signal transduction	hsa04012	ErbB signaling pathway	9/214	85/8644	0.00023796	0.00880455	gene taxa component 3	NAFL
153	Cellular Processes	Cell growth and death	hsa04216	Ferroptosis	7/212	41/8644	5.35E-05	0.00885599	gene taxa component 1	NAFL
154	Genetic Information Processing	Translation	hsa03013	Nucleocytoplasmic transport	11/212	108/8644	6.39E-05	0.00885599	gene taxa component 1	NAFL
155	Environmental Information Processing	Signal transduction	hsa04370	VEGF signaling pathway	7/214	59/8644	0.00059081	0.01804863	gene taxa component 3	NAFL
156	Human Diseases	Infectious disease: bacterial	hsa05130	Pathogenic Escherichia coli infection	13/214	198/8644	0.00124888	0.03105634	gene taxa component 3	NAFL
157	Organismal Systems	Immune system	hsa04620	Toll-like receptor signaling pathway	9/214	108/8644	0.00138496	0.03105634	gene taxa component 3	NAFL
158	Human Diseases	Cancer: overview	hsa05235	PD-L1 expression and PD-1 checkpoint pathway in cancer	8/214	89/8644	0.0015652	0.03105634	gene taxa component 3	NAFL
159	Organismal Systems	Endocrine system	hsa04917	Prolactin signaling pathway	7/214	70/8644	0.00165054	0.03105634	gene taxa component 3	NAFL
160	Human Diseases	Infectious disease: parasitic	hsa05145	Toxoplasmosis	9/214	111/8644	0.00167872	0.03105634	gene taxa component 3	NAFL
161	Organismal Systems	Environmental adaptation	hsa04714	Thermogenesis	15/205	232/8644	0.00039862	0.03308553	gene taxa component 4	NAFL
162	Human Diseases	Cancer: specific types	hsa05220	Chronic myeloid leukemia	7/214	76/8644	0.00265705	0.03931229	gene taxa component 3	NAFL
163	Human Diseases	Infectious disease: bacterial	hsa05100	Bacterial invasion of epithelial cells	7/214	77/8644	0.00286269	0.03931229	gene taxa component 3	NAFL
164	Human Diseases	Drug resistance: antineoplastic	hsa01522	Endocrine resistance	8/214	98/8644	0.00288615	0.03931229	gene taxa component 3	NAFL
165	Organismal Systems	Immune system	hsa04660	T cell receptor signaling pathway	9/214	121/8644	0.0030357	0.03931229	gene taxa component 3	NAFL
166	Organismal Systems	Immune system	hsa04610	Complement and coagulation cascades	25/1253	86/8644	0.00035517	0.04033696	gene taxa component 10	NAFL
167	Cellular Processes	Cellular community - eukaryotes	hsa04530	Tight junction	11/214	170/8644	0.00327361	0.04037457	gene taxa component 3	NAFL
168	Cellular Processes	Transport and catabolism	hsa04137	Mitophagy - animal	8/214	103/8644	0.0039262	0.04622214	gene taxa component 3	NAFL
169	Genetic Information Processing	Translation	hsa03010	Ribosome	73/1744	167/8644	2.92E-12	9.86E-10	gene taxa component 10	Borderline
170	Cellular Processes	Transport and catabolism	hsa04144	Endocytosis	79/1466	250/8644	6.09E-09	2.05E-06	gene taxa component 1	Borderline
171	Human Diseases	Cancer: overview	hsa05208	Chemical carcinogenesis - reactive oxygen species	77/1744	223/8644	2.86E-07	1.93E-05	gene taxa component 10	Borderline
172	Organismal Systems	Immune system	hsa04610	Complement and coagulation cascades	39/1724	86/8644	7.71E-08	2.61E-05	gene taxa component 8	Borderline
173	Organismal Systems	Immune system	hsa04670	Leukocyte transendothelial migration	46/1724	115/8644	5.33E-07	9.02E-05	gene taxa component 8	Borderline
174	Cellular Processes	Transport and catabolism	hsa04142	Lysosome	47/1543	132/8644	7.35E-07	0.00012419	gene taxa component 2	Borderline
175	Metabolism	Energy metabolism	hsa00190	Oxidative phosphorylation	49/1744	134/8644	6.99E-06	0.00029535	gene taxa component 10	Borderline
176	Human Diseases	Endocrine and metabolic disease	hsa04932	Non-alcoholic fatty liver disease	54/1744	155/8644	1.26E-05	0.00047262	gene taxa component 10	Borderline
177	Human Diseases	Cardiovascular disease	hsa05415	Diabetic cardiomyopathy	66/1744	203/8644	1.97E-05	0.00060386	gene taxa component 10	Borderline

178	Human Diseases	Infectious disease: bacterial	hsa05150	Staphylococcus aureus infection	38/1724	96/8644	6.86E-06	0.00077246	gene taxa component 8	Borderline
179	Human Diseases	Infectious disease: parasitic	hsa05140	Leishmaniasis	32/1724	77/8644	1.10E-05	0.00093227	gene taxa component 8	Borderline
180	Environmental Information Processing	Signal transduction	hsa04010	MAPK signaling pathway	94/1752	301/8644	3.21E-06	0.0010838	gene taxa component 9	Borderline
181	Metabolism	Lipid metabolism	hsa00600	Sphingolipid metabolism	26/1810	54/8644	7.33E-06	0.00123474	gene taxa component 6	Borderline
182	Organismal Systems	Environmental adaptation	hsa04714	Thermogenesis	72/1744	232/8644	4.83E-05	0.00125504	gene taxa component 10	Borderline
183	Organismal Systems	Immune system	hsa04662	B cell receptor signaling pathway	33/1724	84/8644	3.26E-05	0.0015751	gene taxa component 8	Borderline
184	Cellular Processes	Transport and catabolism	hsa04145	Phagosome	51/1724	152/8644	4.96E-05	0.00209376	gene taxa component 8	Borderline
185	Genetic Information Processing	Chromosome	hsa03083	Polycomb repressive complex	34/1810	84/8644	3.44E-05	0.0038674	gene taxa component 6	Borderline
186	Cellular Processes	Cellular community - eukaryotes	hsa04510	Focal adhesion	66/1752	203/8644	2.29E-05	0.00387022	gene taxa component 9	Borderline
187	Cellular Processes	Transport and catabolism	hsa04140	Autophagy - animal	55/1752	165/8644	5.02E-05	0.00507776	gene taxa component 9	Borderline
188	Human Diseases	Cancer: specific types	hsa05222	Small cell lung cancer	35/1752	92/8644	6.01E-05	0.00507776	gene taxa component 9	Borderline
189	Environmental Information Processing	Signaling molecules and interaction	hsa04514	Cell adhesion molecules	51/1724	158/8644	0.00015191	0.00540942	gene taxa component 8	Borderline
190	Genetic Information Processing	Folding, sorting and degradation	hsa03050	Proteasome	20/1744	46/8644	0.0002755	0.00620784	gene taxa component 10	Borderline
191	Human Diseases	Substance dependence	hsa05033	Nicotine addiction	19/1689	40/8644	5.85E-05	0.00659174	gene taxa component 7	Borderline
192	Human Diseases	Cancer: specific types	hsa05211	Renal cell carcinoma	27/1610	69/8644	5.32E-05	0.00671474	gene taxa component 4	Borderline
193	Cellular Processes	Cell growth and death	hsa04218	Cellular senescence	49/1610	156/8644	7.46E-05	0.00671474	gene taxa component 4	Borderline
194	Genetic Information Processing	Transcription	hsa03020	RNA polymerase	16/1744	34/8644	0.00037696	0.00796322	gene taxa component 10	Borderline
195	Genetic Information Processing	Folding, sorting and degradation	hsa04141	Protein processing in endoplasmic reticulum	49/1466	170/8644	7.17E-05	0.00829008	gene taxa component 1	Borderline
196	Metabolism	Global and overview maps	hsa01240	Biosynthesis of cofactors	45/1466	153/8644	8.27E-05	0.00829008	gene taxa component 1	Borderline
197	Human Diseases	Cardiovascular disease	hsa05416	Viral myocarditis	26/1724	67/8644	0.00027522	0.0083857	gene taxa component 8	Borderline
198	Organismal Systems	Immune system	hsa04612	Antigen processing and presentation	29/1724	78/8644	0.00029772	0.0083857	gene taxa component 8	Borderline
199	Environmental Information Processing	Signal transduction	hsa04072	Phospholipase D signaling pathway	43/1466	148/8644	0.00015996	0.00882492	gene taxa component 1	Borderline
200	Human Diseases	Cardiovascular disease	hsa05412	Arrhythmogenic right ventricular cardiomyopathy	31/1689	84/8644	0.00014788	0.00917498	gene taxa component 7	Borderline
201	Human Diseases	Cardiovascular disease	hsa05414	Dilated cardiomyopathy	36/1689	103/8644	0.00016287	0.00917498	gene taxa component 7	Borderline
202	Environmental Information Processing	Signal transduction	hsa04020	Calcium signaling pathway	73/1689	253/8644	0.00019148	0.00924575	gene taxa component 7	Borderline
203	Environmental Information Processing	Signal transduction	hsa04151	PI3K-Akt signaling pathway	101/1752	359/8644	0.00016669	0.00968353	gene taxa component 9	Borderline
204	Environmental Information Processing	Signaling molecules and interaction	hsa04512	ECM-receptor interaction	33/1752	89/8644	0.0001719	0.00968353	gene taxa component 9	Borderline
205	Metabolism	Amino acid metabolism	hsa00310	Lysine degradation	26/1810	63/8644	0.00019327	0.01085506	gene taxa component 6	Borderline
206	Organismal Systems	Circulatory system	hsa04260	Cardiac muscle contraction	31/1689	87/8644	0.00030703	0.01297188	gene taxa component 7	Borderline
207	Human Diseases	Infectious disease: viral	hsa05167	Kaposi sarcoma-associated herpesvirus infection	55/1543	194/8644	0.00018122	0.01320891	gene taxa component 2	Borderline
208	Metabolism	Carbohydrate metabolism	hsa00040	Pentose and glucuronate interconversions	16/1543	36/8644	0.0001954	0.01320891	gene taxa component 2	Borderline
209	Organismal Systems	Immune system	hsa04611	Platelet activation	39/1516	124/8644	0.00010352	0.013755	gene taxa component 3	Borderline
210	Human Diseases	Cardiovascular disease	hsa05410	Hypertrophic cardiomyopathy	32/1516	97/8644	0.00015798	0.013755	gene taxa component 3	Borderline
211	Human Diseases	Cancer: overview	hsa05235	PD-L1 expression and PD-1 checkpoint pathway in cancer	30/1516	89/8644	0.00016278	0.013755	gene taxa component 3	Borderline
212	Environmental Information Processing	Signal transduction	hsa04071	Sphingolipid signaling pathway	42/1810	121/8644	0.00029049	0.01398494	gene taxa component 6	Borderline
213	Environmental Information Processing	Signal transduction	hsa04370	VEGF signaling pathway	21/1466	59/8644	0.00042284	0.01420749	gene taxa component 1	Borderline
214	Organismal Systems	Endocrine system	hsa04919	Thyroid hormone signaling pathway	41/1752	121/8644	0.00029652	0.01431748	gene taxa component 9	Borderline
215	Cellular Processes	Cell motility	hsa04810	Regulation of actin cytoskeleton	68/1752	229/8644	0.0003785	0.01533732	gene taxa component 9	Borderline
216	Organismal Systems	Aging	hsa04211	Longevity regulating pathway	32/1752	89/8644	0.00040839	0.01533732	gene taxa component 9	Borderline
217	Cellular Processes	Cell motility	hsa04814	Motor proteins	57/1689	193/8644	0.00049495	0.01672934	gene taxa component 7	Borderline
218	Human Diseases	Infectious disease: bacterial	hsa05152	Tuberculosis	54/1724	180/8644	0.00075754	0.01852692	gene taxa component 8	Borderline
219	Organismal Systems	Immune system	hsa04666	Fc gamma R-mediated phagocytosis	33/1724	97/8644	0.00076739	0.01852692	gene taxa component 8	Borderline
220	Cellular Processes	Cellular community - eukaryotes	hsa04550	Signaling pathways regulating pluripotency of stem cells	43/1610	143/8644	0.00057276	0.02144675	gene taxa component 4	Borderline
221	Human Diseases	Cancer: overview	hsa05205	Proteoglycans in cancer	61/1752	205/8644	0.00069851	0.02230668	gene taxa component 9	Borderline
222	Environmental Information Processing	Signal transduction	hsa04015	Rap1 signaling pathway	62/1752	210/8644	0.00079195	0.02230668	gene taxa component 9	Borderline
223	Genetic Information Processing	Translation	hsa03013	Nucleocytoplasmic transport	32/1466	108/8644	0.00073111	0.02233219	gene taxa component 1	Borderline
224	Environmental Information Processing	Signal transduction	hsa04390	Hippo signaling pathway	46/1610	157/8644	0.0007023	0.0236675	gene taxa component 4	Borderline
225	Metabolism	Carbohydrate metabolism	hsa00630	Glyoxylate and dicarboxylate metabolism	14/1752	30/8644	0.00101407	0.02481023	gene taxa component 9	Borderline
226	Organismal Systems	Digestive system	hsa04974	Protein digestion and absorption	34/1689	103/8644	0.00080821	0.02483402	gene taxa component 7	Borderline
227	Organismal Systems	Immune system	hsa04625	C-type lectin receptor signaling pathway	33/1610	104/8644	0.00087297	0.02491187	gene taxa component 4	Borderline
228	Human Diseases	Infectious disease: bacterial	hsa05131	Shigellosis	66/1610	247/8644	0.00095321	0.02491187	gene taxa component 4	Borderline
229	Environmental Information Processing	Signal transduction	hsa04150	mTOR signaling pathway	45/1610	156/8644	0.00114141	0.02491187	gene taxa component 4	Borderline
230	Environmental Information Processing	Signal transduction	hsa04371	Apelin signaling pathway	41/1610	139/8644	0.00115769	0.02491187	gene taxa component 4	Borderline
231	Human Diseases	Infectious disease: bacterial	hsa05132	Salmonella infection	66/1610	249/8644	0.00119574	0.02491187	gene taxa component 4	Borderline
232	Human Diseases	Cancer: overview	hsa05231	Choline metabolism in cancer	31/1610	98/8644	0.00130612	0.02491187	gene taxa component 4	Borderline
233	Cellular Processes	Cell growth and death	hsa04115	p53 signaling pathway	25/1610	74/8644	0.0013306	0.02491187	gene taxa component 4	Borderline
234	Human Diseases	Cancer: specific types	hsa05214	Glioma	25/1610	75/8644	0.00164947	0.02526682	gene taxa component 4	Borderline
235	Metabolism	Global and overview maps	hsa01200	Carbon metabolism	39/1810	115/8644	0.00079283	0.02557768	gene taxa component 6	Borderline
236	Metabolism	Carbohydrate metabolism	hsa00640	Propanoate metabolism	15/1810	32/8644	0.00091078	0.02557768	gene taxa component 6	Borderline
237	Human Diseases	Cancer: specific types	hsa05220	Chronic myeloid leukemia	25/1610	76/8644	0.00203165	0.02697095	gene taxa component 4	Borderline

238	Metabolism	Metabolism of cofactors and vitamins	hsa00770	Pantothenate and CoA biosynthesis	10/1610	21/8644	0.00232112	0.02697095	gene taxa component 4	Borderline
239	Environmental Information Processing	Signal transduction	hsa04668	TNF signaling pathway	34/1610	114/8644	0.00240097	0.02697095	gene taxa component 4	Borderline
240	Organismal Systems	Immune system	hsa04062	Chemokine signaling pathway	56/1724	192/8644	0.00128148	0.02887593	gene taxa component 8	Borderline
241	Human Diseases	Infectious disease: bacterial	hsa05130	Pathogenic Escherichia coli infection	53/1610	198/8644	0.00277677	0.02910595	gene taxa component 4	Borderline
242	Human Diseases	Cancer: specific types	hsa05213	Endometrial cancer	20/1610	58/8644	0.00297262	0.02910595	gene taxa component 4	Borderline
243	Organismal Systems	Endocrine system	hsa04910	Insulin signaling pathway	39/1610	137/8644	0.00305397	0.02910595	gene taxa component 4	Borderline
244	Organismal Systems	Endocrine system	hsa04935	Growth hormone synthesis, secretion and action	35/1610	120/8644	0.00315149	0.02910595	gene taxa component 4	Borderline
245	Human Diseases	Cancer: specific types	hsa05225	Hepatocellular carcinoma	46/1610	168/8644	0.00319561	0.02910595	gene taxa component 4	Borderline
246	Cellular Processes	Cellular community - eukaryotes	hsa04530	Tight junction	47/1516	170/8644	0.00063703	0.02975995	gene taxa component 3	Borderline
247	Organismal Systems	Immune system	hsa04622	RIG-I-like receptor signaling pathway	24/1516	71/8644	0.00068074	0.02975995	gene taxa component 3	Borderline
248	Human Diseases	Cancer: specific types	hsa05210	Colorectal cancer	26/1466	86/8644	0.00161171	0.03155855	gene taxa component 1	Borderline
249	Human Diseases	Drug resistance: antineoplastic	hsa01521	EGFR tyrosine kinase inhibitor resistance	25/1610	79/8644	0.00366043	0.03162987	gene taxa component 4	Borderline
250	Human Diseases	Cancer: specific types	hsa05221	Acute myeloid leukemia	22/1610	67/8644	0.00376848	0.03174944	gene taxa component 4	Borderline
251	Organismal Systems	Endocrine system	hsa04911	Insulin secretion	29/1689	86/8644	0.00130779	0.03356461	gene taxa component 7	Borderline
252	Environmental Information Processing	Signal transduction	hsa04022	cGMP-PKG signaling pathway	49/1689	167/8644	0.00139025	0.03356461	gene taxa component 7	Borderline
253	Metabolism	Carbohydrate metabolism	hsa00562	Inositol phosphate metabolism	26/1752	73/8644	0.00161241	0.03633297	gene taxa component 9	Borderline
254	Metabolism	Metabolism of cofactors and vitamins	hsa00760	Nicotinate and nicotinamide metabolism	14/1610	37/8644	0.00475965	0.0372936	gene taxa component 4	Borderline
255	Organismal Systems	Immune system	hsa04672	Intestinal immune network for IgA production	19/1724	49/8644	0.00179309	0.03787896	gene taxa component 8	Borderline
256	Environmental Information Processing	Signal transduction	hsa04330	Notch signaling pathway	20/1466	62/8644	0.00230765	0.03876847	gene taxa component 1	Borderline
257	Metabolism	Glycan biosynthesis and metabolism	hsa00510	N-Glycan biosynthesis	18/1610	53/8644	0.00565144	0.04140292	gene taxa component 4	Borderline
258	Genetic Information Processing	Folding, sorting and degradation	hsa04120	Ubiquitin mediated proteolysis	39/1516	142/8644	0.00202343	0.04142871	gene taxa component 3	Borderline
259	Human Diseases	Drug resistance: antineoplastic	hsa01522	Endocrine resistance	29/1516	98/8644	0.00220626	0.04142871	gene taxa component 3	Borderline
260	Human Diseases	Cancer: specific types	hsa05224	Breast cancer	40/1610	147/8644	0.00640669	0.0431513	gene taxa component 4	Borderline
261	Organismal Systems	Circulatory system	hsa04270	Vascular smooth muscle contraction	37/1610	134/8644	0.00662821	0.0431513	gene taxa component 4	Borderline
262	Organismal Systems	Immune system	hsa04660	T cell receptor signaling pathway	34/1610	121/8644	0.00676687	0.0431513	gene taxa component 4	Borderline
263	Organismal Systems	Immune system	hsa04620	Toll-like receptor signaling pathway	31/1610	108/8644	0.00678641	0.0431513	gene taxa component 4	Borderline
264	Metabolism	Global and overview maps	hsa01212	Fatty acid metabolism	19/1516	57/8644	0.002861	0.04538743	gene taxa component 3	Borderline
265	Organismal Systems	Endocrine system	hsa04921	Oxytocin signaling pathway	41/1516	154/8644	0.00295421	0.04538743	gene taxa component 3	Borderline
266	Cellular Processes	Transport and catabolism	hsa04146	Peroxisome	28/1744	83/8644	0.00256579	0.04581595	gene taxa component 10	Borderline
267	Metabolism	Amino acid metabolism	hsa00280	Valine, leucine and isoleucine degradation	19/1810	48/8644	0.00244763	0.04582506	gene taxa component 6	Borderline
268	Cellular Processes	Transport and catabolism	hsa04137	Mitophagy - animal	34/1810	103/8644	0.00277638	0.04678193	gene taxa component 6	Borderline
269	Environmental Information Processing	Signaling molecules and interaction	hsa04080	Neuroactive ligand-receptor interaction	94/1689	367/8644	0.00219228	0.04939946	gene taxa component 7	Borderline

Supplementary Table 17. Functional annotation of host-microbiota interactions by SparceCCA in all stages in bulk RNA-seq validation data

	category	subcategory	ID	Description	GeneRatio	BgRatio	pvalue	FDR	component	stage
1	Genetic Information Processing	Translation	hsa03010	Ribosome	18/196	167/8644	3.68E-08	0.0000089	gene_taxa_component_8	F0
2	Cellular Processes	Cell motility	hsa04810	Regulation of actin cytoskeleton	25/305	229/8644	0.00000045	0.000123714	gene_taxa_component_10	F0
3	Human Diseases	Infectious disease: bacterial	hsa05110	Vibrio cholerae infection	8/196	50/8644	0.0000144	0.001159265	gene_taxa_component_8	F0
4	Genetic Information Processing	Replication and repair	hsa03420	Nucleotide excision repair	45411	63/8644	0.0000531	0.003503503	gene_taxa_component_2	F0
5	Metabolism	Lipid metabolism	hsa00062	Fatty acid elongation	3/33	27/8644	0.000139318	0.003529397	gene_taxa_component_4	F0
6	Metabolism	Metabolism of cofactors and vitamins	hsa00790	Folate biosynthesis	3/33	27/8644	0.000139318	0.003529397	gene_taxa_component_4	F0
7	Metabolism	Lipid metabolism	hsa01040	Biosynthesis of unsaturated fatty acids	3/33	27/8644	0.000139318	0.003529397	gene_taxa_component_4	F0
8	Genetic Information Processing	Folding, sorting and degradation	hsa04120	Ubiquitin mediated proteolysis	12/196	142/8644	0.0000872	0.005273359	gene_taxa_component_8	F0
9	Cellular Processes	Cell growth and death	hsa04110	Cell cycle	12/196	157/8644	0.0002266	0.009139549	gene_taxa_component_8	F0
10	Environmental Information Processing	Signal transduction	hsa04390	Hippo signaling pathway	12/196	157/8644	0.0002266	0.009139549	gene_taxa_component_8	F0
11	Cellular Processes	Transport and catabolism	hsa04140	Autophagy - animal	12/196	165/8644	0.000358894	0.012407483	gene_taxa_component_8	F0
12	Human Diseases	Cancer: specific types	hsa05210	Colorectal cancer	8/196	86/8644	0.000708002	0.021417067	gene_taxa_component_8	F0
13	Environmental Information Processing	Signal transduction	hsa04150	mTOR signaling pathway	11/196	156/8644	0.000816554	0.02195623	gene_taxa_component_8	F0
14	Organismal Systems	Endocrine system	hsa04910	Insulin signaling pathway	10/196	137/8644	0.001078308	0.026095051	gene_taxa_component_8	F0
15	Cellular Processes	Cell growth and death	hsa04115	p53 signaling pathway	7/196	74/8644	0.001379791	0.030355402	gene_taxa_component_8	F0
16	Genetic Information Processing	Information processing in viruses	hsa03250	Viral life cycle - HIV-1	9/305	63/8644	0.000333026	0.031883401	gene_taxa_component_10	F0
17	Human Diseases	Infectious disease: bacterial	hsa05100	Bacterial invasion of epithelial cells	10/305	77/8644	0.000347819	0.031883401	gene_taxa_component_10	F0
18	Human Diseases	Cancer: specific types	hsa05213	Endometrial cancer	6/196	58/8644	0.00191172	0.035587394	gene_taxa_component_8	F0
19	Cellular Processes	Transport and catabolism	hsa04137	Mitophagy - animal	8/196	103/8644	0.002288037	0.037964346	gene_taxa_component_8	F0
20	Cellular Processes	Transport and catabolism	hsa04145	Phagosome	10/196	152/8644	0.002353162	0.037964346	gene_taxa_component_8	F0
21	Metabolism	Global and overview maps	hsa01240	Biosynthesis of cofactors	4/33	153/8644	0.00258976	0.049205431	gene_taxa_component_4	F0
22	Genetic Information Processing	Translation	hsa03010	Ribosome	11/36	167/8644	3.98E-11	5.3E-09	gene_taxa_component_2	F1
23	Metabolism	Amino acid metabolism	hsa00310	Lysine degradation	45372	63/8644	0.000446883	0.010725188	gene_taxa_component_1	F1
24	Metabolism	Energy metabolism	hsa00190	Oxidative phosphorylation	4/33	134/8644	0.001594312	0.034011995	gene_taxa_component_10	F1
25	Genetic Information Processing	Translation	hsa03010	Ribosome	45565	167/8644	3.05E-09	0.00000015	gene_taxa_component_8	F2
26	Genetic Information Processing	Folding, sorting and degradation	hsa03050	Proteasome	4/31	46/8644	0.0000199	0.000894798	gene_taxa_component_4	F2
27	Genetic Information Processing	Chromosome	hsa03082	ATP-dependent chromatin remodeling	4/31	117/8644	0.000756403	0.00680763	gene_taxa_component_4	F2
28	Metabolism	Energy metabolism	hsa00190	Oxidative phosphorylation	4/31	134/8644	0.00125562	0.009056259	gene_taxa_component_4	F2
29	Human Diseases	Cancer: overview	hsa05208	Chemical carcinogenesis - reactive oxygen species	45442	223/8644	0.000919249	0.014634061	gene_taxa_component_9	F2
30	Organismal Systems	Environmental adaptation	hsa04714	Thermogenesis	45442	232/8644	0.001098061	0.014634061	gene_taxa_component_9	F2
31	Genetic Information Processing	Transcription	hsa03040	Spliceosome	6/39	216/8644	0.000371798	0.014963724	gene_taxa_component_2	F2
32	Human Diseases	Drug resistance: antineoplastic	hsa01523	Antifolate resistance	2/31	30/8644	0.005086632	0.022889843	gene_taxa_component_4	F2
33	Genetic Information Processing	Folding, sorting and degradation	hsa03050	Proteasome	20/1000	46/8644	4.48E-08	0.00000739	gene_taxa_component_8	F3
34	Human Diseases	Cardiovascular disease	hsa05415	Diabetic cardiomyopathy	60/1340	203/8644	0.000000223	0.0000139	gene_taxa_component_10	F3
35	Human Diseases	Cancer: overview	hsa05208	Chemical carcinogenesis - reactive oxygen species	64/1340	223/8644	0.000000289	0.0000139	gene_taxa_component_10	F3
36	Metabolism	Energy metabolism	hsa00190	Oxidative phosphorylation	43/1340	134/8644	0.00000106	0.0000444	gene_taxa_component_10	F3
37	Genetic Information Processing	Translation	hsa00970	Aminoacyl-tRNA biosynthesis	12/255	66/8644	0.000000401	0.000105797	gene_taxa_component_1	F3
38	Genetic Information Processing	Folding, sorting and degradation	hsa04141	Protein processing in endoplasmic reticulum	41/1000	170/8644	0.00000282	0.000133165	gene_taxa_component_8	F3
39	Human Diseases	Endocrine and metabolic disease	hsa04932	Non-alcoholic fatty liver disease	46/1340	155/8644	0.00000507	0.000189149	gene_taxa_component_10	F3
40	Genetic Information Processing	Translation	hsa03010	Ribosome	48/1340	167/8644	0.00000839	0.000282044	gene_taxa_component_10	F3
41	Organismal Systems	Environmental adaptation	hsa04714	Thermogenesis	60/1340	232/8644	0.0000252	0.00065247	gene_taxa_component_10	F3
42	Genetic Information Processing	Replication and repair	hsa03420	Nucleotide excision repair	23/1340	63/8644	0.0000352	0.000845331	gene_taxa_component_10	F3
43	Genetic Information Processing	Folding, sorting and degradation	hsa04120	Ubiquitin mediated proteolysis	49/1626	142/8644	0.00000586	0.001968254	gene_taxa_component_7	F3
44	Human Diseases	Infectious disease: bacterial	hsa05132	Salmonella infection	44/791	249/8644	0.0000136	0.002422525	gene_taxa_component_3	F3
45	Genetic Information Processing	Replication and repair	hsa03430	Mismatch repair	10/791	23/8644	0.0000147	0.002422525	gene_taxa_component_3	F3
46	Metabolism	Carbohydrate metabolism	hsa00562	Inositol phosphate metabolism	29/1626	73/8644	0.0000246	0.00414074	gene_taxa_component_7	F3
47	Cellular Processes	Transport and catabolism	hsa04137	Mitophagy - animal	25/1000	103/8644	0.000216524	0.005103779	gene_taxa_component_8	F3
48	Human Diseases	Infectious disease: bacterial	hsa05131	Shigellosis	47/1000	247/8644	0.000353865	0.00729847	gene_taxa_component_8	F3
49	Cellular Processes	Cell growth and death	hsa04114	Oocyte meiosis	29/1000	131/8644	0.000385362	0.00748056	gene_taxa_component_8	F3
50	Metabolism	Carbohydrate metabolism	hsa00630	Glyoxylate and dicarboxylate metabolism	6/255	30/8644	0.000203322	0.008946188	gene_taxa_component_1	F3
51	Genetic Information Processing	Folding, sorting and degradation	hsa04130	SNARE interactions in vesicular transport	14/1141	33/8644	0.0000319	0.010636161	gene_taxa_component_4	F3
52	Genetic Information Processing	Translation	hsa03013	Nucleocytoplasmic transport	24/1000	108/8644	0.001123697	0.018540993	gene_taxa_component_8	F3
53	Genetic Information Processing	Translation	hsa03015	mRNA surveillance pathway	22/1000	97/8644	0.001342185	0.021091482	gene_taxa_component_8	F3
54	Human Diseases	Infectious disease: viral	hsa05169	Epstein-Barr virus infection	48/1340	202/8644	0.001243347	0.021987616	gene_taxa_component_10	F3
55	Human Diseases	Infectious disease: bacterial	hsa05134	Legionellosis	18/1340	56/8644	0.001403845	0.023584596	gene_taxa_component_10	F3
56	Metabolism	Amino acid metabolism	hsa00280	Valine, leucine and isoleucine degradation	16/1340	48/8644	0.001660466	0.025359839	gene_taxa_component_10	F3
57	Genetic Information Processing	Replication and repair	hsa03410	Base excision repair	12/791	44/8644	0.000426196	0.028043706	gene_taxa_component_3	F3
58	Environmental Information Processing	Signal transduction	hsa04070	Phosphatidylinositol signaling system	33/1626	97/8644	0.000255516	0.028617777	gene_taxa_component_7	F3
59	Metabolism	Global and overview maps	hsa01200	Carbon metabolism	30/1340	115/8644	0.002216192	0.032375669	gene_taxa_component_10	F3
60	Cellular Processes	Transport and catabolism	hsa04144	Endocytosis	39/791	250/8644	0.000613458	0.033637966	gene_taxa_component_3	F3
61	Cellular Processes	Transport and catabolism	hsa04140	Autophagy - animal	49/1626	165/8644	0.000430057	0.036124807	gene_taxa_component_7	F3

62	Human Diseases	Drug resistance: antineoplastic	hsa01524	Platinum drug resistance	21/1340	73/8644	0.002790625	0.039068747	gene taxa component 10	F3
63	Genetic Information Processing	Transcription	hsa03040	Spliceosome	39/1000	216/8644	0.0029494	0.040769687	gene taxa component 8	F3
64	Genetic Information Processing	Folding, sorting and degradation	hsa03018	RNA degradation	18/1000	79/8644	0.00336779	0.042745022	gene taxa component 8	F3
65	Genetic Information Processing	Transcription	hsa03020	RNA polymerase	10/1000	34/8644	0.003978112	0.048621365	gene taxa component 8	F3
66	Genetic Information Processing	Folding, sorting and degradation	hsa03050	Proteasome	5/36	46/8644	0.00000114	0.0000717	gene taxa component 4	F4
67	Metabolism	Global and overview maps	hsa01240	Biosynthesis of cofactors	21/341	153/8644	0.000000536	0.000158719	gene taxa component 7	F4
68	Human Diseases	Endocrine and metabolic disease	hsa04932	Non-alcoholic fatty liver disease	45406	155/8644	0.000798904	0.00399452	gene taxa component 9	F4
69	Genetic Information Processing	Folding, sorting and degradation	hsa04120	Ubiquitin mediated proteolysis	25/602	142/8644	0.0000135	0.004207171	gene taxa component 10	F4
70	Human Diseases	Infectious disease: viral	hsa05169	Epstein-Barr virus infection	45406	202/8644	0.002131116	0.009590024	gene taxa component 9	F4
71	Metabolism	Energy metabolism	hsa00190	Oxidative phosphorylation	4/32	134/8644	0.001417923	0.012297102	gene taxa component 5	F4
72	Genetic Information Processing	Translation	hsa03013	Nucleocytoplasmic transport	19/602	108/8644	0.00014604	0.012335545	gene taxa component 10	F4
73	Organismal Systems	Environmental adaptation	hsa04714	Thermogenesis	45406	232/8644	0.003520635	0.014402597	gene taxa component 9	F4
74	Metabolism	Metabolism of cofactors and vitamins	hsa00790	Folate biosynthesis	3/39	27/8644	0.000230466	0.017515435	gene taxa component 1	F4
75	Genetic Information Processing	Folding, sorting and degradation	hsa04141	Protein processing in endoplasmic reticulum	4/32	170/8644	0.003378632	0.019596065	gene taxa component 5	F4
76	Metabolism	Metabolism of cofactors and vitamins	hsa00130	Ubiquinone and other terpenoid-quinone biosynthesis	2/39	11/8644	0.001063338	0.020203428	gene taxa component 1	F4
77	Genetic Information Processing	Translation	hsa03010	Ribosome	4/36	167/8644	0.004889565	0.030804262	gene taxa component 4	F4
78	Metabolism	Amino acid metabolism	hsa00270	Cysteine and methionine metabolism	45346	52/8644	0.009001917	0.031160481	gene taxa component 9	F4
79	Metabolism	Carbohydrate metabolism	hsa00630	Glyoxylate and dicarboxylate metabolism	2/36	30/8644	0.006817886	0.039047891	gene taxa component 4	F4
80	Genetic Information Processing	Folding, sorting and degradation	hsa03018	RNA degradation	13/460	79/8644	0.000243192	0.045263371	gene taxa component 8	F4
81	Human Diseases	Cancer: overview	hsa05203	Viral carcinogenesis	23/460	204/8644	0.000508581	0.045263371	gene taxa component 8	F4
82	Cellular Processes	Transport and catabolism	hsa04142	Lysosome	17/460	132/8644	0.000603512	0.045263371	gene taxa component 8	F4
83	Metabolism	Metabolism of cofactors and vitamins	hsa00730	Thiamine metabolism	4/246	15/8644	0.000682163	0.046241342	gene taxa component 6	F4
84	Human Diseases	Cancer: overview	hsa05208	Chemical carcinogenesis - reactive oxygen species	4/32	223/8644	0.008797267	0.046385592	gene taxa component 5	F4
